# Common variants of T-cells contribute differently to phenotypic variation in sarcoidosis

**DOI:** 10.1038/s41598-017-05754-7

**Published:** 2017-07-17

**Authors:** Natalia V. Rivera, Michael Hagemann-Jensen, Manuel A. R. Ferreira, Susanna Kullberg, Anders Eklund, Nicholas G. Martin, Leonid Padyukov, Johan Grunewald

**Affiliations:** 1 Department of Medicine, Respiratory Unit, Karolinska Institutet, Karolinska University Hospital, SE-171 76 Stockholm, Sweden; 2 Center for Molecular Medicine, Karolinska Institutet, SE-171 76 Stockholm, Sweden; 3 QIMR Berghofer Medical Research Institute, Royal Brisbane Hospital, Queensland, 4029 Australia; 4 Department of Medicine, Rheumatology Unit, Karolinska Institutet, Karolinska University Hospital, SE-171 76 Stockholm, Sweden

## Abstract

The involvement of the immune system, particularly the role of T-cells, in sarcoidosis is unclear. The existence of higher CD4+ T-cells and increased CD4/CD8 ratio may indicate a pathogenic role of T-cells in the disease. In this study, we quantified the contribution of T-cells associated variants and of CD4/CD8 ratio in sarcoidosis phenotypes, Löfgren’s syndrome (LS) and non- Löfgren’s syndrome (non-LS). We employed a polygenic-based approach using genome-wide association studies results on relative levels of T-cells in healthy individuals to measure the genetic contribution of T-cells in sarcoidosis entities. Results revealed that the genetic architecture of LS is highly influenced by genetic variants associated with CD8+ T-cells and CD4/CD8 ratio, explaining up to 7.94% and 6.49% of LS variation, respectively; whereas, the genetic architecture of non-LS is minimally influenced by T-cells, explaining a phenotypic variation of <1%. Moreover, pleiotropy assessment between T-cells and LS/non-LS associated-variants led to the discovery of highly scored pathway maps that shared common factors related to antigen presentation and T-cell regulatory mechanisms. Differences in significant polygenic scores, presence of pleiotropy, and distinct genetic factors provide further insights on how genetic variants and genes associated with relative levels of T-cell subtypes contribute differently to sarcoidosis phenotypes.

## Introduction

The involvement of the immune system, particularly T-cells homeostasis, is a strong determinant in the pathogenesis of immune-mediated diseases. Sarcoidosis is an inflammatory disease of unknown etiology driven by T-cell mechanisms, particularly by accumulation of activated CD4 T-cells in the lungs and by the formation of noncaseating epithelioid cell granulomas. When triggered by factors as yet unidentified, disease promoting determinants - antigen presenting cells (APCs) - release cytokines and other inflammatory factors, leading to a milieu that induces recruitment and activation of Th1 CD4+ T-cells and monocytes to the lungs, as well as to a local proliferation of cells. In sarcoidosis, the lung is the main affected organ and lung-compartmentalization of CD4+ T-cells is often present, revealing up to ten times as many CD4+ T-cells as the peripheral blood, thus leading to an elevated CD4/CD8 ratio as measured in broncoalveolar lavage (BAL) fluid^1^. The existence of higher CD4+ T-cells in BAL fluid results in an increased CD4/CD8 ratio (often > 3.5) and may indicate a pathogenic role of T-cells and T-cells differentiation in the disease, suggesting an immune mechanism in the pathophysiology.

Due to the disease-specific effects, it is obvious that T-cell - related phenotypes may serve as interesting intermediate traits^2, 3^, in studying the disease, with the goal of dissecting the genetic complexity of sarcoidosis. The levels of immune-related cells such as T-cells are partly heritable traits, as determined by cellular phenotype heritability^4^ and by plasticity of T-cells response^5–9^ (an active field of research).

Genome-wide association studies (GWAs) of sarcoidosis have revealed few loci of interest^10–16^. Particularly, our group performed a high-density mapping association study on two sarcoidosis phenotypes, Löfgren’s syndrome (LS) and non-Löfgren’s syndrome (non-LS), using Immunochip SNP-array, and found that each phenotype has a distinct genetic architecture with a shared genomic overlap located in the MHC class II region^17^. Interestingly, the genetic susceptibility for LS was found to be concentrated within the extended MHC region^18^, whereas for non-LS it expanded throughout the genome. However, as has been shown in many association studies, common variants do not explain the absolute heritability or causality of either sarcoidosis phenotype. Hence, the underlying genetic predisposition is expected to be explained by many common variants with small effects derived from intermediate traits or phenotypes, which can be estimated by genome-wide profiling, i.e. combining several independent variants into additive risk scores for each individual^19–21^.

In this study, genetic predictors of relative levels of T-cells (CD3+, CD4+, and CD8+) measured by flow-cytometry, and of derived CD4/CD8 ratio in peripheral blood from healthy individuals (data available from Ferreira *et al*.^22^), were used to measure the genetic contribution of T-cells subtypes in sarcoidosis entities, LS and non-LS. Specifically, our aim was to evaluate the contribution of genetic variants associated with relative levels of T-cells in healthy individuals by quantifying their susceptibility towards the phenotypic variation in sarcoidosis phenotypes. In doing so, we employed a polygenic-based modelling approach. We constructed polygenic scores using results from GWAS of relative levels of T-cells^22^ and tested their association with sarcoidosis phenotypes, LS and non-LS, respectively. We also evaluated the implication of genetic variants associated with LS and non-LS, as reported in our recent work^17^ in T-cells (CD4+ and CD8+) and CD4/CD8 in blood of the healthy and lung of the diseased (through broncoalveolar lavage fluid - BAL), as a measure of pleiotropy^23, 24^. To gain biological insights in order to identify shared pathway maps, processes networks, and/or GO processes among LS (and non-LS) and T-cells, we conducted enrichment analysis in the intersected sets between associated genetic variants of LS (and non-LS) and associated genetic variants of T-cells (and of CD4/CD8 ratio) in both healthy and diseased groups. Lastly, in significant polygenic scores, we quantified the genetic contributions of the variants associated with LS, non-LS, T-cells, LS and T-cells, and non-LS and T-cells - so as to identify patterns and relationships among these phenotypes.

Genetic variants and single nucleotide polymorphisms (SNPs) are interchangeably used throughout the manuscript.

## Results

### Study samples

**Figure 1:**
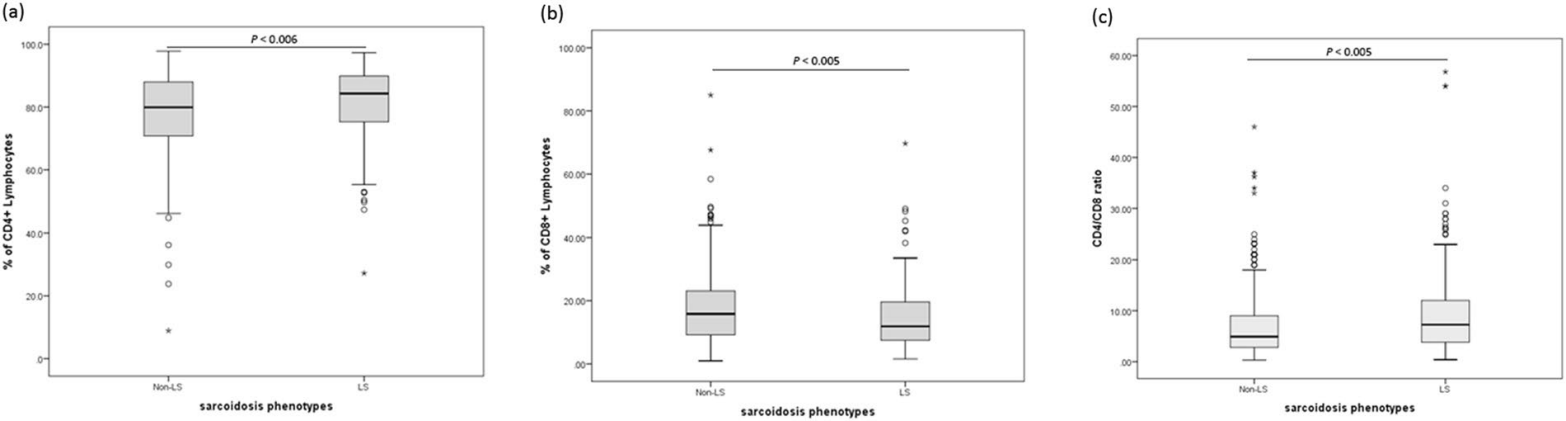
Distribution of flow cytometry measured percentages of CD4+ (**a**), and CD8+ (**b**), and CD4/CD8 ratio (**c**) among sarcoidosis phenotypes, LS and non-LS at *P* < 0.05.

We included 384 LS, 664 non-LS and 2,086 healthy controls (HC); the mean age was 38.49 ± 9.7 years in the LS vs. HC group, and 43.4 ± 12.3 years in the non-LS vs. HC group. The levels of BAL T-cells and flow cytometry results for CD3+, CD4+, CD8+ and derived CD4/CD8 ratio that were available for sarcoidosis phenotype (205 LS and 314 non-LS) are provided in Table 1. Significant differences in CD4+ and CD8+ T-cells percentages (mean, median ± SD %) and CD4/CD8 ratio between LS and non-LS were observed at *P* < 0.05, as illustrated in Fig. 1.

**Table 1:** Descriptive statistics of LS and non-LS groups.

Variable	non-LS (N = 664)			LS (N = 384)			HC (N = 2,086)	*P*-value
Gender (% of male)	57.8%			55.2%			28.4%	—
**Age, (years), mean ± SD**	43.4 ± 12.3			38.49 ± 9.7			53.4 ± 11.3	—
**BAL T-cell distribution and flow cytometry data**	**Mean**	**Median**	**SD**	**Mean**	**Median**	**SD**		***P*** **-value**
Total Cell Number	174990.02	96115	330483.3	132073.1	95645	134349.5	—	0.345
Percentage of T-cells	29.08%	28.90%	13.60%	28.17%	27.10%	15.02%	—	0.312
Count of T-cells	52724.78	23638	118425.5	39164.2	19923	70875.5	—	0.086
Percentage of T-cells CD3+	60.14%	68.15%	27.88%	65.05%	75.00%	26.58%	—	0.107
Count of T-cells CD3+	19687.79	12424	25002.606	17918.12	10196.5	22035.19	—	0.428
Percentage of T-cells CD4+	77.17%	79.90%	13.92%	80.59%	84.30%	12.67%	—	**0.006**
Count of T-cells CD4+	16003.81	9726	21707.4	15144.81%	8400	20112.89	—	0.648
Percentage of T-cells CD8+	18.32%	15.80%	12.63%	15.14%	11.90%	11.44%	—	**0.005**
Count of T-cells CD8+	3057.09	1710	3970.55	2140.82	1378.00	3111.50	—	**0.012**
CD4/CD8 ratio	6.72	4.90	5.84	9.57	7.30	9.96	—	**0.005**
CD4/CD8 ratio in LS HLA-DRB1*03 carriers	—	—	—	10.04	6.80	11.76	—	0.962
CD4/CD8 ratio in LS HLA-DRB1*03 non-carriers	—	—	—	8.40	7.20	5.77	—	—

### Polygenic scores using unpruned target sets

Cross-prediction analysis for both LS and non-LS sarcoidosis phenotypes (using an unpruned set of 118,177 SNPs) showed remarkable differences between LS and non-LS sarcoidosis phenotypes with regard to the influence of associated-variants of T-cells.

**Figure 2:**
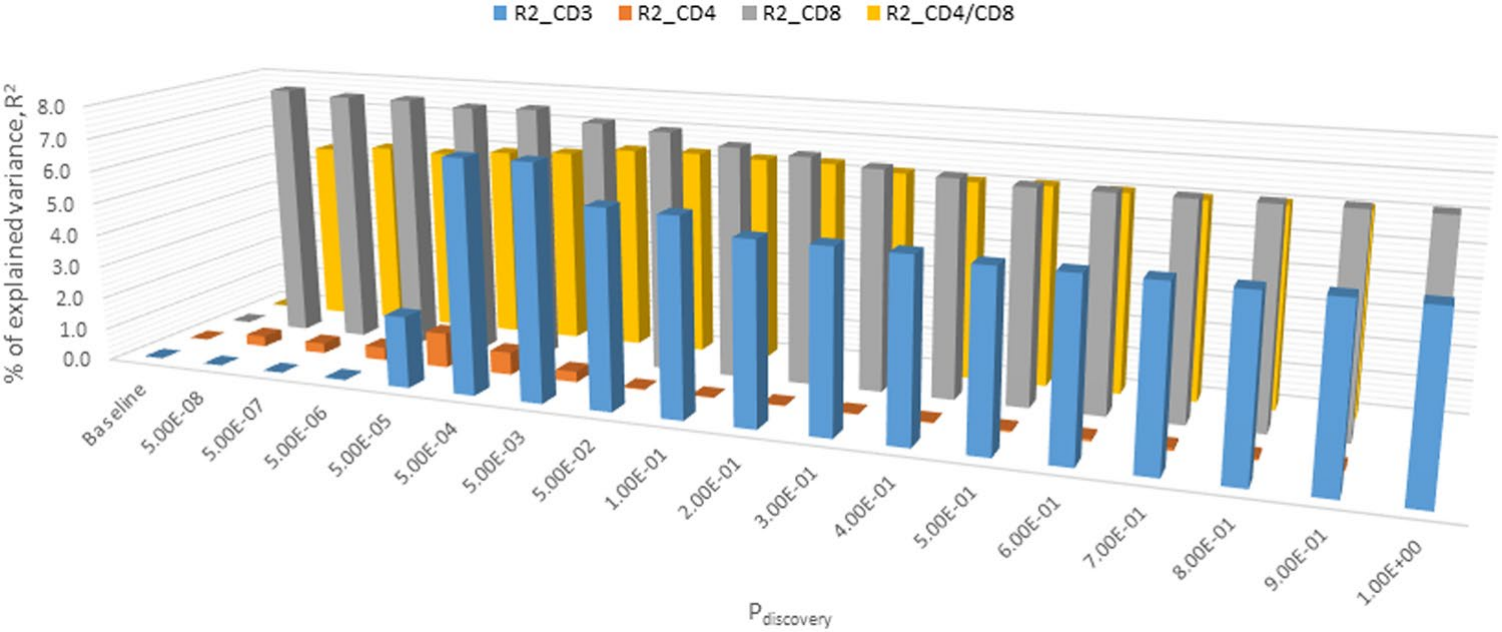
Summary of polygenic scores derived from T-lymphocyte subsets and CD4/CD8 ratio by P_discovery_ thresholds for LS (384 cases and 2,086 controls) using unpruned sets. Tabulated results for score P-values and discriminatory analysis metrics are available in Supplementary Table 1. Baseline represents the percentage of explained variance obtained from the null model adjusted for gender and age. R^2^ is the percentage of explained variance at P_discovery_ thresholds (17 sets, starting from 5.0E-8 to 1.0); R2_CD3 represents the percentage of explained variance using CD3-associated variants; R2_CD4 represents the percentage of explained variance using CD4-associated variants; R2_CD8 represents the percentage of explained variance using CD8-associated variants; and R2_CD4/CD8 represents the percentage of explained variance using CD4/CD8-associted variants.

In LS, genetic variants associated with CD3+ T-cell levels explained maximum variations of 7.13% (*SCORE*_*profile*_ *P* = 5.49 × 10^−26^ at P_discovery_ <5 × 10^−3^, Fig. 2, Supplementary Table S2) and 7.28% (*SCORE*_*profile*_ *P* = 2.02 × 10^−26^ from chromosome 6, Table 2, complete results in Supplementary Table S3). Genetic variants associated with CD8+ T-cell levels explained phenotypic variations of 7.94% (*SCORE*_*profile*_ *P* = 1.78 × 10^−28^ at P_discovery_ < 5 × 10^−8^), (Fig. 2, Supplementary Table S2) and 7.60% (*SCORE*_*profile*_ *P* = 5.62 × 10^−27^ from chromosome 6), (Table 2, complete results in Supplementary Table S3). Genetic variants associated with CD4+ T-cell levels showed maximum variations of <1.10% in both P_discovery_ (Supplementary Table S2) and chromosome (Table 2, complete results in Supplementary Table S3) sets. Genetic variants associated with CD4/CD8 ratio explained maximum variations of 6.34% (*SCORE*_*profile*_ *P* = 2.76 × 10^−22^ at P_discovery_ <5 × 10^−2^), (Fig. 2, Supplementary Table S2) and 6.49% (*SCORE*_*profile*_ *P* = 9.69 × 10^−23^ from chromosome 6), (Table 2, complete results in Supplementary Table S3).

**Table 2:** Summary of polygenic scores derived from T-cells subsets (CD4+ and CD8+) and CD4/CD8 ratio by chromosome for LS sarcoidosis (384 cases and 2,086 controls) using unpruned sets.

CHR	CD4+				CD8+				CD4/CD8 ratio			
	Nagelkerke R^2^ (%)	% of explained variance	C-statistic	SCORE P-value	Nagelkerke R^2^ (%)	% of explained variance	C-statistic	SCORE P-value	Nagelkerke R^2^ (%)	% of explained variance	C-statistic	SCORE P-value
Baseline	21.81%	0.00%	0.81	**5.48E-73**	21.81%	0.00%	0.81	**5.48E-73**	21.81%	0.00%	0.81	**5.48E-73**
1	21.85%	0.04%	0.81	5.33E-01	22.14%	0.34%	0.81	**2.76E-02**	22.03%	0.22%	0.81	7.64E-02
2	21.83%	0.02%	0.81	8.93E-01	21.83%	0.02%	0.81	9.05E-01	21.83%	0.02%	0.81	9.74E-01
3	21.93%	0.12%	0.81	2.10E-01	21.95%	0.14%	0.81	1.74E-01	21.84%	0.03%	0.81	7.14E-01
4	21.93%	0.12%	0.81	2.18E-01	21.83%	0.02%	0.81	8.77E-01	21.95%	0.14%	0.81	1.67E-01
5	21.83%	0.03%	0.81	7.44E-01	22.16%	0.35%	0.81	**2.45E-02**	21.99%	0.18%	0.81	1.20E-01
6	22.22%	0.41%	0.81	**1.61E-02**	29.41%	7.60%	0.83	**5.62E-27**	28.29%	6.49%	0.83	**9.69E-23**
7	21.83%	0.02%	0.81	9.27E-01	22.08%	0.27%	0.81	5.01E-02	21.96%	0.15%	0.81	1.55E-01
8	22.02%	0.21%	0.81	9.13E-02	22.02%	0.21%	0.81	8.64E-02	21.84%	0.03%	0.81	6.96E-01
9	21.97%	0.16%	0.81	1.44E-01	21.84%	0.03%	0.81	6.49E-01	21.83%	0.02%	0.81	9.30E-01
10	21.86%	0.06%	0.81	4.49E-01	21.88%	0.07%	0.81	3.63E-01	21.83%	0.02%	0.81	9.93E-01
11	21.88%	0.08%	0.81	3.49E-01	21.86%	0.05%	0.81	4.79E-01	21.83%	0.02%	0.81	7.71E-01
12	21.86%	0.05%	0.81	5.11E-01	22.30%	0.50%	0.81	**7.29E-03**	22.15%	0.34%	0.81	**2.90E-02**
13	21.94%	0.13%	0.81	1.88E-01	21.93%	0.12%	0.81	2.07E-01	21.83%	0.02%	0.81	8.98E-01
14	21.83%	0.02%	0.81	9.79E-01	21.85%	0.04%	0.81	5.28E-01	21.85%	0.04%	0.81	5.58E-01
15	21.84%	0.03%	0.81	6.36E-01	21.83%	0.02%	0.81	9.51E-01	21.85%	0.04%	0.81	5.89E-01
16	22.01%	0.20%	0.81	9.45E-02	21.83%	0.02%	0.81	8.04E-01	21.96%	0.15%	0.81	1.55E-01
17	22.03%	0.22%	0.81	7.73E-02	21.85%	0.04%	0.81	5.82E-01	22.15%	0.34%	0.81	**2.64E-02**
18	21.86%	0.06%	0.81	4.51E-01	21.84%	0.03%	0.81	6.83E-01	21.83%	0.02%	0.81	8.77E-01
19	21.87%	0.06%	0.81	4.35E-01	21.87%	0.06%	0.81	4.13E-01	21.85%	0.04%	0.81	5.90E-01
20	21.86%	0.06%	0.81	4.51E-01	21.83%	0.02%	0.81	9.07E-01	21.86%	0.05%	0.81	4.72E-01
21	21.87%	0.06%	0.81	4.06E-01	21.91%	0.10%	0.81	2.67E-01	22.08%	0.28%	0.81	**4.86E-02**
22	21.88%	0.07%	0.81	3.70E-01	21.87%	0.06%	0.81	4.41E-01	21.91%	0.10%	0.81	2.60E-01

CHR = chromosome; P-value is the statistical significance appertaining to the derived polygenic score profile (SCORE_profile_).

**Figure 3:**
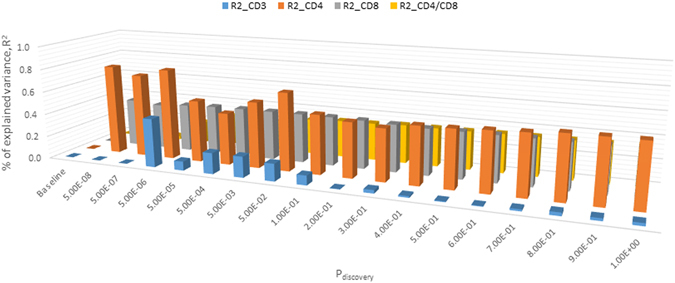
Summary of polygenic scores derived from T-cells and CD4/CD8 ratio by P_discovery_ thresholds for non-LS (664 cases and 2,086 controls) using unpruned sets. Further details are available in Supplementary Table 2. Baseline represents the percentage of explained variance obtained from the null model adjusted for gender and age. R^2^ is the percentage of explained variance at P_discovery_ thresholds (17 sets, starting from 5.0E-8 to 1.0); R2_CD3 represents the percentage of explained variance using CD3-associated variants; R2_CD4 represents the percentage of explained variance using CD4-associated variants; R2_CD8 represents the percentage of explained variance using CD8-associated variants; and R2_CD4/CD8 represents the percentage of explained variance using CD4/CD8-associated variants.

In non-LS, genetic variants associated with CD3+, CD4+ T-cell levels and CD4/CD8 ratio explained maximum variations of 0.18% (*SCORE*_*profile*_ *P* = 3.33 × 10^−2^), 0.79% (*SCORE*_*profile*_ *P* = 1.04 × 10^−6^), and 0.19% (*SCORE*_*profile*_ *P* = 3.23 × 10^−2^), respectively (Fig. 3, Supplementary Table S4) using P_discovery_ thresholds. No significant phenotypic variation was captured by genetic variants associated with CD8+ T-cell levels using P_discovery_ thresholds; however, using chromosome sets, a phenotypic variation of 1.10% was observed from chromosomes 2 and 10 (Supplementary Table S5).

Interestingly, in both LS and non-LS, the null model (defined by regression on sarcoidosis phenotype adjusted for gender and age at onset) explained a phenotypic variation of 21.81% (*P* = 5.48 × 10^−73^) for LS (Table 2) and 17.40% (*P* = 1.23 × 10^−73^) for non-LS (Table 3).

**Table 3:** Summary of polygenic scores derived from T-cells subsets (CD4+ and CD8+) and CD4:CD8 ratio by chromosome for non-LS sarcoidosis (664 cases and 2,086 controls) using unpruned sets.

CHR	CD4+				CD8+				CD4/CD8 ratio			
	Nagelkerke R^2^ (%)	% of explained variance	C-statistic	SCORE P-value	Nagelkerke R^2^ (%)	% of explained variance	C-statistic	SCORE P-value	Nagelkerke R^2^ (%)	% of explained variance	C-statistic	SCORE P-value
Baseline	17.40%	0.00%	0.73	**1.23E-73**	17.40%	0.00%	0.73	**1.23E-73**	17.40%	0.00%	0.73	**1.23E-73**
1	17.10%	0.30%	0.74	1.17E-01	17.05%	0.35%	0.73	2.14E-01	16.97%	0.43%	0.73	9.48E-01
2	17.03%	0.37%	0.74	2.93E-01	17.29%	0.11%	0.74	**1.38E-02**	17.11%	0.29%	0.74	1.03E-01
3	17.04%	0.36%	0.73	2.49E-01	16.99%	0.42%	0.73	6.10E-01	16.98%	0.43%	0.73	8.30E-01
4	17.04%	0.36%	0.74	2.51E-01	17.06%	0.35%	0.74	2.08E-01	16.99%	0.42%	0.73	6.28E-01
5	16.97%	0.43%	0.73	9.23E-01	17.17%	0.24%	0.74	5.55E-02	17.15%	0.26%	0.74	7.10E-02
6	17.81%	0.41%	0.74	**6.16E-05**	16.98%	0.43%	0.73	8.46E-01	17.10%	0.30%	0.73	1.16E-01
7	16.98%	0.43%	0.73	8.57E-01	17.07%	0.34%	0.73	1.79E-01	17.00%	0.40%	0.73	4.49E-01
8	17.01%	0.39%	0.73	3.83E-01	16.97%	0.43%	0.73	9.93E-01	16.99%	0.41%	0.73	5.76E-01
9	17.03%	0.38%	0.74	3.23E-01	16.98%	0.42%	0.73	6.46E-01	16.97%	0.43%	0.73	8.73E-01
10	16.99%	0.42%	0.73	6.32E-01	17.30%	0.11%	0.74	**1.35E-02**	17.29%	0.12%	0.74	**1.47E-02**
11	16.98%	0.43%	0.73	8.10E-01	16.99%	0.41%	0.73	5.38E-01	17.03%	0.37%	0.73	2.94E-01
12	17.01%	0.40%	0.74	4.42E-01	16.99%	0.41%	0.73	5.31E-01	17.07%	0.34%	0.74	1.80E-01
13	16.99%	0.42%	0.73	6.11E-01	17.08%	0.32%	0.74	1.52E-01	17.11%	0.29%	0.74	1.03E-01
14	17.03%	0.38%	0.74	3.06E-01	17.04%	0.36%	0.74	2.56E-01	16.98%	0.43%	0.73	8.01E-01
15	17.05%	0.36%	0.73	2.46E-01	17.00%	0.41%	0.74	5.04E-01	17.17%	0.24%	0.74	5.59E-02
16	17.08%	0.32%	0.74	1.51E-01	17.00%	0.41%	0.74	5.20E-01	17.23%	0.18%	0.74	**2.87E-02**
17	16.99%	0.42%	0.73	6.02E-01	17.06%	0.35%	0.74	2.05E-01	17.00%	0.41%	0.74	4.85E-01
18	17.04%	0.36%	0.74	2.63E-01	17.07%	0.33%	0.74	1.76E-01	16.98%	0.43%	0.73	7.85E-01
19	16.98%	0.43%	0.73	7.95E-01	16.97%	0.43%	0.73	9.93E-01	16.97%	0.43%	0.73	9.82E-01
20	16.98%	0.42%	0.74	6.96E-01	16.97%	0.43%	0.73	9.85E-01	16.99%	0.41%	0.73	5.29E-01
21	17.00%	0.40%	0.73	4.55E-01	17.03%	0.37%	0.74	2.96E-01	17.00%	0.40%	0.74	4.43E-01
22	16.99%	0.42%	0.73	6.25E-01	17.00%	0.40%	0.73	4.70E-01	17.12%	0.29%	0.74	9.97E-02

CHR = chromosome; P-value is the statistical significance appertaining to the derived polygenic score profile (SCORE_profile_).

Results from discriminatory analysis defined by the *c* statistic (equivalent to ROC metrics for dichotomous outcome) are provided, together with summary statistics for all polygenic scores computed.

In LS HLA-DRB1*03 carriers, no significant phenotypic variations were observed using P_discovery_ thresholds (Supplementary Table S6A). However, using chromosome sets, small phenotypic variations of <1% were observed with genetics variants associated with CD3+ and CD8+ T-cell levels (0.67%, *SCORE*_*profile*_ *P* = 3 × 10^−2^ from chromosome 16, and 0.58%, *SCORE*_*profile*_ *P* = 4.3 × 10^−2^ from chromosome 12, respectively), (Supplementary Table S6B). In LS HLA-DRB1*03 non-carriers, phenotypic variations of 1% (*SCORE*_*profile*_ *P* = 9.16 × 10^−3^), 1.3% (*SCORE*_*profile*_ *P* = 2.36 × 10^−3^) and 1.8% (*SCORE*_*profile*_ *P* = 3.89 × 10^−4^) were observed using P_discovery_ sets and genetic variants associated with CD4+ and CD8+ T-cell levels, and CD4/CD8 ratio, respectively (Supplementary Table S7A). Similar observations were noted using chromosome sets (Supplementary Table S7B).

### Polygenic scores using pruned target sets

Using a fewer number of SNPs in the target sets by means of LD-based pruning, the results from polygenic profiling for LS and non-LS continued to show a similar pattern of significance, as observed in the unpruned analyses.

In LS, genetic variants associated with CD3+ T-cell levels explained phenotypic variations of 0.83% (*SCORE*_*profile*_ *P* = 4.82 × 10^−4^ at P_discovery_ <5 × 10^−5^), (Supplementary Table S8A) and 2.29% (*SCORE*_*profile*_ *P* = 5.32 × 10^−9^ from chromosome 6), (Supplementary Table S9A). Genetic variants associated with CD8+ T-cell levels explained phenotypic variations of 3.89% (*SCORE*_*profile*_ *P* = 6.94 × 10^−14^ at P_discovery_ <5 × 10^−4^), (Supplementary Table S8C) and 3.81% (*SCORE*_*profile*_ *P* = 7.03 × 10^−14^ from chromosome 6), (Supplementary Table S9C). Genetic variants associated with CD4+ T-cell levels explained phenotypic variation of <0.5% using both P_discovery_ (Supplementary Table S8B) and chromosome (Supplementary Table S9B) sets. Genetic variants associated with CD4/CD8 ratio explained phenotypic variations of 5.13% (*SCORE*_*profile*_ *P* = 6.91 × 10^−18^ at P_discovery_ <5 × 10^−7^), (Supplementary Table S8D) and 3.20% (*SCORE*_*profile*_ *P* = 6.63 × 10^−12^ from chromosome 6), (Supplementary Table S9D).

In non-LS, polygenic scores from T-cells subtypes and CD4/CD8 ratio explained phenotypic variations of 0.10% using P_discovery_ (Supplementary Table S10A–D) and 0.22% chromosome (Supplementary Table S11A–D) sets.

Polygenic profiling based on genic-SNPS (n = 11,078) and intergenic-SNPS (n = 14,527) with pairwise LD defined by *r* ^*2*^ <0.25 substantiated the above observations.

In LS, genic- and intergenic-SNPs associated with CD3+ T-cell levels explained maximum phenotypic variations of 0.28% and 1.90% using P_discovery_ (Supplementary Table S8A), and 2.26% and 1.34% using chromosome (Supplementary Table S9A) sets, respectively. Genic- and intergenic-SNPs associated with CD8+ T-cell levels explained maximum phenotypic variations of 3.89% and 2.20% using P_discovery_ (Supplementary Table S8C) and 2.49% and 2.80% using chromosome (Supplementary Table S9C) sets, respectively. Genic- and intergenic-SNPs associated with CD4/CD8 ratio explained similar phenotypic variations as observed with CD8+ T-cell levels (Supplementary Table S8D and S9D). Genic- and intergenic-SNPS associated with CD4+ T-cell levels explained maximum phenotypic variations of 0.72% and 0.5% using P_dicovery_ (Supplementary Table S8B) and chromosome (Supplementary Table S9B) sets, respectively.

In non-LS, genic-SNPs associated with CD3+ T-cell levels explained a maximum phenotypic variation of 5.61% using chromosome sets (Supplementary Table S11A). Similarly, genic-SNPs associated with CD8+ T-cell levels and CD4/CD8 ratio explained a maximum phenotypic variation of 4.84% using P_discovery_ sets (Supplementary Table S10C and D). Genic-SNPs associated with CD4+ T-cell levels explained a maximum variation of 4.35% using chromosome sets (Supplementary Table S11B). Minimal phenotypic variations were observed using intergenic-SNPs associated with T-cell subtypes and CD4/CD8 ratio.

In LS HLA-DRB1*03 carriers, no significant phenotypic variations were observed using genic- and intergenic-SNPs associated with T-cell subtypes and CD4/CD8 ratio and P_discovery_ sets (Supplementary Table S12B); however, significant phenotypic variations were observed using chromosome sets (Supplementary Table S12B). In LS HLA-DRB1*03 non-carriers, small phenotypic variations were found using P_discovery_ (Supplementary Table S13A) and chromosome (Supplementary Table S13B) sets.

### Pleiotropy assessment of LS and non-LS genetic variants in susceptibility of CD4+ and CD8+ T-cells and CD4/CD8 ratio

**Figure 4:**
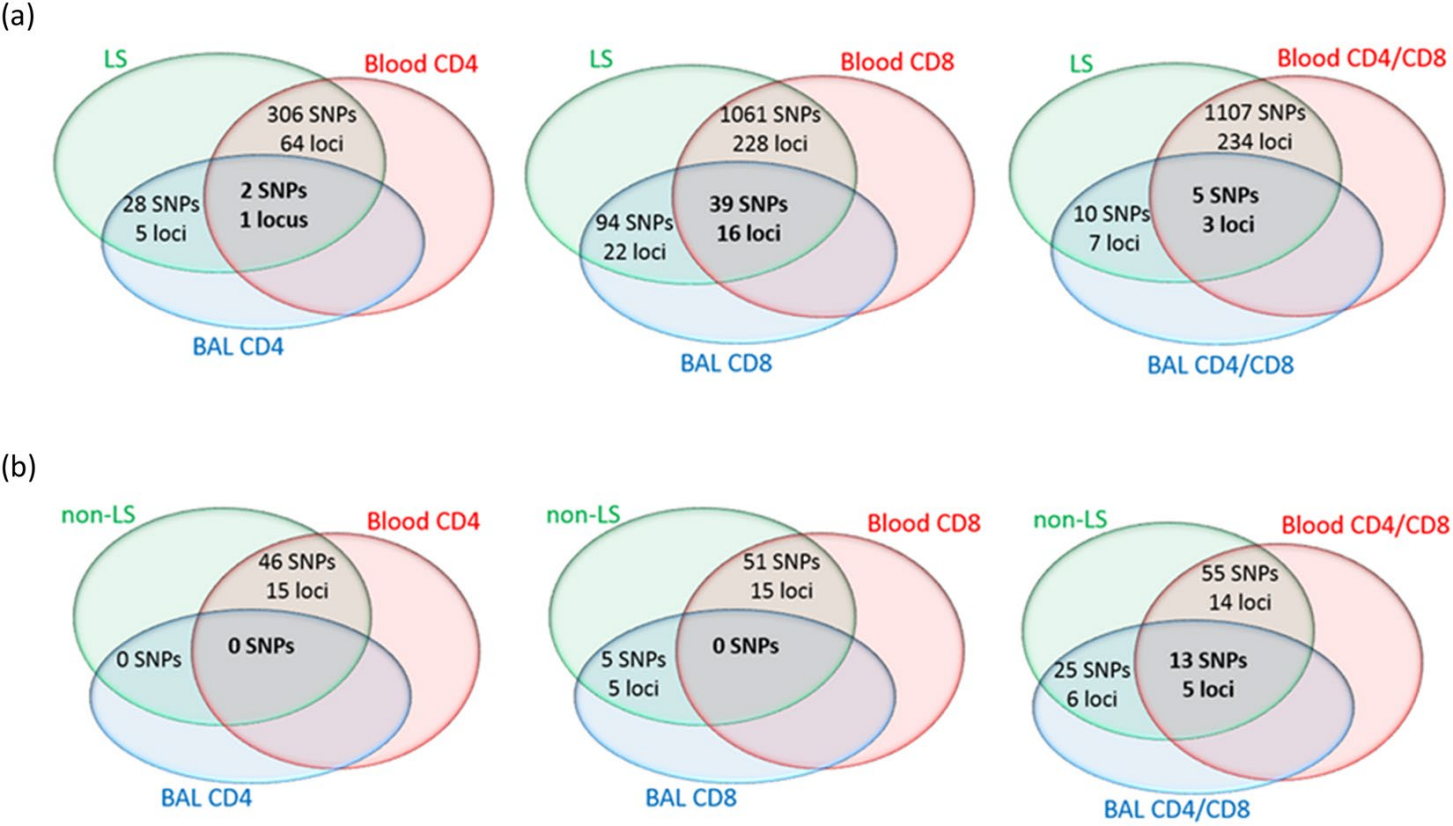
Illustration of plausibility of pleiotropy between genes of sarcoidosis phenotypes (LS and non-LS) and of T-cells. (**a**) Evaluation of 1900 LS-associated variants at meta-P < 5 × 10^-5^ (denoting 294 loci) as reported in ref. 17. (**b**) Evaluation of 98 non-LS associated variants at meta-P < 5 × 10^-5^ (denoting 23 loci) as reported in ref. 17. Genetic variants associated are grouped as follows: LS and non-LS, respectively; T-cells (CD4 and CD8) and CD4/CD8 in blood in the healthy; T-cells (CD4 and CD8) and CD4/CD8 in lung through broncoalveolar lavage fluid (BAL) in the diseased (LS and non-LS cases).

Results from assessing LS and non-LS-associated variants for susceptibility of CD4+ and CD8+ T-cells and of CD4/CD8 ratio in both healthy and diseased test groups provided considerable evidence for the plausibility of pleiotropy between susceptibility to sarcoidosis and T-cell levels. In particular, the evaluation of 1,900 LS-associated variants with meta-P < 5 × 10^−5^ as has been previously published^17^ revealed that several LS-associated variants were also associated with CD4+ and CD8+ T-cell relative counts and CD4/CD8 ratio in the blood of the healthy and in the BAL of LS cases, at a significance threshold of *P* < 0.05. The overlapping associations defined by the intersection (defined by the number of shared SNPs and common loci) between genetic variants associated with LS and T-cell subtypes in blood, and between genetic variants associated with LS and T-cell subtypes in BAL, are shown in Fig. 4(a). A complete table of the LS variants with association results of CD4+ and CD8+ T-cell relative counts and of CD4/CD8 ratio in both healthy and diseased groups is provided in Supplementary Table S14. In a similar manner, the evaluation of 98 non-LS-associated variants with meta-P < 5 × 10^−5^, as has been previously published^17^ also showed overlapping associations between CD4+ T-cell relative count in blood from healthy individuals and CD4+ and CD8+ T-cell relative counts and CD4/CD8 ratio in BAL from non-LS cases, at a significance threshold of *P* < 0.05. The intersection between genetic variants associated with non-LS and T-cell subtypes in blood and between genetic variants associated non-LS and T-cells in BAL is shown in Fig. 4(b). A complete table of the non-LS variants with association results of CD4+ and CD8+ T-cell relative counts and of CD4/CD8 ratio in both healthy and diseased test groups is provided in Supplementary Table S15.

Results from a comparison assessment, based on enrichment analysis conducted in the intersected sets, showed highly scored pathways maps, process networks, and GO processes sharing similar components among the intersected sets in question. For LS, we observed that the pathways maps, process networks, and GO processes that scored highest among intersected sets were related to immune responses corresponding to: (a) antigen presentation by MHC class I and II molecules; (b) complement pathways; and (c) immunoreuglatory mechanisms of T-lymphocyte responses and peripheral tolerance under inflammatory conditions (Supplementary Table S16A–F). The intersected sets were defined by sets of: (i) variants associated with LS and CD4+ T-cell relative count in blood; (ii) variants associated with LS and CD4+ T-cell relative count in BAL; (iii) variants associated with LS and CD8+ T-cell relative count in blood; (iv) variants associated with LS and CD8+ T-cell relative count in BAL; (v) variants associated with LS and CD4/CD8 ratio in blood; and (vi) variants associated with LS and CD4/CD8 ratio in BAL. Similarly, for non-LS, enrichment analysis conducted in the intersected sets between variants associated with non-LS and T-cells in blood and BAL revealed highly scored pathways maps, process networks, and GO processes sharing similar components related to: (a) immune response caused by antigen presentation by MHC class II molecules; (b) immune response due to HMGB1/TLR signaling; and (c) T-cell regulation mechanisms (Supplementary Table S17A–F).

### Quantifying genetic contributions in significant polygenic scores

In the LS phenotype (Table 4), using pruned SNPs, the genetic contributions in significant polygenic scores were: from 3.3% to 12.5% by variants associated with LS; from 4.5% to 6.8% by variants associated with CD4+ and CD8+ T-cell relative counts and CD4/CD8 ratio in the healthy; and from 0.2% to 1.8% by variants associated with both LS and T-cell relative counts. Using pruned genic-SNPs, the genetic contributions were: from 4.1% to 11.6% by variants associated with LS; from 4.3% to 7.0% by variants associated with CD4+ and CD8+ T-cell relative counts and CD4/CD8 ratio; and from 0.2% and 1.5% by variants associated with both LS and T-cell relative counts. Using pruned intergenic-SNPs, the genetic contributions were: from 1.9% to 13% by variants associated with LS; from 3.9% to 7.5% by variants associated with T-cell relative counts and CD4/CD8 ratio; and up to 2.1% by variants associated with both LS and T-cell relative counts.

**Table 4:** Summary of genetic contributions by LS and T-cells (CD4+ and CD8+) and CD4: CD8 ratio in significant polygenic scores derived using pruned sets.

	CHR	CD4 all-SNPs			CD4 genic-SNPs			CD4 intergenic-SNPs		
Num. of SNP predictors	5	—			414			—		
LS-assoc, CD4-assoc, both, (%)		—	—	—	4.11%	5.31%	0.24%	—	—	—
Num. of SNP predictors	6	—			—			829		
LS-assoc, CD4-assoc, both, (%)		—	—	—	—	—	—	13.03%	6.63%	2.05%
Num. of SNP predictors	19	450						201		
LS-assoc, CD4-assoc, both, (%)		3.33%	6.22%	0.22%				2.99%	7.46%	0.50%
Num. of SNP predictors	22	—			150			—		
LS-assoc, CD4-assoc, both, (%)		—	—	—	6.00%	6.67%	1.33%	—	—	—
		**CD8 all-SNPs**			**CD8 genic-SNPs**			**CD8 intergenic-SNPs**		
Num. of SNP predictors	1	—			—			880		
LS-assoc, CD8-assoc, both, (%)		—	—	—	—	—	—	3.86%	4.55%	0.34%
Num. of SNP predictors	4	—			—			705		
LS-assoc, CD8-assoc, both, (%)		—	—	—	—	—	—	4.54%	4.54%	0.14%
Num. of SNP predictors	6	1373			544			829		
LS-assoc, CD8-assoc, both, (%)		12.45%	6.77%	1.82%	11.58%	6.99%	1.47%	13.03%	6.63%	2.05%
Num. of SNP predictors	7	995			468			—		
LS-assoc, CD8-assoc, both, (%)		5.53%	4.52%	0.20%	4.06%	4.27%	0.21%	—	—	—
Num. of SNP predictors	21	—			—			155		
LS-assoc, CD8-assoc, both, (%)		—	—	—	—	—	—	4.52%	3.87%	0.65%
		**CD4/CD8 all-SNPs**			**CD4/CD8 genic-SNPs**			**CD4/CD8 intergenic-SNPs**		
Num. of SNP predictors	6	1373			544			829		
LS-assoc, CD4/CD8-assoc, both, (%)		12.45%	6.77%	1.82%	11.58%	6.99%	1.47%	13.03%	6.63%	2.05%
Num. of SNP predictors	9	—			342			—		
LS-assoc, CD4/CD8-assoc, both, (%)		—	—	—	4.09%	5.56%	0.58%	—	—	—
Num. of SNP predictors	12	—			—			463		
LS-assoc, CD4/CD8-assoc, both, (%)		—	—	—	—	—	—	1.94%	5.18%	0.00%
Num. of SNP predictors	18	—			—			346		
LS-assoc, CD4/CD8-assoc, both, (%)								3.76%	5.78%	0.00%
Num. of SNP predictors	19	450			—			—		
LS-assoc, CD4/CD8-assoc, both, (%)		3.33%	6.22%	0.22%	—	—	—	—	—	—
Num. of SNP predictors	21	243			—			155		
LS-assoc, CD4/CD8-assoc, both, (%)		3.70%	4.94%	0.41%	—	—	—	4.52%	3.87%	0.65%

In the non-LS phenotype (Table 5), using pruned SNPs, the genetic contributions in significant polygenic scores were: from 2.4% to 8.2% by variants associated with non-LS; from 3.4% to 6.2% by variants associated with T-cells relative counts; and <1% by variants associated with both non-LS- and T-cell relative counts. Using pruned genic-SNPs, the genetic contributions were: from 1.5% to 4.2% by variants associated with non-LS; from 5.4% to 5.6% by variants associated with T-cell relative counts; and <0.5% by variants associated with both non-LS and T-cell relative counts. Likewise, using pruned intergenic-SNPs, the genetic contributions were: from 2.2% to 10.3% by variants associated with non-LS; from 3.9% to 7.5% by variants associated with T-cell relative counts; and <0.6% by variants associated with both non-LS and T-cell relative counts.

**Table 5:** Summary of genetic contributions by non-LS and T-cells (CD4+ and CD8+) and CD4:CD8 ratio in significant polygenic scores derived using pruned sets.

	CHR	CD4 all-SNPs			CD4 genic-SNPs			CD4 intergenic-SNPs		
Num. of SNP predictors	2	1652			671			—		
Non-LS-assoc, CD4-assoc, both, (%)		4.00%	5.02%	0.36%	4.17%	5.37%	0.45%	—	—	—
Num. of SNP predictors	5	1192			—			—		
Non-LS-assoc, CD4-assoc, both, (%)		3.69%	4.95%	0.34%	—	—	—	—	—	—
Num. of SNP predictors	9	—			342			—		
Non-LS-assoc, CD4-assoc, both, (%)		—	—	—	1.46%	5.56%	0.29%	—	—	—
Num. of SNP predictors	12	871			—			—		
Non-LS-assoc, CD4-assoc, both, (%)		3.90%	4.94%	0.80%	—	—	—	—	—	—
Num. of SNP predictors	19	450			—			201		
Non-LS-assoc, CD4-assoc, both, (%)		5.78%	6.22%	0.00%	—	—	—	3.48%	7.46%	0.00%
		**CD8 all-SNPs**			**CD8 genic-SNPs**			**CD8 intergenic-SNPs**		
Num. of SNP predictors	2	1652			671			—		
Non-LS-assoc, CD8-assoc, both, (%)		4.00%	5.02%	0.36%	4.17%	5.37%	0.45%	—	—	—
Num. of SNP predictors	7	995			—			527		
Non-LS-assoc, CD8-assoc, both, (%)		4.02%	4.82%	0.40%	—	—	—	4.36%	5.12%	0.57%
Num. of SNP predictors	14	617			—			—		
Non-LS-assoc, CD8-assoc, both, (%)		4.86%	3.40%	0.49%	—	—	—	—	—	—
Num. of SNP predictors	16	665						365		
Non-LS-assoc, CD8-assoc, both, (%)		2.41%	4.96%	0.45%				3.84%	6.85%	0.00%
		**CD4/CD8 all-SNPs**			**CD4/CD8 genic-SNPs**			**CD4/CD8 intergenic-SNPs**		
Num. of SNP predictors	9	—			—			457		
Non-LS-assoc, CD4/CD8-assoc, both, (%)		—	—	—	—	—	—	2.19%	7.22%	0.22%
Num. of SNP predictors	12	871			—			—		
Non-LS-assoc, CD4/CD8-assoc, both, (%)		3.90%	4.94%	0.80%	—	—	—	—	—	—
Num. of SNP predictors	20	—			—			283		
Non-LS-assoc, CD4/CD8-assoc, both, (%)		—	—	—	—	—	—	4.24%	4.59%	0.00%
Num. of SNP predictors	21	243			—			155		
Non-LS-assoc, CD4/CD8-assoc, both, (%)		8.23%	4.94%	0.00%	—	—	—	10.32%	3.87%	0.00%

## Discussion

Genetic profiling of common variants associated with T-cells provides substantial evidence that variants associated with T-cell relative counts are tightly implicated in the genetic structure of sarcoidosis, and that the cumulative effect of these variants is distributed differently in sarcoidosis phenotypes, LS and non-LS.

In particular, our results demonstrate that the genetic architecture of LS is highly and distinctly influenced by susceptibility to CD3+ T-cells levels and particularly to CD8+ T-cell levels, which explained phenotypic variations of 7.28% and 7.94%, respectively. Also, the susceptibility to the derived CD4/CD8 ratio showed to be a significant genetic contributor, as it explained a phenotypic variation of 6.49%. Surprisingly, the genetic contribution of CD4+ T-cell levels explained only 1.07% of the LS variation. Additionally, further analyses on the genetic contribution from T-cell levels revealed that most of the LS explained variation was captured by both genic- and intergenic-SNPs located on chromosome 6. This is in agreement with our recent discovery about the genetics of LS^17^, in which the susceptibility to LS clustered within the extended MHC region^25^. Considering the effect of HLA-DBR1*03 in LS with regard to good prognosis, showed no significant phenotypic variation in LS HLA-DRB1*03 carriers, suggesting a plausible interaction between HLA-DRB1*03 and variants associated with T-cell levels, particularly with CD8+ T-cells. In the LS HLA-DRB1*03 non-carriers, on the other hand, the findings of few significant phenotypic variations explained by the genetics of T-cells suggests plausible interactions between other HLA*-DRB1* alleles and variants associated with T-cell levels. These results, in addition to highlighting a significant genetic influence of CD8+ T-cell levels and *HLA-DRB1* in LS, also suggest that the increased numbers and activation of CD4+ T-cells found in the BAL of LS patients may be due to interactions between the genes of CD8+ and CD4+ T-cells with *HLA-DRB1* and other genetic and/or environmental factors. This observation strengthens previously reported data on antigen presentation in the pathogenesis of sarcoidosis^26^.

Interestingly, in non-LS, the susceptibility to CD3+, CD4+, and CD8+ T-cell levels and of CD4/CD8 ratio is shown to have minimal effect on explaining its phenotypic variation, which was found to be <1%. This finding suggests that common genetic variants of T-cell levels and of CD4/CD8 ratio have marginal influence in the disease architecture of non-LS, and therefore other genetic factors are more likely to be involved.

Results using fewer numbers of genetic variants by means of LD-based SNP pruning also revealed analogous effects for explaining the phenotypic variation of sarcoidosis phenotypes (LS and non-LS). In agreement with our findings using the unpruned datasets, common variants associated with T-cell levels and CD4/CD8 ratio are shown to have strong effects in the disease architecture of LS and weak effects in the disease architecture of non-LS. Moreover, partitioning the genome by variant type (i.e., genic- and intergenic-SNPs) also revealed similar results for explaining the phenotypic variation of LS and non-LS. That is, while both genic- and intergenic-SNPs associated with T-cell levels and of CD4/CD8 ratio are important genetic factors for explaining the phenotypic variation of LS, only genic-SNPs seem to be influential for non-LS.

Through this methodology, we have been able to quantify the influence of the susceptibility of T-cell levels (i.e. T-cells genes) in sarcoidosis, particularly in two of its main phenotypes, LS and non-LS. Polygenic profiling is an approach that captures the strength of a relationship between a genetic variant and a phenotype by weighting the contribution of the susceptible allele (usually the SNP effect-size estimated by GWAS) and aggregating it into a profile score^19, 27–29^. Hence, polygenic profiles are often additive summaries of genetic susceptibility from a set of genetic variants selected by a method of choice (e.g., P_discovery_, chromosome, variant type, and so on) and are used to construct polygenic scores under the assumption that each selected variant makes an additive contribution to the phenotype. This assumption essentially infers that gene interactions and epistatic effects may be captured by taking into account a number of selected variants that may be interacting with each other under an additive hypothesis. Keeping this in mind, in the non-LS phenotype, whose disease architecture appears to be multifactorial and has a separate genetic susceptibility^17^ compared to LS, polygenic profiling of T-cell levels and CD4/CD8 ratio explained on average <1% of the phenotypic variation by genic- and intergenic-SNPs and <5.6% by genic-SNPs, suggesting that the accrued weighted effect by genic- and intergenic-SNPs is decreased, probably due to contradictory variant effects, and hence contributing minimally to the missing variation of non-LS. In the LS phenotype, on the other hand, the weighted accrued effect contributed by both genic- and intergenic variants complemented each other, exhibiting a cumulative strong effect towards the phenotypic variation of LS, and thus suggesting epistatic mechanisms between genic and intergenic regions. In particular, since genetic variants associated with LS are clustered within chromosome 6, it is plausible that interactions may occur with *in-cis* mechanisms under an additive hypothesis, which may explain the observed considerable phenotypic variation. In line with this hypothesis, molecular functions of T-cells related to immunomodulatory mechanisms regulating inflammatory processes and formation of granulomas may be tightly coupled with genetic mechanisms of LS.

Assessment of LS and non-LS associated variants (as reported in ref. 17) in GWAS of T-cell levels, and CD4/CD8 ratio in blood of the healthy (as reported in ref. 22), and in lung of LS and non-LS patients (unpublished data), as a measure of pleiotropy provided further insights about T-cell genes in sarcoidosis. Enrichment analysis based on overlapping variants (defined by intersected sets) between sarcoidosis phenotypes (LS and non-LS) and T-cell subtypes in both blood and in lung revealed exciting findings, which highlighted immune responses related to antigen presentation by MHC molecules class I and II, signaling pathways, such as complement pathway and TRL signaling pathway, and T-cell regulatory mechanisms – all which are relevant to pathogenesis of sarcoidosis, a disease alleged to be T-cell driven.

Furthermore, quantification of genetic contributions in significant polygenic scores in LS and non-LS phenotypes across T-cell subtypes showed that genetic variants and genomic loci (genic and intergenic) involved in T-cell susceptibility in the healthy contribute differently to the phenotypic variation of LS and non-LS, respectively. Similarly, genetic contributions within each phenotype may be determined by specific gene sets from LS and T-cells, and from non-LS and T-cells, respectively.

The major strength of this study is that sarcoidosis cases in both LS and non-LS and healthy controls are well-characterized and that we were able to inspect genetic variants associated with CD3+, CD4+, and CD8+ T-cell levels and CD4/CD8 ratio in the genetics of LS and non-LS (as tagged by the Immunochip SNP-array). The methodologies adopted for estimating polygenic profiles are hypothesis-free, which enabled us to capture genetic contributions from high and relevant variants at the genome-wide level. One limitation of our study is that we did not have complete data for BAL T-cells differential counts and flow cytometry data for all cases who were genotyped on the Immunochip, nor for healthy controls - which could have allowed us to conduct further specific analyses. Another limitation is that the calculated cumulative phenotypic variation of LS and of non-LS may be somewhat lower than the actual value, given that we used SNP genetic effects (betas also known as weights) of T-cells derived from blood in healthy individuals. Also, polygenic scores explaining the phenotypic variation of LS and non-LS are to some degree limited to SNPs tagged by the Immunochip, thus the contribution of other common variants that are available in denser SNP-arrays may be missing. A third limitation is that we did not examine SNPs interactions or *gene x gene* interactions following identification of genetic variants in significant score profiles, since a greater sample size is required to conduct such analysis.

In summary, inspecting the effect of common variants associated with CD3+, CD4+, and CD8+ T-cell levels and CD4/CD8 ratio demonstrated that the phenotypic variation of LS is substantially explained by genetic variants associated with T-cell levels, particularly those associated with the CD8+ type, a novel finding of this study. Another interesting finding is that, regardless of the number of variants or variant type (genic or intergenic), the phenotypic variation of LS continued to be proportionately explained. This observation is in an agreement with the polygenic score profiling for height, for example, conducted by Yang *et al*., which showed that the resulting estimate of proportion of variance explained does not depend on the number of SNPs and that remaining phenotypic variation may be due to incomplete linkage-disequilibrium (LD) between casual variants and genotyped SNPs^30^. With regards to the genetics of non-LS, the susceptibility of T-cell levels and CD4/CD8 ratio was shown to have minimal effect on the phenotypic variation when all common variants were assessed; however, this was not the case when genic-SNPs associated with CD8+ T-cell levels were considered - these showed a significant phenotypic variation. The combined effect of age and gender that explained 21.81% and 17.40% of the phenotypic variation in LS and non-LS, respectively, also provides new insights regarding the influence of these factors into the genetics of sarcoidosis, as the phenotypic variation of LS and non-LS were more than twice as high, thus suggesting that age and gender are strong contributors in mechanisms of disease in sarcoidosis.

Pleiotropy assessment between genetic variants associated with sarcoidosis and T-cell levels led to the discovery of highly scored pathway maps that shared common factors related to antigen presentation by MHC molecules class I and II, and to T-cell regulatory mechanisms, highlighting T-cell genes contribution in sarcoidosis, which is distinctly scored in each phenotype, LS and non-LS. We also demonstrated that genetic variants associated with CD8+ T-cell levels and CD4/CD8 ratio are key determinants in sarcoidosis, particularly in LS. Thus, genes associated with CD8+ T-cells may explain the increased CD4/CD8 ratio in the disease. This evidence of pleiotropy between the genetics of T-cells and sarcoidosis provides a deeper insight into the mechanisms of the disease. Further investigations for pinpointing genetic contributors by T-cell genes should be performed. These should include quantifying the transcriptome of the lung and blood by RNA-sequencing, as well as targeted DNA-sequencing of chromosome 6, which would give deeper insights about the role of T-cell genes in sarcoidosis. Additionally, RNA-sequencing of specific T-cells, such as CD4+ and CD8+, and perhaps other T-cell subpopulations such as regulatory T-cells (Treg), would be beneficial for identifying expressed genes in sarcoidosis phenotypes. Investigation of gene *x gene* interactions, and chromatic immunoprecipitation sequencing (CHIP-seq) to understand the functional relationship between transcription factors and target genes located in the extended MHC region, are also recommended.

## Methods

### Discovery set

Using summary genome-wide association results of T-cell levels (CD3+, CD4+, and CD8+) and of derived CD4/CD8 ratio, as conducted in ref. 22, polygenic scores were constructed. In brief: GWA analyses of T-lymphocyte subsets in peripheral blood were performed using a gene discovery set of 2,538 adolescent twins from 1,089 Australian families sampled from the general population, whose DNA was genotyped with Illumina 610-Quad BeadChip (529,721 SNPs). The genotype data set was then extended by imputation to 2.3 million SNPs using data from the CEU HapMap samples (phase I+II, release 22, build 36) and MACH software^31^. Analyses of T-lymphocyte subsets were performed with AutoPrep (coulter) and direct fluorochromome-conjugated monoclonal antibodies to CD3, CD4, and CD8 antigens (Coulter). Subsequent analyses were performed on an Epics 753 cytofluorograph (Coulter) with the use of standardized control samples and machine settings. The CD4/CD8 ratio was calculated from the relative levels of CD4+ and CD8+ T-cells measured by flow-cytometry. GWA analyses were conducted on each T-lymphocyte subset and derived CD4/CD8 ratio, and were adjusted for age and sex and normalized with an inverse-normal transformation. Further details of the study are available elsewhere (22).

### Target sets

Two target sets of sarcoidosis were used for T-lymphocyte polygenic profiling: Löfgren’s syndrome (LS) consisting of 2,470 subjects (384 cases and 2,086 controls); and non-Löfgren’s syndrome (non-LS) consisting of 2,750 subjects (664 cases and 2,086 controls). Further details of the phenotypic description for cases and controls are available in ref. 17. Briefly, in all sarcoidosis cases i.e., LS and non-LS, bronchoscopy with bronchoalveolar lavage (BAL) was performed as previously described^32^. Diagnosis was in accordance with criteria established by the World Association of Sarcoidosis and Other Granulomatous Disorders (WASOG)^33^. Specifically, these included typical clinical and radiographic manifestations, findings at bronchoscopy with BAL including an elevated CD4/CD8 ratio, and, if required, positive biopsies, as well as exclusion of other diagnoses. Löfgren syndrome was defined as an acute onset disease, usually with fever, chest radiographic findings of bilateral hilar lymphadenopathy, sometimes with pulmonary infiltrates, and erythema nodosum and/or bilateral ankle arthritis.

Samples from the target set were genotyped using Immunochip SNP-array and quality control filtering was performed as described in ref. 17. Briefly, SNPs that had call rate <98%, minor allele frequency (MAF) <2%, and Hardy-Weinberg Equilibrium (HWE) *P* < 1 × 10^−5^ (tested only in controls) were excluded from the analysis. Individuals with missing genotype <98% were also removed. QC filtering output 118,177 SNPs from the Immunochip SNP-array. High-density mapping association analyses for LS and non-LS were performed and are available elsewhere^17^.

### Molecular analysis in sarcoidosis phenotypes using BAL cells

*For the BAL handling*. The differential cell counts were based on the May-Grünwald and Giemsa staining. *Flow cytometry analysis*. Due to the extensive time period for collecting and acquiring the samples, different generations of flow cytometers were used for acquisition, as well as different conjugated antibodies for human CD3, CD4 and CD8 cells. In general, after BAL handling, cells *ex-vivo* were stained with Pacific Blue conjugated mouse anti-Human CD3 (UCHT1), FITC conjugated mouse anti-Human CD3 (UCHT1), APC-H7 mouse anti-Human CD4 (SK3), Pe-Cy5 mouse anti-Human CD4 (SK3, MT310), AmCyan mouse anti-Human CD8 (SK1), PE mouse anti-Human CD8 (SK1). Cells were washed twice in PBS+1% Ab serum (Sigma) before staining on ice for 20 min in the dark. Flow cytometry data acquisition was carried out on BD FACSCalibur, BD FACS Canto II (BD Biosciences) using Cellquest, BD FACSDiva software. Analysis was performed via FlowJo X (Treestar) software. Ratios were calculated from percentage CD4 and CD8 expressing CD3+ cells in 1,399 patients with verified diagnosis of sarcoidosis (488 LS and 911 non-LS).

### Statistical analysis in sarcoidosis phenotypes using BAL cell types

Descriptive statistics i.e., mean, median ± SD of BAL T-cells (CD3+CD4+ and CD8+) and derived CD4/CD8 ratio were analyzed in each LS and non-LS group, respectively. No bronchoscopy procedure was performed in healthy controls and therefore no data on BAL were available. BAL fluid relative levels of T-cells distributions were assessed by a non-parametric method using Mann-Whitney U test. BAL derived CD4/CD8 ratio was dichotomized by a threshold of 3.5 and evaluated using likelihood ratio test. All statistical analyses were performed using R software.

### Calculation of polygenic scores in sarcoidosis phenotypes

**Figure 5:**
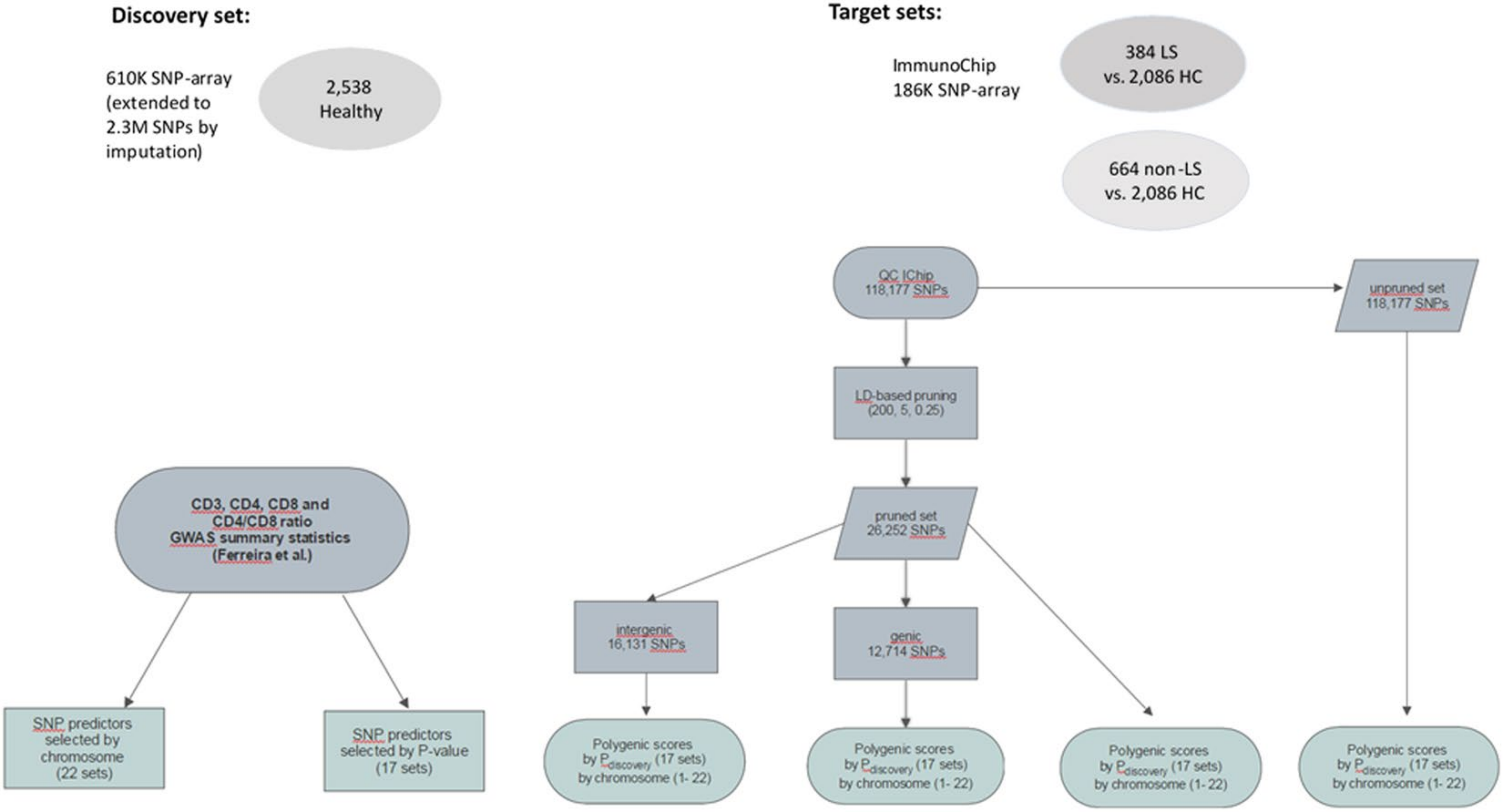
Genetic risk score profiling methodology flow chart.

Polygenic scores were calculated in quality-controlled sarcoidosis target sets (LS and non-LS) for the different sets by P-value called P_discovery_ and by chromosome. This was done by multiplying the number of risk alleles per SNP (0, 1 or 2) by the effect size from the discovery set, and summed them across all the SNPs in that specific cluster using the score function implemented in PLINK software. The significance of the polygenic score was tested in the likelihood ratio test (LTR) by using it as a predictor in a logistic regression model, with gender and age at onset as covariates, and was set to *P* < 0.05. The explained variance (defined by the pseudo Nagelkerke R^2^) was calculated as the difference between the null model adjusted for both gender and age at onset and the alternative model adjusted for polygenic score, gender and age at onset, which were regressed on the dependent variable, either LS or non-LS. Additionally, discriminative ability was assessed by the concordance (*c*) statistic. For binary outcome, the *c* statistic is identical to the area under the receiving operating characteristic curve (ROC)^34^. Both logistic regression and discriminative analyses were performed using the *rms* package in R software. For each corresponding P_*discovery*_ and chromosome sets, a polygenic score (SCORE_profile_) was derived as described above. Specifically, in each target set, an unpruned (118,177 SNPS) and a pruned (26,252 SNPs) set were employed in order to explain the proportion of phenotypic variation. Pruning was performed using linkage-disequilibrium (LD) information and by selecting SNPs recursively based on a LD *r* ^*2*^ < 0.25 within a sliding window of 200 SNPs, with a step-size of 5 SNPs at a time, as implemented in PLINK. Additionally, in order to examine the individual contribution of the type of genetic variant, genic SNPs (defined as variants located in introns, exons, and UTR regions) and intergenic SNPs (defined as variants located between genes) were selected and used for calculating polygenic scores, respectively. Polygenic profiles were then calculated by P_*discovery*_ thresholds and by chromosomes using genic-SNPs (pruned set: n = 12,714) and intergenic-SNPs (pruned set: n = 16,131), respectively. LD information between pairs of genic and intergenic SNPs was obtained by computing LD values using pairwise LD measures, as implemented in PLINK. Only SNPs based on pairwise LD *r* ^*2*^ <0.25 were selected for analysis. A graphical illustration of the design and methodology of the study is shown in Fig. 5.

Additionally, to examine the effect of HLA-DRB1*03 in the LS group, given its strong association with good disease course, polygenic scores in LS HLA-DRB1*03 subgroups, adjusted for gender and age at onset, were also computed.

### Pleiotropy assessment of LS and non-LS genetic variants in susceptibility of T-cells (CD4 and CD8) and CD4/CD8 ratio

To determine whether genetic variants of sarcoidosis phenotypes, LS and non-LS, were implicated in genetic susceptibility of T-cells, as a measure of pleiotropy, we evaluated LS and non-LS genetic variants from the meta-analysis association assessment, as reported in ref. 17. Genetic variants with meta-P value < 5 × 10^−5^ were selected and were looked up in genome-wide association studies of CD4 and CD8 relative counts and CD4/CD8 ratio measured in blood of the healthy, as reported in (22), and in association studies of CD4 and CD8 relative counts and CD4/CD8 ratio measured in BAL of the diseased, LS and non-LS patients (unpublished data). Association studies on BAL CD4+ and CD8+ T-cell relative counts and CD4/CD8 ratio were performed using linear regression under an additive model, adjusted for gender and age at onset, in PLINK. Significance threshold for an association was set to *P* < 0.05. Additionally, to gain biological insights in order to identify pathway maps, processes networks, and/or gene ontology (GO) processes shared by LS (and non-LS) and T-cells, we conducted enrichment analysis in intersected sets encapsulating genetic variants associated with LS, non-LS, T-cells (CD4+ and CD8+ relative counts) and CD4/CD8 ratio in both healthy and diseased groups. Enrichment analysis was performed using MetaCore™ integrated software (Thomson Reuters, New York, USA) and establishing a significance threshold based on false discovery rate (FDR) <0.05.

### Quantifying genetic contributions in significant polygenic scores

To determine the genetic contribution by variants associated with sarcoidosis (LS and non-LS) and T-cells (CD4+ and CD8+ relative counts) and CD4/CD8 ratio among significant polygenic scores, we identified genetic variants in each chromosome and P_discovery_ sets and obtained the total number of predictors that were used to calculate the polygenetic score. We then linked each SNP predictor to the association P-value calculated in the Immunochip analysis of sarcoidosis phenotypes, as described in ref. 17, and in the GWAS of T-lymphocytes, as described in ref. 22, respectively. For each significant polygenic score, the genetic contribution was calculated as the percentage, i.e., dividing the total number of genetic variants with association P-value < 0.05 by the total number of SNP predictors and multiplying by 100%.

## Electronic supplementary material

Supplementary Information
