# Supplementary material for: Common variants of T-cells contribute differently to phenotypic variation in sarcoidosis
Source: Sci Rep. 2017 Jul 17;7:5623. doi: 10.1038/s41598-017-05754-7 (PMC5514043; doi:10.1038/s41598-017-05754-7)
Supplement: Supplementary file 1 — Supplementary Information [file 41598_2017_5754_MOESM1_ESM.pdf]

Supplementary Information

Common variants of T-cells contribute differently to phenotypic variation in sarcoidosis

Running Title: Genes of T-cells are implicated in sarcoidosis

Natalia V Rivera[1,2,*], Michael Hagemann-Jensen[1,2], Manuel A.R. Ferreira[3], Susanna Kullberg[1], Anders Eklund[1], Nicholas G. Martin[3], Leonid Padyukov[2,4], Johan Grunewald[1,2,*]

[1]Department of Medicine, Respiratory Unit, Karolinska Institutet, Karolinska University Hospital, SE-171 76 Stockholm, Sweden

[2]Center for Molecular Medicine, Karolinska Institutet, SE-171 76 Stockholm, Sweden

[3]QIMR Berghofer Medical Research Institute, Royal Brisbane Hospital, Queensland 4029, Australia

[4]Department of Medicine, Rheumatology Unit, Karolinska Institutet, Karolinska University Hospital, SE-171 76 Stockholm, Sweden

SUPPLEMENTARY FIGURES

Supplementary Figure 1 Representative figure from a non-LS sarcoidosis patient of the gating strategy applied for flow cytometry analysis of BAL CD4 and CD8 T-lymphocytes.

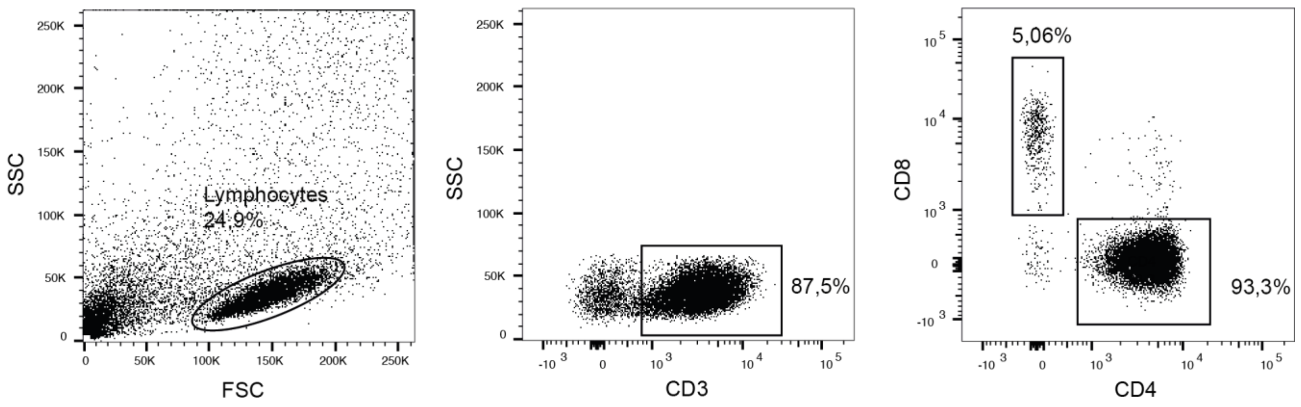

SUPPLEMENTARY TABLES

Supplementary Table S1 Summary of GWAs SNPs mapped to SNPs in ImmunoChip for both LS and non-LS phenotypes by $P_{discovery}$ (a) and chromosome (b) sets used in unpruned and pruned analyses

(a) By $P_{discovery}$ sets

(b) By chromosome sets

Supplementary Table S2 Summary of polygenic scores derived from T-lymphocytes subsets (a) – (c) and CD4/CD8 ratio (d) by $P_{discovery}$ thresholds for LS sarcoidosis (384 cases and 2,086 controls) using unpruned sets.

(a) CD3+

(b) CD4+

(c) CD8+

(d) CD4/CD8

Supplementary Table S3 Summary of polygenic scores derived from T-lymphocytes subsets (a) – (c) and CD4/CD8 ratio (d) by chromosome for LS sarcoidosis (384 cases and 2,086 controls) using unpruned sets.

(a) CD3+

(b) CD4+

(c) CD8+

(d) CD4/CD8

Supplementary Table S4 Summary of polygenic scores derived from T-lymphocytes subsets (a) – (c) and CD4:CD8 ratio (d) T-lymphocytes by $P_{discovery}$ thresholds for non-LS sarcoidosis (664 cases and 2,086 controls) using unpruned sets.

(a) CD3+

(b) CD4+

(c) CD8+

(d) CD4/CD8

Supplementary Table S5 Summary of polygenic scores derived from T-lymphocytes subsets (a) – (c) and CD4:CD8 ratio (d) T-lymphocytes by chromosome for non-LS sarcoidosis (664 cases and 2,086 controls) using unpruned sets.

(a) CD3+

(b) CD4+

(c) CD8+

(d) CD4/CD8

Supplementary Table S6 Summary of polygenic scores derived from T-lymphocytes subsets and CD4/CD8 ratio by $P_{discovery}$ thresholds (a) and by chromosome (b) for LS HLA-DRB1*03 carriers (253 cases and 512 controls) using unpruned sets.

(a) By $P_{discovery}$

(b) By chromosome

Supplementary Table S7 Summary of polygenic scores derived from T-lymphocytes subsets and CD4/CD8 ratio by $P_{discovery}$ thresholds (a) and by chromosome (b) for LS HLA-DRB1*03 non-carriers (131 cases and 1,574 controls) using unpruned sets.

(a) By $P_{discovery}$

(b) By chromosome

Supplementary Table S8 Summary of polygenic scores derived from T-lymphocytes subsets (a) – (c) and CD4/CD8 ratio (d) by $P_{discovery}$ thresholds for LS sarcoidosis (384 cases and 2,086 controls) using pruned sets.

(a) CD3+

(b) CD4+

(c) CD8+

(d) CD4/CD8

Supplementary Table S9 Summary of polygenic scores derived from T-lymphocytes subsets (a) – (c) and CD4/CD8 ratio (d) by chromosome for LS sarcoidosis (384 cases and 2,086 controls) using pruned sets. 9

(a) CD3+

(b) CD4+

(c) CD8+

(d) CD4/CD8

Supplementary Table S10 Summary of polygenic scores derived from T-lymphocytes subsets (a)-(c) and CD4:CD8 ratio (d) T-lymphocytes by $P_{discovery}$ thresholds for non-LS sarcoidosis (664 cases and 2,086 controls) using pruned sets.

(a) CD3+

(b) CD4+

(c) CD8+

(d) CD4/CD8

Supplementary Table S11 Summary of polygenic scores derived from T-lymphocytes subsets (a)-(c) and CD4:CD8 ratio (d) T-lymphocytes by chromosome for non-LS sarcoidosis (664 cases and 2,086 controls) using pruned sets.

(a) CD3+

(b) CD4+

(c) CD8+

(d) CD4/CD8

Supplementary Table S12 Summary of polygenic scores derived from T-lymphocytes subsets and CD4/CD8 ratio by $P_{discovery}$ thresholds (a) and by chromosome (b) for LS HLA-DRB1*03 carriers (253 cases and 512 controls) using pruned sets.

(a) By $P_{discovery}$

(b) By chromosome

Supplementary Table S13 Summary of polygenic scores derived from T-lymphocytes subsets and CD4/CD8 ratio by $P_{discovery}$ thresholds (a) and by chromosome (b) for LS HLA-DRB1*03 non-carriers (131 cases and 1,574 controls) using pruned sets.

(a) By $P_{discovery}$

(b) By chromosome

Supplementary Table S14 Pleiotropy assessment of genetic variants of LS (ref. 17) in T-lymphocytes (CD4 and CD8 relative counts) and CD4/CD8 ratio in both blood of the healthy (ref. 22) and BAL of LS cases (unpublished data)

Supplementary Table S15 Pleiotropy assessment of genetic variants of non-LS (ref. 17) in T-lymphocytes (CD4 and CD8 relative counts) and CD4/CD8 ratio in blood of the healthy (ref. 22) and BAL of non-LS cases (unpublished data)

Supplementary Table S16 Enrichment analysis in intersected sets between LS and T-cells (CD4+, CD8+, and CD4/CD8 ratio) associated variants in healthy and diseased groups

(a) LS-associated variants in blood CD4 of healthy individuals

(b) LS-associated variants in BAL CD4 T-cells of LS cases

(c) LS-associated variants in blood CD8 T-cells of healthy individuals

(d) LS-associated variants in BAL CD8 T-cells of LS cases

(e) LS-associated variants in blood CD4/CD8 ratio of healthy individuals

(f) LS-associated variants in BAL CD4/CD8 ratio of LS cases

Supplementary Table S17 Enrichment analysis conducted in intersected sets between genetic variants of non-LS and of T-cells (CD4+, CD8+, CD4/CD8 ratio) in healthy and diseased groups

(a) Non-LS-associated variants in blood CD4 of healthy individuals

(b) Non-LS-associated variants in BAL CD4 T-cells of non-LS cases

(c) Non-LS-associated variants in blood CD8 T-cells of healthy individuals

(d) Non-LS-associated variants in BAL CD8 T-cells of non-LS cases

(e) Non-LS-associated variants in blood CD4/CD8 ratio of healthy individuals

(f) Non-LS-associated variants in BAL CD4/CD8 ratio of non-LS cases

Supplemetnary Table S1. Summary of GWAs SNPs mapped to SNPs in ImmunoChip for both LS and non-LS phenotypes by $P_{discovery}$ (A) and chromosome (B) sets used in unpruned and pruned analyses

(A) by $P_{discovery}$ sets

			unpruned SNPs		pruned SNPs			unpruned SNPs		pruned SNPs			unpruned SNPs		pruned SNPs			unpruned SNPs		pruned SNPs	
		**Number of GWAS SNP predictors**	Number of mapped SNPs	Number of mapped alleles	Number of mapped SNPs	Number of mapped alleles	**Number of GWAS SNP predictors**	Number of mapped SNPs	Number of mapped alleles	Number of mapped SNPs	Number of mapped alleles	**Number of GWAS SNP predictors**	Number of mapped SNPs	Number of mapped alleles	Number of mapped SNPs	Number of mapped alleles	**Number of GWAS SNP predictors**	Number of mapped SNPs	Number of mapped alleles	Number of mapped SNPs	Number of mapped alleles
$P_{discovery}$ set	P-value threshold	**CD3**					**CD4**					**CD8**					**CD4/CD8**				
1	5.0E-08	0	0	0	0	0	18	10	8	0	0	287	169	101	9	6	1,041	535	313	17	9
2	5.0E-07	0	0	0	0	0	27	15	11	0	0	471	263	161	11	7	1,388	732	423	22	13
3	5.0E-06	7	4	2	1	1	45	27	19	1	1	783	415	240	13	9	1,862	1,000	579	34	21
4	5.0E-05	170	5	3	2	2	322	72	47	2	2	1,267	613	348	24	15	2,422	1,289	742	39	25
5	5.0E-04	1,615	113	77	13	10	1,853	227	127	12	4	2,922	961	568	43	27	4,411	1,755	906	68	36
6	5.0E-03	13,104	690	392	94	58	12,840	747	413	104	65	15,427	1,836	1,042	132	77	16,833	2,824	1,655	180	93
7	5.0E-02	122,006	4,424	2,435	983	620	119,543	4,982	2,754	973	533	125,603	5,707	3,157	995	531	125,495	6,880	3,777	1,026	536
8	1.0E-01	241,572	8,096	4,472	1,949	1,048	239,929	8,698	4,791	1,912	1,035	243,751	9,499	5,286	1,972	1,044	242,915	10,530	5,803	1,964	1,055
9	2.0E-01	471,193	15,196	8,464	3,734	2,034	468,197	15,725	8,662	3,788	2,026	465,959	16,180	8,935	3,759	2,004	467,187	17,026	9,381	3,729	1,966
10	3.0E-01	705,683	22,094	12,269	5,644	3,067	705,032	22,929	12,692	5,706	3,038	702,190	23,047	12,732	5,651	3,039	701,960	24,236	13,359	5,627	2,969
11	4.0E-01	941,712	29,059	16,057	7,545	4,063	941,341	29,853	16,508	7,606	4,043	935,317	29,920	16,500	7,511	4,044	937,243	31,343	17,265	7,525	4,009
12	5.0E-01	1,177,443	36,129	19,918	9,430	5,019	1,177,238	37,103	20,483	9,489	5,061	1,171,802	37,101	20,448	9,423	5,081	1,170,365	38,053	20,942	9,343	4,985
13	6.0E-01	1,410,197	43,095	23,787	11,299	6,014	1,413,484	44,015	24,310	11,397	6,089	1,405,816	43,697	24,036	11,253	6,043	1,406,229	44,778	24,678	11,249	5,998
14	7.0E-01	1,642,259	50,243	27,731	13,164	7,006	1,646,684	50,848	28,057	13,240	7,089	1,641,354	50,674	27,916	13,159	7,053	1,640,305	51,498	28,390	13,100	6,968
15	8.0E-01	1,875,398	57,244	31,594	15,065	8,005	1,877,778	57,469	31,662	15,068	8,053	1,875,037	57,572	31,751	15,047	8,045	1,877,860	58,351	32,171	15,078	8,026
16	9.0E-01	2,109,168	64,460	35,573	16,946	9,027	2,109,931	64,529	35,550	16,905	9,028	2,108,716	64,370	35,479	16,888	9,005	2,110,954	65,005	35,831	16,951	9,021
17	1.0E+00	2,342,667	71,582	39,444	18,850	10,022	2,342,916	71,538	39,421	18,826	10,015	2,342,770	71,598	39,461	18,836	10,028	2,342,625	71,606	39,459	18,831	10,017

(B) By chromosome sets

		unpruned SNPs		pruned SNPs	
CHR set	**Number of GWAS SNP predictors CDx**	Number of mapped SNPs	Number of mapped alleles	Number of mapped SNPs	Number of mapped alleles
1	177,447	7,022	3,883	1,621	865
2	205,537	7,543	4,175	1,652	889
3	161,979	4,505	2,521	1,180	634
4	150,720	2,703	1,465	1,106	687
5	156,457	4,923	2,721	1,192	639
6	170,214	9,492	6,299	1,373	752
7	132,211	3,099	1,617	995	497
8	138,442	3,398	1,889	988	514
9	112,700	2,566	1,369	799	407
10	127,415	3,826	2,128	984	549
11	120,218	3,196	1,774	964	516
12	114,712	3,582	1,955	871	459
13	96,187	1,819	1,008	604	341
14	77,159	2,349	1,318	617	312
15	64,862	1,316	710	593	307
16	63,395	2,370	1,310	665	359
17	51,641	1,767	980	517	270
18	70,396	1,384	767	536	280
19	31,469	1,344	772	450	261
20	57,272	1,545	792	508	263
21	31,533	722	392	243	130
22	29,971	1,128	632	301	160

CDx refers to CD3+, CD4, and CD8+ T-cells

Supplementary Table S2. Summary of polygenic scores derived from T-lymphocytes subsets and CD4/CD8 ratio by $P_{discovery}$ thresholds for LS (384 cases and 2,086 controls) using unpruned sets.

	CD3 all-SNPs				CD4 all-SNPs				CD8 all-SNPs				CD4/CD8 all-SNPs			
$P_{discovery}$	Nagelkerke R^2 (%)	% explained variance	C-statistic	SCORE P	Nagelkerke R^2 (%)	% explained variance	C-statistic	SCORE P	Nagelkerke R^2 (%)	% explained variance	C-statistic	SCORE P	Nagelkerke R^2 (%)	% explained variance	C-statistic	SCORE P
Baseline	21.81%	0.00%	0.81	**5.48E-73**	21.81%	0.00%	0.81	**5.48E-73**	21.81%	0.00%	0.81	**5.48E-73**	21.81%	0.00%	0.81	**5.48E-73**
5.0E-08	–	–	–	–	22.13%	0.32%	0.81	**3.60E-02**	29.75%	7.94%	0.83	**1.78E-28**	27.46%	5.65%	0.83	**6.88E-20**
5.0E-07	–	–	–	–	22.12%	0.31%	0.81	**4.02E-02**	29.64%	7.83%	0.83	**3.09E-28**	27.62%	5.81%	0.83	**2.02E-20**
5.0E-06	21.84%	0.04%	0.81	5.91E-01	22.21%	0.40%	0.81	**1.88E-02**	29.66%	7.85%	0.83	**3.12E-28**	27.58%	5.77%	0.83	**2.28E-20**
5.0E-05	23.99%	2.19%	0.81	**1.67E-08**	22.88%	1.07%	0.81	**1.19E-04**	29.54%	7.73%	0.83	**9.03E-28**	27.75%	5.94%	0.83	**6.07E-21**
5.0E-04	28.91%	7.10%	0.83	**5.85E-26**	22.50%	0.69%	0.81	**1.54E-03**	29.62%	7.81%	0.83	**7.68E-28**	27.87%	6.06%	0.83	**2.33E-21**
5.0E-03	28.94%	7.13%	0.83	**5.49E-26**	22.13%	0.32%	0.81	**3.16E-02**	29.32%	7.51%	0.83	**9.07E-27**	28.10%	6.30%	0.83	**4.42E-22**
5.0E-02	27.79%	5.99%	0.83	**5.62E-22**	21.84%	0.03%	0.81	6.39E-01	29.19%	7.38%	0.83	**3.49E-26**	28.15%	6.34%	0.83	**2.76E-22**
1.0E-01	27.74%	5.93%	0.83	**1.04E-21**	21.83%	0.02%	0.81	9.08E-01	28.86%	7.05%	0.83	**3.89E-25**	28.10%	6.29%	0.83	**4.15E-22**
2.0E-01	27.25%	5.44%	0.82	**4.38E-20**	21.83%	0.02%	0.81	7.89E-01	28.72%	6.91%	0.83	**1.08E-24**	28.11%	6.30%	0.83	**3.70E-22**
3.0E-01	27.24%	5.43%	0.82	**5.08E-20**	21.83%	0.02%	0.81	8.49E-01	28.51%	6.70%	0.83	**5.05E-24**	27.97%	6.16%	0.83	**1.03E-21**
4.0E-01	27.20%	5.39%	0.82	**6.64E-20**	21.84%	0.03%	0.81	6.99E-01	28.39%	6.59%	0.83	**1.13E-23**	27.86%	6.05%	0.83	**2.40E-21**
5.0E-01	27.08%	5.27%	0.82	**1.73E-19**	21.86%	0.05%	0.81	4.97E-01	28.28%	6.48%	0.83	**2.55E-23**	27.89%	6.08%	0.83	**1.88E-21**
6.0E-01	27.08%	5.27%	0.82	**1.83E-19**	21.88%	0.07%	0.81	3.85E-01	28.29%	6.48%	0.83	**2.42E-23**	27.86%	6.05%	0.83	**2.44E-21**
7.0E-01	27.07%	5.26%	0.82	**1.86E-19**	21.89%	0.08%	0.81	3.41E-01	28.28%	6.47%	0.83	**2.66E-23**	27.81%	6.00%	0.83	**3.35E-21**
8.0E-01	27.03%	5.23%	0.82	**2.48E-19**	21.88%	0.07%	0.81	3.59E-01	28.30%	6.49%	0.83	**2.33E-23**	27.83%	6.02%	0.83	**2.90E-21**
9.0E-01	27.04%	5.24%	0.82	**2.31E-19**	21.88%	0.07%	0.81	3.88E-01	28.33%	6.52%	0.83	**1.86E-23**	27.85%	6.04%	0.83	**2.59E-21**
1.0E+00	27.04%	5.23%	0.82	**2.33E-19**	21.87%	0.07%	0.81	3.95E-01	28.34%	6.53%	0.83	**1.73E-23**	27.85%	6.04%	0.83	**2.64E-21**

Supplementary Table S3. Summary of polygenic scores derived from T-lymphocytes subsets and CD4/CD8 ratio by chromsomes for LS (384 cases and 2,086 controls) using unpruned sets.

	CD3 all-SNPs				CD4 all-SNPs				CD8 all-SNPs				CD4/CD8 all-SNPs			
CHR	Nagelkerke R^2 (%)	% explained variance	C-statistic	SCORE P	Nagelkerke R^2 (%)	% explained variance	C-statistic	SCORE P	Nagelkerke R^2 (%)	% explained variance	C-statistic	SCORE P	Nagelkerke R^2 (%)	% explained variance	C-statistic	SCORE P
Baseline	21.81%	0.00%	0.81	**5.48E-73**	21.81%	0.00%	0.81	**5.48E-73**	21.81%	0.00%	0.81	**5.48E-73**	21.81%	0.00%	0.81	**5.48E-73**
1	21.92%	0.11%	0.81	2.31E-01	21.85%	0.04%	0.81	5.33E-01	22.14%	0.34%	0.81	**2.76E-02**	22.03%	0.22%	0.81	7.64E-02
2	21.83%	0.02%	0.81	9.14E-01	21.83%	0.02%	0.81	8.93E-01	21.83%	0.02%	0.81	9.05E-01	21.83%	0.02%	0.81	9.74E-01
3	21.92%	0.12%	0.81	2.26E-01	21.93%	0.12%	0.81	2.10E-01	21.95%	0.14%	0.81	1.74E-01	21.84%	0.03%	0.81	7.14E-01
4	21.84%	0.03%	0.81	6.28E-01	21.93%	0.12%	0.81	2.18E-01	21.83%	0.02%	0.81	8.77E-01	21.95%	0.14%	0.81	1.67E-01
5	21.98%	0.17%	0.81	1.31E-01	21.83%	0.03%	0.81	7.44E-01	22.16%	0.35%	0.81	**2.45E-02**	21.99%	0.18%	0.81	1.20E-01
6	29.09%	7.28%	0.83	**2.02E-26**	22.22%	0.41%	0.81	**1.61E-02**	29.41%	7.60%	0.83	**5.62E-27**	28.29%	6.49%	0.83	**9.69E-23**
7	21.88%	0.08%	0.81	3.49E-01	21.83%	0.02%	0.81	9.27E-01	22.08%	0.27%	0.81	5.01E-02	21.96%	0.15%	0.81	1.55E-01
8	22.08%	0.28%	0.81	**4.81E-02**	22.02%	0.21%	0.81	9.13E-02	22.02%	0.21%	0.81	8.64E-02	21.84%	0.03%	0.81	6.96E-01
9	21.94%	0.13%	0.81	1.97E-01	21.97%	0.16%	0.81	1.44E-01	21.84%	0.03%	0.81	6.49E-01	21.83%	0.02%	0.81	9.30E-01
10	21.89%	0.08%	0.81	3.44E-01	21.86%	0.06%	0.81	4.49E-01	21.88%	0.07%	0.81	3.63E-01	21.83%	0.02%	0.81	9.93E-01
11	21.89%	0.09%	0.81	3.11E-01	21.88%	0.08%	0.81	3.49E-01	21.86%	0.05%	0.81	4.79E-01	21.83%	0.02%	0.81	7.71E-01
12	21.84%	0.03%	0.81	6.78E-01	21.86%	0.05%	0.81	5.11E-01	22.30%	0.50%	0.81	**7.29E-03**	22.15%	0.34%	0.81	**2.90E-02**
13	21.88%	0.07%	0.81	3.69E-01	21.94%	0.13%	0.81	1.88E-01	21.93%	0.12%	0.81	2.07E-01	21.83%	0.02%	0.81	8.98E-01
14	21.83%	0.03%	0.81	7.39E-01	21.83%	0.02%	0.81	9.79E-01	21.85%	0.04%	0.81	5.28E-01	21.85%	0.04%	0.81	5.58E-01
15	21.83%	0.02%	0.81	7.87E-01	21.84%	0.03%	0.81	6.36E-01	21.83%	0.02%	0.81	9.51E-01	21.85%	0.04%	0.81	5.89E-01
16	21.97%	0.16%	0.81	1.41E-01	22.01%	0.20%	0.81	9.45E-02	21.83%	0.02%	0.81	8.04E-01	21.96%	0.15%	0.81	1.55E-01
17	21.90%	0.09%	0.81	3.04E-01	22.03%	0.22%	0.81	7.73E-02	21.85%	0.04%	0.81	5.82E-01	22.15%	0.34%	0.81	**2.64E-02**
18	21.84%	0.04%	0.81	6.05E-01	21.86%	0.06%	0.81	4.51E-01	21.84%	0.03%	0.81	6.83E-01	21.83%	0.02%	0.81	8.77E-01
19	21.88%	0.07%	0.81	3.57E-01	21.87%	0.06%	0.81	4.35E-01	21.87%	0.06%	0.81	4.13E-01	21.85%	0.04%	0.81	5.90E-01
20	21.85%	0.04%	0.81	5.74E-01	21.86%	0.06%	0.81	4.51E-01	21.83%	0.02%	0.81	9.07E-01	21.86%	0.05%	0.81	4.72E-01
21	21.83%	0.02%	0.81	8.05E-01	21.87%	0.06%	0.81	4.06E-01	21.91%	0.10%	0.81	2.67E-01	22.08%	0.28%	0.81	**4.86E-02**
22	21.83%	0.02%	0.81	9.32E-01	21.88%	0.07%	0.81	3.70E-01	21.87%	0.06%	0.81	4.41E-01	21.91%	0.10%	0.81	2.60E-01

Supplementary Table S4. Summary of polygenic scores derived from T-lymphocytes subsets and CD4/CD8 ratio by $P_{discovery}$ thresholds for non-LS (664 cases and 2,086 controls) using unpruned sets.

	CD3 all-SNPs				CD4 all-SNPs				CD8 all-SNPs				CD4/CD8 all-SNPs			
$P_{discovery}$	Nagelkerke R^2 (%)	% explained variance	C-statistic	SCORE P	Nagelkerke R^2 (%)	% explained variance	C-statistic	SCORE P	Nagelkerke R^2 (%)	% explained variance	C-statistic	SCORE P	Nagelkerke R^2 (%)	% explained variance	C-statistic	SCORE P
Baseline	17.40%	0.00%	0.73	**1.23E-73**	17.40%	0.00%	0.73	**1.23E-73**	17.40%	0.00%	0.73	**1.23E-73**	17.40%	0.00%	0.73	**1.23E-73**
5.0E-08	–	–	–	–	18.19%	0.79%	0.74	**1.04E-06**	16.98%	0.42%	0.73	6.53E-01	17.51%	0.10%	0.74	**1.41E-03**
5.0E-07	–	–	–	–	18.13%	0.73%	0.74	**1.95E-06**	17.01%	0.40%	0.73	4.36E-01	17.38%	0.03%	0.74	**5.64E-03**
5.0E-06	16.98%	0.43%	0.73	7.41E-01	18.20%	0.79%	0.74	**9.89E-07**	16.99%	0.42%	0.73	6.25E-01	17.21%	0.19%	0.74	**3.23E-02**
5.0E-05	17.48%	0.08%	0.74	**1.97E-03**	17.95%	0.54%	0.74	**1.41E-05**	16.98%	0.43%	0.73	8.09E-01	17.13%	0.28%	0.74	8.78E-02
5.0E-04	17.22%	0.18%	0.74	**3.33E-02**	17.86%	0.45%	0.74	**4.05E-05**	16.97%	0.43%	0.73	9.44E-01	17.10%	0.31%	0.73	1.24E-01
5.0E-03	17.22%	0.18%	0.74	**3.31E-02**	17.98%	0.58%	0.74	**1.27E-05**	16.97%	0.43%	0.73	9.13E-01	17.12%	0.28%	0.74	9.38E-02
5.0E-02	17.25%	0.15%	0.74	**2.36E-02**	18.08%	0.68%	0.74	**4.70E-06**	16.97%	0.43%	0.73	9.94E-01	17.09%	0.31%	0.74	1.37E-01
1.0E-01	17.32%	0.08%	0.74	**1.16E-02**	17.92%	0.52%	0.74	**2.33E-05**	16.98%	0.43%	0.73	8.45E-01	17.08%	0.33%	0.74	1.58E-01
2.0E-01	17.40%	0.00%	0.74	**5.13E-03**	17.88%	0.48%	0.74	**3.34E-05**	16.98%	0.42%	0.73	7.21E-01	17.08%	0.33%	0.74	1.62E-01
3.0E-01	17.43%	0.03%	0.74	**3.74E-03**	17.86%	0.45%	0.74	**4.38E-05**	16.99%	0.41%	0.73	5.78E-01	17.07%	0.34%	0.74	1.87E-01
4.0E-01	17.42%	0.01%	0.74	**4.32E-03**	17.91%	0.50%	0.74	**2.74E-05**	17.00%	0.41%	0.73	4.88E-01	17.07%	0.34%	0.74	1.84E-01
5.0E-01	17.41%	0.01%	0.74	**4.63E-03**	17.91%	0.50%	0.74	**2.70E-05**	17.00%	0.41%	0.73	4.93E-01	17.07%	0.34%	0.73	1.83E-01
6.0E-01	17.40%	0.00%	0.74	**5.17E-03**	17.92%	0.52%	0.74	**2.39E-05**	17.00%	0.41%	0.73	4.90E-01	17.06%	0.34%	0.73	1.92E-01
7.0E-01	17.42%	0.02%	0.74	**4.21E-03**	17.93%	0.52%	0.74	**2.18E-05**	17.00%	0.41%	0.73	4.90E-01	17.06%	0.34%	0.73	1.96E-01
8.0E-01	17.43%	0.03%	0.74	**3.71E-03**	17.95%	0.55%	0.74	**1.78E-05**	17.00%	0.40%	0.73	4.82E-01	17.06%	0.34%	0.73	1.94E-01
9.0E-01	17.43%	0.02%	0.74	**3.95E-03**	17.95%	0.55%	0.74	**1.78E-05**	17.00%	0.40%	0.73	4.79E-01	17.06%	0.34%	0.73	1.95E-01
1.0E+00	17.43%	0.02%	0.74	**3.88E-03**	17.95%	0.54%	0.74	**1.80E-05**	17.00%	0.40%	0.73	4.81E-01	17.06%	0.34%	0.73	1.96E-01

Supplementary Table S5. Summary of polygenic scores derived from T-lymphocytes subsets and CD4/CD8 ratio by chromosomes for non-LS (664 cases and 2,086 controls) using unpruned sets.

	CD3 all-SNPs				CD4 all-SNPs				CD8 all-SNPs				CD4/CD8 all-SNPs			
CHR	Nagelkerke R^2 (%)	% explained variance	C-statistic	SCORE P	Nagelkerke R^2 (%)	% explained variance	C-statistic	SCORE P	Nagelkerke R^2 (%)	% explained variance	C-statistic	SCORE P	Nagelkerke R^2 (%)	% explained variance	C-statistic	SCORE P
Baseline	17.40%	0.00%	0.73	**1.23E-73**	17.40%	0.00%	0.73	**1.23E-73**	17.40%	0.00%	0.73	**1.23E-73**	17.40%	0.00%	0.73	**1.23E-73**
1	17.07%	0.34%	0.73	1.81E-01	17.10%	0.30%	0.74	1.17E-01	17.05%	0.35%	0.73	2.14E-01	16.97%	0.43%	0.73	9.48E-01
2	17.22%	0.18%	0.74	**3.07E-02**	17.03%	0.37%	0.74	2.93E-01	17.29%	0.11%	0.74	**1.38E-02**	17.11%	0.29%	0.74	1.03E-01
3	17.01%	0.39%	0.73	3.87E-01	17.04%	0.36%	0.73	2.49E-01	16.99%	0.42%	0.73	6.10E-01	16.98%	0.43%	0.73	8.30E-01
4	17.11%	0.30%	0.74	1.12E-01	17.04%	0.36%	0.74	2.51E-01	17.06%	0.35%	0.74	2.08E-01	16.99%	0.42%	0.73	6.28E-01
5	17.01%	0.39%	0.74	3.96E-01	16.97%	0.43%	0.73	9.23E-01	17.17%	0.24%	0.74	5.55E-02	17.15%	0.26%	0.74	7.10E-02
6	17.16%	0.24%	0.74	6.25E-02	17.81%	0.41%	0.74	**6.16E-05**	16.98%	0.43%	0.73	8.46E-01	17.10%	0.30%	0.73	1.16E-01
7	17.03%	0.37%	0.74	2.81E-01	16.98%	0.43%	0.73	8.57E-01	17.07%	0.34%	0.73	1.79E-01	17.00%	0.40%	0.73	4.49E-01
8	17.00%	0.40%	0.73	4.69E-01	17.01%	0.39%	0.73	3.83E-01	16.97%	0.43%	0.73	9.93E-01	16.99%	0.41%	0.73	5.76E-01
9	17.00%	0.40%	0.73	4.74E-01	17.03%	0.38%	0.74	3.23E-01	16.98%	0.42%	0.73	6.46E-01	16.97%	0.43%	0.73	8.73E-01
10	17.09%	0.32%	0.74	1.46E-01	16.99%	0.42%	0.73	6.32E-01	17.30%	0.11%	0.74	**1.35E-02**	17.29%	0.12%	0.74	**1.47E-02**
11	16.97%	0.43%	0.73	8.84E-01	16.98%	0.43%	0.73	8.10E-01	16.99%	0.41%	0.73	5.38E-01	17.03%	0.37%	0.73	2.94E-01
12	16.99%	0.41%	0.73	5.39E-01	17.01%	0.40%	0.74	4.42E-01	16.99%	0.41%	0.73	5.31E-01	17.07%	0.34%	0.74	1.80E-01
13	16.98%	0.43%	0.73	8.33E-01	16.99%	0.42%	0.73	6.11E-01	17.08%	0.32%	0.74	1.52E-01	17.11%	0.29%	0.74	1.03E-01
14	17.08%	0.33%	0.74	1.64E-01	17.03%	0.38%	0.74	3.06E-01	17.04%	0.36%	0.74	2.56E-01	16.98%	0.43%	0.73	8.01E-01
15	17.01%	0.40%	0.73	4.19E-01	17.05%	0.36%	0.73	2.46E-01	17.00%	0.41%	0.74	5.04E-01	17.17%	0.24%	0.74	5.59E-02
16	16.97%	0.43%	0.73	9.02E-01	17.08%	0.32%	0.74	1.51E-01	17.00%	0.41%	0.74	5.20E-01	17.23%	0.18%	0.74	**2.87E-02**
17	17.04%	0.37%	0.74	2.80E-01	16.99%	0.42%	0.73	6.02E-01	17.06%	0.35%	0.74	2.05E-01	17.00%	0.41%	0.74	4.85E-01
18	17.06%	0.34%	0.74	1.99E-01	17.04%	0.36%	0.74	2.63E-01	17.07%	0.33%	0.74	1.76E-01	16.98%	0.43%	0.73	7.85E-01
19	16.98%	0.42%	0.73	6.74E-01	16.98%	0.43%	0.73	7.95E-01	16.97%	0.43%	0.73	9.93E-01	16.97%	0.43%	0.73	9.82E-01
20	16.98%	0.42%	0.73	7.06E-01	16.98%	0.42%	0.74	6.96E-01	16.97%	0.43%	0.73	9.85E-01	16.99%	0.41%	0.73	5.29E-01
21	16.98%	0.42%	0.73	6.58E-01	17.00%	0.40%	0.73	4.55E-01	17.03%	0.37%	0.74	2.96E-01	17.00%	0.40%	0.74	4.43E-01
22	16.97%	0.43%	0.73	9.86E-01	16.99%	0.42%	0.73	6.25E-01	17.00%	0.40%	0.73	4.70E-01	17.12%	0.29%	0.74	9.97E-02

Supplementary Table S6. Summary of polygenic scores derived from T-lymphocytes subsets and CD4/CD8 ratio by $P_{discovery}$ thresholds (A) and by chromosomes (B) for LS *HLA-DRB1*03* carriers (253 cases and 512 controls) using unpruned sets.

A. By $P_{discovery}$

	CD3 all-SNPs				CD4 all-SNPs				CD8 all-SNPs				CD4/CD8 all-SNPs			
$P_{discovery}$	Nagelkerke R^2 (%)	% explained variance	C-statistic	SCORE P	Nagelkerke R^2 (%)	% explained variance	C-statistic	SCORE P	Nagelkerke R^2 (%)	% explained variance	C-statistic	SCORE P	Nagelkerke R^2 (%)	% explained variance	C-statistic	SCORE P
Baseline	36.47%	0.00%	0.84	**3.45E-50**	36.47%	0.00%	0.84	**3.45E-50**	36.47%	0.00%	0.84	**3.45E-50**	36.47%	0.00%	0.84	**3.45E-50**
5.0E-08	–	–	–	–	36.53%	0.06%	0.84	5.08E-01	37.00%	0.53%	0.85	5.37E-02	36.93%	0.46%	0.85	7.24E-02
5.0E-07	–	–	–	–	36.52%	0.05%	0.84	5.51E-01	36.99%	0.52%	0.85	5.56E-02	–	–	–	–
5.0E-06	–	–	–	–	36.52%	0.05%	0.84	5.58E-01	36.98%	0.51%	0.85	5.84E-02	36.79%	0.31%	0.84	1.36E-01
5.0E-05	36.48%	0.01%	0.84	8.01E-01	36.63%	0.15%	0.84	3.00E-01	36.94%	0.47%	0.85	6.85E-02	36.75%	0.28%	0.84	1.60E-01
5.0E-04	36.81%	0.34%	0.84	1.20E-01	36.53%	0.06%	0.84	5.24E-01	36.94%	0.47%	0.85	6.92E-02	36.74%	0.26%	0.84	1.71E-01
5.0E-03	36.78%	0.31%	0.85	1.37E-01	–	–	–	–	36.83%	0.36%	0.85	1.11E-01	36.75%	0.27%	0.84	1.63E-01
5.0E-02	36.57%	0.10%	0.84	4.05E-01	36.52%	0.05%	0.84	5.56E-01	36.74%	0.27%	0.84	1.67E-01	36.75%	0.28%	0.84	1.57E-01
1.0E-01	36.54%	0.07%	0.84	4.88E-01	36.59%	0.12%	0.84	3.53E-01	36.68%	0.20%	0.84	2.29E-01	36.76%	0.29%	0.84	1.52E-01
2.0E-01	36.52%	0.04%	0.84	5.81E-01	36.62%	0.14%	0.84	3.13E-01	36.66%	0.19%	0.84	2.46E-01	36.81%	0.34%	0.84	1.23E-01
3.0E-01	36.51%	0.04%	0.84	5.97E-01	36.58%	0.11%	0.84	3.76E-01	36.62%	0.15%	0.84	3.09E-01	36.78%	0.30%	0.84	1.42E-01
4.0E-01	36.51%	0.04%	0.84	5.87E-01	36.59%	0.12%	0.84	3.65E-01	36.61%	0.14%	0.84	3.27E-01	36.74%	0.27%	0.84	1.65E-01
5.0E-01	–	–	–	–	36.58%	0.10%	0.84	3.90E-01	36.58%	0.11%	0.84	3.74E-01	36.75%	0.28%	0.84	1.57E-01
6.0E-01	36.51%	0.04%	0.84	6.09E-01	36.54%	0.06%	0.84	5.01E-01	36.58%	0.11%	0.84	3.82E-01	36.75%	0.28%	0.84	1.62E-01
7.0E-01	36.50%	0.03%	0.84	6.33E-01	–	–	–	–	36.58%	0.11%	0.84	3.83E-01	36.74%	0.26%	0.84	1.72E-01
8.0E-01	–	–	–	–	–	–	–	–	–	–	–	–	36.74%	0.27%	0.84	1.67E-01
9.0E-01	–	–	–	–	36.54%	0.07%	0.84	4.87E-01	–	–	–	–	36.75%	0.28%	0.84	1.61E-01
1.0E+00	–	–	–	–	36.54%	0.07%	0.84	4.81E-01	–	–	–	–	36.75%	0.27%	0.84	1.64E-01

B. By chromosome

CHR	CD3 all-SNPS				CD4 all-SNPS				CD8 all-SNPS				CD4/CD8 all-SNPS			
	Nagelkerke R^2 (%)	% explained variance	C-statistic	SCORE P	Nagelkerke R^2 (%)	% explained variance	C-statistic	SCORE P	Nagelkerke R^2 (%)	% explained variance	C-statistic	SCORE P	Nagelkerke R^2 (%)	% explained variance	C-statistic	SCORE P
Baseline	36.47%	0.00%	0.84	**3.45E-50**	36.47%	0.00%	0.84	**3.45E-50**	36.47%	0.00%	0.84	**3.45E-50**	36.47%	0.00%	0.84	**3.45E-50**
1	36.54%	0.07%	0.84	4.89E-01	36.48%	0.00%	0.84	8.64E-01	36.73%	0.25%	0.85	1.80E-01	36.73%	0.26%	0.84	1.77E-01
2	36.48%	0.01%	0.84	8.47E-01	36.48%	0.00%	0.84	8.76E-01	–	–	–	–	36.50%	0.03%	0.84	6.47E-01
3	36.55%	0.08%	0.84	4.46E-01	–	–	–	–	–	–	–	–	36.57%	0.10%	0.84	4.04E-01
4	36.55%	0.08%	0.84	4.50E-01	36.67%	0.20%	0.84	2.38E-01	–	–	–	–	36.48%	0.01%	0.84	7.98E-01
5	36.48%	0.00%	0.84	8.60E-01	–	–	–	–	36.56%	0.09%	0.85	4.34E-01	–	–	–	–
6	36.69%	0.22%	0.84	2.09E-01	36.47%	0.00%	0.84	9.31E-01	36.75%	0.28%	0.84	1.61E-01	36.78%	0.30%	0.84	1.43E-01
7	36.47%	0.00%	0.84	9.58E-01	–	–	–	–	36.68%	0.20%	0.84	2.30E-01	36.74%	0.27%	0.84	1.71E-01
8	36.69%	0.22%	0.84	2.14E-01	36.80%	0.33%	0.85	1.26E-01	–	–	–	–	36.53%	0.06%	0.84	5.29E-01
9	36.77%	0.30%	0.84	1.48E-01	36.74%	0.26%	0.84	1.71E-01	–	–	–	–	36.47%	0.00%	0.84	9.73E-01
10	36.48%	0.01%	0.84	8.01E-01	–	–	–	–	36.50%	0.03%	0.84	6.49E-01	36.49%	0.02%	0.84	6.92E-01
11	36.99%	0.52%	0.85	5.55E-02	37.00%	0.53%	0.85	5.34E-02	–	–	–	–	36.47%	0.00%	0.84	8.95E-01
12	36.49%	0.02%	0.84	7.34E-01	36.48%	0.01%	0.84	8.36E-01	37.05%	0.58%	0.85	**4.30E-02**	36.77%	0.30%	0.84	1.47E-01
13	36.76%	0.29%	0.85	1.56E-01	36.74%	0.27%	0.84	1.72E-01	36.80%	0.33%	0.85	1.27E-01	–	–	–	–
14	36.49%	0.02%	0.84	7.36E-01	–	–	–	–	36.47%	0.00%	0.84	9.17E-01	36.47%	0.00%	0.84	9.72E-01
15	36.60%	0.13%	0.85	3.37E-01	36.58%	0.11%	0.85	3.86E-01	–	–	–	–	36.56%	0.09%	0.84	4.22E-01
16	37.14%	0.67%	0.85	**3.00E-02**	–	–	–	–	–	–	–	–	36.53%	0.06%	0.84	5.18E-01
17	36.52%	0.05%	0.84	5.50E-01	–	–	–	–	36.49%	0.02%	0.84	7.20E-01	36.83%	0.36%	0.85	1.13E-01
18	36.50%	0.02%	0.84	6.84E-01	36.47%	0.00%	0.84	9.86E-01	–	–	–	–	36.49%	0.02%	0.84	6.99E-01
19	36.54%	0.06%	0.84	5.03E-01	–	–	–	–	–	–	–	–	36.62%	0.14%	0.84	3.12E-01
20	36.49%	0.02%	0.84	7.15E-01	–	–	–	–	36.48%	0.00%	0.84	8.75E-01	36.50%	0.03%	0.84	6.52E-01
21	36.47%	0.00%	0.84	8.99E-01	–	–	–	–	–	–	–	–	36.60%	0.13%	0.84	3.43E-01
22	36.51%	0.04%	0.84	6.09E-01	–	–	–	–	–	–	–	–	36.47%	0.00%	0.84	9.17E-01

Supplementary Table S7. Summary of polygenic scores derived from T-lymphocytes subsets and CD4/CD8 ratio by Pdiscovery thresholds (A) and by chromosomes (B) for LS *HLA-DRB1*03* non-carriers (131 cases and 1,574 controls) using unpruned sets.

A. By $P_{discovery}$

	CD3 all-SNPs				CD4 all-SNPs				CD8 all-SNPs				CD4/CD8 all-SNPs			
$P_{discovery}$	Nagelkerke R^2 (%)	% explained variance	C-statistic	SCORE P	Nagelkerke R^2 (%)	% explained variance	C-statistic	SCORE P	Nagelkerke R^2 (%)	% explained variance	C-statistic	SCORE P	Nagelkerke R^2 (%)	% explained variance	C-statistic	SCORE P
Baseline	11.06%	0.00%	0.77	**1.55E-17**	11.06%	0.00%	0.77	**1.55E-17**	11.06%	0.00%	0.77	**1.55E-17**	11.06%	0.00%	0.77	**1.55E-17**
5.0E-08	–	–	–	–	11.52%	0.46%	0.77	7.28E-02	11.73%	0.67%	0.76	**2.70E-02**	12.87%	1.81%	0.77	**3.89E-04**
5.0E-07	–	–	–	–	11.54%	0.48%	0.77	6.68E-02	11.68%	0.62%	0.76	**3.28E-02**	12.77%	1.71%	0.77	**5.63E-04**
5.0E-06	–	–	–	–	11.75%	0.69%	0.77	**2.70E-02**	–	–	–	–	12.58%	1.52%	0.77	**1.14E-03**
5.0E-05	–	–	–	–	11.68%	0.62%	0.77	**3.65E-02**	–	–	–	–	12.49%	1.43%	0.77	**1.62E-03**
5.0E-04	11.10%	0.04%	0.77	6.04E-01	11.62%	0.56%	0.77	5.10E-02	12.06%	1.00%	0.77	**7.16E-03**	12.49%	1.43%	0.77	**1.64E-03**
5.0E-03	11.13%	0.07%	0.77	4.89E-01	11.78%	0.71%	0.77	**2.76E-02**	11.96%	0.90%	0.76	**1.09E-02**	12.61%	1.55%	0.77	**1.07E-03**
5.0E-02	11.07%	0.01%	0.77	8.06E-01	12.06%	1.00%	0.77	**9.16E-03**	12.36%	1.30%	0.76	**2.36E-03**	12.52%	1.46%	0.77	**1.50E-03**
1.0E-01	11.14%	0.08%	0.77	4.57E-01	11.92%	0.86%	0.77	**1.59E-02**	12.23%	1.17%	0.76	**4.00E-03**	12.55%	1.49%	0.77	**1.32E-03**
2.0E-01	11.13%	0.07%	0.77	4.80E-01	–	–	–	–	12.15%	1.09%	0.76	**5.49E-03**	12.47%	1.41%	0.77	**1.80E-03**
3.0E-01	11.14%	0.08%	0.77	4.51E-01	11.53%	0.47%	0.77	7.60E-02	12.07%	1.01%	0.76	**7.61E-03**	12.45%	1.39%	0.77	**1.97E-03**
4.0E-01	11.18%	0.12%	0.77	3.73E-01	11.52%	0.46%	0.77	7.82E-02	12.03%	0.97%	0.76	**8.92E-03**	12.49%	1.43%	0.77	**1.70E-03**
5.0E-01	11.16%	0.10%	0.77	4.19E-01	11.53%	0.47%	0.77	7.63E-02	12.05%	0.99%	0.76	**8.25E-03**	12.51%	1.45%	0.77	**1.57E-03**
6.0E-01	11.19%	0.13%	0.77	3.44E-01	11.50%	0.44%	0.77	8.40E-02	12.11%	1.05%	0.76	**6.47E-03**	12.50%	1.44%	0.77	**1.65E-03**
7.0E-01	11.21%	0.15%	0.77	3.07E-01	11.51%	0.45%	0.77	8.09E-02	12.10%	1.04%	0.76	**6.81E-03**	12.48%	1.42%	0.77	**1.75E-03**
8.0E-01	11.22%	0.16%	0.77	3.00E-01	–	–	–	–	12.11%	1.05%	0.76	**6.49E-03**	12.48%	1.42%	0.77	**1.79E-03**
9.0E-01	11.23%	0.17%	0.77	2.76E-01	–	–	–	–	12.11%	1.05%	0.76	**6.46E-03**	12.48%	1.42%	0.77	**1.77E-03**
1.0E+00	11.23%	0.17%	0.77	2.78E-01	–	–	–	–	12.12%	1.06%	0.76	**6.32E-03**	12.49%	1.43%	0.77	**1.73E-03**

B. By chromosome

CHR	CD3 all-SNPs				CD4 all-SNPs				CD8 all-SNPs				CD4/CD8 all-SNPs			
	Nagelkerke R^2 (%)	% explained variance	C-statistic	SCORE P	Nagelkerke R^2 (%)	% explained variance	C-statistic	SCORE P	Nagelkerke R^2 (%)	% explained variance	C-statistic	SCORE P	Nagelkerke R^2 (%)	% explained variance	C-statistic	SCORE P
Baseline	11.06%	0.00%	0.77	**1.55E-17**	11.06%	0.00%	0.77	**1.55E-17**	11.06%	0.00%	0.77	**1.55E-17**	11.06%	0.00%	0.77	**1.55E-17**
1	–	–	–	–	11.14%	0.08%	0.77	4.70E-01	–	–	–	–	–	–	–	–
2	11.24%	0.18%	0.77	2.71E-01	11.32%	0.26%	0.77	1.86E-01	–	–	–	–	11.10%	0.04%	0.77	6.25E-01
3	–	–	–	–	–	–	–	–	–	–	–	–	11.07%	0.01%	0.77	8.39E-01
4	–	–	–	–	11.07%	0.01%	0.77	7.96E-01	–	–	–	–	11.23%	0.17%	0.77	2.80E-01
5	11.89%	0.83%	0.77	**1.89E-02**	–	–	–	–	–	–	–	–	–	–	–	–
6	11.29%	0.23%	0.77	1.99E-01	11.94%	0.87%	0.77	**1.41E-02**	12.29%	1.23%	0.76	**2.97E-03**	12.51%	1.45%	0.77	**1.50E-03**
7	–	–	–	–	–	–	–	–	11.07%	0.01%	0.77	8.05E-01	11.06%	0.00%	0.77	9.95E-01
8	–	–	–	–	–	–	–	–	–	–	–	–	11.07%	0.01%	0.77	8.04E-01
9	–	–	–	–	11.06%	0.00%	0.77	9.54E-01	–	–	–	–	–	–	–	–
10	–	–	–	–	–	–	–	–	11.06%	0.00%	0.77	9.50E-01	11.14%	0.08%	0.77	4.52E-01
11	–	–	–	–	–	–	–	–	11.22%	0.16%	0.77	3.05E-01	11.15%	0.09%	0.77	4.29E-01
12	11.07%	0.01%	0.77	7.89E-01	11.07%	0.01%	0.77	7.83E-01	11.15%	0.09%	0.77	4.41E-01	11.11%	0.05%	0.77	5.60E-01
13	–	–	–	–	11.09%	0.03%	0.77	6.58E-01	–	–	–	–	–	–	–	–
14	–	–	–	–	–	–	–	–	11.28%	0.22%	0.77	2.27E-01	11.17%	0.11%	0.77	3.88E-01
15	11.19%	0.13%	0.77	3.58E-01	11.12%	0.05%	0.77	5.45E-01	11.12%	0.06%	0.77	5.11E-01	11.08%	0.02%	0.77	7.39E-01
16	11.07%	0.00%	0.77	8.62E-01	11.09%	0.03%	0.77	6.39E-01	11.10%	0.04%	0.77	5.85E-01	–	–	–	–
17	–	–	–	–	–	–	–	–	11.24%	0.18%	0.77	2.81E-01	11.76%	0.70%	0.77	**2.93E-02**
18	–	–	–	–	–	–	–	–	–	–	–	–	11.10%	0.04%	0.77	6.19E-01
19	–	–	–	–	–	–	–	–	–	–	–	–	11.35%	0.29%	0.77	1.64E-01
20	11.07%	0.01%	0.77	8.21E-01	–	–	–	–	11.07%	0.01%	0.77	7.61E-01	11.06%	0.00%	0.77	9.29E-01
21	–	–	–	–	–	–	–	–	–	–	–	–	11.64%	0.58%	0.77	**4.87E-02**
22	11.25%	0.19%	0.77	2.65E-01	–	–	–	–	–	–	–	–	11.28%	0.22%	0.77	2.27E-01

Supplementary Table S8. Summary of polygenic scores derived from T-lymphocytes subsets (A-C) and CD4/CD8 ratio (D) by $P_{discovery}$ thresholds for LS (384 cases and 2,086 controls) using pruned sets. Note that the pairwise LD between pairs of genic- and intergenic-SNPs is r^2 < 0.25.

A. CD3+

	CD3 pruned-SNPs				CD3 genic-SNPs				CD3 intergenic-SNPs			
$P_{discovery}$	Nagelkerke R^2 (%)	% explained variance	C-statistic	SCORE P	Nagelkerke R^2 (%)	% explained variance	C-statistic	SCORE P	Nagelkerke R^2 (%)	% explained variance	C-statistic	SCORE P
Baseline	21.81%	0.00%	0.81	**5.48E-73**	21.81%	0.00%	0.81	**5.48E-73**	21.81%	0.00%	0.81	**5.48E-73**
5.0E-08	–	–	–	–	–	–	–	–	–	–	–	–
5.0E-07	–	–	–	–	–	–	–	–	–	–	–	–
5.0E-06	21.83%	0.03%	0.81	7.29E-01	21.83%	0.03%	0.81	7.29E-01	–	–	–	–
5.0E-05	22.64%	0.83%	0.81	**4.82E-04**	21.83%	0.03%	0.81	7.29E-01	23.71%	1.90%	0.81	**1.40E-07**
5.0E-04	21.90%	0.10%	0.81	2.78E-01	21.83%	0.02%	0.81	9.95E-01	21.95%	0.14%	0.81	1.67E-01
5.0E-03	21.83%	0.02%	0.81	9.16E-01	21.83%	0.02%	0.81	7.58E-01	21.83%	0.02%	0.81	8.89E-01
5.0E-02	21.97%	0.16%	0.81	1.38E-01	21.88%	0.07%	0.81	3.55E-01	21.92%	0.11%	0.81	2.44E-01
1.0E-01	22.03%	0.22%	0.81	8.22E-02	22.09%	0.28%	0.81	**4.49E-02**	21.85%	0.04%	0.81	5.34E-01
2.0E-01	21.92%	0.11%	0.81	2.28E-01	21.96%	0.15%	0.81	1.59E-01	21.84%	0.03%	0.81	6.72E-01
3.0E-01	21.85%	0.04%	0.81	5.33E-01	21.89%	0.08%	0.81	3.40E-01	21.83%	0.02%	0.81	9.94E-01
4.0E-01	21.84%	0.04%	0.81	6.05E-01	21.85%	0.05%	0.81	5.12E-01	21.83%	0.02%	0.81	9.11E-01
5.0E-01	21.85%	0.04%	0.81	5.84E-01	21.84%	0.03%	0.81	6.59E-01	21.83%	0.02%	0.81	7.67E-01
6.0E-01	21.85%	0.04%	0.81	5.39E-01	21.84%	0.04%	0.81	6.16E-01	21.83%	0.02%	0.81	7.60E-01
7.0E-01	21.83%	0.02%	0.81	8.35E-01	21.83%	0.02%	0.81	8.22E-01	21.83%	0.02%	0.81	9.96E-01
8.0E-01	21.83%	0.02%	0.81	8.91E-01	21.83%	0.02%	0.81	8.61E-01	21.83%	0.02%	0.81	9.59E-01
9.0E-01	21.83%	0.02%	0.81	8.49E-01	21.83%	0.02%	0.81	8.56E-01	21.83%	0.02%	0.81	9.92E-01
1.0E+00	21.83%	0.02%	0.81	8.68E-01	21.83%	0.02%	0.81	8.53E-01	21.83%	0.02%	0.81	9.80E-01

B. CD4+

$P_{discovery}$	CD4 pruned-SNPs				CD4 genic-SNPS				CD4 intergenic-SNPS			
	Nagelkerke R^2 (%)	% explained variance	C-statistic	SCORE P	Nagelkerke R^2 (%)	% explained variance	C-statistic	SCORE P	Nagelkerke R^2 (%)	% explained variance	C-statistic	SCORE P
Baseline	21.81%	0.00%	0.81	**5.48E-73**	21.81%	0.00%	0.81	**5.48E-73**	21.81%	0.00%	0.81	**5.48E-73**
5.0E-08	–	–	–	–	–	–	–	–	–	–	–	–
5.0E-07	–	–	–	–	–	–	–	–	–	–	–	–
5.0E-06	21.83%	0.03%	0.81	7.29E-01	21.83%	0.03%	0.81	7.29E-01	–	–	–	–
5.0E-05	22.22%	0.41%	0.81	**1.44E-02**	22.22%	0.41%	0.81	**1.44E-02**	–	–	–	–
5.0E-04	22.29%	0.48%	0.81	**8.36E-03**	22.16%	0.35%	0.81	**2.41E-02**	21.94%	0.13%	0.81	1.88E-01
5.0E-03	21.83%	0.02%	0.81	9.46E-01	21.84%	0.03%	0.81	6.36E-01	21.84%	0.04%	0.81	6.00E-01
5.0E-02	22.05%	0.24%	0.81	6.73E-02	22.53%	0.72%	0.81	**1.10E-03**	21.83%	0.03%	0.81	7.27E-01
1.0E-01	21.96%	0.15%	0.81	1.61E-01	22.10%	0.29%	0.81	**4.15E-02**	21.83%	0.02%	0.81	8.88E-01
2.0E-01	21.83%	0.02%	0.81	9.95E-01	21.93%	0.12%	0.81	2.17E-01	21.90%	0.10%	0.81	2.76E-01
3.0E-01	21.83%	0.02%	0.81	8.43E-01	21.92%	0.11%	0.81	2.28E-01	21.87%	0.06%	0.81	4.15E-01
4.0E-01	21.83%	0.02%	0.81	9.05E-01	21.83%	0.02%	0.81	7.93E-01	21.83%	0.02%	0.81	9.18E-01
5.0E-01	21.83%	0.02%	0.81	7.73E-01	21.83%	0.02%	0.81	9.99E-01	21.84%	0.03%	0.81	6.54E-01
6.0E-01	21.84%	0.03%	0.81	6.75E-01	21.83%	0.02%	0.81	9.27E-01	21.86%	0.05%	0.81	4.62E-01
7.0E-01	21.84%	0.04%	0.81	6.12E-01	21.83%	0.02%	0.81	9.59E-01	21.86%	0.05%	0.81	4.66E-01
8.0E-01	21.85%	0.04%	0.81	5.79E-01	21.83%	0.02%	0.81	8.73E-01	21.86%	0.05%	0.81	4.83E-01
9.0E-01	21.86%	0.05%	0.81	5.02E-01	21.83%	0.02%	0.81	7.86E-01	21.86%	0.06%	0.81	4.49E-01
1.0E+00	21.86%	0.05%	0.81	4.84E-01	21.83%	0.02%	0.81	7.88E-01	21.87%	0.06%	0.81	4.27E-01

C. CD8+

$P_{discovery}$	CD8 pruned-SNPs Nagelkerke R^2 (%)	% explained variance	C-statistic	SCORE P	CD8 genic-SNPs Nagelkerke R^2 (%)	% explained variance	C-statistic	SCORE P	CD8 intergenic-SNPs Nagelkerke R^2 (%)	% explained variance	C-statistic	SCORE P
Baseline	21.81%	0.00%	0.81	**5.48E-73**	21.81%	0.00%	0.81	**5.48E-73**	21.81%	0.00%	0.81	**5.48E-73**
5.0E-08	23.11%	1.31%	0.82	**1.22E-05**	22.19%	0.39%	0.81	**1.82E-02**	23.05%	1.25%	0.81	**1.89E-05**
5.0E-07	24.41%	2.61%	0.82	**7.23E-10**	23.62%	1.81%	0.81	**2.43E-07**	23.05%	1.25%	0.81	**1.89E-05**
5.0E-06	25.01%	3.20%	0.82	**9.25E-12**	23.64%	1.83%	0.81	**2.12E-07**	24.00%	2.20%	0.82	**1.91E-08**
5.0E-05	24.96%	3.15%	0.82	**1.26E-11**	24.78%	2.98%	0.82	**4.08E-11**	23.03%	1.22%	0.81	**2.65E-05**
5.0E-04	25.70%	3.89%	0.82	**6.94E-14**	25.70%	3.89%	0.82	**5.55E-14**	23.27%	1.46%	0.81	**4.25E-06**
5.0E-03	24.89%	3.08%	0.82	**1.95E-11**	24.32%	2.52%	0.82	**1.06E-09**	23.41%	1.60%	0.81	**1.30E-06**
5.0E-02	22.97%	1.17%	0.81	**3.16E-05**	22.48%	0.68%	0.81	**1.55E-03**	22.50%	0.69%	0.81	**1.47E-03**
1.0E-01	22.55%	0.75%	0.81	**8.94E-04**	22.10%	0.29%	0.81	**4.02E-02**	22.35%	0.55%	0.81	**4.68E-03**
2.0E-01	22.20%	0.39%	0.81	**1.69E-02**	22.03%	0.22%	0.81	7.77E-02	22.03%	0.22%	0.81	7.97E-02
3.0E-01	22.09%	0.28%	0.81	**4.57E-02**	22.07%	0.26%	0.81	5.64E-02	21.90%	0.09%	0.81	2.96E-01
4.0E-01	22.05%	0.24%	0.81	6.56E-02	22.06%	0.25%	0.81	5.99E-02	21.87%	0.06%	0.81	4.21E-01
5.0E-01	21.94%	0.13%	0.81	1.85E-01	21.99%	0.18%	0.81	1.15E-01	21.84%	0.03%	0.81	7.15E-01
6.0E-01	21.97%	0.16%	0.81	1.40E-01	21.98%	0.18%	0.81	1.22E-01	21.85%	0.04%	0.81	5.55E-01
7.0E-01	21.98%	0.17%	0.81	1.27E-01	21.95%	0.14%	0.81	1.79E-01	21.87%	0.07%	0.81	3.99E-01
8.0E-01	21.99%	0.18%	0.81	1.20E-01	21.96%	0.15%	0.81	1.61E-01	21.87%	0.06%	0.81	4.10E-01
9.0E-01	22.00%	0.20%	0.81	1.01E-01	21.99%	0.18%	0.81	1.20E-01	21.87%	0.06%	0.81	4.20E-01
1.0E+00	22.01%	0.20%	0.81	9.57E-02	21.99%	0.18%	0.81	1.14E-01	21.87%	0.06%	0.81	4.11E-01

D. CD4/CD8

	CD4/CD8 pruned-SNPs				CD4/CD8 genic-SNPs				CD4/CD8 intergenic-SNPs			
$P_{discovery}$	Nagelkerke R^2 (%)	% explained variance	C-statistic	SCORE P	Nagelkerke R^2 (%)	% explained variance	C-statistic	SCORE P	Nagelkerke R^2 (%)	% explained variance	C-statistic	SCORE P
Baseline	21.81%	0.00%	0.81	**5.48E-73**	21.81%	0.00%	0.81	**5.48E-73**	21.81%	0.00%	0.81	**5.48E-73**
5.0E-08	26.20%	4.39%	0.82	**1.32E-15**	22.19%	0.39%	0.81	**1.82E-02**	23.05%	1.25%	0.81	**1.89E-05**
5.0E-07	26.94%	5.13%	0.83	**6.91E-18**	23.62%	1.81%	0.81	**2.43E-07**	23.05%	1.25%	0.81	**1.89E-05**
5.0E-06	25.58%	3.77%	0.82	**1.11E-13**	23.64%	1.83%	0.81	**2.12E-07**	24.00%	2.20%	0.82	**1.91E-08**
5.0E-05	25.93%	4.12%	0.82	**8.70E-15**	24.78%	2.98%	0.82	**4.08E-11**	23.03%	1.22%	0.81	**2.65E-05**
5.0E-04	25.14%	3.33%	0.82	**3.07E-12**	25.70%	3.89%	0.82	**5.55E-14**	23.27%	1.46%	0.81	**4.25E-06**
5.0E-03	25.43%	3.62%	0.82	**3.60E-13**	24.32%	2.52%	0.82	**1.06E-09**	23.41%	1.60%	0.81	**1.30E-06**
5.0E-02	24.62%	2.81%	0.82	**1.28E-10**	22.48%	0.68%	0.81	**1.55E-03**	22.50%	0.69%	0.81	**1.47E-03**
1.0E-01	23.66%	1.86%	0.81	**1.58E-07**	22.10%	0.29%	0.81	**4.02E-02**	22.35%	0.55%	0.81	**4.68E-03**
2.0E-01	23.42%	1.61%	0.81	**9.73E-07**	22.03%	0.22%	0.81	7.77E-02	22.03%	0.22%	0.81	7.97E-02
3.0E-01	23.03%	1.22%	0.81	**2.06E-05**	22.07%	0.26%	0.81	5.64E-02	21.90%	0.09%	0.81	2.96E-01
4.0E-01	22.68%	0.87%	0.81	**3.19E-04**	22.06%	0.25%	0.81	5.99E-02	21.87%	0.06%	0.81	4.21E-01
5.0E-01	22.57%	0.76%	0.81	**8.19E-04**	21.99%	0.18%	0.81	1.15E-01	21.84%	0.03%	0.81	7.15E-01
6.0E-01	22.44%	0.64%	0.81	**2.22E-03**	21.98%	0.18%	0.81	1.22E-01	21.85%	0.04%	0.81	5.55E-01
7.0E-01	22.41%	0.60%	0.81	**2.93E-03**	21.95%	0.14%	0.81	1.79E-01	21.87%	0.07%	0.81	3.99E-01
8.0E-01	22.38%	0.57%	0.81	**3.76E-03**	21.96%	0.15%	0.81	1.61E-01	21.87%	0.06%	0.81	4.10E-01
9.0E-01	22.40%	0.59%	0.81	**3.26E-03**	21.99%	0.18%	0.81	1.20E-01	21.87%	0.06%	0.81	4.20E-01
1.0E+00	22.39%	0.58%	0.81	**3.48E-03**	21.99%	0.18%	0.81	1.14E-01	21.87%	0.06%	0.81	4.11E-01

Supplementary Table S9. Summary of polygenic scores derived from T-lymphocytes subsets (A-C) and CD4/CD8 ratio (D) by chromsomes for LS (384 cases and 2,086 controls) using pruned sets. Note that the pairwise LD between pairs of genic- and intergenic-SNPs is $r^2 < 0.25$.

A. CD3+

	CD3 pruned-SNPs				CD3 genic-SNPs				CD3 intergenic-SNPs			
CHR	Nagelkerke R^2 (%)	% explained variance	C-statistic	SCORE P	Nagelkerke R2	% explained variance	C-statistic	SCORE P	Nagelkerke R2	% explained variance	C-statistic	SCORE P
Baseline	21.81%	0.00%	0.81	**5.48E-73**	21.81%	0.00%	0.81	**5.48E-73**	21.81%	0.00%	0.81	**5.48E-73**
1	21.96%	0.15%	0.81	1.56E-01	21.83%	0.02%	0.81	7.89E-01	22.23%	0.42%	0.81	**1.31E-02**
2	21.86%	0.06%	0.81	4.56E-01	21.83%	0.02%	0.81	7.97E-01	22.02%	0.21%	0.81	8.77E-02
3	21.83%	0.02%	0.81	9.11E-01	21.83%	0.02%	0.81	8.55E-01	21.83%	0.02%	0.81	9.46E-01
4	21.93%	0.12%	0.81	2.04E-01	21.84%	0.03%	0.81	6.21E-01	21.97%	0.16%	0.81	1.40E-01
5	21.84%	0.03%	0.81	6.51E-01	21.96%	0.15%	0.81	1.59E-01	21.89%	0.08%	0.81	3.44E-01
6	24.10%	2.29%	0.81	**5.32E-09**	24.07%	2.26%	0.81	**6.23E-09**	23.14%	1.34%	0.81	**8.57E-06**
7	21.91%	0.10%	0.81	2.68E-01	22.01%	0.21%	0.81	9.09E-02	21.83%	0.02%	0.81	8.79E-01
8	21.85%	0.04%	0.81	5.93E-01	21.83%	0.02%	0.81	8.53E-01	21.90%	0.09%	0.81	2.95E-01
9	21.83%	0.02%	0.81	8.94E-01	21.83%	0.02%	0.81	9.78E-01	21.83%	0.03%	0.81	7.26E-01
10	21.88%	0.07%	0.81	3.71E-01	21.83%	0.02%	0.81	8.14E-01	21.87%	0.06%	0.81	4.03E-01
11	21.95%	0.14%	0.81	1.69E-01	22.02%	0.21%	0.81	8.52E-02	21.86%	0.06%	0.81	4.47E-01
12	21.83%	0.02%	0.81	9.29E-01	21.83%	0.02%	0.81	9.32E-01	21.83%	0.02%	0.81	9.89E-01
13	21.92%	0.11%	0.81	2.45E-01	21.87%	0.06%	0.81	4.17E-01	22.06%	0.25%	0.81	6.10E-02
14	21.85%	0.05%	0.81	5.25E-01	21.83%	0.02%	0.81	8.15E-01	21.83%	0.02%	0.81	9.32E-01
15	21.83%	0.02%	0.81	9.15E-01	22.04%	0.24%	0.81	6.88E-02	21.92%	0.11%	0.81	2.37E-01
16	21.96%	0.15%	0.81	1.55E-01	21.88%	0.07%	0.81	3.80E-01	21.87%	0.06%	0.81	4.05E-01
17	21.99%	0.19%	0.81	1.11E-01	21.85%	0.05%	0.81	5.22E-01	21.92%	0.11%	0.81	2.32E-01
18	21.83%	0.02%	0.81	8.07E-01	21.90%	0.09%	0.81	2.94E-01	21.87%	0.07%	0.81	3.97E-01
19	21.89%	0.08%	0.81	3.36E-01	21.83%	0.03%	0.81	7.27E-01	22.21%	0.40%	0.81	**1.58E-02**
20	21.84%	0.03%	0.81	6.22E-01	21.83%	0.03%	0.81	7.52E-01	21.84%	0.03%	0.81	6.50E-01
21	21.83%	0.02%	0.81	8.08E-01	21.93%	0.12%	0.81	2.14E-01	21.94%	0.13%	0.81	1.98E-01
22	21.86%	0.05%	0.81	4.80E-01	21.89%	0.08%	0.81	3.32E-01	21.83%	0.02%	0.81	8.71E-01

B. CD4+

CHR	CD4 pruned-SNPs				CD4 genic-SNPS				CD4 intergenic-SNPS			
	Nagelkerke R^2 (%)	% explained variance	C-statistic	SCORE P	Nagelkerke R2	% explained variance	C-statistic	SCORE P	Nagelkerke R2	% explained variance	C-statistic	SCORE P
Baseline	21.81%	0.00%	0.81	**5.48E-73**	21.81%	0.00%	0.81	**5.48E-73**	21.81%	0.00%	0.81	**5.48E-73**
1	21.89%	0.09%	0.81	3.14E-01	21.85%	0.04%	0.81	5.31E-01	22.11%	0.30%	0.81	**3.96E-02**
2	21.95%	0.14%	0.81	1.74E-01	21.83%	0.02%	0.81	8.31E-01	22.08%	0.27%	0.81	**4.90E-02**
3	21.86%	0.05%	0.81	4.95E-01	21.90%	0.09%	0.81	3.06E-01	21.83%	0.02%	0.81	9.58E-01
4	21.91%	0.10%	0.81	2.66E-01	21.86%	0.05%	0.81	5.07E-01	21.88%	0.08%	0.81	3.54E-01
5	21.92%	0.11%	0.81	2.31E-01	22.26%	0.46%	0.81	**1.00E-02**	21.84%	0.04%	0.81	6.15E-01
6	21.83%	0.02%	0.81	9.45E-01	21.84%	0.04%	0.81	6.18E-01	21.84%	0.04%	0.81	6.08E-01
7	21.83%	0.02%	0.81	8.56E-01	21.87%	0.06%	0.81	4.42E-01	21.89%	0.08%	0.81	3.36E-01
8	21.83%	0.02%	0.81	7.92E-01	21.85%	0.05%	0.81	5.23E-01	21.87%	0.06%	0.81	4.28E-01
9	21.84%	0.03%	0.81	6.32E-01	21.84%	0.04%	0.81	6.01E-01	21.83%	0.02%	0.81	8.59E-01
10	21.84%	0.04%	0.81	6.07E-01	21.83%	0.02%	0.81	9.93E-01	21.86%	0.05%	0.81	4.92E-01
11	21.87%	0.06%	0.81	4.35E-01	21.83%	0.02%	0.81	9.43E-01	21.89%	0.08%	0.81	3.36E-01
12	21.84%	0.03%	0.81	6.20E-01	21.83%	0.02%	0.81	9.21E-01	21.85%	0.04%	0.81	5.47E-01
13	21.86%	0.05%	0.81	4.73E-01	21.84%	0.03%	0.81	7.02E-01	21.92%	0.11%	0.81	2.40E-01
14	21.87%	0.06%	0.81	4.30E-01	21.83%	0.03%	0.81	7.30E-01	21.86%	0.05%	0.81	4.67E-01
15	21.84%	0.03%	0.81	6.72E-01	22.00%	0.19%	0.81	1.08E-01	21.89%	0.08%	0.81	3.39E-01
16	21.86%	0.05%	0.81	4.91E-01	21.83%	0.02%	0.81	8.39E-01	21.87%	0.06%	0.81	4.37E-01
17	21.99%	0.18%	0.81	1.18E-01	21.87%	0.06%	0.81	4.05E-01	21.96%	0.15%	0.81	1.52E-01
18	21.83%	0.02%	0.81	9.10E-01	21.85%	0.04%	0.81	5.56E-01	21.83%	0.02%	0.81	8.10E-01
19	22.08%	0.27%	0.81	**4.91E-02**	21.85%	0.04%	0.81	5.62E-01	22.58%	0.77%	0.81	**7.57E-04**
20	21.85%	0.05%	0.81	5.21E-01	21.88%	0.07%	0.81	3.76E-01	21.83%	0.02%	0.81	9.92E-01
21	21.93%	0.12%	0.81	2.05E-01	21.97%	0.16%	0.81	1.39E-01	21.84%	0.03%	0.81	7.02E-01
22	22.04%	0.23%	0.81	6.99E-02	22.16%	0.35%	0.81	**2.48E-02**	21.83%	0.02%	0.81	9.50E-01

C. CD8+

CHR	CD8 pruned-SNPs				CD8 genic-SNPs				CD8 intergenic-SNPs			
	Nagelkerke R^2 (%)	% explained variance	C-statistic	SCORE P	Nagelkerke R2	% explained variance	C-statistic	SCORE P	Nagelkerke R2	% explained variance	C-statistic	SCORE P
Baseline	21.81%	0.00%	0.81	**5.48E-73**	21.81%	0.00%	0.81	**5.48E-73**	21.81%	0.00%	0.81	**5.48E-73**
1	21.95%	0.14%	0.81	1.71E-01	21.83%	0.02%	0.81	7.64E-01	22.14%	0.33%	0.81	**2.88E-02**
2	21.83%	0.02%	0.81	8.20E-01	21.93%	0.12%	0.81	2.12E-01	21.86%	0.05%	0.81	4.99E-01
3	21.83%	0.02%	0.81	8.22E-01	21.84%	0.03%	0.81	7.02E-01	21.83%	0.02%	0.81	9.61E-01
4	21.93%	0.12%	0.81	2.20E-01	21.84%	0.04%	0.81	6.05E-01	22.08%	0.27%	0.81	5.10E-02
5	21.85%	0.05%	0.81	5.19E-01	21.85%	0.04%	0.81	5.62E-01	21.95%	0.14%	0.81	1.70E-01
6	25.61%	3.81%	0.82	**7.03E-14**	24.29%	2.49%	0.82	**1.32E-09**	24.61%	2.80%	0.81	**1.52E-10**
7	22.16%	0.35%	0.81	**2.56E-02**	22.12%	0.31%	0.81	**3.51E-02**	21.89%	0.08%	0.81	3.25E-01
8	21.83%	0.02%	0.81	8.87E-01	21.88%	0.08%	0.81	3.52E-01	21.88%	0.07%	0.81	3.65E-01
9	21.86%	0.05%	0.81	4.84E-01	22.00%	0.19%	0.81	1.03E-01	21.84%	0.03%	0.81	7.01E-01
10	21.86%	0.05%	0.81	4.62E-01	21.91%	0.11%	0.81	2.51E-01	21.83%	0.02%	0.81	9.25E-01
11	21.91%	0.10%	0.81	2.75E-01	21.96%	0.15%	0.81	1.61E-01	21.83%	0.02%	0.81	8.86E-01
12	21.83%	0.02%	0.81	8.74E-01	21.84%	0.04%	0.81	6.00E-01	21.86%	0.05%	0.81	5.09E-01
13	21.86%	0.05%	0.81	5.02E-01	22.00%	0.19%	0.81	1.09E-01	22.06%	0.25%	0.81	6.22E-02
14	21.84%	0.03%	0.81	6.68E-01	21.84%	0.03%	0.81	6.19E-01	21.88%	0.08%	0.81	3.52E-01
15	21.87%	0.07%	0.81	3.98E-01	21.84%	0.04%	0.81	6.00E-01	22.00%	0.19%	0.81	1.07E-01
16	21.94%	0.13%	0.81	1.86E-01	21.97%	0.16%	0.81	1.36E-01	21.84%	0.03%	0.81	6.77E-01
17	21.91%	0.11%	0.81	2.49E-01	21.93%	0.12%	0.81	2.14E-01	21.85%	0.04%	0.81	5.81E-01
18	21.83%	0.02%	0.81	7.66E-01	22.00%	0.19%	0.81	1.08E-01	21.94%	0.13%	0.81	1.91E-01
19	21.83%	0.02%	0.81	8.01E-01	21.92%	0.11%	0.81	2.40E-01	21.86%	0.05%	0.81	4.68E-01
20	21.83%	0.02%	0.81	9.82E-01	21.87%	0.06%	0.81	4.45E-01	21.87%	0.06%	0.81	4.17E-01
21	22.07%	0.26%	0.81	5.39E-02	21.83%	0.02%	0.81	8.87E-01	22.19%	0.38%	0.81	**1.95E-02**
22	21.83%	0.02%	0.81	9.20E-01	21.85%	0.04%	0.81	5.82E-01	21.85%	0.04%	0.81	5.69E-01

D. CD4/CD8

CHR	CD4/CD8 pruned-SNPs				CD4/CD8 genic-SNPs				CD4/CD8 intergenic-SNPs			
	Nagelkerke R^2 (%)	% explained variance	C-statistic	SCORE P	Nagelkerke R2	% explained variance	C-statistic	SCORE P	Nagelkerke R2	% explained variance	C-statistic	SCORE P
Baseline	21.81%	0.00%	0.81	**5.48E-73**	21.81%	0.00%	0.81	**5.48E-73**	21.81%	0.00%	0.81	**5.48E-73**
1	21.83%	0.02%	0.81	9.66E-01	21.83%	0.02%	0.81	7.64E-01	22.14%	0.33%	0.81	**2.88E-02**
2	21.88%	0.07%	0.81	3.70E-01	21.93%	0.12%	0.81	2.12E-01	21.86%	0.05%	0.81	4.99E-01
3	21.87%	0.06%	0.81	4.14E-01	21.84%	0.03%	0.81	7.02E-01	21.83%	0.02%	0.81	9.61E-01
4	21.86%	0.06%	0.81	4.56E-01	21.84%	0.04%	0.81	6.05E-01	22.08%	0.27%	0.81	5.10E-02
5	21.99%	0.18%	0.81	1.14E-01	21.85%	0.04%	0.81	5.62E-01	21.95%	0.14%	0.81	1.70E-01
6	25.01%	3.20%	0.82	**6.63E-12**	24.29%	2.49%	0.82	**1.32E-09**	24.61%	2.80%	0.81	**1.52E-10**
7	21.97%	0.16%	0.81	1.36E-01	22.12%	0.31%	0.81	**3.51E-02**	21.89%	0.08%	0.81	3.25E-01
8	21.84%	0.03%	0.81	6.27E-01	21.88%	0.08%	0.81	3.52E-01	21.88%	0.07%	0.81	3.65E-01
9	21.85%	0.04%	0.81	5.69E-01	22.00%	0.19%	0.81	1.03E-01	21.84%	0.03%	0.81	7.01E-01
10	21.88%	0.07%	0.81	3.80E-01	21.91%	0.11%	0.81	2.51E-01	21.83%	0.02%	0.81	9.25E-01
11	21.83%	0.02%	0.81	8.56E-01	21.96%	0.15%	0.81	1.61E-01	21.83%	0.02%	0.81	8.86E-01
12	21.87%	0.06%	0.81	4.36E-01	21.84%	0.04%	0.81	6.00E-01	21.86%	0.05%	0.81	5.09E-01
13	21.83%	0.03%	0.81	7.37E-01	22.00%	0.19%	0.81	1.09E-01	22.06%	0.25%	0.81	6.22E-02
14	21.83%	0.02%	0.81	8.51E-01	21.84%	0.03%	0.81	6.19E-01	21.88%	0.08%	0.81	3.52E-01
15	21.99%	0.18%	0.81	1.12E-01	21.84%	0.04%	0.81	6.00E-01	22.00%	0.19%	0.81	1.07E-01
16	21.86%	0.05%	0.81	5.09E-01	21.97%	0.16%	0.81	1.36E-01	21.84%	0.03%	0.81	6.77E-01
17	21.84%	0.04%	0.81	6.00E-01	21.93%	0.12%	0.81	2.14E-01	21.85%	0.04%	0.81	5.81E-01
18	21.86%	0.05%	0.81	4.71E-01	22.00%	0.19%	0.81	1.08E-01	21.94%	0.13%	0.81	1.91E-01
19	22.09%	0.28%	0.81	**4.49E-02**	21.92%	0.11%	0.81	2.40E-01	21.86%	0.05%	0.81	4.68E-01
20	21.87%	0.06%	0.81	4.05E-01	21.87%	0.06%	0.81	4.45E-01	21.87%	0.06%	0.81	4.17E-01
21	22.73%	0.93%	0.82	**2.07E-04**	21.83%	0.02%	0.81	8.87E-01	22.19%	0.38%	0.81	**1.95E-02**
22	21.83%	0.02%	0.81	7.85E-01	21.85%	0.04%	0.81	5.82E-01	21.85%	0.04%	0.81	5.69E-01

Supplementary Table S10. Summary of polygenic scores derived from T-lymphocytes subsets (A-C) and CD4/CD8 ratio (D) T-lymphocytes by $P_{discovery}$ thresholds for non-LS (664 cases and 2,086 controls) using pruned sets. Note that the pairwise LD between pairs of genic- and intergenic-SNPs is $r^2 < 0.25$.

A. CD3+

	CD3 pruned-SNPs				CD3 genic-SNPs				CD3 intergenic-SNPs			
$P_{discovery}$	Nagelkerke R^2 (%)	% explained variance	C-statistic	SCORE P	Nagelkerke R^2 (%)	% explained variance	C-statistic	SCORE P	Nagelkerke R^2 (%)	% explained variance	C-statistic	SCORE P
Baseline	17.40%	0.00%	0.73	**1.23E-73**	17.40%	0.00%	0.73	**1.23E-73**	17.40%	0.00%	0.73	**1.23E-73**
5.0E-08	–	–	–	–	–	–	–	–	–	–	–	–
5.0E-07	–	–	–	–	–	–	–	–	–	–	–	–
5.0E-06	17.50%	0.10%	0.74	**1.63E-03**	8.18%	9.22%	0.78	9.50E-01	–	–	–	–
5.0E-05	16.99%	0.42%	0.73	5.93E-01	8.18%	9.22%	0.78	9.50E-01	17.44%	0.04%	0.74	**3.01E-03**
5.0E-04	16.99%	0.42%	0.73	6.17E-01	8.19%	9.22%	0.77	9.26E-01	17.06%	0.35%	0.74	2.06E-01
5.0E-03	17.01%	0.40%	0.74	4.29E-01	8.96%	8.44%	0.76	2.77E-01	16.98%	0.42%	0.73	6.56E-01
5.0E-02	16.99%	0.41%	0.73	5.48E-01	9.53%	7.87%	0.78	1.52E-01	16.97%	0.43%	0.73	9.89E-01
1.0E-01	16.99%	0.42%	0.73	6.31E-01	9.46%	7.95%	0.75	1.63E-01	17.02%	0.39%	0.73	3.74E-01
2.0E-01	17.00%	0.41%	0.73	4.84E-01	8.35%	9.06%	0.77	6.17E-01	17.03%	0.38%	0.73	3.07E-01
3.0E-01	16.99%	0.42%	0.73	6.15E-01	8.51%	8.89%	0.76	4.78E-01	17.04%	0.36%	0.73	2.59E-01
4.0E-01	16.97%	0.43%	0.73	9.27E-01	8.26%	9.14%	0.77	7.21E-01	16.99%	0.41%	0.73	5.79E-01
5.0E-01	16.98%	0.43%	0.73	7.65E-01	8.19%	9.21%	0.77	8.86E-01	16.98%	0.43%	0.73	8.41E-01
6.0E-01	16.97%	0.43%	0.73	9.95E-01	8.20%	9.21%	0.77	8.68E-01	16.98%	0.43%	0.73	7.39E-01
7.0E-01	16.98%	0.43%	0.73	8.71E-01	8.20%	9.20%	0.77	8.52E-01	16.98%	0.42%	0.73	6.66E-01
8.0E-01	16.98%	0.43%	0.73	7.74E-01	8.20%	9.20%	0.77	8.57E-01	16.99%	0.42%	0.73	6.09E-01
9.0E-01	16.98%	0.43%	0.73	7.46E-01	8.20%	9.21%	0.77	8.69E-01	16.99%	0.41%	0.73	5.36E-01
1.0E+00	16.98%	0.43%	0.73	7.36E-01	8.21%	9.20%	0.77	8.42E-01	17.00%	0.41%	0.73	5.24E-01

B. CD4+

$P_{discovery}$	CD4 pruned-SNPs				CD4 genic-SNPs				CD4 intergenic-SNPs			
	Nagelkerke R^2 (%)	% explained variance	C-statistic	SCORE P	Nagelkerke R^2 (%)	% explained variance	C-statistic	SCORE P	Nagelkerke R^2 (%)	% explained variance	C-statistic	SCORE P
Baseline	17.40%	0.00%	0.73	**1.23E-73**	17.40%	0.00%	0.73	**1.23E-73**	17.40%	0.00%	0.73	**1.23E-73**
5.0E-08	–	–	–	–	–	–	–	–	–	–	–	–
5.0E-07	–	–	–	–	–	–	–	–	–	–	–	–
5.0E-06	17.50%	0.10%	0.74	**1.63E-03**	8.18%	9.22%	0.78	9.50E-01	–	–	–	–
5.0E-05	17.00%	0.41%	0.73	4.99E-01	8.58%	8.83%	0.80	4.39E-01	–	–	–	–
5.0E-04	17.07%	0.33%	0.73	1.72E-01	8.54%	8.86%	0.79	4.61E-01	17.01%	0.39%	0.73	3.95E-01
5.0E-03	16.99%	0.41%	0.73	5.28E-01	8.90%	8.51%	0.78	2.98E-01	16.97%	0.43%	0.73	9.62E-01
5.0E-02	16.98%	0.43%	0.73	7.89E-01	9.32%	8.09%	0.80	1.89E-01	16.98%	0.42%	0.73	7.27E-01
1.0E-01	16.99%	0.42%	0.73	6.36E-01	9.24%	8.16%	0.78	2.05E-01	16.97%	0.43%	0.73	9.43E-01
2.0E-01	16.98%	0.43%	0.73	8.64E-01	8.41%	8.99%	0.77	5.55E-01	16.98%	0.42%	0.73	6.61E-01
3.0E-01	17.01%	0.40%	0.73	4.32E-01	8.27%	9.13%	0.77	7.09E-01	16.97%	0.43%	0.73	9.44E-01
4.0E-01	16.99%	0.42%	0.73	5.98E-01	8.19%	9.22%	0.78	9.34E-01	16.98%	0.43%	0.73	7.99E-01
5.0E-01	16.99%	0.41%	0.73	5.63E-01	8.18%	9.22%	0.79	9.81E-01	16.98%	0.42%	0.73	6.94E-01
6.0E-01	16.99%	0.41%	0.73	5.33E-01	8.18%	9.22%	0.79	9.74E-01	16.98%	0.42%	0.73	6.60E-01
7.0E-01	16.99%	0.42%	0.73	6.18E-01	8.19%	9.21%	0.77	9.07E-01	16.98%	0.43%	0.73	7.72E-01
8.0E-01	16.99%	0.42%	0.73	5.86E-01	8.20%	9.20%	0.77	8.51E-01	16.98%	0.42%	0.73	6.75E-01
9.0E-01	16.99%	0.41%	0.73	5.65E-01	8.20%	9.21%	0.77	8.72E-01	16.98%	0.42%	0.73	6.44E-01
1.0E+00	16.99%	0.41%	0.73	5.50E-01	8.20%	9.21%	0.77	8.72E-01	16.99%	0.42%	0.73	6.15E-01

C. CD8+

$P_{discovery}$	CD8 pruned-SNPs				CD8 genic-SNPs				CD8 intergenic-SNPs			
	Nagelkerke R^2 (%)	% explained variance	C-statistic	SCORE P	Nagelkerke R^2 (%)	% explained variance	C-statistic	SCORE P	Nagelkerke R^2 (%)	% explained variance	C-statistic	SCORE P
Baseline	17.40%	0.00%	0.73	**1.23E-73**	17.40%	0.00%	0.73	**1.23E-73**	17.40%	0.00%	0.73	**1.23E-73**
5.0E-08	17.07%	0.34%	0.74	1.84E-01	10.42%	6.99%	0.82	6.85E-02	16.97%	0.43%	0.73	9.13E-01
5.0E-07	17.00%	0.40%	0.73	4.64E-01	14.72%	2.69%	0.88	**3.03E-03**	16.97%	0.43%	0.73	9.13E-01
5.0E-06	17.04%	0.37%	0.74	2.66E-01	13.88%	3.53%	0.89	**4.95E-03**	16.98%	0.43%	0.73	7.83E-01
5.0E-05	17.03%	0.37%	0.73	2.97E-01	15.76%	1.64%	0.89	**1.58E-03**	17.05%	0.35%	0.73	2.27E-01
5.0E-04	16.97%	0.43%	0.73	9.51E-01	17.64%	0.24%	0.90	**3.88E-04**	17.01%	0.39%	0.73	3.86E-01
5.0E-03	16.98%	0.43%	0.73	8.54E-01	12.57%	4.84%	0.81	**1.05E-02**	17.00%	0.40%	0.73	4.68E-01
5.0E-02	16.98%	0.43%	0.73	7.84E-01	8.46%	8.94%	0.75	5.09E-01	17.00%	0.40%	0.74	4.75E-01
1.0E-01	16.97%	0.43%	0.73	9.78E-01	8.25%	9.16%	0.77	7.48E-01	16.98%	0.42%	0.73	7.18E-01
2.0E-01	16.98%	0.42%	0.73	6.48E-01	8.36%	9.05%	0.79	6.02E-01	17.03%	0.37%	0.73	2.93E-01
3.0E-01	17.01%	0.39%	0.74	3.98E-01	8.54%	8.86%	0.80	4.55E-01	17.03%	0.37%	0.73	2.87E-01
4.0E-01	17.02%	0.38%	0.74	3.26E-01	8.49%	8.91%	0.77	4.90E-01	17.12%	0.29%	0.74	9.92E-02
5.0E-01	17.04%	0.37%	0.74	2.70E-01	8.50%	8.91%	0.78	4.86E-01	17.13%	0.27%	0.74	8.46E-02
6.0E-01	17.02%	0.39%	0.73	3.56E-01	8.48%	8.93%	0.77	5.01E-01	17.10%	0.30%	0.74	1.19E-01
7.0E-01	17.00%	0.40%	0.73	4.43E-01	8.40%	9.00%	0.77	5.60E-01	17.07%	0.33%	0.73	1.77E-01
8.0E-01	17.01%	0.40%	0.73	4.28E-01	8.39%	9.02%	0.77	5.76E-01	17.07%	0.33%	0.73	1.78E-01
9.0E-01	17.01%	0.40%	0.73	4.32E-01	8.36%	9.04%	0.77	5.97E-01	17.07%	0.34%	0.73	1.78E-01
1.0E+00	17.01%	0.40%	0.73	4.38E-01	8.35%	9.05%	0.77	6.11E-01	17.06%	0.34%	0.73	1.89E-01

D. CD4/CD8

$P_{discovery}$	CD4/CD8 pruned-SNPs				CD4/CD8 genic-SNPs				CD4/CD8 intergenic-SNPs			
	Nagelkerke R^2 (%)	% explained variance	C-statistic	SCORE P	Nagelkerke R^2 (%)	% explained variance	C-statistic	SCORE P	Nagelkerke R^2 (%)	% explained variance	C-statistic	SCORE P
Baseline	17.40%	0.00%	0.73	**1.23E-73**	17.40%	0.00%	0.73	**1.23E-73**	17.40%	0.00%	0.73	**1.23E-73**
5.0E-08	17.03%	0.37%	0.74	2.88E-01	10.42%	6.99%	0.82	6.85E-02	16.97%	0.43%	0.73	9.13E-01
5.0E-07	16.99%	0.42%	0.73	6.16E-01	14.72%	2.69%	0.88	**3.03E-03**	16.97%	0.43%	0.73	9.13E-01
5.0E-06	17.02%	0.38%	0.74	3.46E-01	13.88%	3.53%	0.89	**4.95E-03**	16.98%	0.43%	0.73	7.83E-01
5.0E-05	17.04%	0.36%	0.74	2.58E-01	15.76%	1.64%	0.89	**1.58E-03**	17.05%	0.35%	0.73	2.27E-01
5.0E-04	16.99%	0.41%	0.73	5.63E-01	17.64%	0.24%	0.90	**3.88E-04**	17.01%	0.39%	0.73	3.86E-01
5.0E-03	17.02%	0.39%	0.74	3.61E-01	12.57%	4.84%	0.81	**1.05E-02**	17.00%	0.40%	0.73	4.68E-01
5.0E-02	17.04%	0.36%	0.74	2.61E-01	8.46%	8.94%	0.75	5.09E-01	17.00%	0.40%	0.74	4.75E-01
1.0E-01	17.06%	0.35%	0.73	2.12E-01	8.25%	9.16%	0.77	7.48E-01	16.98%	0.42%	0.73	7.18E-01
2.0E-01	16.98%	0.43%	0.73	8.42E-01	8.36%	9.05%	0.79	6.02E-01	17.03%	0.37%	0.73	2.93E-01
3.0E-01	16.97%	0.43%	0.73	9.79E-01	8.54%	8.86%	0.80	4.55E-01	17.03%	0.37%	0.73	2.87E-01
4.0E-01	16.97%	0.43%	0.73	9.16E-01	8.49%	8.91%	0.77	4.90E-01	17.12%	0.29%	0.74	9.92E-02
5.0E-01	16.99%	0.41%	0.73	5.56E-01	8.50%	8.91%	0.78	4.86E-01	17.13%	0.27%	0.74	8.46E-02
6.0E-01	17.00%	0.40%	0.73	4.68E-01	8.48%	8.93%	0.77	5.01E-01	17.10%	0.30%	0.74	1.19E-01
7.0E-01	17.01%	0.39%	0.73	3.99E-01	8.40%	9.00%	0.77	5.60E-01	17.07%	0.33%	0.73	1.77E-01
8.0E-01	17.01%	0.39%	0.73	4.05E-01	8.39%	9.02%	0.77	5.76E-01	17.07%	0.33%	0.73	1.78E-01
9.0E-01	17.02%	0.39%	0.73	3.72E-01	8.36%	9.04%	0.77	5.97E-01	17.07%	0.34%	0.73	1.78E-01
1.0E+00	17.01%	0.39%	0.73	3.84E-01	8.35%	9.05%	0.77	6.11E-01	17.06%	0.34%	0.73	1.89E-01

Supplementary Table S11. Summary of polygenic scores derived from T-lymphocytes subsets (A-C) and CD4:CD8 ratio (D) T-lymphocytes by chromosomes for non-LS (664 cases and 2,086 controls) using pruned sets. Note that the pairwise LD between pairs of genic- and intergenic-SNPs is $r^2 < 0.25$.

A. CD3+

CHR	CD3 pruned-SNPs Nagelkerke R^2 (%)	% explained variance	C-statistic	SCORE P	CD3 genic-SNPs Nagelkerke R^2 (%)	% explained variance	C-statistic	SCORE P	CD3 intergenic-SNPs Nagelkerke R^2 (%)	% explained variance	C-statistic	SCORE P
Baseline	17.40%	0.00%	0.73	**1.23E-73**	17.40%	0.00%	0.73	**1.23E-73**	17.40%	0.00%	0.73	**1.23E-73**
1	17.09%	0.32%	0.74	1.40E-01	8.29%	9.11%	0.79	6.78E-01	17.07%	0.34%	0.74	1.80E-01
2	17.34%	0.06%	0.74	**8.15E-03**	8.21%	9.20%	0.77	8.35E-01	16.98%	0.42%	0.73	6.48E-01
3	16.98%	0.42%	0.73	6.84E-01	8.26%	9.15%	0.78	7.31E-01	16.97%	0.43%	0.73	9.68E-01
4	17.07%	0.33%	0.74	1.74E-01	8.22%	9.19%	0.79	8.15E-01	17.12%	0.28%	0.74	9.24E-02
5	17.18%	0.22%	0.74	**4.63E-02**	9.68%	7.73%	0.79	1.33E-01	17.05%	0.35%	0.73	2.21E-01
6	17.01%	0.40%	0.73	4.19E-01	9.27%	8.13%	0.81	2.00E-01	17.03%	0.38%	0.73	3.18E-01
7	17.00%	0.40%	0.73	4.65E-01	10.44%	6.96%	0.78	6.32E-02	17.00%	0.41%	0.74	5.20E-01
8	16.97%	0.43%	0.73	9.85E-01	8.19%	9.22%	0.76	9.11E-01	17.06%	0.35%	0.73	2.08E-01
9	17.03%	0.37%	0.73	3.02E-01	8.39%	9.01%	0.79	5.70E-01	16.97%	0.43%	0.73	9.41E-01
10	17.02%	0.38%	0.73	3.33E-01	8.19%	9.21%	0.76	8.83E-01	17.04%	0.36%	0.74	2.63E-01
11	16.99%	0.42%	0.73	6.19E-01	8.20%	9.21%	0.78	8.65E-01	17.00%	0.40%	0.74	4.64E-01
12	17.08%	0.33%	0.73	1.59E-01	8.27%	9.13%	0.79	7.09E-01	16.98%	0.42%	0.73	6.52E-01
13	16.98%	0.42%	0.73	6.45E-01	8.18%	9.22%	0.77	9.90E-01	17.01%	0.39%	0.73	3.79E-01
14	16.99%	0.41%	0.73	5.69E-01	8.43%	8.97%	0.77	5.31E-01	17.00%	0.41%	0.73	4.90E-01
15	17.04%	0.37%	0.74	2.74E-01	9.19%	8.21%	0.79	2.15E-01	17.11%	0.30%	0.74	1.13E-01
16	17.26%	0.14%	0.74	**1.90E-02**	9.03%	8.37%	0.81	2.54E-01	17.17%	0.24%	0.74	5.59E-02
17	17.02%	0.38%	0.74	3.35E-01	9.99%	7.42%	0.78	1.03E-01	17.02%	0.39%	0.74	3.71E-01
18	16.98%	0.43%	0.73	7.76E-01	8.41%	8.99%	0.78	5.51E-01	16.97%	0.43%	0.73	9.50E-01
19	17.20%	0.21%	0.74	**3.96E-02**	8.24%	9.17%	0.77	7.70E-01	17.33%	0.07%	0.74	**9.12E-03**
20	16.97%	0.43%	0.73	9.15E-01	8.89%	8.51%	0.76	2.97E-01	17.00%	0.40%	0.73	4.74E-01
21	16.97%	0.43%	0.73	8.84E-01	11.79%	5.61%	0.82	**1.67E-02**	16.99%	0.42%	0.73	6.38E-01
22	16.98%	0.43%	0.73	7.44E-01	11.98%	5.42%	0.82	**1.83E-02**	16.99%	0.42%	0.73	6.11E-01

B. CD4+

CHR	CD4 pruned-SNPs				CD4 genic-SNPs				CD4 intergenic-SNPs			
	Nagelkerke R^2 (%)	% explained variance	C-statistic	SCORE P	Nagelkerke R^2 (%)	% explained variance	C-statistic	SCORE P	Nagelkerke R^2 (%)	% explained variance	C-statistic	SCORE P
Baseline	17.40%	0.00%	0.73	**1.23E-73**	17.40%	0.00%	0.73	**1.23E-73**	17.40%	0.00%	0.73	**1.23E-73**
1	17.04%	0.37%	0.74	2.75E-01	8.34%	9.07%	0.78	6.28E-01	17.11%	0.30%	0.74	1.13E-01
2	17.27%	0.14%	0.74	**1.83E-02**	8.24%	9.17%	0.79	7.66E-01	16.97%	0.43%	0.73	8.78E-01
3	16.98%	0.42%	0.73	7.28E-01	8.20%	9.20%	0.78	8.54E-01	16.97%	0.43%	0.73	9.03E-01
4	17.02%	0.39%	0.73	3.58E-01	8.34%	9.06%	0.79	6.19E-01	17.06%	0.35%	0.74	2.07E-01
5	17.23%	0.17%	0.74	**2.67E-02**	9.68%	7.73%	0.81	1.34E-01	17.17%	0.23%	0.74	5.40E-02
6	16.98%	0.42%	0.73	6.71E-01	8.78%	8.62%	0.78	3.36E-01	16.99%	0.41%	0.73	5.43E-01
7	16.98%	0.43%	0.73	7.37E-01	10.60%	6.81%	0.77	5.44E-02	16.98%	0.43%	0.73	7.86E-01
8	16.97%	0.43%	0.73	9.87E-01	8.35%	9.06%	0.77	6.14E-01	17.02%	0.38%	0.73	3.26E-01
9	17.01%	0.39%	0.73	3.83E-01	8.24%	9.17%	0.77	7.71E-01	17.00%	0.40%	0.73	4.65E-01
10	17.01%	0.40%	0.73	4.20E-01	8.18%	9.22%	0.76	9.47E-01	16.99%	0.41%	0.73	5.45E-01
11	16.98%	0.43%	0.73	7.47E-01	8.18%	9.22%	0.77	9.69E-01	16.98%	0.43%	0.73	7.57E-01
12	17.31%	0.09%	0.74	**1.17E-02**	8.28%	9.12%	0.79	6.95E-01	17.06%	0.34%	0.74	1.91E-01
13	16.98%	0.43%	0.73	7.57E-01	8.38%	9.02%	0.78	5.78E-01	16.98%	0.42%	0.73	6.84E-01
14	16.98%	0.42%	0.73	7.02E-01	8.24%	9.17%	0.77	7.64E-01	17.11%	0.30%	0.73	1.14E-01
15	17.03%	0.37%	0.74	2.88E-01	8.51%	8.89%	0.78	4.75E-01	17.20%	0.20%	0.74	**3.69E-02**
16	17.11%	0.30%	0.74	1.10E-01	8.59%	8.81%	0.81	4.28E-01	17.07%	0.33%	0.74	1.68E-01
17	16.99%	0.41%	0.74	5.26E-01	10.21%	7.19%	0.81	8.34E-02	16.98%	0.43%	0.73	7.83E-01
18	16.97%	0.43%	0.73	9.42E-01	8.29%	9.12%	0.79	6.85E-01	16.98%	0.42%	0.73	6.58E-01
19	17.36%	0.04%	0.74	**6.80E-03**	8.20%	9.21%	0.77	8.72E-01	17.66%	0.25%	0.74	**3.30E-04**
20	16.99%	0.42%	0.74	6.36E-01	8.48%	8.92%	0.77	4.96E-01	16.99%	0.41%	0.73	5.62E-01
21	16.97%	0.43%	0.73	9.05E-01	13.05%	4.35%	0.84	**4.80E-03**	16.99%	0.42%	0.74	6.32E-01
22	16.98%	0.43%	0.73	8.17E-01	13.28%	4.12%	0.86	**6.00E-03**	16.98%	0.43%	0.73	8.31E-01

C. CD8+

CHR	CD8 pruned-SNPs				CD8 genic-SNPs				CD8 intergenic-SNPs			
	Nagelkerke R^2 (%)	% explained variance	C-statistic	SCORE P	Nagelkerke R^2 (%)	% explained variance	C-statistic	SCORE P	Nagelkerke R^2 (%)	% explained variance	C-statistic	SCORE P
Baseline	17.40%	0.00%	0.73	**1.23E-73**	17.40%	0.00%	0.73	**1.23E-73**	17.40%	0.00%	0.73	**1.23E-73**
1	17.08%	0.33%	0.74	1.65E-01	8.20%	9.21%	0.78	8.76E-01	16.98%	0.43%	0.73	7.75E-01
2	17.29%	0.12%	0.74	**1.46E-02**	8.72%	8.68%	0.78	3.65E-01	17.05%	0.35%	0.74	2.25E-01
3	16.98%	0.42%	0.73	6.50E-01	8.25%	9.16%	0.79	7.50E-01	16.98%	0.43%	0.73	8.30E-01
4	17.06%	0.35%	0.73	2.11E-01	8.27%	9.13%	0.79	7.09E-01	17.09%	0.31%	0.74	1.31E-01
5	17.03%	0.37%	0.74	2.88E-01	10.29%	7.12%	0.81	7.22E-02	16.97%	0.43%	0.73	8.99E-01
6	16.97%	0.43%	0.73	9.52E-01	14.06%	3.34%	0.88	**4.03E-03**	17.01%	0.39%	0.73	4.06E-01
7	17.04%	0.36%	0.74	2.47E-01	9.74%	7.66%	0.80	1.25E-01	17.12%	0.29%	0.74	1.02E-01
8	16.98%	0.43%	0.73	8.21E-01	8.18%	9.22%	0.79	9.43E-01	17.06%	0.34%	0.73	1.98E-01
9	17.01%	0.39%	0.73	3.79E-01	9.04%	8.36%	0.80	2.53E-01	16.98%	0.42%	0.73	6.60E-01
10	16.99%	0.41%	0.73	5.80E-01	8.21%	9.20%	0.79	8.42E-01	17.05%	0.35%	0.74	2.23E-01
11	16.97%	0.43%	0.73	9.98E-01	8.18%	9.22%	0.79	9.79E-01	16.99%	0.42%	0.73	6.37E-01
12	16.97%	0.43%	0.73	9.34E-01	8.20%	9.21%	0.78	8.74E-01	17.00%	0.41%	0.73	4.91E-01
13	16.97%	0.43%	0.73	9.51E-01	8.25%	9.15%	0.79	7.45E-01	17.02%	0.38%	0.73	3.32E-01
14	17.19%	0.21%	0.74	**4.18E-02**	8.64%	8.76%	0.76	4.01E-01	17.02%	0.38%	0.74	3.38E-01
15	17.03%	0.37%	0.74	2.87E-01	9.87%	7.54%	0.79	1.08E-01	17.04%	0.36%	0.74	2.46E-01
16	17.20%	0.20%	0.74	**3.75E-02**	8.35%	9.05%	0.80	6.05E-01	17.13%	0.28%	0.74	8.96E-02
17	16.98%	0.42%	0.74	6.60E-01	8.66%	8.75%	0.80	3.96E-01	17.03%	0.38%	0.74	3.19E-01
18	16.97%	0.43%	0.73	9.58E-01	8.36%	9.05%	0.79	6.06E-01	16.97%	0.43%	0.73	9.03E-01
19	17.00%	0.41%	0.73	4.93E-01	8.28%	9.12%	0.78	6.95E-01	17.04%	0.36%	0.73	2.56E-01
20	16.98%	0.42%	0.73	7.22E-01	8.69%	8.71%	0.76	3.79E-01	17.19%	0.22%	0.73	**4.36E-02**
21	17.03%	0.37%	0.74	2.94E-01	9.13%	8.28%	0.80	2.25E-01	17.13%	0.28%	0.74	9.01E-02
22	16.98%	0.42%	0.73	6.85E-01	10.57%	6.84%	0.78	5.84E-02	17.04%	0.36%	0.73	2.49E-01

D. CD4/CD8

CHR	CD4/CD8 pruned-SNPs				CD4/CD8 genic-SNPs				CD4/CD8 intergenic-SNPs			
	Nagelkerke R^2 (%)	% explained variance	C-statistic	SCORE P	Nagelkerke R^2 (%)	% explained variance	C-statistic	SCORE P	Nagelkerke R^2 (%)	% explained variance	C-statistic	SCORE P
Baseline	17.40%	0.00%	0.73	**1.23E-73**	17.40%	0.00%	0.73	**1.23E-73**	17.40%	0.00%	0.73	**1.23E-73**
1	16.99%	0.42%	0.73	6.35E-01	8.20%	9.21%	0.78	8.76E-01	16.98%	0.43%	0.73	7.75E-01
2	16.98%	0.43%	0.73	8.60E-01	8.72%	8.68%	0.78	3.65E-01	17.05%	0.35%	0.74	2.25E-01
3	16.98%	0.43%	0.73	7.48E-01	8.25%	9.16%	0.79	7.50E-01	16.98%	0.43%	0.73	8.30E-01
4	16.97%	0.43%	0.73	9.08E-01	8.27%	9.13%	0.79	7.09E-01	17.09%	0.31%	0.74	1.31E-01
5	16.99%	0.42%	0.73	5.97E-01	10.29%	7.12%	0.81	7.22E-02	16.97%	0.43%	0.73	8.99E-01
6	16.98%	0.43%	0.73	8.55E-01	14.06%	3.34%	0.88	**4.03E-03**	17.01%	0.39%	0.73	4.06E-01
7	16.99%	0.41%	0.74	5.53E-01	9.74%	7.66%	0.80	1.25E-01	17.12%	0.29%	0.74	1.02E-01
8	16.99%	0.41%	0.73	5.26E-01	8.18%	9.22%	0.79	9.43E-01	17.06%	0.34%	0.73	1.98E-01
9	17.08%	0.33%	0.74	1.62E-01	9.04%	8.36%	0.80	2.53E-01	16.98%	0.42%	0.73	6.60E-01
10	16.98%	0.43%	0.73	7.51E-01	8.21%	9.20%	0.79	8.42E-01	17.05%	0.35%	0.74	2.23E-01
11	16.99%	0.42%	0.73	6.02E-01	8.18%	9.22%	0.79	9.79E-01	16.99%	0.42%	0.73	6.37E-01
12	17.31%	0.09%	0.74	**1.12E-02**	8.20%	9.21%	0.78	8.74E-01	17.00%	0.41%	0.73	4.91E-01
13	16.99%	0.42%	0.74	6.28E-01	8.25%	9.15%	0.79	7.45E-01	17.02%	0.38%	0.73	3.32E-01
14	17.09%	0.31%	0.74	1.35E-01	8.64%	8.76%	0.76	4.01E-01	17.02%	0.38%	0.74	3.38E-01
15	16.98%	0.43%	0.73	7.65E-01	9.87%	7.54%	0.79	1.08E-01	17.04%	0.36%	0.74	2.46E-01
16	16.97%	0.43%	0.73	9.96E-01	8.35%	9.05%	0.80	6.05E-01	17.13%	0.28%	0.74	8.96E-02
17	16.97%	0.43%	0.73	9.95E-01	8.66%	8.75%	0.80	3.96E-01	17.03%	0.38%	0.74	3.19E-01
18	16.98%	0.43%	0.73	7.70E-01	8.36%	9.05%	0.79	6.06E-01	16.97%	0.43%	0.73	9.03E-01
19	17.07%	0.33%	0.74	1.73E-01	8.28%	9.12%	0.78	6.95E-01	17.04%	0.36%	0.73	2.56E-01
20	17.00%	0.40%	0.73	4.61E-01	8.69%	8.71%	0.76	3.79E-01	17.19%	0.22%	0.73	**4.36E-02**
21	17.21%	0.20%	0.74	**3.57E-02**	9.13%	8.28%	0.80	2.25E-01	17.13%	0.28%	0.74	9.01E-02
22	16.99%	0.41%	0.73	5.81E-01	10.57%	6.84%	0.78	5.84E-02	17.04%	0.36%	0.73	2.49E-01

Supplementary Table S12. Summary of polygenic scores derived from T-lymphocytes subsets and CD4/CD8 ratio by $P_{discovery}$ thresholds (A) and by chromosomes (B) for LS *HLA-DRB1*03* carriers (253 cases and 512 controls) using pruned sets.

A. By $P_{discovery}$

	CD3 pruned-SNPs				CD4 pruned-SNPs				CD8 pruned-SNPs				CD4/CD8 pruned-SNPs			
$P_{discovery}$	Nagelkerke R^2 (%)	% explained variance	C-statistic	SCORE P	Nagelkerke R^2 (%)	% explained variance	C-statistic	SCORE P	Nagelkerke R^2 (%)	% explained variance	C-statistic	SCORE P	Nagelkerke R^2 (%)	% explained variance	C-statistic	SCORE P
Baseline	36.47%	0.00%	0.84	**3.45E-50**	36.47%	0.00%	0.84	**3.45E-50**	36.47%	0.00%	0.84	**3.45E-50**	36.47%	0.00%	0.84	**3.45E-50**
5.0E-08	–	–	–	–	–	–	–	–	36.48%	0.01%	0.84	8.05E-01	–	–	–	–
5.0E-07	–	–	–	–	–	–	–	–	36.48%	0.01%	0.84	7.97E-01	–	–	–	–
5.0E-06	36.47%	0.00%	0.84	9.77E-01	36.47%	0.00%	0.84	9.77E-01	–	–	–	–	–	–	–	–
5.0E-05	36.47%	0.00%	0.84	9.22E-01	36.50%	0.03%	0.84	6.52E-01	36.50%	0.03%	0.84	6.55E-01	36.62%	0.15%	0.84	3.06E-01
5.0E-04	36.62%	0.15%	0.84	3.03E-01	36.63%	0.16%	0.84	2.87E-01	36.78%	0.31%	0.84	1.40E-01	36.51%	0.04%	0.84	6.02E-01
5.0E-03	–	–	–	–	36.88%	0.40%	0.84	9.12E-02	–	–	–	–	36.55%	0.08%	0.84	4.51E-01
5.0E-02	36.49%	0.02%	0.84	6.99E-01	–	–	–	–	–	–	–	–	–	–	–	–
1.0E-01	–	–	–	–	–	–	–	–	–	–	–	–	–	–	–	–
2.0E-01	36.48%	0.01%	0.84	8.07E-01	36.48%	0.01%	0.84	7.80E-01	–	–	–	–	–	–	–	–
3.0E-01	–	–	–	–	36.47%	0.00%	0.84	9.25E-01	–	–	–	–	–	–	–	–
4.0E-01	–	–	–	–	36.49%	0.02%	0.84	7.28E-01	–	–	–	–	–	–	–	–
5.0E-01	–	–	–	–	–	–	–	–	–	–	–	–	36.53%	0.05%	0.84	5.35E-01
6.0E-01	–	–	–	–	–	–	–	–	–	–	–	–	–	–	–	–
7.0E-01	–	–	–	–	36.49%	0.02%	0.84	7.01E-01	–	–	–	–	–	–	–	–
8.0E-01	36.50%	0.02%	0.84	6.85E-01	–	–	–	–	–	–	–	–	–	–	–	–
9.0E-01	–	–	–	–	36.49%	0.02%	0.84	7.06E-01	–	–	–	–	–	–	–	–
1.0E+00	36.49%	0.02%	0.84	7.01E-01	36.50%	0.02%	0.84	6.84E-01	–	–	–	–	–	–	–	–

B. By chromosome

CHR	CD3 pruned-SNPs				CD4 pruned-SNPs				CD8 pruned-SNPs				CD4/CD8 pruned-SNPs			
	Nagelkerke R^2 (%)	% explained variance	C-statistic	SCORE P	Nagelkerke R^2 (%)	% explained variance	C-statistic	SCORE P	Nagelkerke R^2 (%)	% explained variance	C-statistic	SCORE P	Nagelkerke R^2 (%)	% explained variance	C-statistic	SCORE P
Baseline	36.47%	0.00%	0.84	**3.45E-50**	36.47%	0.00%	0.84	**3.45E-50**	36.47%	0.00%	0.84	**3.45E-50**	36.47%	0.00%	0.84	**3.45E-50**
1	36.83%	0.35%	0.85	1.14E-01	–	–	–	–	37.07%	0.60%	0.85	**3.96E-02**	36.68%	0.21%	0.85	2.26E-01
2	36.47%	0.00%	0.84	9.48E-01	36.48%	0.00%	0.84	8.80E-01	–	–	–	–	36.48%	0.00%	0.84	8.56E-01
3	36.55%	0.08%	0.84	4.55E-01	36.48%	0.01%	0.84	7.89E-01	36.59%	0.11%	0.84	3.70E-01	36.55%	0.08%	0.84	4.51E-01
4	36.55%	0.07%	0.84	4.72E-01	36.67%	0.19%	0.84	2.42E-01	–	–	–	–	–	–	–	–
5	36.48%	0.00%	0.84	8.58E-01	36.68%	0.21%	0.84	2.20E-01	–	–	–	–	–	–	–	–
6	36.48%	0.01%	0.84	7.68E-01	–	–	–	–	–	–	–	–	36.71%	0.23%	0.85	1.99E-01
7	36.49%	0.02%	0.84	6.92E-01	–	–	–	–	–	–	–	–	36.71%	0.24%	0.85	1.92E-01
8	36.53%	0.06%	0.85	5.26E-01	–	–	–	–	–	–	–	–	36.52%	0.05%	0.84	5.62E-01
9	–	–	–	–	36.53%	0.05%	0.84	5.35E-01	36.53%	0.06%	0.84	5.11E-01	36.60%	0.13%	0.84	3.36E-01
10	–	–	–	–	36.93%	0.46%	0.85	7.35E-02	36.68%	0.21%	0.84	2.23E-01	36.48%	0.01%	0.84	8.06E-01
11	–	–	–	–	–	–	–	–	36.62%	0.15%	0.85	3.02E-01	–	–	–	–
12	–	–	–	–	36.51%	0.03%	0.84	6.26E-01	–	–	–	–	–	–	–	–
13	36.49%	0.02%	0.84	7.02E-01	36.48%	0.01%	0.84	7.67E-01	36.52%	0.05%	0.84	5.46E-01	–	–	–	–
14	–	–	–	–	–	–	–	–	–	–	–	–	36.53%	0.06%	0.84	5.26E-01
15	36.49%	0.02%	0.84	7.18E-01	36.58%	0.11%	0.85	3.81E-01	–	–	–	–	37.08%	0.60%	0.85	**3.92E-02**
16	–	–	–	–	–	–	–	–	–	–	–	–	36.57%	0.10%	0.84	4.09E-01
17	36.67%	0.20%	0.85	2.32E-01	–	–	–	–	–	–	–	–	36.47%	0.00%	0.84	9.35E-01
18	–	–	–	–	–	–	–	–	–	–	–	–	36.53%	0.05%	0.84	5.40E-01
19	36.54%	0.07%	0.84	4.91E-01	–	–	–	–	–	–	–	–	–	–	–	–
20	36.55%	0.08%	0.84	4.62E-01	–	–	–	–	–	–	–	–	36.47%	0.00%	0.84	9.77E-01
21	–	–	–	–	–	–	–	–	–	–	–	–	38.04%	1.57%	0.85	**9.44E-04**
22	36.48%	0.01%	0.84	7.98E-01	36.51%	0.04%	0.84	5.85E-01	–	–	–	–	36.65%	0.18%	0.84	2.64E-01

Supplementary Table S13. Summary of polygenic scores derived from T-lymphocytes subsets and CD4/CD8 ratio by Pdiscovery thresholds (A) and by chromosomes (B) for LS *HLA-DRB1*03* non-carriers (131 cases and 1,574 controls) using pruned sets.

A. By $P_{discovery}$

	CD3 pruned-SNPs				CD4 pruned-SNPs				CD8 pruned-SNPs				CD4/CD8 pruned-SNPs			
$P_{discovery}$	Nagelkerke R^2 (%)	% explained variance	C-statistic	SCORE P	Nagelkerke R^2 (%)	% explained variance	C-statistic	SCORE P	Nagelkerke R^2 (%)	% explained variance	C-statistic	SCORE P	Nagelkerke R^2 (%)	% explained variance	C-statistic	SCORE P
Baseline	11.06%	0.00%	0.77	**1.55E-17**	11.06%	0.00%	0.77	**1.55E-17**	11.06%	0.00%	0.77	**1.55E-17**	11.06%	0.00%	0.77	**1.55E-17**
5.0E-08	–	–	–	–	–	–	–	–	11.13%	0.07%	0.77	4.92E-01	11.74%	0.68%	0.76	**3.18E-02**
5.0E-07	–	–	–	–	–	–	–	–	11.32%	0.26%	0.77	1.88E-01	11.62%	0.56%	0.77	5.31E-02
5.0E-06	11.10%	0.04%	0.77	6.05E-01	11.10%	0.04%	0.77	6.05E-01	–	–	–	–	11.67%	0.61%	0.77	**4.16E-02**
5.0E-05	–	–	–	–	11.21%	0.15%	0.77	3.18E-01	–	–	–	–	11.69%	0.63%	0.77	**3.85E-02**
5.0E-04	–	–	–	–	11.25%	0.19%	0.77	2.60E-01	–	–	–	–	11.36%	0.29%	0.77	1.59E-01
5.0E-03	–	–	–	–	–	–	–	–	–	–	–	–	–	–	–	–
5.0E-02	11.25%	0.19%	0.77	2.65E-01	–	–	–	–	–	–	–	–	11.57%	0.51%	0.77	6.48E-02
1.0E-01	–	–	–	–	11.18%	0.12%	0.77	3.70E-01	–	–	–	–	11.17%	0.11%	0.77	3.98E-01
2.0E-01	11.09%	0.03%	0.77	6.43E-01	11.06%	0.00%	0.77	9.70E-01	–	–	–	–	11.08%	0.02%	0.77	7.25E-01
3.0E-01	11.18%	0.12%	0.77	3.66E-01	–	–	–	–	–	–	–	–	–	–	–	–
4.0E-01	–	–	–	–	11.08%	0.02%	0.77	7.40E-01	–	–	–	–	–	–	–	–
5.0E-01	–	–	–	–	–	–	–	–	–	–	–	–	11.06%	0.00%	0.77	8.66E-01
6.0E-01	–	–	–	–	–	–	–	–	–	–	–	–	11.06%	0.00%	0.77	9.59E-01
7.0E-01	–	–	–	–	11.06%	0.00%	0.77	8.86E-01	–	–	–	–	–	–	–	–
8.0E-01	–	–	–	–	11.07%	0.01%	0.77	7.99E-01	–	–	–	–	–	–	–	–
9.0E-01	–	–	–	–	–	–	–	–	–	–	–	–	–	–	–	–
1.0E+00	–	–	–	–	–	–	–	–	–	–	–	–	–	–	–	–

B. By chromosome

	CD3 pruned-SNPs				CD4 pruned-SNPs				CD8 pruned-SNPs				CD4/CD8 pruned-SNPs			
CHR	Nagelkerke R^2 (%)	% explained variance	C-statistic	SCORE P	Nagelkerke R^2 (%)	% explained variance	C-statistic	SCORE P	Nagelkerke R^2 (%)	% explained variance	C-statistic	SCORE P	Nagelkerke R^2 (%)	% explained variance	C-statistic	SCORE P
Baseline	11.06%	0.00%	0.77	**1.55E-17**	11.06%	0.00%	0.77	**1.55E-17**	11.06%	0.00%	0.77	**1.55E-17**	11.06%	0.00%	0.77	**1.55E-17**
1	11.08%	0.02%	0.77	7.30E-01	11.06%	0.00%	0.77	9.29E-01	–	–	–	–	–	–	–	–
2	–	–	–	–	–	–	–	–	–	–	–	–	–	–	–	–
3	–	–	–	–	11.11%	0.05%	0.77	5.68E-01	11.07%	0.01%	0.77	7.63E-01	–	–	–	–
4	11.28%	0.22%	0.77	2.21E-01	11.13%	0.07%	0.77	5.04E-01	11.36%	0.30%	0.77	1.56E-01	–	–	–	–
5	–	–	–	–	11.06%	0.00%	0.77	9.61E-01	–	–	–	–	11.07%	0.01%	0.77	8.28E-01
6	–	–	–	–	–	–	–	–	–	–	–	–	11.24%	0.18%	0.77	2.66E-01
7	–	–	–	–	11.08%	0.02%	0.77	7.21E-01	–	–	–	–	11.06%	0.00%	0.77	8.98E-01
8	–	–	–	–	–	–	–	–	–	–	–	–	–	–	–	–
9	11.06%	0.00%	0.77	9.07E-01	–	–	–	–	–	–	–	–	–	–	–	–
10	11.07%	0.01%	0.77	8.48E-01	–	–	–	–	11.16%	0.10%	0.77	4.17E-01	11.35%	0.29%	0.77	1.67E-01
11	–	–	–	–	–	–	–	–	11.07%	0.01%	0.77	8.24E-01	–	–	–	–
12	11.10%	0.04%	0.77	6.20E-01	–	–	–	–	–	–	–	–	–	–	–	–
13	11.17%	0.11%	0.77	3.99E-01	–	–	–	–	–	–	–	–	11.19%	0.13%	0.77	3.59E-01
14	11.19%	0.13%	0.77	3.58E-01	–	–	–	–	–	–	–	–	11.07%	0.01%	0.77	8.36E-01
15	–	–	–	–	11.09%	0.03%	0.77	6.56E-01	–	–	–	–	–	–	–	–
16	–	–	–	–	–	–	–	–	11.12%	0.06%	0.77	5.17E-01	11.14%	0.08%	0.77	4.72E-01
17	–	–	–	–	–	–	–	–	–	–	–	–	11.39%	0.33%	0.77	1.35E-01
18	11.18%	0.12%	0.77	3.70E-01	–	–	–	–	–	–	–	–	11.12%	0.06%	0.77	5.35E-01
19	11.08%	0.02%	0.77	7.46E-01	–	–	–	–	–	–	–	–	12.21%	1.15%	0.77	**5.37E-03**
20	11.11%	0.05%	0.77	5.74E-01	–	–	–	–	–	–	–	–	11.14%	0.08%	0.77	4.52E-01
21	–	–	–	–	–	–	–	–	–	–	–	–	11.80%	0.74%	0.77	**2.58E-02**
22	11.24%	0.18%	0.77	2.69E-01	–	–	–	–	11.09%	0.03%	0.77	6.66E-01	11.16%	0.10%	0.77	4.14E-01

Supplementary Table S14. Pleiotropy assessment of genetic variants of LS (ref. 17) in T-lymphocytes (CD4 and CD8 relative counts) and CD4/CD8 ratio in both blood of the healthy (ref. 22) and BAL of LS cases (unpublished data)

							Fixed effects model (ref. 17)				Blood CD4 (in healthy)			BAL CD4 (in diseased)			Blood CD8 (in healthy)			BAL CD8 (in diseased)			Blood CD4/CD8 (in healthy)			BAL CD4/CD8 (in diseased)		
SNP	CHR	BP	CA/NCA	Gene Symbol	Gene Location	Num. Cohorts	OR	L95	U95	Meta-P	Beta	SE	P	Beta	SE	P	Beta	SE	P	Beta	SE	P	Beta	SE	P	Beta	SE	P
rs3130288	6	32203979	A/C	*CREBL1*	UTR	4	4.163	3.537	4.898	**4.30E-66**	-	-	-	-0.053	0.125	6.76E-01	-	-	-	-0.173	0.123	1.64E-01	-	-	-	0.013	0.075	8.61E-01
rs3129927	6	32441805	C/A	*C6orf10*	INTRON	2	4.036	3.362	4.845	**1.29E-50**	0.025	0.047	6.00E-01	-0.053	0.125	6.76E-01	0.177	0.047	**1.70E-04**	-0.173	0.123	1.64E-01	-0.171	0.047	**3.10E-04**	0.065	0.075	3.87E-01
rs9268219	6	32392086	C/A	*C6orf10*	INTRON	2	4.055	3.375	4.871	**1.51E-50**	0.058	0.049	2.40E-01	-0.022	0.126	8.60E-01	0.222	0.049	**5.40E-06**	-0.154	0.124	2.16E-01	-0.189	0.049	**1.10E-04**	0.081	0.075	2.79E-01
rs3129950	6	32466179	C/G	*C6orf10 | BTNL2*	INTERGENIC	2	4.062	3.380	4.882	**1.79E-50**	0.005	0.045	9.10E-01	-0.049	0.127	6.98E-01	0.150	0.044	**7.60E-04**	-0.173	0.124	1.67E-01	-0.158	0.045	**4.10E-04**	0.074	0.075	3.25E-01
rs2395149	6	32433540	A/G	*C6orf10*	INTRON	2	3.981	3.319	4.774	**3.55E-50**	0.034	0.048	4.70E-01	-0.053	0.125	6.76E-01	0.188	0.047	**7.00E-05**	-0.173	0.123	1.64E-01	-0.174	0.047	**2.50E-04**	0.065	0.075	3.87E-01
rs3135394	6	32516475	G/A	*HLA-DRA*	INTRON	2	3.921	3.274	4.696	**7.66E-50**	0.052	0.049	2.90E-01	-0.081	0.125	5.19E-01	0.213	0.049	**1.30E-05**	-0.181	0.122	1.45E-01	-0.186	0.049	**1.50E-04**	0.056	0.075	4.54E-01
rs1265757	6	32410360	A/G	*C6orf10*	INTRON	2	3.972	3.310	4.765	**8.02E-50**	-	-	-	-0.053	0.125	6.76E-01	-	-	-	-0.173	0.123	1.64E-01	-	-	-	0.065	0.075	3.87E-01
rs9268235	6	32398186	A/G	*C6orf10*	INTRON	2	3.971	3.309	4.764	**8.22E-50**	0.058	0.049	2.40E-01	-0.053	0.125	6.76E-01	0.222	0.049	**5.40E-06**	-0.173	0.123	1.64E-01	-0.189	0.049	**1.10E-04**	0.065	0.075	3.87E-01
rs7775397	6	32369230	C/A	*C6orf10*	CODING	2	3.940	3.284	4.728	**2.94E-49**	0.058	0.049	2.40E-01	-0.053	0.125	6.76E-01	0.222	0.049	**5.40E-06**	-0.173	0.123	1.64E-01	-0.189	0.049	**1.10E-04**	0.065	0.075	3.87E-01
rs9268177	6	32382860	A/C	*C6orf10*	INTRON	2	3.940	3.284	4.728	**2.98E-49**	0.058	0.049	2.40E-01	-0.053	0.125	6.76E-01	0.222	0.049	**5.40E-06**	-0.173	0.123	1.64E-01	-0.189	0.049	**1.10E-04**	0.065	0.075	3.87E-01
rs2187668	6	32713862	A/G	*HLA-DQA1*	INTRON	2	3.938	3.282	4.725	**3.29E-49**	0.046	0.047	3.30E-01	-0.056	0.125	6.56E-01	0.169	0.046	**2.50E-04**	-0.170	0.122	1.70E-01	-0.144	0.046	**2.00E-03**	0.047	0.074	5.26E-01
rs389884	6	32048876	G/A	*STK19*	INTRON	2	3.879	3.239	4.646	**4.28E-49**	0.050	0.049	3.00E-01	-0.053	0.125	6.76E-01	0.240	0.048	**6.50E-07**	-0.173	0.123	1.64E-01	-0.218	0.049	**7.40E-06**	0.015	0.075	8.46E-01
rs3132971	6	32338234	C/A	*NOTCH4 | C6orf10*	INTERGENIC	2	3.932	3.276	4.719	**6.55E-49**	-	-	-	-0.053	0.125	6.76E-01	-	-	-	-0.173	0.123	1.64E-01	-	-	-	0.034	0.076	6.53E-01
rs1794282	6	32774504	A/G	*HLA-DQB1 | HLA-DQA2*	INTERGENIC	2	3.916	3.263	4.700	**9.82E-49**	0.058	0.050	2.40E-01	-0.053	0.125	6.76E-01	0.212	0.049	**1.60E-05**	-0.173	0.123	1.64E-01	-0.180	0.049	**2.60E-04**	0.073	0.075	3.27E-01
rs1150753	6	32167845	G/A	*TNXB*	INTRON	2	3.895	3.247	4.672	**1.26E-48**	0.033	0.047	4.90E-01	-0.053	0.125	6.76E-01	0.231	0.047	**7.30E-07**	-0.173	0.123	1.64E-01	-0.224	0.047	**1.90E-06**	0.016	0.076	8.36E-01
rs3129716	6	32765414	G/A	*HLA-DQB1 | HLA-DQA2*	INTERGENIC	2	3.875	3.232	4.646	**1.77E-48**	-	-	-	-0.056	0.125	6.56E-01	-	-	-	-0.170	0.122	1.70E-01	-	-	-	0.051	0.074	4.93E-01
rs2854275	6	32736406	A/C	*HLA-DQB1*	INTRON	2	3.907	3.254	4.691	**2.39E-48**	-	-	-	-0.056	0.125	6.56E-01	-	-	-	-0.170	0.122	1.70E-01	-	-	-	0.048	0.075	5.18E-01
rs2856674	6	32767623	G/A	*HLA-DQB1 | HLA-DQA2*	INTERGENIC	2	3.884	3.237	4.660	**2.80E-48**	0.044	0.047	3.40E-01	-0.056	0.125	6.56E-01	0.170	0.046	**2.30E-04**	-0.170	0.122	1.70E-01	-0.146	0.046	**1.70E-03**	0.051	0.074	4.93E-01
rs3130490	6	31847099	A/C	*C6orf27*	INTRON	2	3.820	3.189	4.577	**6.92E-48**	-	-	-	-0.062	0.125	6.22E-01	-	-	-	-0.170	0.122	1.70E-01	-	-	-	0.010	0.075	8.89E-01
rs1270942	6	32026839	G/A	*CFB*	INTRON	2	3.792	3.168	4.539	**7.12E-48**	0.048	0.049	3.30E-01	-0.053	0.125	6.76E-01	0.242	0.048	**5.40E-07**	-0.173	0.123	1.64E-01	-0.221	0.049	**5.20E-06**	0.016	0.075	8.37E-01
rs3132449	6	31733992	A/G	*APOM | C6orf47*	INTERGENIC	2	3.760	3.144	4.497	**1.11E-47**	-	-	-	-0.062	0.125	6.22E-01	-	-	-	-0.170	0.122	1.70E-01	-	-	-	0.009	0.075	9.02E-01
rs519417	6	31986412	A/G	*ZBTB12 | C2*	INTERGENIC	2	3.777	3.155	4.520	**1.34E-47**	-	-	-	-0.053	0.125	6.76E-01	-	-	-	-0.173	0.123	1.64E-01	-	-	-	0.016	0.075	8.37E-01
rs497309	6	32000463	C/A	*ZBTB12 | C2*	INTERGENIC	2	3.780	3.157	4.526	**1.82E-47**	0.048	0.049	3.30E-01	-0.053	0.125	6.76E-01	0.242	0.048	**5.60E-07**	-0.173	0.123	1.64E-01	-0.221	0.049	**5.30E-06**	0.016	0.075	8.37E-01
rs3117577	6	31835453	G/A	*MSH5*	INTRON	2	3.757	3.140	4.495	**1.96E-47**	0.025	0.045	5.80E-01	-0.062	0.125	6.22E-01	0.213	0.045	**1.90E-06**	-0.170	0.122	1.70E-01	-0.204	0.045	**5.60E-06**	0.010	0.075	8.89E-01
rs3117582	6	31728499	C/A	*BAT3 | APOM*	INTERGENIC	2	3.731	3.120	4.460	**2.53E-47**	0.038	0.048	4.30E-01	-0.062	0.125	6.22E-01	0.264	0.047	**2.40E-08**	-0.170	0.122	1.70E-01	-0.246	0.048	**2.60E-07**	0.009	0.075	9.02E-01
rs558702	6	31978305	A/G	*ZBTB12 | C2*	INTERGENIC	2	3.747	3.131	4.485	**4.72E-47**	0.043	0.049	3.80E-01	-0.062	0.125	6.22E-01	0.248	0.048	**3.20E-07**	-0.170	0.122	1.70E-01	-0.233	0.049	**1.70E-06**	0.007	0.076	9.31E-01
rs3101018	6	31813843	A/G	*CLIC1 | MSH5*	INTERGENIC	2	3.725	3.115	4.455	**4.88E-47**	0.041	0.048	4.00E-01	-0.062	0.125	6.22E-01	0.257	0.048	**6.40E-08**	-0.170	0.122	1.70E-01	-0.246	0.048	**2.80E-07**	0.010	0.075	8.89E-01
rs3130679	6	31915519	G/A	*C6orf48*	UTR	2	3.742	3.126	4.478	**6.02E-47**	0.040	0.048	4.00E-01	-0.062	0.125	6.22E-01	0.256	0.048	**8.00E-08**	-0.170	0.122	1.70E-01	-0.245	0.048	**3.40E-07**	0.010	0.075	8.89E-01
rs3117574	6	31833209	A/G	*MSH5*	INTRON	2	3.723	3.112	4.455	**8.41E-47**	0.041	0.048	4.00E-01	-0.062	0.125	6.22E-01	0.257	0.048	**6.40E-08**	-0.170	0.122	1.70E-01	-0.246	0.048	**2.80E-07**	0.010	0.075	8.89E-01
rs3131378	6	31833264	G/A	*MSH5*	INTRON	2	3.740	3.123	4.478	**1.00E-46**	0.041	0.048	4.00E-01	-0.062	0.125	6.22E-01	0.257	0.048	**6.40E-08**	-0.170	0.122	1.70E-01	-0.246	0.048	**2.80E-07**	0.010	0.075	8.89E-01
rs9267531	6	31744721	G/A	*CSNK2B*	INTRON	2	3.729	3.115	4.463	**1.01E-46**	0.038	0.048	4.30E-01	-0.062	0.125	6.22E-01	0.264	0.047	**2.40E-08**	-0.170	0.122	1.70E-01	-0.246	0.048	**2.60E-07**	0.009	0.075	9.02E-01
rs3117575	6	31834232	G/A	*MSH5*	INTRON	2	3.719	3.108	4.449	**1.02E-46**	0.041	0.048	4.00E-01	-0.062	0.125	6.22E-01	0.257	0.048	**6.40E-08**	-0.170	0.122	1.70E-01	-0.246	0.048	**2.80E-07**	0.010	0.075	8.89E-01
rs3115671	6	31842324	A/C	*C6orf27*	CODING	2	3.718	3.107	4.448	**1.05E-46**	0.040	0.048	4.10E-01	-0.062	0.125	6.22E-01	0.254	0.048	**1.00E-07**	-0.170	0.122	1.70E-01	-0.243	0.048	**4.30E-07**	0.010	0.075	8.89E-01
rs3132450	6	31704117	G/A	*BAT2*	INTRON	2	3.738	3.122	4.475	**1.07E-46**	0.039	0.048	4.10E-01	-0.062	0.125	6.22E-01	0.270	0.047	**1.10E-08**	-0.170	0.122	1.70E-01	-0.245	0.048	**2.90E-07**	0.009	0.075	9.02E-01
rs3131383	6	31812273	A/C	*CLIC1*	UTR	2	3.717	3.106	4.447	**1.12E-46**	0.041	0.048	4.00E-01	-0.062	0.125	6.22E-01	0.257	0.048	**6.40E-08**	-0.170	0.122	1.70E-01	-0.246	0.048	**2.80E-07**	0.010	0.075	8.89E-01
rs3130484	6	31823861	G/A	*MSH5*	INTRON	2	3.715	3.105	4.445	**1.17E-46**	0.043	0.048	3.70E-01	-0.062	0.125	6.22E-01	0.259	0.047	**4.70E-08**	-0.170	0.122	1.70E-01	-0.246	0.048	**2.50E-07**	0.010	0.075	8.89E-01
rs915652	6	31857121	A/G	*VARS*	INTRON	2	3.739	3.122	4.479	**1.59E-46**	0.040	0.048	4.10E-01	-0.067	0.125	5.91E-01	0.256	0.048	**8.00E-08**	-0.167	0.123	1.78E-01	-0.245	0.048	**3.20E-07**	0.011	0.075	8.81E-01
rs3131379	6	31829012	A/G	*MSH5*	INTRON	2	3.716	3.104	4.447	**1.80E-46**	0.041	0.048	4.00E-01	-0.062	0.125	6.22E-01	0.257	0.048	**6.40E-08**	-0.170	0.122	1.70E-01	-0.246	0.048	**2.80E-07**	0.010	0.075	8.89E-01
rs3131618	6	31542600	G/A	*HCP5 | 3.8-1*	INTERGENIC	2	3.789	3.157	4.548	**2.14E-46**	-	-	-	0.021	0.125	8.66E-01	-	-	-	-0.136	0.123	2.74E-01	-	-	-	0.002	0.075	9.75E-01
rs3132510	6	31280130	G/A	*HCG27 | HLA-C*	INTERGENIC	2	3.796	3.160	4.560	**3.67E-46**	-	-	-	0.027	0.124	8.28E-01	-	-	-	-0.119	0.123	3.36E-01	-	-	-	0.022	0.075	7.68E-01
rs4143332	6	31456344	A/G	*LOC729816 | LOC100129668*	INTERGENIC	2	3.748	3.125	4.495	**5.06E-46**	0.028	0.046	5.40E-01	-0.005	0.124	9.67E-01	0.294	0.045	**6.90E-11**	-0.138	0.122	2.63E-01	-0.285	0.046	**3.90E-10**	-0.001	0.075	9.90E-01
rs3130985	6	31193335	A/G	*CDSN*	INTRON	2	3.673	3.069	4.395	**9.53E-46**	-	-	-	-0.034	0.124	7.83E-01	-	-	-	-0.160	0.122	1.95E-01	-	-	-	0.008	0.075	9.17E-01
rs3130544	6	31166319	A/C	*HCG22 | C6orf15*	INTERGENIC	2	3.655	3.056	4.373	**1.30E-45**	0.011	0.045	8.00E-01	-0.034	0.124	7.83E-01	0.277	0.044	**4.00E-10**	-0.160	0.122	1.95E-01	-0.284	0.045	**2.50E-10**	0.007	0.075	9.26E-01
rs3131619	6	31542310	A/T	*HCP5 | 3.8-1*	INTERGENIC	2	3.676	3.070	4.401	**1.41E-45**	-	-	-	-0.005	0.124	9.67E-01	-	-	-	-0.138	0.122	2.63E-01	-	-	-	0.002	0.075	9.81E-01
rs3094013	6	31542345	A/G	*HCP5 | 3.8-1*	INTERGENIC	2	3.688	3.078	4.419	**1.95E-45**	0.036	0.044	4.20E-01	-0.005	0.124	9.67E-01	0.277	0.043	**1.70E-10**	-0.138	0.122	2.63E-01	-0.259	0.044	**4.10E-09**	0.002	0.075	9.81E-01
rs9267488	6	31622226	G/A	*ATP6V1G2*	INTRON	2	3.624	3.031	4.333	**2.67E-45**	-	-	-	-0.044	0.124	7.21E-01	-	-	-	-0.157	0.122	2.01E-01	-	-	-	-0.003	0.075	9.67E-01
rs3094222	6	31189413	G/A	*C6orf15 | PSORS1C1*	INTERGENIC	2	3.648	3.048	4.368	**4.00E-45**	0.007	0.044	8.70E-01	-0.034	0.124	7.83E-01	0.270	0.043	**4.20E-10**	-0.160	0.122	1.95E-01	-0.280	0.044	**1.40E-10**	0.015	0.075	8.40E-01
rs3130557	6	31202682	A/G	*PSORS1C1*	INTRON	2	3.630	3.033	4.344	**5.87E-45**	0.011	0.045	8.00E-01	-0.034	0.124	7.83E-01	0.272	0.044	**6.70E-10**	-0.160	0.122	1.95E-01	-0.279	0.045	**3.90E-10**	-0.061	0.076	4.22E-01
rs3094605	6	31538673	G/C	*LOC100129668 | HCP5*	INTERGENIC	2	3.649	3.047	4.371	**6.57E-45**	-	-	-	-0.005	0.124	9.67E-01	-	-	-	-0.138	0.122	2.63E-01	-	-	-	0.002	0.075	9.81E-01
rs2001097	6	32491836	A/C	*BTNL2 | HLA-DRA*	INTERGENIC	2	3.170	2.699	3.724	**7.05E-45**	0.071	0.042	9.40E-02	-0.128	0.122	2.98E-01	0.068	0.042	1.08E-01	-0.279	0.117	**2.01E-02**	-0.013	0.042	7.60E-01	0.032	0.074	6.69E-01
rs3131788	6	31132775	A/G	*HCG22*	UTR	2	3.607	3.013	4.317	**2.00E-44**	0.011	0.045	8.00E-01	-0.034	0.124	7.83E-01	0.277	0.044	**4.00E-10**	-0.160	0.122	1.95E-01	-0.284	0.045	**2.50E-10**	0.007	0.075	9.26E-01
rs3135353	6	32500855	A/G	*BTNL2 | HLA-DRA*	INTERGENIC	2	3.293	2.785	3.894	**3.24E-44**	0.083	0.045	6.40E-02	-0.068	0.123	5.82E-01	0.089	0.044	**4.30E-02**	-0.219	0.120	7.20E-02	-0.028	0.045	5.30E-01	0.057	0.075	4.49E-01
rs3099844	6	31556955	A/C	*3.8-1 | MICB*	INTERGENIC	2	3.576	2.989	4.277	**3.27E-44**	0.044	0.045	3.40E-01	-0.054	0.124	6.63E-01	0.285	0.045	**1.90E-10**	-0.024	0.124	8.49E-01	-0.259	0.045	**1.20E-08**	-0.019	0.075	7.97E-01
rs2239805	6	32519354	C/A	*HLA-DRA*	INTRON	2	3.130	2.666	3.674	**3.39E-44**	0.064	0.042	1.29E-01	-0.128	0.122	2.98E-01	0.067	0.042	1.10E-01	-0.279	0.117	**2.01E-02**	-0.019	0.042	6.60E-01	0.032	0.074	6.69E-01
rs3135351	6	32500923	A/C	*BTNL2 | HLA-DRA*	INTERGENIC	2	3.143	2.675	3.692	**4.49E-44**	0.071	0.042	9.50E-02	-0.128	0.122	2.98E-01	0.069	0.042	9.80E-02	-0.279	0.117	**2.01E-02**	-0.015	0.042	7.20E-01	0.032	0.074	6.69E-01
rs3135356	6	32499494	A/G	*BTNL2 | HLA-DRA*	INTERGENIC	2	3.142	2.674	3.692	**4.64E-44**	-	-	-	-0.128	0.122	2.98E-01	-	-	-	-0.279	0.117	**2.01E-02**	-	-	-	0.032	0.074	6.69E-01
rs3135382	6	32491419	C/A	*BTNL2 | HLA-DRA*	INTERGENIC	2	3.163	2.689	3.721	**5.45E-44**	0.071	0.042	9.40E-02	-0.128	0.122	2.98E-01	0.068	0.042	1.08E-01	-0.279	0.117	**2.01E-02**	-0.013	0.042	7.60E-01	0.032	0.074	6.69E-01
rs6930933	6	32491388	A/C	*BTNL2 | HLA-DRA*	INTERGENIC	2	3.121	2.657	3.667	**1.25E-43**	0.071	0.042	9.40E-02	-0.128	0.122	2.98E-01	0.068	0.042	1.08E-01	-0.279	0.117	**2.01E-02**	-0.013	0.042	7.60E-01	0.032	0.074	6.67E-01
rs3135372	6	32493760	A/G	*BTNL2 | HLA-DRA*	INTERGENIC	2	3.129	2.663	3.678	**1.52E-43**	0.071	0.042	9.40E-02	-0.128	0.122	2.98E-01	0.068	0.042	1.08E-01	-0.279	0.117	**2.01E-02**	-0.013	0.042	7.60E-01	0.032	0.074	6.69E-01
rs2213580	6	32496552	G/A	*BTNL2 | HLA-DRA*	INTERGENIC	2	3.139	2.669	3.691	**1.77E-43**	0.071	0.042	9.40E-02	-0.128	0.122	2.98E-01	0.068	0.042	1.08E-01	-0.279	0.117	**2.01E-02**	-0.013	0.042	7.60E-01	0.032	0.074	6.69E-01
rs2395171	6	32502515	A/G	*BTNL2 | HLA-DRA*	INTERGENIC	2	3.126	2.660	3.675	**1.78E-43**	0.071	0.042	9.50E-02	-0.128	0.122	2.98E-01	0.069	0.042	9.80E-02	-0.279	0.117	**2.01E-02**	-0.015	0.042	7.20E-01	0.032	0.074	6.69E-01
rs3135374	6	32493583	A/C	*BTNL2 | HLA-DRA*	INTERGENIC	2	3.117	2.652	3.663	**2.76E-43**	0.071	0.042	9.40E-02	-0.128	0.122	2.98E-01	0.068	0.042	1.08E-01	-0.279	0.117	**2.01E-02**	-0.013	0.042	7.60E-01	0.032	0.074	6.69E-01
rs9268530	6	32491201	G/A	*BTNL2 | HLA-DRA*	INTERGENIC	2	3.123	2.656	3.673	**3.50E-43**	0.071	0.042	9.40E-02	-0.128	0.122	2.98E-01	0.068	0.042	1.08E-01	-0.279	0.117	**2.01E-02**	-0.013	0.042	7.60E-01	0.032	0.074	6.69E-01

rs2395162	6	32495758	A/C	*BTNL2 | HLA-DRA*	INTERGENIC	2	3.123	2.656	3.672	**3.57E-43**	0.071	0.042	9.40E-02	-0.128	0.122	2.98E-01	0.068	0.042	1.08E-01	-0.279	0.117	**2.01E-02**	-0.013	0.042	7.60E-01	0.032	0.074	6.69E-01
rs2187820	6	32493851	A/G	*BTNL2 | HLA-DRA*	INTERGENIC	2	3.122	2.655	3.671	**3.71E-43**	0.071	0.042	9.40E-02	-0.128	0.122	2.98E-01	0.068	0.042	1.08E-01	-0.279	0.117	**2.01E-02**	-0.013	0.042	7.60E-01	0.032	0.074	6.69E-01
rs2395164	6	32495838	A/G	*BTNL2 | HLA-DRA*	INTERGENIC	2	3.122	2.655	3.671	**3.71E-43**	0.071	0.042	9.40E-02	-0.128	0.122	2.98E-01	0.068	0.042	1.08E-01	-0.279	0.117	**2.01E-02**	-0.013	0.042	7.60E-01	0.032	0.074	6.69E-01
rs3135376	6	32493448	G/A	*BTNL2 | HLA-DRA*	INTERGENIC	2	3.122	2.655	3.671	**3.71E-43**	0.071	0.042	9.40E-02	-0.128	0.122	2.98E-01	0.068	0.042	1.08E-01	-0.279	0.117	**2.01E-02**	-0.013	0.042	7.60E-01	0.032	0.074	6.69E-01
rs3135378	6	32493077	A/G	*BTNL2 | HLA-DRA*	INTERGENIC	2	3.122	2.655	3.671	**3.71E-43**	0.071	0.042	9.40E-02	-0.128	0.122	2.98E-01	0.068	0.042	1.08E-01	-0.279	0.117	**2.01E-02**	-0.013	0.042	7.60E-01	0.032	0.074	6.69E-01
rs3135393	6	32516820	G/A	*HLA-DRA*	INTRON	2	3.107	2.643	3.652	**5.63E-43**	0.064	0.042	1.29E-01	-0.128	0.122	2.98E-01	0.067	0.042	1.10E-01	-0.279	0.117	**2.01E-02**	-0.019	0.042	6.60E-01	0.032	0.074	6.69E-01
rs2239806	6	32519285	A/G	*HLA-DRA*	INTRON	2	3.090	2.630	3.630	**7.18E-43**	0.064	0.042	1.29E-01	-0.128	0.122	2.98E-01	0.067	0.042	1.10E-01	-0.279	0.117	**2.01E-02**	-0.019	0.042	6.60E-01	0.032	0.074	6.69E-01
rs1051336	6	32520570	A/G	*HLA-DRA*	UTR	2	3.089	2.630	3.629	**7.53E-43**	0.064	0.042	1.29E-01	-0.128	0.122	2.98E-01	0.067	0.042	1.10E-01	-0.279	0.117	**2.01E-02**	-0.019	0.042	6.60E-01	0.032	0.074	6.69E-01
rs1041885	6	32520787	A/T	*HLA-DRA*	UTR	2	3.089	2.629	3.629	**7.56E-43**	0.064	0.042	1.29E-01	-0.128	0.122	2.98E-01	0.067	0.042	1.10E-01	-0.279	0.117	**2.01E-02**	-0.019	0.042	6.60E-01	0.032	0.074	6.69E-01
rs3134796	6	32297899	G/A	*NOTCH4*	INTRON	2	3.512	2.935	4.202	**8.09E-43**	-	-	-	-0.073	0.125	5.62E-01	-	-	-	-0.184	0.122	1.36E-01	-	-	-	0.034	0.075	6.47E-01
rs3131296	6	32280971	A/G	*NOTCH4*	INTRON	2	3.494	2.921	4.178	**9.61E-43**	0.052	0.046	2.60E-01	-0.073	0.125	5.62E-01	0.189	0.045	**3.20E-05**	-0.184	0.122	1.36E-01	-0.154	0.046	**7.60E-04**	0.035	0.075	6.39E-01
rs3132956	6	32287416	A/G	*NOTCH4*	INTRON	2	3.489	2.918	4.172	**9.77E-43**	0.052	0.046	2.60E-01	-0.073	0.125	5.62E-01	0.189	0.045	**3.20E-05**	-0.184	0.122	1.36E-01	-0.154	0.046	**7.60E-04**	0.034	0.075	6.47E-01
rs9268534	6	32491285	C/A	*BTNL2 | HLA-DRA*	INTERGENIC	2	3.124	2.654	3.677	**1.03E-42**	0.071	0.042	9.40E-02	-0.128	0.122	2.98E-01	0.068	0.042	1.08E-01	-0.279	0.117	**2.01E-02**	-0.013	0.042	7.60E-01	0.032	0.074	6.69E-01
rs3134942	6	32276749	A/C	*NOTCH4*	CODING	2	3.482	2.911	4.165	**1.99E-42**	0.050	0.046	2.70E-01	-0.073	0.125	5.62E-01	0.186	0.045	**4.20E-05**	-0.184	0.122	1.36E-01	-0.152	0.046	**8.90E-04**	0.034	0.075	6.47E-01
rs2395161	6	32495730	C/A	*BTNL2 | HLA-DRA*	INTERGENIC	2	3.142	2.665	3.704	**2.25E-42**	0.071	0.042	9.40E-02	-0.111	0.125	3.79E-01	0.068	0.042	1.08E-01	-0.278	0.121	**2.42E-02**	-0.013	0.042	7.60E-01	0.020	0.076	7.94E-01
rs3129887	6	32518669	A/G	*HLA-DRA*	INTRON	2	3.045	2.594	3.575	**3.58E-42**	0.064	0.042	1.29E-01	-0.128	0.122	2.98E-01	0.067	0.042	1.10E-01	-0.279	0.117	**2.01E-02**	-0.019	0.042	6.60E-01	0.025	0.074	7.39E-01
rs2071278	6	32273422	G/A	*NOTCH4*	INTRON	2	3.377	2.833	4.026	**5.49E-42**	0.050	0.045	2.60E-01	-0.117	0.124	3.49E-01	0.137	0.044	**1.90E-03**	-0.063	0.124	6.13E-01	-0.101	0.045	**2.30E-02**	0.014	0.075	8.54E-01
rs2395158	6	32482573	G/A	*BTNL2*	INTRON	2	3.182	2.691	3.764	**1.29E-41**	-	-	-	0.008	0.123	9.47E-01	-	-	-	-0.139	0.121	2.56E-01	-	-	-	0.049	0.075	5.10E-01
rs3130303	6	32313845	G/A	*NOTCH4 | C6orf10*	INTERGENIC	2	3.386	2.837	4.041	**1.31E-41**	-	-	-	-0.080	0.124	5.22E-01	-	-	-	-0.189	0.121	1.24E-01	-	-	-	0.029	0.075	7.01E-01
rs3129891	6	32523058	A/G	*HLA-DRA | HLA-DRB5*	INTERGENIC	2	2.984	2.546	3.496	**1.42E-41**	-	-	-	-0.041	0.123	7.37E-01	-	-	-	-0.217	0.119	7.28E-02	-	-	-	0.032	0.074	6.70E-01
rs6901158	6	32313920	A/G	*NOTCH4 | C6orf10*	INTERGENIC	2	3.370	2.824	4.023	**2.63E-41**	0.061	0.045	1.70E-01	-0.080	0.124	5.22E-01	0.145	0.044	**1.02E-03**	-0.189	0.121	1.24E-01	-0.097	0.044	**2.90E-02**	0.029	0.075	7.01E-01
rs6908065	6	32491319	C/A	*BTNL2 | HLA-DRA*	INTERGENIC	2	3.049	2.591	3.587	**4.26E-41**	0.073	0.042	8.40E-02	-0.143	0.126	2.59E-01	0.058	0.042	1.60E-01	-0.288	0.121	**2.01E-02**	0.000	0.042	1.00E+00	0.039	0.075	6.03E-01
rs9266669	6	31456056	A/G	*LOC729816 | LOC100129668*	INTERGENIC	2	3.348	2.805	3.996	**7.17E-41**	0.016	0.042	7.10E-01	0.088	0.123	4.76E-01	0.235	0.042	**1.60E-08**	-0.138	0.121	2.61E-01	-0.237	0.042	**1.90E-08**	0.036	0.075	6.33E-01
rs2233974	6	31187995	C/G	*C6orf15*	CODING	2	3.312	2.779	3.948	**9.45E-41**	-0.038	0.040	3.50E-01	-0.067	0.123	5.88E-01	0.172	0.040	**1.60E-05**	-0.086	0.122	4.82E-01	-0.219	0.040	**5.50E-08**	-0.015	0.075	8.47E-01
rs3131643	6	31550761	A/G	*3.8-1 | MICB*	INTERGENIC	2	3.176	2.679	3.764	**1.80E-40**	-	-	-	-0.132	0.124	2.90E-01	-	-	-	-0.215	0.121	7.96E-02	-	-	-	0.004	0.075	9.60E-01
rs1264341	6	30910444	G/A	*IER3 | DDR1*	INTERGENIC	2	3.431	2.858	4.118	**6.22E-40**	-0.010	0.045	8.20E-01	-0.017	0.125	8.95E-01	0.259	0.045	**8.20E-09**	-0.152	0.123	2.22E-01	-0.285	0.045	**3.80E-10**	-0.004	0.075	9.60E-01
rs2535332	6	30921228	A/G	*IER3 | DDR1*	INTERGENIC	2	3.435	2.860	4.125	**7.42E-40**	-0.010	0.045	8.20E-01	-0.017	0.125	8.95E-01	0.259	0.045	**8.20E-09**	-0.152	0.123	2.22E-01	-0.285	0.045	**3.80E-10**	-0.004	0.075	9.60E-01
rs693906	6	31943143	C/G	*SLC44A4*	INTRON	2	3.272	2.742	3.904	**1.75E-39**	-	-	-	-0.081	0.124	5.16E-01	-	-	-	-0.181	0.122	1.41E-01	-	-	-	0.029	0.075	7.01E-01
rs886420	6	30987615	A/G	*GTF2H4*	INTRON	2	3.411	2.840	4.096	**2.13E-39**	-	-	-	-0.043	0.125	7.32E-01	-	-	-	-0.155	0.123	2.09E-01	-	-	-	-0.004	0.075	9.59E-01
rs2535340	6	30946476	G/A	*IER3 | DDR1*	INTERGENIC	2	3.409	2.838	4.094	**2.39E-39**	-	-	-	-0.017	0.125	8.95E-01	-	-	-	-0.152	0.123	2.22E-01	-	-	-	-0.004	0.075	9.60E-01
rs9262263	6	30941936	G/A	*IER3 | DDR1*	INTERGENIC	2	3.409	2.838	4.094	**2.39E-39**	-	-	-	-0.017	0.125	8.95E-01	-	-	-	-0.152	0.123	2.22E-01	-	-	-	-0.004	0.075	9.60E-01
rs1264304	6	30990394	A/G	*VARS2*	UTR	2	3.403	2.830	4.092	**9.14E-39**	-	-	-	-0.043	0.125	7.32E-01	-	-	-	-0.155	0.123	2.09E-01	-	-	-	-0.004	0.075	9.59E-01
rs1264308	6	30987966	A/G	*GTF2H4*	INTRON	2	3.403	2.830	4.092	**9.14E-39**	-0.002	0.044	9.70E-01	-0.043	0.125	7.32E-01	0.213	0.043	**7.90E-07**	-0.155	0.123	2.09E-01	-0.226	0.044	**2.10E-07**	-0.004	0.075	9.59E-01
rs886422	6	30972258	A/G	*DDR1*	INTRON	2	3.403	2.830	4.092	**9.34E-39**	-0.002	0.044	9.70E-01	-0.043	0.125	7.32E-01	0.213	0.043	**7.90E-07**	-0.155	0.123	2.09E-01	-0.226	0.044	**2.10E-07**	-0.004	0.075	9.59E-01
rs1264373	6	30877252	A/G	*IER3 | DDR1*	INTERGENIC	2	3.364	2.802	4.040	**1.26E-38**	-	-	-	-0.001	0.125	9.91E-01	-	-	-	-0.135	0.123	2.76E-01	-	-	-	0.010	0.074	8.90E-01
rs1150755	6	32146528	A/G	*TNXB*	INTRON	2	3.043	2.570	3.601	**2.84E-38**	0.058	0.042	1.80E-01	0.029	0.124	8.16E-01	0.210	0.042	**6.20E-07**	-0.149	0.122	2.27E-01	-0.181	0.042	**1.80E-05**	0.010	0.075	8.93E-01
rs1150758	6	32136127	C/G	*TNXB*	INTRON	2	3.015	2.548	3.569	**1.02E-37**	0.057	0.042	1.80E-01	0.029	0.124	8.16E-01	0.213	0.042	**4.00E-07**	-0.149	0.122	2.27E-01	-0.185	0.042	**1.10E-05**	0.010	0.075	8.93E-01
rs2524067	6	31353800	G/A	*HLA-C | HLA-B*	INTERGENIC	2	3.060	2.580	3.631	**1.14E-37**	0.016	0.042	7.00E-01	0.017	0.124	8.91E-01	0.278	0.042	**2.50E-11**	-0.109	0.123	3.78E-01	-0.276	0.042	**5.20E-11**	-0.013	0.075	8.61E-01
rs3095328	6	30831760	A/G	*IER3 | DDR1*	INTERGENIC	2	3.342	2.779	4.019	**1.30E-37**	-	-	-	0.016	0.124	8.96E-01	-	-	-	-0.152	0.122	2.16E-01	-	-	-	0.025	0.074	7.34E-01
rs2524069	6	31352768	T/A	*HLA-C | HLA-B*	INTERGENIC	2	3.064	2.581	3.638	**1.83E-37**	0.016	0.042	7.00E-01	0.018	0.124	8.82E-01	0.278	0.042	**2.50E-11**	-0.135	0.123	2.74E-01	-0.276	0.042	**5.20E-11**	0.001	0.075	9.86E-01
rs2596565	6	31461308	A/G	*LOC729816 | LOC100129668*	INTERGENIC	1	3.870	3.145	4.762	**2.01E-37**	0.031	0.045	5.00E-01	-0.005	0.124	9.67E-01	0.296	0.045	**4.40E-11**	-0.138	0.122	2.63E-01	-0.286	0.046	**3.40E-10**	-0.001	0.075	9.90E-01
rs3117103	6	32457535	A/T	*C6orf10 | BTNL2*	INTERGENIC	1	3.800	3.094	4.667	**3.65E-37**	0.005	0.045	9.10E-01	0.052	0.127	6.82E-01	0.150	0.044	**7.60E-04**	-0.075	0.126	5.53E-01	-0.158	0.045	**4.10E-04**	0.065	0.075	3.88E-01
rs1265754	6	32411670	A/T	*C6orf10*	CODING	1	3.970	3.210	4.910	**4.76E-37**	0.058	0.049	2.40E-01	-0.053	0.125	6.76E-01	0.222	0.049	**5.40E-06**	-0.173	0.123	1.64E-01	-0.189	0.049	**1.10E-04**	0.065	0.075	3.91E-01
rs3095089	6	31041773	A/C	*LOC100129065 | C6orf205*	INTERGENIC	2	2.899	2.461	3.416	**4.87E-37**	-0.017	0.041	6.80E-01	-0.083	0.125	5.08E-01	0.165	0.041	**4.50E-05**	-0.137	0.123	2.71E-01	-0.194	0.041	**2.10E-06**	-0.008	0.075	9.20E-01
rs1150754	6	32158736	A/G	*TNXB*	INTRON	2	3.001	2.533	3.555	**5.12E-37**	0.052	0.042	2.10E-01	0.029	0.124	8.16E-01	0.206	0.041	**5.90E-07**	-0.149	0.122	2.27E-01	-0.182	0.042	**1.10E-05**	0.010	0.075	8.93E-01
rs1264361	6	30885477	G/A	*IER3 | DDR1*	INTERGENIC	2	3.233	2.697	3.875	**6.13E-37**	-	-	-	-0.022	0.124	8.62E-01	-	-	-	-0.137	0.122	2.65E-01	-	-	-	-0.004	0.074	9.62E-01
rs886425	6	30889280	G/A	*IER3 | DDR1*	INTERGENIC	2	3.233	2.697	3.875	**6.13E-37**	-	-	-	-0.022	0.124	8.62E-01	-	-	-	-0.137	0.122	2.65E-01	-	-	-	-0.004	0.074	9.62E-01
rs886424	6	30889981	A/G	*IER3 | DDR1*	INTERGENIC	2	3.241	2.703	3.886	**6.31E-37**	0.002	0.044	9.70E-01	-0.022	0.124	8.62E-01	0.238	0.044	**5.20E-08**	-0.137	0.122	2.65E-01	-0.249	0.044	**1.60E-08**	-0.004	0.074	9.62E-01
rs3132579	6	31048968	G/A	*LOC100129065 | C6orf205*	INTERGENIC	2	2.932	2.483	3.462	**7.08E-37**	0.008	0.041	8.40E-01	-0.074	0.124	5.52E-01	0.175	0.041	**1.80E-05**	-0.108	0.123	3.83E-01	-0.182	0.041	**9.80E-06**	0.004	0.075	9.52E-01
rs1269852	6	32188169	C/G	*TNXB | CREBL1*	INTERGENIC	1	3.830	3.112	4.713	**7.40E-37**	0.049	0.047	3.00E-01	-0.053	0.125	6.76E-01	0.234	0.046	**4.60E-07**	-0.173	0.123	1.64E-01	-0.212	0.047	**5.70E-06**	0.012	0.075	8.73E-01
rs3129981	6	30866836	A/G	*IER3 | DDR1*	INTERGENIC	2	3.179	2.659	3.802	**8.49E-37**	-	-	-	-0.026	0.124	8.34E-01	-	-	-	-0.151	0.122	2.17E-01	-	-	-	-0.004	0.074	9.59E-01
rs7750641	6	31237289	A/G	*TCF19*	CODING	1	3.820	3.104	4.701	**1.01E-36**	0.015	0.045	7.40E-01	-0.034	0.124	7.83E-01	0.278	0.044	**3.00E-10**	-0.160	0.122	1.95E-01	-0.282	0.045	**2.70E-10**	0.008	0.075	9.16E-01
rs3095326	6	30833820	A/G	*IER3 | DDR1*	INTERGENIC	2	3.268	2.720	3.927	**1.14E-36**	0.025	0.044	5.70E-01	0.016	0.124	8.96E-01	0.231	0.044	**1.10E-07**	-0.152	0.122	2.16E-01	-0.220	0.044	**5.80E-07**	0.025	0.074	7.34E-01
rs1264353	6	30895741	A/C	*IER3 | DDR1*	INTERGENIC	2	3.225	2.690	3.868	**1.25E-36**	0.002	0.044	9.70E-01	-0.022	0.124	8.62E-01	0.238	0.044	**5.20E-08**	-0.137	0.122	2.65E-01	-0.249	0.044	**1.60E-08**	-0.004	0.074	9.62E-01
rs9268127	6	32361537	G/A	*NOTCH4 | C6orf10*	INTERGENIC	2	2.917	2.470	3.444	**1.40E-36**	0.135	0.070	5.30E-02	-0.104	0.122	3.99E-01	-0.055	0.070	4.30E-01	-0.219	0.119	7.13E-02	0.167	0.070	**1.70E-02**	0.035	0.074	6.34E-01
rs599707	6	31916415	A/G	*C6orf48 | NEU1*	INTERGENIC	1	3.750	3.054	4.605	**1.88E-36**	0.040	0.048	4.00E-01	-0.062	0.125	6.22E-01	0.256	0.048	**8.00E-08**	-0.170	0.122	1.70E-01	-0.245	0.048	**3.40E-07**	0.010	0.075	8.94E-01
rs3129933	6	32444139	A/G	*C6orf10*	INTRON	2	2.918	2.471	3.447	**1.99E-36**	0.081	0.040	**4.60E-02**	-0.104	0.122	3.99E-01	0.102	0.040	**1.05E-02**	-0.219	0.119	7.13E-02	-0.043	0.040	2.90E-01	0.035	0.074	6.34E-01
rs3129925	6	32441397	A/G	*C6orf10*	INTRON	2	2.873	2.438	3.385	**2.07E-36**	-	-	-	-0.104	0.122	3.99E-01	-	-	-	-0.219	0.119	7.13E-02	-	-	-	0.035	0.074	6.34E-01
rs3117137	6	32417889	A/G	*C6orf10*	INTRON	2	2.857	2.427	3.364	**2.11E-36**	-	-	-	-0.080	0.123	5.15E-01	-	-	-	-0.167	0.121	1.70E-01	-	-	-	0.001	0.074	9.90E-01
rs1003878	6	32407800	A/G	*C6orf10*	CODING	2	2.857	2.426	3.363	**2.14E-36**	0.068	0.038	7.60E-02	-0.080	0.123	5.15E-01	0.069	0.038	6.90E-02	-0.167	0.121	1.70E-01	-0.011	0.038	7.80E-01	0.001	0.074	9.90E-01
rs3129949	6	32406792	A/C	*C6orf10*	INTRON	2	2.857	2.426	3.363	**2.14E-36**	0.063	0.038	9.70E-02	-0.080	0.123	5.15E-01	0.073	0.038	5.30E-02	-0.167	0.121	1.70E-01	-0.020	0.038	6.00E-01	0.001	0.074	9.90E-01
rs3129939	6	32444744	G/A	*C6orf10*	INTRON	2	2.872	2.437	3.384	**2.26E-36**	0.101	0.041	**1.45E-02**	-0.104	0.122	3.99E-01	0.111	0.041	**6.90E-03**	-0.219	0.119	7.13E-02	-0.033	0.041	4.30E-01	0.035	0.074	6.34E-01
rs3129973	6	30829122	A/G	*IER3 | DDR1*	INTERGENIC	2	3.259	2.712	3.918	**2.45E-36**	0.026	0.045	5.70E-01	0.016	0.124	8.96E-01	0.251	0.044	**1.60E-08**	-0.152	0.122	2.16E-01	-0.242	0.045	**7.10E-08**	0.025	0.074	7.34E-01
rs204989	6	32269830	A/G	*GPSM3*	INTRON	2	2.966	2.504	3.514	**2.76E-36**	0.094	0.040	**1.90E-02**	-0.042	0.124	7.37E-01	0.080	0.040	**4.20E-02**	0.040	0.123	7.44E-01	-0.005	0.040	9.00E-01	-0.002	0.075	9.83E-01
rs6910668	6	32371436	C/A	*C6orf10*	INTRON	2	2.901	2.457	3.425	**2.84E-36**	0.080	0.040	**4.40E-02**	-0.104	0.122	3.99E-01	0.115	0.040	**3.50E-03**	-0.219	0.119	7.13E-02	-0.057	0.040	1.50E-01	0.035	0.074	6.34E-01
rs7745174	6	32374773	A/G	*C6orf10*	INTRON	2	2.901	2.457	3.425	**2.84E-36**	-	-	-	-0.104	0.122	3.99E-01	-	-	-	-0.219	0.119	7.13E-02	-	-	-	0.035	0.074	6.34E-01
rs204991	6	32269344	G/A	*GPSM3*	INTRON	2	2.971	2.507	3.521	**3.40E-36**	0.094	0.040	**1.90E-02**	-0.042	0.124	7.37E-01	0.080	0.040	**4.20E-02**	0.040	0.123	7.44E-01	-0.005	0.040	9.00E-01	-0.002	0.075	9.83E-01
rs3093988	6	31600432	A/G	*MICB | MCCD1*	INTERGENIC	2	2.948	2.490	3.491	**3.80E-36**	0.004	0.041	9.30E-01	-0.047	0.123	7.03E-01	0.150	0.040	**1.80E-04**	-0.195	0.120	1.08E-01	-0.152	0.041	**1.90E-04**	0.006	0.074	9.34E-01
rs3128987	6	31542177	G/A	*HCP5 | 3.8-1*	INTERGENIC	2	2.964	2.502	3.513	**4.16E-36**	-	-	-	0.057	0.123	6.43E-01	-	-	-	-0.056	0.122	6.49E-01	-	-	-	-0.034	0.075	6.50E-01

rs3130070	6	31699787	G/A	*BAT2*	INTRON	2	2.899	2.455	3.424	**4.33E-36**	-	-	-	-0.192	0.122	1.20E-01	-	-	-	-0.275	0.119	**2.36E-02**	-	-	-	0.036	0.075	6.35E-01
rs11229	6	31711749	G/A	*BAT2*	CODING	2	2.899	2.455	3.424	**4.35E-36**	-	-	-	-0.192	0.122	1.20E-01	-	-	-	-0.275	0.119	**2.36E-02**	-	-	-	0.036	0.075	6.35E-01
rs1033499	6	32415510	A/G	*C6orf10*	INTRON	2	2.911	2.463	3.440	**4.59E-36**	-	-	-	-0.104	0.122	3.99E-01	-	-	-	-0.219	0.119	7.13E-02	-	-	-	0.035	0.074	6.34E-01
rs2143461	6	32443325	A/G	*C6orf10*	INTRON	2	2.874	2.437	3.391	**5.21E-36**	-	-	-	-0.104	0.122	3.99E-01	-	-	-	-0.219	0.119	7.13E-02	-	-	-	0.035	0.074	6.34E-01
rs3130622	6	31700503	C/G	*BAT2*	INTRON	2	2.920	2.470	3.454	**5.38E-36**	-	-	-	-0.192	0.122	1.20E-01	-	-	-	-0.275	0.119	**2.36E-02**	-	-	-	0.036	0.075	6.35E-01
rs204990	6	32269408	A/C	*GPSM3*	INTRON	2	2.958	2.496	3.506	**5.89E-36**	0.094	0.040	**1.90E-02**	-0.042	0.124	7.37E-01	0.080	0.040	**4.20E-02**	0.040	0.123	7.44E-01	-0.005	0.040	9.00E-01	-0.002	0.075	9.83E-01
rs3115665	6	31697243	C/G	*BAT2*	INTRON	2	2.907	2.460	3.437	**7.13E-36**	-	-	-	-0.192	0.122	1.20E-01	-	-	-	-0.275	0.119	**2.36E-02**	-	-	-	0.036	0.075	6.35E-01
rs3117583	6	31727555	G/A	*BAT3*	INTRON	2	2.906	2.458	3.435	**7.69E-36**	0.072	0.041	7.80E-02	-0.192	0.122	1.20E-01	0.119	0.041	**3.50E-03**	-0.275	0.119	**2.36E-02**	-0.059	0.041	1.48E-01	0.036	0.075	6.35E-01
rs3129926	6	32441458	G/A	*C6orf10*	INTRON	2	2.874	2.436	3.392	**8.13E-36**	-	-	-	-0.104	0.122	3.99E-01	-	-	-	-0.219	0.119	7.13E-02	-	-	-	0.035	0.074	6.34E-01
rs3130628	6	31717251	G/A	*BAT3*	INTRON	2	2.905	2.457	3.434	**8.20E-36**	0.074	0.041	7.30E-02	-0.192	0.122	1.20E-01	0.119	0.041	**3.30E-03**	-0.275	0.119	**2.36E-02**	-0.059	0.041	1.50E-01	0.036	0.075	6.35E-01
rs3129924	6	32441277	A/G	*C6orf10*	INTRON	2	2.874	2.435	3.392	**8.21E-36**	-	-	-	-0.104	0.122	3.99E-01	-	-	-	-0.219	0.119	7.13E-02	-	-	-	0.035	0.074	6.34E-01
rs3094228	6	31537906	G/A	*LOC100129668 | HCP5*	INTERGENIC	2	2.923	2.470	3.459	**8.60E-36**	-0.007	0.039	8.50E-01	0.024	0.123	8.48E-01	0.273	0.039	**1.90E-12**	-0.070	0.122	5.66E-01	-0.286	0.039	**3.10E-13**	-0.042	0.074	5.76E-01
rs1055384	6	31609537	A/G	*BAT1*	INTRON	2	2.940	2.483	3.483	**8.80E-36**	-	-	-	-0.047	0.123	7.03E-01	-	-	-	-0.195	0.120	1.08E-01	-	-	-	0.007	0.075	9.26E-01
rs3130626	6	31706468	G/A	*BAT2*	CODING	2	2.912	2.462	3.444	**9.20E-36**	0.073	0.041	7.40E-02	-0.203	0.121	9.83E-02	0.123	0.041	**2.40E-03**	-0.275	0.118	**2.32E-02**	-0.059	0.041	1.50E-01	0.033	0.075	6.65E-01
rs1480380	6	33021224	A/G	*HLA-DMB | HLA-DMA*	INTERGENIC	2	3.436	2.831	4.171	**9.24E-36**	0.029	0.054	6.00E-01	-0.065	0.123	5.99E-01	0.118	0.053	**2.80E-02**	-0.041	0.123	7.42E-01	-0.088	0.054	1.03E-01	0.008	0.074	9.18E-01
rs3099840	6	31538700	G/A	*LOC100129668 | HCP5*	INTERGENIC	2	2.938	2.480	3.480	**1.07E-35**	0.031	0.041	4.50E-01	0.024	0.123	8.48E-01	0.280	0.041	**5.00E-12**	-0.070	0.122	5.66E-01	-0.268	0.041	**6.10E-11**	-0.042	0.074	5.76E-01
rs3130673	6	30854498	A/C	*IER3 | DDR1*	INTERGENIC	2	3.170	2.644	3.801	**1.17E-35**	0.023	0.044	6.00E-01	-0.004	0.124	9.76E-01	0.237	0.044	**6.60E-08**	-0.155	0.122	2.07E-01	-0.229	0.044	**2.30E-07**	0.011	0.074	8.80E-01
rs3130641	6	30872060	A/G	*IER3 | DDR1*	INTERGENIC	2	3.169	2.643	3.799	**1.26E-35**	0.018	0.044	6.80E-01	-0.004	0.124	9.76E-01	0.243	0.044	**2.70E-08**	-0.155	0.122	2.07E-01	-0.241	0.044	**5.10E-08**	0.011	0.074	8.80E-01
rs3131050	6	30868004	G/A	*IER3 | DDR1*	INTERGENIC	2	3.169	2.643	3.799	**1.26E-35**	0.020	0.044	6.60E-01	-0.004	0.124	9.76E-01	0.241	0.044	**3.80E-08**	-0.155	0.122	2.07E-01	-0.237	0.044	**8.70E-08**	0.011	0.074	8.80E-01
rs3131060	6	30871270	A/G	*IER3 | DDR1*	INTERGENIC	2	3.169	2.643	3.799	**1.26E-35**	0.018	0.044	6.80E-01	-0.004	0.124	9.76E-01	0.243	0.044	**2.70E-08**	-0.155	0.122	2.07E-01	-0.241	0.044	**5.10E-08**	0.011	0.074	8.80E-01
rs377763	6	32307122	A/C	*NOTCH4 | C6orf10*	INTERGENIC	2	2.809	2.387	3.305	**1.47E-35**	0.075	0.042	7.30E-02	-0.009	0.123	9.40E-01	0.078	0.041	5.80E-02	-0.175	0.120	1.51E-01	-0.012	0.042	7.70E-01	0.005	0.074	9.48E-01
rs2517546	6	31117034	A/G	*LOC729792*	INTRON	2	3.020	2.537	3.594	**1.70E-35**	0.018	0.043	6.80E-01	-0.046	0.124	7.15E-01	0.230	0.042	**4.40E-08**	-0.181	0.122	1.43E-01	-0.226	0.043	**1.10E-07**	-0.003	0.075	9.72E-01
rs3130627	6	31708830	A/C	*BAT2*	INTRON	2	2.898	2.451	3.428	**1.72E-35**	-	-	-	-0.192	0.122	1.20E-01	-	-	-	-0.275	0.119	**2.36E-02**	-	-	-	0.036	0.075	6.35E-01
rs2736157	6	31708799	G/A	*BAT2*	INTRON	2	2.898	2.450	3.427	**1.75E-35**	0.073	0.041	7.40E-02	-0.192	0.122	1.20E-01	0.123	0.041	**2.40E-03**	-0.275	0.119	**2.36E-02**	-0.059	0.041	1.50E-01	0.036	0.075	6.35E-01
rs3129985	6	30870521	A/G	*IER3 | DDR1*	INTERGENIC	2	3.177	2.648	3.813	**1.88E-35**	0.018	0.044	6.80E-01	-0.004	0.124	9.76E-01	0.243	0.044	**2.70E-08**	-0.155	0.122	2.07E-01	-0.241	0.044	**5.10E-08**	0.011	0.074	8.80E-01
rs2535296	6	31168198	G/A	*HCG22 | C6orf15*	INTERGENIC	2	2.953	2.489	3.503	**2.03E-35**	-	-	-	-0.056	0.126	6.58E-01	-	-	-	-0.198	0.122	1.11E-01	-	-	-	-0.014	0.075	8.49E-01
rs2143462	6	32443182	A/G	*C6orf10*	INTRON	2	2.845	2.412	3.356	**2.15E-35**	0.081	0.040	**4.60E-02**	-0.104	0.122	3.99E-01	0.102	0.040	**1.05E-02**	-0.219	0.119	7.13E-02	-0.043	0.040	2.90E-01	0.035	0.074	6.34E-01
rs3094125	6	30817336	A/G	*FLOT1*	INTRON	2	3.200	2.663	3.846	**2.40E-35**	-	-	-	-0.026	0.125	8.35E-01	-	-	-	-0.033	0.124	7.89E-01	-	-	-	0.018	0.075	8.13E-01
rs3132580	6	31028103	A/G	*DPCR1*	CODING	2	3.165	2.638	3.797	**2.54E-35**	-0.006	0.044	9.00E-01	-0.065	0.125	6.07E-01	0.218	0.043	**4.30E-07**	-0.173	0.123	1.63E-01	-0.235	0.044	**7.20E-08**	0.015	0.075	8.40E-01
rs3130623	6	31705679	A/G	*BAT2*	INTRON	2	2.858	2.421	3.374	**2.75E-35**	0.073	0.041	7.40E-02	-0.200	0.121	1.03E-01	0.123	0.041	**2.40E-03**	-0.282	0.118	**1.99E-02**	-0.059	0.041	1.50E-01	0.021	0.075	7.75E-01
rs3094086	6	31027370	A/G	*LOC100129065 | DPCR1*	INTERGENIC	2	3.151	2.627	3.779	**3.34E-35**	-0.006	0.044	9.00E-01	-0.065	0.125	6.07E-01	0.218	0.043	**4.30E-07**	-0.173	0.123	1.63E-01	-0.235	0.044	**7.20E-08**	0.015	0.075	8.40E-01
rs9262143	6	30760760	A/G	*KIAA1949*	CODING	2	3.178	2.645	3.817	**4.45E-35**	0.011	0.046	8.00E-01	-0.012	0.125	9.21E-01	0.273	0.045	**1.30E-09**	-0.166	0.122	1.80E-01	-0.279	0.046	**1.00E-09**	0.026	0.075	7.33E-01
rs886423	6	30890184	G/C	*IER3 | DDR1*	INTERGENIC	2	3.034	2.544	3.618	**4.85E-35**	-0.014	0.041	7.40E-01	-0.044	0.124	7.23E-01	0.159	0.040	**8.10E-05**	-0.134	0.122	2.78E-01	-0.177	0.041	**1.50E-05**	-0.028	0.074	7.09E-01
rs1264352	6	30897626	C/G	*IER3 | DDR1*	INTERGENIC	2	3.033	2.543	3.618	**5.00E-35**	-0.011	0.041	7.80E-01	0.040	0.124	7.47E-01	0.160	0.040	**7.20E-05**	-0.044	0.123	7.18E-01	-0.177	0.041	**1.50E-05**	-0.034	0.074	6.49E-01
rs1264350	6	30904524	G/A	*IER3 | DDR1*	INTERGENIC	2	3.131	2.612	3.753	**5.42E-35**	-0.002	0.045	9.70E-01	-0.025	0.125	8.40E-01	0.267	0.045	**2.20E-09**	-0.157	0.122	2.03E-01	-0.285	0.045	**2.50E-10**	0.029	0.074	6.96E-01
rs3132585	6	30795593	G/C	*MDC1 | TUBB*	INTERGENIC	2	3.189	2.653	3.834	**5.51E-35**	-	-	-	-0.026	0.125	8.35E-01	-	-	-	-0.033	0.124	7.89E-01	-	-	-	0.018	0.075	8.13E-01
rs3132581	6	31021437	A/G	*SFTPG | LOC100129065*	INTERGENIC	2	3.161	2.633	3.796	**6.13E-35**	-0.006	0.044	9.00E-01	-0.068	0.124	5.86E-01	0.218	0.043	**4.30E-07**	-0.179	0.122	1.47E-01	-0.235	0.044	**7.20E-08**	0.015	0.075	8.36E-01
rs9262132	6	30719329	A/C	*C6orf134*	UTR	2	3.159	2.629	3.795	**1.00E-34**	-	-	-	-0.012	0.125	9.21E-01	-	-	-	-0.166	0.122	1.80E-01	-	-	-	0.028	0.075	7.12E-01
rs3131934	6	31039823	G/A	*LOC100129065 | C6orf205*	INTERGENIC	2	3.110	2.595	3.727	**1.01E-34**	-0.006	0.043	8.90E-01	-0.039	0.125	7.54E-01	0.225	0.042	**1.10E-07**	-0.150	0.123	2.29E-01	-0.245	0.043	**1.10E-08**	-0.004	0.075	9.63E-01
rs1800629	6	31651010	A/G	*LTA | TNF*	INTERGENIC	2	2.869	2.424	3.396	**1.69E-34**	-0.004	0.039	9.20E-01	-0.010	0.123	9.33E-01	0.104	0.039	**7.40E-03**	-0.177	0.121	1.46E-01	-0.107	0.039	**5.90E-03**	-0.004	0.074	9.58E-01
rs3132645	6	30517228	G/A	*RPP21 | HLA-E*	INTERGENIC	2	3.181	2.643	3.828	**1.72E-34**	0.004	0.046	9.40E-01	0.005	0.126	9.69E-01	0.256	0.045	**1.30E-08**	-0.013	0.125	9.15E-01	-0.265	0.046	**6.10E-09**	0.025	0.075	7.37E-01
rs3129822	6	30454187	A/C	*RPP21 | HLA-E*	INTERGENIC	2	3.226	2.675	3.892	**1.89E-34**	0.021	0.047	6.60E-01	0.033	0.125	7.89E-01	0.274	0.047	**3.80E-09**	-0.151	0.122	2.21E-01	-0.270	0.047	**1.10E-08**	0.023	0.075	7.58E-01
rs9262135	6	30726885	G/A	*C6orf136*	INTRON	2	3.160	2.628	3.799	**1.94E-34**	0.003	0.045	9.40E-01	-0.012	0.125	9.21E-01	0.258	0.045	**7.10E-09**	-0.166	0.122	1.80E-01	-0.270	0.045	**2.30E-09**	0.028	0.075	7.12E-01
rs56133930	6	30456439	G/A	*RPP21 | HLA-E*	INTERGENIC	2	3.201	2.655	3.858	**2.80E-34**	-	-	-	0.033	0.125	7.89E-01	-	-	-	-0.151	0.122	2.21E-01	-	-	-	0.023	0.075	7.58E-01
rs3130247	6	30623022	G/A	*GNL1*	CODING	2	3.145	2.617	3.781	**2.98E-34**	-0.008	0.045	8.50E-01	-0.012	0.125	9.21E-01	0.247	0.044	**2.80E-08**	-0.166	0.122	1.80E-01	-0.268	0.045	**2.80E-09**	0.024	0.075	7.52E-01
rs3132451	6	31690004	C/G	*NCR3 | AIF1*	INTERGENIC	2	2.797	2.370	3.300	**3.79E-34**	0.059	0.040	1.36E-01	-0.222	0.122	7.42E-02	0.099	0.039	**1.18E-02**	-0.255	0.120	**3.81E-02**	-0.040	0.040	3.20E-01	0.028	0.076	7.13E-01
rs3130126	6	30461718	A/C	*RPP21 | HLA-E*	INTERGENIC	2	3.197	2.651	3.855	**4.62E-34**	-	-	-	0.033	0.125	7.89E-01	-	-	-	-0.151	0.122	2.21E-01	-	-	-	0.023	0.075	7.58E-01
rs3130141	6	30540156	A/C	*RPP21 | HLA-E*	INTERGENIC	2	3.140	2.611	3.775	**4.64E-34**	-0.003	0.045	9.40E-01	0.005	0.126	9.69E-01	0.252	0.045	**2.10E-08**	-0.013	0.125	9.15E-01	-0.267	0.046	**4.40E-09**	0.025	0.075	7.37E-01
rs3132630	6	30453097	A/G	*RPP21 | HLA-E*	INTERGENIC	2	3.197	2.651	3.855	**4.67E-34**	0.021	0.047	6.60E-01	0.033	0.125	7.89E-01	0.274	0.047	**3.80E-09**	-0.151	0.122	2.21E-01	-0.270	0.047	**1.10E-08**	0.023	0.075	7.58E-01
rs3094703	6	30466936	A/G	*RPP21 | HLA-E*	INTERGENIC	2	3.196	2.651	3.855	**4.76E-34**	0.021	0.047	6.60E-01	0.033	0.125	7.89E-01	0.274	0.047	**3.80E-09**	-0.151	0.122	2.21E-01	-0.270	0.047	**1.10E-08**	0.023	0.075	7.58E-01
rs3129818	6	30450945	A/C	*RPP21 | HLA-E*	INTERGENIC	2	3.196	2.651	3.855	**4.76E-34**	0.021	0.047	6.60E-01	0.033	0.125	7.89E-01	0.274	0.047	**3.80E-09**	-0.151	0.122	2.21E-01	-0.270	0.047	**1.10E-08**	0.023	0.075	7.58E-01
rs3129820	6	30451548	A/G	*RPP21 | HLA-E*	INTERGENIC	2	3.196	2.651	3.855	**4.76E-34**	0.021	0.047	6.60E-01	0.033	0.125	7.89E-01	0.274	0.047	**3.80E-09**	-0.151	0.122	2.21E-01	-0.270	0.047	**1.10E-08**	0.023	0.075	7.58E-01
rs3132625	6	30455699	G/A	*RPP21 | HLA-E*	INTERGENIC	2	3.196	2.651	3.855	**4.76E-34**	0.021	0.047	6.60E-01	0.033	0.125	7.89E-01	0.274	0.047	**3.80E-09**	-0.151	0.122	2.21E-01	-0.270	0.047	**1.10E-08**	0.023	0.075	7.58E-01
rs3132638	6	30449450	G/A	*RPP21 | HLA-E*	INTERGENIC	2	3.196	2.651	3.855	**4.76E-34**	-	-	-	0.033	0.125	7.89E-01	-	-	-	-0.151	0.122	2.21E-01	-	-	-	0.023	0.075	7.58E-01
rs3129821	6	30452017	A/G	*RPP21 | HLA-E*	INTERGENIC	2	3.196	2.651	3.855	**4.82E-34**	0.021	0.047	6.60E-01	0.033	0.125	7.89E-01	0.274	0.047	**3.80E-09**	-0.151	0.122	2.21E-01	-0.270	0.047	**1.10E-08**	0.023	0.075	7.58E-01
rs3132631	6	30452624	A/G	*RPP21 | HLA-E*	INTERGENIC	2	3.196	2.651	3.855	**4.82E-34**	0.021	0.047	6.60E-01	0.033	0.125	7.89E-01	0.274	0.047	**3.80E-09**	-0.151	0.122	2.21E-01	-0.270	0.047	**1.10E-08**	0.023	0.075	7.58E-01
rs3129809	6	30443600	G/A	*RPP21 | HLA-E*	INTERGENIC	2	3.196	2.650	3.854	**4.97E-34**	0.023	0.047	6.20E-01	0.033	0.125	7.89E-01	0.271	0.047	**6.10E-09**	-0.151	0.122	2.21E-01	-0.263	0.047	**2.30E-08**	0.023	0.075	7.58E-01
rs3130123	6	30460626	A/G	*RPP21 | HLA-E*	INTERGENIC	2	3.196	2.650	3.854	**4.97E-34**	-	-	-	0.033	0.125	7.89E-01	-	-	-	-0.151	0.122	2.21E-01	-	-	-	0.023	0.075	7.58E-01
rs3130370	6	30453273	G/A	*RPP21 | HLA-E*	INTERGENIC	2	3.196	2.650	3.854	**4.97E-34**	0.021	0.047	6.60E-01	0.033	0.125	7.89E-01	0.274	0.047	**3.80E-09**	-0.151	0.122	2.21E-01	-0.270	0.047	**1.10E-08**	0.023	0.075	7.58E-01
rs3130662	6	30821787	A/C	*IER3 | DDR1*	INTERGENIC	2	2.972	2.493	3.542	**5.43E-34**	-	-	-	-0.015	0.124	9.01E-01	-	-	-	-0.032	0.123	7.98E-01	-	-	-	-0.026	0.074	7.26E-01
rs3129877	6	32516575	A/G	*HLA-DRA*	INTRON	2	2.603	2.231	3.038	**5.84E-34**	0.028	0.036	4.30E-01	-0.069	0.123	5.74E-01	0.035	0.035	3.20E-01	-0.241	0.119	**4.61E-02**	-0.021	0.036	5.60E-01	0.035	0.074	6.35E-01
rs9276931	6	33036962	G/A	*HLA-DMA | BRD2*	INTERGENIC	2	3.321	2.736	4.031	**5.87E-34**	0.031	0.054	5.70E-01	-0.076	0.123	5.42E-01	0.119	0.053	**2.50E-02**	0.103	0.122	4.02E-01	-0.088	0.054	1.03E-01	-0.001	0.074	9.94E-01
rs3094024	6	30603839	G/A	*HLA-E | GNL1*	INTERGENIC	2	3.138	2.609	3.774	**5.91E-34**	-0.008	0.045	8.50E-01	-0.012	0.125	9.21E-01	0.247	0.044	**2.80E-08**	-0.166	0.122	1.80E-01	-0.268	0.045	**2.80E-09**	0.024	0.075	7.52E-01
rs3131064	6	30871872	G/A	*IER3 | DDR1*	INTERGENIC	2	2.925	2.460	3.479	**6.29E-34**	0.013	0.040	7.50E-01	-0.042	0.124	7.36E-01	0.163	0.040	**4.20E-05**	-0.168	0.122	1.71E-01	-0.156	0.040	**1.00E-04**	-0.016	0.074	8.29E-01
rs910051	6	32423489	C/A	*C6orf10*	INTRON	1	3.560	2.900	4.370	**6.59E-34**	-	-	-	-0.053	0.125	6.76E-01	-	-	-	-0.173	0.123	1.64E-01	-	-	-	0.054	0.077	4.83E-01
rs3129817	6	30450732	A/G	*RPP21 | HLA-E*	INTERGENIC	2	3.155	2.621	3.799	**6.68E-34**	-	-	-	0.036	0.124	7.70E-01	-	-	-	-0.141	0.122	2.54E-01	-	-	-	0.024	0.075	7.47E-01
rs2734583	6	31613459	G/A	*BAT1*	INTRON	1	3.520	2.872	4.314	**7.15E-34**	0.045	0.046	3.20E-01	-0.044	0.124	7.21E-01	0.281	0.045	**4.90E-10**	-0.157	0.122	2.01E-01	-0.256	0.046	**2.30E-08**	-0.003	0.075	9.67E-01
rs3132616	6	30524435	G/A	*RPP21 | HLA-E*	INTERGENIC	2	3.143	2.611	3.783	**8.74E-34**	-0.003	0.045	9.40E-01	0.015	0.125	9.07E-01	0.252	0.045	**2.10E-08**	-0.154	0.123	2.16E-01	-0.267	0.046	**4.40E-09**	0.033	0.075	6.57E-01
rs2233956	6	31189184	G/A	*C6orf15 | PSORS1C1*	INTERGENIC	2	2.935	2.466	3.494	**9.66E-34**	0.030	0.039	4.50E-01	-0.046	0.124	7.13E-01	0.174	0.038	**5.30E-06**	-0.145	0.122	2.39E-01	-0.163	0.039	**2.50E-05**	0.005	0.074	9.52E-01

rs3132610	6	30652380	G/A	*ABCF1*	INTRON	2	3.073	2.562	3.686	**1.08E-33**	-0.009	0.045	8.50E-01	-0.012	0.125	9.21E-01	0.246	0.044	**3.20E-08**	-0.166	0.122	1.80E-01	-0.266	0.045	**3.30E-09**	0.024	0.075	7.52E-01
rs3130477	6	31536899	G/A	*LOC100129668 | HCP5*	INTERGENIC	1	3.580	2.911	4.402	**1.17E-33**	-	-	-	0.009	0.125	9.42E-01	-	-	-	-0.152	0.122	2.20E-01	-	-	-	0.007	0.075	9.28E-01
rs3132627	6	30454739	T/A	*RPP21 | HLA-E*	INTERGENIC	2	3.211	2.657	3.880	**1.42E-33**	0.021	0.047	6.60E-01	0.059	0.124	6.38E-01	0.274	0.047	**3.80E-09**	-0.145	0.122	2.40E-01	-0.270	0.047	**1.10E-08**	0.041	0.075	5.80E-01
rs3094054	6	30441484	A/C	*RPP21 | HLA-E*	INTERGENIC	2	3.169	2.628	3.822	**1.43E-33**	0.023	0.047	6.20E-01	0.059	0.124	6.38E-01	0.271	0.047	**6.10E-09**	-0.145	0.122	2.40E-01	-0.263	0.047	**2.30E-08**	0.041	0.075	5.84E-01
rs983561	6	32511633	C/A	*BTNL2 | HLA-DRA*	INTERGENIC	2	2.570	2.205	2.996	**1.66E-33**	0.024	0.036	5.10E-01	-0.065	0.123	5.98E-01	0.034	0.035	3.30E-01	-0.228	0.119	5.91E-02	-0.023	0.036	5.10E-01	0.036	0.074	6.29E-01
rs3094050	6	30466570	G/A	*RPP21 | HLA-E*	INTERGENIC	2	3.174	2.631	3.830	**1.74E-33**	0.021	0.047	6.60E-01	0.033	0.125	7.89E-01	0.274	0.047	**3.80E-09**	-0.151	0.122	2.21E-01	-0.270	0.047	**1.10E-08**	0.023	0.075	7.58E-01
rs3131783	6	31040047	A/G	*LOC100129065 | C6orf205*	INTERGENIC	2	3.028	2.528	3.626	**2.26E-33**	-0.003	0.045	9.40E-01	-0.049	0.125	6.97E-01	0.232	0.044	**1.40E-07**	-0.152	0.123	2.20E-01	-0.249	0.045	**2.40E-08**	0.005	0.074	9.46E-01
rs3129876	6	32515990	A/G	*HLA-DRA*	INTRON	2	2.607	2.230	3.048	**2.33E-33**	0.025	0.036	4.90E-01	-0.065	0.123	5.98E-01	0.033	0.035	3.50E-01	-0.228	0.119	5.91E-02	-0.021	0.036	5.50E-01	0.036	0.074	6.29E-01
rs3131921	6	31015314	G/A	*SFTPG | LOC100129065*	INTERGENIC	2	3.009	2.515	3.601	**2.63E-33**	-	-	-	-0.068	0.124	5.86E-01	-	-	-	-0.179	0.122	1.47E-01	-	-	-	0.010	0.075	8.88E-01
rs3129881	6	32517462	A/G	*HLA-DRA*	INTRON	2	2.594	2.220	3.029	**2.65E-33**	0.025	0.036	4.90E-01	-0.065	0.123	5.98E-01	0.036	0.035	3.00E-01	-0.228	0.119	5.91E-02	-0.025	0.036	4.90E-01	0.036	0.074	6.29E-01
rs3129872	6	32515131	A/T	*BTNL2 | HLA-DRA*	INTERGENIC	2	2.582	2.212	3.013	**2.71E-33**	0.024	0.036	5.10E-01	-0.065	0.123	5.98E-01	0.034	0.035	3.30E-01	-0.228	0.119	5.91E-02	-0.023	0.036	5.10E-01	0.036	0.074	6.29E-01
rs3135342	6	32504593	A/C	*BTNL2 | HLA-DRA*	INTERGENIC	2	2.582	2.212	3.013	**2.74E-33**	0.028	0.036	4.40E-01	-0.065	0.123	5.98E-01	0.036	0.035	3.10E-01	-0.228	0.119	5.91E-02	-0.022	0.036	5.40E-01	0.036	0.074	6.29E-01
rs3130116	6	30473427	G/A	*RPP21 | HLA-E*	INTERGENIC	2	3.112	2.586	3.745	**2.76E-33**	-	-	-	0.015	0.125	9.07E-01	-	-	-	-0.154	0.123	2.16E-01	-	-	-	0.037	0.075	6.19E-01
rs3129812	6	30445953	A/G	*RPP21 | HLA-E*	INTERGENIC	2	3.163	2.622	3.817	**2.77E-33**	0.021	0.047	6.60E-01	0.059	0.124	6.38E-01	0.274	0.047	**3.80E-09**	-0.145	0.122	2.40E-01	-0.270	0.047	**1.10E-08**	0.041	0.075	5.80E-01
rs3129858	6	32508498	A/G	*BTNL2 | HLA-DRA*	INTERGENIC	2	2.581	2.211	3.013	**2.82E-33**	0.024	0.036	5.10E-01	-0.065	0.123	5.98E-01	0.034	0.035	3.30E-01	-0.228	0.119	5.91E-02	-0.023	0.036	5.10E-01	0.036	0.074	6.29E-01
rs3130352	6	30436336	A/G	*RPP21 | HLA-E*	INTERGENIC	2	3.190	2.640	3.854	**2.83E-33**	0.023	0.047	6.20E-01	0.059	0.124	6.38E-01	0.271	0.047	**6.10E-09**	-0.145	0.122	2.40E-01	-0.263	0.047	**2.30E-08**	0.041	0.075	5.80E-01
rs3129848	6	32505027	A/G	*BTNL2 | HLA-DRA*	INTERGENIC	2	2.581	2.211	3.012	**2.84E-33**	0.028	0.036	4.40E-01	-0.065	0.123	5.98E-01	0.036	0.035	3.10E-01	-0.228	0.119	5.91E-02	-0.022	0.036	5.40E-01	0.036	0.074	6.29E-01
rs3129853	6	32506626	A/G	*BTNL2 | HLA-DRA*	INTERGENIC	2	2.581	2.211	3.012	**2.84E-33**	-	-	-	-0.065	0.123	5.98E-01	-	-	-	-0.228	0.119	5.91E-02	-	-	-	0.036	0.074	6.29E-01
rs3135339	6	32507239	C/G	*BTNL2 | HLA-DRA*	INTERGENIC	2	2.581	2.211	3.012	**2.84E-33**	0.027	0.036	4.50E-01	-0.065	0.123	5.98E-01	0.034	0.035	3.30E-01	-0.228	0.119	5.91E-02	-0.020	0.036	5.70E-01	0.036	0.074	6.29E-01
rs2395172	6	32507820	G/A	*BTNL2 | HLA-DRA*	INTERGENIC	2	2.581	2.211	3.012	**2.89E-33**	0.024	0.036	5.10E-01	-0.065	0.123	5.98E-01	0.034	0.035	3.30E-01	-0.228	0.119	5.91E-02	-0.023	0.036	5.10E-01	0.036	0.074	6.29E-01
rs3094624	6	30434788	A/T	*RPP21 | HLA-E*	INTERGENIC	2	3.159	2.618	3.812	**3.32E-33**	-	-	-	0.059	0.124	6.38E-01	-	-	-	-0.145	0.122	2.40E-01	-	-	-	0.041	0.075	5.80E-01
rs3130350	6	30435818	A/C	*RPP21 | HLA-E*	INTERGENIC	2	3.158	2.617	3.811	**3.41E-33**	0.023	0.047	6.20E-01	0.059	0.124	6.38E-01	0.271	0.047	**6.10E-09**	-0.145	0.122	2.40E-01	-0.263	0.047	**2.30E-08**	0.041	0.075	5.80E-01
rs3130377	6	30431372	A/G	*RPP21 | HLA-E*	INTERGENIC	2	3.158	2.617	3.811	**3.41E-33**	0.023	0.047	6.20E-01	0.059	0.124	6.38E-01	0.271	0.047	**6.10E-09**	-0.145	0.122	2.40E-01	-0.263	0.047	**2.30E-08**	0.041	0.075	5.80E-01
rs3094058	6	30435175	A/G	*RPP21 | HLA-E*	INTERGENIC	2	3.158	2.617	3.810	**3.45E-33**	-	-	-	0.059	0.124	6.38E-01	-	-	-	-0.145	0.122	2.40E-01	-	-	-	0.041	0.075	5.80E-01
rs2105902	6	32503676	A/T	*BTNL2 | HLA-DRA*	INTERGENIC	1	3.180	2.632	3.843	**4.43E-33**	-	-	-	-0.068	0.123	5.82E-01	-	-	-	-0.219	0.120	7.20E-02	-	-	-	0.057	0.075	4.49E-01
rs2395181	6	32515382	C/G	*BTNL2 | HLA-DRA*	INTERGENIC	2	2.568	2.200	2.998	**6.04E-33**	0.023	0.036	5.20E-01	-0.065	0.123	5.98E-01	0.034	0.035	3.40E-01	-0.228	0.119	5.91E-02	-0.024	0.036	5.10E-01	0.036	0.074	6.29E-01
rs1064627	6	30806520	G/A	*FLOT1*	INTRON	2	2.901	2.436	3.455	**7.70E-33**	0.022	0.038	5.60E-01	-0.004	0.123	9.77E-01	0.152	0.038	**5.20E-05**	-0.042	0.122	7.30E-01	-0.133	0.038	**4.20E-04**	-0.035	0.074	6.33E-01
rs3129875	6	32515446	G/A	*BTNL2 | HLA-DRA*	INTERGENIC	2	2.569	2.200	3.000	**9.32E-33**	0.014	0.035	7.00E-01	-0.065	0.123	5.98E-01	0.033	0.035	3.50E-01	-0.228	0.119	5.91E-02	-0.030	0.035	4.00E-01	0.036	0.074	6.29E-01
rs3129815	6	30448507	A/G	*RPP21 | HLA-E*	INTERGENIC	2	3.153	2.610	3.809	**1.16E-32**	0.021	0.047	6.60E-01	0.059	0.124	6.38E-01	0.274	0.047	**3.80E-09**	-0.145	0.122	2.40E-01	-0.270	0.047	**1.10E-08**	0.041	0.075	5.80E-01
rs3129878	6	32516713	C/A	*HLA-DRA*	INTRON	2	2.556	2.190	2.983	**1.18E-32**	0.028	0.036	4.30E-01	-0.055	0.123	6.56E-01	0.035	0.035	3.20E-01	-0.245	0.118	**4.24E-02**	-0.021	0.036	5.60E-01	0.033	0.074	6.54E-01
rs3130351	6	30436171	A/G	*RPP21 | HLA-E*	INTERGENIC	2	3.151	2.608	3.807	**1.24E-32**	0.023	0.047	6.20E-01	0.059	0.124	6.38E-01	0.271	0.047	**6.10E-09**	-0.145	0.122	2.40E-01	-0.263	0.047	**2.30E-08**	0.041	0.075	5.80E-01
rs3094034	6	30471330	T/A	*RPP21 | HLA-E*	INTERGENIC	2	3.151	2.608	3.807	**1.25E-32**	0.020	0.047	6.60E-01	0.059	0.124	6.38E-01	0.275	0.047	**3.70E-09**	-0.145	0.122	2.40E-01	-0.270	0.047	**1.00E-08**	0.041	0.075	5.80E-01
rs3094036	6	30471064	C/G	*RPP21 | HLA-E*	INTERGENIC	2	3.151	2.608	3.807	**1.25E-32**	0.020	0.047	6.60E-01	0.059	0.124	6.38E-01	0.275	0.047	**3.70E-09**	-0.145	0.122	2.40E-01	-0.270	0.047	**1.00E-08**	0.041	0.075	5.80E-01
rs3094056	6	30438050	A/G	*RPP21 | HLA-E*	INTERGENIC	2	3.143	2.603	3.796	**1.27E-32**	-	-	-	0.059	0.124	6.38E-01	-	-	-	-0.145	0.122	2.40E-01	-	-	-	0.038	0.075	6.15E-01
rs3094057	6	30437945	A/G	*RPP21 | HLA-E*	INTERGENIC	2	3.143	2.603	3.796	**1.27E-32**	0.023	0.047	6.20E-01	0.059	0.124	6.38E-01	0.271	0.047	**6.10E-09**	-0.145	0.122	2.40E-01	-0.263	0.047	**2.30E-08**	0.038	0.075	6.15E-01
rs1264377	6	30872886	A/G	*IER3 | DDR1*	INTERGENIC	2	2.885	2.421	3.437	**1.97E-32**	0.007	0.043	8.70E-01	-0.062	0.123	6.17E-01	0.226	0.043	**1.20E-07**	-0.068	0.122	5.82E-01	-0.234	0.043	**6.60E-08**	0.003	0.074	9.63E-01
rs3094622	6	30435931	G/A	*RPP21 | HLA-E*	INTERGENIC	2	3.094	2.566	3.730	**2.46E-32**	0.023	0.047	6.20E-01	0.059	0.124	6.38E-01	0.271	0.047	**6.10E-09**	-0.145	0.122	2.40E-01	-0.263	0.047	**2.30E-08**	0.041	0.075	5.82E-01
rs3094621	6	30436732	G/A	*RPP21 | HLA-E*	INTERGENIC	2	3.093	2.566	3.729	**2.54E-32**	-	-	-	0.059	0.124	6.38E-01	-	-	-	-0.145	0.122	2.40E-01	-	-	-	0.041	0.075	5.82E-01
rs3129953	6	32469799	A/G	*C6orf10 | BTNL2*	INTERGENIC	1	3.130	2.591	3.781	**2.70E-32**	0.051	0.041	2.10E-01	0.008	0.123	9.47E-01	0.046	0.041	2.50E-01	-0.139	0.121	2.56E-01	-0.007	0.041	8.60E-01	0.051	0.075	4.92E-01
rs3132647	6	30438716	G/A	*RPP21 | HLA-E*	INTERGENIC	2	3.113	2.578	3.758	**3.34E-32**	0.023	0.047	6.20E-01	0.059	0.124	6.38E-01	0.271	0.047	**6.10E-09**	-0.145	0.122	2.40E-01	-0.263	0.047	**2.30E-08**	0.043	0.075	5.69E-01
rs3129963	6	32488186	G/A	*BTNL2 | HLA-DRA*	INTERGENIC	1	3.000	2.500	3.600	**3.45E-32**	0.049	0.041	2.20E-01	-0.054	0.123	6.64E-01	0.057	0.040	1.60E-01	-0.203	0.120	9.44E-02	-0.019	0.040	6.40E-01	0.027	0.074	7.16E-01
rs3135375	6	32493551	A/G	*BTNL2 | HLA-DRA*	INTERGENIC	1	3.000	2.500	3.600	**3.45E-32**	0.071	0.042	9.40E-02	-0.128	0.122	2.98E-01	0.068	0.042	1.08E-01	-0.279	0.117	**2.01E-02**	-0.013	0.042	7.60E-01	0.032	0.074	6.67E-01
rs3132634	6	30451251	C/A	*RPP21 | HLA-E*	INTERGENIC	2	3.113	2.578	3.759	**3.56E-32**	0.021	0.047	6.60E-01	0.056	0.124	6.56E-01	0.274	0.047	**3.80E-09**	-0.155	0.122	2.09E-01	-0.270	0.047	**1.10E-08**	0.041	0.075	5.88E-01
rs1264326	6	30959888	A/C	*DDR1*	UTR	1	3.520	2.852	4.344	**8.93E-32**	-	-	-	-0.017	0.125	8.95E-01	-	-	-	-0.152	0.123	2.22E-01	-	-	-	-0.004	0.075	9.60E-01
rs3130364	6	30427133	T/A	*RPP21 | HLA-E*	INTERGENIC	2	3.075	2.547	3.711	**1.23E-31**	0.047	0.047	3.20E-01	0.021	0.125	8.70E-01	0.285	0.047	**1.10E-09**	-0.152	0.122	2.19E-01	-0.261	0.047	**4.00E-08**	-0.052	0.075	4.93E-01
rs3130564	6	31209653	A/G	*PSORS1C1 | PSORS1C2*	INTERGENIC	2	2.669	2.264	3.147	**1.54E-31**	0.028	0.037	4.40E-01	-0.083	0.125	5.09E-01	0.119	0.036	**1.05E-03**	-0.228	0.122	6.58E-02	-0.099	0.036	**6.70E-03**	0.015	0.075	8.38E-01
rs3130349	6	32255674	A/G	*RNF5*	CODING	2	2.782	2.343	3.303	**1.63E-31**	0.097	0.041	**1.90E-02**	-0.104	0.124	4.05E-01	0.115	0.041	**4.80E-03**	-0.204	0.121	9.80E-02	-0.040	0.041	3.30E-01	0.020	0.075	7.89E-01
rs1634721	6	31085659	A/G	*C6orf205 | LOC729792*	INTERGENIC	1	3.470	2.815	4.278	**2.15E-31**	0.014	0.045	7.60E-01	-0.054	0.124	6.68E-01	0.280	0.044	**2.60E-10**	-0.151	0.122	2.23E-01	-0.285	0.045	**1.80E-10**	0.007	0.075	9.28E-01
rs3134940	6	32257794	G/A	*AGER*	INTRON	2	2.775	2.337	3.295	**2.33E-31**	0.092	0.041	**2.60E-02**	-0.104	0.124	4.05E-01	0.102	0.041	**1.19E-02**	-0.204	0.121	9.80E-02	-0.031	0.041	4.50E-01	0.020	0.075	7.89E-01
rs3095340	6	30834918	C/A	*IER3 | DDR1*	INTERGENIC	2	2.867	2.401	3.424	**2.80E-31**	0.010	0.041	8.00E-01	-0.024	0.124	8.45E-01	0.184	0.040	**4.60E-06**	-0.177	0.121	1.49E-01	-0.186	0.041	**4.80E-06**	0.007	0.074	9.22E-01
rs3129884	6	32518188	G/A	*HLA-DRA*	INTRON	1	2.930	2.443	3.514	**4.68E-31**	-	-	-	-0.128	0.122	2.98E-01	-	-	-	-0.279	0.117	**2.01E-02**	-	-	-	0.032	0.074	6.67E-01
rs3094061	6	30429168	C/A	*RPP21 | HLA-E*	INTERGENIC	2	2.986	2.480	3.595	**8.06E-31**	0.050	0.047	2.90E-01	0.038	0.124	7.58E-01	0.282	0.047	**1.80E-09**	-0.156	0.122	2.04E-01	-0.254	0.047	**8.30E-08**	0.061	0.074	4.13E-01
rs3094067	6	30407224	C/A	*TRIM39*	INTRON	2	3.057	2.528	3.696	**8.35E-31**	0.040	0.047	3.90E-01	0.021	0.125	8.70E-01	0.286	0.047	**8.40E-10**	-0.152	0.122	2.19E-01	-0.269	0.047	**1.20E-08**	-0.048	0.075	5.20E-01
rs3130375	6	30429711	A/C	*RPP21 | HLA-E*	INTERGENIC	2	2.959	2.459	3.559	**1.32E-30**	0.023	0.047	6.20E-01	0.038	0.124	7.58E-01	0.271	0.047	**6.10E-09**	-0.156	0.122	2.04E-01	-0.263	0.047	**2.30E-08**	0.061	0.074	4.13E-01
rs3134605	6	32267934	G/A	*GPSM3*	CODING	2	2.664	2.254	3.148	**1.36E-30**	-	-	-	-0.147	0.123	2.38E-01	-	-	-	-0.086	0.123	4.90E-01	-	-	-	0.002	0.075	9.81E-01
rs3129859	6	32508917	G/C	*BTNL2 | HLA-DRA*	INTERGENIC	2	2.480	2.124	2.895	**1.38E-30**	0.012	0.035	7.30E-01	-0.084	0.123	4.96E-01	0.015	0.035	6.80E-01	-0.243	0.119	**4.39E-02**	-0.012	0.035	7.30E-01	0.042	0.074	5.66E-01
rs2523735	6	30230636	C/G	*TRIM10*	INTRON	2	2.905	2.421	3.487	**2.14E-30**	0.049	0.045	2.80E-01	0.044	0.125	7.25E-01	0.249	0.045	**2.80E-08**	-0.140	0.123	2.59E-01	-0.220	0.045	**1.30E-06**	0.033	0.075	6.63E-01
rs1264322	6	30965873	A/G	*DDR1*	INTRON	1	3.420	2.771	4.221	**2.36E-30**	-0.002	0.044	9.70E-01	-0.043	0.125	7.32E-01	0.213	0.043	**7.90E-07**	-0.155	0.123	2.09E-01	-0.226	0.044	**2.10E-07**	-0.004	0.075	9.59E-01
rs2157678	6	30236928	A/C	*TRIM10 | TRIM15*	INTERGENIC	2	3.007	2.490	3.631	**2.44E-30**	0.035	0.047	4.50E-01	0.021	0.125	8.70E-01	0.269	0.046	**4.90E-09**	-0.152	0.122	2.19E-01	-0.256	0.047	**3.80E-08**	-0.048	0.075	5.20E-01
rs3891175	6	32742445	A/G	*HLA-DQB1 | HLA-DQA2*	INTERGENIC	2	2.688	2.269	3.184	**2.46E-30**	0.042	0.037	2.70E-01	-0.088	0.124	4.83E-01	0.030	0.037	4.10E-01	-0.042	0.124	7.35E-01	0.008	0.037	8.30E-01	0.034	0.074	6.45E-01
rs11158	6	30260446	A/C	*TRIM26*	UTR	2	3.052	2.521	3.695	**2.60E-30**	0.046	0.047	3.30E-01	0.021	0.125	8.70E-01	0.279	0.046	**1.40E-09**	-0.152	0.122	2.19E-01	-0.258	0.047	**3.40E-08**	-0.048	0.075	5.20E-01
rs2857595	6	31676448	A/G	*NCR3 | AIF1*	INTERGENIC	2	2.581	2.194	3.037	**2.71E-30**	0.045	0.039	2.40E-01	-0.106	0.122	3.89E-01	0.133	0.038	**5.00E-04**	-0.210	0.120	8.38E-02	-0.094	0.039	**1.41E-02**	-0.022	0.074	7.65E-01
rs3115663	6	31709822	G/A	*BAT2*	INTRON	1	2.990	2.478	3.607	**2.78E-30**	0.073	0.041	7.40E-02	-0.192	0.122	1.20E-01	0.123	0.041	**2.40E-03**	-0.275	0.119	**2.36E-02**	-0.059	0.041	1.50E-01	0.036	0.075	6.35E-01
rs2844789	6	30220774	A/G	*TRIM40*	INTRON	2	3.020	2.499	3.650	**2.80E-30**	-	-	-	0.021	0.125	8.70E-01	-	-	-	-0.152	0.122	2.19E-01	-	-	-	-0.042	0.075	5.72E-01
rs3130376	6	30431088	A/G	*RPP21 | HLA-E*	INTERGENIC	2	2.917	2.427	3.505	**3.51E-30**	-	-	-	0.038	0.124	7.58E-01	-	-	-	-0.156	0.122	2.04E-01	-	-	-	0.061	0.074	4.13E-01
rs3132649	6	30429036	A/G	*RPP21 | HLA-E*	INTERGENIC	2	2.935	2.439	3.531	**3.63E-30**	0.047	0.047	3.20E-01	0.038	0.124	7.58E-01	0.285	0.047	**1.10E-09**	-0.156	0.122	2.04E-01	-0.261	0.047	**4.00E-08**	0.061	0.074	4.13E-01
rs3132947	6	32284760	A/C	*NOTCH4*	INTRON	2	2.641	2.235	3.122	**4.56E-30**	0.101	0.040	**1.21E-02**	-0.147	0.123	2.38E-01	0.067	0.040	9.20E-02	-0.086	0.123	4.90E-01	0.016	0.040	7.00E-01	0.002	0.075	9.81E-01
rs3094627	6	30429461	G/A	*RPP21 | HLA-E*	INTERGENIC	2	2.935	2.438	3.533	**5.10E-30**	0.023	0.047	6.20E-01	0.038	0.124	7.58E-01	0.271	0.047	**6.10E-09**	-0.156	0.122	2.04E-01	-0.263	0.047	**2.30E-08**	0.061	0.074	4.13E-01
rs2844657	6	30937501	G/A	*IER3 | DDR1*	INTERGENIC	2	2.571	2.184	3.025	**6.08E-30**	-0.032	0.039	4.10E-01	-0.056	0.124	6.54E-01	0.124	0.039	**1.42E-03**	-0.117	0.122	3.43E-01	-0.160	0.039	**4.60E-05**	0.000	0.075	9.99E-01

rs3132935	6	32279053	G/A	*NOTCH4*	INTRON	2	2.640	2.233	3.122	**7.25E-30**	0.102	0.040	**1.18E-02**	-0.147	0.123	2.38E-01	0.070	0.040	7.90E-02	-0.086	0.123	4.90E-01	0.012	0.040	7.60E-01	0.002	0.075	9.81E-01
rs2187823	6	32547486	A/G	*HLA-DRA | HLA-DRB5*	INTERGENIC	2	2.437	2.089	2.843	**8.02E-30**	-	-	-	-0.069	0.123	5.76E-01	-	-	-	-0.195	0.120	1.08E-01	-	-	-	-0.018	0.074	8.12E-01
rs9268976	6	32542852	A/T	*HLA-DRA | HLA-DRB5*	INTERGENIC	2	2.437	2.089	2.843	**8.02E-30**	0.047	0.050	3.50E-01	-0.069	0.123	5.76E-01	-0.157	0.050	**1.60E-03**	-0.195	0.120	1.08E-01	0.213	0.050	**2.00E-05**	-0.018	0.074	8.12E-01
rs2157338	6	32547301	A/G	*HLA-DRA | HLA-DRB5*	INTERGENIC	2	2.437	2.089	2.842	**8.20E-30**	-	-	-	-0.069	0.123	5.76E-01	-	-	-	-0.195	0.120	1.08E-01	-	-	-	-0.018	0.074	8.12E-01
rs7747521	6	32539083	G/A	*HLA-DRA | HLA-DRB5*	INTERGENIC	2	2.437	2.089	2.842	**8.20E-30**	0.071	0.038	6.20E-02	-0.069	0.123	5.76E-01	0.046	0.038	2.20E-01	-0.195	0.120	1.08E-01	0.010	0.038	8.00E-01	-0.018	0.074	8.12E-01
rs9268885	6	32539683	A/G	*HLA-DRA | HLA-DRB5*	INTERGENIC	2	2.437	2.089	2.842	**8.20E-30**	0.046	0.050	3.50E-01	-0.069	0.123	5.76E-01	-0.158	0.050	**1.60E-03**	-0.195	0.120	1.08E-01	0.214	0.050	**2.00E-05**	-0.018	0.074	8.12E-01
rs9268977	6	32542917	G/A	*HLA-DRA | HLA-DRB5*	INTERGENIC	2	2.437	2.089	2.842	**8.20E-30**	0.047	0.050	3.50E-01	-0.069	0.123	5.76E-01	-0.157	0.050	**1.60E-03**	-0.195	0.120	1.08E-01	0.213	0.050	**2.00E-05**	-0.018	0.074	8.12E-01
rs9268980	6	32543101	G/A	*HLA-DRA | HLA-DRB5*	INTERGENIC	2	2.437	2.089	2.842	**8.20E-30**	-	-	-	-0.069	0.123	5.76E-01	-	-	-	-0.195	0.120	1.08E-01	-	-	-	-0.018	0.074	8.12E-01
rs9269043	6	32546576	G/A	*HLA-DRA | HLA-DRB5*	INTERGENIC	2	2.437	2.089	2.842	**8.20E-30**	0.060	0.038	1.08E-01	-0.069	0.123	5.76E-01	0.035	0.037	3.40E-01	-0.195	0.120	1.08E-01	0.016	0.037	6.80E-01	-0.018	0.074	8.12E-01
rs9268862	6	32538145	C/A	*HLA-DRA | HLA-DRB5*	INTERGENIC	2	2.446	2.096	2.855	**8.78E-30**	0.062	0.038	1.02E-01	-0.069	0.123	5.76E-01	0.035	0.037	3.50E-01	-0.195	0.120	1.08E-01	0.018	0.038	6.40E-01	-0.018	0.074	8.12E-01
rs3130355	6	30426306	A/G	*RPP21 | HLA-E*	INTERGENIC	2	2.969	2.459	3.583	**9.28E-30**	-	-	-	0.021	0.125	8.70E-01	-	-	-	-0.152	0.122	2.19E-01	-	-	-	-0.048	0.075	5.20E-01
rs3094628	6	30393291	C/G	*LOC100133303*	INTRON	2	2.953	2.448	3.562	**1.06E-29**	0.048	0.047	3.00E-01	0.019	0.124	8.77E-01	0.286	0.046	**5.80E-10**	-0.162	0.122	1.87E-01	-0.262	0.047	**2.10E-08**	-0.048	0.075	5.20E-01
rs3130380	6	30387109	A/G	*LOC100133303*	INTRON	2	2.953	2.448	3.563	**1.29E-29**	0.040	0.047	3.90E-01	0.021	0.125	8.70E-01	0.286	0.047	**8.40E-10**	-0.152	0.122	2.19E-01	-0.269	0.047	**1.20E-08**	-0.048	0.075	5.20E-01
rs3130374	6	30429315	A/G	*RPP21 | HLA-E*	INTERGENIC	2	2.911	2.418	3.504	**1.43E-29**	0.050	0.047	2.90E-01	0.038	0.124	7.58E-01	0.282	0.047	**1.80E-09**	-0.156	0.122	2.04E-01	-0.254	0.047	**8.30E-08**	0.062	0.075	4.10E-01
rs3134945	6	32254470	A/C	*RNF5*	INTRON	2	2.616	2.213	3.091	**1.68E-29**	0.066	0.039	8.60E-02	-0.150	0.125	2.33E-01	0.071	0.038	6.30E-02	-0.062	0.125	6.19E-01	-0.010	0.039	7.90E-01	-0.009	0.075	9.01E-01
rs3134930	6	32299598	A/G	*NOTCH4*	INTRON	2	2.552	2.168	3.003	**1.69E-29**	0.075	0.041	6.80E-02	-0.147	0.123	2.36E-01	0.134	0.041	**9.80E-04**	-0.238	0.120	5.10E-02	-0.085	0.041	**3.70E-02**	0.142	0.074	5.77E-02
rs1573296	6	30235784	G/A	*TRIM10*	INTRON	2	2.944	2.440	3.552	**1.85E-29**	0.034	0.046	4.70E-01	0.021	0.125	8.70E-01	0.270	0.046	**4.30E-09**	-0.152	0.122	2.19E-01	-0.258	0.046	**2.80E-08**	-0.048	0.075	5.20E-01
rs3095153	6	31007174	A/G	*SFTPG*	UTR	1	3.310	2.688	4.076	**1.98E-29**	-0.002	0.044	9.70E-01	-0.054	0.126	6.70E-01	0.213	0.043	**7.90E-07**	-0.174	0.123	1.64E-01	-0.226	0.044	**2.10E-07**	0.019	0.075	8.03E-01
rs2106072	6	30261342	A/G	*TRIM26*	UTR	2	2.981	2.465	3.606	**2.02E-29**	0.047	0.047	3.10E-01	0.021	0.125	8.70E-01	0.280	0.046	**1.40E-09**	-0.152	0.122	2.19E-01	-0.257	0.047	**3.90E-08**	-0.048	0.075	5.20E-01
rs2517576	6	30923014	A/G	*IER3 | DDR1*	INTERGENIC	2	2.573	2.183	3.033	**2.07E-29**	-	-	-	-0.056	0.124	6.54E-01	-	-	-	-0.117	0.122	3.43E-01	-	-	-	0.000	0.075	9.99E-01
rs1264318	6	30971509	G/C	*DDR1*	INTRON	2	2.565	2.177	3.022	**2.08E-29**	-	-	-	-0.056	0.123	6.49E-01	-	-	-	-0.122	0.122	3.20E-01	-	-	-	0.000	0.074	9.98E-01
rs4428528	6	32538340	G/C	*HLA-DRA | HLA-DRB5*	INTERGENIC	2	2.437	2.087	2.847	**2.25E-29**	0.062	0.038	1.02E-01	-0.069	0.123	5.76E-01	0.035	0.037	3.50E-01	-0.195	0.120	1.08E-01	0.018	0.038	6.40E-01	-0.017	0.074	8.17E-01
rs9276731	6	32873563	A/C	*HLA-DQB2 | HLA-DOB*	INTERGENIC	1	3.370	2.727	4.165	**2.52E-29**	0.041	0.051	4.20E-01	-0.051	0.124	6.79E-01	0.159	0.050	**1.60E-03**	-0.190	0.121	1.21E-01	-0.136	0.051	**7.30E-03**	0.075	0.074	3.18E-01
rs3134608	6	32225949	C/A	*PRRT1*	INTRON	2	2.603	2.203	3.075	**2.55E-29**	0.064	0.038	9.70E-02	-0.150	0.125	2.33E-01	0.073	0.038	5.60E-02	-0.062	0.125	6.19E-01	-0.014	0.038	7.20E-01	-0.006	0.076	9.42E-01
rs3852215	6	32743479	G/A	*HLA-DQB1 | HLA-DQA2*	INTERGENIC	2	2.634	2.225	3.119	**2.81E-29**	-	-	-	-0.084	0.125	5.05E-01	-	-	-	-0.047	0.125	7.09E-01	-	-	-	0.055	0.075	4.62E-01
rs2844659	6	30932511	A/G	*IER3 | DDR1*	INTERGENIC	2	2.565	2.176	3.024	**3.12E-29**	-0.032	0.039	4.10E-01	-0.056	0.124	6.54E-01	0.124	0.039	**1.42E-03**	-0.117	0.122	3.43E-01	-0.160	0.039	**4.60E-05**	0.000	0.075	9.99E-01
rs1970	6	30233522	A/G	*TRIM10*	INTRON	2	2.936	2.432	3.545	**3.56E-29**	-	-	-	0.021	0.125	8.70E-01	-	-	-	-0.152	0.122	2.19E-01	-	-	-	-0.048	0.075	5.20E-01
rs5021448	6	32815684	A/G	*HLA-DQB1 | HLA-DQA2*	INTERGENIC	2	2.524	2.145	2.969	**6.14E-29**	0.024	0.039	5.40E-01	-0.045	0.123	7.16E-01	0.052	0.038	1.80E-01	-0.196	0.120	1.07E-01	-0.032	0.039	4.00E-01	0.076	0.074	3.06E-01
rs3094078	6	30332949	T/A	*TRIM26 | FLJ45422*	INTERGENIC	2	2.931	2.426	3.542	**8.55E-29**	0.042	0.047	3.70E-01	0.021	0.125	8.70E-01	0.288	0.047	**6.50E-10**	-0.152	0.122	2.19E-01	-0.271	0.047	**1.00E-08**	-0.048	0.075	5.20E-01
rs2523578	6	31436521	G/A	*HLA-B | LOC729816*	INTERGENIC	2	2.497	2.126	2.934	**8.60E-29**	-	-	-	0.036	0.124	7.74E-01	-	-	-	-0.161	0.122	1.93E-01	-	-	-	0.017	0.075	8.20E-01
rs2859078	6	32810427	G/A	*HLA-DQB1 | HLA-DQA2*	INTERGENIC	2	2.492	2.122	2.927	**9.79E-29**	0.026	0.039	5.10E-01	-0.031	0.123	8.04E-01	0.052	0.039	1.80E-01	-0.180	0.121	1.40E-01	-0.030	0.039	4.40E-01	0.075	0.074	3.09E-01
rs8321	6	30140501	C/A	*ZNRD1*	UTR	2	2.972	2.452	3.601	**1.06E-28**	0.028	0.048	5.60E-01	0.035	0.124	7.81E-01	0.293	0.047	**5.10E-10**	-0.133	0.122	2.82E-01	-0.287	0.048	**1.90E-09**	-0.042	0.075	5.75E-01
rs2023473	6	30183592	G/A	*TRIM31*	INTRON	2	2.917	2.415	3.523	**1.15E-28**	-	-	-	0.021	0.125	8.70E-01	-	-	-	-0.152	0.122	2.19E-01	-	-	-	-0.042	0.075	5.72E-01
rs3094064	6	30404232	A/G	*TRIM39*	INTRON	2	2.893	2.399	3.490	**1.15E-28**	0.040	0.047	3.90E-01	0.021	0.125	8.70E-01	0.286	0.047	**8.40E-10**	-0.152	0.122	2.19E-01	-0.269	0.047	**1.20E-08**	-0.048	0.075	5.20E-01
rs3096697	6	32242488	A/G	*EGFL8*	CODING	2	2.599	2.195	3.076	**1.22E-28**	0.065	0.038	9.20E-02	-0.150	0.125	2.33E-01	0.072	0.038	5.90E-02	-0.062	0.125	6.19E-01	-0.012	0.038	7.50E-01	-0.009	0.075	9.01E-01
rs763026	6	32799723	A/G	*HLA-DQB1 | HLA-DQA2*	INTERGENIC	2	2.498	2.125	2.937	**1.32E-28**	0.021	0.039	5.80E-01	-0.031	0.123	8.04E-01	0.050	0.038	2.00E-01	-0.180	0.121	1.40E-01	-0.032	0.039	4.10E-01	0.075	0.074	3.09E-01
rs9275652	6	32793800	C/G	*HLA-DQB1 | HLA-DQA2*	INTERGENIC	2	2.498	2.125	2.937	**1.32E-28**	0.021	0.039	5.80E-01	-0.031	0.123	8.04E-01	0.050	0.038	2.00E-01	-0.180	0.121	1.40E-01	-0.032	0.039	4.10E-01	0.075	0.074	3.09E-01
rs9275660	6	32794174	A/C	*HLA-DQB1 | HLA-DQA2*	INTERGENIC	2	2.498	2.125	2.937	**1.32E-28**	-	-	-	-0.031	0.123	8.04E-01	-	-	-	-0.180	0.121	1.40E-01	-	-	-	0.075	0.074	3.09E-01
rs9275686	6	32795548	A/G	*HLA-DQB1 | HLA-DQA2*	INTERGENIC	2	2.498	2.125	2.937	**1.32E-28**	0.021	0.039	5.80E-01	-0.031	0.123	8.04E-01	0.050	0.038	2.00E-01	-0.180	0.121	1.40E-01	-0.032	0.039	4.10E-01	0.075	0.074	3.09E-01
rs3129935	6	32444183	G/A	*C6orf10*	INTRON	1	2.860	2.375	3.443	**1.33E-28**	-	-	-	-0.104	0.122	3.99E-01	-	-	-	-0.219	0.119	7.13E-02	-	-	-	0.035	0.074	6.34E-01
rs9275936	6	32800911	A/G	*HLA-DQB1 | HLA-DQA2*	INTERGENIC	2	2.497	2.124	2.936	**1.37E-28**	0.021	0.039	5.80E-01	-0.031	0.123	8.04E-01	0.050	0.038	2.00E-01	-0.180	0.121	1.40E-01	-0.032	0.039	4.10E-01	0.075	0.074	3.09E-01
rs3095330	6	30801612	G/A	*TUBB | FLOT1*	INTERGENIC	2	2.565	2.171	3.031	**1.67E-28**	0.026	0.038	5.00E-01	-0.055	0.123	6.56E-01	0.154	0.038	**4.40E-05**	-0.063	0.122	6.10E-01	-0.132	0.038	**5.40E-04**	-0.030	0.074	6.89E-01
rs2523978	6	30191687	A/G	*TRIM31 | TRIM40*	INTERGENIC	2	2.945	2.433	3.566	**1.70E-28**	-	-	-	0.021	0.125	8.70E-01	-	-	-	-0.152	0.122	2.19E-01	-	-	-	-0.038	0.075	6.16E-01
rs2517622	6	30263128	G/C	*TRIM26*	INTRON	2	2.928	2.420	3.541	**1.82E-28**	0.047	0.047	3.10E-01	0.021	0.125	8.70E-01	0.280	0.046	**1.40E-09**	-0.152	0.122	2.19E-01	-0.257	0.047	**3.90E-08**	-0.048	0.075	5.20E-01
rs2523729	6	30245366	A/G	*TRIM15*	INTRON	2	2.920	2.415	3.531	**1.87E-28**	0.038	0.047	4.10E-01	0.021	0.125	8.70E-01	0.274	0.046	**2.60E-09**	-0.152	0.122	2.19E-01	-0.259	0.047	**2.80E-08**	-0.048	0.075	5.20E-01
rs3094127	6	30805426	G/A	*FLOT1*	INTRON	2	2.578	2.180	3.049	**1.90E-28**	0.022	0.038	5.60E-01	-0.011	0.123	9.27E-01	0.152	0.038	**5.20E-05**	-0.064	0.122	6.05E-01	-0.133	0.038	**4.20E-04**	-0.031	0.074	6.80E-01
rs3134946	6	32253971	C/G	*AGPAT1 | RNF5*	INTERGENIC	2	2.564	2.170	3.029	**1.94E-28**	0.066	0.039	8.60E-02	-0.150	0.125	2.33E-01	0.071	0.038	6.30E-02	-0.062	0.125	6.19E-01	-0.010	0.039	7.90E-01	-0.009	0.075	9.01E-01
rs3096689	6	32253977	C/G	*AGPAT1 | RNF5*	INTERGENIC	2	2.564	2.170	3.029	**1.95E-28**	0.066	0.039	8.60E-02	-0.150	0.125	2.33E-01	0.071	0.038	6.30E-02	-0.062	0.125	6.19E-01	-0.010	0.039	7.90E-01	-0.009	0.075	9.01E-01
rs9263708	6	31203249	G/A	*PSORS1C1*	INTRON	2	2.495	2.122	2.934	**1.98E-28**	-	-	-	-0.088	0.125	4.81E-01	-	-	-	-0.199	0.122	1.08E-01	-	-	-	0.015	0.075	8.43E-01
rs9275659	6	32794081	A/C	*HLA-DQB1 | HLA-DQA2*	INTERGENIC	2	2.495	2.121	2.933	**1.98E-28**	0.021	0.039	5.80E-01	-0.031	0.123	8.04E-01	0.050	0.038	2.00E-01	-0.180	0.121	1.40E-01	-0.032	0.039	4.10E-01	0.075	0.074	3.09E-01
rs3134944	6	32254622	A/G	*RNF5*	INTRON	2	2.563	2.169	3.028	**2.00E-28**	-	-	-	-0.150	0.125	2.33E-01	-	-	-	-0.062	0.125	6.19E-01	-	-	-	-0.009	0.075	9.01E-01
rs3130347	6	32242634	G/A	*EGFL8*	INTRON	2	2.563	2.169	3.029	**2.02E-28**	-	-	-	-0.150	0.125	2.33E-01	-	-	-	-0.062	0.125	6.19E-01	-	-	-	-0.009	0.075	9.01E-01
rs3131297	6	32248983	T/A	*AGPAT1*	INTRON	2	2.563	2.169	3.028	**2.03E-28**	0.065	0.038	9.20E-02	-0.150	0.125	2.33E-01	0.072	0.038	5.90E-02	-0.062	0.125	6.19E-01	-0.012	0.038	7.50E-01	-0.009	0.075	9.01E-01
rs2507997	6	31422760	G/A	*HLA-C | HLA-B*	INTERGENIC	2	2.503	2.127	2.946	**2.60E-28**	-	-	-	-0.174	0.123	1.63E-01	-	-	-	-0.214	0.122	8.24E-02	-	-	-	-0.040	0.075	5.97E-01
rs11961853	6	30473608	A/G	*RPP21 | HLA-E*	INTERGENIC	2	2.659	2.235	3.165	**2.98E-28**	0.026	0.043	5.50E-01	-0.025	0.124	8.41E-01	0.212	0.042	**5.50E-07**	-0.146	0.122	2.34E-01	-0.200	0.043	**3.10E-06**	0.002	0.074	9.83E-01
rs3134952	6	32221549	A/T	*FKBPL | PRRT1*	INTERGENIC	2	2.570	2.173	3.040	**3.18E-28**	0.062	0.038	1.03E-01	-0.150	0.125	2.33E-01	0.086	0.038	**2.30E-02**	-0.062	0.125	6.19E-01	-0.027	0.038	4.90E-01	-0.004	0.076	9.59E-01
rs9276555	6	32830732	A/G	*HLA-DQA2 | HLA-DQB2*	INTERGENIC	2	2.531	2.145	2.986	**3.48E-28**	0.017	0.039	6.70E-01	-0.042	0.123	7.35E-01	0.055	0.038	1.50E-01	-0.166	0.121	1.75E-01	-0.042	0.039	2.80E-01	0.099	0.074	1.82E-01
rs3130781	6	31022531	G/A	*LOC100129065 | DPCR1*	INTERGENIC	2	2.510	2.131	2.957	**3.50E-28**	-0.015	0.038	6.90E-01	-0.078	0.123	5.25E-01	0.106	0.038	**5.10E-03**	-0.143	0.121	2.41E-01	-0.126	0.038	**1.04E-03**	0.011	0.074	8.78E-01
rs2844777	6	30313993	A/G	*TRIM26 | FLJ45422*	INTERGENIC	2	2.899	2.397	3.505	**4.57E-28**	0.042	0.047	3.70E-01	0.021	0.125	8.70E-01	0.288	0.047	**6.50E-10**	-0.152	0.122	2.19E-01	-0.271	0.047	**1.00E-08**	-0.048	0.075	5.20E-01
rs3129763	6	32698903	A/G	*HLA-DRB1 | HLA-DQA1*	INTERGENIC	2	2.514	2.132	2.963	**4.61E-28**	0.072	0.039	6.10E-02	-0.081	0.126	5.24E-01	0.153	0.038	**6.20E-05**	-0.042	0.126	7.40E-01	-0.102	0.038	**7.80E-03**	0.019	0.075	8.06E-01
rs9268220	6	32392318	A/G	*C6orf10*	INTRON	1	2.860	2.370	3.451	**5.30E-28**	0.103	0.041	**1.23E-02**	-0.104	0.122	3.99E-01	0.126	0.041	**2.10E-03**	-0.219	0.119	7.13E-02	-0.047	0.041	2.50E-01	0.035	0.074	6.34E-01
rs9276607	6	32845235	A/C	*HLA-DQB2 | HLA-DOB*	INTERGENIC	2	2.513	2.131	2.963	**5.62E-28**	-	-	-	-0.042	0.123	7.35E-01	-	-	-	-0.166	0.121	1.75E-01	-	-	-	0.093	0.074	2.09E-01
rs9261290	6	30146626	G/A	*PPP1R11 / RNF39*	UTR	2	2.924	2.413	3.542	**5.89E-28**	0.029	0.048	5.40E-01	0.035	0.124	7.81E-01	0.295	0.047	**3.80E-10**	-0.133	0.122	2.82E-01	-0.288	0.048	**1.50E-09**	-0.042	0.075	5.75E-01
rs3130284	6	32248465	G/A	*AGPAT1*	INTRON	2	2.560	2.164	3.030	**7.21E-28**	0.065	0.038	9.20E-02	-0.150	0.125	2.33E-01	0.072	0.038	5.90E-02	-0.062	0.125	6.19E-01	-0.012	0.038	7.50E-01	-0.009	0.075	9.01E-01
rs3129890	6	32522251	G/A	*HLA-DRA | HLA-DRB5*	INTERGENIC	2	2.372	2.031	2.769	**8.46E-28**	-	-	-	-0.022	0.123	8.57E-01	-	-	-	-0.195	0.120	1.09E-01	-	-	-	0.005	0.075	9.50E-01
rs3134947	6	32253183	A/G	*AGPAT1*	INTRON	2	2.547	2.153	3.012	**9.51E-28**	0.065	0.038	9.20E-02	-0.172	0.124	1.69E-01	0.072	0.038	5.90E-02	-0.055	0.125	6.59E-01	-0.012	0.038	7.50E-01	-0.013	0.075	8.62E-01
rs3131787	6	31007503	G/A	*SFTPG*	CODING	2	2.517	2.132	2.971	**9.88E-28**	-0.014	0.038	7.10E-01	-0.078	0.123	5.25E-01	0.106	0.038	**5.00E-03**	-0.143	0.121	2.41E-01	-0.125	0.038	**1.10E-03**	0.017	0.074	8.21E-01
rs1264706	6	30171631	G/C	*RNF39 | TRIM31*	INTERGENIC	2	2.915	2.405	3.533	**1.16E-27**	-	-	-	0.035	0.124	7.81E-01	-	-	-	-0.133	0.122	2.82E-01	-	-	-	-0.042	0.075	5.75E-01
rs3095329	6	30801795	G/A	*TUBB | FLOT1*	INTERGENIC	2	2.555	2.159	3.025	**1.16E-27**	0.022	0.038	5.60E-01	0.002	0.123	9.85E-01	0.152	0.038	**5.20E-05**	-0.055	0.122	6.55E-01	-0.133	0.038	**4.20E-04**	-0.021	0.074	7.75E-01

rs4678	6	31001920	A/G	*VARS2*	CODING	2	2.514	2.130	2.967	**1.16E-27**	-0.014	0.038	7.10E-01	-0.078	0.123	5.25E-01	0.106	0.038	**5.00E-03**	-0.143	0.121	2.41E-01	-0.125	0.038	**1.10E-03**	0.017	0.074	8.21E-01
rs1059612	6	30816934	A/G	*FLOT1*	INTRON	1	3.240	2.622	4.003	**1.27E-27**	0.031	0.044	4.80E-01	-0.016	0.124	8.98E-01	0.249	0.043	**9.10E-09**	-0.172	0.122	1.63E-01	-0.239	0.044	**4.90E-08**	0.026	0.075	7.29E-01
rs3129943	6	32446673	G/A	*C6orf10*	INTRON	2	2.394	2.046	2.802	**1.38E-27**	0.079	0.037	**3.20E-02**	-0.085	0.123	4.91E-01	0.073	0.037	**4.60E-02**	-0.165	0.121	1.77E-01	-0.004	0.037	9.10E-01	-0.010	0.074	8.90E-01
rs9276584	6	32838813	A/G	*HLA-DQB2*	INTRON	2	2.496	2.117	2.944	**1.53E-27**	0.019	0.039	6.30E-01	-0.042	0.123	7.35E-01	0.065	0.039	9.40E-02	-0.166	0.121	1.75E-01	-0.052	0.039	1.80E-01	0.095	0.074	2.00E-01
rs3132584	6	30796406	A/C	*TUBB*	INTRON	2	2.548	2.152	3.016	**1.70E-27**	0.022	0.038	5.60E-01	0.002	0.123	9.85E-01	0.152	0.038	**5.20E-05**	-0.055	0.122	6.55E-01	-0.133	0.038	**4.20E-04**	-0.021	0.074	7.75E-01
rs3117099	6	32466248	A/G	*C6orf10 | BTNL2*	INTERGENIC	2	2.383	2.037	2.788	**1.89E-27**	0.048	0.038	2.10E-01	0.011	0.123	9.27E-01	0.019	0.038	6.10E-01	-0.151	0.121	2.16E-01	0.022	0.038	5.60E-01	0.013	0.075	8.67E-01
rs3132550	6	31194027	A/G	*CDSN*	INTRON	2	2.445	2.080	2.875	**2.56E-27**	0.011	0.036	7.60E-01	-0.054	0.125	6.67E-01	0.090	0.035	**1.05E-02**	-0.187	0.122	1.30E-01	-0.078	0.035	**2.80E-02**	0.014	0.075	8.47E-01
rs2853928	6	31365490	A/C	*HLA-C | HLA-B*	INTERGENIC	2	2.427	2.067	2.850	**2.88E-27**	-0.048	0.033	1.60E-01	0.036	0.124	7.74E-01	0.294	0.033	**8.00E-19**	-0.161	0.122	1.93E-01	-0.359	0.033	**6.80E-27**	0.025	0.076	7.43E-01
rs2523544	6	31441541	A/G	*HLA-B | LOC729816*	INTERGENIC	2	2.381	2.034	2.786	**2.94E-27**	0.064	0.039	1.02E-01	0.012	0.124	9.22E-01	0.197	0.039	**3.80E-07**	-0.046	0.123	7.12E-01	-0.149	0.039	**1.50E-04**	-0.001	0.075	9.94E-01
rs3094216	6	31192027	G/A	*CDSN*	CODING	2	2.443	2.078	2.872	**2.94E-27**	0.011	0.036	7.60E-01	-0.054	0.125	6.67E-01	0.090	0.035	**1.05E-02**	-0.187	0.122	1.30E-01	-0.078	0.035	**2.80E-02**	0.014	0.075	8.47E-01
rs2247056	6	31373469	A/G	*HLA-C | HLA-B*	INTERGENIC	2	2.415	2.058	2.833	**3.46E-27**	-0.046	0.034	1.70E-01	0.036	0.124	7.74E-01	0.293	0.033	**1.00E-18**	-0.161	0.122	1.93E-01	-0.357	0.033	**1.10E-26**	0.025	0.076	7.43E-01
rs3094220	6	31190966	G/A	*CDSN*	UTR	2	2.442	2.076	2.872	**4.52E-27**	0.011	0.036	7.60E-01	-0.054	0.125	6.67E-01	0.090	0.035	**1.05E-02**	-0.187	0.122	1.30E-01	-0.078	0.035	**2.80E-02**	0.014	0.075	8.47E-01
rs3095336	6	30846425	A/G	*IER3 | DDR1*	INTERGENIC	1	3.130	2.542	3.854	**5.65E-27**	0.023	0.044	6.00E-01	-0.004	0.124	9.76E-01	0.237	0.044	**6.60E-08**	-0.155	0.122	2.07E-01	-0.229	0.044	**2.30E-07**	0.011	0.074	8.80E-01
rs3095298	6	31190911	A/G	*CDSN*	UTR	2	2.437	2.072	2.867	**5.87E-27**	-	-	-	-0.040	0.126	7.50E-01	-	-	-	-0.174	0.124	1.66E-01	-	-	-	0.013	0.075	8.59E-01
rs9500927	6	33069339	A/G	*BRD2 | HLA-DOA*	INTERGENIC	2	2.669	2.231	3.192	**5.95E-27**	0.047	0.044	2.90E-01	0.002	0.123	9.88E-01	0.084	0.044	5.50E-02	0.020	0.123	8.74E-01	-0.047	0.044	2.80E-01	0.059	0.074	4.24E-01
rs642093	6	32690053	A/G	*HLA-DRB1 | HLA-DQA1*	INTERGENIC	2	2.402	2.047	2.820	**8.38E-27**	-	-	-	-0.020	0.127	8.76E-01	-	-	-	0.027	0.126	8.28E-01	-	-	-	0.020	0.075	7.89E-01
rs7758736	6	32866372	A/G	*HLA-DQB2 | HLA-DOB*	INTERGENIC	2	2.510	2.121	2.970	**9.53E-27**	0.070	0.042	9.50E-02	-0.105	0.122	3.95E-01	0.066	0.041	1.11E-01	-0.241	0.119	**4.63E-02**	-0.009	0.042	8.20E-01	0.093	0.074	2.06E-01
rs204995	6	32262263	G/A	*PBX2*	INTRON	2	2.442	2.074	2.876	**1.05E-26**	0.132	0.038	**4.90E-04**	-0.028	0.124	8.23E-01	0.059	0.038	1.13E-01	-0.103	0.123	4.07E-01	0.049	0.038	2.00E-01	0.006	0.075	9.37E-01
rs2524054	6	31360375	A/C	*HLA-C | HLA-B*	INTERGENIC	2	2.412	2.052	2.836	**1.36E-26**	-0.037	0.033	2.60E-01	0.036	0.124	7.74E-01	0.310	0.033	**6.20E-21**	-0.161	0.122	1.93E-01	-0.368	0.033	**2.10E-28**	0.025	0.076	7.43E-01
rs3135363	6	32497626	G/A	*BTNL2 | HLA-DRA*	INTERGENIC	2	2.314	1.983	2.700	**1.52E-26**	0.086	0.037	**1.90E-02**	-0.107	0.122	3.85E-01	0.077	0.036	**3.40E-02**	-0.152	0.121	2.13E-01	-0.012	0.036	7.40E-01	0.093	0.074	2.11E-01
rs9268861	6	32537872	A/C	*HLA-DRA | HLA-DRB5*	INTERGENIC	1	2.600	2.180	3.100	**1.97E-26**	0.044	0.039	2.60E-01	-0.158	0.122	2.02E-01	0.083	0.039	**3.20E-02**	-0.230	0.120	5.94E-02	-0.049	0.039	2.10E-01	-0.024	0.074	7.49E-01
rs204994	6	32262976	A/G	*PBX2*	INTRON	2	2.444	2.071	2.883	**3.27E-26**	0.132	0.038	**4.90E-04**	-0.028	0.124	8.23E-01	0.059	0.038	1.13E-01	-0.103	0.123	4.07E-01	0.049	0.038	2.00E-01	0.006	0.075	9.37E-01
rs3094600	6	31455123	A/G	*LOC729816 | LOC100129668*	INTERGENIC	2	2.330	1.992	2.725	**3.56E-26**	-0.011	0.036	7.60E-01	0.023	0.123	8.53E-01	0.137	0.036	**1.40E-04**	-0.128	0.121	2.94E-01	-0.159	0.036	**1.30E-05**	0.010	0.075	8.91E-01
rs3094049	6	30467339	A/G	*RPP21 | HLA-E*	INTERGENIC	1	3.170	2.558	3.928	**5.01E-26**	-	-	-	0.033	0.125	7.89E-01	-	-	-	-0.151	0.122	2.21E-01	-	-	-	0.023	0.075	7.58E-01
rs9268197	6	32385912	A/C	*C6orf10*	INTRON	2	2.328	1.990	2.724	**5.24E-26**	0.109	0.038	**4.40E-03**	-0.108	0.122	3.82E-01	0.116	0.038	**2.30E-03**	-0.214	0.119	7.73E-02	-0.031	0.038	4.20E-01	0.012	0.074	8.75E-01
rs5021728	6	32686631	G/A	*HLA-DRB1 | HLA-DQA1*	INTERGENIC	2	2.369	2.018	2.781	**5.28E-26**	-	-	-	-0.044	0.127	7.28E-01	-	-	-	0.007	0.126	9.54E-01	-	-	-	-0.002	0.076	9.78E-01
rs176095	6	32266297	G/A	*PBX2 | GPSM3*	INTERGENIC	2	2.443	2.069	2.885	**5.91E-26**	0.132	0.038	**4.90E-04**	-0.028	0.124	8.23E-01	0.059	0.038	1.13E-01	-0.103	0.123	4.07E-01	0.049	0.038	2.00E-01	0.006	0.075	9.37E-01
rs3130117	6	30616935	A/G	*HLA-E | GNL1*	INTERGENIC	1	3.070	2.492	3.783	**6.08E-26**	-0.008	0.045	8.50E-01	-0.012	0.125	9.21E-01	0.247	0.044	**2.80E-08**	-0.166	0.122	1.80E-01	-0.268	0.045	**2.80E-09**	0.024	0.075	7.52E-01
rs424232	6	32316302	A/G	*NOTCH4 | C6orf10*	INTERGENIC	2	2.358	2.010	2.767	**7.35E-26**	0.035	0.036	3.30E-01	-0.030	0.124	8.10E-01	0.062	0.035	8.10E-02	-0.079	0.123	5.23E-01	-0.037	0.036	3.00E-01	0.057	0.075	4.45E-01
rs3130782	6	31022822	A/G	*LOC100129065 | DPCR1*	INTERGENIC	2	2.661	2.217	3.195	**7.84E-26**	-0.006	0.044	9.00E-01	-0.021	0.124	8.69E-01	0.218	0.043	**4.30E-07**	-0.160	0.122	1.93E-01	-0.235	0.044	**7.20E-08**	0.005	0.074	9.45E-01
rs2442749	6	31460019	G/A	*LOC729816 | LOC100129668*	INTERGENIC	2	2.292	1.963	2.676	**8.79E-26**	-0.009	0.036	8.00E-01	-0.058	0.124	6.40E-01	0.092	0.036	**9.80E-03**	-0.102	0.122	4.07E-01	-0.106	0.036	**3.30E-03**	0.017	0.075	8.17E-01
rs652888	6	31959213	G/A	*EHMT2*	INTRON	2	2.341	1.996	2.745	**1.11E-25**	0.104	0.040	**9.00E-03**	-0.155	0.124	2.17E-01	0.143	0.039	**2.60E-04**	-0.124	0.124	3.18E-01	-0.057	0.040	1.47E-01	-0.039	0.075	6.08E-01
rs9257809	6	29464310	G/A	*OR12D3 | OR12D2*	INTERGENIC	2	2.819	2.322	3.423	**1.12E-25**	0.027	0.047	5.70E-01	0.035	0.124	7.81E-01	0.227	0.046	**8.50E-07**	-0.133	0.122	2.82E-01	-0.210	0.047	**7.20E-06**	-0.035	0.074	6.35E-01
rs204992	6	32264886	A/G	*PBX2*	INTRON	2	2.433	2.059	2.874	**1.41E-25**	-	-	-	-0.028	0.124	8.23E-01	-	-	-	-0.103	0.123	4.07E-01	-	-	-	0.006	0.075	9.37E-01
rs1535039	6	29519411	G/A	*OR10C1 | OR2H1*	INTERGENIC	2	2.793	2.303	3.388	**1.82E-25**	0.000	0.046	1.00E+00	0.035	0.124	7.81E-01	0.209	0.046	**5.50E-06**	-0.133	0.122	2.82E-01	-0.213	0.047	**4.90E-06**	-0.027	0.074	7.13E-01
rs442694	6	29464666	A/C	*OR12D3 | OR12D2*	INTERGENIC	2	2.793	2.303	3.388	**1.82E-25**	0.027	0.047	5.70E-01	0.035	0.124	7.81E-01	0.227	0.046	**8.50E-07**	-0.133	0.122	2.82E-01	-0.210	0.047	**7.20E-06**	-0.027	0.074	7.13E-01
rs2746150	6	29550680	A/G	*OR2H1 | MAS1L*	INTERGENIC	2	2.805	2.310	3.405	**2.08E-25**	0.035	0.049	4.70E-01	0.035	0.124	7.81E-01	0.259	0.048	**9.10E-08**	-0.133	0.122	2.82E-01	-0.240	0.049	**1.00E-06**	-0.027	0.074	7.13E-01
rs416352	6	32315371	A/C	*NOTCH4 | C6orf10*	INTERGENIC	2	2.281	1.952	2.666	**3.16E-25**	0.073	0.035	**3.80E-02**	0.017	0.123	8.88E-01	0.030	0.035	3.90E-01	-0.143	0.121	2.43E-01	0.032	0.035	3.70E-01	0.057	0.074	4.46E-01
rs521828	6	32399621	A/G	*C6orf10 | LOC100131609*	INTERGENIC	2	2.275	1.948	2.657	**3.21E-25**	0.074	0.036	**3.90E-02**	-0.085	0.123	4.91E-01	0.072	0.036	**4.30E-02**	-0.165	0.121	1.77E-01	-0.009	0.036	8.00E-01	-0.022	0.074	7.71E-01
rs9257800	6	29448722	A/G	*OR5V1 | OR12D3*	INTERGENIC	2	2.770	2.284	3.359	**4.04E-25**	0.036	0.049	4.70E-01	0.052	0.124	6.76E-01	0.252	0.049	**2.40E-07**	-0.124	0.123	3.17E-01	-0.238	0.049	**1.40E-06**	-0.025	0.075	7.36E-01
rs1233491	6	29569709	C/G	*MAS1L | LOC729653*	INTERGENIC	2	2.795	2.301	3.395	**4.12E-25**	0.027	0.048	5.70E-01	0.035	0.124	7.81E-01	0.249	0.047	**1.30E-07**	-0.133	0.122	2.82E-01	-0.237	0.048	**6.90E-07**	-0.027	0.074	7.13E-01
rs1233489	6	29571022	A/T	*MAS1L | LOC729653*	INTERGENIC	2	2.786	2.295	3.383	**4.39E-25**	0.027	0.048	5.70E-01	0.035	0.124	7.81E-01	0.249	0.047	**1.30E-07**	-0.133	0.122	2.82E-01	-0.237	0.048	**6.90E-07**	-0.027	0.074	7.13E-01
rs2858324	6	32768353	A/G	*HLA-DQB1 | HLA-DQA2*	INTERGENIC	2	2.271	1.944	2.654	**5.78E-25**	-	-	-	-0.104	0.122	3.99E-01	-	-	-	-0.280	0.117	**1.99E-02**	-	-	-	0.050	0.074	4.98E-01
rs429479	6	29480302	G/A	*OR12D2 | OR11A1*	INTERGENIC	2	2.775	2.285	3.369	**6.34E-25**	0.024	0.046	6.10E-01	0.035	0.124	7.81E-01	0.223	0.046	**1.30E-06**	-0.133	0.122	2.82E-01	-0.208	0.047	**8.30E-06**	-0.035	0.075	6.37E-01
rs9262141	6	30752116	G/A	*DHX16 | KIAA1949*	INTERGENIC	1	2.860	2.342	3.493	**6.73E-25**	0.011	0.046	8.00E-01	-0.036	0.125	7.73E-01	0.273	0.045	**1.30E-09**	-0.174	0.122	1.61E-01	-0.279	0.046	**1.00E-09**	-0.058	0.075	4.45E-01
rs2856717	6	32778286	A/G	*HLA-DQB1 | HLA-DQA2*	INTERGENIC	2	2.275	1.946	2.661	**7.38E-25**	-	-	-	-0.104	0.122	3.99E-01	-	-	-	-0.280	0.117	**1.99E-02**	-	-	-	0.050	0.074	4.98E-01
rs1265181	6	31263764	G/C	*LOC100130889 | HCG27*	INTERGENIC	2	2.424	2.048	2.870	**7.86E-25**	0.028	0.037	4.50E-01	-0.023	0.124	8.55E-01	0.122	0.037	**9.30E-04**	-0.157	0.122	2.02E-01	-0.103	0.037	**5.30E-03**	0.144	0.073	5.08E-02
rs9268856	6	32537697	A/C	*HLA-DRA | HLA-DRB5*	INTERGENIC	1	2.480	2.086	2.948	**7.91E-25**	0.062	0.038	1.02E-01	-0.069	0.123	5.76E-01	0.035	0.037	3.50E-01	-0.195	0.120	1.08E-01	0.018	0.038	6.40E-01	-0.030	0.075	6.87E-01
rs1265178	6	31269208	A/G	*LOC100130889 | HCG27*	INTERGENIC	2	2.424	2.048	2.869	**8.02E-25**	0.028	0.037	4.50E-01	-0.023	0.124	8.55E-01	0.122	0.037	**9.30E-04**	-0.157	0.122	2.02E-01	-0.103	0.037	**5.30E-03**	0.144	0.073	5.08E-02
rs404240	6	29631936	G/A	*UBD*	CODING	2	2.767	2.279	3.360	**9.03E-25**	0.024	0.048	6.20E-01	0.035	0.124	7.81E-01	0.262	0.047	**3.50E-08**	-0.133	0.122	2.82E-01	-0.255	0.048	**1.10E-07**	-0.027	0.074	7.13E-01
rs3094712	6	30459327	G/A	*RPP21 | HLA-E*	INTERGENIC	1	3.160	2.537	3.936	**9.28E-25**	0.021	0.047	6.60E-01	0.059	0.124	6.38E-01	0.274	0.047	**3.80E-09**	-0.145	0.122	2.40E-01	-0.270	0.047	**1.10E-08**	0.041	0.075	5.80E-01
rs2647012	6	32772436	A/G	*HLA-DQB1 | HLA-DQA2*	INTERGENIC	2	2.279	1.947	2.666	**9.41E-25**	-0.106	0.032	**9.20E-04**	-0.104	0.122	3.99E-01	0.082	0.032	**9.40E-03**	-0.280	0.117	**1.99E-02**	-0.183	0.032	**9.50E-09**	0.050	0.074	4.98E-01
rs2395177	6	32513054	C/G	*BTNL2 | HLA-DRA*	INTERGENIC	1	2.460	2.071	2.922	**1.11E-24**	0.024	0.036	5.10E-01	-0.065	0.123	5.98E-01	0.034	0.035	3.30E-01	-0.228	0.119	5.91E-02	-0.023	0.036	5.10E-01	0.036	0.074	6.29E-01
rs1233480	6	29585393	A/G	*LOC729653 | UBD*	INTERGENIC	2	2.720	2.246	3.293	**1.19E-24**	0.027	0.048	5.80E-01	0.032	0.125	8.01E-01	0.251	0.047	**1.00E-07**	-0.143	0.123	2.48E-01	-0.241	0.048	**4.90E-07**	-0.036	0.075	6.32E-01
rs1612904	6	32776996	C/A	*HLA-DQB1 | HLA-DQA2*	INTERGENIC	2	2.270	1.940	2.656	**1.26E-24**	-	-	-	-0.088	0.123	4.73E-01	-	-	-	-0.261	0.118	**3.06E-02**	-	-	-	0.061	0.074	4.07E-01
rs3134745	6	31350741	A/G	*HLA-C | HLA-B*	INTERGENIC	2	2.276	1.943	2.666	**2.12E-24**	-	-	-	-0.097	0.124	4.36E-01	-	-	-	-0.180	0.121	1.43E-01	-	-	-	-0.043	0.075	5.65E-01
rs9268212	6	32389867	G/A	*C6orf10*	INTRON	2	2.251	1.926	2.632	**2.23E-24**	-	-	-	-0.108	0.122	3.82E-01	-	-	-	-0.214	0.119	7.73E-02	-	-	-	0.012	0.074	8.75E-01
rs204999	6	32217957	G/A	*FKBPL | PRRT1*	INTERGENIC	2	2.328	1.979	2.739	**2.37E-24**	0.091	0.036	**1.13E-02**	-0.119	0.124	3.42E-01	0.042	0.036	2.40E-01	0.014	0.124	9.10E-01	0.043	0.036	2.40E-01	0.051	0.075	4.95E-01
rs9275596	6	32789609	G/A	*HLA-DQB1 | HLA-DQA2*	INTERGENIC	2	2.262	1.932	2.648	**2.90E-24**	-0.105	0.032	**1.22E-03**	-0.088	0.123	4.73E-01	0.104	0.032	**1.15E-03**	-0.261	0.118	**3.06E-02**	-0.205	0.032	**2.30E-10**	0.061	0.074	4.07E-01
rs2746149	6	29543334	G/A	*OR2H1 | MAS1L*	INTERGENIC	2	2.727	2.246	3.311	**3.69E-24**	0.013	0.047	7.70E-01	0.035	0.124	7.81E-01	0.220	0.046	**2.10E-06**	-0.133	0.122	2.82E-01	-0.212	0.047	**6.10E-06**	-0.027	0.074	7.13E-01
rs130079	6	31220716	A/C	*CCHCR1*	CODING	2	2.355	1.996	2.779	**3.75E-24**	0.007	0.037	8.40E-01	-0.034	0.123	7.82E-01	0.113	0.036	**1.80E-03**	-0.150	0.121	2.22E-01	-0.111	0.037	**2.30E-03**	0.113	0.074	1.26E-01
rs929157	6	30245188	A/G	*TRIM15*	INTRON	2	2.557	2.132	3.067	**4.36E-24**	0.007	0.042	8.60E-01	-0.027	0.125	8.29E-01	0.201	0.042	**1.60E-06**	-0.150	0.123	2.26E-01	-0.199	0.042	**2.70E-06**	-0.068	0.075	3.63E-01
rs3132685	6	30053928	A/G	*HCG9*	INTRON	2	2.640	2.187	3.186	**4.66E-24**	0.021	0.048	6.60E-01	0.035	0.124	7.81E-01	0.291	0.047	**6.70E-10**	-0.133	0.122	2.82E-01	-0.292	0.048	**1.00E-09**	-0.037	0.075	6.25E-01
rs9268202	6	32387318	A/G	*C6orf10*	INTRON	2	2.244	1.919	2.625	**4.89E-24**	0.109	0.038	**4.40E-03**	-0.108	0.122	3.82E-01	0.116	0.038	**2.30E-03**	-0.214	0.119	7.73E-02	-0.031	0.038	4.20E-01	0.012	0.074	8.75E-01
rs3130457	6	31255173	G/A	*LOC100130889 | HCG27*	INTERGENIC	2	2.348	1.990	2.771	**4.99E-24**	0.029	0.037	4.30E-01	-0.033	0.124	7.92E-01	0.124	0.037	**7.30E-04**	-0.174	0.121	1.55E-01	-0.105	0.037	**4.40E-03**	0.127	0.074	8.58E-02
rs1634719	6	31081144	G/A	*C6orf205 | LOC729792*	INTERGENIC	2	2.237	1.913	2.614	**5.16E-24**	-0.010	0.035	7.60E-01	-0.100	0.122	4.15E-01	0.081	0.034	**1.80E-02**	0.095	0.122	4.40E-01	-0.089	0.035	**1.05E-02**	-0.037	0.074	6.18E-01
rs6934776	6	32387794	A/G	*C6orf10*	INTRON	2	2.243	1.918	2.624	**5.24E-24**	-	-	-	-0.108	0.122	3.82E-01	-	-	-	-0.214	0.119	7.73E-02	-	-	-	0.012	0.074	8.75E-01
rs3094120	6	30839535	A/G	*IER3 | DDR1*	INTERGENIC	2	2.304	1.959	2.708	**5.37E-24**	-	-	-	0.096	0.122	4.37E-01	-	-	-	-0.048	0.122	6.94E-01	-	-	-	0.046	0.074	5.39E-01
rs3094121	6	30838939	A/G	*IER3 | DDR1*	INTERGENIC	2	2.303	1.959	2.708	**5.43E-24**	-0.006	0.037	8.60E-01	0.096	0.122	4.37E-01	0.154	0.037	**3.10E-05**	-0.048	0.122	6.94E-01	-0.162	0.037	**1.30E-05**	0.046	0.074	5.39E-01

rs1235162	6	29645203	G/A	*UBD | SNORD32B*	INTERGENIC	2	2.702	2.228	3.277	**5.52E-24**	0.036	0.048	4.60E-01	0.117	0.123	3.44E-01	0.284	0.048	**2.40E-09**	-0.044	0.123	7.21E-01	-0.269	0.048	**2.40E-08**	-0.037	0.075	6.17E-01
rs2517645	6	30230602	G/A	*TRIM10*	INTRON	2	2.480	2.079	2.959	**5.64E-24**	0.017	0.042	6.80E-01	-0.002	0.126	9.89E-01	0.186	0.041	**6.40E-06**	-0.139	0.124	2.64E-01	-0.172	0.042	**3.50E-05**	0.013	0.074	8.66E-01
rs886403	6	31065597	G/A	*C6orf205*	UTR	2	2.228	1.907	2.603	**5.67E-24**	-0.001	0.035	9.70E-01	-0.094	0.122	4.44E-01	0.114	0.035	**1.16E-03**	-0.010	0.122	9.38E-01	-0.120	0.035	**7.50E-04**	-0.024	0.074	7.45E-01
rs3094116	6	30846387	T/A	*IER3 | DDR1*	INTERGENIC	2	2.244	1.918	2.626	**6.83E-24**	-0.019	0.036	6.00E-01	0.014	0.123	9.07E-01	0.137	0.035	**1.00E-04**	0.048	0.122	6.97E-01	-0.159	0.036	**8.90E-06**	-0.008	0.075	9.11E-01
rs9276556	6	32831211	G/C	*HLA-DQA2 | HLA-DQB2*	INTERGENIC	1	2.570	2.139	3.088	**7.35E-24**	0.014	0.039	7.20E-01	-0.042	0.123	7.35E-01	0.054	0.038	1.60E-01	-0.166	0.121	1.75E-01	-0.043	0.039	2.60E-01	0.096	0.074	1.96E-01
rs3117425	6	29368410	A/G	*LOC651503 | OR5U1*	INTERGENIC	2	2.683	2.214	3.251	**7.51E-24**	0.035	0.049	4.70E-01	0.059	0.124	6.37E-01	0.244	0.048	**4.30E-07**	-0.115	0.123	3.51E-01	-0.227	0.049	**3.00E-06**	-0.016	0.074	8.26E-01
rs1150765	6	31235541	A/G	*TCF19*	INTRON	2	2.329	1.976	2.746	**8.02E-24**	0.010	0.037	7.90E-01	-0.034	0.123	7.82E-01	0.117	0.036	**1.16E-03**	-0.150	0.121	2.22E-01	-0.114	0.036	**1.80E-03**	0.113	0.074	1.26E-01
rs3864299	6	32379652	T/A	*C6orf10*	INTRON	2	2.228	1.906	2.604	**8.11E-24**	-	-	-	-0.108	0.122	3.82E-01	-	-	-	-0.214	0.119	7.73E-02	-	-	-	0.012	0.074	8.75E-01
rs1018430	6	32389666	A/G	*C6orf10*	INTRON	2	2.219	1.900	2.592	**8.34E-24**	0.109	0.038	**4.40E-03**	-0.108	0.122	3.82E-01	0.116	0.038	**2.30E-03**	-0.214	0.119	7.73E-02	-0.031	0.038	4.20E-01	0.013	0.074	8.65E-01
rs3130455	6	31233957	T/A	*CCHCR1*	UTR	2	2.394	2.019	2.838	**8.60E-24**	0.016	0.037	6.60E-01	-0.024	0.124	8.46E-01	0.125	0.037	**6.40E-04**	-0.129	0.122	2.92E-01	-0.115	0.037	**1.80E-03**	0.130	0.073	7.91E-02
rs9268125	6	32360656	G/A	*NOTCH4 | C6orf10*	INTERGENIC	2	2.225	1.904	2.600	**8.70E-24**	-	-	-	-0.108	0.122	3.82E-01	-	-	-	-0.214	0.119	7.73E-02	-	-	-	0.012	0.074	8.75E-01
rs7750783	6	32376058	A/G	*C6orf10*	INTRON	2	2.225	1.904	2.600	**8.79E-24**	0.091	0.038	**1.49E-02**	-0.108	0.122	3.82E-01	0.109	0.037	**3.50E-03**	-0.214	0.119	7.73E-02	-0.039	0.037	2.90E-01	0.010	0.074	8.98E-01
rs3117439	6	29374462	A/G	*LOC651503 | OR5U1*	INTERGENIC	2	2.705	2.228	3.285	**9.72E-24**	0.035	0.049	4.70E-01	0.059	0.124	6.37E-01	0.244	0.048	**4.30E-07**	-0.115	0.123	3.51E-01	-0.227	0.049	**3.00E-06**	-0.018	0.075	8.15E-01
rs3094122	6	30836339	C/A	*IER3 | DDR1*	INTERGENIC	2	2.275	1.938	2.671	**9.90E-24**	-0.015	0.036	6.70E-01	0.104	0.122	3.99E-01	0.142	0.036	**6.80E-05**	0.093	0.122	4.50E-01	-0.160	0.036	**8.70E-06**	0.021	0.074	7.77E-01
rs2239529	6	30186309	A/G	*TRIM31*	CODING	2	2.414	2.032	2.867	**1.06E-23**	0.035	0.042	4.10E-01	0.002	0.123	9.89E-01	0.231	0.042	**3.10E-08**	-0.134	0.121	2.73E-01	-0.214	0.042	**3.80E-07**	0.051	0.074	4.92E-01
rs3118357	6	28910128	A/G	*LOC442181 | LOC401242*	INTERGENIC	2	2.734	2.246	3.327	**1.10E-23**	0.038	0.051	4.50E-01	0.037	0.124	7.66E-01	0.268	0.050	**8.50E-08**	-0.118	0.122	3.38E-01	-0.250	0.051	**7.90E-07**	-0.016	0.074	8.32E-01
rs9268176	6	32382057	A/G	*C6orf10*	INTRON	2	2.236	1.910	2.617	**1.17E-23**	0.101	0.038	**8.30E-03**	-0.108	0.122	3.82E-01	0.110	0.038	**3.80E-03**	-0.214	0.119	7.73E-02	-0.031	0.038	4.20E-01	0.012	0.074	8.75E-01
rs7775332	6	32378341	A/T	*C6orf10*	INTRON	2	2.238	1.912	2.620	**1.19E-23**	0.111	0.038	**3.80E-03**	-0.108	0.122	3.82E-01	0.114	0.038	**2.70E-03**	-0.214	0.119	7.73E-02	-0.027	0.038	4.80E-01	0.012	0.075	8.74E-01
rs3096674	6	32346197	A/G	*NOTCH4 | C6orf10*	INTERGENIC	2	2.245	1.916	2.629	**1.20E-23**	-	-	-	-0.108	0.122	3.82E-01	-	-	-	-0.214	0.119	7.73E-02	-	-	-	0.012	0.074	8.75E-01
rs3132931	6	32343873	C/A	*NOTCH4 | C6orf10*	INTERGENIC	2	2.245	1.916	2.629	**1.20E-23**	0.094	0.038	**1.24E-02**	-0.108	0.122	3.82E-01	0.112	0.037	**2.70E-03**	-0.214	0.119	7.73E-02	-0.040	0.037	2.80E-01	0.012	0.074	8.75E-01
rs9268131	6	32362430	G/A	*NOTCH4 | C6orf10*	INTERGENIC	2	2.245	1.916	2.629	**1.20E-23**	-	-	-	-0.108	0.122	3.82E-01	-	-	-	-0.214	0.119	7.73E-02	-	-	-	0.012	0.074	8.75E-01
rs3115557	6	32347629	A/G	*NOTCH4 | C6orf10*	INTERGENIC	2	2.244	1.916	2.629	**1.23E-23**	-	-	-	-0.108	0.122	3.82E-01	-	-	-	-0.214	0.119	7.73E-02	-	-	-	0.012	0.074	8.75E-01
rs2442736	6	31454600	G/C	*LOC729816 | LOC100129668*	INTERGENIC	2	2.319	1.967	2.734	**1.27E-23**	0.088	0.038	**2.00E-02**	0.027	0.123	8.29E-01	0.211	0.038	**2.30E-08**	-0.062	0.123	6.12E-01	-0.146	0.038	**1.30E-04**	-0.017	0.074	8.16E-01
rs3129942	6	32446261	A/C	*C6orf10*	INTRON	1	2.460	2.063	2.934	**1.30E-23**	0.079	0.037	**3.20E-02**	-0.085	0.123	4.91E-01	0.073	0.037	**4.60E-02**	-0.165	0.121	1.77E-01	-0.004	0.037	9.10E-01	-0.017	0.074	8.23E-01
rs6915455	6	32391472	A/G	*C6orf10*	INTRON	2	2.217	1.897	2.591	**1.38E-23**	0.105	0.038	**5.70E-03**	-0.108	0.122	3.82E-01	0.115	0.038	**2.30E-03**	-0.214	0.119	7.73E-02	-0.033	0.038	3.80E-01	0.011	0.074	8.84E-01
rs1559874	6	32351133	A/G	*NOTCH4 | C6orf10*	INTERGENIC	2	2.242	1.914	2.626	**1.41E-23**	0.094	0.038	**1.24E-02**	-0.108	0.122	3.82E-01	0.112	0.037	**2.70E-03**	-0.214	0.119	7.73E-02	-0.040	0.037	2.80E-01	0.012	0.074	8.75E-01
rs3131931	6	31053244	T/A	*LOC100129065 | C6orf205*	INTERGENIC	2	2.217	1.897	2.590	**1.42E-23**	0.001	0.036	9.80E-01	-0.088	0.123	4.77E-01	0.123	0.036	**5.20E-04**	0.018	0.122	8.83E-01	-0.127	0.036	**4.20E-04**	-0.017	0.074	8.21E-01
rs2524074	6	31352000	G/A	*HLA-C | HLA-B*	INTERGENIC	2	2.226	1.903	2.604	**1.45E-23**	-0.049	0.033	1.31E-01	0.057	0.124	6.49E-01	0.290	0.032	**2.10E-19**	-0.135	0.123	2.75E-01	-0.354	0.032	**9.60E-28**	0.005	0.075	9.44E-01
rs1634718	6	31080844	G/A	*C6orf205 | LOC729792*	INTERGENIC	2	2.209	1.891	2.580	**1.53E-23**	0.002	0.036	9.50E-01	-0.088	0.123	4.77E-01	0.118	0.035	**8.70E-04**	0.018	0.122	8.83E-01	-0.120	0.036	**8.20E-04**	-0.015	0.074	8.43E-01
rs1018434	6	32389338	G/A	*C6orf10*	INTRON	2	2.215	1.895	2.588	**1.54E-23**	0.109	0.038	**4.40E-03**	-0.108	0.122	3.82E-01	0.116	0.038	**2.30E-03**	-0.214	0.119	7.73E-02	-0.031	0.038	4.20E-01	0.012	0.074	8.75E-01
rs3749971	6	29450754	A/G	*OR12D3*	CODING	2	2.677	2.207	3.248	**1.59E-23**	0.036	0.049	4.70E-01	0.052	0.124	6.76E-01	0.252	0.049	**2.40E-07**	-0.124	0.123	3.17E-01	-0.238	0.049	**1.40E-06**	-0.025	0.075	7.36E-01
rs3118361	6	29006266	A/G	*TRIM27 | C6orf100*	INTERGENIC	2	2.725	2.239	3.317	**1.62E-23**	0.039	0.047	4.10E-01	0.039	0.124	7.57E-01	0.193	0.047	**3.80E-05**	-0.119	0.122	3.33E-01	-0.160	0.047	**7.20E-04**	-0.011	0.074	8.78E-01
rs9393929	6	28804042	A/C	*LOC646160 | LOC442181*	INTERGENIC	2	2.703	2.224	3.286	**1.64E-23**	0.041	0.050	4.20E-01	0.037	0.124	7.66E-01	0.269	0.050	**7.20E-08**	-0.118	0.122	3.38E-01	-0.248	0.050	**8.90E-07**	-0.016	0.075	8.33E-01
rs720466	6	31233684	G/C	*CCHCR1*	INTRON	2	2.312	1.962	2.726	**1.79E-23**	0.010	0.037	7.90E-01	-0.034	0.123	7.82E-01	0.117	0.036	**1.16E-03**	-0.150	0.121	2.22E-01	-0.114	0.036	**1.80E-03**	0.113	0.074	1.26E-01
rs2844785	6	30255209	C/A	*TRIM15 | TRIM26*	INTERGENIC	2	2.509	2.094	3.006	**1.90E-23**	-	-	-	-0.027	0.125	8.29E-01	-	-	-	-0.150	0.123	2.26E-01	-	-	-	-0.073	0.075	3.33E-01
rs6909790	6	32390957	G/A	*C6orf10*	INTRON	2	2.227	1.903	2.606	**1.90E-23**	0.109	0.038	**4.40E-03**	-0.108	0.122	3.82E-01	0.116	0.038	**2.30E-03**	-0.214	0.119	7.73E-02	-0.031	0.038	4.20E-01	0.012	0.074	8.75E-01
rs2523989	6	30186254	A/G	*TRIM31*	CODING	2	2.393	2.016	2.840	**1.93E-23**	0.035	0.042	4.10E-01	0.002	0.123	9.89E-01	0.231	0.042	**3.10E-08**	-0.134	0.121	2.73E-01	-0.214	0.042	**3.80E-07**	0.051	0.074	4.92E-01
rs1018433	6	32389488	A/T	*C6orf10*	INTRON	2	2.227	1.903	2.606	**1.96E-23**	0.109	0.038	**4.40E-03**	-0.108	0.122	3.82E-01	0.116	0.038	**2.30E-03**	-0.214	0.119	7.73E-02	-0.031	0.038	4.20E-01	0.012	0.074	8.75E-01
rs9268215	6	32390449	G/A	*C6orf10*	INTRON	2	2.226	1.902	2.606	**1.98E-23**	-	-	-	-0.108	0.122	3.82E-01	-	-	-	-0.214	0.119	7.73E-02	-	-	-	0.012	0.074	8.75E-01
rs3117427	6	29382115	G/A	*LOC651503 | OR5U1*	INTERGENIC	2	2.686	2.212	3.262	**2.15E-23**	0.036	0.049	4.60E-01	0.059	0.124	6.37E-01	0.238	0.048	**7.30E-07**	-0.115	0.123	3.51E-01	-0.224	0.049	**4.00E-06**	-0.016	0.074	8.26E-01
rs2517598	6	30188253	A/G	*TRIM31*	CODING	2	2.374	2.003	2.815	**2.29E-23**	0.039	0.042	3.60E-01	0.002	0.123	9.89E-01	0.227	0.041	**4.20E-08**	-0.134	0.121	2.73E-01	-0.207	0.042	**7.80E-07**	0.047	0.074	5.24E-01
rs9268878	6	32539270	T/A	*HLA-DRA | HLA-DRB5*	INTERGENIC	1	2.410	2.027	2.866	**2.33E-23**	0.062	0.038	1.02E-01	-0.069	0.123	5.76E-01	0.035	0.037	3.50E-01	-0.195	0.120	1.08E-01	0.018	0.038	6.40E-01	-0.018	0.074	8.12E-01
rs2517425	6	31057649	C/A	*LOC100129065 | C6orf205*	INTERGENIC	2	2.217	1.895	2.593	**2.34E-23**	-0.001	0.035	9.80E-01	-0.094	0.122	4.44E-01	0.115	0.035	**1.10E-03**	-0.010	0.122	9.38E-01	-0.119	0.035	**7.70E-04**	-0.022	0.074	7.68E-01
rs2844793	6	30188475	A/G	*TRIM31*	CODING	2	2.387	2.011	2.834	**2.52E-23**	0.039	0.042	3.60E-01	0.002	0.123	9.89E-01	0.227	0.041	**4.20E-08**	-0.134	0.121	2.73E-01	-0.207	0.042	**7.80E-07**	0.051	0.074	4.92E-01
rs2050189	6	32447625	G/A	*C6orf10*	UTR	2	2.240	1.911	2.626	**2.75E-23**	0.046	0.038	2.20E-01	-0.039	0.123	7.52E-01	0.104	0.037	**5.60E-03**	-0.151	0.121	2.16E-01	-0.067	0.038	7.50E-02	0.038	0.075	6.12E-01
rs3096673	6	32345991	G/A	*NOTCH4 | C6orf10*	INTERGENIC	2	2.220	1.897	2.598	**2.92E-23**	0.094	0.038	**1.24E-02**	-0.108	0.122	3.82E-01	0.112	0.037	**2.70E-03**	-0.214	0.119	7.73E-02	-0.040	0.037	2.80E-01	0.013	0.074	8.65E-01
rs3096681	6	32343155	G/A	*NOTCH4 | C6orf10*	INTERGENIC	2	2.220	1.897	2.598	**2.92E-23**	-	-	-	-0.108	0.122	3.82E-01	-	-	-	-0.214	0.119	7.73E-02	-	-	-	0.013	0.074	8.65E-01
rs3115553	6	32353805	A/G	*NOTCH4 | C6orf10*	INTERGENIC	2	2.220	1.897	2.598	**2.92E-23**	0.094	0.038	**1.24E-02**	-0.108	0.122	3.82E-01	0.112	0.037	**2.70E-03**	-0.214	0.119	7.73E-02	-0.040	0.037	2.80E-01	0.013	0.074	8.65E-01
rs3115569	6	32332117	A/G	*NOTCH4 | C6orf10*	INTERGENIC	2	2.220	1.897	2.598	**2.92E-23**	-	-	-	-0.108	0.122	3.82E-01	-	-	-	-0.214	0.119	7.73E-02	-	-	-	0.013	0.074	8.65E-01
rs3130340	6	32352605	G/A	*NOTCH4 | C6orf10*	INTERGENIC	2	2.220	1.897	2.598	**2.92E-23**	0.094	0.038	**1.24E-02**	-0.108	0.122	3.82E-01	0.112	0.037	**2.70E-03**	-0.214	0.119	7.73E-02	-0.040	0.037	2.80E-01	0.013	0.074	8.65E-01
rs3132928	6	32341993	A/G	*NOTCH4 | C6orf10*	INTERGENIC	2	2.220	1.897	2.598	**2.92E-23**	-	-	-	-0.108	0.122	3.82E-01	-	-	-	-0.214	0.119	7.73E-02	-	-	-	0.013	0.074	8.65E-01
rs7751896	6	32363388	A/G	*NOTCH4 | C6orf10*	INTERGENIC	2	2.219	1.896	2.598	**2.92E-23**	-	-	-	-0.108	0.122	3.82E-01	-	-	-	-0.214	0.119	7.73E-02	-	-	-	0.011	0.074	8.86E-01
rs9268055	6	32338586	G/A	*NOTCH4 | C6orf10*	INTERGENIC	2	2.220	1.897	2.598	**2.92E-23**	-	-	-	-0.108	0.122	3.82E-01	-	-	-	-0.214	0.119	7.73E-02	-	-	-	0.013	0.074	8.65E-01
rs3115560	6	32344120	A/G	*NOTCH4 | C6orf10*	INTERGENIC	2	2.219	1.896	2.597	**2.95E-23**	0.094	0.038	**1.24E-02**	-0.108	0.122	3.82E-01	0.112	0.037	**2.70E-03**	-0.214	0.119	7.73E-02	-0.040	0.037	2.80E-01	0.013	0.074	8.60E-01
rs3130837	6	29056071	A/C	*C6orf100 | ZNF311*	INTERGENIC	2	2.702	2.221	3.287	**3.04E-23**	0.039	0.050	4.40E-01	0.037	0.124	7.66E-01	0.261	0.050	**1.50E-07**	-0.118	0.122	3.38E-01	-0.242	0.050	**1.40E-06**	-0.016	0.074	8.32E-01
rs3864300	6	32379785	A/C	*C6orf10*	INTRON	2	2.209	1.889	2.584	**3.09E-23**	0.109	0.038	**4.40E-03**	-0.108	0.122	3.82E-01	0.116	0.038	**2.30E-03**	-0.214	0.119	7.73E-02	-0.031	0.038	4.20E-01	0.012	0.074	8.75E-01
rs9268165	6	32380305	G/A	*C6orf10*	INTRON	2	2.209	1.889	2.583	**3.09E-23**	-	-	-	-0.108	0.122	3.82E-01	-	-	-	-0.214	0.119	7.73E-02	-	-	-	0.012	0.074	8.75E-01
rs9268192	6	32385189	A/G	*C6orf10*	INTRON	2	2.209	1.889	2.584	**3.09E-23**	-	-	-	-0.108	0.122	3.82E-01	-	-	-	-0.214	0.119	7.73E-02	-	-	-	0.012	0.074	8.75E-01
rs6457536	6	32381743	G/A	*C6orf10*	INTRON	2	2.209	1.889	2.583	**3.12E-23**	0.109	0.038	**4.40E-03**	-0.108	0.122	3.82E-01	0.116	0.038	**2.30E-03**	-0.214	0.119	7.73E-02	-0.031	0.038	4.20E-01	0.012	0.074	8.75E-01
rs9268198	6	32386165	A/G	*C6orf10*	INTRON	2	2.209	1.889	2.583	**3.12E-23**	-	-	-	-0.108	0.122	3.82E-01	-	-	-	-0.214	0.119	7.73E-02	-	-	-	0.012	0.074	8.75E-01
rs200990	6	27923802	C/A	*HIST1H2BN | HIST1H2AL*	INTERGENIC	2	2.642	2.181	3.200	**3.19E-23**	-	-	-	0.050	0.123	6.86E-01	-	-	-	-0.091	0.122	4.59E-01	-	-	-	-0.038	0.074	6.07E-01
rs7742654	6	32379421	G/C	*C6orf10*	INTRON	2	2.209	1.889	2.583	**3.22E-23**	0.109	0.038	**4.40E-03**	-0.108	0.122	3.82E-01	0.116	0.038	**2.30E-03**	-0.214	0.119	7.73E-02	-0.031	0.038	4.20E-01	0.012	0.074	8.75E-01
rs3130895	6	29013770	A/G	*TRIM27 | C6orf100*	INTERGENIC	2	2.694	2.215	3.276	**3.26E-23**	-	-	-	0.039	0.124	7.57E-01	-	-	-	-0.119	0.122	3.33E-01	-	-	-	-0.016	0.074	8.25E-01
rs13194504	6	28738670	A/G	*LOC646160 | LOC442181*	INTERGENIC	2	2.713	2.227	3.304	**3.41E-23**	0.035	0.050	4.90E-01	0.037	0.124	7.66E-01	0.269	0.050	**7.30E-08**	-0.118	0.122	3.38E-01	-0.252	0.050	**5.80E-07**	-0.023	0.074	7.57E-01
rs3131036	6	30836269	A/G	*IER3 | DDR1*	INTERGENIC	2	2.226	1.901	2.608	**3.47E-23**	-0.023	0.036	5.20E-01	0.028	0.123	8.22E-01	0.135	0.035	**1.40E-04**	0.061	0.122	6.21E-01	-0.159	0.036	**8.00E-06**	0.003	0.075	9.68E-01
rs6457374	6	31380240	G/A	*HLA-C | HLA-B*	INTERGENIC	1	2.470	2.066	2.954	**3.73E-23**	-0.044	0.034	1.90E-01	0.039	0.125	7.59E-01	0.294	0.033	**8.10E-19**	-0.159	0.123	1.99E-01	-0.356	0.033	**1.60E-26**	0.025	0.076	7.43E-01
rs1311918	6	28861625	A/G	*LOC442181 | LOC401242*	INTERGENIC	2	2.697	2.215	3.283	**4.85E-23**	-	-	-	0.037	0.124	7.66E-01	-	-	-	-0.118	0.122	3.38E-01	-	-	-	-0.016	0.074	8.32E-01
rs1233579	6	28820642	G/A	*LOC442181 | LOC401242*	INTERGENIC	2	2.695	2.214	3.282	**5.12E-23**	0.037	0.051	4.60E-01	0.037	0.124	7.66E-01	0.268	0.050	**8.10E-08**	-0.118	0.122	3.38E-01	-0.250	0.051	**7.20E-07**	-0.016	0.074	8.32E-01
rs2523984	6	30189982	A/G	*TRIM31 | TRIM40*	INTERGENIC	2	2.364	1.993	2.804	**5.36E-23**	0.039	0.042	3.60E-01	0.002	0.123	9.89E-01	0.227	0.041	**4.20E-08**	-0.134	0.121	2.73E-01	-0.207	0.042	**7.80E-07**	0.047	0.074	5.24E-01

rs2245420	6	30190667	C/G	*TRIM31 | TRIM40*	INTERGENIC	2	2.363	1.992	2.803	**5.46E-23**	0.039	0.042	3.60E-01	0.002	0.123	9.89E-01	0.227	0.041	**4.20E-08**	-0.134	0.121	2.73E-01	-0.207	0.042	**7.80E-07**	0.047	0.074	5.24E-01
rs2249099	6	30187286	A/C	*TRIM31*	INTRON	2	2.363	1.992	2.803	**5.46E-23**	0.035	0.042	4.10E-01	0.002	0.123	9.89E-01	0.231	0.042	**3.10E-08**	-0.134	0.121	2.73E-01	-0.214	0.042	**3.80E-07**	0.047	0.074	5.24E-01
rs9257136	6	28831820	C/A	*LOC442181 | LOC401242*	INTERGENIC	2	2.688	2.209	3.271	**5.50E-23**	-0.010	0.058	8.60E-01	0.037	0.124	7.66E-01	0.247	0.057	**1.40E-05**	-0.118	0.122	3.38E-01	-0.280	0.057	**1.00E-06**	-0.016	0.074	8.32E-01
rs2517403	6	31174988	G/A	*HCG22 | C6orf15*	INTERGENIC	2	2.158	1.852	2.514	**5.63E-23**	-0.036	0.034	2.90E-01	-0.098	0.123	4.27E-01	0.111	0.034	**1.03E-03**	0.027	0.123	8.26E-01	-0.148	0.034	**1.40E-05**	-0.099	0.074	1.80E-01
rs2844635	6	31183460	G/A	*HCG22 | C6orf15*	INTERGENIC	2	2.166	1.858	2.526	**5.79E-23**	-0.036	0.034	2.90E-01	-0.098	0.123	4.27E-01	0.111	0.034	**1.03E-03**	0.027	0.123	8.26E-01	-0.148	0.034	**1.40E-05**	-0.099	0.074	1.80E-01
rs2230683	6	28999155	G/A	*TRIM27*	CODING	2	2.691	2.210	3.276	**6.14E-23**	0.039	0.047	4.10E-01	0.039	0.124	7.57E-01	0.193	0.047	**3.80E-05**	-0.119	0.122	3.33E-01	-0.160	0.047	**7.20E-04**	-0.016	0.074	8.25E-01
rs1265159	6	31248026	A/G	*POU5F1 | LOC100130889*	INTERGENIC	2	2.311	1.956	2.730	**6.17E-23**	0.028	0.037	4.50E-01	-0.016	0.123	8.95E-01	0.122	0.037	**8.50E-04**	-0.189	0.121	1.21E-01	-0.104	0.037	**4.70E-03**	0.134	0.073	7.03E-02
rs6909427	6	32376679	C/A	*C6orf10*	INTRON	2	2.213	1.890	2.591	**6.18E-23**	0.091	0.038	**1.49E-02**	-0.108	0.122	3.82E-01	0.109	0.037	**3.50E-03**	-0.214	0.119	7.73E-02	-0.039	0.037	2.90E-01	0.013	0.074	8.65E-01
rs3749966	6	32369485	G/A	*C6orf10*	CODING	2	2.212	1.889	2.591	**6.24E-23**	0.091	0.038	**1.49E-02**	-0.108	0.122	3.82E-01	0.109	0.037	**3.50E-03**	-0.214	0.119	7.73E-02	-0.039	0.037	2.90E-01	0.013	0.074	8.65E-01
rs9268167	6	32380415	A/C	*C6orf10*	INTRON	2	2.212	1.889	2.591	**6.24E-23**	-	-	-	-0.108	0.122	3.82E-01	-	-	-	-0.214	0.119	7.73E-02	-	-	-	0.013	0.074	8.65E-01
rs3117326	6	29348357	A/G	*LOC651503 | OR5U1*	INTERGENIC	2	2.663	2.192	3.235	**6.45E-23**	0.030	0.049	5.40E-01	0.037	0.124	7.66E-01	0.241	0.048	**5.80E-07**	-0.118	0.122	3.38E-01	-0.229	0.049	**2.60E-06**	-0.017	0.074	8.23E-01
rs3130834	6	29356128	G/A	*LOC651503 | OR5U1*	INTERGENIC	2	2.663	2.192	3.235	**6.45E-23**	0.030	0.049	5.40E-01	0.037	0.124	7.66E-01	0.241	0.048	**5.80E-07**	-0.118	0.122	3.38E-01	-0.229	0.049	**2.60E-06**	-0.017	0.074	8.23E-01
rs6935269	6	32368328	G/A	*NOTCH4 | C6orf10*	INTERGENIC	2	2.212	1.889	2.590	**6.50E-23**	0.091	0.038	**1.49E-02**	-0.108	0.122	3.82E-01	0.109	0.037	**3.50E-03**	-0.214	0.119	7.73E-02	-0.039	0.037	2.90E-01	0.013	0.074	8.65E-01
rs2523719	6	30276298	A/C	*TRIM26*	INTRON	2	2.488	2.075	2.982	**6.69E-23**	0.010	0.043	8.10E-01	-0.019	0.125	8.78E-01	0.213	0.042	**5.00E-07**	-0.135	0.123	2.76E-01	-0.208	0.043	**1.10E-06**	-0.073	0.075	3.30E-01
rs3135309	6	28963784	C/A	*LOC401242 | TRIM27*	INTERGENIC	2	2.682	2.204	3.264	**6.83E-23**	0.034	0.047	4.70E-01	0.039	0.124	7.57E-01	0.199	0.047	**2.10E-05**	-0.119	0.122	3.33E-01	-0.171	0.047	**3.00E-04**	-0.016	0.074	8.25E-01
rs1233604	6	28842655	A/G	*LOC442181 | LOC401242*	INTERGENIC	2	2.680	2.203	3.262	**7.21E-23**	0.041	0.051	4.20E-01	0.037	0.124	7.66E-01	0.269	0.050	**7.40E-08**	-0.118	0.122	3.38E-01	-0.249	0.051	**8.90E-07**	-0.016	0.074	8.32E-01
rs3131343	6	28883543	A/G	*LOC442181 | LOC401242*	INTERGENIC	2	2.678	2.201	3.259	**7.76E-23**	0.045	0.050	3.70E-01	0.037	0.124	7.66E-01	0.251	0.049	**3.00E-07**	-0.118	0.122	3.38E-01	-0.226	0.050	**5.00E-06**	-0.016	0.074	8.32E-01
rs7767099	6	28876677	A/G	*LOC442181 | LOC401242*	INTERGENIC	2	2.678	2.201	3.259	**7.76E-23**	0.038	0.051	4.50E-01	0.037	0.124	7.66E-01	0.268	0.050	**8.50E-08**	-0.118	0.122	3.38E-01	-0.250	0.051	**7.90E-07**	-0.016	0.074	8.32E-01
rs3094117	6	30845465	C/A	*IER3 | DDR1*	INTERGENIC	2	2.202	1.882	2.577	**7.79E-23**	-0.023	0.036	5.20E-01	0.016	0.123	8.96E-01	0.135	0.035	**1.40E-04**	-0.077	0.122	5.29E-01	-0.159	0.036	**8.00E-06**	0.018	0.074	8.12E-01
rs1233599	6	28839167	C/A	*LOC442181 | LOC401242*	INTERGENIC	2	2.672	2.197	3.250	**7.83E-23**	-	-	-	0.037	0.124	7.66E-01	-	-	-	-0.118	0.122	3.38E-01	-	-	-	-0.016	0.074	8.32E-01
rs4324798	6	28884096	A/G	*LOC442181 | LOC401242*	INTERGENIC	2	2.678	2.201	3.259	**7.89E-23**	0.038	0.051	4.50E-01	0.037	0.124	7.66E-01	0.268	0.050	**8.50E-08**	-0.118	0.122	3.38E-01	-0.250	0.051	**7.90E-07**	-0.016	0.074	8.32E-01
rs3132389	6	28939000	C/A	*LOC401242*	INTRON	2	2.669	2.194	3.246	**8.80E-23**	0.035	0.051	4.90E-01	0.037	0.124	7.66E-01	0.271	0.050	**6.00E-08**	-0.118	0.122	3.38E-01	-0.255	0.050	**4.20E-07**	-0.016	0.074	8.32E-01
rs1233396	6	29654778	A/G	*UBD | SNORD32B*	INTERGENIC	2	2.552	2.117	3.076	**8.96E-23**	0.039	0.047	4.10E-01	0.102	0.124	4.10E-01	0.257	0.047	**3.70E-08**	-0.060	0.123	6.29E-01	-0.239	0.047	**4.30E-07**	-0.030	0.075	6.89E-01
rs3129986	6	30871541	A/G	*IER3 | DDR1*	INTERGENIC	1	2.750	2.247	3.366	**9.61E-23**	-	-	-	-0.004	0.124	9.76E-01	-	-	-	-0.155	0.122	2.07E-01	-	-	-	0.005	0.074	9.46E-01
rs3864302	6	32386770	A/G	*C6orf10*	INTRON	2	2.192	1.874	2.564	**9.74E-23**	0.109	0.038	**4.40E-03**	-0.108	0.122	3.82E-01	0.116	0.038	**2.30E-03**	-0.214	0.119	7.73E-02	-0.031	0.038	4.20E-01	0.013	0.074	8.65E-01
rs3094112	6	30869713	G/A	*IER3 | DDR1*	INTERGENIC	2	2.237	1.905	2.627	**9.75E-23**	-0.004	0.038	9.10E-01	0.096	0.122	4.37E-01	0.170	0.037	**5.30E-06**	-0.048	0.122	6.94E-01	-0.178	0.038	**2.30E-06**	0.046	0.074	5.39E-01
rs3132392	6	28946608	G/A	*LOC401242 | TRIM27*	INTERGENIC	2	2.679	2.200	3.262	**1.01E-22**	0.038	0.051	4.60E-01	0.037	0.124	7.66E-01	0.272	0.050	**5.20E-08**	-0.118	0.122	3.38E-01	-0.256	0.051	**4.40E-07**	-0.016	0.074	8.32E-01
rs3129788	6	29165618	A/G	*OR2B3P | OR2J3*	INTERGENIC	2	2.665	2.191	3.241	**1.03E-22**	0.039	0.050	4.40E-01	0.037	0.124	7.66E-01	0.261	0.050	**1.50E-07**	-0.118	0.122	3.38E-01	-0.242	0.050	**1.40E-06**	-0.016	0.074	8.32E-01
rs2523985	6	30189313	G/A	*TRIM31 | TRIM40*	INTERGENIC	2	2.356	1.985	2.796	**1.09E-22**	-	-	-	0.002	0.123	9.89E-01	-	-	-	-0.134	0.121	2.73E-01	-	-	-	0.047	0.074	5.24E-01
rs3130888	6	29057939	G/A	*C6orf100 | ZNF311*	INTERGENIC	2	2.655	2.184	3.227	**1.18E-22**	0.031	0.050	5.30E-01	0.037	0.124	7.66E-01	0.250	0.049	**4.30E-07**	-0.118	0.122	3.38E-01	-0.237	0.050	**2.20E-06**	-0.016	0.074	8.32E-01
rs2524142	6	31371501	C/G	*HLA-C | HLA-B*	INTERGENIC	1	2.460	2.054	2.946	**1.29E-22**	-0.048	0.033	1.60E-01	0.036	0.124	7.74E-01	0.294	0.033	**8.00E-19**	-0.161	0.122	1.93E-01	-0.359	0.033	**6.80E-27**	0.025	0.076	7.43E-01
rs1265061	6	31186236	A/C	*HCG22 | C6orf15*	INTERGENIC	2	2.158	1.849	2.517	**1.40E-22**	-0.036	0.034	3.00E-01	-0.098	0.123	4.27E-01	0.112	0.034	**9.60E-04**	0.027	0.123	8.26E-01	-0.148	0.034	**1.50E-05**	-0.099	0.074	1.80E-01
rs2517618	6	30266106	A/G	*TRIM26*	INTRON	2	2.478	2.066	2.972	**1.41E-22**	0.014	0.042	7.40E-01	-0.027	0.125	8.29E-01	0.205	0.042	**1.00E-06**	-0.150	0.123	2.26E-01	-0.197	0.043	**3.40E-06**	-0.068	0.075	3.63E-01
rs707929	6	31850046	G/A	*C6orf27*	INTRON	2	2.136	1.835	2.487	**1.43E-22**	0.043	0.034	2.00E-01	-0.094	0.123	4.46E-01	0.062	0.034	6.60E-02	-0.039	0.123	7.51E-01	-0.033	0.034	3.40E-01	-0.084	0.074	2.59E-01
rs926552	6	29656068	A/G	*UBD | SNORD32B*	INTERGENIC	2	2.540	2.107	3.062	**1.43E-22**	0.039	0.047	4.10E-01	0.102	0.124	4.10E-01	0.257	0.047	**3.70E-08**	-0.060	0.123	6.29E-01	-0.239	0.047	**4.30E-07**	-0.030	0.075	6.89E-01
rs6908726	6	28779322	G/C	*LOC646160 | LOC442181*	INTERGENIC	2	2.631	2.167	3.194	**1.44E-22**	0.038	0.051	4.50E-01	0.037	0.124	7.66E-01	0.272	0.050	**5.40E-08**	-0.118	0.122	3.38E-01	-0.252	0.051	**6.00E-07**	-0.015	0.074	8.36E-01
rs3132650	6	30420155	G/A	*TRIM39 | RPP21*	INTERGENIC	2	2.426	2.031	2.899	**1.48E-22**	-	-	-	-0.045	0.124	7.15E-01	-	-	-	-0.165	0.122	1.80E-01	-	-	-	-0.081	0.074	2.79E-01
rs2245822	6	31338779	A/G	*HCG27 | HLA-C*	INTERGENIC	2	2.249	1.911	2.646	**1.58E-22**	0.005	0.036	8.80E-01	0.009	0.123	9.41E-01	0.066	0.036	6.50E-02	-0.116	0.122	3.43E-01	-0.066	0.036	7.00E-02	0.134	0.073	7.03E-02
rs3130845	6	29031346	G/A	*C6orf100 | ZNF311*	INTERGENIC	2	2.661	2.186	3.238	**1.58E-22**	0.032	0.047	5.00E-01	0.037	0.124	7.66E-01	0.199	0.047	**2.20E-05**	-0.118	0.122	3.38E-01	-0.173	0.047	**2.70E-04**	-0.016	0.074	8.32E-01
rs3130773	6	29203887	A/G	*OR2J3 | OR2J2*	INTERGENIC	2	2.647	2.177	3.218	**1.60E-22**	0.039	0.050	4.40E-01	0.037	0.124	7.66E-01	0.261	0.050	**1.50E-07**	-0.118	0.122	3.38E-01	-0.242	0.050	**1.40E-06**	-0.016	0.074	8.32E-01
rs3117143	6	29139121	A/C	*OR2W1 | LOC100129636*	INTERGENIC	2	2.658	2.184	3.235	**1.74E-22**	0.039	0.050	4.40E-01	0.037	0.124	7.66E-01	0.261	0.050	**1.50E-07**	-0.118	0.122	3.38E-01	-0.242	0.050	**1.40E-06**	-0.016	0.074	8.32E-01
rs3130893	6	29088686	G/A	*ZNF311 | LOC100129636*	INTERGENIC	2	2.644	2.175	3.214	**1.78E-22**	0.039	0.050	4.40E-01	0.037	0.124	7.66E-01	0.261	0.050	**1.50E-07**	-0.118	0.122	3.38E-01	-0.242	0.050	**1.40E-06**	-0.016	0.074	8.32E-01
rs9275698	6	32795951	G/A	*HLA-DQB1 | HLA-DQA2*	INTERGENIC	2	2.155	1.846	2.514	**1.93E-22**	0.040	0.033	2.20E-01	-0.023	0.123	8.53E-01	-0.014	0.033	6.70E-01	-0.041	0.122	7.39E-01	0.056	0.033	8.60E-02	0.038	0.074	6.12E-01
rs3116830	6	29275554	A/G	*OR2J2 | LOC651503*	INTERGENIC	2	2.648	2.177	3.221	**2.05E-22**	0.031	0.049	5.30E-01	0.037	0.124	7.66E-01	0.242	0.048	**5.50E-07**	-0.118	0.122	3.38E-01	-0.229	0.049	**2.60E-06**	-0.016	0.074	8.32E-01
rs13205911	6	28232093	A/G	*ZNF192*	UTR	2	2.678	2.196	3.265	**2.09E-22**	0.057	0.051	2.60E-01	0.052	0.123	6.73E-01	0.258	0.050	**2.50E-07**	-0.106	0.122	3.90E-01	-0.227	0.051	**7.70E-06**	-0.032	0.074	6.68E-01
rs13218875	6	27991991	A/G	*OR2B2 | OR2B6*	INTERGENIC	2	2.690	2.204	3.284	**2.16E-22**	-	-	-	0.052	0.123	6.73E-01	-	-	-	-0.106	0.122	3.90E-01	-	-	-	-0.032	0.074	6.68E-01
rs3129837	6	30414285	G/A	*TRIM39*	INTRON	2	2.410	2.019	2.877	**2.24E-22**	-	-	-	-0.045	0.124	7.15E-01	-	-	-	-0.165	0.122	1.80E-01	-	-	-	-0.081	0.075	2.82E-01
rs3132390	6	28940767	G/A	*LOC401242 | TRIM27*	INTERGENIC	2	2.612	2.152	3.169	**2.34E-22**	0.035	0.051	4.90E-01	0.037	0.124	7.66E-01	0.271	0.050	**6.00E-08**	-0.118	0.122	3.38E-01	-0.255	0.050	**4.20E-07**	-0.016	0.075	8.33E-01
rs200948	6	27943251	G/A	*HIST1H1B*	CODING	2	2.580	2.131	3.123	**2.42E-22**	0.029	0.049	5.60E-01	0.050	0.123	6.86E-01	0.216	0.048	**7.20E-06**	-0.091	0.122	4.59E-01	-0.206	0.049	**2.30E-05**	-0.045	0.074	5.41E-01
rs887468	6	31249502	A/G	*POU5F1 | LOC100130889*	INTERGENIC	2	2.229	1.896	2.620	**2.44E-22**	0.004	0.035	9.20E-01	-0.028	0.123	8.19E-01	0.073	0.035	**3.80E-02**	-0.133	0.121	2.76E-01	-0.076	0.035	**3.20E-02**	0.114	0.074	1.23E-01
rs13211507	6	28365356	G/A	*PGBD1*	INTRON	2	2.686	2.201	3.279	**2.55E-22**	0.029	0.051	5.60E-01	0.037	0.124	7.66E-01	0.265	0.050	**1.50E-07**	-0.118	0.122	3.38E-01	-0.254	0.051	**6.30E-07**	-0.018	0.074	8.08E-01
rs13197574	6	28168218	G/A	*ZNF165 | LOC100129195*	INTERGENIC	2	2.679	2.196	3.268	**2.71E-22**	0.047	0.051	3.50E-01	0.052	0.123	6.73E-01	0.267	0.051	**1.30E-07**	-0.106	0.122	3.90E-01	-0.240	0.051	**2.70E-06**	-0.032	0.074	6.68E-01
rs200490	6	27904914	A/C	*HIST1H4J | HIST1H4K*	INTERGENIC	2	2.570	2.124	3.109	**2.81E-22**	0.032	0.049	5.10E-01	0.050	0.123	6.86E-01	0.219	0.048	**5.70E-06**	-0.091	0.122	4.59E-01	-0.206	0.049	**2.40E-05**	-0.038	0.074	6.07E-01
rs9257803	6	29452374	A/G	*OR12D3 | OR12D2*	INTERGENIC	2	2.545	2.107	3.073	**2.87E-22**	0.036	0.049	4.70E-01	0.032	0.124	7.94E-01	0.252	0.049	**2.40E-07**	-0.135	0.122	2.75E-01	-0.238	0.049	**1.40E-06**	-0.018	0.075	8.13E-01
rs9257248	6	28911270	A/G	*LOC442181 | LOC401242*	INTERGENIC	2	2.647	2.174	3.221	**2.88E-22**	0.038	0.051	4.50E-01	0.037	0.124	7.66E-01	0.268	0.050	**8.50E-08**	-0.118	0.122	3.38E-01	-0.250	0.051	**7.90E-07**	-0.016	0.074	8.32E-01
rs41468444	6	27945246	G/A	*HIST1H1B | HIST1H3I*	INTERGENIC	2	2.588	2.136	3.136	**2.98E-22**	-	-	-	0.050	0.123	6.86E-01	-	-	-	-0.091	0.122	4.59E-01	-	-	-	-0.045	0.074	5.41E-01
rs1793891	6	31329677	A/G	*HCG27 | HLA-C*	INTERGENIC	2	2.204	1.879	2.586	**3.05E-22**	0.008	0.036	8.30E-01	0.009	0.123	9.41E-01	0.066	0.036	6.70E-02	-0.116	0.122	3.43E-01	-0.063	0.036	8.10E-02	0.136	0.073	6.56E-02
rs13213152	6	28457677	G/A	*ZKSCAN3 | ZSCAN12*	INTERGENIC	2	2.674	2.192	3.262	**3.16E-22**	0.043	0.049	3.90E-01	0.037	0.124	7.66E-01	0.248	0.048	**3.20E-07**	-0.118	0.122	3.38E-01	-0.228	0.049	**3.20E-06**	-0.018	0.074	8.06E-01
rs2232423	6	28474130	G/A	*ZSCAN12*	UTR	2	2.674	2.192	3.262	**3.16E-22**	0.042	0.049	3.90E-01	0.037	0.124	7.66E-01	0.251	0.048	**1.80E-07**	-0.118	0.122	3.38E-01	-0.233	0.049	**1.70E-06**	-0.018	0.074	8.06E-01
rs2248880	6	31341489	G/A	*HCG27 | HLA-C*	INTERGENIC	2	2.242	1.904	2.639	**3.23E-22**	0.005	0.036	8.80E-01	0.009	0.123	9.41E-01	0.066	0.036	6.50E-02	-0.116	0.122	3.43E-01	-0.066	0.036	7.00E-02	0.134	0.073	7.03E-02
rs1043618	6	31891486	G/C	*HSPA1A*	UTR	2	2.123	1.823	2.471	**3.24E-22**	0.006	0.033	8.60E-01	-0.150	0.123	2.28E-01	0.033	0.033	3.20E-01	0.027	0.124	8.28E-01	-0.025	0.033	4.50E-01	-0.104	0.074	1.63E-01
rs2517613	6	30273641	A/G	*TRIM26*	INTRON	2	2.455	2.046	2.944	**3.84E-22**	0.010	0.043	8.10E-01	-0.019	0.125	8.78E-01	0.213	0.042	**5.00E-07**	-0.135	0.123	2.76E-01	-0.208	0.043	**1.10E-06**	-0.070	0.075	3.51E-01
rs2523720	6	30274865	A/G	*TRIM26*	UTR	2	2.454	2.046	2.944	**3.99E-22**	0.010	0.043	8.10E-01	-0.019	0.125	8.78E-01	0.213	0.042	**5.00E-07**	-0.135	0.123	2.76E-01	-0.208	0.043	**1.10E-06**	-0.070	0.075	3.51E-01
rs2188100	6	30289862	A/G	*TRIM26 | FLJ45422*	INTERGENIC	2	2.453	2.045	2.942	**4.19E-22**	-	-	-	-0.019	0.125	8.78E-01	-	-	-	-0.135	0.123	2.76E-01	-	-	-	-0.070	0.075	3.51E-01
rs885916	6	30310550	A/G	*TRIM26 | FLJ45422*	INTERGENIC	2	2.452	2.044	2.942	**4.23E-22**	0.014	0.043	7.40E-01	-0.027	0.125	8.29E-01	0.215	0.042	**3.30E-07**	-0.150	0.123	2.26E-01	-0.207	0.043	**1.10E-06**	-0.068	0.075	3.63E-01
rs200989	6	27924421	G/A	*HIST1H2BN | HIST1H2AL*	INTERGENIC	2	2.572	2.123	3.115	**4.36E-22**	0.029	0.049	5.60E-01	0.050	0.123	6.86E-01	0.216	0.048	**7.20E-06**	-0.091	0.122	4.59E-01	-0.206	0.049	**2.30E-05**	-0.035	0.074	6.35E-01
rs2517612	6	30277071	G/A	*TRIM26*	INTRON	2	2.444	2.039	2.930	**4.46E-22**	0.010	0.043	8.10E-01	-0.019	0.125	8.78E-01	0.213	0.042	**5.00E-07**	-0.135	0.123	2.76E-01	-0.208	0.043	**1.10E-06**	-0.070	0.075	3.51E-01
rs200995	6	27921673	G/A	*HIST1H2BN | HIST1H2AL*	INTERGENIC	2	2.564	2.118	3.105	**4.60E-22**	0.032	0.049	5.10E-01	0.050	0.123	6.86E-01	0.219	0.048	**5.70E-06**	-0.091	0.122	4.59E-01	-0.206	0.049	**2.40E-05**	-0.038	0.074	6.07E-01
rs175597	6	27918605	G/A	*HIST1H2BN | HIST1H2AL*	INTERGENIC	2	2.564	2.118	3.104	**4.73E-22**	0.032	0.049	5.10E-01	0.050	0.123	6.86E-01	0.219	0.048	**5.70E-06**	-0.091	0.122	4.59E-01	-0.206	0.049	**2.40E-05**	-0.038	0.074	6.07E-01

rs2747054	6	27891338	G/A	*HIST1H2BM | HIST1H4J*	INTERGENIC	2	2.563	2.117	3.103	**4.83E-22**	-	-	-	0.050	0.123	6.86E-01	-	-	-	-0.091	0.122	4.59E-01	-	-	-	-0.038	0.074	6.07E-01
rs201002	6	27916171	G/A	*HIST1H2BN | HIST1H2AL*	INTERGENIC	2	2.563	2.117	3.102	**4.93E-22**	0.032	0.049	5.10E-01	0.050	0.123	6.86E-01	0.219	0.048	**5.70E-06**	-0.091	0.122	4.59E-01	-0.206	0.049	**2.40E-05**	-0.038	0.074	6.07E-01
rs9257805	6	29454308	G/A	*OR12D3 | OR12D2*	INTERGENIC	2	2.538	2.101	3.067	**5.00E-22**	0.036	0.049	4.70E-01	0.032	0.124	7.94E-01	0.252	0.049	**2.40E-07**	-0.135	0.122	2.75E-01	-0.238	0.049	**1.40E-06**	-0.024	0.075	7.52E-01
rs9271858	6	32703201	A/G	*HLA-DRB1 | HLA-DQA1*	INTERGENIC	2	0.444	0.376	0.524	**5.53E-22**	-	-	-	0.099	0.123	4.22E-01	-	-	-	0.141	0.121	2.49E-01	-	-	-	-0.048	0.074	5.14E-01
rs200501	6	27896921	A/G	*HIST1H2BM | HIST1H4J*	INTERGENIC	2	2.560	2.114	3.099	**5.59E-22**	0.032	0.049	5.10E-01	0.135	0.122	2.71E-01	0.219	0.048	**5.70E-06**	0.000	0.122	9.99E-01	-0.206	0.049	**2.40E-05**	-0.045	0.074	5.47E-01
rs2471980	6	31908847	G/C	*HSPA1B | C6orf48*	INTERGENIC	2	2.125	1.823	2.477	**5.63E-22**	0.021	0.035	5.60E-01	-0.155	0.122	2.06E-01	0.107	0.035	**2.00E-03**	-0.080	0.122	5.14E-01	-0.097	0.035	**5.40E-03**	-0.074	0.074	3.19E-01
rs9272143	6	32708781	A/G	*HLA-DRB1 | HLA-DQA1*	INTERGENIC	2	0.444	0.376	0.524	**5.77E-22**	-	-	-	0.099	0.123	4.22E-01	-	-	-	0.141	0.121	2.49E-01	-	-	-	-0.048	0.074	5.14E-01
rs9276435	6	32821845	A/G	*HLA-DQA2*	INTRON	1	2.590	2.133	3.145	**7.10E-22**	0.017	0.039	6.60E-01	-0.112	0.125	3.74E-01	0.050	0.039	2.00E-01	-0.177	0.123	1.56E-01	-0.036	0.039	3.60E-01	0.087	0.075	2.46E-01
rs1964995	6	32557389	G/A	*HLA-DRA | HLA-DRB5*	INTERGENIC	4	0.498	0.432	0.574	**7.31E-22**	-	-	-	0.110	0.123	3.76E-01	-	-	-	0.160	0.122	1.94E-01	-	-	-	0.004	0.074	9.59E-01
rs3130517	6	31298282	G/A	*HCG27 | HLA-C*	INTERGENIC	2	2.194	1.869	2.576	**7.49E-22**	-	-	-	-0.100	0.124	4.24E-01	-	-	-	-0.150	0.122	2.25E-01	-	-	-	0.089	0.074	2.27E-01
rs156743	6	28075068	G/A	*LOC442175 | ZNF165*	INTERGENIC	2	2.471	2.054	2.972	**7.96E-22**	0.034	0.046	4.60E-01	-0.008	0.123	9.47E-01	0.203	0.046	**8.10E-06**	-0.123	0.122	3.17E-01	-0.181	0.046	**8.60E-05**	-0.053	0.074	4.73E-01
rs200483	6	27882803	A/G	*LOC100131289 | HIST1H2BL*	INTERGENIC	2	2.556	2.110	3.096	**8.60E-22**	0.032	0.049	5.10E-01	0.050	0.123	6.86E-01	0.219	0.048	**5.70E-06**	-0.091	0.122	4.59E-01	-0.206	0.049	**2.40E-05**	-0.038	0.074	6.07E-01
rs370155	6	27890010	C/A	*HIST1H3H | HIST1H2AJ*	INTERGENIC	2	2.555	2.109	3.095	**8.76E-22**	-	-	-	0.050	0.123	6.86E-01	-	-	-	-0.091	0.122	4.59E-01	-	-	-	-0.038	0.074	6.07E-01
rs2523979	6	30191494	A/T	*TRIM31 | TRIM40*	INTERGENIC	2	2.317	1.951	2.752	**9.91E-22**	0.023	0.043	5.90E-01	-0.029	0.123	8.14E-01	0.246	0.042	**5.60E-09**	-0.130	0.121	2.86E-01	-0.243	0.043	**1.30E-08**	-0.031	0.074	6.73E-01
rs2523976	6	30192675	A/T	*TRIM31 | TRIM40*	INTERGENIC	2	2.343	1.967	2.789	**1.20E-21**	0.020	0.043	6.30E-01	-0.021	0.123	8.65E-01	0.246	0.042	**4.80E-09**	-0.144	0.121	2.39E-01	-0.245	0.043	**8.40E-09**	-0.025	0.074	7.36E-01
rs2844790	6	30202092	C/G	*TRIM31 | TRIM40*	INTERGENIC	2	2.353	1.974	2.804	**1.21E-21**	0.021	0.044	6.30E-01	-0.011	0.123	9.32E-01	0.260	0.044	**3.00E-09**	-0.127	0.122	3.02E-01	-0.260	0.044	**4.20E-09**	-0.031	0.074	6.72E-01
rs1003879	6	32407570	A/G	*C6orf10*	INTRON	2	2.142	1.832	2.505	**1.24E-21**	0.074	0.034	**2.80E-02**	-0.047	0.123	7.05E-01	0.037	0.033	2.60E-01	-0.002	0.122	9.90E-01	0.025	0.034	4.60E-01	0.072	0.074	3.32E-01
rs202906	6	28119631	G/A	*LOC442175 | ZNF165*	INTERGENIC	2	2.469	2.050	2.974	**1.54E-21**	0.032	0.046	4.90E-01	-0.008	0.123	9.47E-01	0.202	0.046	**9.30E-06**	-0.123	0.122	3.17E-01	-0.181	0.046	**8.00E-05**	-0.053	0.074	4.73E-01
rs34706883	6	27913234	C/A	*HIST1H4K | HIST1H2AK*	INTERGENIC	2	2.645	2.165	3.230	**1.54E-21**	-	-	-	0.073	0.123	5.54E-01	-	-	-	-0.096	0.122	4.35E-01	-	-	-	-0.018	0.074	8.04E-01
rs13199772	6	27942064	G/A	*HIST1H2AL | HIST1H1B*	INTERGENIC	2	2.663	2.177	3.258	**1.55E-21**	0.063	0.051	2.10E-01	0.073	0.123	5.54E-01	0.248	0.050	**9.40E-07**	-0.096	0.122	4.35E-01	-0.208	0.051	**4.80E-05**	-0.026	0.074	7.30E-01
rs2844627	6	31337441	A/G	*HCG27 | HLA-C*	INTERGENIC	2	2.186	1.862	2.568	**1.56E-21**	0.005	0.036	9.00E-01	0.009	0.123	9.41E-01	0.064	0.036	7.40E-02	-0.116	0.122	3.43E-01	-0.064	0.036	7.70E-02	0.136	0.073	6.56E-02
rs3129840	6	30415321	G/C	*TRIM39*	INTRON	2	2.387	1.996	2.855	**1.57E-21**	0.020	0.042	6.20E-01	-0.045	0.124	7.15E-01	0.196	0.041	**1.90E-06**	-0.165	0.122	1.80E-01	-0.179	0.042	**1.90E-05**	-0.073	0.075	3.28E-01
rs3130473	6	31307187	A/G	*HCG27 | HLA-C*	INTERGENIC	2	2.196	1.867	2.584	**2.40E-21**	0.037	0.036	3.00E-01	-0.083	0.124	5.05E-01	0.108	0.035	**2.30E-03**	-0.197	0.121	1.09E-01	-0.084	0.036	**1.90E-02**	0.112	0.074	1.31E-01
rs13194781	6	27923618	G/A	*HIST1H2BN | HIST1H2AL*	INTERGENIC	2	2.638	2.159	3.224	**2.58E-21**	0.063	0.051	2.10E-01	0.073	0.123	5.54E-01	0.248	0.050	**9.40E-07**	-0.096	0.122	4.35E-01	-0.208	0.051	**4.80E-05**	-0.018	0.074	8.04E-01
rs13212651	6	27914964	G/A	*HIST1H2BN | HIST1H2AL*	INTERGENIC	2	2.638	2.158	3.224	**2.58E-21**	-	-	-	0.073	0.123	5.54E-01	-	-	-	-0.096	0.122	4.35E-01	-	-	-	-0.018	0.074	8.04E-01
rs2523988	6	30187108	G/A	*TRIM31*	INTRON	2	2.317	1.948	2.757	**2.64E-21**	0.019	0.043	6.60E-01	-0.021	0.123	8.65E-01	0.250	0.043	**3.90E-09**	-0.144	0.121	2.39E-01	-0.251	0.043	**5.50E-09**	-0.025	0.074	7.40E-01
rs2523986	6	30189225	A/G	*TRIM31 | TRIM40*	INTERGENIC	2	2.317	1.947	2.757	**2.67E-21**	0.023	0.043	5.90E-01	-0.021	0.123	8.65E-01	0.246	0.042	**5.60E-09**	-0.144	0.121	2.39E-01	-0.243	0.043	**1.30E-08**	-0.025	0.074	7.40E-01
rs2523987	6	30187972	C/A	*TRIM31*	INTRON	2	2.317	1.947	2.757	**2.67E-21**	0.019	0.043	6.60E-01	-0.021	0.123	8.65E-01	0.250	0.043	**3.90E-09**	-0.144	0.121	2.39E-01	-0.251	0.043	**5.50E-09**	-0.025	0.074	7.40E-01
rs149990	6	28106237	A/G	*LOC442175 | ZNF165*	INTERGENIC	2	2.462	2.043	2.967	**2.78E-21**	0.034	0.046	4.60E-01	-0.008	0.123	9.47E-01	0.203	0.046	**8.10E-06**	-0.123	0.122	3.17E-01	-0.181	0.046	**8.60E-05**	-0.053	0.074	4.73E-01
rs3094629	6	30340932	A/G	*FLJ45422*	UTR	2	2.351	1.969	2.806	**3.13E-21**	0.021	0.042	6.20E-01	-0.045	0.124	7.15E-01	0.203	0.041	**8.00E-07**	-0.165	0.122	1.80E-01	-0.186	0.042	**7.80E-06**	-0.073	0.075	3.28E-01
rs3130403	6	30339988	C/A	*FLJ45422*	UTR	2	2.349	1.968	2.804	**3.36E-21**	0.021	0.042	6.20E-01	-0.045	0.124	7.15E-01	0.203	0.041	**8.00E-07**	-0.165	0.122	1.80E-01	-0.186	0.042	**7.80E-06**	-0.073	0.075	3.28E-01
rs3094070	6	30339808	G/C	*FLJ45422*	UTR	2	2.349	1.968	2.804	**3.39E-21**	0.021	0.042	6.20E-01	-0.045	0.124	7.15E-01	0.203	0.041	**8.00E-07**	-0.165	0.122	1.80E-01	-0.186	0.042	**7.80E-06**	-0.073	0.075	3.28E-01
rs3129699	6	30337968	A/G	*FLJ45422*	INTRON	2	2.349	1.968	2.804	**3.39E-21**	-	-	-	-0.045	0.124	7.15E-01	-	-	-	-0.165	0.122	1.80E-01	-	-	-	-0.073	0.075	3.28E-01
rs3129701	6	30340651	A/G	*FLJ45422*	UTR	2	2.349	1.968	2.804	**3.39E-21**	0.021	0.042	6.20E-01	-0.045	0.124	7.15E-01	0.203	0.041	**8.00E-07**	-0.165	0.122	1.80E-01	-0.186	0.042	**7.80E-06**	-0.073	0.075	3.28E-01
rs3129703	6	30341537	A/C	*FLJ45422*	UTR	2	2.349	1.968	2.804	**3.39E-21**	0.021	0.042	6.20E-01	-0.045	0.124	7.15E-01	0.203	0.041	**8.00E-07**	-0.165	0.122	1.80E-01	-0.186	0.042	**7.80E-06**	-0.073	0.075	3.28E-01
rs3129706	6	30342910	G/A	*FLJ45422 | LOC100133303*	INTERGENIC	2	2.349	1.968	2.804	**3.39E-21**	-	-	-	-0.045	0.124	7.15E-01	-	-	-	-0.165	0.122	1.80E-01	-	-	-	-0.073	0.075	3.28E-01
rs3129707	6	30343183	A/G	*FLJ45422 | LOC100133303*	INTERGENIC	2	2.349	1.968	2.804	**3.39E-21**	0.021	0.042	6.20E-01	-0.045	0.124	7.15E-01	0.203	0.041	**8.00E-07**	-0.165	0.122	1.80E-01	-0.186	0.042	**7.80E-06**	-0.073	0.075	3.28E-01
rs3129831	6	30342606	T/A	*FLJ45422*	UTR	2	2.349	1.968	2.804	**3.39E-21**	0.021	0.042	6.20E-01	-0.045	0.124	7.15E-01	0.203	0.041	**8.00E-07**	-0.165	0.122	1.80E-01	-0.186	0.042	**7.80E-06**	-0.073	0.075	3.28E-01
rs3130398	6	30338739	A/G	*FLJ45422*	INTRON	2	2.349	1.968	2.804	**3.39E-21**	0.021	0.042	6.20E-01	-0.045	0.124	7.15E-01	0.203	0.041	**8.00E-07**	-0.165	0.122	1.80E-01	-0.186	0.042	**7.80E-06**	-0.073	0.075	3.28E-01
rs3130400	6	30338756	G/A	*FLJ45422*	INTRON	2	2.349	1.968	2.804	**3.39E-21**	-	-	-	-0.045	0.124	7.15E-01	-	-	-	-0.165	0.122	1.80E-01	-	-	-	-0.073	0.075	3.28E-01
rs3130401	6	30339252	G/A	*FLJ45422*	UTR	2	2.349	1.968	2.804	**3.39E-21**	0.021	0.042	6.20E-01	-0.045	0.124	7.15E-01	0.203	0.041	**8.00E-07**	-0.165	0.122	1.80E-01	-0.186	0.042	**7.80E-06**	-0.073	0.075	3.28E-01
rs3130405	6	30342131	A/G	*FLJ45422*	UTR	2	2.349	1.968	2.804	**3.39E-21**	0.021	0.042	6.20E-01	-0.045	0.124	7.15E-01	0.203	0.041	**8.00E-07**	-0.165	0.122	1.80E-01	-0.186	0.042	**7.80E-06**	-0.073	0.075	3.28E-01
rs3132658	6	30339645	G/A	*FLJ45422*	UTR	2	2.349	1.968	2.804	**3.39E-21**	0.021	0.042	6.20E-01	-0.045	0.124	7.15E-01	0.203	0.041	**8.00E-07**	-0.165	0.122	1.80E-01	-0.186	0.042	**7.80E-06**	-0.073	0.075	3.28E-01
rs3132659	6	30339309	G/A	*FLJ45422*	UTR	2	2.349	1.968	2.804	**3.39E-21**	0.021	0.042	6.20E-01	-0.045	0.124	7.15E-01	0.203	0.041	**8.00E-07**	-0.165	0.122	1.80E-01	-0.186	0.042	**7.80E-06**	-0.073	0.075	3.28E-01
rs3129832	6	30342636	G/A	*FLJ45422*	UTR	2	2.349	1.968	2.804	**3.42E-21**	0.021	0.042	6.20E-01	-0.045	0.124	7.15E-01	0.203	0.041	**8.00E-07**	-0.165	0.122	1.80E-01	-0.186	0.042	**7.80E-06**	-0.073	0.075	3.28E-01
rs3094071	6	30339747	A/G	*FLJ45422*	UTR	2	2.349	1.967	2.804	**3.43E-21**	0.021	0.042	6.20E-01	-0.045	0.124	7.15E-01	0.203	0.041	**8.00E-07**	-0.165	0.122	1.80E-01	-0.186	0.042	**7.80E-06**	-0.073	0.075	3.28E-01
rs3094073	6	30339203	A/G	*FLJ45422*	UTR	2	2.348	1.967	2.803	**3.49E-21**	0.021	0.042	6.20E-01	-0.045	0.124	7.15E-01	0.203	0.041	**8.00E-07**	-0.165	0.122	1.80E-01	-0.186	0.042	**7.80E-06**	-0.073	0.075	3.28E-01
rs3094630	6	30340415	A/G	*FLJ45422*	UTR	2	2.348	1.967	2.803	**3.49E-21**	0.021	0.042	6.20E-01	-0.045	0.124	7.15E-01	0.203	0.041	**8.00E-07**	-0.165	0.122	1.80E-01	-0.186	0.042	**7.80E-06**	-0.073	0.075	3.28E-01
rs3129705	6	30342700	A/G	*FLJ45422*	UTR	2	2.348	1.967	2.803	**3.49E-21**	0.021	0.042	6.20E-01	-0.045	0.124	7.15E-01	0.203	0.041	**8.00E-07**	-0.165	0.122	1.80E-01	-0.186	0.042	**7.80E-06**	-0.073	0.075	3.28E-01
rs2022083	6	30321519	A/T	*TRIM26 | FLJ45422*	INTERGENIC	2	2.354	1.971	2.812	**3.66E-21**	0.018	0.042	6.60E-01	-0.045	0.124	7.15E-01	0.198	0.041	**1.90E-06**	-0.165	0.122	1.80E-01	-0.183	0.042	**1.30E-05**	-0.073	0.075	3.28E-01
rs2517597	6	30189168	A/G	*TRIM31 | TRIM40*	INTERGENIC	2	2.309	1.941	2.747	**3.88E-21**	0.023	0.043	5.90E-01	-0.021	0.123	8.65E-01	0.246	0.042	**5.60E-09**	-0.144	0.121	2.39E-01	-0.243	0.043	**1.30E-08**	-0.025	0.074	7.40E-01
rs3132506	6	31284205	G/A	*HCG27 | HLA-C*	INTERGENIC	2	2.191	1.861	2.579	**4.27E-21**	-	-	-	-0.052	0.124	6.76E-01	-	-	-	-0.193	0.121	1.15E-01	-	-	-	0.129	0.074	8.06E-02
rs1611635	6	29944442	A/G	*LOC100133214 | HLA-H*	INTERGENIC	2	2.367	1.979	2.832	**4.44E-21**	0.017	0.040	6.80E-01	0.001	0.123	9.95E-01	0.162	0.039	**3.90E-05**	-0.143	0.121	2.45E-01	-0.158	0.040	**7.10E-05**	-0.025	0.074	7.40E-01
rs3130467	6	31295054	A/G	*HCG27 | HLA-C*	INTERGENIC	2	2.178	1.852	2.562	**5.09E-21**	-	-	-	-0.100	0.124	4.24E-01	-	-	-	-0.150	0.122	2.25E-01	-	-	-	0.089	0.074	2.27E-01
rs707938	6	31837338	G/A	*MSH5*	SYNONYMOUS	2	2.079	1.785	2.421	**5.25E-21**	0.035	0.034	3.00E-01	-0.044	0.123	7.20E-01	0.066	0.034	5.10E-02	-0.007	0.123	9.58E-01	-0.044	0.034	1.90E-01	-0.069	0.074	3.53E-01
rs3132954	6	32419437	A/G	*C6orf10*	INTRON	2	2.127	1.817	2.490	**5.63E-21**	-	-	-	0.027	0.123	8.27E-01	-	-	-	-0.160	0.121	1.92E-01	-	-	-	-0.025	0.074	7.33E-01
rs3094074	6	30338909	G/A	*FLJ45422*	INTRON	2	2.349	1.966	2.808	**6.14E-21**	-	-	-	-0.045	0.124	7.15E-01	-	-	-	-0.165	0.122	1.80E-01	-	-	-	-0.073	0.075	3.28E-01
rs2523980	6	30191484	G/A	*TRIM31 | TRIM40*	INTERGENIC	2	2.291	1.927	2.724	**6.27E-21**	-	-	-	-0.021	0.123	8.65E-01	-	-	-	-0.144	0.121	2.39E-01	-	-	-	-0.025	0.074	7.40E-01
rs2188099	6	30317897	G/A	*TRIM26 | FLJ45422*	INTERGENIC	2	2.347	1.964	2.806	**6.73E-21**	0.018	0.042	6.60E-01	-0.045	0.124	7.15E-01	0.198	0.041	**1.90E-06**	-0.165	0.122	1.80E-01	-0.183	0.042	**1.30E-05**	-0.073	0.075	3.28E-01
rs2844773	6	30315474	A/C	*TRIM26 | FLJ45422*	INTERGENIC	2	2.347	1.964	2.806	**6.73E-21**	0.018	0.042	6.60E-01	-0.045	0.124	7.15E-01	0.198	0.041	**1.90E-06**	-0.165	0.122	1.80E-01	-0.183	0.042	**1.30E-05**	-0.073	0.075	3.28E-01
rs200484	6	27883653	G/A	*HIST1H2BL*	CODING	2	2.513	2.073	3.046	**6.76E-21**	0.032	0.049	5.10E-01	0.046	0.124	7.14E-01	0.219	0.048	**5.70E-06**	-0.085	0.123	4.93E-01	-0.206	0.049	**2.40E-05**	-0.047	0.075	5.31E-01
rs58547911	6	32519624	A/C	*HLA-DRA*	CODING	2	2.096	1.795	2.448	**7.80E-21**	-	-	-	-0.055	0.123	6.54E-01	-	-	-	-0.228	0.119	6.00E-02	-	-	-	-0.046	0.074	5.32E-01
rs17587	6	32933068	A/G	*PSMB9*	CODING	2	2.119	1.811	2.480	**8.39E-21**	0.105	0.036	**3.50E-03**	0.073	0.123	5.53E-01	0.053	0.036	1.37E-01	-0.060	0.122	6.27E-01	0.038	0.036	2.90E-01	0.031	0.074	6.80E-01
rs204993	6	32263559	G/A	*PBX2*	INTRON	2	2.152	1.833	2.527	**8.51E-21**	0.139	0.035	**6.80E-05**	-0.011	0.124	9.27E-01	0.026	0.034	4.50E-01	-0.121	0.122	3.27E-01	0.093	0.035	**7.10E-03**	0.062	0.075	4.05E-01
rs389883	6	32055439	C/A	*STK19*	INTRON	2	2.094	1.793	2.445	**8.87E-21**	-0.069	0.033	**4.00E-02**	-0.034	0.126	7.89E-01	0.253	0.033	**1.80E-14**	-0.149	0.124	2.32E-01	-0.330	0.033	**4.20E-23**	-0.019	0.075	8.01E-01
rs707928	6	31850569	G/A	*C6orf27*	INTRON	2	2.082	1.785	2.429	**9.75E-21**	0.052	0.035	1.34E-01	-0.094	0.123	4.46E-01	0.076	0.034	**2.70E-02**	-0.039	0.123	7.51E-01	-0.045	0.035	2.00E-01	-0.084	0.074	2.59E-01
rs4502931	6	32488760	A/T	*BTNL2 | HLA-DRA*	INTERGENIC	2	0.469	0.400	0.550	**9.91E-21**	-0.073	0.032	**2.30E-02**	0.019	0.123	8.79E-01	0.146	0.032	**4.30E-06**	0.135	0.121	2.69E-01	-0.223	0.032	**3.70E-12**	-0.014	0.074	8.54E-01
rs2516491	6	31593080	C/A	*MICB | MCCD1*	INTERGENIC	2	2.101	1.797	2.455	**1.03E-20**	-	-	-	-0.138	0.122	2.62E-01	-	-	-	-0.259	0.118	**3.20E-02**	-	-	-	0.005	0.074	9.49E-01
rs2517596	6	30191834	G/C	*TRIM31 | TRIM40*	INTERGENIC	2	2.294	1.927	2.731	**1.03E-20**	-	-	-	-0.021	0.123	8.65E-01	-	-	-	-0.144	0.121	2.39E-01	-	-	-	-0.027	0.074	7.12E-01

rs3130552	6	31190106	A/G	C6orf15 | PSORS1C1	INTERGENIC	2	2.080	1.784	2.427	**1.07E-20**	-0.011	0.033	7.40E-01	-0.105	0.124	3.99E-01	0.078	0.033	**1.80E-02**	-0.117	0.123	3.46E-01	-0.085	0.033	**1.04E-02**	-0.050	0.074	5.04E-01
rs4642516	6	32765521	A/C	HLA-DQB1 | HLA-DQA2	INTERGENIC	2	0.433	0.363	0.516	**1.10E-20**	-	-	-	0.037	0.123	7.65E-01	-	-	-	0.253	0.118	**3.60E-02**	-	-	-	-0.054	0.074	4.68E-01
rs2523981	6	30191161	A/C	TRIM31 | TRIM40	INTERGENIC	2	2.292	1.925	2.729	**1.15E-20**	-	-	-	-0.021	0.123	8.65E-01	-	-	-	-0.144	0.121	2.39E-01	-	-	-	-0.025	0.074	7.40E-01
rs2523983	6	30190200	A/C	TRIM31 | TRIM40	INTERGENIC	2	2.284	1.919	2.717	**1.22E-20**	0.023	0.043	5.90E-01	-0.021	0.123	8.65E-01	0.246	0.042	**5.60E-09**	-0.144	0.121	2.39E-01	-0.243	0.043	**1.30E-08**	-0.027	0.074	7.12E-01
rs3130404	6	30340229	G/A	FLJ45422	UTR	2	2.326	1.947	2.778	**1.30E-20**	0.021	0.042	6.20E-01	-0.071	0.125	5.70E-01	0.203	0.041	**8.00E-07**	-0.163	0.123	1.90E-01	-0.186	0.042	**7.80E-06**	-0.083	0.075	2.66E-01
rs630379	6	32030233	A/C	RDBP	INTRON	2	2.104	1.798	2.461	**1.58E-20**	-0.062	0.033	6.50E-02	-0.071	0.126	5.75E-01	0.259	0.033	**5.00E-15**	-0.161	0.124	2.00E-01	-0.329	0.033	**4.40E-23**	-0.025	0.076	7.38E-01
rs2394894	6	31314899	G/A	HCG27 | HLA-C	INTERGENIC	2	2.179	1.849	2.569	**1.68E-20**	0.034	0.036	3.40E-01	-0.052	0.124	6.76E-01	0.113	0.036	**1.50E-03**	-0.193	0.121	1.15E-01	-0.092	0.036	**1.11E-02**	0.130	0.074	7.86E-02
rs2394895	6	31314958	G/A	HCG27 | HLA-C	INTERGENIC	2	2.179	1.849	2.569	**1.68E-20**	0.034	0.036	3.40E-01	-0.052	0.124	6.76E-01	0.113	0.036	**1.50E-03**	-0.193	0.121	1.15E-01	-0.092	0.036	**1.11E-02**	0.130	0.074	7.86E-02
rs6904596	6	27599278	A/G	ZNF184 | LOC100131289	INTERGENIC	2	2.576	2.109	3.146	**1.68E-20**	0.066	0.050	1.90E-01	0.050	0.123	6.86E-01	0.242	0.050	**1.20E-06**	-0.091	0.122	4.59E-01	-0.200	0.050	**7.50E-05**	-0.058	0.074	4.32E-01
rs130071	6	31224189	A/G	CCHCR1	CODING	2	2.111	1.803	2.471	**1.72E-20**	-	-	-	-0.042	0.123	7.35E-01	-	-	-	-0.200	0.120	1.01E-01	-	-	-	0.048	0.074	5.13E-01
rs149943	6	28110367	A/G	LOC442175 | ZNF165	INTERGENIC	2	2.368	1.974	2.842	**1.87E-20**	0.034	0.046	4.60E-01	-0.008	0.123	9.47E-01	0.203	0.046	**8.10E-06**	-0.123	0.122	3.17E-01	-0.181	0.046	**8.60E-05**	-0.057	0.074	4.43E-01
rs9261630	6	30335894	T/A	FLJ45422	INTRON	2	2.316	1.939	2.766	**2.05E-20**	-	-	-	-0.045	0.124	7.15E-01	-	-	-	-0.165	0.122	1.80E-01	-	-	-	-0.073	0.075	3.28E-01
rs3135392	6	32517220	A/C	HLA-DRA	INTRON	2	2.066	1.772	2.409	**2.26E-20**	0.068	0.033	**3.80E-02**	-0.098	0.123	4.25E-01	0.004	0.033	9.00E-01	-0.237	0.119	**4.99E-02**	0.051	0.033	1.22E-01	-0.014	0.074	8.53E-01
rs419788	6	32036778	A/G	SKIV2L	INTRON	2	2.091	1.788	2.445	**2.34E-20**	-0.062	0.033	6.50E-02	-0.034	0.126	7.89E-01	0.259	0.033	**5.00E-15**	-0.149	0.124	2.32E-01	-0.329	0.033	**4.40E-23**	-0.025	0.076	7.38E-01
rs2854008	6	31420517	A/G	HLA-C | HLA-B	INTERGENIC	1	2.390	1.987	2.875	**2.47E-20**	0.004	0.035	9.10E-01	-0.180	0.124	1.52E-01	0.085	0.034	**1.36E-02**	-0.193	0.123	1.22E-01	-0.079	0.035	**2.20E-02**	-0.060	0.076	4.27E-01
rs10484399	6	27642507	G/A	ZNF184 | LOC100131289	INTERGENIC	2	2.589	2.116	3.168	**2.57E-20**	0.071	0.051	1.60E-01	0.073	0.123	5.54E-01	0.251	0.050	**6.60E-07**	-0.096	0.122	4.35E-01	-0.205	0.051	**6.20E-05**	-0.018	0.074	8.04E-01
rs3095151	6	31008129	A/C	SFTPG | LOC100129065	INTERGENIC	1	2.430	2.013	2.934	**2.57E-20**	-0.014	0.038	7.10E-01	-0.078	0.123	5.25E-01	0.106	0.038	**5.00E-03**	-0.143	0.121	2.41E-01	-0.125	0.038	**1.10E-03**	0.017	0.074	8.21E-01
rs2734986	6	29926547	G/A	LOC100133214 | HLA-H	INTERGENIC	2	2.320	1.941	2.774	**2.61E-20**	0.006	0.041	8.80E-01	0.001	0.123	9.95E-01	0.179	0.041	**1.00E-05**	-0.143	0.121	2.45E-01	-0.195	0.041	**1.80E-06**	-0.025	0.074	7.40E-01
rs7754768	6	32528157	G/A	HLA-DRA | HLA-DRB5	INTERGENIC	2	2.088	1.785	2.442	**3.09E-20**	-	-	-	-0.042	0.123	7.35E-01	-	-	-	-0.239	0.119	**4.93E-02**	-	-	-	-0.041	0.074	5.78E-01
rs440454	6	32035321	A/G	SKIV2L	INTRON	2	2.090	1.787	2.446	**3.34E-20**	-0.062	0.033	6.50E-02	-0.034	0.126	7.89E-01	0.259	0.033	**5.00E-15**	-0.149	0.124	2.32E-01	-0.329	0.033	**4.40E-23**	-0.025	0.076	7.38E-01
rs1015466	6	30193895	T/A	TRIM31 | TRIM40	INTERGENIC	2	2.266	1.904	2.698	**3.70E-20**	0.020	0.043	6.30E-01	-0.021	0.123	8.65E-01	0.246	0.042	**4.80E-09**	-0.144	0.121	2.39E-01	-0.245	0.043	**8.40E-09**	-0.023	0.074	7.56E-01
rs1541269	6	30211339	A/G	TRIM31 | TRIM40	INTERGENIC	2	2.300	1.926	2.748	**3.86E-20**	0.031	0.045	4.90E-01	-0.011	0.123	9.32E-01	0.271	0.044	**7.40E-10**	-0.127	0.122	3.02E-01	-0.264	0.045	**3.10E-09**	-0.030	0.074	6.84E-01
rs3130977	6	31189968	G/A	C6orf15 | PSORS1C1	INTERGENIC	2	2.052	1.759	2.395	**6.51E-20**	-0.013	0.033	6.90E-01	-0.127	0.124	3.09E-01	0.082	0.033	**1.26E-02**	-0.131	0.123	2.89E-01	-0.091	0.033	**5.60E-03**	-0.056	0.074	4.50E-01
rs644045	6	31991936	A/G	ZBTB12 | C2	INTERGENIC	2	2.051	1.758	2.393	**7.07E-20**	-0.032	0.032	3.20E-01	-0.011	0.124	9.32E-01	0.211	0.032	**4.10E-11**	-0.066	0.123	5.91E-01	-0.258	0.032	**1.30E-15**	0.056	0.075	4.51E-01
rs9268832	6	32535767	A/G	HLA-DRA | HLA-DRB5	INTERGENIC	2	2.069	1.770	2.419	**7.69E-20**	-0.091	0.032	**4.70E-03**	-0.042	0.123	7.35E-01	0.139	0.032	**1.40E-05**	-0.239	0.119	**4.93E-02**	-0.230	0.032	**9.70E-13**	-0.041	0.074	5.78E-01
rs7195	6	32520517	A/G	HLA-DRA	UTR	2	2.073	1.772	2.425	**8.09E-20**	-0.098	0.032	**2.50E-03**	-0.055	0.123	6.54E-01	0.133	0.032	**3.20E-05**	-0.228	0.119	6.00E-02	-0.228	0.032	**1.40E-12**	-0.046	0.074	5.35E-01
rs2213585	6	32521128	G/A	HLA-DRA | HLA-DRB5	INTERGENIC	2	2.072	1.772	2.424	**8.41E-20**	-0.098	0.032	**2.50E-03**	-0.055	0.123	6.54E-01	0.133	0.032	**3.20E-05**	-0.228	0.119	6.00E-02	-0.228	0.032	**1.40E-12**	-0.046	0.074	5.35E-01
rs2227139	6	32521437	G/A	HLA-DRA | HLA-DRB5	INTERGENIC	2	2.072	1.772	2.424	**8.41E-20**	-0.098	0.032	**2.60E-03**	-0.055	0.123	6.54E-01	0.135	0.032	**2.60E-05**	-0.228	0.119	6.00E-02	-0.230	0.032	**1.10E-12**	-0.046	0.074	5.35E-01
rs2213586	6	32521072	A/G	HLA-DRA | HLA-DRB5	INTERGENIC	2	2.072	1.771	2.424	**8.58E-20**	-0.098	0.032	**2.50E-03**	-0.055	0.123	6.54E-01	0.133	0.032	**3.20E-05**	-0.228	0.119	6.00E-02	-0.228	0.032	**1.40E-12**	-0.046	0.074	5.35E-01
rs9272219	6	32710247	A/C	HLA-DRB1 | HLA-DQA1	INTERGENIC	2	2.071	1.770	2.424	**1.00E-19**	0.086	0.036	**1.80E-02**	-0.106	0.126	4.04E-01	0.142	0.036	**7.70E-05**	-0.041	0.126	7.47E-01	-0.079	0.036	**2.90E-02**	-0.008	0.075	9.11E-01
rs9273012	6	32719619	G/A	HLA-DQA1 | HLA-DQB1	INTERGENIC	2	2.071	1.770	2.424	**1.00E-19**	-	-	-	-0.106	0.126	4.04E-01	-	-	-	-0.041	0.126	7.47E-01	-	-	-	-0.008	0.075	9.11E-01
rs5007259	6	32487079	G/A	BTNL2 | HLA-DRA	INTERGENIC	2	0.458	0.387	0.543	**1.09E-19**	-0.073	0.032	**2.30E-02**	0.019	0.123	8.79E-01	0.146	0.032	**4.30E-06**	0.135	0.121	2.69E-01	-0.223	0.032	**3.70E-12**	-0.014	0.074	8.54E-01
rs805294	6	31796196	G/A	LY6G6C	INTRON	2	2.015	1.732	2.345	**1.09E-19**	0.047	0.034	1.70E-01	-0.112	0.122	3.64E-01	0.054	0.034	1.05E-01	-0.049	0.122	6.88E-01	-0.019	0.034	5.80E-01	-0.060	0.074	4.19E-01
rs6926737	6	32483723	A/G	BTNL2 | HLA-DRA	INTERGENIC	2	0.459	0.387	0.543	**1.10E-19**	0.073	0.032	**2.30E-02**	0.019	0.123	8.79E-01	-0.133	0.032	**2.50E-05**	0.135	0.121	2.69E-01	0.208	0.032	**6.00E-11**	-0.014	0.074	8.54E-01
rs7749305	6	27554545	G/A	ZNF184 | LOC100131289	INTERGENIC	2	2.497	2.050	3.043	**1.11E-19**	-	-	-	0.050	0.123	6.86E-01	-	-	-	-0.091	0.122	4.59E-01	-	-	-	-0.056	0.074	4.51E-01
rs5007263	6	32486960	G/A	BTNL2 | HLA-DRA	INTERGENIC	2	0.459	0.388	0.543	**1.12E-19**	-0.076	0.032	**1.90E-02**	0.019	0.123	8.79E-01	0.146	0.032	**4.80E-06**	0.135	0.121	2.69E-01	-0.225	0.032	**2.60E-12**	-0.014	0.074	8.54E-01
rs5007265	6	32486844	C/A	BTNL2 | HLA-DRA	INTERGENIC	2	0.459	0.388	0.543	**1.12E-19**	-0.065	0.032	**4.20E-02**	0.019	0.123	8.79E-01	0.146	0.032	**4.20E-06**	0.135	0.121	2.69E-01	-0.216	0.032	**1.60E-11**	-0.014	0.074	8.54E-01
rs6932542	6	32488240	G/A	BTNL2 | HLA-DRA	INTERGENIC	2	0.459	0.388	0.543	**1.12E-19**	-0.073	0.032	**2.30E-02**	0.019	0.123	8.79E-01	0.146	0.032	**4.30E-06**	0.135	0.121	2.69E-01	-0.223	0.032	**3.70E-12**	-0.014	0.074	8.54E-01
rs9268507	6	32485517	G/A	BTNL2 | HLA-DRA	INTERGENIC	2	0.459	0.388	0.543	**1.12E-19**	-0.065	0.032	**4.20E-02**	0.019	0.123	8.79E-01	0.146	0.032	**4.20E-06**	0.135	0.121	2.69E-01	-0.216	0.032	**1.60E-11**	-0.014	0.074	8.54E-01
rs13195040	6	27521903	G/A	ZNF391 / ZNF184	INTERGENIC	2	2.556	2.087	3.130	**1.15E-19**	0.046	0.051	3.70E-01	0.073	0.123	5.54E-01	0.243	0.051	**1.80E-06**	-0.096	0.122	4.35E-01	-0.215	0.052	**3.00E-05**	-0.038	0.075	6.10E-01
rs805304	6	31806067	C/A	DDAH2 | CLIC1	INTERGENIC	2	2.028	1.740	2.362	**1.23E-19**	0.047	0.034	1.70E-01	-0.112	0.122	3.64E-01	0.054	0.034	1.05E-01	-0.049	0.122	6.88E-01	-0.019	0.034	5.80E-01	-0.063	0.074	3.96E-01
rs1144708	6	31817999	A/G	MSH5	INTRON	2	2.027	1.740	2.362	**1.27E-19**	-	-	-	-0.112	0.122	3.64E-01	-	-	-	-0.049	0.122	6.88E-01	-	-	-	-0.063	0.074	3.96E-01
rs707916	6	31805537	A/G	DDAH2	INTRON	2	2.027	1.740	2.362	**1.29E-19**	0.047	0.034	1.70E-01	-0.112	0.122	3.64E-01	0.054	0.034	1.05E-01	-0.049	0.122	6.88E-01	-0.019	0.034	5.80E-01	-0.063	0.074	3.98E-01
rs805305	6	31805366	C/G	DDAH2	INTRON	2	2.027	1.740	2.361	**1.30E-19**	0.047	0.034	1.70E-01	-0.112	0.122	3.64E-01	0.054	0.034	1.05E-01	-0.049	0.122	6.88E-01	-0.019	0.034	5.80E-01	-0.063	0.074	3.96E-01
rs9268831	6	32535726	A/G	HLA-DRA | HLA-DRB5	INTERGENIC	2	0.471	0.400	0.555	**1.65E-19**	0.078	0.032	**1.48E-02**	-0.028	0.123	8.23E-01	-0.124	0.032	**9.50E-05**	0.209	0.120	8.48E-02	0.209	0.032	**6.30E-11**	0.003	0.074	9.63E-01
rs3130556	6	31202615	A/G	PSORS1C1	INTRON	2	2.041	1.747	2.383	**1.96E-19**	-	-	-	-0.102	0.123	4.09E-01	-	-	-	-0.115	0.122	3.47E-01	-	-	-	-0.021	0.074	7.81E-01
rs9468413	6	28797651	C/A	LOC646160 | LOC442181	INTERGENIC	2	2.307	1.923	2.769	**2.68E-19**	0.015	0.042	7.20E-01	-0.043	0.124	7.27E-01	0.099	0.042	**1.80E-02**	-0.122	0.122	3.20E-01	-0.077	0.042	6.80E-02	-0.033	0.074	6.61E-01
rs4394270	6	32817942	G/A	HLA-DQA2	INTRON	1	2.200	1.852	2.613	**2.70E-19**	-	-	-	-0.036	0.123	7.70E-01	-	-	-	-0.054	0.122	6.62E-01	-	-	-	0.036	0.074	6.27E-01
rs6456834	6	28808331	A/C	LOC442181	UTR	2	2.306	1.922	2.768	**2.78E-19**	0.015	0.042	7.20E-01	-0.043	0.124	7.27E-01	0.099	0.042	**1.80E-02**	-0.122	0.122	3.20E-01	-0.077	0.042	6.80E-02	-0.033	0.074	6.61E-01
rs910050	6	32423632	C/G	C6orf10	INTRON	2	2.006	1.723	2.336	**2.95E-19**	-	-	-	0.012	0.123	9.23E-01	-	-	-	-0.118	0.121	3.34E-01	-	-	-	-0.016	0.074	8.29E-01
rs3117122	6	32424582	A/G	C6orf10	INTRON	2	2.014	1.729	2.348	**2.96E-19**	-	-	-	0.012	0.123	9.23E-01	-	-	-	-0.118	0.121	3.34E-01	-	-	-	-0.016	0.074	8.29E-01
rs2076537	6	32425613	A/G	C6orf10	INTRON	2	2.014	1.728	2.347	**3.01E-19**	0.040	0.033	2.30E-01	0.012	0.123	9.23E-01	0.070	0.033	**3.20E-02**	-0.118	0.121	3.34E-01	-0.036	0.033	2.80E-01	-0.016	0.074	8.29E-01
rs761188	6	32425951	G/A	C6orf10	INTRON	2	2.014	1.728	2.347	**3.01E-19**	0.040	0.033	2.30E-01	0.012	0.123	9.23E-01	0.070	0.033	**3.20E-02**	-0.118	0.121	3.34E-01	-0.036	0.033	2.80E-01	-0.016	0.074	8.29E-01
rs969893	6	32424059	A/C	C6orf10	INTRON	2	2.014	1.728	2.347	**3.07E-19**	-	-	-	0.012	0.123	9.23E-01	-	-	-	-0.118	0.121	3.34E-01	-	-	-	-0.016	0.074	8.29E-01
rs1633005	6	29872451	A/G	HCG4 | HLA-G	INTERGENIC	2	2.236	1.875	2.667	**3.41E-19**	0.005	0.040	9.10E-01	-0.035	0.123	7.79E-01	0.158	0.039	**5.80E-05**	-0.157	0.121	1.99E-01	-0.167	0.040	**2.70E-05**	-0.004	0.074	9.59E-01
rs521977	6	31944806	A/C	SLC44A4	INTRON	2	2.039	1.744	2.384	**4.24E-19**	-	-	-	-0.043	0.125	7.32E-01	-	-	-	-0.196	0.122	1.12E-01	-	-	-	-0.012	0.075	8.72E-01
rs406511	6	29484364	C/G	OR12D2 | OR11A1	INTERGENIC	1	2.710	2.176	3.374	**5.05E-19**	0.025	0.047	5.90E-01	0.035	0.124	7.81E-01	0.221	0.046	**1.60E-06**	-0.133	0.122	2.82E-01	-0.205	0.047	**1.10E-05**	-0.035	0.074	6.35E-01
rs6934429	6	32387600	A/C	C6orf10	INTRON	1	2.200	1.849	2.618	**6.58E-19**	0.109	0.038	**4.40E-03**	-0.108	0.122	3.82E-01	0.116	0.038	**2.30E-03**	-0.214	0.119	7.73E-02	-0.031	0.038	4.20E-01	0.012	0.074	8.75E-01
rs915895	6	32298195	G/A	NOTCH4	INTRON	2	2.008	1.721	2.343	**7.33E-19**	0.101	0.034	**2.90E-03**	-0.009	0.124	9.40E-01	-0.022	0.033	5.20E-01	-0.017	0.123	8.93E-01	0.110	0.034	**1.06E-03**	0.029	0.075	7.04E-01
rs3130944	6	31304650	C/G	HCG27 | HLA-C	INTERGENIC	1	2.300	1.913	2.765	**7.37E-19**	0.038	0.036	2.90E-01	-0.083	0.124	5.05E-01	0.109	0.035	**2.00E-03**	-0.197	0.121	1.09E-01	-0.085	0.036	**1.70E-02**	0.112	0.074	1.31E-01
rs3095325	6	31194976	A/G	CDSN	INTRON	2	2.002	1.717	2.334	**7.52E-19**	-	-	-	-0.108	0.122	3.82E-01	-	-	-	0.042	0.122	7.30E-01	-	-	-	-0.069	0.074	3.52E-01
rs537160	6	32024379	A/G	CFB	INTRON	2	2.002	1.717	2.334	**7.55E-19**	-0.057	0.033	8.30E-02	-0.037	0.127	7.71E-01	0.197	0.032	**1.10E-09**	-0.153	0.125	2.23E-01	-0.257	0.033	**2.70E-15**	-0.019	0.076	8.02E-01
rs2239520	6	31196901	A/G	CDSN | PSORS1C1	INTERGENIC	2	2.002	1.717	2.334	**7.60E-19**	-0.015	0.032	6.50E-01	-0.108	0.122	3.82E-01	0.043	0.032	1.80E-01	0.042	0.122	7.30E-01	-0.051	0.032	1.16E-01	-0.069	0.074	3.52E-01
rs3130989	6	31194356	C/G	CDSN	INTRON	2	2.001	1.717	2.333	**7.61E-19**	-0.014	0.032	6.70E-01	-0.108	0.122	3.82E-01	0.044	0.032	1.70E-01	0.042	0.122	7.30E-01	-0.051	0.032	1.12E-01	-0.069	0.074	3.52E-01
rs3130979	6	31190176	G/C	C6orf15 | PSORS1C1	INTERGENIC	2	2.005	1.719	2.339	**8.37E-19**	-0.011	0.033	7.40E-01	-0.069	0.124	5.78E-01	0.078	0.033	**1.80E-02**	-0.115	0.123	3.54E-01	-0.085	0.033	**1.04E-02**	-0.028	0.074	7.02E-01
rs2853961	6	31339968	A/G	HCG27 | HLA-C	INTERGENIC	2	2.019	1.728	2.359	**8.88E-19**	-0.033	0.033	3.30E-01	-0.057	0.123	6.46E-01	0.022	0.033	5.00E-01	-0.027	0.122	8.24E-01	-0.049	0.033	1.43E-01	0.032	0.074	6.68E-01
rs17693963	6	27818144	C/A	ZNF184 | LOC100131289	INTERGENIC	2	2.444	2.005	2.979	**8.95E-19**	0.067	0.051	1.90E-01	0.149	0.122	2.27E-01	0.250	0.050	**6.50E-07**	0.138	0.121	2.58E-01	-0.207	0.051	**4.70E-05**	-0.034	0.074	6.53E-01
rs9257793	6	29443133	G/A	OR5V1 | OR12D3	INTERGENIC	2	2.395	1.973	2.906	**9.26E-19**	0.025	0.049	6.00E-01	0.087	0.129	5.03E-01	0.229	0.048	**1.70E-06**	-0.097	0.129	4.54E-01	-0.224	0.048	**4.00E-06**	-0.003	0.077	9.70E-01
rs2596560	6	31463297	G/A	LOC729816 | LOC100129668	INTERGENIC	1	2.220	1.860	2.650	**1.03E-18**	0.017	0.038	6.60E-01	-0.045	0.124	7.17E-01	0.144	0.038	**1.50E-04**	-0.188	0.121	1.24E-01	-0.134	0.038	**4.50E-04**	-0.045	0.075	5.50E-01

rs1480383	6	32848834	C/A	*HLA-DQB2 | HLA-DOB*	INTERGENIC	2	2.020	1.728	2.361	**1.11E-18**	0.076	0.036	**3.80E-02**	-0.017	0.125	8.90E-01	0.082	0.036	**2.20E-02**	-0.154	0.122	2.12E-01	-0.016	0.036	6.60E-01	-0.012	0.075	8.75E-01
rs3095314	6	31197610	A/G	*CDSN | PSORS1C1*	INTERGENIC	2	1.994	1.710	2.324	**1.21E-18**	-0.015	0.032	6.50E-01	-0.108	0.122	3.82E-01	0.043	0.032	1.80E-01	0.042	0.122	7.30E-01	-0.051	0.032	1.16E-01	-0.069	0.074	3.52E-01
rs2523443	6	29523443	A/C	*OR10C1 | OR2H1*	INTERGENIC	1	2.700	2.165	3.367	**1.23E-18**	0.000	0.046	1.00E+00	0.035	0.124	7.81E-01	0.209	0.046	**5.50E-06**	-0.133	0.122	2.82E-01	-0.213	0.047	**4.90E-06**	-0.027	0.074	7.13E-01
rs3095317	6	31196650	G/A	*CDSN | PSORS1C1*	INTERGENIC	2	1.994	1.709	2.326	**1.66E-18**	-	-	-	-0.108	0.122	3.82E-01	-	-	-	0.042	0.122	7.30E-01	-	-	-	-0.069	0.074	3.52E-01
rs2523992	6	30183082	C/A	*TRIM31*	INTRON	2	2.146	1.809	2.544	**1.67E-18**	0.033	0.041	4.30E-01	-0.019	0.123	8.75E-01	0.248	0.041	**1.30E-09**	-0.078	0.122	5.23E-01	-0.237	0.041	**1.00E-08**	-0.038	0.074	6.10E-01
rs2763979	6	31902571	A/G	*HSPA1A | HSPA1B*	INTERGENIC	2	1.980	1.699	2.307	**2.24E-18**	0.005	0.034	8.70E-01	-0.140	0.123	2.58E-01	0.058	0.033	8.30E-02	-0.092	0.123	4.56E-01	-0.053	0.034	1.17E-01	-0.082	0.074	2.68E-01
rs9268135	6	32363208	G/A	*NOTCH4 | C6orf10*	INTERGENIC	1	2.170	1.824	2.582	**2.26E-18**	0.135	0.070	5.30E-02	-0.108	0.122	3.82E-01	-0.055	0.070	4.30E-01	-0.214	0.119	7.73E-02	0.167	0.070	**1.70E-02**	0.012	0.074	8.75E-01
rs443198	6	32298384	G/A	*NOTCH4*	CODING	2	1.994	1.708	2.328	**2.47E-18**	0.079	0.033	**1.60E-02**	-0.045	0.124	7.19E-01	-0.062	0.032	5.40E-02	0.074	0.123	5.49E-01	0.143	0.032	**1.10E-05**	0.009	0.075	9.01E-01
rs2858867	6	32683303	G/A	*HLA-DRB1 | HLA-DQA1*	INTERGENIC	2	1.994	1.707	2.329	**3.02E-18**	-	-	-	-0.034	0.125	7.87E-01	-	-	-	-0.043	0.124	7.27E-01	-	-	-	-0.010	0.075	8.91E-01
rs3948793	6	32867426	A/G	*HLA-DQB2 | HLA-DOB*	INTERGENIC	2	1.952	1.678	2.270	**3.91E-18**	0.093	0.034	**5.70E-03**	0.010	0.126	9.38E-01	0.057	0.033	8.60E-02	-0.184	0.123	1.40E-01	0.023	0.033	4.80E-01	0.022	0.075	7.65E-01
rs7745040	6	32772310	G/A	*HLA-DQB1 | HLA-DQA2*	INTERGENIC	2	0.476	0.402	0.563	**3.97E-18**	0.123	0.033	**1.70E-04**	0.010	0.123	9.33E-01	0.001	0.032	9.80E-01	0.101	0.122	4.10E-01	0.106	0.032	**1.14E-03**	-0.037	0.074	6.13E-01
rs1264587	6	30387053	C/G	*LOC100133303*	INTRON	2	2.108	1.781	2.495	**4.24E-18**	-	-	-	-0.013	0.124	9.16E-01	-	-	-	-0.146	0.122	2.36E-01	-	-	-	-0.062	0.075	4.08E-01
rs853685	6	28396764	A/G	*PGBD1 | ZNF323*	INTERGENIC	2	2.265	1.882	2.725	**5.07E-18**	0.019	0.043	6.70E-01	0.032	0.124	7.99E-01	0.152	0.043	**4.00E-04**	-0.059	0.123	6.32E-01	-0.146	0.043	**7.50E-04**	-0.045	0.074	5.47E-01
rs9268213	6	32390059	G/A	*C6orf10*	INTRON	1	2.160	1.814	2.572	**5.10E-18**	-	-	-	-0.108	0.122	3.82E-01	-	-	-	-0.214	0.119	7.73E-02	-	-	-	0.012	0.074	8.75E-01
rs1264622	6	30364915	A/G	*FLJ45422 | LOC100133303*	INTERGENIC	2	2.108	1.780	2.497	**5.70E-18**	0.000	0.040	1.00E+00	-0.013	0.124	9.16E-01	0.189	0.039	**1.40E-06**	-0.146	0.122	2.36E-01	-0.203	0.040	**2.90E-07**	-0.060	0.075	4.27E-01
rs2050190	6	32447054	G/A	*C6orf10*	INTRON	2	1.958	1.681	2.281	**5.87E-18**	0.081	0.034	**1.90E-02**	-0.050	0.123	6.84E-01	0.079	0.034	**2.00E-02**	-0.038	0.122	7.59E-01	-0.023	0.034	5.10E-01	0.070	0.074	3.42E-01
rs9272105	6	32707977	G/A	*HLA-DRB1 | HLA-DQA1*	INTERGENIC	2	0.493	0.420	0.579	**6.13E-18**	-	-	-	0.105	0.122	3.94E-01	-	-	-	0.142	0.121	2.46E-01	-	-	-	-0.031	0.074	6.76E-01
rs2523554	6	31439808	G/A	*HLA-B | LOC729816*	INTERGENIC	2	1.968	1.687	2.295	**7.04E-18**	-0.040	0.032	2.10E-01	0.016	0.125	8.96E-01	0.241	0.032	**2.40E-14**	-0.066	0.124	5.94E-01	-0.287	0.032	**2.00E-19**	0.032	0.076	6.76E-01
rs9276644	6	32853021	G/A	*HLA-DQB2 | HLA-DOB*	INTERGENIC	2	1.954	1.678	2.277	**7.44E-18**	0.095	0.034	**4.90E-03**	0.010	0.126	9.38E-01	0.056	0.033	9.30E-02	-0.184	0.123	1.40E-01	0.026	0.033	4.30E-01	0.022	0.075	7.65E-01
rs412657	6	32319063	C/A	*NOTCH4 | C6orf10*	INTERGENIC	2	1.959	1.679	2.285	**1.12E-17**	0.069	0.034	**3.90E-02**	0.009	0.123	9.39E-01	-0.010	0.033	7.80E-01	-0.002	0.123	9.87E-01	0.071	0.034	**3.50E-02**	0.008	0.075	9.20E-01
rs9276612	6	32850690	A/G	*HLA-DQB2 | HLA-DOB*	INTERGENIC	2	1.957	1.677	2.283	**1.40E-17**	0.100	0.034	**3.40E-03**	0.010	0.126	9.38E-01	0.057	0.034	9.20E-02	-0.184	0.123	1.40E-01	0.032	0.034	3.50E-01	0.022	0.075	7.65E-01
rs2517544	6	31117487	C/G	*LOC729792*	INTRON	2	1.955	1.676	2.281	**1.54E-17**	0.015	0.034	6.60E-01	-0.095	0.125	4.50E-01	0.067	0.034	**4.70E-02**	-0.125	0.124	3.16E-01	-0.051	0.034	1.35E-01	-0.008	0.075	9.15E-01
rs7341328	6	32383172	A/G	*C6orf10*	INTRON	1	2.150	1.803	2.564	**1.61E-17**	0.109	0.038	**4.40E-03**	-0.108	0.122	3.82E-01	0.116	0.038	**2.30E-03**	-0.214	0.119	7.73E-02	-0.031	0.038	4.20E-01	0.012	0.074	8.75E-01
rs429916	6	33086565	A/C	*HLA-DOA | HLA-DPA1*	INTERGENIC	2	2.600	2.086	3.241	**1.86E-17**	0.138	0.061	**2.30E-02**	-0.068	0.123	5.82E-01	0.034	0.060	5.70E-01	-0.049	0.122	6.90E-01	0.095	0.061	1.19E-01	-0.061	0.074	4.11E-01
rs2844571	6	31443626	G/A	*LOC729816 | LOC100129668*	INTERGENIC	2	1.921	1.652	2.233	**2.02E-17**	0.061	0.034	7.80E-02	-0.020	0.126	8.73E-01	0.071	0.034	**3.80E-02**	-0.111	0.124	3.74E-01	-0.018	0.034	6.00E-01	0.001	0.075	9.90E-01
rs9276909	6	32958817	A/G	*PSMB9 | HLA-DMB*	INTERGENIC	1	2.120	1.782	2.522	**2.39E-17**	0.103	0.036	**4.30E-03**	0.073	0.123	5.53E-01	0.049	0.036	1.70E-01	-0.060	0.122	6.27E-01	0.040	0.036	2.70E-01	0.031	0.074	6.80E-01
rs2844697	6	31040288	A/G	*LOC100129065 | C6orf205*	INTERGENIC	2	1.919	1.650	2.231	**2.42E-17**	0.011	0.034	7.40E-01	-0.040	0.123	7.47E-01	0.038	0.034	2.60E-01	-0.128	0.122	2.98E-01	-0.025	0.034	4.70E-01	-0.018	0.075	8.09E-01
rs853676	6	28407666	A/G	*ZNF323*	INTRON	2	2.209	1.839	2.653	**2.46E-17**	0.016	0.044	7.10E-01	0.032	0.124	7.99E-01	0.157	0.043	**2.70E-04**	-0.059	0.123	6.32E-01	-0.153	0.044	**4.40E-04**	-0.045	0.074	5.47E-01
rs746647	6	31222161	G/A	*CCHCR1*	INTRON	2	1.966	1.681	2.299	**2.60E-17**	-0.014	0.034	6.90E-01	-0.061	0.124	6.24E-01	0.101	0.034	**3.10E-03**	-0.189	0.121	1.23E-01	-0.117	0.034	**6.40E-04**	0.058	0.074	4.36E-01
rs1610682	6	29909303	G/A	*LOC100133214 | HLA-H*	INTERGENIC	2	2.042	1.730	2.411	**3.31E-17**	-0.022	0.037	5.50E-01	-0.039	0.123	7.52E-01	0.129	0.036	**4.10E-04**	-0.188	0.120	1.23E-01	-0.155	0.037	**2.60E-05**	0.004	0.074	9.56E-01
rs3757188	6	28215336	G/A	*LOC100129195*	UTR	2	2.211	1.838	2.659	**3.43E-17**	-	-	-	0.106	0.123	3.94E-01	-	-	-	-0.016	0.123	8.96E-01	-	-	-	-0.060	0.074	4.16E-01
rs9380064	6	28251097	G/A	*ZNF192 | LOC222699*	INTERGENIC	2	2.211	1.838	2.659	**3.43E-17**	0.074	0.044	9.60E-02	0.106	0.123	3.94E-01	0.189	0.044	**1.60E-05**	-0.016	0.123	8.96E-01	-0.136	0.044	**2.20E-03**	-0.060	0.074	4.16E-01
rs9461832	6	33164362	A/G	*HLA-DPB1 | HLA-DPB2*	INTERGENIC	2	2.699	2.143	3.400	**3.49E-17**	0.047	0.073	5.20E-01	0.017	0.123	8.89E-01	0.082	0.072	2.60E-01	-0.132	0.122	2.82E-01	-0.059	0.073	4.20E-01	-0.038	0.074	6.11E-01
rs4713139	6	28200664	A/G	*ZSCAN16*	INTRON	2	2.210	1.838	2.658	**3.55E-17**	0.070	0.044	1.14E-01	0.106	0.123	3.94E-01	0.184	0.044	**2.60E-05**	-0.016	0.123	8.96E-01	-0.133	0.044	**2.70E-03**	-0.060	0.074	4.16E-01
rs4713140	6	28205172	A/G	*ZSCAN16*	INTRON	2	2.210	1.838	2.657	**3.60E-17**	0.070	0.044	1.14E-01	0.106	0.123	3.94E-01	0.184	0.044	**2.60E-05**	-0.016	0.123	8.96E-01	-0.133	0.044	**2.70E-03**	-0.060	0.074	4.16E-01
rs1117490	6	30278489	G/A	*TRIM26*	INTRON	2	2.095	1.764	2.489	**3.80E-17**	0.028	0.038	4.60E-01	0.022	0.124	8.58E-01	0.130	0.038	**6.30E-04**	-0.047	0.124	7.06E-01	-0.106	0.038	**5.80E-03**	-0.055	0.075	4.63E-01
rs2523722	6	30273252	A/G	*TRIM26*	INTRON	2	2.102	1.768	2.499	**4.20E-17**	0.032	0.038	4.10E-01	0.022	0.124	8.58E-01	0.130	0.038	**5.90E-04**	-0.047	0.124	7.06E-01	-0.103	0.038	**7.20E-03**	-0.053	0.075	4.83E-01
rs885912	6	30282612	A/C	*TRIM26*	INTRON	2	2.094	1.762	2.489	**4.54E-17**	0.028	0.038	4.60E-01	0.022	0.124	8.58E-01	0.130	0.038	**6.30E-04**	-0.047	0.124	7.06E-01	-0.106	0.038	**5.80E-03**	-0.055	0.075	4.63E-01
rs3132571	6	31013292	G/A	*SFTPG | LOC100129065*	INTERGENIC	2	1.947	1.667	2.275	**4.63E-17**	0.033	0.033	3.30E-01	0.010	0.123	9.38E-01	0.061	0.033	6.20E-02	0.077	0.122	5.31E-01	-0.041	0.033	2.10E-01	-0.012	0.074	8.70E-01
rs2517611	6	30277306	G/A	*TRIM26*	INTRON	2	2.093	1.761	2.487	**4.70E-17**	0.028	0.038	4.70E-01	0.022	0.124	8.58E-01	0.129	0.038	**6.40E-04**	-0.047	0.124	7.06E-01	-0.106	0.038	**5.70E-03**	-0.053	0.075	4.83E-01
rs1265158	6	31248720	C/G	*POU5F1 | LOC100130889*	INTERGENIC	2	1.931	1.656	2.253	**5.08E-17**	-0.010	0.033	7.70E-01	-0.081	0.123	5.12E-01	0.051	0.033	1.19E-01	-0.016	0.122	8.99E-01	-0.057	0.033	8.30E-02	0.005	0.074	9.51E-01
rs1611133	6	29917361	A/G	*LOC100133214 | HLA-H*	INTERGENIC	2	2.033	1.722	2.400	**5.21E-17**	-0.022	0.037	5.50E-01	-0.039	0.123	7.52E-01	0.129	0.036	**4.10E-04**	-0.188	0.120	1.23E-01	-0.155	0.037	**2.60E-05**	0.004	0.074	9.56E-01
rs1265114	6	31225167	A/G	*CCHCR1*	INTRON	2	1.951	1.669	2.281	**5.27E-17**	-0.014	0.034	6.90E-01	-0.060	0.123	6.27E-01	0.101	0.034	**3.10E-03**	-0.190	0.120	1.18E-01	-0.117	0.034	**6.40E-04**	0.058	0.074	4.35E-01
rs1264709	6	30164455	T/A	*RNF39 | TRIM31*	INTERGENIC	2	2.134	1.787	2.548	**5.31E-17**	0.010	0.040	8.10E-01	0.001	0.123	9.95E-01	0.163	0.039	**3.40E-05**	-0.143	0.121	2.45E-01	-0.167	0.040	**2.60E-05**	-0.025	0.074	7.40E-01
rs1265087	6	31217789	A/G	*PSORS1C1 | CCHCR1*	INTERGENIC	2	1.957	1.673	2.290	**5.42E-17**	-0.014	0.034	6.90E-01	-0.060	0.123	6.27E-01	0.101	0.034	**3.10E-03**	-0.190	0.120	1.18E-01	-0.117	0.034	**6.40E-04**	0.058	0.074	4.35E-01
rs259926	6	30099846	A/G	*HCG9 | ZNRD1*	INTERGENIC	2	2.134	1.787	2.548	**5.44E-17**	-	-	-	0.001	0.123	9.95E-01	-	-	-	-0.143	0.121	2.45E-01	-	-	-	-0.025	0.074	7.40E-01
rs1265078	6	31220581	C/G	*CCHCR1*	INTRON	2	1.957	1.673	2.291	**5.47E-17**	-0.014	0.034	6.90E-01	-0.060	0.123	6.27E-01	0.101	0.034	**3.10E-03**	-0.190	0.120	1.18E-01	-0.117	0.034	**6.40E-04**	0.058	0.074	4.35E-01
rs1265112	6	31225998	G/A	*CCHCR1*	INTRON	2	1.957	1.673	2.291	**5.47E-17**	-0.014	0.034	6.90E-01	-0.060	0.123	6.27E-01	0.101	0.034	**3.10E-03**	-0.190	0.120	1.18E-01	-0.117	0.034	**6.40E-04**	0.058	0.074	4.35E-01
rs11965763	6	33184660	G/A	*HLA-DPB1 | HLA-DPB2*	INTERGENIC	2	2.667	2.120	3.355	**5.51E-17**	0.039	0.072	5.90E-01	0.017	0.123	8.89E-01	0.087	0.071	2.20E-01	-0.132	0.122	2.82E-01	-0.058	0.072	4.20E-01	-0.038	0.074	6.11E-01
rs1576	6	31218370	G/C	*CCHCR1*	CODING	2	1.957	1.672	2.290	**5.51E-17**	-0.014	0.034	6.90E-01	-0.060	0.123	6.27E-01	0.101	0.034	**3.10E-03**	-0.190	0.120	1.18E-01	-0.117	0.034	**6.40E-04**	0.057	0.074	4.40E-01
rs6911848	6	33183237	A/G	*HLA-DPB1 | HLA-DPB2*	INTERGENIC	2	2.667	2.120	3.355	**5.51E-17**	0.039	0.072	5.90E-01	0.017	0.123	8.89E-01	0.087	0.071	2.20E-01	-0.132	0.122	2.82E-01	-0.058	0.072	4.20E-01	-0.038	0.074	6.11E-01
rs6936204	6	32325070	A/G	*NOTCH4 | C6orf10*	INTERGENIC	2	1.914	1.644	2.228	**5.71E-17**	-0.062	0.032	5.60E-02	-0.089	0.123	4.72E-01	0.187	0.032	**4.60E-09**	-0.228	0.119	5.95E-02	-0.259	0.032	**7.80E-16**	-0.044	0.074	5.56E-01
rs9380069	6	28311279	G/A	*ZNF193 | ZKSCAN4*	INTERGENIC	2	2.169	1.809	2.601	**6.37E-17**	0.040	0.045	3.70E-01	0.013	0.124	9.15E-01	0.187	0.044	**2.30E-05**	-0.113	0.123	3.61E-01	-0.160	0.045	**3.50E-04**	-0.043	0.074	5.67E-01
rs3129012	6	30096621	A/G	*HCG9 | ZNRD1*	INTERGENIC	2	2.122	1.778	2.531	**7.05E-17**	-0.003	0.037	9.30E-01	0.001	0.123	9.95E-01	0.144	0.037	**9.30E-05**	-0.143	0.121	2.45E-01	-0.155	0.037	**3.10E-05**	-0.025	0.074	7.40E-01
rs1800683	6	31648050	A/G	*NFKBIL1 | LTA*	INTERGENIC	2	1.908	1.639	2.221	**7.31E-17**	-	-	-	-0.178	0.121	1.47E-01	-	-	-	-0.205	0.120	9.23E-02	-	-	-	0.006	0.075	9.37E-01
rs2517614	6	30271934	A/G	*TRIM26*	INTRON	2	2.094	1.760	2.491	**7.91E-17**	0.032	0.038	4.00E-01	0.022	0.124	8.58E-01	0.130	0.038	**5.80E-04**	-0.047	0.124	7.06E-01	-0.103	0.038	**7.20E-03**	-0.053	0.075	4.83E-01
rs9366717	6	28299036	A/G	*LOC222699 | ZNF193*	INTERGENIC	2	2.203	1.830	2.653	**8.06E-17**	0.037	0.043	3.80E-01	0.013	0.124	9.15E-01	0.180	0.042	**2.10E-05**	-0.113	0.123	3.61E-01	-0.156	0.043	**2.50E-04**	-0.041	0.074	5.82E-01
rs9393908	6	28298809	A/T	*LOC222699 | ZNF193*	INTERGENIC	2	2.203	1.830	2.653	**8.06E-17**	0.037	0.043	3.80E-01	0.013	0.124	9.15E-01	0.178	0.042	**2.50E-05**	-0.113	0.123	3.61E-01	-0.155	0.043	**2.90E-04**	-0.041	0.074	5.82E-01
rs2517985	6	31226921	G/A	*CCHCR1*	INTRON	2	1.942	1.661	2.271	**8.38E-17**	-	-	-	-0.060	0.123	6.27E-01	-	-	-	-0.190	0.120	1.18E-01	-	-	-	0.062	0.074	3.98E-01
rs2844776	6	30279806	G/A	*TRIM26*	INTRON	2	2.086	1.754	2.480	**8.63E-17**	0.028	0.038	4.60E-01	0.022	0.124	8.58E-01	0.130	0.038	**6.30E-04**	-0.047	0.124	7.06E-01	-0.106	0.038	**5.80E-03**	-0.055	0.075	4.63E-01
rs5000634	6	32771542	G/A	*HLA-DQB1 | HLA-DQA2*	INTERGENIC	2	0.477	0.401	0.568	**9.03E-17**	0.123	0.033	**1.60E-04**	0.010	0.123	9.33E-01	0.003	0.032	9.20E-01	0.101	0.122	4.10E-01	0.104	0.032	**1.43E-03**	-0.037	0.074	6.13E-01
rs1264714	6	30150188	A/C	*RNF39*	INTRON	2	2.126	1.780	2.541	**9.65E-17**	-	-	-	0.001	0.123	9.95E-01	-	-	-	-0.143	0.121	2.45E-01	-	-	-	-0.029	0.075	7.03E-01
rs11244	6	32888701	A/G	*HLA-DOB*	UTR	2	1.921	1.647	2.241	**9.69E-17**	0.055	0.036	1.31E-01	-0.089	0.128	4.88E-01	0.064	0.036	7.60E-02	-0.245	0.124	5.20E-02	-0.024	0.036	5.10E-01	0.004	0.075	9.55E-01
rs971570	6	30280492	C/A	*TRIM26*	UTR	2	2.076	1.747	2.467	**1.02E-16**	0.028	0.038	4.60E-01	0.022	0.124	8.58E-01	0.130	0.038	**6.30E-04**	-0.047	0.124	7.06E-01	-0.106	0.038	**5.80E-03**	-0.055	0.075	4.63E-01
rs9267444	6	31591437	A/G	*MICB | MCCD1*	INTERGENIC	2	1.904	1.635	2.216	**1.04E-16**	0.002	0.033	9.50E-01	-0.105	0.123	3.96E-01	0.118	0.033	**3.20E-04**	-0.187	0.120	1.25E-01	-0.118	0.033	**3.20E-04**	-0.043	0.074	5.64E-01
rs2072633	6	32027557	A/G	*CFB*	INTRON	2	1.927	1.650	2.250	**1.14E-16**	-0.031	0.031	3.20E-01	0.014	0.124	9.12E-01	0.131	0.031	**2.40E-05**	-0.116	0.122	3.47E-01	-0.168	0.031	**8.60E-08**	0.054	0.075	4.71E-01
rs2523716	6	30278504	A/G	*TRIM26*	INTRON	2	2.085	1.753	2.481	**1.15E-16**	0.028	0.038	4.60E-01	0.022	0.124	8.58E-01	0.130	0.038	**6.30E-04**	-0.047	0.124	7.06E-01	-0.106	0.038	**5.80E-03**	-0.055	0.075	4.63E-01
rs2243868	6	31369255	A/G	*HLA-C | HLA-B*	INTERGENIC	2	1.919	1.645	2.239	**1.20E-16**	-0.057	0.032	7.50E-02	0.029	0.124	8.15E-01	0.162	0.031	**2.40E-07**	-0.158	0.122	2.00E-01	-0.228	0.032	**6.30E-13**	0.141	0.074	5.79E-02
rs2524040	6	31365604	A/G	*HLA-C | HLA-B*	INTERGENIC	2	1.919	1.645	2.239	**1.20E-16**	-	-	-	0.029	0.124	8.15E-01	-	-	-	-0.158	0.122	2.00E-01	-	-	-	0.141	0.074	5.79E-02

rs2524156	6	31368376	A/G	*HLA-C | HLA-B*	INTERGENIC	2	1.919	1.645	2.239	**1.20E-16**	-0.057	0.032	7.50E-02	0.029	0.124	8.15E-01	0.162	0.031	**2.40E-07**	-0.158	0.122	2.00E-01	-0.228	0.032	**6.30E-13**	0.141	0.074	5.79E-02
rs2524163	6	31367558	G/A	*HLA-C | HLA-B*	INTERGENIC	2	1.919	1.645	2.239	**1.20E-16**	-0.057	0.032	7.50E-02	0.029	0.124	8.15E-01	0.162	0.031	**2.40E-07**	-0.158	0.122	2.00E-01	-0.228	0.032	**6.30E-13**	0.141	0.074	5.79E-02
rs2853933	6	31362067	A/G	*HLA-C | HLA-B*	INTERGENIC	2	1.919	1.645	2.239	**1.20E-16**	-0.057	0.032	7.50E-02	0.029	0.124	8.15E-01	0.162	0.031	**2.40E-07**	-0.158	0.122	2.00E-01	-0.228	0.032	**6.30E-13**	0.141	0.074	5.79E-02
rs9276815	6	32933485	A/G	*PSMB9*	INTRON	2	1.908	1.637	2.223	**1.21E-16**	0.097	0.034	**4.90E-03**	0.049	0.123	6.94E-01	0.030	0.034	3.70E-01	-0.096	0.122	4.36E-01	0.052	0.034	1.27E-01	0.016	0.074	8.32E-01
rs2517529	6	31184957	C/G	*HCG22 | C6orf15*	INTERGENIC	2	1.909	1.638	2.225	**1.22E-16**	-0.025	0.032	4.30E-01	-0.118	0.123	3.41E-01	0.029	0.032	3.70E-01	-0.017	0.123	8.93E-01	-0.045	0.032	1.60E-01	-0.090	0.074	2.22E-01
rs11796	6	31609191	T/A	*BAT1*	INTRON	2	1.881	1.619	2.184	**1.32E-16**	0.079	0.033	**1.80E-02**	-0.178	0.121	1.47E-01	0.026	0.033	4.40E-01	-0.205	0.120	9.23E-02	0.046	0.033	1.70E-01	0.002	0.075	9.75E-01
rs2523713	6	30281309	A/G	*TRIM26*	INTRON	2	2.076	1.746	2.468	**1.34E-16**	0.028	0.038	4.60E-01	0.022	0.124	8.58E-01	0.130	0.038	**6.30E-04**	-0.047	0.124	7.06E-01	-0.106	0.038	**5.80E-03**	-0.055	0.075	4.63E-01
rs2517610	6	30278259	G/A	*TRIM26*	INTRON	2	2.063	1.737	2.449	**1.41E-16**	-	-	-	0.022	0.124	8.58E-01	-	-	-	-0.047	0.124	7.06E-01	-	-	-	-0.055	0.075	4.63E-01
rs9267947	6	32319196	G/A	*NOTCH4 | C6orf10*	INTERGENIC	2	1.909	1.638	2.225	**1.41E-16**	0.045	0.033	1.70E-01	-0.030	0.123	8.09E-01	-0.022	0.032	5.00E-01	-0.036	0.123	7.71E-01	0.064	0.032	**4.80E-02**	-0.050	0.074	4.98E-01
rs11965538	6	28347894	A/G	*ZNF187 / ZNF187*	MISSENCE	2	2.166	1.803	2.603	**1.48E-16**	0.055	0.042	1.90E-01	0.013	0.124	9.15E-01	0.137	0.041	**9.20E-04**	-0.113	0.123	3.61E-01	-0.090	0.042	**3.10E-02**	-0.038	0.074	6.10E-01
rs2853922	6	31374169	A/G	*HLA-C | HLA-B*	INTERGENIC	2	1.927	1.649	2.252	**1.50E-16**	-	-	-	0.029	0.124	8.15E-01	-	-	-	-0.158	0.122	2.00E-01	-	-	-	0.141	0.074	5.79E-02
rs2524089	6	31374501	C/A	*HLA-C | HLA-B*	INTERGENIC	2	1.927	1.649	2.252	**1.51E-16**	-0.056	0.032	8.00E-02	0.029	0.124	8.15E-01	0.162	0.031	**2.80E-07**	-0.158	0.122	2.00E-01	-0.226	0.032	**8.50E-13**	0.141	0.074	5.79E-02
rs6901575	6	28358963	A/G	*PGBD1*	INTRON	2	2.157	1.797	2.589	**1.51E-16**	0.013	0.050	7.90E-01	0.013	0.124	9.15E-01	0.205	0.049	**3.20E-05**	-0.113	0.123	3.61E-01	-0.203	0.050	**4.60E-05**	-0.041	0.074	5.82E-01
rs909253	6	31648292	G/A	*LTA*	INTRON	2	1.900	1.631	2.213	**1.57E-16**	0.069	0.033	**3.70E-02**	-0.178	0.121	1.47E-01	0.022	0.033	4.90E-01	-0.205	0.120	9.23E-02	0.039	0.033	2.40E-01	0.007	0.075	9.31E-01
rs1041981	6	31648763	A/C	*LTA*	CODING	2	1.900	1.631	2.213	**1.62E-16**	0.069	0.033	**3.70E-02**	-0.178	0.121	1.47E-01	0.022	0.033	4.90E-01	-0.205	0.120	9.23E-02	0.039	0.033	2.40E-01	0.007	0.075	9.31E-01
rs2523721	6	30274245	A/G	*TRIM26*	CODING	2	2.071	1.742	2.463	**1.63E-16**	0.028	0.038	4.70E-01	0.022	0.124	8.58E-01	0.129	0.038	**6.40E-04**	-0.047	0.124	7.06E-01	-0.106	0.038	**5.70E-03**	-0.055	0.075	4.63E-01
rs3130553	6	31190264	G/A	*C6orf15 | PSORS1C1*	INTERGENIC	2	1.907	1.636	2.224	**1.67E-16**	-0.036	0.032	2.70E-01	-0.113	0.122	3.61E-01	0.049	0.032	1.26E-01	0.108	0.122	3.76E-01	-0.076	0.032	**1.70E-02**	-0.101	0.074	1.74E-01
rs2517549	6	31116577	A/C	*LOC729792*	INTRON	2	1.888	1.623	2.196	**1.79E-16**	0.003	0.033	9.30E-01	-0.092	0.123	4.58E-01	0.051	0.033	1.21E-01	-0.090	0.123	4.66E-01	-0.050	0.033	1.31E-01	-0.025	0.074	7.42E-01
rs2523901	6	31117116	G/A	*LOC729792*	INTRON	2	1.888	1.623	2.196	**1.79E-16**	-	-	-	-0.092	0.123	4.58E-01	-	-	-	-0.090	0.123	4.66E-01	-	-	-	-0.025	0.074	7.42E-01
rs2508015	6	31118179	A/G	*LOC729792*	INTRON	2	1.902	1.632	2.218	**2.27E-16**	-0.008	0.033	8.10E-01	-0.142	0.123	2.51E-01	0.036	0.032	2.70E-01	-0.076	0.123	5.41E-01	-0.039	0.033	2.30E-01	-0.050	0.074	5.03E-01
rs3130059	6	31617263	G/C	*SNORD84 | BAT1*	INTERGENIC	2	1.885	1.620	2.193	**2.31E-16**	0.075	0.033	**2.30E-02**	-0.178	0.121	1.47E-01	0.024	0.033	4.60E-01	-0.205	0.120	9.23E-02	0.044	0.033	1.90E-01	0.002	0.075	9.75E-01
rs3132657	6	30341492	G/A	*FLJ45422*	UTR	1	2.310	1.891	2.822	**2.37E-16**	0.021	0.042	6.20E-01	-0.045	0.124	7.15E-01	0.203	0.041	**8.00E-07**	-0.165	0.122	1.80E-01	-0.186	0.042	**7.80E-06**	-0.073	0.075	3.28E-01
rs2071594	6	31620699	C/G	*ATP6V1G2*	UTR	2	1.884	1.620	2.192	**2.39E-16**	0.075	0.033	**2.30E-02**	-0.178	0.121	1.47E-01	0.024	0.033	4.60E-01	-0.205	0.120	9.23E-02	0.044	0.033	1.90E-01	0.002	0.075	9.75E-01
rs9276900	6	32956544	C/A	*PSMB9 | HLA-DMB*	INTERGENIC	2	1.896	1.627	2.209	**2.40E-16**	0.089	0.034	**8.10E-03**	0.046	0.123	7.12E-01	0.018	0.033	5.80E-01	-0.129	0.122	2.94E-01	0.057	0.033	8.90E-02	0.012	0.074	8.75E-01
rs3129791	6	29062272	A/G	*C6orf100 | ZNF311*	INTERGENIC	1	2.540	2.033	3.174	**2.43E-16**	0.039	0.050	4.40E-01	0.037	0.124	7.66E-01	0.261	0.050	**1.50E-07**	-0.118	0.122	3.38E-01	-0.242	0.050	**1.40E-06**	-0.016	0.074	8.32E-01
rs2442744	6	31453355	A/G	*LOC729816 | LOC100129668*	INTERGENIC	2	1.871	1.610	2.173	**2.61E-16**	0.053	0.034	1.15E-01	0.055	0.124	6.55E-01	0.058	0.033	8.00E-02	-0.113	0.122	3.60E-01	-0.013	0.033	6.90E-01	0.021	0.075	7.76E-01
rs2507984	6	31453575	A/G	*LOC729816 | LOC100129668*	INTERGENIC	2	1.871	1.610	2.173	**2.61E-16**	0.053	0.034	1.15E-01	0.055	0.124	6.55E-01	0.058	0.033	8.00E-02	-0.113	0.122	3.60E-01	-0.013	0.033	6.90E-01	0.021	0.075	7.76E-01
rs2844546	6	31452636	A/G	*LOC729816 | LOC100129668*	INTERGENIC	2	1.871	1.610	2.173	**2.61E-16**	0.053	0.034	1.15E-01	0.055	0.124	6.55E-01	0.058	0.033	8.00E-02	-0.113	0.122	3.60E-01	-0.013	0.033	6.90E-01	0.021	0.075	7.76E-01
rs10456362	6	28329795	A/G	*ZKSCAN4 | NKAPL*	INTERGENIC	2	2.153	1.792	2.587	**2.63E-16**	0.046	0.044	2.90E-01	0.013	0.124	9.15E-01	0.143	0.043	**9.40E-04**	-0.113	0.123	3.61E-01	-0.111	0.044	**1.11E-02**	-0.041	0.074	5.82E-01
rs2799077	6	28342576	A/G	*NKAPL | ZNF187*	INTERGENIC	2	2.151	1.790	2.584	**2.83E-16**	0.046	0.044	2.90E-01	0.013	0.124	9.15E-01	0.141	0.043	**1.09E-03**	-0.113	0.123	3.61E-01	-0.108	0.044	**1.29E-02**	-0.041	0.074	5.82E-01
rs1419183	6	28350773	C/A	*ZNF187*	INTRON	2	2.151	1.790	2.584	**2.91E-16**	0.046	0.044	2.90E-01	0.013	0.124	9.15E-01	0.141	0.043	**1.09E-03**	-0.113	0.123	3.61E-01	-0.108	0.044	**1.29E-02**	-0.041	0.074	5.82E-01
rs1778508	6	28337860	G/A	*NKAPL | ZNF187*	INTERGENIC	2	2.150	1.790	2.584	**2.91E-16**	0.046	0.044	2.90E-01	0.013	0.124	9.15E-01	0.141	0.043	**1.09E-03**	-0.113	0.123	3.61E-01	-0.108	0.044	**1.29E-02**	-0.041	0.074	5.82E-01
rs3128982	6	31525170	G/A	*LOC100129668 | HCP5*	INTERGENIC	2	1.890	1.623	2.202	**2.93E-16**	0.007	0.033	8.30E-01	-0.027	0.123	8.30E-01	0.077	0.033	**2.00E-02**	-0.175	0.121	1.53E-01	-0.078	0.033	**1.90E-02**	0.009	0.074	9.06E-01
rs404890	6	32306845	A/C	*NOTCH4 | C6orf10*	INTERGENIC	2	1.888	1.621	2.200	**3.08E-16**	0.066	0.033	**4.50E-02**	-0.020	0.126	8.76E-01	-0.002	0.033	9.60E-01	0.183	0.123	1.42E-01	0.064	0.033	5.10E-02	0.076	0.075	3.08E-01
rs2071466	6	32913285	A/G	*TAP2*	INTRON	1	2.000	1.692	2.363	**4.06E-16**	-	-	-	0.086	0.123	4.86E-01	-	-	-	-0.090	0.122	4.62E-01	-	-	-	0.016	0.074	8.25E-01
rs7757767	6	32953851	G/A	*PSMB9 | HLA-DMB*	INTERGENIC	2	1.877	1.613	2.185	**4.42E-16**	0.089	0.034	**8.10E-03**	0.057	0.123	6.46E-01	0.018	0.033	5.80E-01	-0.137	0.121	2.64E-01	0.057	0.033	8.90E-02	0.010	0.074	8.92E-01
rs886391	6	30434507	A/T	*RPP21 | HLA-E*	INTERGENIC	2	1.929	1.646	2.260	**4.51E-16**	-	-	-	0.139	0.122	2.57E-01	-	-	-	-0.025	0.122	8.40E-01	-	-	-	0.048	0.074	5.21E-01
rs2508049	6	29931862	G/A	*LOC100133214 | HLA-H*	INTERGENIC	1	2.300	1.881	2.812	**4.52E-16**	0.018	0.040	6.50E-01	-0.002	0.124	9.84E-01	0.163	0.039	**3.50E-05**	-0.152	0.122	2.15E-01	-0.158	0.040	**7.30E-05**	-0.026	0.074	7.29E-01
rs3130630	6	31618912	C/A	*BAT1 | ATP6V1G2*	INTERGENIC	2	1.877	1.612	2.185	**5.00E-16**	-	-	-	-0.178	0.121	1.47E-01	-	-	-	-0.205	0.120	9.23E-02	-	-	-	0.005	0.075	9.51E-01
rs1264372	6	30877705	A/G	*IER3 | DDR1*	INTERGENIC	2	1.888	1.619	2.202	**5.58E-16**	-	-	-	-0.110	0.123	3.76E-01	-	-	-	-0.230	0.120	5.97E-02	-	-	-	0.026	0.075	7.23E-01
rs6929408	6	31408628	A/G	*HLA-C | HLA-B*	INTERGENIC	2	1.892	1.621	2.208	**5.79E-16**	0.028	0.033	4.00E-01	-0.067	0.123	5.87E-01	0.057	0.033	8.00E-02	-0.019	0.122	8.78E-01	-0.025	0.033	4.40E-01	-0.062	0.074	4.05E-01
rs9267911	6	32313088	G/A	*NOTCH4 | C6orf10*	INTERGENIC	2	1.885	1.617	2.199	**5.91E-16**	0.078	0.032	**1.60E-02**	0.047	0.123	7.06E-01	-0.030	0.032	3.50E-01	0.185	0.120	1.30E-01	0.106	0.032	**1.09E-03**	0.055	0.074	4.59E-01
rs3763327	6	32521808	G/C	*HLA-DRA | HLA-DRB5*	INTERGENIC	1	2.030	1.710	2.410	**6.03E-16**	-0.096	0.032	**3.10E-03**	-0.055	0.123	6.54E-01	0.129	0.032	**5.90E-05**	-0.228	0.119	6.00E-02	-0.221	0.032	**7.70E-12**	-0.047	0.074	5.26E-01
rs2524005	6	30007656	A/G	*HCG4P6 | HLA-A*	INTERGENIC	2	2.042	1.718	2.429	**6.52E-16**	0.014	0.038	7.10E-01	-0.079	0.123	5.19E-01	0.133	0.037	**3.70E-04**	-0.179	0.120	1.41E-01	-0.131	0.038	**5.50E-04**	-0.026	0.074	7.29E-01
rs4947350	6	32875598	G/A	*HLA-DQB2 | HLA-DOB*	INTERGENIC	2	1.949	1.658	2.292	**6.71E-16**	0.031	0.039	4.30E-01	0.022	0.125	8.62E-01	0.090	0.039	**2.10E-02**	-0.105	0.124	4.01E-01	-0.070	0.039	7.50E-02	0.060	0.075	4.21E-01
rs2853935	6	31361857	G/A	*HLA-C | HLA-B*	INTERGENIC	2	1.909	1.632	2.234	**6.96E-16**	-0.057	0.032	7.50E-02	0.029	0.124	8.15E-01	0.162	0.031	**2.40E-07**	-0.158	0.122	2.00E-01	-0.228	0.032	**6.30E-13**	0.141	0.074	5.79E-02
rs2516412	6	31570300	A/C	*3.8-1 | MICB*	INTERGENIC	2	1.873	1.608	2.182	**7.35E-16**	-	-	-	-0.046	0.123	7.08E-01	-	-	-	0.001	0.123	9.97E-01	-	-	-	-0.068	0.074	3.62E-01
rs2849015	6	32306914	A/G	*NOTCH4 | C6orf10*	INTERGENIC	2	1.890	1.619	2.206	**7.48E-16**	0.066	0.033	**4.50E-02**	-0.020	0.126	8.76E-01	-0.002	0.033	9.60E-01	0.183	0.123	1.42E-01	0.064	0.033	5.10E-02	0.076	0.075	3.08E-01
rs805301	6	31726100	C/A	*BAT3*	INTRON	2	1.858	1.598	2.160	**7.57E-16**	0.046	0.034	1.80E-01	-0.161	0.123	1.95E-01	0.049	0.034	1.42E-01	-0.054	0.123	6.60E-01	-0.008	0.034	8.10E-01	-0.088	0.074	2.35E-01
rs805302	6	31725936	C/A	*BAT3*	INTRON	2	1.857	1.598	2.159	**7.61E-16**	-	-	-	-0.161	0.123	1.95E-01	-	-	-	-0.054	0.123	6.60E-01	-	-	-	-0.088	0.074	2.35E-01
rs2021722	6	30282110	A/G	*TRIM26*	INTRON	2	2.034	1.711	2.419	**8.32E-16**	0.028	0.038	4.60E-01	0.022	0.124	8.58E-01	0.130	0.038	**6.30E-04**	-0.047	0.124	7.06E-01	-0.106	0.038	**5.80E-03**	-0.055	0.075	4.63E-01
rs3130402	6	30339566	G/A	*FLJ45422*	UTR	1	2.290	1.871	2.802	**8.57E-16**	-	-	-	-0.045	0.124	7.15E-01	-	-	-	-0.165	0.122	1.80E-01	-	-	-	-0.069	0.075	3.56E-01
rs1632854	6	31083628	T/A	*C6orf205 | LOC729792*	INTERGENIC	2	1.867	1.603	2.174	**8.60E-16**	0.036	0.032	2.70E-01	-0.116	0.122	3.47E-01	0.029	0.032	3.60E-01	0.129	0.122	2.91E-01	0.002	0.032	9.40E-01	-0.118	0.073	1.10E-01
rs6937871	6	31406799	C/A	*HLA-C | HLA-B*	INTERGENIC	2	1.884	1.615	2.199	**8.84E-16**	-	-	-	-0.067	0.123	5.87E-01	-	-	-	-0.019	0.122	8.78E-01	-	-	-	-0.062	0.074	4.05E-01
rs6931062	6	32943593	A/C	*PSMB9 | HLA-DMB*	INTERGENIC	2	1.878	1.610	2.189	**8.87E-16**	0.089	0.034	**8.10E-03**	0.057	0.123	6.46E-01	0.018	0.033	5.80E-01	-0.137	0.121	2.64E-01	0.057	0.033	8.90E-02	0.010	0.074	8.92E-01
rs3093974	6	31614189	A/G	*BAT1*	INTRON	2	1.860	1.599	2.163	**8.92E-16**	-	-	-	-0.178	0.121	1.47E-01	-	-	-	-0.205	0.120	9.23E-02	-	-	-	0.007	0.075	9.22E-01
rs6929078	6	32946642	G/A	*PSMB9 | HLA-DMB*	INTERGENIC	2	1.877	1.610	2.189	**9.02E-16**	0.089	0.034	**8.10E-03**	0.057	0.123	6.46E-01	0.018	0.033	5.80E-01	-0.137	0.121	2.64E-01	0.057	0.033	8.90E-02	0.010	0.074	8.92E-01
rs1580345	6	32945674	C/A	*PSMB9 | HLA-DMB*	INTERGENIC	2	1.877	1.610	2.189	**9.05E-16**	-	-	-	0.057	0.123	6.46E-01	-	-	-	-0.137	0.121	2.64E-01	-	-	-	0.010	0.074	8.92E-01
rs4947259	6	32959485	A/G	*PSMB9 | HLA-DMB*	INTERGENIC	2	1.869	1.604	2.177	**9.43E-16**	0.087	0.034	**9.60E-03**	0.057	0.123	6.46E-01	0.017	0.033	6.00E-01	-0.137	0.121	2.64E-01	0.056	0.033	9.30E-02	0.010	0.074	8.92E-01
rs2170185	6	32957617	G/A	*PSMB9 | HLA-DMB*	INTERGENIC	2	1.869	1.604	2.177	**9.66E-16**	-	-	-	0.057	0.123	6.46E-01	-	-	-	-0.137	0.121	2.64E-01	-	-	-	0.010	0.074	8.92E-01
rs241438	6	32905598	A/G	*TAP2*	INTRON	2	1.857	1.595	2.161	**1.43E-15**	-0.007	0.034	8.30E-01	-0.018	0.123	8.86E-01	-0.021	0.033	5.30E-01	-0.182	0.120	1.36E-01	0.019	0.033	5.80E-01	0.080	0.074	2.80E-01
rs2844499	6	31583892	A/T	*MICB*	INTRON	2	1.864	1.599	2.172	**1.47E-15**	0.049	0.035	1.60E-01	-0.046	0.124	7.12E-01	0.070	0.035	**4.20E-02**	0.001	0.124	9.94E-01	-0.026	0.035	4.50E-01	-0.068	0.074	3.60E-01
rs2071592	6	31623319	A/T	*ATP6V1G2 | NFKBIL1*	INTERGENIC	2	1.869	1.603	2.180	**1.51E-15**	0.057	0.033	8.50E-02	-0.118	0.122	3.38E-01	0.056	0.033	8.60E-02	-0.161	0.121	1.88E-01	-0.006	0.033	8.60E-01	-0.001	0.075	9.93E-01
rs2516400	6	31589084	A/G	*MICB | MCCD1*	INTERGENIC	2	1.862	1.598	2.170	**1.56E-15**	0.041	0.035	2.40E-01	-0.046	0.123	7.08E-01	0.082	0.035	**1.90E-02**	0.001	0.123	9.97E-01	-0.045	0.035	2.00E-01	-0.068	0.074	3.62E-01
rs805303	6	31724345	A/G	*BAT3*	INTRON	2	1.857	1.595	2.162	**1.59E-15**	0.046	0.034	1.80E-01	-0.161	0.123	1.95E-01	0.049	0.034	1.42E-01	-0.054	0.123	6.60E-01	-0.008	0.034	8.10E-01	-0.087	0.074	2.43E-01
rs2517862	6	29929916	A/G	*LOC100133214 | HLA-H*	INTERGENIC	1	2.280	1.862	2.792	**1.61E-15**	0.018	0.040	6.50E-01	-0.002	0.124	9.84E-01	0.163	0.039	**3.50E-05**	-0.152	0.122	2.15E-01	-0.158	0.040	**7.30E-05**	-0.026	0.074	7.29E-01
rs2523860	6	31153553	C/G	*HCG22 | C6orf15*	INTERGENIC	2	1.871	1.604	2.182	**1.63E-15**	0.029	0.032	3.80E-01	-0.139	0.122	2.59E-01	-0.045	0.032	1.60E-01	0.061	0.122	6.18E-01	0.067	0.032	**3.80E-02**	-0.122	0.073	9.85E-02
rs1634717	6	31080568	A/C	*C6orf205 | LOC729792*	INTERGENIC	2	1.859	1.596	2.166	**1.77E-15**	0.036	0.032	2.70E-01	-0.116	0.122	3.47E-01	0.029	0.032	3.60E-01	0.129	0.122	2.91E-01	0.002	0.032	9.40E-01	-0.118	0.073	1.10E-01
rs6928350	6	33191175	G/C	*HLA-DPB2*	INTRON	2	2.566	2.035	3.237	**1.77E-15**	0.056	0.076	4.60E-01	0.026	0.123	8.35E-01	0.134	0.075	7.40E-02	-0.118	0.122	3.34E-01	-0.096	0.076	2.10E-01	-0.041	0.074	5.80E-01

rs3129683	6	29394649	G/C	*OR5U1 | OR5V1*	INTERGENIC	2	2.073	1.732	2.481	**1.92E-15**	-0.026	0.042	5.30E-01	0.101	0.123	4.14E-01	0.147	0.041	**3.80E-04**	-0.045	0.122	7.13E-01	-0.188	0.042	**6.70E-06**	-0.006	0.074	9.32E-01
rs9272358	6	32712516	A/G	*HLA-DRB1 | HLA-DQA1*	INTERGENIC	1	0.440	0.359	0.539	**2.07E-15**	-	-	-	0.010	0.124	9.35E-01	-	-	-	0.120	0.123	3.31E-01	-	-	-	-0.019	0.074	8.02E-01
rs753725	6	30998850	A/G	*VARS2*	INTRON	2	1.881	1.609	2.199	**2.14E-15**	-0.009	0.032	7.70E-01	0.009	0.123	9.41E-01	0.128	0.032	**4.90E-05**	0.061	0.122	6.19E-01	-0.153	0.032	**1.60E-06**	0.024	0.074	7.47E-01
rs2517467	6	30997239	G/A	*VARS2*	INTRON	2	1.881	1.609	2.198	**2.15E-15**	-	-	-	0.009	0.123	9.41E-01	-	-	-	0.061	0.122	6.19E-01	-	-	-	0.024	0.074	7.47E-01
rs1046089	6	31710946	A/G	*BAT2*	CODING	2	1.847	1.586	2.150	**2.48E-15**	0.061	0.034	7.40E-02	-0.172	0.123	1.66E-01	0.063	0.034	6.40E-02	-0.037	0.124	7.66E-01	-0.004	0.034	9.00E-01	-0.078	0.074	2.95E-01
rs434841	6	32299019	A/G	*NOTCH4*	INTRON	2	1.847	1.587	2.151	**2.73E-15**	0.120	0.036	**9.10E-04**	-0.090	0.123	4.65E-01	0.022	0.036	5.40E-01	-0.210	0.120	8.45E-02	0.078	0.036	**3.00E-02**	-0.104	0.074	1.62E-01
rs2855812	6	31580699	A/C	*MICB*	INTRON	1	2.010	1.690	2.390	**2.87E-15**	0.032	0.037	3.90E-01	0.006	0.124	9.62E-01	0.143	0.037	**1.20E-04**	0.068	0.123	5.81E-01	-0.117	0.037	**1.80E-03**	-0.082	0.074	2.71E-01
rs2535294	6	31170112	G/A	*HCG22 | C6orf15*	INTERGENIC	2	1.873	1.603	2.188	**2.89E-15**	0.014	0.032	6.70E-01	-0.170	0.121	1.67E-01	-0.033	0.032	3.00E-01	0.056	0.122	6.47E-01	0.039	0.032	2.30E-01	-0.122	0.073	9.82E-02
rs813115	6	31727999	G/A	*BAT3*	INTRON	2	1.849	1.587	2.154	**3.34E-15**	-	-	-	-0.161	0.123	1.95E-01	-	-	-	-0.054	0.123	6.60E-01	-	-	-	-0.088	0.074	2.35E-01
rs2249464	6	30996140	A/G	*VARS2*	CODING	2	1.873	1.602	2.189	**3.36E-15**	-0.009	0.032	7.70E-01	0.009	0.123	9.41E-01	0.128	0.032	**4.90E-05**	0.061	0.122	6.19E-01	-0.153	0.032	**1.60E-06**	0.024	0.074	7.47E-01
rs9268969	6	32542327	A/G	*HLA-DRA | HLA-DRB5*	INTERGENIC	2	0.450	0.369	0.549	**3.91E-15**	0.094	0.032	**3.60E-03**	0.041	0.124	7.45E-01	-0.028	0.032	3.70E-01	0.121	0.123	3.26E-01	0.113	0.032	**4.00E-04**	-0.005	0.074	9.50E-01
rs2532934	6	31002738	G/A	*VARS2 | SFTPG*	INTERGENIC	2	1.862	1.594	2.175	**4.03E-15**	-0.009	0.032	7.70E-01	0.009	0.123	9.41E-01	0.128	0.032	**4.90E-05**	0.061	0.122	6.19E-01	-0.153	0.032	**1.60E-06**	0.018	0.074	8.12E-01
rs3131621	6	31533478	G/A	*LOC100129668 | HCP5*	INTERGENIC	2	1.842	1.581	2.146	**4.47E-15**	0.001	0.032	9.80E-01	0.050	0.124	6.86E-01	0.073	0.032	**2.40E-02**	-0.178	0.121	1.47E-01	-0.080	0.032	**1.29E-02**	0.031	0.074	6.82E-01
rs2535319	6	30822458	G/A	*IER3 | DDR1*	INTERGENIC	2	1.833	1.575	2.133	**4.62E-15**	0.018	0.032	5.90E-01	-0.010	0.123	9.36E-01	0.040	0.032	2.00E-01	-0.024	0.122	8.48E-01	-0.022	0.032	4.80E-01	0.025	0.074	7.37E-01
rs2516408	6	31571470	A/G	*3.8-1 | MICB*	INTERGENIC	2	1.855	1.589	2.165	**4.85E-15**	-	-	-	-0.046	0.123	7.08E-01	-	-	-	0.001	0.123	9.97E-01	-	-	-	-0.068	0.074	3.62E-01
rs7381897	6	31181882	G/A	*HCG22 | C6orf15*	INTERGENIC	2	1.855	1.589	2.165	**5.12E-15**	0.028	0.032	3.90E-01	-0.139	0.122	2.59E-01	-0.042	0.032	1.90E-01	0.061	0.122	6.18E-01	0.060	0.032	6.30E-02	-0.121	0.073	9.95E-02
rs1064191	6	31183354	G/A	*HCG22 | C6orf15*	INTERGENIC	2	1.854	1.588	2.165	**5.27E-15**	0.028	0.032	3.90E-01	-0.139	0.122	2.59E-01	-0.042	0.032	1.90E-01	0.061	0.122	6.18E-01	0.060	0.032	6.30E-02	-0.121	0.073	9.95E-02
rs200991	6	27923473	A/C	*HIST1H2BN | HIST1H2AL*	INTERGENIC	2	2.060	1.719	2.469	**5.38E-15**	0.028	0.043	5.10E-01	0.115	0.124	3.57E-01	0.166	0.042	**9.00E-05**	0.134	0.123	2.78E-01	-0.154	0.043	**3.20E-04**	-0.081	0.074	2.80E-01
rs2517455	6	31167879	G/A	*HCG22 | C6orf15*	INTERGENIC	2	1.854	1.588	2.164	**5.39E-15**	0.023	0.032	4.80E-01	-0.139	0.122	2.59E-01	-0.045	0.032	1.60E-01	0.061	0.122	6.18E-01	0.058	0.032	7.20E-02	-0.122	0.073	9.85E-02
rs916570	6	30174010	G/A	*RNF39 | TRIM31*	INTERGENIC	2	1.912	1.625	2.249	**5.51E-15**	0.009	0.035	8.00E-01	-0.167	0.121	1.75E-01	0.088	0.035	**1.11E-02**	-0.193	0.120	1.13E-01	-0.080	0.035	**2.20E-02**	-0.073	0.074	3.25E-01
rs2523589	6	31435313	A/C	*HLA-B | LOC729816*	INTERGENIC	2	0.525	0.447	0.617	**5.52E-15**	0.110	0.032	**5.30E-04**	-0.056	0.123	6.50E-01	-0.206	0.031	**5.60E-11**	0.147	0.121	2.28E-01	0.319	0.032	**5.20E-24**	-0.032	0.074	6.65E-01
rs9268853	6	32537621	G/A	*HLA-DRA | HLA-DRB5*	INTERGENIC	2	0.459	0.377	0.558	**5.61E-15**	0.096	0.034	**4.30E-03**	0.041	0.124	7.45E-01	-0.107	0.033	**1.20E-03**	0.121	0.123	3.26E-01	0.201	0.033	**1.90E-09**	-0.005	0.074	9.50E-01
rs2242660	6	31705732	A/G	*BAT2*	INTRON	2	1.825	1.569	2.122	**5.66E-15**	0.048	0.034	1.50E-01	-0.161	0.123	1.95E-01	0.054	0.034	1.06E-01	-0.054	0.123	6.60E-01	-0.008	0.034	8.20E-01	-0.088	0.074	2.35E-01
rs2395185	6	32541145	A/C	*HLA-DRA | HLA-DRB5*	INTERGENIC	2	0.459	0.377	0.558	**5.68E-15**	0.095	0.034	**4.60E-03**	0.041	0.124	7.45E-01	-0.112	0.033	**7.30E-04**	0.121	0.123	3.26E-01	0.205	0.033	**8.20E-10**	-0.005	0.074	9.50E-01
rs9268923	6	32540813	A/G	*HLA-DRA | HLA-DRB5*	INTERGENIC	2	0.459	0.377	0.558	**5.68E-15**	0.094	0.032	**3.60E-03**	0.041	0.124	7.45E-01	-0.028	0.032	3.70E-01	0.121	0.123	3.26E-01	0.113	0.032	**4.00E-04**	-0.005	0.074	9.50E-01
rs9405108	6	32546626	A/G	*HLA-DRA | HLA-DRB5*	INTERGENIC	2	0.459	0.377	0.558	**5.68E-15**	-	-	-	0.041	0.124	7.45E-01	-	-	-	0.121	0.123	3.26E-01	-	-	-	-0.005	0.074	9.50E-01
rs9368726	6	32546520	G/A	*HLA-DRA | HLA-DRB5*	INTERGENIC	2	0.459	0.378	0.558	**5.88E-15**	-	-	-	0.041	0.124	7.45E-01	-	-	-	0.121	0.123	3.26E-01	-	-	-	-0.005	0.074	9.50E-01
rs41365645	6	30312378	C/G	*TRIM26 | FLJ45422*	INTERGENIC	1	2.290	1.860	2.820	**5.97E-15**	-	-	-	-0.027	0.125	8.29E-01	-	-	-	-0.150	0.123	2.26E-01	-	-	-	-0.057	0.075	4.46E-01
rs2734985	6	29926641	G/A	*LOC100133214 | HLA-H*	INTERGENIC	2	1.948	1.648	2.304	**6.37E-15**	-0.002	0.036	9.50E-01	0.045	0.123	7.17E-01	0.120	0.035	**6.90E-04**	-0.145	0.121	2.34E-01	-0.125	0.036	**4.40E-04**	-0.039	0.074	5.99E-01
rs2844465	6	31709001	G/A	*BAT2*	INTRON	2	1.840	1.578	2.145	**7.11E-15**	-	-	-	-0.161	0.123	1.95E-01	-	-	-	-0.054	0.123	6.60E-01	-	-	-	-0.084	0.074	2.59E-01
rs2239804	6	32519501	G/A	*HLA-DRA*	INTRON	2	0.506	0.426	0.600	**7.46E-15**	0.075	0.032	**1.90E-02**	0.011	0.123	9.31E-01	-0.105	0.031	**8.50E-04**	0.196	0.120	1.07E-01	0.182	0.032	**8.80E-09**	0.050	0.074	5.01E-01
rs6911419	6	32517765	G/A	*HLA-DRA*	INTRON	2	0.506	0.426	0.600	**7.46E-15**	0.029	0.073	6.90E-01	0.011	0.123	9.31E-01	-0.225	0.072	**1.80E-03**	0.196	0.120	1.07E-01	0.271	0.072	**1.90E-04**	0.050	0.074	5.01E-01
rs6931646	6	32517759	A/G	*HLA-DRA*	INTRON	2	0.506	0.426	0.600	**7.46E-15**	-	-	-	0.011	0.123	9.31E-01	-	-	-	0.196	0.120	1.07E-01	-	-	-	0.050	0.074	5.01E-01
rs9268658	6	32518694	A/G	*HLA-DRA*	INTRON	2	0.506	0.426	0.600	**7.46E-15**	-	-	-	0.011	0.123	9.31E-01	-	-	-	0.196	0.120	1.07E-01	-	-	-	0.050	0.074	5.01E-01
rs154977	6	33007996	G/C	*PSMB9 | HLA-DMB*	INTERGENIC	2	1.875	1.600	2.197	**7.50E-15**	-	-	-	0.027	0.123	8.27E-01	-	-	-	0.053	0.122	6.67E-01	-	-	-	0.007	0.074	9.26E-01
rs1265063	6	31185917	G/A	*HCG22 | C6orf15*	INTERGENIC	2	1.847	1.582	2.156	**7.92E-15**	0.028	0.032	3.90E-01	-0.139	0.122	2.59E-01	-0.042	0.032	1.90E-01	0.061	0.122	6.18E-01	0.060	0.032	6.30E-02	-0.121	0.073	9.95E-02
rs1265064	6	31185711	C/G	*HCG22 | C6orf15*	INTERGENIC	2	1.847	1.582	2.156	**7.92E-15**	0.028	0.032	3.90E-01	-0.139	0.122	2.59E-01	-0.042	0.032	1.90E-01	0.061	0.122	6.18E-01	0.060	0.032	6.30E-02	-0.121	0.073	9.95E-02
rs2621426	6	32954548	A/G	*PSMB9 | HLA-DMB*	INTERGENIC	2	1.846	1.581	2.154	**7.95E-15**	0.089	0.034	**8.10E-03**	0.057	0.123	6.46E-01	0.018	0.033	5.80E-01	-0.137	0.121	2.64E-01	0.057	0.033	8.90E-02	0.008	0.075	9.16E-01
rs2517450	6	31169418	A/G	*HCG22 | C6orf15*	INTERGENIC	2	1.846	1.581	2.155	**8.19E-15**	0.028	0.032	3.90E-01	-0.139	0.122	2.59E-01	-0.042	0.032	1.90E-01	0.061	0.122	6.18E-01	0.060	0.032	6.30E-02	-0.122	0.073	9.85E-02
rs200956	6	27947725	G/A	*HIST1H3I*	CODING	2	2.033	1.699	2.431	**8.26E-15**	0.027	0.044	5.30E-01	0.117	0.123	3.47E-01	0.182	0.043	**2.40E-05**	0.142	0.122	2.49E-01	-0.168	0.044	**1.20E-04**	-0.084	0.074	2.60E-01
rs2517453	6	31168094	A/G	*HCG22 | C6orf15*	INTERGENIC	2	1.838	1.576	2.144	**8.52E-15**	0.028	0.032	3.90E-01	-0.139	0.122	2.59E-01	-0.042	0.032	1.90E-01	0.061	0.122	6.18E-01	0.060	0.032	6.30E-02	-0.122	0.073	9.85E-02
rs2517868	6	29927317	A/C	*LOC100133214 | HLA-H*	INTERGENIC	2	1.954	1.650	2.315	**8.79E-15**	0.006	0.041	8.90E-01	0.063	0.123	6.10E-01	0.180	0.040	**9.30E-06**	-0.143	0.121	2.44E-01	-0.196	0.041	**1.60E-06**	-0.025	0.074	7.34E-01
rs2734974	6	29941758	G/A	*LOC100133214 | HLA-H*	INTERGENIC	2	1.934	1.637	2.285	**9.06E-15**	0.044	0.038	2.40E-01	-0.009	0.123	9.40E-01	0.108	0.037	**3.60E-03**	-0.112	0.122	3.60E-01	-0.071	0.038	5.70E-02	0.049	0.074	5.14E-01
rs805257	6	31742172	G/A	*CSNK2B*	INTRON	2	1.835	1.573	2.139	**9.51E-15**	0.044	0.034	1.90E-01	-0.161	0.123	1.95E-01	0.049	0.034	1.43E-01	-0.054	0.123	6.60E-01	-0.009	0.034	7.80E-01	-0.088	0.074	2.35E-01
rs2734980	6	29929875	A/G	*LOC100133214 | HLA-H*	INTERGENIC	2	1.945	1.643	2.301	**9.72E-15**	-0.004	0.036	9.00E-01	0.042	0.123	7.35E-01	0.118	0.035	**8.60E-04**	-0.155	0.121	2.06E-01	-0.125	0.036	**4.60E-04**	-0.040	0.074	5.89E-01
rs2734984	6	29928810	G/A	*LOC100133214 | HLA-H*	INTERGENIC	2	1.945	1.643	2.301	**9.72E-15**	-0.004	0.036	9.00E-01	0.042	0.123	7.35E-01	0.118	0.035	**8.60E-04**	-0.155	0.121	2.06E-01	-0.125	0.036	**4.60E-04**	-0.040	0.074	5.89E-01
rs5013093	6	29928565	A/G	*LOC100133214 | HLA-H*	INTERGENIC	2	1.945	1.643	2.301	**9.72E-15**	-0.006	0.036	8.70E-01	0.042	0.123	7.35E-01	0.120	0.035	**6.90E-04**	-0.155	0.121	2.06E-01	-0.129	0.036	**3.10E-04**	-0.040	0.074	5.89E-01
rs1986997	6	31336389	A/G	*HCG27 | HLA-C*	INTERGENIC	1	1.970	1.659	2.339	**9.83E-15**	-0.033	0.032	3.00E-01	0.053	0.124	6.70E-01	0.204	0.032	**1.20E-10**	-0.152	0.122	2.18E-01	-0.250	0.032	**4.50E-15**	0.137	0.074	6.44E-02
rs2517861	6	29929961	A/G	*LOC100133214 | HLA-H*	INTERGENIC	2	1.944	1.643	2.301	**9.86E-15**	-0.004	0.036	9.00E-01	0.042	0.123	7.35E-01	0.118	0.035	**8.60E-04**	-0.155	0.121	2.06E-01	-0.125	0.036	**4.60E-04**	-0.040	0.074	5.89E-01
rs2844651	6	31008643	A/G	*SFTPG | LOC100129065*	INTERGENIC	2	1.852	1.584	2.165	**1.01E-14**	-0.012	0.032	7.00E-01	0.009	0.123	9.41E-01	0.127	0.032	**5.90E-05**	0.061	0.122	6.19E-01	-0.154	0.032	**1.20E-06**	0.032	0.075	6.75E-01
rs2535290	6	31171111	G/A	*HCG22 | C6orf15*	INTERGENIC	2	1.854	1.585	2.168	**1.04E-14**	0.028	0.032	3.90E-01	-0.139	0.122	2.59E-01	-0.042	0.032	1.90E-01	0.061	0.122	6.18E-01	0.060	0.032	6.30E-02	-0.121	0.073	9.95E-02
rs1064190	6	31183094	A/C	*HCG22 | C6orf15*	INTERGENIC	2	1.846	1.580	2.156	**1.08E-14**	0.028	0.032	3.90E-01	-0.139	0.122	2.59E-01	-0.042	0.032	1.90E-01	0.061	0.122	6.18E-01	0.060	0.032	6.30E-02	-0.123	0.074	9.57E-02
rs2517592	6	30201116	A/G	*TRIM31 | TRIM40*	INTERGENIC	2	1.867	1.592	2.189	**1.48E-14**	0.010	0.037	7.80E-01	-0.054	0.123	6.60E-01	0.080	0.036	**2.90E-02**	-0.162	0.121	1.84E-01	-0.067	0.037	6.80E-02	-0.006	0.074	9.37E-01
rs2517600	6	30182142	A/G	*TRIM31*	INTRON	2	1.852	1.583	2.167	**1.50E-14**	-	-	-	-0.076	0.123	5.37E-01	-	-	-	-0.058	0.122	6.35E-01	-	-	-	-0.091	0.074	2.19E-01
rs2523700	6	31561008	C/G	*3.8-1 | MICB*	INTERGENIC	2	1.828	1.566	2.133	**1.87E-14**	0.048	0.035	1.70E-01	-0.019	0.123	8.77E-01	0.117	0.035	**7.10E-04**	0.046	0.122	7.10E-01	-0.076	0.035	**3.00E-02**	-0.089	0.074	2.28E-01
rs2523708	6	31559258	A/G	*3.8-1 | MICB*	INTERGENIC	2	1.828	1.566	2.133	**1.87E-14**	0.048	0.035	1.70E-01	-0.019	0.123	8.77E-01	0.117	0.035	**7.10E-04**	0.046	0.122	7.10E-01	-0.076	0.035	**3.00E-02**	-0.089	0.074	2.28E-01
rs2269426	6	32184477	A/G	*TNXB*	INTRON	2	0.510	0.429	0.606	**1.96E-14**	0.090	0.032	**5.50E-03**	-0.098	0.123	4.27E-01	-0.103	0.032	**1.31E-03**	0.199	0.120	1.03E-01	0.195	0.032	**1.30E-09**	-0.013	0.074	8.65E-01
rs589428	6	31956199	A/C	*LOC100128067*	UTR	2	1.812	1.556	2.110	**2.06E-14**	-0.023	0.033	4.90E-01	-0.088	0.124	4.81E-01	0.223	0.032	**6.40E-12**	-0.103	0.123	4.08E-01	-0.255	0.033	**5.20E-15**	-0.041	0.075	5.87E-01
rs415929	6	32297010	G/A	*NOTCH4*	CODING	2	0.494	0.412	0.592	**2.09E-14**	0.065	0.035	6.10E-02	-0.055	0.123	6.58E-01	-0.089	0.034	**9.40E-03**	0.217	0.119	7.30E-02	0.155	0.035	**7.10E-06**	0.007	0.074	9.26E-01
rs2535303	6	31163402	A/G	*HCG22 | C6orf15*	INTERGENIC	2	1.840	1.574	2.152	**2.10E-14**	0.027	0.032	4.00E-01	-0.139	0.122	2.59E-01	-0.046	0.032	1.50E-01	0.061	0.122	6.18E-01	0.063	0.032	5.00E-02	-0.122	0.073	9.85E-02
rs2239803	6	32519811	A/G	*HLA-DRA*	INTRON	2	0.518	0.438	0.613	**2.20E-14**	0.075	0.032	**1.90E-02**	0.004	0.123	9.75E-01	-0.105	0.031	**8.50E-04**	0.192	0.120	1.15E-01	0.182	0.032	**8.80E-09**	0.041	0.074	5.83E-01
rs3869109	6	31292175	A/G	*HCG27 | HLA-C*	INTERGENIC	2	1.831	1.568	2.139	**2.24E-14**	-0.040	0.031	2.00E-01	-0.030	0.124	8.08E-01	0.183	0.031	**3.20E-09**	-0.153	0.122	2.16E-01	-0.231	0.031	**1.20E-13**	0.098	0.074	1.86E-01
rs2233966	6	31188838	G/A	*C6orf15 | PSORS1C1*	INTERGENIC	2	1.829	1.566	2.136	**2.28E-14**	0.029	0.032	3.70E-01	-0.128	0.122	3.00E-01	-0.036	0.032	2.60E-01	0.089	0.122	4.66E-01	0.054	0.032	9.10E-02	-0.110	0.073	1.35E-01
rs9263964	6	31294018	G/A	*HCG27 | HLA-C*	INTERGENIC	2	1.831	1.567	2.138	**2.30E-14**	-0.070	0.031	**2.40E-02**	-0.030	0.124	8.08E-01	0.197	0.031	**1.70E-10**	-0.153	0.122	2.16E-01	-0.274	0.031	**9.10E-19**	0.098	0.074	1.86E-01
rs9263967	6	31294224	A/G	*HCG27 | HLA-C*	INTERGENIC	2	1.831	1.567	2.138	**2.32E-14**	-0.040	0.031	2.00E-01	-0.030	0.124	8.08E-01	0.183	0.031	**3.20E-09**	-0.153	0.122	2.16E-01	-0.231	0.031	**1.20E-13**	0.098	0.074	1.86E-01
rs209181	6	28900456	A/G	*LOC442181 | LOC401242*	INTERGENIC	2	2.031	1.692	2.437	**2.60E-14**	-0.013	0.043	7.70E-01	0.003	0.123	9.80E-01	0.178	0.043	**3.10E-05**	-0.129	0.121	2.94E-01	-0.205	0.043	**2.10E-06**	0.001	0.074	9.91E-01
rs720465	6	31233756	A/C	*CCHCR1*	INTRON	2	1.850	1.579	2.168	**2.61E-14**	0.002	0.034	9.50E-01	-0.062	0.123	6.17E-01	0.096	0.034	**4.60E-03**	-0.067	0.122	5.83E-01	-0.096	0.034	**5.00E-03**	0.066	0.074	3.71E-01
rs2734574	6	31601867	A/T	*MICB | MCCD1*	INTERGENIC	2	1.789	1.540	2.079	**2.68E-14**	0.046	0.032	1.60E-01	-0.088	0.123	4.77E-01	0.049	0.032	1.23E-01	-0.183	0.120	1.32E-01	-0.010	0.032	7.70E-01	0.056	0.074	4.50E-01
rs3104389	6	32703075	A/C	*HLA-DRB1 | HLA-DQA1*	INTERGENIC	2	0.448	0.365	0.551	**2.73E-14**	-	-	-	0.004	0.123	9.77E-01	-	-	-	0.120	0.122	3.30E-01	-	-	-	-0.001	0.074	9.85E-01

rs535586	6	31968316	A/G	*EHMT2*	CODING	2	1.811	1.554	2.111	**2.79E-14**	-0.023	0.033	4.90E-01	-0.088	0.124	4.81E-01	0.223	0.032	**6.40E-12**	-0.103	0.123	4.08E-01	-0.255	0.033	**5.20E-15**	-0.041	0.075	5.87E-01
rs2516415	6	31567721	A/G	*3.8-1 | MICB*	INTERGENIC	2	1.808	1.551	2.106	**3.23E-14**	0.066	0.035	5.80E-02	-0.082	0.123	5.06E-01	0.076	0.034	**2.70E-02**	-0.005	0.122	9.70E-01	-0.017	0.035	6.20E-01	-0.079	0.074	2.88E-01
rs2251830	6	31124957	A/C	*LOC729792 | HCG22*	INTERGENIC	2	1.813	1.555	2.115	**3.30E-14**	-0.016	0.032	6.10E-01	-0.221	0.121	7.28E-02	0.004	0.032	8.90E-01	-0.018	0.124	8.85E-01	-0.009	0.032	7.70E-01	0.004	0.074	9.58E-01
rs2523864	6	31126525	A/G	*LOC729792 | HCG22*	INTERGENIC	2	1.813	1.555	2.115	**3.30E-14**	-0.016	0.032	6.10E-01	-0.221	0.121	7.28E-02	0.004	0.032	8.90E-01	-0.018	0.124	8.85E-01	-0.009	0.032	7.70E-01	0.004	0.074	9.58E-01
rs2428517	6	31128131	G/A	*LOC729792 | HCG22*	INTERGENIC	2	1.813	1.555	2.114	**3.32E-14**	-0.016	0.032	6.10E-01	-0.221	0.121	7.28E-02	0.004	0.032	8.90E-01	-0.018	0.124	8.85E-01	-0.009	0.032	7.70E-01	0.004	0.074	9.58E-01
rs9368716	6	32414068	A/G	*C6orf10*	INTRON	2	0.535	0.455	0.629	**3.43E-14**	0.099	0.032	**1.90E-03**	-0.024	0.123	8.46E-01	-0.151	0.032	**2.00E-06**	0.184	0.120	1.31E-01	0.246	0.032	**1.30E-14**	0.082	0.074	2.67E-01
rs477515	6	32677669	A/G	*HLA-DRB1 | HLA-DQA1*	INTERGENIC	2	0.461	0.378	0.563	**3.47E-14**	-	-	-	0.003	0.123	9.80E-01	-	-	-	0.139	0.121	2.56E-01	-	-	-	-0.019	0.074	7.93E-01
rs2523674	6	31544768	G/A	*HCP5 | 3.8-1*	INTERGENIC	2	1.800	1.546	2.096	**3.69E-14**	0.022	0.032	4.90E-01	-0.035	0.123	7.78E-01	0.030	0.032	3.50E-01	0.114	0.122	3.54E-01	-0.006	0.032	8.50E-01	-0.059	0.074	4.28E-01
rs2516500	6	31561619	A/G	*3.8-1 | MICB*	INTERGENIC	2	1.820	1.559	2.125	**3.70E-14**	0.048	0.035	1.70E-01	-0.019	0.123	8.77E-01	0.117	0.035	**7.10E-04**	0.046	0.122	7.10E-01	-0.076	0.035	**3.00E-02**	-0.087	0.074	2.43E-01
rs1264698	6	30175156	C/A	*RNF39 | TRIM31*	INTERGENIC	2	1.911	1.616	2.259	**3.84E-14**	0.020	0.036	5.70E-01	-0.129	0.122	2.96E-01	0.116	0.036	**1.09E-03**	-0.188	0.120	1.23E-01	-0.103	0.036	**4.10E-03**	-0.038	0.074	6.12E-01
rs16891725	6	26587129	A/G	*BTN2A1 | BTN1A1*	INTERGENIC	2	2.120	1.744	2.576	**4.35E-14**	0.083	0.046	6.90E-02	0.102	0.123	4.11E-01	0.146	0.045	**1.17E-03**	0.011	0.122	9.28E-01	-0.089	0.045	5.00E-02	-0.064	0.074	3.88E-01
rs4947342	6	32761048	A/G	*HLA-DQB1 | HLA-DQA2*	INTERGENIC	2	0.425	0.341	0.531	**4.35E-14**	0.053	0.038	1.60E-01	-0.015	0.124	9.02E-01	-0.071	0.037	5.60E-02	0.217	0.120	7.43E-02	0.124	0.037	**8.90E-04**	-0.057	0.074	4.40E-01
rs2253908	6	31444870	A/G	*LOC729816 | LOC100129668*	INTERGENIC	1	1.890	1.601	2.231	**5.64E-14**	0.061	0.034	7.80E-02	-0.025	0.124	8.44E-01	0.071	0.034	**3.80E-02**	-0.113	0.123	3.62E-01	-0.018	0.034	6.00E-01	0.000	0.075	9.98E-01
rs3130320	6	32331236	A/G	*NOTCH4 | C6orf10*	INTERGENIC	2	1.795	1.540	2.091	**6.14E-14**	-0.059	0.033	7.10E-02	-0.089	0.123	4.72E-01	0.196	0.032	**1.10E-09**	-0.228	0.119	5.95E-02	-0.266	0.032	**2.30E-16**	-0.041	0.074	5.80E-01
rs1610726	6	29846644	G/C	*IFITM4P | HCG4*	INTERGENIC	2	1.816	1.554	2.123	**6.89E-14**	0.002	0.034	9.50E-01	-0.045	0.123	7.13E-01	0.067	0.034	**4.70E-02**	-0.081	0.122	5.09E-01	-0.061	0.034	7.40E-02	0.028	0.074	7.01E-01
rs967005	6	28318667	A/G	*ZNF193 | ZKSCAN4*	INTERGENIC	2	1.931	1.625	2.293	**6.92E-14**	0.037	0.040	3.60E-01	0.017	0.125	8.93E-01	0.140	0.039	**3.80E-04**	-0.140	0.123	2.60E-01	-0.114	0.040	**4.10E-03**	0.110	0.074	1.39E-01
rs3868082	6	31315671	A/G	*HCG27 | HLA-C*	INTERGENIC	1	1.930	1.625	2.293	**7.13E-14**	-	-	-	-0.006	0.125	9.61E-01	-	-	-	-0.088	0.124	4.83E-01	-	-	-	0.103	0.074	1.68E-01
rs6923139	6	26421327	A/G	*HIST1H4H | LOC100132361*	INTERGENIC	2	2.197	1.788	2.701	**7.35E-14**	0.019	0.050	7.10E-01	0.098	0.123	4.27E-01	0.144	0.050	**3.70E-03**	-0.026	0.123	8.36E-01	-0.139	0.050	**5.30E-03**	-0.075	0.074	3.11E-01
rs2524084	6	31349618	G/A	*HLA-C | HLA-B*	INTERGENIC	2	1.784	1.532	2.078	**9.45E-14**	0.057	0.034	9.80E-02	-0.092	0.123	4.56E-01	0.102	0.034	**2.50E-03**	-0.049	0.122	6.89E-01	-0.052	0.034	1.24E-01	-0.018	0.074	8.07E-01
rs2248613	6	31556411	A/C	*3.8-1 | MICB*	INTERGENIC	2	1.803	1.544	2.106	**9.99E-14**	0.044	0.034	2.00E-01	0.070	0.124	5.73E-01	0.108	0.034	**1.27E-03**	0.190	0.121	1.22E-01	-0.069	0.034	**4.00E-02**	-0.097	0.075	1.98E-01
rs3115572	6	32328462	C/G	*NOTCH4 | C6orf10*	INTERGENIC	2	1.783	1.531	2.077	**1.03E-13**	-0.051	0.032	1.11E-01	-0.093	0.122	4.49E-01	0.185	0.032	**4.80E-09**	-0.221	0.119	6.84E-02	-0.248	0.032	**7.60E-15**	-0.058	0.074	4.35E-01
rs13207945	6	32687686	G/A	*HLA-DRB1 | HLA-DQA1*	INTERGENIC	2	0.536	0.455	0.632	**1.09E-13**	-	-	-	-0.079	0.129	5.42E-01	-	-	-	0.002	0.129	9.87E-01	-	-	-	0.012	0.075	8.75E-01
rs2736426	6	31853263	G/A	*C6orf27 | VARS*	INTERGENIC	2	0.544	0.463	0.639	**1.09E-13**	-0.031	0.032	3.30E-01	0.094	0.123	4.44E-01	0.032	0.032	3.20E-01	0.076	0.122	5.36E-01	-0.060	0.032	6.30E-02	0.052	0.074	4.83E-01
rs532098	6	32686030	A/G	*HLA-DRB1 | HLA-DQA1*	INTERGENIC	2	0.536	0.455	0.632	**1.09E-13**	-	-	-	-0.079	0.129	5.42E-01	-	-	-	0.002	0.129	9.87E-01	-	-	-	0.012	0.075	8.75E-01
rs9271588	6	32698931	G/A	*HLA-DRB1 | HLA-DQA1*	INTERGENIC	2	0.537	0.456	0.633	**1.20E-13**	-	-	-	0.042	0.123	7.35E-01	-	-	-	0.109	0.122	3.75E-01	-	-	-	-0.017	0.074	8.23E-01
rs2844575	6	31442924	G/A	*LOC729816 | LOC100129668*	INTERGENIC	2	0.543	0.463	0.639	**1.23E-13**	0.114	0.031	**2.80E-04**	0.031	0.123	8.02E-01	-0.184	0.031	**3.00E-09**	0.187	0.120	1.23E-01	0.299	0.031	**1.20E-21**	-0.001	0.074	9.92E-01
rs9467704	6	26427465	A/G	*HIST1H4H | LOC100132361*	INTERGENIC	2	2.196	1.783	2.705	**1.31E-13**	0.034	0.053	5.20E-01	0.098	0.123	4.27E-01	0.181	0.053	**5.90E-04**	-0.026	0.123	8.36E-01	-0.165	0.053	**1.90E-03**	-0.075	0.074	3.11E-01
rs1633030	6	29853773	G/A	*IFITM4P | HCG4*	INTERGENIC	2	1.809	1.546	2.116	**1.33E-13**	0.002	0.034	9.50E-01	-0.040	0.124	7.47E-01	0.067	0.034	**4.70E-02**	-0.079	0.123	5.25E-01	-0.061	0.034	7.40E-02	0.030	0.074	6.88E-01
rs1633010	6	29870921	T/A	*HCG4 | HLA-G*	INTERGENIC	2	1.803	1.542	2.108	**1.37E-13**	0.003	0.034	9.20E-01	-0.070	0.123	5.70E-01	0.069	0.034	**4.10E-02**	-0.098	0.122	4.22E-01	-0.062	0.034	6.90E-02	0.021	0.074	7.73E-01
rs1632983	6	29881488	A/G	*HCG4 | HLA-G*	INTERGENIC	2	1.803	1.542	2.107	**1.41E-13**	-	-	-	-0.070	0.123	5.70E-01	-	-	-	-0.098	0.122	4.22E-01	-	-	-	0.021	0.074	7.73E-01
rs1736991	6	29874905	C/G	*HCG4 | HLA-G*	INTERGENIC	2	1.803	1.542	2.107	**1.41E-13**	-	-	-	-0.070	0.123	5.70E-01	-	-	-	-0.098	0.122	4.22E-01	-	-	-	0.021	0.074	7.73E-01
rs1633041	6	29841202	A/G	*IFITM4P | HCG4*	INTERGENIC	2	1.809	1.546	2.117	**1.45E-13**	0.005	0.035	8.80E-01	-0.025	0.123	8.40E-01	0.099	0.034	**4.00E-03**	-0.071	0.122	5.62E-01	-0.097	0.035	**5.40E-03**	0.049	0.074	5.05E-01
rs1737041	6	29844208	A/C	*IFITM4P | HCG4*	INTERGENIC	2	1.809	1.546	2.117	**1.46E-13**	0.003	0.035	9.40E-01	-0.025	0.123	8.40E-01	0.100	0.035	**3.90E-03**	-0.071	0.122	5.62E-01	-0.100	0.035	**4.40E-03**	0.049	0.074	5.05E-01
rs1264697	6	30175547	C/G	*RNF39 | TRIM31*	INTERGENIC	2	1.880	1.590	2.222	**1.50E-13**	0.020	0.036	5.70E-01	-0.129	0.122	2.96E-01	0.116	0.036	**1.09E-03**	-0.188	0.120	1.23E-01	-0.103	0.036	**4.10E-03**	-0.038	0.074	6.12E-01
rs29218	6	29715408	G/A	*GABBR1 | MOG*	INTERGENIC	2	1.879	1.589	2.221	**1.50E-13**	0.007	0.039	8.50E-01	-0.033	0.123	7.90E-01	0.143	0.039	**2.00E-04**	-0.053	0.122	6.66E-01	-0.153	0.039	**8.30E-05**	-0.035	0.075	6.35E-01
rs1264696	6	30175813	A/G	*RNF39 | TRIM31*	INTERGENIC	2	1.879	1.590	2.222	**1.52E-13**	-	-	-	-0.129	0.122	2.96E-01	-	-	-	-0.188	0.120	1.23E-01	-	-	-	-0.038	0.074	6.12E-01
rs9276711	6	32865275	A/G	*HLA-DQB2 | HLA-DOB*	INTERGENIC	1	1.900	1.602	2.253	**1.58E-13**	-	-	-	0.010	0.126	9.38E-01	-	-	-	-0.184	0.123	1.40E-01	-	-	-	0.023	0.075	7.59E-01
rs1736969	6	29884369	A/T	*HCG4 | HLA-G*	INTERGENIC	2	1.799	1.539	2.103	**1.69E-13**	0.005	0.035	8.90E-01	-0.025	0.123	8.40E-01	0.101	0.034	**3.30E-03**	-0.071	0.122	5.62E-01	-0.099	0.035	**4.40E-03**	0.049	0.074	5.05E-01
rs2523535	6	31444229	G/A	*LOC729816 | LOC100129668*	INTERGENIC	2	1.756	1.511	2.039	**1.77E-13**	0.092	0.032	**4.50E-03**	0.038	0.124	7.62E-01	-0.004	0.032	9.10E-01	0.080	0.123	5.20E-01	0.091	0.032	**4.60E-03**	0.055	0.074	4.62E-01
rs735765	6	28278276	A/G	*ZNF192 | LOC222699*	INTERGENIC	1	2.180	1.772	2.682	**1.78E-13**	0.061	0.044	1.60E-01	0.106	0.123	3.94E-01	0.154	0.043	**3.50E-04**	-0.016	0.123	8.96E-01	-0.109	0.044	**1.22E-02**	-0.060	0.074	4.16E-01
rs2857218	6	32963318	C/G	*PSMB9 | HLA-DMB*	INTERGENIC	2	1.754	1.510	2.036	**1.81E-13**	0.055	0.033	9.60E-02	0.087	0.123	4.79E-01	0.000	0.033	9.90E-01	-0.120	0.121	3.25E-01	0.041	0.033	2.10E-01	0.030	0.074	6.85E-01
rs1108052	6	30177774	C/A	*RNF39 | TRIM31*	INTERGENIC	2	1.864	1.579	2.201	**1.86E-13**	-	-	-	-0.129	0.122	2.96E-01	-	-	-	-0.188	0.120	1.23E-01	-	-	-	-0.038	0.074	6.12E-01
rs1264695	6	30175871	G/A	*RNF39 | TRIM31*	INTERGENIC	2	1.864	1.579	2.201	**1.86E-13**	0.020	0.036	5.70E-01	-0.129	0.122	2.96E-01	0.116	0.036	**1.09E-03**	-0.188	0.120	1.23E-01	-0.103	0.036	**4.10E-03**	-0.038	0.074	6.12E-01
rs1264703	6	30173395	C/G	*RNF39 | TRIM31*	INTERGENIC	2	1.875	1.586	2.217	**1.87E-13**	0.031	0.036	3.90E-01	-0.126	0.122	3.09E-01	0.135	0.036	**1.50E-04**	-0.180	0.121	1.41E-01	-0.114	0.036	**1.50E-03**	-0.037	0.074	6.22E-01
rs71557344	6	26745703	G/A	*ZNF322A*	CODING	1	2.380	1.889	2.999	**1.89E-13**	-	-	-	0.073	0.123	5.54E-01	-	-	-	-0.096	0.122	4.35E-01	-	-	-	-0.032	0.075	6.70E-01
rs3130316	6	32329206	A/G	*NOTCH4 | C6orf10*	INTERGENIC	2	1.784	1.529	2.082	**1.90E-13**	-0.051	0.032	1.11E-01	-0.093	0.122	4.49E-01	0.185	0.032	**4.80E-09**	-0.221	0.119	6.84E-02	-0.248	0.032	**7.60E-15**	-0.058	0.074	4.35E-01
rs6920256	6	26645780	A/G	*BTN1A1 | HMGN4*	INTERGENIC	2	2.065	1.702	2.505	**1.90E-13**	-	-	-	0.102	0.123	4.11E-01	-	-	-	0.011	0.122	9.28E-01	-	-	-	-0.064	0.074	3.88E-01
rs9468317	6	28306435	G/A	*ZNF193*	INTRON	2	1.889	1.594	2.238	**2.02E-13**	0.037	0.040	3.60E-01	0.017	0.125	8.93E-01	0.140	0.039	**3.80E-04**	-0.140	0.123	2.60E-01	-0.114	0.040	**4.10E-03**	0.110	0.074	1.39E-01
rs9295768	6	28317081	A/G	*ZNF193 | ZKSCAN4*	INTERGENIC	2	1.889	1.594	2.238	**2.03E-13**	0.037	0.040	3.60E-01	0.017	0.125	8.93E-01	0.140	0.039	**3.80E-04**	-0.140	0.123	2.60E-01	-0.114	0.040	**4.10E-03**	0.110	0.074	1.39E-01
rs2893910	6	27391233	A/T	*POM121L2 | FKSG83*	INTERGENIC	2	1.960	1.637	2.346	**2.21E-13**	0.005	0.040	9.10E-01	0.021	0.124	8.64E-01	0.066	0.040	9.80E-02	-0.084	0.122	4.94E-01	-0.051	0.040	2.10E-01	-0.083	0.075	2.69E-01
rs1383267	6	32941624	A/G	*PSMB9 | HLA-DMB*	INTERGENIC	2	1.763	1.515	2.052	**2.33E-13**	0.134	0.033	**4.10E-05**	-0.015	0.123	9.03E-01	0.040	0.032	2.20E-01	-0.197	0.120	1.04E-01	0.071	0.032	**2.80E-02**	-0.026	0.074	7.23E-01
rs7767288	6	32937991	A/G	*PSMB9 | HLA-DMB*	INTERGENIC	2	1.763	1.515	2.052	**2.33E-13**	0.132	0.033	**4.80E-05**	-0.015	0.123	9.03E-01	0.039	0.032	2.30E-01	-0.197	0.120	1.04E-01	0.071	0.032	**2.80E-02**	-0.026	0.074	7.23E-01
rs1265052	6	31188450	A/G	*C6orf15 | PSORS1C1*	INTERGENIC	2	0.558	0.477	0.652	**2.39E-13**	0.024	0.033	4.60E-01	0.144	0.122	2.40E-01	-0.055	0.032	8.80E-02	-0.045	0.122	7.17E-01	0.069	0.032	**3.30E-02**	0.125	0.073	9.08E-02
rs4634439	6	26705983	G/A	*ABT1*	INTRON	2	2.075	1.707	2.522	**2.39E-13**	0.090	0.048	5.90E-02	0.040	0.124	7.49E-01	0.215	0.047	**5.50E-06**	-0.065	0.123	6.01E-01	-0.149	0.048	**1.80E-03**	-0.056	0.074	4.52E-01
rs916569	6	30174076	A/G	*RNF39 | TRIM31*	INTERGENIC	2	1.880	1.588	2.227	**2.41E-13**	0.020	0.036	5.70E-01	-0.129	0.122	2.96E-01	0.116	0.036	**1.09E-03**	-0.188	0.120	1.23E-01	-0.103	0.036	**4.10E-03**	-0.038	0.074	6.12E-01
rs2239689	6	32138262	A/G	*TNXB*	INTRON	2	0.528	0.444	0.626	**2.78E-13**	0.077	0.035	**2.80E-02**	-0.041	0.123	7.42E-01	-0.051	0.035	1.44E-01	0.100	0.122	4.14E-01	0.115	0.035	**1.00E-03**	0.022	0.074	7.62E-01
rs1629068	6	29873270	G/A	*HCG4 | HLA-G*	INTERGENIC	2	1.801	1.538	2.109	**2.87E-13**	0.005	0.035	8.90E-01	-0.025	0.123	8.40E-01	0.101	0.034	**3.30E-03**	-0.071	0.122	5.62E-01	-0.099	0.035	**4.40E-03**	0.049	0.074	5.05E-01
rs1632987	6	29880528	G/C	*HCG4 | HLA-G*	INTERGENIC	2	1.801	1.538	2.109	**2.87E-13**	-	-	-	-0.025	0.123	8.40E-01	-	-	-	-0.071	0.122	5.62E-01	-	-	-	0.049	0.074	5.05E-01
rs3131073	6	29028951	A/G	*C6orf100 | ZNF311*	INTERGENIC	1	2.350	1.868	2.957	**3.03E-13**	-	-	-	0.070	0.128	5.86E-01	-	-	-	-0.099	0.127	4.40E-01	-	-	-	-0.036	0.077	6.35E-01
rs4713600	6	32930836	A/C	*PSMB9*	INTRON	2	1.763	1.514	2.053	**3.07E-13**	0.132	0.033	**4.80E-05**	-0.015	0.123	9.03E-01	0.039	0.032	2.30E-01	-0.197	0.120	1.04E-01	0.071	0.032	**2.80E-02**	-0.026	0.074	7.23E-01
rs1633021	6	29854848	G/A	*IFITM4P | HCG4*	INTERGENIC	2	1.825	1.552	2.145	**3.16E-13**	0.037	0.036	3.10E-01	-0.043	0.123	7.26E-01	0.120	0.036	**8.50E-04**	-0.050	0.122	6.81E-01	-0.094	0.036	**9.90E-03**	0.036	0.074	6.31E-01
rs2294878	6	32475773	A/C	*BTNL2*	INTRON	2	0.557	0.476	0.652	**3.22E-13**	-	-	-	-0.035	0.123	7.80E-01	-	-	-	0.097	0.122	4.32E-01	-	-	-	-0.007	0.074	9.24E-01
rs7766356	6	28508517	G/A	*ZSCAN23*	INTRON	2	1.925	1.614	2.296	**3.40E-13**	0.008	0.039	8.30E-01	-0.054	0.123	6.62E-01	0.110	0.038	**4.10E-03**	-0.122	0.122	3.18E-01	-0.108	0.039	**4.90E-03**	0.012	0.074	8.76E-01
rs2535238	6	29753017	A/C	*ZFP57*	UTR	2	1.831	1.556	2.156	**3.49E-13**	0.015	0.037	6.90E-01	-0.043	0.123	7.28E-01	0.102	0.037	**5.60E-03**	-0.058	0.123	6.36E-01	-0.098	0.037	**8.40E-03**	-0.052	0.074	4.82E-01
rs2747421	6	29753097	G/C	*ZFP57*	UTR	2	1.831	1.556	2.156	**3.49E-13**	0.015	0.037	6.90E-01	-0.043	0.123	7.28E-01	0.102	0.037	**5.60E-03**	-0.058	0.123	6.36E-01	-0.098	0.037	**8.40E-03**	-0.052	0.074	4.82E-01
rs9379897	6	26709505	G/A	*ABT1 | ZNF322A*	INTERGENIC	2	2.059	1.695	2.501	**3.52E-13**	0.090	0.048	5.90E-02	0.040	0.124	7.49E-01	0.215	0.047	**5.50E-06**	-0.065	0.123	6.01E-01	-0.149	0.048	**1.80E-03**	-0.056	0.074	4.52E-01
rs375984	6	29752481	A/G	*ZFP57*	INTRON	2	1.831	1.556	2.155	**3.56E-13**	0.015	0.037	6.90E-01	-0.043	0.123	7.28E-01	0.102	0.037	**5.60E-03**	-0.058	0.123	6.36E-01	-0.098	0.037	**8.40E-03**	-0.052	0.074	4.82E-01
rs3129073	6	29723801	G/A	*GABBR1 | MOG*	INTERGENIC	2	1.870	1.579	2.213	**3.72E-13**	0.007	0.039	8.50E-01	-0.018	0.123	8.82E-01	0.143	0.039	**2.00E-04**	-0.039	0.122	7.53E-01	-0.153	0.039	**8.30E-05**	-0.030	0.074	6.84E-01
rs9469220	6	32766288	A/G	*HLA-DQB1 | HLA-DQA2*	INTERGENIC	2	0.553	0.472	0.649	**3.74E-13**	0.086	0.032	**6.70E-03**	0.029	0.123	8.15E-01	-0.085	0.031	**6.80E-03**	0.219	0.119	7.07E-02	0.172	0.032	**5.00E-08**	-0.035	0.074	6.38E-01

rs1633034	6	29851542	G/A	*IFITM4P | HCG4*	INTERGENIC	2	1.779	1.523	2.080	**4.23E-13**	0.005	0.034	8.80E-01	-0.070	0.123	5.70E-01	0.066	0.034	**4.90E-02**	-0.098	0.122	4.22E-01	-0.057	0.034	9.10E-02	0.024	0.074	7.45E-01
rs1264702	6	30173554	G/A	*RNF39 | TRIM31*	INTERGENIC	2	1.866	1.576	2.210	**4.44E-13**	0.031	0.036	3.90E-01	-0.126	0.122	3.09E-01	0.135	0.036	**1.50E-04**	-0.180	0.121	1.41E-01	-0.114	0.036	**1.50E-03**	-0.037	0.074	6.22E-01
rs1117489	6	30278806	G/A	*TRIM26*	INTRON	1	2.060	1.694	2.505	**4.65E-13**	-	-	-	0.022	0.124	8.58E-01	-	-	-	-0.047	0.124	7.06E-01	-	-	-	-0.052	0.075	4.87E-01
rs41500444	6	32038420	C/A	*SKIV2L*	INTRON	2	1.752	1.505	2.039	**4.73E-13**	-	-	-	0.062	0.123	6.14E-01	-	-	-	0.075	0.122	5.44E-01	-	-	-	0.037	0.074	6.15E-01
rs3130573	6	31214247	G/A	*PSORS1C1*	INTRON	2	1.758	1.509	2.049	**4.75E-13**	-0.002	0.034	9.60E-01	-0.002	0.124	9.89E-01	0.124	0.034	**2.60E-04**	-0.238	0.120	5.01E-02	-0.134	0.034	**9.50E-05**	-0.006	0.074	9.36E-01
rs3763347	6	32930953	A/T	*PSMB9*	INTRON	2	1.754	1.506	2.043	**4.95E-13**	0.132	0.033	**4.80E-05**	-0.015	0.123	9.03E-01	0.039	0.032	2.30E-01	-0.197	0.120	1.04E-01	0.071	0.032	**2.80E-02**	-0.026	0.074	7.23E-01
rs2246618	6	31586965	A/G	*MICB | MCCD1*	INTERGENIC	1	1.870	1.578	2.217	**5.40E-13**	0.041	0.035	2.40E-01	-0.046	0.123	7.08E-01	0.082	0.035	**1.90E-02**	0.001	0.123	9.97E-01	-0.045	0.035	2.00E-01	-0.068	0.074	3.65E-01
rs2535292	6	31170393	A/G	*HCG22 | C6orf15*	INTERGENIC	1	1.870	1.578	2.217	**5.40E-13**	-0.033	0.033	3.20E-01	-0.139	0.122	2.59E-01	0.043	0.032	1.80E-01	0.061	0.122	6.18E-01	-0.065	0.032	**4.60E-02**	-0.122	0.073	9.85E-02
rs1736983	6	29876028	A/G	*HCG4 | HLA-G*	INTERGENIC	2	1.793	1.530	2.101	**5.42E-13**	0.012	0.035	7.20E-01	-0.025	0.123	8.40E-01	0.082	0.034	**1.70E-02**	-0.071	0.122	5.62E-01	-0.073	0.035	**3.50E-02**	0.049	0.074	5.05E-01
rs9268615	6	32510867	A/G	*BTNL2 | HLA-DRA*	INTERGENIC	2	0.533	0.450	0.633	**5.80E-13**	0.050	0.033	1.31E-01	0.005	0.123	9.70E-01	-0.064	0.032	**4.70E-02**	0.089	0.122	4.68E-01	0.113	0.033	**5.30E-04**	0.101	0.074	1.69E-01
rs17336532	6	28651243	A/G	*ZNF452*	CODING	2	1.884	1.586	2.238	**5.84E-13**	-0.033	0.038	3.90E-01	-0.085	0.123	4.94E-01	0.070	0.037	6.00E-02	-0.142	0.121	2.45E-01	-0.098	0.037	**8.80E-03**	-0.026	0.074	7.29E-01
rs2251396	6	31472686	A/G	*LOC729816 | LOC100129668*	INTERGENIC	2	1.750	1.502	2.038	**6.19E-13**	0.031	0.034	3.60E-01	-0.080	0.124	5.23E-01	0.031	0.034	3.70E-01	-0.271	0.120	**2.69E-02**	-0.004	0.034	9.10E-01	-0.023	0.075	7.54E-01
rs16901848	6	28654931	A/G	*ZNF452*	INTRON	2	1.846	1.562	2.181	**6.48E-13**	-0.047	0.037	2.10E-01	-0.109	0.123	3.76E-01	0.052	0.037	1.60E-01	-0.165	0.121	1.78E-01	-0.089	0.037	**1.70E-02**	-0.024	0.074	7.50E-01
rs2535310	6	31161236	C/A	*HCG22 | C6orf15*	INTERGENIC	1	1.890	1.589	2.248	**6.61E-13**	0.027	0.032	4.00E-01	-0.139	0.122	2.59E-01	-0.046	0.032	1.50E-01	0.061	0.122	6.18E-01	0.063	0.032	5.00E-02	-0.122	0.073	9.85E-02
rs241411	6	32969609	G/C	*PSMB9 | HLA-DMB*	INTERGENIC	2	1.738	1.495	2.022	**7.05E-13**	0.049	0.033	1.38E-01	0.097	0.123	4.34E-01	-0.002	0.033	9.40E-01	-0.113	0.122	3.60E-01	0.038	0.033	2.50E-01	0.032	0.074	6.70E-01
rs14004	6	32515687	A/C	*HLA-DRA*	UTR	2	0.535	0.451	0.635	**7.21E-13**	0.047	0.033	1.50E-01	0.021	0.123	8.67E-01	-0.063	0.032	5.10E-02	0.069	0.122	5.76E-01	0.110	0.033	**7.80E-04**	0.068	0.074	3.61E-01
rs3131063	6	30871735	A/G	*IER3 | DDR1*	INTERGENIC	2	1.752	1.503	2.042	**7.24E-13**	0.015	0.032	6.40E-01	-0.036	0.123	7.71E-01	0.069	0.032	**3.00E-02**	-0.090	0.122	4.62E-01	-0.049	0.032	1.26E-01	0.108	0.073	1.44E-01
rs2844647	6	31118992	A/G	*LOC729792*	INTRON	2	1.750	1.502	2.039	**7.39E-13**	0.001	0.032	9.80E-01	-0.150	0.123	2.27E-01	-0.001	0.031	9.70E-01	-0.003	0.124	9.79E-01	0.004	0.032	9.00E-01	-0.063	0.074	3.96E-01
rs4452638	6	27337244	A/G	*PRSS16 | POM121L2*	INTERGENIC	2	2.022	1.668	2.452	**7.66E-13**	0.039	0.047	4.00E-01	0.041	0.123	7.42E-01	0.222	0.046	**1.50E-06**	-0.125	0.122	3.07E-01	-0.200	0.047	**1.90E-05**	-0.014	0.075	8.53E-01
rs10484439	6	26417887	A/G	*HIST1H4H | LOC100132361*	INTERGENIC	2	2.170	1.755	2.683	**8.68E-13**	0.034	0.053	5.20E-01	0.095	0.123	4.45E-01	0.181	0.053	**5.90E-04**	-0.097	0.123	4.31E-01	-0.165	0.053	**1.90E-03**	-0.042	0.075	5.71E-01
rs2127676	6	32957819	C/G	*PSMB9 | HLA-DMB*	INTERGENIC	1	1.850	1.563	2.190	**8.82E-13**	-	-	-	0.057	0.123	6.46E-01	-	-	-	-0.137	0.121	2.64E-01	-	-	-	0.010	0.074	8.92E-01
rs13219354	6	27293643	G/A	*HIST1H2AH | PRSS16*	INTERGENIC	2	2.014	1.662	2.440	**8.87E-13**	0.047	0.047	3.20E-01	0.041	0.123	7.42E-01	0.204	0.047	**1.20E-05**	-0.125	0.122	3.07E-01	-0.174	0.047	**2.20E-04**	-0.015	0.075	8.45E-01
rs6912584	6	28417569	G/A	*ZNF323 | ZKSCAN3*	INTERGENIC	2	1.854	1.565	2.196	**9.25E-13**	-0.027	0.037	4.70E-01	-0.065	0.123	5.99E-01	0.071	0.037	5.60E-02	-0.128	0.121	2.97E-01	-0.093	0.037	**1.26E-02**	-0.003	0.074	9.73E-01
rs1110446	6	30178916	A/G	*TRIM31*	UTR	2	1.826	1.547	2.156	**1.12E-12**	0.021	0.036	5.70E-01	-0.110	0.122	3.70E-01	0.124	0.036	**5.20E-04**	-0.176	0.120	1.48E-01	-0.111	0.036	**2.10E-03**	-0.047	0.074	5.27E-01
rs9379851	6	26462759	C/A	*LOC100132361 | BTN3A2*	INTERGENIC	2	2.022	1.665	2.455	**1.18E-12**	0.056	0.047	2.30E-01	0.040	0.124	7.49E-01	0.166	0.047	**3.90E-04**	-0.065	0.123	6.01E-01	-0.131	0.047	**5.30E-03**	-0.071	0.075	3.44E-01
rs356971	6	30087776	C/A	*HCG9 | ZNRD1*	INTERGENIC	1	2.070	1.693	2.530	**1.22E-12**	0.003	0.040	9.40E-01	0.001	0.123	9.95E-01	0.174	0.040	**1.10E-05**	-0.143	0.121	2.45E-01	-0.186	0.040	**3.30E-06**	-0.025	0.075	7.42E-01
rs3094204	6	31199971	G/A	*CDSN | PSORS1C1*	INTERGENIC	2	0.574	0.492	0.669	**1.29E-12**	0.079	0.031	**1.06E-02**	0.007	0.123	9.53E-01	-0.203	0.031	**3.00E-11**	0.006	0.122	9.64E-01	0.290	0.031	**4.90E-21**	0.065	0.074	3.78E-01
rs9268657	6	32517634	A/G	*HLA-DRA*	INTRON	2	0.539	0.455	0.640	**1.35E-12**	0.050	0.033	1.23E-01	0.010	0.123	9.38E-01	-0.063	0.032	5.10E-02	0.090	0.122	4.63E-01	0.113	0.033	**5.50E-04**	0.070	0.074	3.41E-01
rs7746199	6	27369303	A/G	*PRSS16 | POM121L2*	INTERGENIC	1	2.050	1.680	2.501	**1.45E-12**	0.002	0.040	9.60E-01	0.021	0.124	8.64E-01	0.066	0.040	9.80E-02	-0.084	0.122	4.94E-01	-0.053	0.040	1.90E-01	-0.083	0.075	2.69E-01
rs9268589	6	32506180	A/G	*BTNL2 | HLA-DRA*	INTERGENIC	2	0.534	0.449	0.635	**1.45E-12**	-	-	-	0.010	0.123	9.38E-01	-	-	-	0.090	0.122	4.63E-01	-	-	-	0.070	0.074	3.41E-01
rs9268606	6	32508048	A/G	*BTNL2 | HLA-DRA*	INTERGENIC	2	0.534	0.449	0.636	**1.46E-12**	0.048	0.033	1.45E-01	0.010	0.123	9.38E-01	-0.064	0.032	**4.80E-02**	0.090	0.122	4.63E-01	0.111	0.033	**6.40E-04**	0.070	0.074	3.41E-01
rs9268645	6	32516505	G/C	*HLA-DRA*	INTRON	2	0.534	0.449	0.636	**1.46E-12**	0.047	0.033	1.50E-01	0.010	0.123	9.38E-01	-0.063	0.032	5.10E-02	0.090	0.122	4.63E-01	0.110	0.033	**7.80E-04**	0.070	0.074	3.41E-01
rs9268557	6	32497283	G/A	*BTNL2 | HLA-DRA*	INTERGENIC	2	0.559	0.475	0.656	**1.50E-12**	0.037	0.032	2.40E-01	0.076	0.123	5.38E-01	-0.073	0.032	**2.10E-02**	0.150	0.121	2.19E-01	0.111	0.032	**5.00E-04**	0.082	0.074	2.67E-01
rs9257794	6	29443640	A/G	*OR5V1 | OR12D3*	INTERGENIC	2	1.807	1.533	2.129	**1.59E-12**	-0.011	0.036	7.70E-01	0.044	0.124	7.22E-01	0.134	0.036	**1.80E-04**	0.000	0.124	9.98E-01	-0.156	0.036	**1.50E-05**	-0.055	0.074	4.62E-01
rs7766862	6	32140985	A/G	*TNXB*	INTRON	2	0.528	0.442	0.630	**1.61E-12**	0.077	0.035	**2.80E-02**	-0.041	0.123	7.42E-01	-0.051	0.035	1.44E-01	0.100	0.122	4.14E-01	0.115	0.035	**1.00E-03**	0.022	0.074	7.62E-01
rs6902493	6	32130876	A/G	*TNXB*	INTRON	2	0.528	0.442	0.630	**1.64E-12**	-	-	-	-0.041	0.123	7.42E-01	-	-	-	0.100	0.122	4.14E-01	-	-	-	0.022	0.074	7.62E-01
rs2187818	6	32503546	A/C	*BTNL2 | HLA-DRA*	INTERGENIC	2	0.535	0.450	0.636	**1.65E-12**	0.047	0.033	1.50E-01	0.010	0.123	9.38E-01	-0.064	0.032	**4.80E-02**	0.090	0.122	4.63E-01	0.111	0.033	**6.90E-04**	0.070	0.074	3.41E-01
rs9268585	6	32505381	C/A	*BTNL2 | HLA-DRA*	INTERGENIC	2	0.535	0.450	0.636	**1.65E-12**	-	-	-	0.010	0.123	9.38E-01	-	-	-	0.090	0.122	4.63E-01	-	-	-	0.070	0.074	3.41E-01
rs2857009	6	32127724	C/G	*TNXB*	INTRON	2	0.528	0.442	0.630	**1.66E-12**	-	-	-	-0.036	0.124	7.71E-01	-	-	-	0.045	0.124	7.18E-01	-	-	-	0.027	0.074	7.21E-01
rs2071295	6	32146678	A/G	*TNXB*	INTRON	2	0.528	0.442	0.630	**1.70E-12**	0.078	0.035	**2.50E-02**	-0.041	0.123	7.42E-01	-0.047	0.035	1.70E-01	0.100	0.122	4.14E-01	0.112	0.035	**1.23E-03**	0.022	0.074	7.62E-01
rs2844502	6	31553195	A/G	*3.8-1 | MICB*	INTERGENIC	2	1.726	1.483	2.009	**1.74E-12**	0.044	0.034	2.00E-01	0.027	0.123	8.29E-01	0.108	0.034	**1.27E-03**	0.183	0.121	1.35E-01	-0.069	0.034	**4.00E-02**	-0.093	0.074	2.10E-01
rs9273363	6	32734250	A/C	*HLA-DQA1 | HLA-DQB1*	INTERGENIC	2	1.756	1.501	2.053	**1.75E-12**	0.093	0.036	**8.70E-03**	-0.059	0.124	6.35E-01	0.082	0.035	**1.90E-02**	-0.156	0.122	2.05E-01	-0.012	0.035	7.30E-01	0.031	0.074	6.80E-01
rs3117301	6	29762679	A/G	*ZFP57 | HLA-F*	INTERGENIC	2	1.791	1.523	2.106	**1.86E-12**	0.018	0.037	6.30E-01	-0.057	0.124	6.45E-01	0.110	0.036	**2.50E-03**	-0.046	0.123	7.07E-01	-0.103	0.037	**4.90E-03**	-0.069	0.074	3.53E-01
rs2747457	6	29764396	C/A	*ZFP57 | HLA-F*	INTERGENIC	2	1.790	1.523	2.106	**1.89E-12**	0.018	0.037	6.30E-01	-0.057	0.124	6.45E-01	0.110	0.036	**2.50E-03**	-0.046	0.123	7.07E-01	-0.103	0.037	**4.90E-03**	-0.069	0.074	3.53E-01
rs12191230	6	33076576	A/G	*BRD2 | HLA-DOA*	INTERGENIC	2	1.775	1.513	2.083	**2.03E-12**	-0.015	0.037	6.80E-01	-0.012	0.123	9.22E-01	-0.007	0.037	8.60E-01	-0.080	0.122	5.14E-01	-0.004	0.037	9.10E-01	0.020	0.075	7.88E-01
rs12192713	6	33076834	A/G	*BRD2 | HLA-DOA*	INTERGENIC	2	1.775	1.513	2.083	**2.03E-12**	-0.016	0.037	6.60E-01	-0.012	0.123	9.22E-01	-0.006	0.037	8.80E-01	-0.080	0.122	5.14E-01	-0.006	0.037	8.70E-01	0.020	0.075	7.88E-01
rs3131932	6	31048307	A/G	*LOC100129065 | C6orf205*	INTERGENIC	2	1.725	1.482	2.009	**2.11E-12**	0.032	0.031	3.10E-01	-0.178	0.122	1.49E-01	0.002	0.031	9.60E-01	0.041	0.123	7.41E-01	0.027	0.031	3.90E-01	-0.019	0.074	7.92E-01
rs1610601	6	29808162	A/C	*HLA-F | FLJ35429*	INTERGENIC	2	1.795	1.525	2.113	**2.12E-12**	0.032	0.038	3.90E-01	-0.051	0.123	6.78E-01	0.140	0.037	**1.70E-04**	-0.037	0.122	7.61E-01	-0.127	0.037	**7.10E-04**	-0.026	0.074	7.31E-01
rs6921919	6	28433180	G/C	*ZNF323 | ZKSCAN3*	INTERGENIC	2	1.853	1.560	2.202	**2.19E-12**	-0.008	0.038	8.30E-01	-0.046	0.123	7.08E-01	0.104	0.037	**5.30E-03**	-0.116	0.121	3.43E-01	-0.115	0.038	**2.40E-03**	-0.010	0.074	8.97E-01
rs6922111	6	28433287	A/G	*ZNF323 | ZKSCAN3*	INTERGENIC	2	1.853	1.560	2.202	**2.19E-12**	-0.008	0.038	8.30E-01	-0.046	0.123	7.08E-01	0.104	0.037	**5.30E-03**	-0.116	0.121	3.43E-01	-0.115	0.038	**2.40E-03**	-0.010	0.074	8.97E-01
rs1633063	6	29834025	A/G	*IFITM4P | HCG4*	INTERGENIC	2	1.804	1.530	2.128	**2.22E-12**	0.028	0.037	4.60E-01	-0.051	0.123	6.78E-01	0.139	0.037	**1.70E-04**	-0.037	0.122	7.61E-01	-0.130	0.037	**5.00E-04**	-0.026	0.074	7.31E-01
rs2242664	6	31947310	G/A	*SLC44A4*	CODING	2	1.721	1.479	2.003	**2.22E-12**	-0.006	0.032	8.50E-01	-0.104	0.123	4.00E-01	0.212	0.031	**1.30E-11**	-0.179	0.121	1.43E-01	-0.233	0.032	**1.50E-13**	-0.092	0.074	2.17E-01
rs2242665	6	31947288	G/A	*SLC44A4*	CODING	2	1.721	1.479	2.003	**2.22E-12**	-0.006	0.032	8.50E-01	-0.104	0.123	4.00E-01	0.212	0.031	**1.30E-11**	-0.179	0.121	1.43E-01	-0.233	0.032	**1.50E-13**	-0.092	0.074	2.17E-01
rs241439	6	32905515	C/A	*TAP2*	INTRON	2	1.721	1.479	2.002	**2.25E-12**	0.014	0.033	6.70E-01	-0.018	0.123	8.86E-01	-0.043	0.033	1.80E-01	-0.182	0.120	1.36E-01	0.060	0.033	6.70E-02	0.072	0.074	3.29E-01
rs9276884	6	32952948	A/G	*PSMB9 | HLA-DMB*	INTERGENIC	1	1.840	1.552	2.182	**2.41E-12**	0.086	0.034	**1.04E-02**	0.068	0.124	5.83E-01	0.017	0.033	6.10E-01	-0.126	0.123	3.09E-01	0.057	0.034	8.90E-02	0.013	0.074	8.62E-01
rs1632996	6	29877486	T/A	*HCG4 | HLA-G*	INTERGENIC	2	1.742	1.491	2.035	**2.49E-12**	0.014	0.033	6.70E-01	-0.125	0.122	3.10E-01	0.037	0.033	2.70E-01	-0.130	0.121	2.90E-01	-0.015	0.033	6.40E-01	0.003	0.074	9.69E-01
rs9271488	6	32696978	A/C	*HLA-DRB1 | HLA-DQA1*	INTERGENIC	1	0.470	0.380	0.581	**2.57E-12**	-	-	-	0.004	0.123	9.77E-01	-	-	-	0.120	0.122	3.30E-01	-	-	-	-0.001	0.074	9.85E-01
rs577272	6	31945942	G/A	*SLC44A4*	INTRON	2	1.728	1.482	2.015	**2.73E-12**	-0.006	0.032	8.50E-01	-0.104	0.123	4.00E-01	0.212	0.031	**1.30E-11**	-0.179	0.121	1.43E-01	-0.233	0.032	**1.50E-13**	-0.092	0.074	2.17E-01
rs644774	6	31946469	G/A	*SLC44A4*	INTRON	2	1.728	1.482	2.015	**2.73E-12**	-0.006	0.032	8.50E-01	-0.104	0.123	4.00E-01	0.212	0.031	**1.30E-11**	-0.179	0.121	1.43E-01	-0.233	0.032	**1.50E-13**	-0.092	0.074	2.17E-01
rs644827	6	31946420	A/G	*SLC44A4*	CODING	2	1.728	1.482	2.015	**2.73E-12**	-0.006	0.032	8.50E-01	-0.104	0.123	4.00E-01	0.212	0.031	**1.30E-11**	-0.179	0.121	1.43E-01	-0.233	0.032	**1.50E-13**	-0.092	0.074	2.17E-01
rs2071293	6	32170665	A/G	*TNXB*	INTRON	2	0.536	0.450	0.638	**2.85E-12**	0.074	0.035	**3.30E-02**	-0.041	0.123	7.42E-01	-0.051	0.034	1.42E-01	0.100	0.122	4.14E-01	0.112	0.035	**1.26E-03**	0.022	0.074	7.62E-01
rs241437	6	32905662	G/A	*TAP2*	INTRON	2	1.729	1.482	2.016	**2.89E-12**	0.014	0.033	6.70E-01	-0.031	0.123	8.02E-01	-0.043	0.033	1.80E-01	-0.192	0.120	1.14E-01	0.060	0.033	6.70E-02	0.067	0.074	3.65E-01
rs9268499	6	32483673	A/G	*BTNL2 | HLA-DRA*	INTERGENIC	2	0.497	0.408	0.605	**2.92E-12**	0.064	0.034	6.10E-02	-0.016	0.123	8.97E-01	-0.098	0.034	**3.70E-03**	0.156	0.121	2.02E-01	0.167	0.034	**8.70E-07**	0.039	0.074	5.96E-01
rs2246954	6	31373241	A/G	*HLA-C | HLA-B*	INTERGENIC	2	1.731	1.483	2.020	**3.20E-12**	-0.051	0.032	1.09E-01	0.092	0.123	4.59E-01	0.159	0.032	**4.70E-07**	-0.099	0.122	4.20E-01	-0.220	0.032	**4.40E-12**	0.118	0.074	1.13E-01
rs2844549	6	31451853	A/G	*LOC729816 | LOC100129668*	INTERGENIC	1	1.800	1.526	2.124	**3.30E-12**	0.053	0.034	1.15E-01	0.055	0.124	6.55E-01	0.058	0.033	8.00E-02	-0.113	0.122	3.60E-01	-0.013	0.033	6.90E-01	0.023	0.075	7.58E-01
rs6934993	6	29445479	G/A	*OR5V1 | OR12D3*	INTERGENIC	2	1.806	1.529	2.134	**3.35E-12**	-0.011	0.036	7.70E-01	0.044	0.124	7.22E-01	0.134	0.036	**1.80E-04**	0.000	0.124	9.98E-01	-0.156	0.036	**1.50E-05**	-0.055	0.074	4.62E-01
rs7383606	6	32874571	A/G	*HLA-DQB2 | HLA-DOB*	INTERGENIC	2	1.720	1.476	2.004	**3.40E-12**	0.055	0.034	1.12E-01	-0.023	0.127	8.59E-01	0.064	0.034	6.10E-02	-0.169	0.124	1.78E-01	-0.021	0.034	5.40E-01	-0.002	0.075	9.84E-01
rs2844796	6	30180490	A/G	*TRIM31*	INTRON	2	1.804	1.528	2.130	**3.42E-12**	0.006	0.036	8.70E-01	-0.129	0.122	2.96E-01	0.120	0.036	**7.60E-04**	-0.188	0.120	1.23E-01	-0.119	0.036	**9.20E-04**	-0.022	0.074	7.70E-01
rs1383261	6	32873429	A/G	*HLA-DQB2 | HLA-DOB*	INTERGENIC	2	1.720	1.476	2.003	**3.43E-12**	0.055	0.034	1.12E-01	-0.023	0.127	8.59E-01	0.064	0.034	6.10E-02	-0.169	0.124	1.78E-01	-0.021	0.034	5.40E-01	-0.002	0.075	9.84E-01

rs2072110	6	30178157	G/A	*RNF39 | TRIM31*	INTERGENIC	2	1.799	1.525	2.123	**3.46E-12**	-	-	-	-0.129	0.122	2.96E-01	-	-	-	-0.188	0.120	1.23E-01	-	-	-	-0.029	0.074	6.97E-01
rs7381376	6	32875651	G/C	*HLA-DQB2 | HLA-DOB*	INTERGENIC	2	1.719	1.476	2.003	**3.49E-12**	0.055	0.034	1.12E-01	-0.023	0.127	8.59E-01	0.064	0.034	6.10E-02	-0.169	0.124	1.78E-01	-0.021	0.034	5.40E-01	-0.002	0.075	9.84E-01
rs2240071	6	30178911	C/G	*TRIM31*	UTR	2	1.811	1.532	2.141	**3.59E-12**	0.022	0.036	5.50E-01	-0.110	0.122	3.70E-01	0.123	0.036	**5.60E-04**	-0.176	0.120	1.48E-01	-0.109	0.036	**2.50E-03**	-0.047	0.074	5.27E-01
rs9268457	6	32458014	G/A	*C6orf10 | BTNL2*	INTERGENIC	2	0.469	0.379	0.581	**3.60E-12**	-	-	-	-0.016	0.123	8.97E-01	-	-	-	0.156	0.121	2.02E-01	-	-	-	0.100	0.074	1.80E-01
rs3131622	6	31528479	C/A	*LOC100129668 | HCP5*	INTERGENIC	2	1.698	1.463	1.972	**3.75E-12**	-0.018	0.032	5.60E-01	-0.087	0.123	4.80E-01	0.006	0.031	8.40E-01	-0.186	0.120	1.27E-01	-0.023	0.031	4.60E-01	-0.034	0.074	6.46E-01
rs2248617	6	31556512	A/G	*3.8-1 | MICB*	INTERGENIC	2	1.726	1.479	2.013	**3.91E-12**	0.044	0.034	2.00E-01	0.027	0.123	8.29E-01	0.108	0.034	**1.27E-03**	0.183	0.121	1.35E-01	-0.069	0.034	**4.00E-02**	-0.093	0.074	2.10E-01
rs2395488	6	31553888	G/A	*3.8-1 | MICB*	INTERGENIC	2	1.726	1.479	2.013	**3.91E-12**	0.044	0.034	2.00E-01	0.027	0.123	8.29E-01	0.108	0.034	**1.27E-03**	0.183	0.121	1.35E-01	-0.069	0.034	**4.00E-02**	-0.093	0.074	2.10E-01
rs2596536	6	31555827	A/G	*3.8-1 | MICB*	INTERGENIC	2	1.726	1.479	2.013	**3.91E-12**	0.044	0.034	2.00E-01	0.027	0.123	8.29E-01	0.108	0.034	**1.27E-03**	0.183	0.121	1.35E-01	-0.069	0.034	**4.00E-02**	-0.093	0.074	2.10E-01
rs2248289	6	30180700	G/A	*TRIM31*	INTRON	2	1.805	1.528	2.133	**4.05E-12**	0.006	0.036	8.70E-01	-0.129	0.122	2.96E-01	0.120	0.036	**7.60E-04**	-0.188	0.120	1.23E-01	-0.119	0.036	**9.20E-04**	-0.022	0.074	7.70E-01
rs1362068	6	29850087	G/A	*IFITM4P | HCG4*	INTERGENIC	2	1.724	1.478	2.011	**4.49E-12**	0.012	0.033	7.20E-01	-0.125	0.122	3.10E-01	0.034	0.033	3.00E-01	-0.130	0.121	2.90E-01	-0.014	0.033	6.60E-01	0.006	0.074	9.37E-01
rs9268542	6	32492699	G/A	*BTNL2 | HLA-DRA*	INTERGENIC	2	0.542	0.456	0.645	**4.49E-12**	0.059	0.033	7.30E-02	0.008	0.123	9.46E-01	-0.068	0.033	**3.80E-02**	0.114	0.122	3.51E-01	0.128	0.033	**9.40E-05**	0.067	0.074	3.68E-01
rs9268556	6	32494942	G/A	*BTNL2 | HLA-DRA*	INTERGENIC	2	0.542	0.456	0.645	**4.49E-12**	0.059	0.033	7.30E-02	0.008	0.123	9.46E-01	-0.068	0.033	**3.80E-02**	0.114	0.122	3.51E-01	0.128	0.033	**9.40E-05**	0.067	0.074	3.68E-01
rs885937	6	29877042	T/A	*HCG4 | HLA-G*	INTERGENIC	2	1.735	1.484	2.027	**4.61E-12**	0.014	0.033	6.70E-01	-0.125	0.122	3.10E-01	0.037	0.033	2.70E-01	-0.130	0.121	2.90E-01	-0.015	0.033	6.40E-01	0.005	0.074	9.46E-01
rs1362070	6	29850278	G/A	*IFITM4P | HCG4*	INTERGENIC	2	1.734	1.484	2.027	**4.65E-12**	0.011	0.033	7.40E-01	-0.125	0.122	3.10E-01	0.041	0.033	2.10E-01	-0.130	0.121	2.90E-01	-0.023	0.033	4.90E-01	0.005	0.074	9.46E-01
rs9265198	6	31397689	G/A	*HLA-C | HLA-B*	INTERGENIC	1	1.840	1.548	2.187	**4.67E-12**	-	-	-	-0.067	0.123	5.87E-01	-	-	-	-0.019	0.122	8.78E-01	-	-	-	-0.062	0.074	4.05E-01
rs2523457	6	31473686	A/G	*LOC729816 | LOC100129668*	INTERGENIC	2	1.714	1.471	1.997	**4.79E-12**	0.025	0.034	4.70E-01	-0.095	0.125	4.49E-01	0.016	0.033	6.20E-01	-0.249	0.121	**4.33E-02**	0.008	0.034	8.00E-01	-0.030	0.075	6.90E-01
rs4151657	6	32025519	G/A	*CFB*	INTRON	2	0.534	0.446	0.638	**4.94E-12**	0.035	0.034	3.10E-01	0.066	0.123	5.93E-01	-0.100	0.034	**3.10E-03**	0.104	0.122	3.94E-01	0.133	0.034	**9.60E-05**	0.024	0.074	7.44E-01
rs1632926	6	30069253	G/A	*HCG9 | ZNRD1*	INTERGENIC	1	2.050	1.672	2.513	**4.98E-12**	0.009	0.039	8.10E-01	0.011	0.125	9.31E-01	0.176	0.039	**6.00E-06**	-0.138	0.123	2.63E-01	-0.182	0.039	**3.50E-06**	-0.027	0.076	7.23E-01
rs10807100	6	32411046	A/G	*C6orf10*	INTRON	2	0.564	0.479	0.664	**5.39E-12**	-	-	-	-0.011	0.123	9.29E-01	-	-	-	0.053	0.122	6.66E-01	-	-	-	0.101	0.074	1.71E-01
rs2071534	6	32932296	A/G	*PSMB9*	INTRON	2	1.712	1.469	1.994	**5.42E-12**	0.110	0.032	**6.00E-04**	0.041	0.124	7.39E-01	0.038	0.032	2.30E-01	-0.129	0.122	2.94E-01	0.053	0.032	9.80E-02	-0.046	0.074	5.35E-01
rs3763348	6	32930885	A/G	*PSMB9*	INTRON	2	1.712	1.469	1.994	**5.42E-12**	0.102	0.032	**1.50E-03**	0.041	0.124	7.39E-01	0.041	0.032	2.00E-01	-0.129	0.122	2.94E-01	0.044	0.032	1.70E-01	-0.046	0.074	5.35E-01
rs3763346	6	32931235	G/A	*PSMB9*	INTRON	2	1.712	1.469	1.994	**5.50E-12**	0.110	0.032	**6.00E-04**	0.041	0.124	7.39E-01	0.038	0.032	2.30E-01	-0.129	0.122	2.94E-01	0.053	0.032	9.80E-02	-0.046	0.074	5.35E-01
rs2071476	6	32933357	G/A	*PSMB9*	INTRON	2	1.711	1.469	1.994	**5.53E-12**	0.110	0.032	**6.00E-04**	0.041	0.124	7.39E-01	0.038	0.032	2.30E-01	-0.129	0.122	2.94E-01	0.053	0.032	9.80E-02	-0.046	0.074	5.35E-01
rs6930981	6	32934428	G/A	*PSMB9*	INTRON	2	1.711	1.469	1.994	**5.53E-12**	0.110	0.032	**6.00E-04**	0.041	0.124	7.39E-01	0.038	0.032	2.30E-01	-0.129	0.122	2.94E-01	0.053	0.032	9.80E-02	-0.046	0.074	5.35E-01
rs539703	6	32396440	C/A	*C6orf10*	INTRON	2	0.564	0.480	0.664	**5.57E-12**	0.084	0.033	**1.06E-02**	-0.011	0.123	9.29E-01	-0.115	0.033	**4.20E-04**	0.053	0.122	6.66E-01	0.191	0.033	**6.40E-09**	0.101	0.074	1.71E-01
rs9268528	6	32491086	G/A	*BTNL2 | HLA-DRA*	INTERGENIC	2	0.544	0.457	0.647	**5.85E-12**	0.059	0.033	7.30E-02	0.008	0.123	9.46E-01	-0.068	0.033	**3.80E-02**	0.114	0.122	3.51E-01	0.128	0.033	**9.40E-05**	0.074	0.074	3.19E-01
rs3021061	6	32759817	G/C	*HLA-DQB1 | HLA-DQA2*	INTERGENIC	1	0.430	0.338	0.547	**6.05E-12**	-	-	-	-0.015	0.124	9.02E-01	-	-	-	0.217	0.120	7.43E-02	-	-	-	-0.057	0.074	4.40E-01
rs3130980	6	31190383	A/G	*C6orf15 | PSORS1C1*	INTERGENIC	2	0.535	0.447	0.640	**6.65E-12**	0.090	0.034	**7.50E-03**	0.103	0.122	4.01E-01	-0.169	0.033	**3.60E-07**	-0.118	0.121	3.33E-01	0.258	0.033	**1.20E-14**	0.114	0.073	1.22E-01
rs9501259	6	33163529	A/G	*HLA-DPB1 | HLA-DPB2*	INTERGENIC	2	2.128	1.715	2.641	**6.84E-12**	0.047	0.073	5.20E-01	0.017	0.123	8.89E-01	0.082	0.072	2.60E-01	-0.132	0.122	2.82E-01	-0.059	0.073	4.20E-01	-0.038	0.074	6.11E-01
rs3806156	6	32481676	A/C	*BTNL2*	INTRON	2	0.534	0.446	0.639	**7.87E-12**	0.062	0.033	6.40E-02	-0.108	0.123	3.84E-01	-0.103	0.033	**1.80E-03**	0.050	0.123	6.87E-01	0.171	0.033	**2.50E-07**	-0.022	0.074	7.63E-01
rs2076523	6	32478813	G/A	*BTNL2*	CODING	2	0.534	0.446	0.639	**7.88E-12**	0.062	0.033	6.40E-02	-0.108	0.123	3.84E-01	-0.103	0.033	**1.80E-03**	0.050	0.123	6.87E-01	0.171	0.033	**2.50E-07**	-0.022	0.074	7.63E-01
rs1736976	6	29881978	G/A	*HCG4 | HLA-G*	INTERGENIC	2	1.723	1.474	2.014	**8.07E-12**	0.014	0.033	6.70E-01	-0.107	0.122	3.84E-01	0.037	0.033	2.70E-01	-0.126	0.121	3.05E-01	-0.015	0.033	6.40E-01	0.024	0.074	7.50E-01
rs2143468	6	32416981	A/T	*C6orf10*	INTRON	2	0.572	0.487	0.671	**8.07E-12**	0.084	0.033	**1.09E-02**	-0.011	0.123	9.29E-01	-0.117	0.033	**3.70E-04**	0.053	0.122	6.66E-01	0.192	0.033	**5.40E-09**	0.100	0.074	1.78E-01
rs3817963	6	32476065	G/A	*BTNL2*	INTRON	2	0.491	0.400	0.602	**8.34E-12**	0.070	0.035	**4.40E-02**	-0.016	0.123	8.97E-01	-0.094	0.034	**6.00E-03**	0.156	0.121	2.02E-01	0.168	0.035	**1.10E-06**	0.039	0.074	5.96E-01
rs3817966	6	32475825	G/A	*BTNL2*	INTRON	2	0.491	0.400	0.602	**8.34E-12**	0.070	0.035	**4.40E-02**	-0.016	0.123	8.97E-01	-0.094	0.034	**6.00E-03**	0.156	0.121	2.02E-01	0.168	0.035	**1.10E-06**	0.039	0.074	5.96E-01
rs1610647	6	29870047	G/A	*HCG4 | HLA-G*	INTERGENIC	2	1.727	1.476	2.020	**8.51E-12**	-	-	-	-0.125	0.122	3.10E-01	-	-	-	-0.130	0.121	2.90E-01	-	-	-	0.005	0.074	9.46E-01
rs1610663	6	29886605	A/G	*HCG4 | HLA-G*	INTERGENIC	2	1.722	1.473	2.013	**8.63E-12**	0.002	0.034	9.50E-01	-0.083	0.123	5.03E-01	0.073	0.034	**2.90E-02**	-0.104	0.122	3.98E-01	-0.071	0.034	**3.70E-02**	0.032	0.074	6.68E-01
rs2523408	6	29886088	C/A	*HCG4 | HLA-G*	INTERGENIC	2	1.722	1.473	2.013	**8.63E-12**	0.002	0.034	9.50E-01	-0.083	0.123	5.03E-01	0.073	0.034	**2.90E-02**	-0.104	0.122	3.98E-01	-0.071	0.034	**3.70E-02**	0.032	0.074	6.68E-01
rs2523454	6	31475844	A/G	*LOC729816 | LOC100129668*	INTERGENIC	2	1.711	1.467	1.997	**8.75E-12**	0.032	0.034	3.50E-01	-0.079	0.124	5.25E-01	0.031	0.033	3.60E-01	-0.202	0.121	9.91E-02	-0.001	0.034	9.90E-01	-0.033	0.075	6.62E-01
rs1611205	6	29867802	G/A	*HCG4*	UTR	2	1.731	1.479	2.027	**8.77E-12**	0.014	0.033	6.70E-01	-0.114	0.123	3.61E-01	0.037	0.033	2.70E-01	-0.115	0.123	3.54E-01	-0.015	0.033	6.40E-01	0.007	0.074	9.25E-01
rs2736428	6	31951903	A/G	*SLC44A4*	INTRON	2	0.540	0.453	0.645	**9.07E-12**	0.050	0.035	1.46E-01	0.050	0.123	6.86E-01	-0.067	0.034	**4.90E-02**	0.082	0.122	5.02E-01	0.113	0.035	**1.12E-03**	0.057	0.074	4.42E-01
rs3763311	6	32484154	A/G	*BTNL2 | HLA-DRA*	INTERGENIC	2	0.494	0.403	0.605	**1.01E-11**	0.070	0.035	**4.40E-02**	-0.016	0.123	8.97E-01	-0.094	0.034	**6.00E-03**	0.156	0.121	2.02E-01	0.168	0.035	**1.10E-06**	0.039	0.074	5.96E-01
rs1233585	6	28824324	G/A	*LOC442181 | LOC401242*	INTERGENIC	2	1.804	1.522	2.138	**1.05E-11**	-0.003	0.040	9.50E-01	0.020	0.123	8.73E-01	0.164	0.039	**3.40E-05**	-0.145	0.121	2.35E-01	-0.175	0.040	**1.20E-05**	0.155	0.073	**3.51E-02**
rs13196606	6	28478057	A/G	*ZSCAN12 | ZSCAN23*	INTERGENIC	2	1.780	1.507	2.102	**1.09E-11**	-0.016	0.036	6.40E-01	-0.088	0.123	4.78E-01	0.094	0.035	**8.00E-03**	-0.080	0.122	5.14E-01	-0.110	0.036	**2.00E-03**	-0.021	0.074	7.76E-01
rs16894091	6	28498116	A/T	*ZSCAN23*	INTRON	2	1.783	1.509	2.107	**1.16E-11**	-	-	-	-0.088	0.123	4.78E-01	-	-	-	-0.080	0.122	5.14E-01	-	-	-	-0.021	0.074	7.76E-01
rs9268516	6	32487467	A/G	*BTNL2 | HLA-DRA*	INTERGENIC	2	0.498	0.407	0.609	**1.25E-11**	0.069	0.035	**4.70E-02**	-0.016	0.123	8.97E-01	-0.098	0.034	**4.20E-03**	0.156	0.121	2.02E-01	0.171	0.034	**6.50E-07**	0.039	0.074	5.96E-01
rs16894106	6	28508318	A/G	*ZSCAN23*	INTRON	2	1.781	1.507	2.105	**1.26E-11**	-0.016	0.036	6.40E-01	-0.088	0.123	4.78E-01	0.094	0.035	**8.00E-03**	-0.080	0.122	5.14E-01	-0.110	0.036	**2.00E-03**	-0.021	0.074	7.76E-01
rs3806157	6	32481779	C/A	*BTNL2*	INTRON	2	0.541	0.453	0.646	**1.27E-11**	0.062	0.033	6.40E-02	-0.108	0.123	3.84E-01	-0.103	0.033	**1.80E-03**	0.050	0.123	6.87E-01	0.171	0.033	**2.50E-07**	-0.022	0.074	7.63E-01
rs2294880	6	32475700	G/A	*BTNL2*	INTRON	2	0.498	0.407	0.610	**1.28E-11**	0.070	0.035	**4.40E-02**	-0.016	0.123	8.97E-01	-0.094	0.034	**6.00E-03**	0.156	0.121	2.02E-01	0.168	0.035	**1.10E-06**	0.039	0.074	5.96E-01
rs2076525	6	32478594	G/A	*BTNL2*	INTRON	2	0.498	0.407	0.610	**1.30E-11**	0.070	0.035	**4.40E-02**	-0.016	0.123	8.97E-01	-0.094	0.034	**6.00E-03**	0.156	0.121	2.02E-01	0.168	0.035	**1.10E-06**	0.039	0.074	5.96E-01
rs3763307	6	32482600	A/T	*BTNL2*	INTRON	2	0.498	0.407	0.610	**1.30E-11**	0.070	0.035	**4.40E-02**	-0.016	0.123	8.97E-01	-0.094	0.034	**6.00E-03**	0.156	0.121	2.02E-01	0.168	0.035	**1.10E-06**	0.039	0.074	5.96E-01
rs3817962	6	32476292	A/C	*BTNL2*	INTRON	2	0.499	0.408	0.610	**1.31E-11**	0.070	0.035	**4.40E-02**	-0.016	0.123	8.97E-01	-0.094	0.034	**6.00E-03**	0.156	0.121	2.02E-01	0.168	0.035	**1.10E-06**	0.039	0.074	5.96E-01
rs9268474	6	32465143	G/A	*C6orf10 | BTNL2*	INTERGENIC	2	0.499	0.408	0.610	**1.31E-11**	0.070	0.035	**4.40E-02**	-0.016	0.123	8.97E-01	-0.094	0.034	**6.00E-03**	0.156	0.121	2.02E-01	0.168	0.035	**1.10E-06**	0.039	0.074	5.96E-01
rs9268480	6	32471822	A/G	*BTNL2*	CODING	2	0.499	0.407	0.610	**1.31E-11**	0.070	0.035	**4.40E-02**	-0.016	0.123	8.97E-01	-0.094	0.034	**6.00E-03**	0.156	0.121	2.02E-01	0.168	0.035	**1.10E-06**	0.039	0.074	5.96E-01
rs9268481	6	32472334	G/A	*BTNL2*	INTRON	2	0.499	0.407	0.610	**1.31E-11**	-	-	-	-0.016	0.123	8.97E-01	-	-	-	0.156	0.121	2.02E-01	-	-	-	0.039	0.074	5.96E-01
rs9268482	6	32475755	T/A	*BTNL2*	INTRON	2	0.499	0.408	0.610	**1.31E-11**	-	-	-	-0.016	0.123	8.97E-01	-	-	-	0.156	0.121	2.02E-01	-	-	-	0.039	0.074	5.96E-01
rs2076522	6	32479157	G/C	*BTNL2*	INTRON	2	0.499	0.408	0.610	**1.32E-11**	0.070	0.035	**4.40E-02**	-0.016	0.123	8.97E-01	-0.094	0.034	**6.00E-03**	0.156	0.121	2.02E-01	0.168	0.035	**1.10E-06**	0.039	0.074	5.96E-01
rs8180664	6	32455468	A/G	*C6orf10 | BTNL2*	INTERGENIC	2	0.499	0.408	0.610	**1.32E-11**	0.075	0.035	**3.10E-02**	-0.016	0.123	8.97E-01	-0.098	0.034	**4.30E-03**	0.156	0.121	2.02E-01	0.176	0.035	**4.00E-07**	0.039	0.074	5.96E-01
rs9268401	6	32449296	G/A	*C6orf10 | BTNL2*	INTERGENIC	2	0.499	0.408	0.610	**1.32E-11**	-	-	-	-0.016	0.123	8.97E-01	-	-	-	0.156	0.121	2.02E-01	-	-	-	0.039	0.074	5.96E-01
rs9268403	6	32449451	G/A	*C6orf10 | BTNL2*	INTERGENIC	2	0.499	0.408	0.610	**1.32E-11**	0.075	0.035	**3.10E-02**	-0.016	0.123	8.97E-01	-0.098	0.034	**4.30E-03**	0.156	0.121	2.02E-01	0.176	0.035	**4.00E-07**	0.039	0.074	5.96E-01
rs9268428	6	32452951	A/C	*C6orf10 | BTNL2*	INTERGENIC	2	0.499	0.408	0.610	**1.32E-11**	-	-	-	-0.016	0.123	8.97E-01	-	-	-	0.156	0.121	2.02E-01	-	-	-	0.039	0.074	5.96E-01
rs9268456	6	32457924	A/C	*C6orf10 | BTNL2*	INTERGENIC	2	0.499	0.408	0.610	**1.32E-11**	-	-	-	-0.016	0.123	8.97E-01	-	-	-	0.156	0.121	2.02E-01	-	-	-	0.039	0.074	5.96E-01
rs9268459	6	32458754	A/G	*C6orf10 | BTNL2*	INTERGENIC	2	0.499	0.408	0.610	**1.32E-11**	-	-	-	-0.016	0.123	8.97E-01	-	-	-	0.156	0.121	2.02E-01	-	-	-	0.039	0.074	5.96E-01
rs2280774	6	32036670	A/G	*SKIV2L*	INTRON	2	0.541	0.452	0.646	**1.35E-11**	0.045	0.035	1.90E-01	-0.003	0.123	9.83E-01	-0.074	0.034	**3.10E-02**	0.080	0.122	5.15E-01	0.113	0.035	**1.10E-03**	0.032	0.074	6.65E-01
rs9393714	6	26481719	A/C	*BTN3A2*	INTRON	2	1.962	1.614	2.385	**1.35E-11**	0.074	0.048	1.21E-01	0.040	0.124	7.49E-01	0.176	0.047	**1.90E-04**	-0.065	0.123	6.01E-01	-0.127	0.048	**7.60E-03**	-0.073	0.074	3.26E-01
rs7774981	6	28454889	G/A	*ZKSCAN3 | ZSCAN12*	INTERGENIC	2	1.779	1.505	2.103	**1.37E-11**	-0.016	0.036	6.60E-01	-0.114	0.124	3.62E-01	0.093	0.035	**8.30E-03**	-0.080	0.123	5.17E-01	-0.109	0.036	**2.20E-03**	-0.027	0.074	7.22E-01
rs1610699	6	29886966	C/A	*HCG4 | HLA-G*	INTERGENIC	2	1.715	1.466	2.007	**1.51E-11**	-	-	-	-0.080	0.124	5.21E-01	-	-	-	-0.102	0.123	4.08E-01	-	-	-	0.033	0.074	6.58E-01
rs1610602	6	29810040	A/C	*HLA-F | FLJ35429*	INTERGENIC	2	1.728	1.473	2.027	**1.87E-11**	0.035	0.034	3.10E-01	-0.064	0.123	6.05E-01	0.115	0.034	**7.20E-04**	0.020	0.122	8.69E-01	-0.092	0.034	**7.40E-03**	-0.040	0.075	5.93E-01
rs6903989	6	29410009	A/C	*OR5U1 | OR5V1*	INTERGENIC	2	1.780	1.504	2.106	**1.88E-11**	-0.021	0.037	5.70E-01	0.062	0.124	6.18E-01	0.070	0.036	5.60E-02	-0.073	0.123	5.58E-01	-0.101	0.037	**6.10E-03**	0.135	0.074	6.76E-02

rs1736915	6	29812295	A/G	*HLA-F | FLJ35429*	INTERGENIC	2	1.727	1.473	2.026	**1.89E-11**	0.034	0.034	3.30E-01	-0.064	0.123	6.05E-01	0.118	0.034	**5.20E-04**	0.020	0.122	8.69E-01	-0.095	0.034	**5.40E-03**	-0.040	0.075	5.93E-01
rs929160	6	29814772	T/A	*HLA-F | FLJ35429*	INTERGENIC	2	1.727	1.472	2.026	**1.94E-11**	0.055	0.036	1.24E-01	-0.064	0.123	6.05E-01	0.146	0.036	**4.00E-05**	0.020	0.122	8.69E-01	-0.111	0.036	**2.10E-03**	-0.040	0.075	5.93E-01
rs9380335	6	33133152	G/C	*HLA-DOA | HLA-DPA1*	INTERGENIC	2	1.865	1.554	2.239	**2.25E-11**	-	-	-	0.053	0.123	6.68E-01	-	-	-	0.016	0.122	8.97E-01	-	-	-	-0.001	0.074	9.92E-01
rs3077	6	33141000	G/A	*HLA-DPA1*	UTR	2	1.865	1.554	2.239	**2.26E-11**	-0.058	0.041	1.60E-01	0.053	0.123	6.68E-01	0.023	0.041	5.80E-01	0.016	0.122	8.97E-01	-0.082	0.041	**4.70E-02**	-0.001	0.074	9.92E-01
rs9268835	6	32536093	A/G	*HLA-DRA | HLA-DRB5*	INTERGENIC	2	0.491	0.398	0.605	**2.27E-11**	-	-	-	0.063	0.124	6.13E-01	-	-	-	0.133	0.122	2.78E-01	-	-	-	0.009	0.074	9.06E-01
rs9393713	6	26481657	A/G	*BTN3A2*	INTRON	2	1.938	1.597	2.353	**2.28E-11**	0.074	0.048	1.21E-01	0.040	0.124	7.49E-01	0.176	0.047	**1.90E-04**	-0.065	0.123	6.01E-01	-0.127	0.048	**7.60E-03**	-0.073	0.074	3.26E-01
rs2022533	6	32415238	C/A	*C6orf10*	INTRON	2	0.563	0.476	0.667	**2.30E-11**	-	-	-	-0.011	0.123	9.29E-01	-	-	-	0.053	0.122	6.66E-01	-	-	-	0.101	0.074	1.71E-01
rs524578	6	32403335	A/G	*LOC100131609 | C6orf10*	INTERGENIC	2	0.563	0.476	0.667	**2.30E-11**	0.084	0.033	**1.09E-02**	-0.011	0.123	9.29E-01	-0.117	0.033	**3.70E-04**	0.053	0.122	6.66E-01	0.192	0.033	**5.40E-09**	0.101	0.074	1.71E-01
rs6929776	6	32411489	A/G	*C6orf10*	INTRON	2	0.563	0.476	0.667	**2.30E-11**	0.084	0.033	**1.09E-02**	-0.011	0.123	9.29E-01	-0.117	0.033	**3.70E-04**	0.053	0.122	6.66E-01	0.192	0.033	**5.40E-09**	0.101	0.074	1.71E-01
rs9348880	6	32404758	A/G	*LOC100131609 | C6orf10*	INTERGENIC	2	0.563	0.476	0.667	**2.30E-11**	0.084	0.033	**1.09E-02**	-0.011	0.123	9.29E-01	-0.117	0.033	**3.70E-04**	0.053	0.122	6.66E-01	0.192	0.033	**5.40E-09**	0.101	0.074	1.71E-01
rs9366793	6	32409267	G/A	*C6orf10*	INTRON	2	0.563	0.476	0.667	**2.30E-11**	0.084	0.033	**1.09E-02**	-0.011	0.123	9.29E-01	-0.117	0.033	**3.70E-04**	0.053	0.122	6.66E-01	0.192	0.033	**5.40E-09**	0.101	0.074	1.71E-01
rs9380290	6	32409430	G/C	*C6orf10*	INTRON	2	0.563	0.476	0.667	**2.30E-11**	-	-	-	-0.011	0.123	9.29E-01	-	-	-	0.053	0.122	6.66E-01	-	-	-	0.101	0.074	1.71E-01
rs9268838	6	32536693	A/G	*HLA-DRA | HLA-DRB5*	INTERGENIC	2	0.491	0.399	0.605	**2.32E-11**	-	-	-	0.063	0.124	6.13E-01	-	-	-	0.133	0.122	2.78E-01	-	-	-	0.009	0.074	9.06E-01
rs2856997	6	32889754	A/C	*HLA-DOB*	INTRON	2	0.576	0.490	0.678	**2.35E-11**	-0.006	0.033	8.50E-01	0.161	0.122	1.91E-01	-0.082	0.032	**1.11E-02**	0.092	0.122	4.53E-01	0.085	0.033	**9.10E-03**	0.014	0.074	8.49E-01
rs9268494	6	32483330	C/A	*BTNL2 | HLA-DRA*	INTERGENIC	2	0.510	0.418	0.621	**2.37E-11**	0.070	0.035	**4.40E-02**	-0.025	0.123	8.40E-01	-0.094	0.034	**6.00E-03**	0.147	0.121	2.28E-01	0.168	0.035	**1.10E-06**	0.029	0.074	7.00E-01
rs9268497	6	32483402	A/G	*BTNL2 | HLA-DRA*	INTERGENIC	2	0.510	0.418	0.621	**2.37E-11**	0.070	0.035	**4.40E-02**	-0.025	0.123	8.40E-01	-0.094	0.034	**6.00E-03**	0.147	0.121	2.28E-01	0.168	0.035	**1.10E-06**	0.029	0.074	7.00E-01
rs525607	6	32400062	A/G	*C6orf10 | LOC100131609*	INTERGENIC	2	0.564	0.476	0.667	**2.38E-11**	-	-	-	-0.011	0.123	9.29E-01	-	-	-	0.053	0.122	6.66E-01	-	-	-	0.101	0.074	1.71E-01
rs547077	6	32397296	G/A	*C6orf10*	INTRON	2	0.564	0.476	0.667	**2.38E-11**	0.084	0.033	**1.06E-02**	-0.011	0.123	9.29E-01	-0.115	0.033	**4.20E-04**	0.053	0.122	6.66E-01	0.191	0.033	**6.40E-09**	0.101	0.074	1.71E-01
rs3130685	6	31314185	A/G	*HCG27 | HLA-C*	INTERGENIC	2	1.687	1.447	1.967	**2.44E-11**	-0.009	0.032	7.90E-01	-0.097	0.123	4.30E-01	0.049	0.032	1.25E-01	-0.048	0.122	6.94E-01	-0.055	0.032	8.70E-02	0.018	0.074	8.05E-01
rs12176317	6	26480765	G/A	*BTN3A2*	INTRON	2	1.946	1.600	2.366	**2.48E-11**	0.074	0.048	1.21E-01	0.040	0.124	7.49E-01	0.176	0.047	**1.90E-04**	-0.065	0.123	6.01E-01	-0.127	0.048	**7.60E-03**	-0.073	0.074	3.26E-01
rs1265086	6	31217861	A/C	*PSORS1C1 | CCHCR1*	INTERGENIC	2	1.690	1.448	1.971	**2.48E-11**	0.008	0.032	8.10E-01	-0.154	0.123	2.14E-01	-0.022	0.032	4.90E-01	-0.146	0.122	2.37E-01	0.021	0.032	5.10E-01	-0.003	0.074	9.63E-01
rs9379858	6	26475668	G/A	*BTN3A2*	INTRON	2	1.935	1.594	2.350	**2.55E-11**	0.074	0.048	1.21E-01	0.040	0.124	7.49E-01	0.176	0.047	**1.90E-04**	-0.065	0.123	6.01E-01	-0.127	0.048	**7.60E-03**	-0.073	0.074	3.26E-01
rs9379859	6	26477528	A/G	*BTN3A2*	INTRON	2	1.935	1.594	2.350	**2.55E-11**	0.074	0.048	1.21E-01	0.040	0.124	7.49E-01	0.176	0.047	**1.90E-04**	-0.065	0.123	6.01E-01	-0.127	0.048	**7.60E-03**	-0.073	0.074	3.26E-01
rs9393705	6	26468990	A/G	*LOC100132361 | BTN3A2*	INTERGENIC	2	1.935	1.594	2.350	**2.55E-11**	0.062	0.047	1.90E-01	0.040	0.124	7.49E-01	0.165	0.047	**4.00E-04**	-0.065	0.123	6.01E-01	-0.126	0.047	**7.60E-03**	-0.073	0.074	3.26E-01
rs9393708	6	26470622	G/A	*LOC100132361 | BTN3A2*	INTERGENIC	2	1.935	1.594	2.350	**2.55E-11**	0.062	0.047	1.90E-01	0.040	0.124	7.49E-01	0.165	0.047	**4.00E-04**	-0.065	0.123	6.01E-01	-0.126	0.047	**7.60E-03**	-0.073	0.074	3.26E-01
rs1977	6	26485525	G/A	*BTN3A2*	UTR	2	1.930	1.591	2.341	**2.63E-11**	0.080	0.047	9.10E-02	0.040	0.124	7.49E-01	0.165	0.047	**4.50E-04**	-0.065	0.123	6.01E-01	-0.108	0.047	**2.20E-02**	-0.073	0.074	3.26E-01
rs9366653	6	26462226	A/G	*LOC100132361 | BTN3A2*	INTERGENIC	2	1.944	1.598	2.363	**2.68E-11**	0.056	0.047	2.30E-01	0.040	0.124	7.49E-01	0.166	0.047	**3.90E-04**	-0.065	0.123	6.01E-01	-0.131	0.047	**5.30E-03**	-0.073	0.074	3.26E-01
rs1014258	6	29418660	A/G	*OR5U1 | OR5V1*	INTERGENIC	2	1.762	1.491	2.081	**2.69E-11**	-0.025	0.037	4.90E-01	0.062	0.124	6.18E-01	0.074	0.036	**4.10E-02**	-0.073	0.123	5.58E-01	-0.110	0.037	**2.70E-03**	0.136	0.074	6.60E-02
rs17421624	6	32174155	G/A	*TNXB*	INTRON	2	0.536	0.446	0.644	**2.69E-11**	0.074	0.035	**3.30E-02**	-0.041	0.123	7.42E-01	-0.051	0.034	1.42E-01	0.100	0.122	4.14E-01	0.112	0.035	**1.26E-03**	0.022	0.074	7.62E-01
rs3868542	6	31253818	G/A	*LOC100130889 | HCG27*	INTERGENIC	2	0.576	0.490	0.678	**2.73E-11**	0.061	0.033	6.70E-02	0.136	0.123	2.75E-01	-0.128	0.033	**1.00E-04**	0.153	0.122	2.14E-01	0.186	0.033	**2.10E-08**	-0.022	0.074	7.65E-01
rs3871248	6	31253970	A/G	*LOC100130889 | HCG27*	INTERGENIC	2	0.576	0.490	0.678	**2.73E-11**	0.061	0.033	6.70E-02	0.136	0.123	2.75E-01	-0.128	0.033	**1.00E-04**	0.153	0.122	2.14E-01	0.186	0.033	**2.10E-08**	-0.022	0.074	7.65E-01
rs4947305	6	31252408	G/A	*POU5F1 | LOC100130889*	INTERGENIC	2	0.576	0.490	0.678	**2.73E-11**	0.061	0.033	6.70E-02	0.136	0.123	2.75E-01	-0.128	0.033	**1.00E-04**	0.153	0.122	2.14E-01	0.186	0.033	**2.10E-08**	-0.022	0.074	7.65E-01
rs926594	6	32413348	C/A	*C6orf10*	INTRON	2	0.565	0.478	0.669	**2.89E-11**	0.084	0.033	**1.09E-02**	-0.011	0.123	9.29E-01	-0.117	0.033	**3.70E-04**	0.053	0.122	6.66E-01	0.192	0.033	**5.40E-09**	0.101	0.074	1.71E-01
rs1474728	6	32410045	A/G	*C6orf10*	INTRON	2	0.565	0.478	0.669	**2.90E-11**	0.084	0.033	**1.09E-02**	-0.011	0.123	9.29E-01	-0.117	0.033	**3.70E-04**	0.053	0.122	6.66E-01	0.192	0.033	**5.40E-09**	0.101	0.074	1.71E-01
rs4959094	6	32421114	A/G	*C6orf10*	INTRON	2	0.565	0.478	0.669	**2.90E-11**	-	-	-	-0.011	0.123	9.29E-01	-	-	-	0.053	0.122	6.66E-01	-	-	-	0.101	0.074	1.71E-01
rs508805	6	32403328	A/G	*LOC100131609 | C6orf10*	INTERGENIC	2	0.565	0.478	0.669	**2.90E-11**	0.084	0.033	**1.09E-02**	-0.011	0.123	9.29E-01	-0.117	0.033	**3.70E-04**	0.053	0.122	6.66E-01	0.192	0.033	**5.40E-09**	0.101	0.074	1.71E-01
rs6930681	6	32412009	A/G	*C6orf10*	INTRON	2	0.565	0.478	0.669	**2.90E-11**	-	-	-	-0.011	0.123	9.29E-01	-	-	-	0.053	0.122	6.66E-01	-	-	-	0.101	0.074	1.71E-01
rs910052	6	32407851	A/C	*C6orf10*	INTRON	2	0.565	0.478	0.669	**2.90E-11**	-	-	-	-0.011	0.123	9.29E-01	-	-	-	0.053	0.122	6.66E-01	-	-	-	0.101	0.074	1.71E-01
rs9348882	6	32409300	A/G	*C6orf10*	INTRON	2	0.565	0.478	0.669	**2.90E-11**	-	-	-	-0.011	0.123	9.29E-01	-	-	-	0.053	0.122	6.66E-01	-	-	-	0.101	0.074	1.71E-01
rs9357140	6	32409492	A/G	*C6orf10*	INTRON	2	0.565	0.478	0.669	**2.90E-11**	-	-	-	-0.011	0.123	9.29E-01	-	-	-	0.053	0.122	6.66E-01	-	-	-	0.101	0.074	1.71E-01
rs9405090	6	32406350	G/A	*C6orf10*	CODING	2	0.565	0.478	0.669	**2.90E-11**	0.084	0.033	**1.09E-02**	-0.011	0.123	9.29E-01	-0.117	0.033	**3.70E-04**	0.053	0.122	6.66E-01	0.192	0.033	**5.40E-09**	0.101	0.074	1.71E-01
rs9368713	6	32405315	G/A	*LOC100131609 | C6orf10*	INTERGENIC	2	0.565	0.478	0.669	**2.91E-11**	0.070	0.052	1.80E-01	-0.011	0.123	9.29E-01	-0.216	0.052	**3.00E-05**	0.053	0.122	6.66E-01	0.299	0.052	**8.90E-09**	0.101	0.074	1.71E-01
rs504203	6	32402821	G/A	*LOC100131609 | C6orf10*	INTERGENIC	2	0.565	0.478	0.669	**2.92E-11**	-	-	-	-0.011	0.123	9.29E-01	-	-	-	0.053	0.122	6.66E-01	-	-	-	0.101	0.074	1.71E-01
rs505274	6	32402970	A/G	*LOC100131609 | C6orf10*	INTERGENIC	2	0.565	0.478	0.669	**2.92E-11**	0.084	0.033	**1.09E-02**	-0.011	0.123	9.29E-01	-0.117	0.033	**3.70E-04**	0.053	0.122	6.66E-01	0.192	0.033	**5.40E-09**	0.101	0.074	1.71E-01
rs1474729	6	32410023	C/A	*C6orf10*	INTRON	2	0.565	0.478	0.669	**2.93E-11**	-	-	-	-0.011	0.123	9.29E-01	-	-	-	0.053	0.122	6.66E-01	-	-	-	0.101	0.074	1.71E-01
rs523627	6	32399815	A/C	*C6orf10 | LOC100131609*	INTERGENIC	2	0.565	0.478	0.669	**3.02E-11**	0.086	0.033	**9.20E-03**	-0.011	0.123	9.29E-01	-0.123	0.033	**1.80E-04**	0.053	0.122	6.66E-01	0.203	0.033	**8.30E-10**	0.101	0.074	1.71E-01
rs552339	6	32400693	A/G	*C6orf10 | LOC100131609*	INTERGENIC	2	0.565	0.478	0.669	**3.02E-11**	-	-	-	-0.011	0.123	9.29E-01	-	-	-	0.053	0.122	6.66E-01	-	-	-	0.101	0.074	1.71E-01
rs6930435	6	29409201	A/G	*OR5U1 | OR5V1*	INTERGENIC	2	1.768	1.495	2.092	**3.09E-11**	-0.025	0.037	4.90E-01	0.062	0.124	6.18E-01	0.074	0.036	**4.10E-02**	-0.073	0.123	5.58E-01	-0.110	0.037	**2.70E-03**	0.136	0.074	6.60E-02
rs485774	6	32398932	G/A	*C6orf10*	INTRON	2	0.566	0.478	0.669	**3.19E-11**	0.084	0.033	**1.06E-02**	-0.011	0.123	9.29E-01	-0.115	0.033	**4.20E-04**	0.053	0.122	6.66E-01	0.191	0.033	**6.40E-09**	0.101	0.074	1.71E-01
rs9267956	6	32321616	G/A	*NOTCH4 | C6orf10*	INTERGENIC	2	0.570	0.482	0.673	**3.22E-11**	0.073	0.033	**2.70E-02**	-0.003	0.123	9.80E-01	-0.113	0.033	**5.50E-04**	0.048	0.122	6.95E-01	0.185	0.033	**2.00E-08**	0.124	0.073	9.36E-02
rs2395153	6	32453573	C/G	*C6orf10 | BTNL2*	INTERGENIC	2	0.565	0.477	0.669	**3.31E-11**	0.038	0.032	2.40E-01	0.041	0.123	7.38E-01	-0.092	0.032	**4.20E-03**	0.175	0.120	1.51E-01	0.130	0.032	**5.40E-05**	0.044	0.074	5.54E-01
rs9393710	6	26475812	G/A	*BTN3A2*	INTRON	2	1.935	1.592	2.353	**3.55E-11**	-	-	-	0.040	0.124	7.49E-01	-	-	-	-0.065	0.123	6.01E-01	-	-	-	-0.073	0.074	3.26E-01
rs9295740	6	27797481	A/G	*ZNF184 | LOC100131289*	INTERGENIC	2	1.794	1.509	2.132	**3.56E-11**	0.010	0.041	8.20E-01	0.120	0.123	3.35E-01	0.162	0.041	**6.60E-05**	0.096	0.123	4.35E-01	-0.161	0.041	**8.80E-05**	-0.042	0.075	5.71E-01
rs6457389	6	31403999	G/A	*HLA-C | HLA-B*	INTERGENIC	1	1.810	1.518	2.158	**3.62E-11**	-	-	-	-0.089	0.124	4.72E-01	-	-	-	-0.029	0.123	8.12E-01	-	-	-	-0.057	0.074	4.44E-01
rs2071351	6	33151908	G/A	*HLA-DPB1*	INTRON	2	1.851	1.542	2.222	**3.98E-11**	-0.061	0.042	1.44E-01	0.056	0.123	6.49E-01	0.032	0.041	4.30E-01	0.025	0.122	8.42E-01	-0.096	0.041	**2.10E-02**	0.000	0.074	9.97E-01
rs2621326	6	32891874	A/G	*HLA-DOB*	INTRON	2	0.584	0.498	0.685	**4.00E-11**	0.001	0.033	9.70E-01	0.176	0.121	1.52E-01	-0.077	0.032	**1.70E-02**	0.081	0.122	5.09E-01	0.087	0.033	**7.90E-03**	0.012	0.074	8.67E-01
rs9469332	6	33142854	G/A	*HLA-DPA1*	INTRON	2	1.864	1.550	2.243	**4.10E-11**	-0.053	0.042	2.00E-01	0.053	0.123	6.68E-01	0.027	0.041	5.10E-01	0.016	0.122	8.97E-01	-0.081	0.041	**4.90E-02**	-0.001	0.074	9.92E-01
rs2395309	6	33134224	G/A	*HLA-DOA | HLA-DPA1*	INTERGENIC	2	1.864	1.549	2.243	**4.17E-11**	-0.058	0.041	1.60E-01	0.053	0.123	6.68E-01	0.023	0.041	5.80E-01	0.016	0.122	8.97E-01	-0.082	0.041	**4.70E-02**	-0.001	0.074	9.92E-01
rs3131865	6	29780144	G/C	*ZFP57 | HLA-F*	INTERGENIC	2	1.718	1.463	2.017	**4.22E-11**	-	-	-	-0.144	0.122	2.41E-01	-	-	-	-0.085	0.122	4.86E-01	-	-	-	-0.094	0.074	2.01E-01
rs2269424	6	32240211	A/G	*PPT2 | EGFL8*	INTERGENIC	2	0.537	0.446	0.646	**4.60E-11**	0.096	0.040	**1.70E-02**	0.024	0.123	8.48E-01	-0.025	0.040	5.20E-01	0.155	0.121	2.05E-01	0.106	0.040	**7.80E-03**	0.045	0.074	5.45E-01
rs10214910	6	33145653	A/C	*HLA-DPA1*	INTRON	2	1.852	1.541	2.227	**5.50E-11**	-0.064	0.041	1.22E-01	0.056	0.123	6.49E-01	0.018	0.041	6.50E-01	0.025	0.122	8.42E-01	-0.083	0.041	**4.40E-02**	0.000	0.074	9.97E-01
rs9468344	6	28417548	C/A	*ZNF323 | ZKSCAN3*	INTERGENIC	2	1.703	1.453	1.997	**5.53E-11**	-0.013	0.035	7.00E-01	-0.031	0.123	8.00E-01	0.063	0.035	7.10E-02	-0.142	0.121	2.47E-01	-0.073	0.035	**3.60E-02**	0.120	0.073	1.05E-01
rs2071286	6	32287874	A/G	*NOTCH4*	INTRON	2	0.503	0.409	0.618	**5.96E-11**	0.083	0.041	**4.30E-02**	0.037	0.123	7.64E-01	-0.011	0.041	7.80E-01	0.037	0.122	7.61E-01	0.081	0.041	**4.80E-02**	0.078	0.074	2.91E-01
rs2143466	6	32417301	A/G	*C6orf10*	INTRON	2	0.575	0.487	0.679	**6.31E-11**	-	-	-	-0.032	0.123	7.98E-01	-	-	-	0.053	0.122	6.65E-01	-	-	-	0.091	0.074	2.17E-01
rs2076540	6	32425254	G/A	*C6orf10*	INTRON	2	0.576	0.488	0.679	**6.63E-11**	0.092	0.033	**5.50E-03**	-0.011	0.123	9.29E-01	-0.104	0.033	**1.46E-03**	0.053	0.122	6.66E-01	0.186	0.033	**1.90E-08**	0.108	0.074	1.44E-01
rs761187	6	32426014	G/A	*C6orf10*	INTRON	2	0.576	0.488	0.679	**6.63E-11**	0.092	0.033	**5.50E-03**	-0.011	0.123	9.29E-01	-0.104	0.033	**1.46E-03**	0.053	0.122	6.66E-01	0.186	0.033	**1.90E-08**	0.108	0.074	1.44E-01
rs3131000	6	31199252	C/G	*CDSN | PSORS1C1*	INTERGENIC	2	0.565	0.475	0.670	**6.73E-11**	0.085	0.033	**9.90E-03**	0.104	0.123	3.97E-01	-0.160	0.032	**8.60E-07**	0.011	0.122	9.31E-01	0.244	0.033	**7.30E-14**	0.098	0.074	1.86E-01
rs3130999	6	31199176	G/A	*CDSN | PSORS1C1*	INTERGENIC	2	0.565	0.476	0.670	**6.77E-11**	-	-	-	0.104	0.123	3.97E-01	-	-	-	0.011	0.122	9.31E-01	-	-	-	0.098	0.074	1.86E-01
rs3132542	6	31199142	A/G	*CDSN | PSORS1C1*	INTERGENIC	2	0.565	0.476	0.670	**6.77E-11**	-	-	-	0.104	0.123	3.97E-01	-	-	-	0.011	0.122	9.31E-01	-	-	-	0.098	0.074	1.86E-01

rs477005	6	32378478	G/A	*C6orf10*	INTRON	2	0.576	0.488	0.680	**6.79E-11**	0.091	0.033	**6.10E-03**	-0.011	0.123	9.29E-01	-0.104	0.033	**1.60E-03**	0.053	0.122	6.66E-01	0.184	0.033	**2.50E-08**	0.108	0.074	1.44E-01
rs560505	6	32369749	G/A	*C6orf10*	CODING	2	0.576	0.488	0.680	**6.81E-11**	0.092	0.033	**5.60E-03**	-0.011	0.123	9.29E-01	-0.103	0.033	**1.70E-03**	0.053	0.122	6.66E-01	0.184	0.033	**2.50E-08**	0.108	0.074	1.44E-01
rs546857	6	32397218	A/G	*C6orf10*	INTRON	2	0.576	0.488	0.680	**6.87E-11**	-	-	-	-0.011	0.123	9.29E-01	-	-	-	0.053	0.122	6.66E-01	-	-	-	0.108	0.074	1.44E-01
rs2071477	6	32933326	G/A	*PSMB9*	INTRON	1	1.770	1.491	2.101	**6.92E-11**	0.132	0.033	**4.80E-05**	-0.015	0.123	9.03E-01	0.039	0.032	2.30E-01	-0.197	0.120	1.04E-01	0.071	0.032	**2.80E-02**	-0.026	0.074	7.23E-01
rs6938130	6	32939225	T/A	*PSMB9 | HLA-DMB*	INTERGENIC	1	1.770	1.491	2.101	**6.92E-11**	0.132	0.033	**4.80E-05**	-0.015	0.123	9.03E-01	0.039	0.032	2.30E-01	-0.197	0.120	1.04E-01	0.071	0.032	**2.80E-02**	-0.026	0.074	7.23E-01
rs2301224	6	33146347	A/C	*HLA-DPA1*	INTRON	2	1.841	1.533	2.212	**7.05E-11**	-0.064	0.041	1.22E-01	0.056	0.123	6.49E-01	0.018	0.041	6.50E-01	0.025	0.122	8.42E-01	-0.083	0.041	**4.40E-02**	0.000	0.074	9.97E-01
rs660594	6	31945229	G/A	*SLC44A4*	INTRON	1	1.760	1.485	2.086	**7.44E-11**	-0.006	0.032	8.50E-01	-0.104	0.123	4.00E-01	0.212	0.031	**1.30E-11**	-0.179	0.121	1.43E-01	-0.233	0.032	**1.50E-13**	-0.092	0.074	2.17E-01
rs4713505	6	32212979	A/C	*FKBPL | PRRT1*	INTERGENIC	2	0.545	0.454	0.654	**7.96E-11**	0.079	0.036	**3.10E-02**	-0.011	0.123	9.30E-01	-0.068	0.036	5.90E-02	0.105	0.122	3.92E-01	0.138	0.036	**1.50E-04**	0.000	0.074	9.95E-01
rs1264323	6	30963886	A/G	*DDR1*	INTRON	2	1.661	1.425	1.935	**8.42E-11**	0.001	0.033	9.90E-01	0.057	0.123	6.47E-01	0.008	0.032	8.00E-01	-0.137	0.121	2.62E-01	-0.006	0.032	8.50E-01	-0.003	0.074	9.73E-01
rs522308	6	32689900	A/G	*HLA-DRB1 | HLA-DQA1*	INTERGENIC	1	0.490	0.395	0.608	**8.51E-11**	0.090	0.034	**7.50E-03**	0.003	0.123	9.80E-01	-0.105	0.033	**1.70E-03**	0.139	0.121	2.56E-01	0.192	0.034	**1.20E-08**	-0.019	0.074	7.93E-01
rs2228628	6	32196832	G/C	*CREBL1*	CODING	2	0.545	0.454	0.655	**8.61E-11**	-	-	-	-0.004	0.123	9.74E-01	-	-	-	0.113	0.122	3.58E-01	-	-	-	0.002	0.074	9.74E-01
rs2156875	6	31425326	G/A	*HLA-C | HLA-B*	INTERGENIC	2	0.589	0.502	0.691	**8.65E-11**	-0.009	0.032	7.80E-01	0.220	0.121	7.23E-02	0.040	0.032	2.10E-01	0.124	0.122	3.15E-01	-0.065	0.032	**4.10E-02**	0.120	0.074	1.05E-01
rs2844458	6	31959448	A/C	*EHMT2*	INTRON	2	0.556	0.466	0.664	**8.67E-11**	0.058	0.035	9.40E-02	0.002	0.123	9.87E-01	-0.079	0.034	**2.10E-02**	0.069	0.122	5.73E-01	0.133	0.035	**1.20E-04**	0.047	0.074	5.28E-01
rs1978029	6	32839688	G/A	*HLA-DQB2 | HLA-DOB*	INTERGENIC	2	0.597	0.511	0.698	**8.70E-11**	0.064	0.032	5.00E-02	0.293	0.118	**1.56E-02**	-0.090	0.032	**5.20E-03**	0.245	0.119	**4.37E-02**	0.157	0.032	**1.10E-06**	-0.067	0.074	3.67E-01
rs2076542	6	32425105	A/G	*C6orf10*	INTRON	2	0.572	0.484	0.678	**8.77E-11**	0.085	0.033	**1.07E-02**	-0.011	0.123	9.29E-01	-0.125	0.033	**1.50E-04**	0.053	0.122	6.66E-01	0.204	0.033	**7.50E-10**	0.101	0.074	1.71E-01
rs9268284	6	32423194	A/G	*C6orf10*	INTRON	2	0.573	0.485	0.678	**8.91E-11**	-	-	-	-0.011	0.123	9.29E-01	-	-	-	0.053	0.122	6.66E-01	-	-	-	0.101	0.074	1.71E-01
rs2022534	6	32415115	G/A	*C6orf10*	INTRON	2	0.573	0.485	0.678	**8.96E-11**	0.084	0.033	**1.09E-02**	-0.011	0.123	9.29E-01	-0.117	0.033	**3.70E-04**	0.053	0.122	6.66E-01	0.192	0.033	**5.40E-09**	0.101	0.074	1.71E-01
rs2073046	6	32444052	A/G	*C6orf10*	INTRON	2	0.573	0.485	0.678	**8.96E-11**	0.082	0.033	**1.29E-02**	-0.011	0.123	9.29E-01	-0.119	0.033	**2.90E-04**	0.053	0.122	6.66E-01	0.193	0.033	**4.90E-09**	0.101	0.074	1.71E-01
rs2076538	6	32425449	C/A	*C6orf10*	INTRON	2	0.573	0.485	0.678	**8.96E-11**	0.086	0.033	**9.60E-03**	-0.011	0.123	9.29E-01	-0.116	0.033	**4.00E-04**	0.053	0.122	6.66E-01	0.193	0.033	**4.80E-09**	0.100	0.074	1.77E-01
rs2076541	6	32425193	G/A	*C6orf10*	INTRON	2	0.573	0.485	0.678	**8.96E-11**	0.086	0.033	**9.60E-03**	-0.011	0.123	9.29E-01	-0.116	0.033	**4.00E-04**	0.053	0.122	6.66E-01	0.193	0.033	**4.80E-09**	0.101	0.074	1.71E-01
rs2143465	6	32417330	G/A	*C6orf10*	INTRON	2	0.573	0.485	0.678	**8.96E-11**	0.084	0.033	**1.09E-02**	-0.011	0.123	9.29E-01	-0.117	0.033	**3.70E-04**	0.053	0.122	6.66E-01	0.192	0.033	**5.40E-09**	0.101	0.074	1.71E-01
rs4959025	6	32421121	C/A	*C6orf10*	INTRON	2	0.573	0.485	0.678	**8.96E-11**	-	-	-	-0.011	0.123	9.29E-01	-	-	-	0.053	0.122	6.66E-01	-	-	-	0.101	0.074	1.71E-01
rs4959026	6	32421439	A/C	*C6orf10*	INTRON	2	0.573	0.485	0.678	**8.96E-11**	-	-	-	-0.011	0.123	9.29E-01	-	-	-	0.053	0.122	6.66E-01	-	-	-	0.101	0.074	1.71E-01
rs4959096	6	32422654	A/G	*C6orf10*	INTRON	2	0.573	0.485	0.678	**8.96E-11**	-	-	-	-0.011	0.123	9.29E-01	-	-	-	0.053	0.122	6.66E-01	-	-	-	0.101	0.074	1.71E-01
rs9268260	6	32418945	A/G	*C6orf10*	INTRON	2	0.573	0.485	0.678	**8.96E-11**	-	-	-	-0.011	0.123	9.29E-01	-	-	-	0.053	0.122	6.66E-01	-	-	-	0.101	0.074	1.71E-01
rs9268283	6	32423151	G/A	*C6orf10*	INTRON	2	0.573	0.485	0.678	**8.96E-11**	0.083	0.033	**1.21E-02**	-0.011	0.123	9.29E-01	-0.125	0.033	**1.40E-04**	0.053	0.122	6.66E-01	0.203	0.033	**8.50E-10**	0.101	0.074	1.71E-01
rs9268326	6	32438131	G/A	*C6orf10*	INTRON	2	0.573	0.485	0.678	**8.96E-11**	-	-	-	-0.011	0.123	9.29E-01	-	-	-	0.053	0.122	6.66E-01	-	-	-	0.101	0.074	1.71E-01
rs9268368	6	32441933	G/A	*C6orf10*	CODING	2	0.573	0.485	0.678	**8.96E-11**	0.082	0.033	**1.29E-02**	-0.011	0.123	9.29E-01	-0.119	0.033	**2.90E-04**	0.053	0.122	6.66E-01	0.193	0.033	**4.90E-09**	0.101	0.074	1.71E-01
rs9268285	6	32423535	G/A	*C6orf10*	INTRON	2	0.573	0.485	0.678	**9.02E-11**	-	-	-	-0.011	0.123	9.29E-01	-	-	-	0.053	0.122	6.66E-01	-	-	-	0.101	0.074	1.71E-01
rs9268384	6	32444564	G/A	*C6orf10*	CODING	2	0.573	0.485	0.678	**9.02E-11**	0.082	0.033	**1.29E-02**	-0.011	0.123	9.29E-01	-0.119	0.033	**2.90E-04**	0.053	0.122	6.66E-01	0.193	0.033	**4.90E-09**	0.101	0.074	1.71E-01
rs887466	6	31251490	A/G	*POU5F1 | LOC100130889*	INTERGENIC	2	0.590	0.503	0.692	**9.03E-11**	0.048	0.033	1.45E-01	0.123	0.123	3.22E-01	-0.147	0.032	**5.70E-06**	0.268	0.119	**2.68E-02**	0.198	0.033	**1.30E-09**	-0.036	0.074	6.30E-01
rs1265155	6	31251673	A/G	*POU5F1 | LOC100130889*	INTERGENIC	2	0.585	0.497	0.688	**9.04E-11**	0.061	0.033	6.70E-02	0.136	0.123	2.75E-01	-0.128	0.033	**1.00E-04**	0.153	0.122	2.14E-01	0.186	0.033	**2.10E-08**	-0.022	0.074	7.65E-01
rs547261	6	32390011	A/G	*C6orf10*	INTRON	2	0.573	0.485	0.678	**9.09E-11**	0.084	0.033	**1.06E-02**	-0.011	0.123	9.29E-01	-0.115	0.033	**4.20E-04**	0.053	0.122	6.66E-01	0.191	0.033	**6.40E-09**	0.101	0.074	1.71E-01
rs531094	6	32394080	A/T	*C6orf10*	INTRON	2	0.573	0.485	0.679	**9.15E-11**	0.084	0.033	**1.06E-02**	-0.011	0.123	9.29E-01	-0.115	0.033	**4.20E-04**	0.053	0.122	6.66E-01	0.191	0.033	**6.40E-09**	0.101	0.074	1.71E-01
rs660895	6	32685358	G/A	*HLA-DRB1 | HLA-DQA1*	INTERGENIC	2	0.494	0.399	0.611	**9.17E-11**	0.061	0.039	1.17E-01	0.080	0.123	5.17E-01	-0.034	0.039	3.70E-01	0.009	0.123	9.41E-01	0.088	0.039	**2.40E-02**	-0.005	0.074	9.43E-01
rs482194	6	32368537	G/A	*C6orf10*	UTR	2	0.574	0.485	0.679	**9.22E-11**	0.085	0.033	**9.80E-03**	-0.011	0.123	9.29E-01	-0.114	0.033	**4.70E-04**	0.053	0.122	6.66E-01	0.191	0.033	**6.50E-09**	0.101	0.074	1.71E-01
rs537757	6	32376479	A/T	*C6orf10*	INTRON	2	0.574	0.485	0.679	**9.25E-11**	0.085	0.033	**9.80E-03**	-0.011	0.123	9.29E-01	-0.114	0.033	**4.70E-04**	0.053	0.122	6.66E-01	0.191	0.033	**6.50E-09**	0.101	0.074	1.71E-01
rs502626	6	32386244	G/A	*C6orf10*	INTRON	2	0.574	0.485	0.679	**9.27E-11**	0.084	0.033	**1.06E-02**	-0.011	0.123	9.29E-01	-0.115	0.033	**4.20E-04**	0.053	0.122	6.66E-01	0.191	0.033	**6.40E-09**	0.101	0.074	1.71E-01
rs9268132	6	32362632	G/A	*NOTCH4 | C6orf10*	INTERGENIC	2	0.574	0.485	0.679	**9.36E-11**	0.085	0.033	**9.80E-03**	-0.011	0.123	9.29E-01	-0.114	0.033	**4.70E-04**	0.053	0.122	6.66E-01	0.191	0.033	**6.50E-09**	0.101	0.074	1.71E-01
rs4713518	6	32365315	G/A	*NOTCH4 | C6orf10*	INTERGENIC	2	0.574	0.485	0.679	**9.40E-11**	0.085	0.033	**9.80E-03**	-0.011	0.123	9.29E-01	-0.114	0.033	**4.70E-04**	0.053	0.122	6.66E-01	0.191	0.033	**6.50E-09**	0.101	0.074	1.71E-01
rs8111	6	32191153	A/G	*CREBL1*	UTR	2	0.545	0.454	0.655	**9.47E-11**	0.079	0.036	**3.10E-02**	0.003	0.123	9.79E-01	-0.068	0.036	5.90E-02	0.126	0.121	3.05E-01	0.138	0.036	**1.50E-04**	0.001	0.074	9.94E-01
rs2923006	6	31425078	C/A	*HLA-C | HLA-B*	INTERGENIC	2	0.589	0.501	0.691	**9.50E-11**	-0.009	0.032	7.80E-01	0.220	0.121	7.23E-02	0.040	0.032	2.10E-01	0.124	0.122	3.15E-01	-0.065	0.032	**4.10E-02**	0.120	0.074	1.05E-01
rs1800624	6	32260365	A/T	*AGER | PBX2*	INTERGENIC	2	0.523	0.429	0.636	**1.01E-10**	0.101	0.040	**1.13E-02**	-0.011	0.123	9.29E-01	-0.015	0.039	7.10E-01	0.142	0.121	2.46E-01	0.098	0.040	**1.30E-02**	0.049	0.074	5.13E-01
rs9267817	6	32247791	A/G	*AGPAT1*	INTRON	2	0.528	0.435	0.641	**1.04E-10**	-	-	-	0.024	0.123	8.48E-01	-	-	-	0.155	0.121	2.05E-01	-	-	-	0.045	0.074	5.45E-01
rs241436	6	32905854	G/A	*TAP2*	INTRON	2	1.643	1.413	1.910	**1.09E-10**	0.026	0.033	4.20E-01	0.064	0.123	6.08E-01	0.004	0.032	8.90E-01	-0.107	0.122	3.81E-01	0.022	0.032	5.00E-01	0.027	0.074	7.12E-01
rs1431400	6	33149154	A/G	*HLA-DPA1*	INTRON	2	1.820	1.517	2.184	**1.12E-10**	-0.064	0.041	1.24E-01	0.056	0.123	6.49E-01	0.025	0.041	5.50E-01	0.025	0.122	8.42E-01	-0.090	0.041	**2.90E-02**	0.000	0.074	9.97E-01
rs6914849	6	33148693	A/G	*HLA-DPA1*	INTRON	2	1.820	1.517	2.184	**1.12E-10**	-0.064	0.041	1.23E-01	0.056	0.123	6.49E-01	0.021	0.041	6.10E-01	0.025	0.122	8.42E-01	-0.085	0.041	**3.90E-02**	0.000	0.074	9.97E-01
rs1431399	6	33149012	G/A	*HLA-DPA1*	INTRON	2	1.828	1.521	2.196	**1.20E-10**	-0.064	0.041	1.24E-01	0.056	0.123	6.49E-01	0.025	0.041	5.50E-01	0.025	0.122	8.42E-01	-0.090	0.041	**2.90E-02**	0.000	0.074	9.97E-01
rs1431401	6	33149164	A/G	*HLA-DPA1*	INTRON	2	1.828	1.521	2.196	**1.21E-10**	-0.066	0.041	1.11E-01	0.056	0.123	6.49E-01	0.024	0.041	5.60E-01	0.025	0.122	8.42E-01	-0.091	0.041	**2.80E-02**	0.000	0.074	9.97E-01
rs1233495	6	29552100	A/G	*OR2H1 | MAS1L*	INTERGENIC	2	1.688	1.439	1.979	**1.25E-10**	0.021	0.036	5.50E-01	0.047	0.123	7.02E-01	0.111	0.035	**1.70E-03**	-0.082	0.122	5.02E-01	-0.102	0.036	**4.10E-03**	-0.055	0.074	4.56E-01
rs241414	6	32965779	C/A	*PSMB9 | HLA-DMB*	INTERGENIC	2	1.640	1.410	1.906	**1.25E-10**	0.036	0.033	2.70E-01	0.087	0.123	4.79E-01	0.007	0.032	8.30E-01	-0.120	0.121	3.25E-01	0.016	0.032	6.10E-01	0.012	0.074	8.71E-01
rs13190937	6	28519223	A/G	*ZSCAN23*	UTR	2	1.660	1.422	1.937	**1.26E-10**	-0.049	0.033	1.39E-01	-0.020	0.123	8.70E-01	0.133	0.033	**5.50E-05**	-0.120	0.122	3.26E-01	-0.188	0.033	**1.80E-08**	-0.006	0.074	9.31E-01
rs1736951	6	29893755	A/G	*HCG4 | HLA-G*	INTERGENIC	2	1.654	1.419	1.929	**1.30E-10**	-	-	-	-0.122	0.123	3.24E-01	-	-	-	-0.109	0.122	3.76E-01	-	-	-	0.018	0.074	8.08E-01
rs3131866	6	29779719	A/G	*ZFP57 | HLA-F*	INTERGENIC	2	1.696	1.444	1.992	**1.30E-10**	-	-	-	-0.103	0.123	4.03E-01	-	-	-	-0.079	0.122	5.19E-01	-	-	-	-0.074	0.074	3.19E-01
rs2107203	6	29777809	A/G	*ZFP57 | HLA-F*	INTERGENIC	2	1.696	1.443	1.992	**1.31E-10**	0.020	0.036	5.70E-01	-0.103	0.123	4.03E-01	0.101	0.035	**4.10E-03**	-0.079	0.122	5.19E-01	-0.089	0.035	**1.21E-02**	-0.074	0.074	3.19E-01
rs201004	6	27912913	G/A	*HIST1H4K | HIST1H2AK*	INTERGENIC	2	1.767	1.485	2.102	**1.32E-10**	0.003	0.040	9.40E-01	0.106	0.124	3.92E-01	0.148	0.040	**2.20E-04**	0.110	0.123	3.74E-01	-0.157	0.040	**1.00E-04**	-0.055	0.075	4.59E-01
rs3116802	6	29775869	G/A	*ZFP57 | HLA-F*	INTERGENIC	2	1.696	1.443	1.992	**1.32E-10**	0.019	0.037	6.00E-01	-0.103	0.123	4.03E-01	0.138	0.036	**1.30E-04**	-0.079	0.122	5.19E-01	-0.133	0.036	**2.50E-04**	-0.074	0.074	3.19E-01
rs3129070	6	29776055	A/G	*ZFP57 | HLA-F*	INTERGENIC	2	1.696	1.443	1.992	**1.32E-10**	0.020	0.036	5.70E-01	-0.103	0.123	4.03E-01	0.101	0.035	**4.10E-03**	-0.079	0.122	5.19E-01	-0.089	0.035	**1.21E-02**	-0.074	0.074	3.19E-01
rs7756567	6	26589621	C/A	*BTN2A1 | BTN1A1*	INTERGENIC	2	1.792	1.500	2.141	**1.36E-10**	0.026	0.040	5.20E-01	0.038	0.123	7.57E-01	0.097	0.040	**1.39E-02**	-0.047	0.122	7.01E-01	-0.081	0.040	**4.30E-02**	0.007	0.074	9.28E-01
rs9358946	6	26586906	A/G	*BTN2A1 | BTN1A1*	INTERGENIC	2	1.792	1.500	2.141	**1.37E-10**	0.026	0.040	5.20E-01	0.038	0.123	7.57E-01	0.097	0.040	**1.39E-02**	-0.047	0.122	7.01E-01	-0.081	0.040	**4.30E-02**	0.007	0.074	9.28E-01
rs1233487	6	29576788	G/A	*LOC729653*	INTRON	2	1.680	1.434	1.968	**1.38E-10**	0.022	0.035	5.30E-01	-0.021	0.123	8.64E-01	0.111	0.035	**1.60E-03**	-0.154	0.121	2.07E-01	-0.102	0.035	**4.00E-03**	-0.050	0.074	5.01E-01
rs7773938	6	26582023	A/G	*BTN2A1 | BTN1A1*	INTERGENIC	2	1.792	1.499	2.141	**1.38E-10**	0.025	0.040	5.40E-01	0.038	0.123	7.57E-01	0.098	0.040	**1.29E-02**	-0.047	0.122	7.01E-01	-0.082	0.040	**3.90E-02**	0.007	0.074	9.28E-01
rs9358944	6	26577854	C/A	*BTN2A1 | BTN1A1*	INTERGENIC	2	1.792	1.499	2.141	**1.38E-10**	0.025	0.040	5.40E-01	0.038	0.123	7.57E-01	0.098	0.040	**1.29E-02**	-0.047	0.122	7.01E-01	-0.082	0.040	**3.90E-02**	0.007	0.074	9.28E-01
rs9358945	6	26580093	G/A	*BTN2A1 | BTN1A1*	INTERGENIC	2	1.792	1.499	2.141	**1.38E-10**	0.025	0.040	5.40E-01	0.038	0.123	7.57E-01	0.098	0.040	**1.29E-02**	-0.047	0.122	7.01E-01	-0.082	0.040	**3.90E-02**	0.007	0.074	9.28E-01
rs9366658	6	26577845	A/G	*BTN2A1 | BTN1A1*	INTERGENIC	2	1.792	1.499	2.141	**1.38E-10**	0.025	0.040	5.40E-01	0.038	0.123	7.57E-01	0.098	0.040	**1.30E-02**	-0.047	0.122	7.01E-01	-0.082	0.040	**3.90E-02**	0.007	0.074	9.28E-01
rs2093169	6	26603078	A/G	*BTN2A1 | BTN1A1*	INTERGENIC	2	1.791	1.499	2.140	**1.43E-10**	0.023	0.041	5.60E-01	0.038	0.123	7.57E-01	0.119	0.040	**3.20E-03**	-0.047	0.122	7.01E-01	-0.103	0.040	**1.06E-02**	0.007	0.074	9.28E-01
rs6940698	6	25929559	A/G	*SLC17A1*	INTRON	2	2.030	1.635	2.521	**1.43E-10**	0.043	0.053	4.30E-01	0.020	0.124	8.72E-01	0.143	0.053	**6.70E-03**	-0.113	0.122	3.62E-01	-0.122	0.053	**2.20E-02**	-0.087	0.074	2.45E-01
rs9267803	6	32209740	A/G	*FKBPL | PRRT1*	INTERGENIC	2	0.554	0.462	0.663	**1.44E-10**	0.079	0.036	**3.10E-02**	-0.004	0.123	9.74E-01	-0.068	0.036	5.90E-02	0.113	0.122	3.58E-01	0.138	0.036	**1.50E-04**	0.002	0.074	9.74E-01
rs3132554	6	31192142	G/A	*CDSN*	CODING	1	0.560	0.469	0.669	**1.45E-10**	0.054	0.031	8.50E-02	0.046	0.124	7.15E-01	-0.169	0.031	**4.70E-08**	0.137	0.123	2.70E-01	0.226	0.031	**3.70E-13**	0.022	0.075	7.69E-01

rs429150	6	32183541	G/A	*TNXB*	INTRON	1	0.560	0.469	0.669	**1.45E-10**	0.084	0.032	**8.10E-03**	-0.067	0.123	5.88E-01	-0.183	0.031	**5.20E-09**	0.131	0.121	2.85E-01	0.274	0.032	**3.10E-18**	-0.001	0.074	9.85E-01
rs2071354	6	33152366	G/A	*HLA-DPB1*	INTRON	2	1.920	1.573	2.344	**1.49E-10**	-0.036	0.046	4.30E-01	0.080	0.123	5.18E-01	0.038	0.045	4.00E-01	0.110	0.122	3.70E-01	-0.074	0.045	1.02E-01	-0.030	0.074	6.82E-01
rs987870	6	33150858	G/A	*HLA-DPA1 | HLA-DPB1*	INTERGENIC	2	1.920	1.572	2.344	**1.51E-10**	-0.036	0.046	4.30E-01	0.080	0.123	5.18E-01	0.038	0.045	4.00E-01	0.110	0.122	3.70E-01	-0.074	0.045	1.02E-01	-0.030	0.074	6.82E-01
rs1573649	6	32839236	A/G	*HLA-DQB2*	UTR	2	0.604	0.518	0.705	**1.52E-10**	0.064	0.032	5.00E-02	0.293	0.118	**1.56E-02**	-0.090	0.032	**5.20E-03**	0.245	0.119	**4.37E-02**	0.157	0.032	**1.10E-06**	-0.067	0.074	3.69E-01
rs6902687	6	28521470	G/A	*ZSCAN23 | COX11P*	INTERGENIC	2	1.666	1.425	1.948	**1.52E-10**	-0.049	0.033	1.39E-01	-0.044	0.124	7.24E-01	0.133	0.033	**5.50E-05**	-0.139	0.122	2.61E-01	-0.188	0.033	**1.80E-08**	-0.008	0.075	9.13E-01
rs2301220	6	33146744	A/G	*HLA-DPA1*	INTRON	2	1.820	1.515	2.186	**1.62E-10**	-0.064	0.041	1.22E-01	0.056	0.123	6.49E-01	0.018	0.041	6.50E-01	0.025	0.122	8.42E-01	-0.083	0.041	**4.40E-02**	0.000	0.074	9.97E-01
rs9348904	6	33148813	G/A	*HLA-DPA1*	INTRON	2	1.820	1.515	2.186	**1.62E-10**	-0.064	0.041	1.26E-01	0.056	0.123	6.49E-01	0.020	0.041	6.30E-01	0.025	0.122	8.42E-01	-0.084	0.041	**4.20E-02**	0.000	0.074	9.97E-01
rs3094548	6	29463181	G/C	*OR12D3 | OR12D2*	INTERGENIC	2	1.659	1.420	1.938	**1.85E-10**	0.033	0.034	3.30E-01	-0.080	0.126	5.29E-01	0.041	0.034	2.20E-01	-0.017	0.126	8.94E-01	-0.013	0.034	7.00E-01	-0.103	0.074	1.65E-01
rs4712969	6	25872171	A/G	*SLC17A4*	INTRON	2	2.008	1.620	2.488	**1.94E-10**	0.043	0.053	4.20E-01	0.005	0.123	9.68E-01	0.146	0.053	**5.80E-03**	-0.122	0.121	3.19E-01	-0.124	0.053	**1.90E-02**	-0.096	0.074	1.92E-01
rs4713506	6	32221958	A/G	*FKBPL | PRRT1*	INTERGENIC	2	0.561	0.469	0.670	**1.99E-10**	0.081	0.036	**2.50E-02**	0.010	0.123	9.38E-01	-0.067	0.036	6.20E-02	0.129	0.121	2.93E-01	0.139	0.036	**1.20E-04**	0.005	0.074	9.50E-01
rs241413	6	32966978	G/A	*PSMB9 | HLA-DMB*	INTERGENIC	2	1.630	1.402	1.895	**2.04E-10**	0.036	0.033	2.70E-01	0.087	0.123	4.79E-01	0.007	0.032	8.30E-01	-0.120	0.121	3.25E-01	0.016	0.032	6.10E-01	0.012	0.074	8.67E-01
rs6904071	6	27155235	A/G	*LOC100133205 | HIST1H2BJ*	INTERGENIC	2	1.774	1.487	2.118	**2.11E-10**	-0.002	0.041	9.70E-01	0.079	0.123	5.24E-01	0.121	0.040	**2.70E-03**	-0.067	0.122	5.84E-01	-0.122	0.041	**2.70E-03**	0.059	0.074	4.30E-01
rs3095313	6	31198578	A/G	*CDSN | PSORS1C1*	INTERGENIC	2	0.559	0.467	0.669	**2.18E-10**	0.089	0.034	**8.30E-03**	0.127	0.123	3.04E-01	-0.164	0.034	**1.00E-06**	-0.092	0.123	4.56E-01	0.251	0.034	**1.00E-13**	0.127	0.074	8.70E-02
rs241398	6	32983428	A/G	*PSMB9 | HLA-DMB*	INTERGENIC	2	1.633	1.403	1.900	**2.21E-10**	0.037	0.033	2.50E-01	0.087	0.123	4.79E-01	0.007	0.032	8.20E-01	-0.120	0.121	3.25E-01	0.017	0.032	6.00E-01	0.012	0.074	8.67E-01
rs241402	6	32977023	G/A	*PSMB9 | HLA-DMB*	INTERGENIC	2	1.633	1.403	1.899	**2.24E-10**	0.035	0.033	2.80E-01	0.087	0.123	4.79E-01	0.007	0.032	8.40E-01	-0.120	0.121	3.25E-01	0.016	0.032	6.20E-01	0.012	0.074	8.67E-01
rs13194053	6	27251862	G/A	*HIST1H2AH | PRSS16*	INTERGENIC	2	1.782	1.491	2.130	**2.25E-10**	0.007	0.041	8.60E-01	0.079	0.123	5.24E-01	0.125	0.040	**2.00E-03**	-0.067	0.122	5.84E-01	-0.117	0.041	**4.10E-03**	0.059	0.074	4.30E-01
rs9276586	6	32840915	A/G	*HLA-DQB2 | HLA-DOB*	INTERGENIC	2	0.594	0.506	0.698	**2.27E-10**	0.064	0.032	5.00E-02	0.293	0.118	**1.56E-02**	-0.090	0.032	**5.20E-03**	0.245	0.119	**4.37E-02**	0.157	0.032	**1.10E-06**	-0.067	0.074	3.67E-01
rs241404	6	32973975	G/A	*PSMB9 | HLA-DMB*	INTERGENIC	2	1.632	1.403	1.899	**2.30E-10**	0.035	0.033	2.80E-01	0.087	0.123	4.79E-01	0.007	0.032	8.40E-01	-0.120	0.121	3.25E-01	0.016	0.032	6.20E-01	0.012	0.074	8.67E-01
rs28752872	6	31410729	G/A	*HLA-C | HLA-B*	INTERGENIC	2	0.592	0.504	0.697	**2.37E-10**	-	-	-	0.024	0.124	8.46E-01	-	-	-	0.153	0.122	2.13E-01	-	-	-	0.029	0.075	6.99E-01
rs13191296	6	25792585	A/G	*SCGN*	INTRON	2	2.071	1.654	2.595	**2.38E-10**	0.050	0.055	3.60E-01	0.059	0.123	6.32E-01	0.139	0.054	**1.02E-02**	-0.118	0.121	3.35E-01	-0.112	0.054	**4.00E-02**	-0.067	0.074	3.69E-01
rs2261033	6	31711570	G/A	*BAT2*	INTRON	2	0.588	0.499	0.693	**2.40E-10**	0.020	0.033	5.50E-01	0.022	0.124	8.59E-01	-0.072	0.032	**2.60E-02**	-0.077	0.123	5.36E-01	0.085	0.033	**8.90E-03**	-0.037	0.075	6.21E-01
rs3130906	6	31533160	A/G	*LOC100129668 | HCP5*	INTERGENIC	1	1.730	1.460	2.050	**2.44E-10**	-	-	-	0.051	0.123	6.82E-01	-	-	-	-0.178	0.120	1.44E-01	-	-	-	0.030	0.074	6.82E-01
rs2735028	6	29893517	A/G	*HCG4 | HLA-G*	INTERGENIC	2	1.655	1.416	1.935	**2.51E-10**	-0.005	0.033	8.80E-01	-0.122	0.123	3.24E-01	0.064	0.033	5.10E-02	-0.109	0.122	3.76E-01	-0.064	0.033	5.30E-02	0.018	0.074	8.08E-01
rs9267948	6	32320211	G/A	*NOTCH4 | C6orf10*	INTERGENIC	2	0.589	0.500	0.694	**2.56E-10**	-0.067	0.032	**3.70E-02**	-0.037	0.123	7.63E-01	0.042	0.032	1.80E-01	-0.135	0.121	2.70E-01	-0.106	0.032	**1.02E-03**	-0.020	0.074	7.87E-01
rs1628578	6	29803481	C/A	*HLA-F | FLJ35429*	INTERGENIC	2	1.687	1.435	1.985	**2.67E-10**	0.040	0.036	2.70E-01	-0.099	0.122	4.23E-01	0.131	0.036	**2.50E-04**	-0.071	0.122	5.60E-01	-0.108	0.036	**2.70E-03**	-0.036	0.074	6.26E-01
rs1736922	6	29801948	A/G	*HLA-F*	INTRON	2	1.687	1.434	1.985	**2.68E-10**	0.054	0.035	1.23E-01	-0.099	0.122	4.23E-01	0.084	0.035	**1.50E-02**	-0.071	0.122	5.60E-01	-0.046	0.035	1.80E-01	-0.036	0.074	6.26E-01
rs2073044	6	32446964	A/G	*C6orf10*	INTRON	2	0.520	0.425	0.637	**2.68E-10**	0.050	0.037	1.80E-01	-0.009	0.124	9.45E-01	-0.085	0.037	**2.20E-02**	-0.018	0.123	8.82E-01	0.133	0.037	**3.40E-04**	0.042	0.074	5.67E-01
rs3094207	6	31199388	G/C	*CDSN | PSORS1C1*	INTERGENIC	2	0.561	0.469	0.672	**2.68E-10**	0.089	0.034	**8.30E-03**	0.114	0.122	3.56E-01	-0.164	0.034	**1.00E-06**	-0.100	0.122	4.12E-01	0.251	0.034	**1.00E-13**	0.123	0.073	9.54E-02
rs3130990	6	31195265	A/G	*CDSN*	INTRON	2	0.561	0.469	0.672	**2.68E-10**	-	-	-	0.118	0.123	3.41E-01	-	-	-	-0.107	0.122	3.86E-01	-	-	-	0.123	0.074	9.50E-02
rs3130991	6	31195333	A/G	*CDSN*	INTRON	2	0.561	0.469	0.672	**2.68E-10**	0.089	0.034	**9.00E-03**	0.114	0.122	3.56E-01	-0.165	0.034	**8.40E-07**	-0.100	0.122	4.12E-01	0.252	0.034	**8.50E-14**	0.123	0.073	9.54E-02
rs3130995	6	31198531	A/G	*CDSN | PSORS1C1*	INTERGENIC	2	0.561	0.469	0.672	**2.68E-10**	-	-	-	0.114	0.122	3.56E-01	-	-	-	-0.100	0.122	4.12E-01	-	-	-	0.123	0.073	9.54E-02
rs200994	6	27921792	A/C	*HIST1H2BN | HIST1H2AL*	INTERGENIC	2	1.697	1.440	1.999	**2.70E-10**	0.049	0.038	2.00E-01	0.097	0.123	4.33E-01	0.124	0.037	**8.90E-04**	0.046	0.123	7.13E-01	-0.083	0.038	**2.80E-02**	-0.042	0.074	5.74E-01
rs2516436	6	31527856	G/C	*LOC100129668 | HCP5*	INTERGENIC	2	1.631	1.401	1.899	**2.72E-10**	-	-	-	-0.112	0.123	3.63E-01	-	-	-	-0.111	0.122	3.65E-01	-	-	-	0.025	0.074	7.36E-01
rs3020644	6	32002605	G/A	*ZBTB12 | C2*	INTERGENIC	2	0.582	0.492	0.689	**2.75E-10**	0.039	0.033	2.30E-01	0.123	0.122	3.17E-01	-0.129	0.033	**8.00E-05**	0.051	0.122	6.76E-01	0.167	0.033	**3.90E-07**	0.036	0.074	6.27E-01
rs1559876	6	32323747	C/G	*NOTCH4 | C6orf10*	INTERGENIC	2	0.571	0.480	0.680	**2.82E-10**	0.078	0.033	**1.90E-02**	0.007	0.124	9.55E-01	-0.113	0.033	**5.80E-04**	0.050	0.123	6.87E-01	0.187	0.033	**1.50E-08**	0.128	0.073	8.39E-02
rs1035798	6	32259200	A/G	*AGER*	INTRON	2	0.531	0.436	0.647	**2.91E-10**	0.097	0.040	**1.47E-02**	-0.011	0.123	9.29E-01	-0.020	0.040	6.10E-01	0.142	0.121	2.46E-01	0.102	0.040	**1.02E-02**	0.049	0.074	5.06E-01
rs7744001	6	32734064	A/G	*HLA-DQA1 | HLA-DQB1*	INTERGENIC	2	0.522	0.427	0.639	**2.91E-10**	0.077	0.034	**2.40E-02**	-0.041	0.123	7.37E-01	-0.059	0.034	7.90E-02	0.243	0.119	**4.47E-02**	0.140	0.034	**3.80E-05**	-0.046	0.074	5.34E-01
rs454748	6	32321188	A/G	*NOTCH4 | C6orf10*	INTERGENIC	2	0.606	0.519	0.708	**3.05E-10**	0.049	0.032	1.29E-01	0.013	0.124	9.13E-01	-0.150	0.032	**2.80E-06**	0.078	0.122	5.26E-01	0.206	0.032	**1.90E-10**	0.070	0.074	3.43E-01
rs6941112	6	32054593	A/G	*STK19*	INTRON	2	0.559	0.466	0.670	**3.05E-10**	0.033	0.035	3.60E-01	-0.113	0.122	3.59E-01	-0.098	0.035	**5.10E-03**	0.016	0.122	8.94E-01	0.129	0.035	**2.50E-04**	0.013	0.074	8.62E-01
rs1892250	6	25877003	A/G	*SLC17A4*	INTRON	2	2.005	1.614	2.490	**3.20E-10**	0.043	0.053	4.20E-01	0.005	0.123	9.68E-01	0.146	0.053	**5.80E-03**	-0.122	0.121	3.19E-01	-0.124	0.053	**1.90E-02**	-0.096	0.074	1.92E-01
rs3131001	6	31199510	A/C	*CDSN | PSORS1C1*	INTERGENIC	2	0.563	0.471	0.673	**3.23E-10**	-	-	-	0.114	0.122	3.56E-01	-	-	-	-0.100	0.122	4.12E-01	-	-	-	0.123	0.073	9.54E-02
rs1610742	6	29785931	G/A	*ZFP57 | HLA-F*	INTERGENIC	2	1.685	1.432	1.982	**3.24E-10**	0.022	0.037	5.50E-01	-0.080	0.123	5.16E-01	0.137	0.036	**1.50E-04**	-0.060	0.122	6.24E-01	-0.131	0.037	**3.30E-04**	-0.054	0.074	4.63E-01
rs482044	6	32684042	G/C	*HLA-DRB1 | HLA-DQA1*	INTERGENIC	2	0.594	0.505	0.699	**3.37E-10**	0.096	0.035	**5.70E-03**	-0.106	0.126	4.02E-01	0.133	0.034	**1.20E-04**	0.031	0.126	8.04E-01	-0.064	0.035	6.50E-02	-0.003	0.074	9.73E-01
rs1265099	6	31213392	G/A	*PSORS1C2*	UTR	2	1.620	1.393	1.883	**3.38E-10**	0.055	0.033	9.00E-02	-0.022	0.125	8.64E-01	0.048	0.032	1.35E-01	-0.136	0.123	2.73E-01	-0.002	0.033	9.60E-01	-0.028	0.075	7.12E-01
rs12180820	6	28424457	A/G	*ZNF323 | ZKSCAN3*	INTERGENIC	2	1.672	1.424	1.963	**3.51E-10**	-0.014	0.035	7.00E-01	-0.031	0.123	8.00E-01	0.063	0.035	7.10E-02	-0.142	0.121	2.47E-01	-0.073	0.035	**3.60E-02**	0.120	0.073	1.05E-01
rs9275601	6	32790642	A/G	*HLA-DQB1 | HLA-DQA2*	INTERGENIC	2	1.615	1.390	1.875	**3.51E-10**	0.121	0.032	**1.90E-04**	0.001	0.123	9.95E-01	0.031	0.032	3.30E-01	0.047	0.123	7.04E-01	0.070	0.032	**3.10E-02**	-0.033	0.074	6.51E-01
rs1062470	6	31192414	A/G	*CDSN*	CODING	2	0.564	0.471	0.674	**3.62E-10**	0.089	0.034	**9.00E-03**	0.114	0.122	3.56E-01	-0.165	0.034	**8.40E-07**	-0.100	0.122	4.12E-01	0.252	0.034	**8.50E-14**	0.123	0.073	9.54E-02
rs3117426	6	29379991	A/G	*LOC651503 | OR5U1*	INTERGENIC	2	1.696	1.438	2.001	**3.68E-10**	0.059	0.039	1.32E-01	0.020	0.123	8.69E-01	0.126	0.039	**1.26E-03**	-0.184	0.120	1.32E-01	-0.083	0.039	**3.50E-02**	-0.043	0.074	5.64E-01
rs6913660	6	27199404	A/C	*LOC100133205 | HIST1H2BJ*	INTERGENIC	2	1.774	1.482	2.122	**3.75E-10**	0.003	0.041	9.50E-01	0.079	0.123	5.24E-01	0.121	0.040	**2.70E-03**	-0.067	0.122	5.84E-01	-0.118	0.041	**3.80E-03**	0.059	0.074	4.30E-01
rs3094208	6	31198651	G/A	*CDSN | PSORS1C1*	INTERGENIC	2	0.569	0.477	0.679	**3.78E-10**	0.089	0.034	**8.60E-03**	0.106	0.123	3.93E-01	-0.162	0.034	**1.40E-06**	-0.104	0.123	3.98E-01	0.249	0.034	**1.80E-13**	0.095	0.075	2.05E-01
rs2267647	6	33083489	A/G	*HLA-DOA*	INTRON	2	1.626	1.397	1.894	**3.92E-10**	-0.041	0.033	2.20E-01	-0.152	0.122	2.15E-01	0.045	0.033	1.70E-01	-0.122	0.121	3.20E-01	-0.091	0.033	**6.20E-03**	-0.025	0.074	7.42E-01
rs2517911	6	29792613	G/A	*ZFP57 | HLA-F*	INTERGENIC	2	1.688	1.433	1.989	**3.97E-10**	0.040	0.036	2.70E-01	-0.099	0.122	4.23E-01	0.127	0.036	**3.80E-04**	-0.071	0.122	5.60E-01	-0.103	0.036	**4.20E-03**	-0.036	0.074	6.26E-01
rs3799380	6	26575161	G/A	*BTN2A1*	INTRON	2	1.751	1.469	2.087	**4.02E-10**	0.023	0.040	5.60E-01	0.038	0.123	7.57E-01	0.097	0.039	**1.39E-02**	-0.047	0.122	7.01E-01	-0.082	0.040	**3.80E-02**	0.003	0.074	9.68E-01
rs7749823	6	26266058	C/A	*HIST1H1E | HIST1H2BD*	INTERGENIC	2	1.902	1.555	2.327	**4.06E-10**	0.011	0.047	8.20E-01	0.028	0.124	8.20E-01	0.108	0.047	**2.10E-02**	-0.118	0.122	3.36E-01	-0.109	0.047	**2.00E-02**	-0.058	0.075	4.42E-01
rs3095297	6	31191708	C/A	*CDSN*	UTR	2	0.570	0.477	0.680	**4.10E-10**	-	-	-	0.114	0.122	3.56E-01	-	-	-	-0.100	0.122	4.12E-01	-	-	-	0.123	0.073	9.54E-02
rs2516464	6	31524135	G/A	*LOC100129668 | HCP5*	INTERGENIC	2	1.621	1.393	1.887	**4.30E-10**	-	-	-	-0.112	0.123	3.63E-01	-	-	-	-0.111	0.122	3.65E-01	-	-	-	0.025	0.074	7.36E-01
rs9348881	6	32404820	G/C	*LOC100131609 | C6orf10*	INTERGENIC	2	0.580	0.489	0.688	**4.30E-10**	-	-	-	-0.011	0.123	9.29E-01	-	-	-	0.053	0.122	6.66E-01	-	-	-	0.092	0.075	2.19E-01
rs7905	6	33140953	G/A	*HLA-DPA1*	UTR	2	1.862	1.532	2.264	**4.38E-10**	-0.005	0.047	9.20E-01	0.085	0.123	4.91E-01	0.042	0.047	3.70E-01	0.077	0.122	5.29E-01	-0.047	0.047	3.20E-01	-0.013	0.074	8.66E-01
rs7772827	6	28409122	G/A	*ZNF323*	INTRON	2	1.667	1.420	1.957	**4.39E-10**	-0.013	0.035	7.00E-01	-0.031	0.123	8.00E-01	0.063	0.035	7.10E-02	-0.142	0.121	2.47E-01	-0.073	0.035	**3.60E-02**	0.120	0.073	1.05E-01
rs2844645	6	31123161	A/G	*LOC729792 | HCG22*	INTERGENIC	2	1.639	1.403	1.915	**4.53E-10**	0.006	0.032	8.60E-01	-0.202	0.122	1.03E-01	0.030	0.031	3.30E-01	-0.005	0.124	9.70E-01	-0.032	0.032	3.10E-01	0.016	0.074	8.31E-01
rs3115570	6	32330684	G/A	*NOTCH4 | C6orf10*	INTERGENIC	2	0.590	0.500	0.696	**4.55E-10**	-	-	-	0.023	0.123	8.51E-01	-	-	-	0.102	0.122	4.09E-01	-	-	-	0.083	0.074	2.59E-01
rs13199775	6	25936761	T/A	*SLC17A1*	INTRON	2	1.998	1.607	2.484	**4.57E-10**	0.038	0.053	4.80E-01	0.027	0.123	8.25E-01	0.142	0.053	**7.20E-03**	-0.109	0.122	3.72E-01	-0.125	0.053	**1.90E-02**	-0.084	0.074	2.56E-01
rs1116221	6	30179309	A/G	*TRIM31*	CODING	2	1.669	1.421	1.961	**4.67E-10**	0.023	0.035	5.00E-01	-0.108	0.123	3.81E-01	0.075	0.035	**2.90E-02**	-0.199	0.120	1.03E-01	-0.055	0.035	1.14E-01	0.047	0.074	5.27E-01
rs1632988	6	29880374	A/G	*HCG4 | HLA-G*	INTERGENIC	1	1.770	1.479	2.118	**4.72E-10**	0.005	0.035	8.90E-01	-0.009	0.123	9.43E-01	0.101	0.034	**3.30E-03**	-0.064	0.122	6.01E-01	-0.099	0.035	**4.40E-03**	0.054	0.074	4.70E-01
rs1611350	6	29806800	G/A	*HLA-F | FLJ35429*	INTERGENIC	2	1.625	1.395	1.894	**4.90E-10**	0.047	0.035	1.80E-01	-0.033	0.123	7.88E-01	0.102	0.035	**3.30E-03**	-0.027	0.122	8.28E-01	-0.070	0.035	**4.60E-02**	-0.056	0.075	4.55E-01
rs1573646	6	32839602	C/A	*HLA-DQB2 | HLA-DOB*	INTERGENIC	2	0.600	0.511	0.705	**4.94E-10**	0.064	0.032	5.00E-02	0.293	0.118	**1.56E-02**	-0.090	0.032	**5.20E-03**	0.245	0.119	**4.37E-02**	0.157	0.032	**1.10E-06**	-0.067	0.074	3.67E-01
rs1737049	6	29843083	G/A	*IFITM4P | HCG4*	INTERGENIC	1	1.740	1.461	2.072	**4.99E-10**	-	-	-	-0.045	0.123	7.13E-01	-	-	-	-0.081	0.122	5.09E-01	-	-	-	0.028	0.074	7.01E-01
rs1632962	6	29791787	A/G	*ZFP57 | HLA-F*	INTERGENIC	2	1.685	1.429	1.986	**5.03E-10**	0.040	0.036	2.70E-01	-0.080	0.123	5.16E-01	0.128	0.036	**3.60E-04**	-0.060	0.122	6.24E-01	-0.104	0.036	**4.00E-03**	-0.054	0.074	4.63E-01
rs1416920	6	28410763	G/A	*ZNF323*	INTRON	2	1.663	1.416	1.952	**5.36E-10**	-0.013	0.035	7.00E-01	-0.031	0.123	8.00E-01	0.063	0.035	7.10E-02	-0.142	0.121	2.47E-01	-0.073	0.035	**3.60E-02**	0.120	0.073	1.05E-01

rs7382794	6	32842008	G/A	*HLA-DQB2 | HLA-DOB*	INTERGENIC	2	0.601	0.511	0.706	**5.58E-10**	0.064	0.032	5.00E-02	0.293	0.118	**1.56E-02**	-0.090	0.032	**5.20E-03**	0.245	0.119	**4.37E-02**	0.157	0.032	**1.10E-06**	-0.067	0.074	3.67E-01
rs9276598	6	32841965	A/G	*HLA-DQB2 | HLA-DOB*	INTERGENIC	2	0.601	0.511	0.706	**5.62E-10**	0.061	0.033	6.10E-02	0.293	0.118	**1.56E-02**	-0.088	0.032	**6.70E-03**	0.245	0.119	**4.37E-02**	0.153	0.032	**2.50E-06**	-0.068	0.074	3.60E-01
rs6456728	6	26585758	A/G	*BTN2A1 | BTN1A1*	INTERGENIC	2	1.751	1.467	2.091	**5.76E-10**	0.026	0.040	5.20E-01	0.038	0.123	7.57E-01	0.097	0.040	**1.39E-02**	-0.047	0.122	7.01E-01	-0.081	0.040	**4.30E-02**	0.003	0.074	9.68E-01
rs6902723	6	32839938	A/G	*HLA-DQB2 | HLA-DOB*	INTERGENIC	2	0.601	0.512	0.706	**5.80E-10**	0.064	0.032	5.00E-02	0.293	0.118	**1.56E-02**	-0.090	0.032	**5.20E-03**	0.245	0.119	**4.37E-02**	0.157	0.032	**1.10E-06**	-0.067	0.074	3.67E-01
rs238869	6	29463092	G/A	*OR12D3 | OR12D2*	INTERGENIC	2	1.632	1.398	1.906	**5.92E-10**	-0.003	0.033	9.20E-01	-0.021	0.126	8.70E-01	0.062	0.032	5.40E-02	0.002	0.125	9.90E-01	-0.063	0.032	5.00E-02	-0.102	0.074	1.69E-01
rs2072632	6	32029454	A/G	*RDBP*	INTRON	1	0.540	0.444	0.656	**6.00E-10**	0.042	0.035	2.30E-01	0.014	0.125	9.13E-01	-0.079	0.034	**2.10E-02**	0.079	0.124	5.28E-01	0.115	0.035	**8.40E-04**	0.030	0.075	6.85E-01
rs501220	6	25981004	A/C	*SLC17A3*	INTRON	2	1.865	1.531	2.272	**6.05E-10**	-0.001	0.049	9.80E-01	0.044	0.123	7.22E-01	0.118	0.048	**1.37E-02**	-0.035	0.122	7.74E-01	-0.133	0.048	**5.80E-03**	-0.116	0.073	1.17E-01
rs494620	6	31946692	A/G	*SLC44A4*	CODING	2	0.594	0.504	0.701	**6.23E-10**	0.004	0.032	9.10E-01	-0.029	0.123	8.17E-01	-0.113	0.032	**4.40E-04**	0.152	0.121	2.14E-01	0.130	0.032	**5.90E-05**	0.020	0.074	7.90E-01
rs2647050	6	32777745	G/A	*HLA-DQB1 | HLA-DQA2*	INTERGENIC	2	0.535	0.438	0.652	**6.40E-10**	0.108	0.033	**1.07E-03**	-0.037	0.123	7.65E-01	-0.066	0.033	**4.50E-02**	0.212	0.120	8.06E-02	0.172	0.033	**1.70E-07**	-0.023	0.074	7.56E-01
rs2571376	6	30049163	A/G	*HLA-A | HCG9*	INTERGENIC	2	1.626	1.393	1.898	**7.02E-10**	-0.012	0.034	7.20E-01	-0.031	0.123	8.03E-01	0.143	0.034	**2.00E-05**	-0.044	0.123	7.23E-01	-0.165	0.034	**1.10E-06**	-0.098	0.074	1.85E-01
rs6903130	6	32840188	A/G	*HLA-DQB2 | HLA-DOB*	INTERGENIC	2	0.611	0.522	0.715	**7.47E-10**	0.064	0.032	5.00E-02	0.293	0.118	**1.56E-02**	-0.090	0.032	**5.20E-03**	0.245	0.119	**4.37E-02**	0.157	0.032	**1.10E-06**	-0.067	0.074	3.67E-01
rs2735042	6	29855352	G/A	*IFITM4P | HCG4*	INTERGENIC	1	1.730	1.453	2.060	**7.53E-10**	0.002	0.034	9.50E-01	-0.045	0.123	7.13E-01	0.067	0.034	**4.70E-02**	-0.081	0.122	5.09E-01	-0.061	0.034	7.40E-02	0.028	0.074	7.01E-01
rs3129882	6	32517508	G/A	*HLA-DRA*	INTRON	2	0.602	0.512	0.708	**7.82E-10**	-0.081	0.032	**1.23E-02**	0.069	0.123	5.78E-01	0.080	0.032	**1.17E-02**	0.149	0.121	2.23E-01	-0.154	0.032	**1.60E-06**	-0.040	0.074	5.89E-01
rs3857546	6	26265741	A/G	*HIST1H1E | HIST1H2BD*	INTERGENIC	2	1.874	1.534	2.289	**7.96E-10**	0.008	0.047	8.70E-01	0.028	0.124	8.20E-01	0.107	0.047	**2.10E-02**	-0.118	0.122	3.36E-01	-0.111	0.047	**1.80E-02**	-0.058	0.075	4.42E-01
rs9295661	6	25558005	C/A	*LRRC16*	INTRON	2	2.053	1.632	2.582	**8.06E-10**	0.093	0.058	1.09E-01	-0.042	0.124	7.37E-01	0.154	0.057	**7.20E-03**	-0.165	0.121	1.80E-01	-0.085	0.058	1.39E-01	-0.076	0.074	3.03E-01
rs521539	6	32689951	A/G	*HLA-DRB1 | HLA-DQA1*	INTERGENIC	2	0.502	0.403	0.626	**8.19E-10**	-	-	-	0.080	0.123	5.17E-01	-	-	-	0.009	0.123	9.41E-01	-	-	-	-0.005	0.074	9.43E-01
rs6935723	6	32789647	G/A	*HLA-DQB1 | HLA-DQA2*	INTERGENIC	2	0.462	0.361	0.591	**8.47E-10**	0.110	0.037	**2.90E-03**	-0.151	0.122	2.20E-01	-0.057	0.036	1.15E-01	0.268	0.118	**2.58E-02**	0.160	0.037	**1.30E-05**	-0.052	0.074	4.88E-01
rs9379856	6	26474815	C/A	*BTN3A2*	INTRON	1	1.980	1.592	2.463	**8.48E-10**	0.071	0.048	1.35E-01	0.040	0.124	7.49E-01	0.173	0.047	**2.40E-04**	-0.065	0.123	6.01E-01	-0.126	0.047	**7.90E-03**	-0.069	0.075	3.58E-01
rs2844805	6	30048658	A/G	*HLA-A | HCG9*	INTERGENIC	2	1.635	1.397	1.914	**8.58E-10**	-0.012	0.034	7.20E-01	-0.042	0.123	7.36E-01	0.143	0.034	**2.00E-05**	-0.043	0.122	7.29E-01	-0.165	0.034	**1.10E-06**	-0.103	0.074	1.62E-01
rs2647089	6	32789546	G/A	*HLA-DQB1 | HLA-DQA2*	INTERGENIC	2	0.462	0.361	0.592	**8.63E-10**	0.110	0.037	**2.90E-03**	-0.151	0.122	2.20E-01	-0.057	0.036	1.15E-01	0.268	0.118	**2.58E-02**	0.160	0.037	**1.30E-05**	-0.052	0.074	4.88E-01
rs2647087	6	32789027	C/A	*HLA-DQB1 | HLA-DQA2*	INTERGENIC	2	0.463	0.361	0.592	**8.70E-10**	0.110	0.037	**2.90E-03**	-0.151	0.122	2.20E-01	-0.057	0.036	1.15E-01	0.268	0.118	**2.58E-02**	0.160	0.037	**1.30E-05**	-0.052	0.074	4.88E-01
rs7745656	6	32788948	A/C	*HLA-DQB1 | HLA-DQA2*	INTERGENIC	2	0.463	0.361	0.592	**8.70E-10**	0.110	0.037	**2.90E-03**	-0.151	0.122	2.20E-01	-0.057	0.036	1.15E-01	0.268	0.118	**2.58E-02**	0.160	0.037	**1.30E-05**	-0.052	0.074	4.88E-01
rs1324087	6	25949387	A/G	*SLC17A1 | SLC17A3*	INTERGENIC	2	1.863	1.526	2.273	**9.16E-10**	-0.007	0.048	8.90E-01	-0.030	0.123	8.10E-01	0.110	0.048	**2.10E-02**	-0.111	0.121	3.65E-01	-0.129	0.048	**7.30E-03**	-0.103	0.074	1.61E-01
rs1324088	6	25949101	A/G	*SLC17A1 | SLC17A3*	INTERGENIC	2	1.862	1.526	2.272	**9.37E-10**	-0.007	0.048	8.90E-01	-0.030	0.123	8.10E-01	0.110	0.048	**2.10E-02**	-0.111	0.121	3.65E-01	-0.129	0.048	**7.30E-03**	-0.103	0.074	1.61E-01
rs3095310	6	31199426	A/G	*CDSN | PSORS1C1*	INTERGENIC	1	0.550	0.454	0.666	**9.42E-10**	-	-	-	0.104	0.123	3.97E-01	-	-	-	0.011	0.122	9.31E-01	-	-	-	0.098	0.074	1.86E-01
rs7774434	6	32765556	G/A	*HLA-DQB1 | HLA-DQA2*	INTERGENIC	1	0.550	0.454	0.666	**9.42E-10**	0.010	0.033	7.60E-01	0.060	0.123	6.27E-01	-0.102	0.033	**1.80E-03**	0.174	0.121	1.53E-01	0.119	0.033	**3.20E-04**	-0.055	0.074	4.63E-01
rs6921948	6	31279236	C/A	*HCG27*	UTR	2	0.615	0.527	0.719	**9.47E-10**	0.045	0.031	1.50E-01	-0.073	0.125	5.62E-01	-0.178	0.031	**8.40E-09**	0.044	0.124	7.22E-01	0.229	0.031	**1.60E-13**	-0.086	0.074	2.44E-01
rs2894181	6	31282506	G/A	*HCG27 | HLA-C*	INTERGENIC	2	0.615	0.527	0.719	**9.53E-10**	-	-	-	-0.073	0.125	5.62E-01	-	-	-	0.044	0.124	7.22E-01	-	-	-	-0.086	0.074	2.44E-01
rs2894239	6	32323774	C/A	*NOTCH4 | C6orf10*	INTERGENIC	2	0.604	0.514	0.710	**9.54E-10**	0.035	0.034	3.00E-01	0.023	0.123	8.51E-01	-0.111	0.033	**9.40E-04**	0.102	0.122	4.09E-01	0.151	0.034	**7.60E-06**	0.083	0.074	2.59E-01
rs6457508	6	32324941	A/G	*NOTCH4 | C6orf10*	INTERGENIC	2	0.604	0.514	0.710	**9.63E-10**	0.063	0.033	5.50E-02	0.023	0.123	8.51E-01	-0.128	0.032	**7.80E-05**	0.102	0.122	4.09E-01	0.194	0.033	**2.70E-09**	0.083	0.074	2.59E-01
rs2894240	6	32325824	A/G	*NOTCH4 | C6orf10*	INTERGENIC	2	0.604	0.514	0.710	**9.66E-10**	0.063	0.033	5.50E-02	0.023	0.123	8.51E-01	-0.128	0.032	**7.80E-05**	0.102	0.122	4.09E-01	0.194	0.033	**2.70E-09**	0.083	0.074	2.59E-01
rs3115573	6	32326821	G/A	*NOTCH4 | C6orf10*	INTERGENIC	2	0.604	0.514	0.710	**9.66E-10**	0.063	0.033	5.50E-02	0.023	0.123	8.51E-01	-0.128	0.032	**7.80E-05**	0.102	0.122	4.09E-01	0.194	0.033	**2.70E-09**	0.083	0.074	2.59E-01
rs9267970	6	32325138	G/A	*NOTCH4 | C6orf10*	INTERGENIC	2	0.604	0.514	0.710	**9.66E-10**	0.063	0.033	5.50E-02	0.023	0.123	8.51E-01	-0.128	0.032	**7.80E-05**	0.102	0.122	4.09E-01	0.194	0.033	**2.70E-09**	0.083	0.074	2.59E-01
rs2227127	6	32819760	G/A	*HLA-DQA2*	INTRON	2	0.600	0.509	0.706	**9.67E-10**	0.022	0.033	5.10E-01	0.267	0.119	**2.76E-02**	-0.070	0.032	**3.10E-02**	0.155	0.121	2.04E-01	0.098	0.032	**2.60E-03**	-0.046	0.074	5.39E-01
rs3115576	6	32324828	T/A	*NOTCH4 | C6orf10*	INTERGENIC	2	0.604	0.514	0.710	**9.70E-10**	0.063	0.033	5.50E-02	0.023	0.123	8.51E-01	-0.128	0.032	**7.80E-05**	0.102	0.122	4.09E-01	0.194	0.033	**2.70E-09**	0.083	0.074	2.59E-01
rs440169	6	32321766	A/G	*NOTCH4 | C6orf10*	INTERGENIC	2	0.604	0.514	0.710	**9.85E-10**	0.063	0.033	5.50E-02	0.023	0.123	8.51E-01	-0.128	0.032	**7.80E-05**	0.102	0.122	4.09E-01	0.194	0.033	**2.70E-09**	0.083	0.074	2.59E-01
rs2844503	6	31550710	T/A	*3.8-1 | MICB*	INTERGENIC	2	0.608	0.518	0.713	**1.03E-09**	-0.051	0.032	1.14E-01	0.129	0.122	2.93E-01	-0.195	0.032	**9.80E-10**	0.237	0.119	5.04E-02	0.166	0.032	**2.00E-07**	0.000	0.075	9.96E-01
rs2523962	6	30047523	A/G	*HLA-A | HCG9*	INTERGENIC	2	1.621	1.388	1.893	**1.11E-09**	-0.011	0.034	7.40E-01	-0.030	0.124	8.10E-01	0.142	0.034	**2.40E-05**	-0.042	0.124	7.35E-01	-0.162	0.034	**1.60E-06**	-0.098	0.074	1.85E-01
rs2523964	6	30047273	A/G	*HLA-A | HCG9*	INTERGENIC	2	1.621	1.387	1.893	**1.12E-09**	-0.009	0.034	7.90E-01	-0.031	0.123	8.03E-01	0.137	0.034	**4.70E-05**	-0.044	0.123	7.23E-01	-0.155	0.034	**4.80E-06**	-0.098	0.074	1.85E-01
rs1150711	6	28316514	G/A	*ZNF193 | ZKSCAN4*	INTERGENIC	2	1.615	1.384	1.885	**1.13E-09**	-0.002	0.033	9.40E-01	0.014	0.123	9.11E-01	0.065	0.033	**4.80E-02**	-0.075	0.122	5.42E-01	-0.070	0.033	**3.50E-02**	0.101	0.074	1.73E-01
rs629444	6	25993793	A/G	*SLC17A3 | SLC17A2*	INTERGENIC	2	1.876	1.532	2.298	**1.18E-09**	0.007	0.049	8.90E-01	0.085	0.123	4.90E-01	0.155	0.048	**1.36E-03**	-0.050	0.122	6.85E-01	-0.166	0.049	**6.70E-04**	-0.078	0.074	2.89E-01
rs2844767	6	30330235	A/G	*TRIM26 | FLJ45422*	INTERGENIC	2	1.604	1.377	1.867	**1.20E-09**	-	-	-	0.132	0.122	2.85E-01	-	-	-	0.029	0.122	8.14E-01	-	-	-	0.081	0.074	2.71E-01
rs2395110	6	32323854	A/G	*NOTCH4 | C6orf10*	INTERGENIC	2	0.606	0.516	0.712	**1.23E-09**	0.063	0.033	5.50E-02	0.023	0.123	8.51E-01	-0.128	0.032	**7.80E-05**	0.102	0.122	4.09E-01	0.194	0.033	**2.70E-09**	0.083	0.074	2.59E-01
rs405875	6	32323166	G/A	*NOTCH4 | C6orf10*	INTERGENIC	2	0.606	0.516	0.712	**1.23E-09**	0.063	0.033	5.50E-02	0.023	0.123	8.51E-01	-0.128	0.032	**7.80E-05**	0.102	0.122	4.09E-01	0.194	0.033	**2.70E-09**	0.083	0.074	2.59E-01
rs35252817	6	30446227	G/A	*RPP21 | HLA-E*	INTERGENIC	2	1.599	1.374	1.861	**1.27E-09**	-	-	-	-0.147	0.122	2.31E-01	-	-	-	-0.094	0.122	4.45E-01	-	-	-	0.052	0.074	4.80E-01
rs3873386	6	31381724	G/A	*HLA-C | HLA-B*	INTERGENIC	2	0.611	0.521	0.717	**1.27E-09**	0.066	0.033	**4.60E-02**	0.100	0.125	4.25E-01	-0.092	0.033	**4.90E-03**	0.262	0.120	**3.27E-02**	0.157	0.033	**1.60E-06**	-0.080	0.074	2.84E-01
rs9368737	6	32766407	G/A	*HLA-DQB1 | HLA-DQA2*	INTERGENIC	2	0.536	0.438	0.656	**1.29E-09**	0.107	0.033	**1.28E-03**	0.024	0.123	8.47E-01	-0.066	0.033	**4.60E-02**	0.278	0.118	**2.12E-02**	0.171	0.033	**2.40E-07**	-0.030	0.074	6.92E-01
rs9268521	6	32489352	G/C	*BTNL2 | HLA-DRA*	INTERGENIC	2	0.518	0.419	0.641	**1.34E-09**	-	-	-	0.001	0.124	9.93E-01	-	-	-	-0.012	0.123	9.22E-01	-	-	-	0.053	0.074	4.76E-01
rs9296042	6	32843983	A/G	*HLA-DQB2 | HLA-DOB*	INTERGENIC	2	0.606	0.516	0.713	**1.42E-09**	0.023	0.033	4.80E-01	0.293	0.118	**1.55E-02**	-0.077	0.033	**1.80E-02**	0.143	0.121	2.42E-01	0.109	0.033	**8.80E-04**	-0.042	0.074	5.73E-01
rs2256922	6	30048793	A/G	*HLA-A | HCG9*	INTERGENIC	2	1.610	1.380	1.879	**1.45E-09**	-0.012	0.034	7.20E-01	-0.031	0.123	8.03E-01	0.143	0.034	**2.00E-05**	-0.044	0.123	7.23E-01	-0.165	0.034	**1.10E-06**	-0.098	0.074	1.85E-01
rs915894	6	32298368	C/A	*NOTCH4*	CODING	2	1.591	1.369	1.849	**1.46E-09**	0.129	0.033	**8.70E-05**	-0.044	0.124	7.25E-01	-0.018	0.033	5.90E-01	-0.044	0.123	7.21E-01	0.132	0.033	**6.00E-05**	0.018	0.075	8.05E-01
rs9268345	6	32439819	A/G	*C6orf10*	INTRON	1	0.560	0.464	0.676	**1.50E-09**	-	-	-	-0.011	0.123	9.29E-01	-	-	-	0.053	0.122	6.66E-01	-	-	-	0.101	0.074	1.71E-01
rs1012411	6	30440534	C/A	*RPP21 | HLA-E*	INTERGENIC	2	1.621	1.386	1.896	**1.54E-09**	-0.021	0.033	5.30E-01	0.078	0.123	5.29E-01	0.102	0.033	**2.10E-03**	-0.033	0.122	7.85E-01	-0.127	0.033	**1.30E-04**	0.021	0.074	7.77E-01
rs13203429	6	32852830	C/G	*HLA-DQB2 | HLA-DOB*	INTERGENIC	2	0.598	0.506	0.706	**1.55E-09**	-0.018	0.034	5.90E-01	0.148	0.122	2.29E-01	-0.092	0.033	**5.90E-03**	0.044	0.122	7.22E-01	0.081	0.034	**1.60E-02**	0.024	0.074	7.42E-01
rs9268614	6	32510756	C/A	*BTNL2 | HLA-DRA*	INTERGENIC	2	0.516	0.416	0.640	**1.59E-09**	0.038	0.039	3.30E-01	0.001	0.124	9.93E-01	-0.089	0.038	**1.90E-02**	-0.012	0.123	9.22E-01	0.127	0.038	**9.00E-04**	0.024	0.074	7.41E-01
rs2856726	6	32774699	A/T	*HLA-DQB1 | HLA-DQA2*	INTERGENIC	2	0.538	0.440	0.659	**1.66E-09**	0.106	0.033	**1.35E-03**	-0.042	0.124	7.35E-01	-0.066	0.033	**4.30E-02**	0.210	0.121	8.67E-02	0.171	0.033	**2.20E-07**	-0.025	0.075	7.37E-01
rs4604307	6	33133649	G/C	*HLA-DOA | HLA-DPA1*	INTERGENIC	1	1.890	1.537	2.325	**1.68E-09**	-0.058	0.041	1.60E-01	0.053	0.123	6.68E-01	0.023	0.041	5.80E-01	0.016	0.122	8.97E-01	-0.082	0.041	**4.70E-02**	-0.001	0.074	9.92E-01
rs9275205	6	32765538	G/A	*HLA-DQB1 | HLA-DQA2*	INTERGENIC	1	0.440	0.337	0.575	**1.68E-09**	-	-	-	0.025	0.124	8.43E-01	-	-	-	0.264	0.119	**3.04E-02**	-	-	-	-0.042	0.074	5.73E-01
rs13215054	6	28610773	A/C	*GPX5 | ZNF452*	INTERGENIC	2	1.660	1.407	1.957	**1.71E-09**	-0.021	0.036	5.70E-01	-0.135	0.123	2.78E-01	0.059	0.036	9.60E-02	-0.188	0.121	1.26E-01	-0.075	0.036	**3.70E-02**	-0.007	0.074	9.28E-01
rs3130724	6	29226509	A/C	*OR2J3 | OR2J2*	INTERGENIC	2	1.654	1.404	1.948	**1.74E-09**	0.043	0.039	2.70E-01	0.001	0.123	9.96E-01	0.094	0.039	**1.60E-02**	-0.179	0.120	1.42E-01	-0.056	0.039	1.50E-01	-0.044	0.074	5.51E-01
rs9268362	6	32441319	G/A	*C6orf10*	INTRON	2	0.519	0.419	0.642	**1.81E-09**	0.045	0.038	2.40E-01	-0.088	0.123	4.73E-01	-0.054	0.038	1.60E-01	-0.120	0.121	3.25E-01	0.092	0.038	**1.70E-02**	0.054	0.074	4.69E-01
rs6457617	6	32771829	A/G	*HLA-DQB1 | HLA-DQA2*	INTERGENIC	2	0.614	0.524	0.720	**1.87E-09**	0.095	0.032	**2.80E-03**	0.081	0.123	5.10E-01	-0.022	0.031	4.80E-01	0.121	0.121	3.22E-01	0.104	0.032	**1.02E-03**	-0.032	0.074	6.64E-01
rs6457620	6	32771977	C/G	*HLA-DQB1 | HLA-DQA2*	INTERGENIC	2	0.614	0.524	0.720	**1.87E-09**	0.095	0.032	**2.80E-03**	0.081	0.123	5.10E-01	-0.024	0.031	4.40E-01	0.121	0.121	3.22E-01	0.106	0.032	**7.80E-04**	-0.031	0.074	6.75E-01
rs1264513	6	30520341	A/G	*RPP21 | HLA-E*	INTERGENIC	2	1.597	1.371	1.861	**1.92E-09**	-0.056	0.032	7.90E-02	0.007	0.124	9.56E-01	0.059	0.032	6.10E-02	0.002	0.123	9.89E-01	-0.107	0.032	**8.50E-04**	-0.037	0.074	6.20E-01
rs1736998	6	29873695	A/T	*HCG4 | HLA-G*	INTERGENIC	1	1.710	1.435	2.037	**1.94E-09**	0.014	0.033	6.70E-01	-0.139	0.123	2.62E-01	0.037	0.033	2.70E-01	-0.118	0.123	3.37E-01	-0.015	0.033	6.40E-01	0.001	0.075	9.92E-01
rs2517532	6	31126386	A/G	*LOC729792 | HCG22*	INTERGENIC	2	0.625	0.536	0.729	**2.05E-09**	0.034	0.033	3.00E-01	0.220	0.120	7.15E-02	-0.023	0.032	4.70E-01	-0.067	0.122	5.86E-01	0.043	0.033	1.90E-01	0.047	0.074	5.30E-01
rs2523963	6	30047462	A/T	*HLA-A | HCG9*	INTERGENIC	2	1.612	1.379	1.885	**2.05E-09**	-0.009	0.034	7.90E-01	-0.031	0.123	8.03E-01	0.137	0.034	**4.70E-05**	-0.044	0.123	7.23E-01	-0.155	0.034	**4.80E-06**	-0.098	0.074	1.85E-01
rs2072806	6	26493072	G/C	*BTN2A2*	INTRON	1	1.950	1.567	2.426	**2.06E-09**	0.083	0.047	8.10E-02	0.040	0.124	7.49E-01	0.166	0.047	**4.10E-04**	-0.065	0.123	6.01E-01	-0.107	0.047	**2.40E-02**	-0.076	0.074	3.11E-01
rs3800316	6	27364081	C/A	*PRSS16 | POM121L2*	INTERGENIC	2	1.646	1.398	1.937	**2.11E-09**	-0.004	0.036	9.00E-01	-0.023	0.123	8.50E-01	0.048	0.036	1.80E-01	-0.102	0.122	4.08E-01	-0.041	0.036	2.50E-01	0.020	0.075	7.95E-01

rs2858332	6	32789139	A/C	*HLA-DQB1 | HLA-DQA2*	INTERGENIC	2	0.615	0.524	0.721	**2.12E-09**	0.080	0.032	**1.21E-02**	0.040	0.123	7.44E-01	-0.061	0.031	5.40E-02	0.287	0.117	**1.69E-02**	0.131	0.032	**3.70E-05**	-0.047	0.074	5.23E-01
rs2071279	6	32272852	A/C	*NOTCH4*	INTRON	2	0.555	0.458	0.673	**2.22E-09**	0.037	0.037	3.10E-01	0.017	0.123	8.91E-01	-0.077	0.037	**3.70E-02**	0.255	0.118	**3.50E-02**	0.121	0.037	**1.04E-03**	0.025	0.074	7.37E-01
rs2535324	6	30826014	C/A	*IER3 | DDR1*	INTERGENIC	2	1.609	1.377	1.880	**2.24E-09**	0.028	0.034	4.10E-01	-0.080	0.123	5.19E-01	0.057	0.033	8.70E-02	-0.248	0.119	**4.10E-02**	-0.030	0.034	3.80E-01	-0.014	0.074	8.48E-01
rs2073529	6	26483138	G/A	*BTN3A2*	INTRON	1	1.940	1.561	2.411	**2.33E-09**	0.074	0.048	1.21E-01	0.040	0.124	7.49E-01	0.176	0.047	**1.90E-04**	-0.065	0.123	6.01E-01	-0.127	0.048	**7.60E-03**	-0.073	0.074	3.26E-01
rs9262492	6	31093994	G/A	*C6orf205 | LOC729792*	INTERGENIC	2	0.580	0.485	0.694	**2.45E-09**	0.043	0.033	2.00E-01	-0.182	0.121	1.39E-01	-0.117	0.033	**3.70E-04**	-0.043	0.123	7.25E-01	0.157	0.033	**2.10E-06**	-0.059	0.074	4.25E-01
rs9262498	6	31094814	G/A	*C6orf205 | LOC729792*	INTERGENIC	2	0.580	0.485	0.694	**2.45E-09**	0.023	0.033	4.90E-01	-0.182	0.121	1.39E-01	-0.121	0.033	**2.50E-04**	-0.043	0.123	7.25E-01	0.145	0.033	**1.10E-05**	-0.059	0.074	4.25E-01
rs9267833	6	32285878	G/A	*NOTCH4*	INTRON	2	0.556	0.458	0.674	**2.54E-09**	0.036	0.037	3.30E-01	0.017	0.123	8.91E-01	-0.073	0.037	**4.70E-02**	0.255	0.118	**3.50E-02**	0.116	0.037	**1.60E-03**	0.027	0.074	7.10E-01
rs1633105	6	29891361	G/A	*HCG4 | HLA-G*	INTERGENIC	2	1.581	1.360	1.839	**2.64E-09**	0.015	0.032	6.40E-01	-0.107	0.123	3.86E-01	0.049	0.031	1.18E-01	-0.049	0.123	6.90E-01	-0.030	0.032	3.40E-01	-0.003	0.074	9.69E-01
rs6932590	6	27356910	G/A	*PRSS16 | POM121L2*	INTERGENIC	2	1.652	1.400	1.948	**2.67E-09**	0.006	0.036	8.80E-01	0.051	0.123	6.81E-01	0.072	0.036	**4.60E-02**	-0.058	0.122	6.35E-01	-0.064	0.036	7.90E-02	0.038	0.075	6.11E-01
rs2844730	6	30525593	A/G	*RPP21 | HLA-E*	INTERGENIC	2	1.573	1.355	1.827	**2.78E-09**	-0.055	0.032	8.60E-02	0.007	0.124	9.56E-01	0.058	0.032	6.60E-02	0.002	0.123	9.89E-01	-0.104	0.032	**1.09E-03**	-0.041	0.074	5.83E-01
rs4424066	6	32462406	G/A	*C6orf10 | BTNL2*	INTERGENIC	2	0.608	0.516	0.716	**2.84E-09**	0.031	0.032	3.30E-01	-0.027	0.123	8.26E-01	-0.094	0.032	**3.30E-03**	0.098	0.122	4.25E-01	0.129	0.032	**6.30E-05**	0.020	0.074	7.87E-01
rs2516674	6	30534642	G/C	*RPP21 | HLA-E*	INTERGENIC	2	1.573	1.355	1.827	**2.86E-09**	-	-	-	0.007	0.124	9.56E-01	-	-	-	0.002	0.123	9.89E-01	-	-	-	-0.041	0.074	5.80E-01
rs17587226	6	26070283	A/G	*SLC17A2 | TRIM38*	INTERGENIC	2	1.920	1.548	2.382	**2.87E-09**	0.051	0.051	3.10E-01	0.068	0.123	5.80E-01	0.176	0.051	**5.10E-04**	-0.104	0.122	3.97E-01	-0.149	0.051	**3.50E-03**	-0.056	0.074	4.51E-01
rs2254386	6	31533012	G/A	*LOC100129668 | HCP5*	INTERGENIC	2	1.597	1.369	1.865	**2.93E-09**	-	-	-	-0.041	0.123	7.39E-01	-	-	-	-0.116	0.122	3.44E-01	-	-	-	0.045	0.074	5.46E-01
rs1233699	6	28277137	T/A	*ZNF192 | LOC222699*	INTERGENIC	2	1.623	1.383	1.905	**2.94E-09**	0.079	0.037	**3.60E-02**	0.054	0.124	6.62E-01	0.120	0.037	**1.21E-03**	-0.118	0.123	3.41E-01	-0.049	0.037	1.80E-01	-0.006	0.074	9.37E-01
rs2074473	6	30262178	G/A	*TRIM26*	CODING	2	1.579	1.358	1.836	**2.94E-09**	0.099	0.033	**2.70E-03**	0.114	0.122	3.55E-01	0.010	0.033	7.50E-01	0.030	0.122	8.07E-01	0.080	0.033	**1.49E-02**	0.097	0.074	1.89E-01
rs2516459	6	31527021	G/A	*LOC100129668 | HCP5*	INTERGENIC	2	1.597	1.368	1.865	**2.95E-09**	-0.008	0.031	8.10E-01	-0.041	0.123	7.39E-01	0.045	0.031	1.47E-01	-0.116	0.122	3.44E-01	-0.055	0.031	7.70E-02	0.045	0.074	5.46E-01
rs2523693	6	31526103	A/G	*LOC100129668 | HCP5*	INTERGENIC	2	1.597	1.368	1.865	**2.95E-09**	-0.008	0.031	8.10E-01	-0.041	0.123	7.39E-01	0.045	0.031	1.47E-01	-0.116	0.122	3.44E-01	-0.055	0.031	7.70E-02	0.045	0.074	5.46E-01
rs6457509	6	32324996	C/G	*NOTCH4 | C6orf10*	INTERGENIC	2	0.604	0.511	0.713	**2.97E-09**	0.063	0.033	5.50E-02	0.023	0.123	8.51E-01	-0.128	0.032	**7.80E-05**	0.102	0.122	4.09E-01	0.194	0.033	**2.70E-09**	0.083	0.074	2.59E-01
rs2523971	6	30046237	A/C	*HLA-A | HCG9*	INTERGENIC	2	1.617	1.379	1.895	**3.00E-09**	-0.007	0.035	8.40E-01	-0.011	0.123	9.32E-01	0.172	0.034	**5.40E-07**	-0.033	0.122	7.89E-01	-0.194	0.034	**1.90E-08**	-0.081	0.074	2.75E-01
rs9275338	6	32775321	T/A	*HLA-DQB1 | HLA-DQA2*	INTERGENIC	2	0.482	0.378	0.613	**3.00E-09**	0.060	0.047	2.00E-01	0.106	0.123	3.91E-01	0.009	0.046	8.50E-01	0.112	0.122	3.61E-01	0.037	0.046	4.20E-01	-0.047	0.074	5.26E-01
rs3131115	6	30576770	A/G	*HLA-E | GNL1*	INTERGENIC	2	1.588	1.363	1.851	**3.12E-09**	0.012	0.033	7.00E-01	-0.212	0.120	8.30E-02	0.075	0.032	**1.90E-02**	-0.292	0.117	**1.53E-02**	-0.059	0.032	6.80E-02	0.093	0.074	2.07E-01
rs765977	6	30265633	A/G	*TRIM26*	INTRON	2	1.583	1.360	1.843	**3.17E-09**	0.099	0.033	**2.70E-03**	0.128	0.123	2.98E-01	0.010	0.033	7.50E-01	0.025	0.123	8.38E-01	0.080	0.033	**1.49E-02**	0.105	0.074	1.55E-01
rs6457164	6	30263087	A/G	*TRIM26*	INTRON	2	1.583	1.359	1.842	**3.21E-09**	0.099	0.033	**2.70E-03**	0.128	0.123	2.98E-01	0.010	0.033	7.50E-01	0.025	0.123	8.38E-01	0.080	0.033	**1.49E-02**	0.105	0.074	1.55E-01
rs2395471	6	31348671	A/G	*HLA-C | HLA-B*	INTERGENIC	2	0.592	0.498	0.705	**3.28E-09**	0.036	0.033	2.80E-01	0.138	0.125	2.72E-01	-0.122	0.033	**2.20E-04**	0.280	0.120	**2.29E-02**	0.155	0.033	**2.80E-06**	0.047	0.078	5.49E-01
rs853683	6	28403018	G/A	*ZNF323*	INTRON	2	1.600	1.369	1.870	**3.34E-09**	-0.006	0.033	8.70E-01	-0.017	0.123	8.90E-01	0.057	0.033	8.50E-02	-0.081	0.122	5.10E-01	-0.063	0.033	6.00E-02	0.089	0.074	2.32E-01
rs149901	6	28073482	A/G	*LOC442175 | ZNF165*	INTERGENIC	2	1.626	1.384	1.910	**3.40E-09**	0.089	0.037	**1.80E-02**	0.052	0.124	6.73E-01	0.128	0.037	**5.30E-04**	0.023	0.123	8.53E-01	-0.049	0.037	1.90E-01	-0.047	0.074	5.32E-01
rs2284164	6	30267833	C/A	*TRIM26*	INTRON	2	1.581	1.358	1.840	**3.53E-09**	0.098	0.033	**3.00E-03**	0.128	0.123	2.98E-01	0.010	0.033	7.60E-01	0.025	0.123	8.38E-01	0.080	0.033	**1.50E-02**	0.105	0.074	1.55E-01
rs718254	6	30267018	C/G	*TRIM26*	INTRON	2	1.580	1.358	1.840	**3.58E-09**	0.099	0.033	**2.70E-03**	0.128	0.123	2.98E-01	0.010	0.033	7.50E-01	0.025	0.123	8.38E-01	0.080	0.033	**1.49E-02**	0.105	0.074	1.55E-01
rs13195441	6	32851276	A/G	*HLA-DQB2 | HLA-DOB*	INTERGENIC	2	0.580	0.484	0.695	**3.62E-09**	-0.054	0.037	1.41E-01	0.250	0.120	**4.11E-02**	-0.080	0.036	**2.80E-02**	0.142	0.122	2.47E-01	0.036	0.037	3.30E-01	-0.003	0.074	9.63E-01
rs2260000	6	31701455	G/A	*BAT2*	INTRON	2	0.601	0.508	0.712	**3.62E-09**	0.070	0.034	**4.10E-02**	0.085	0.123	4.92E-01	-0.105	0.034	**1.80E-03**	-0.084	0.122	4.95E-01	0.163	0.034	**1.40E-06**	0.062	0.074	4.01E-01
rs3793127	6	32479893	A/G	*BTNL2*	INTRON	2	0.518	0.416	0.645	**3.70E-09**	0.051	0.039	1.80E-01	0.001	0.124	9.93E-01	-0.070	0.038	6.60E-02	-0.012	0.123	9.22E-01	0.121	0.038	**1.60E-03**	0.061	0.074	4.10E-01
rs1997660	6	28377642	G/A	*PGBD1*	CODING	2	1.607	1.373	1.881	**3.72E-09**	-0.006	0.033	8.70E-01	-0.017	0.123	8.90E-01	0.057	0.033	8.50E-02	-0.081	0.122	5.10E-01	-0.063	0.033	6.00E-02	0.089	0.074	2.32E-01
rs9468333	6	28379177	A/G	*PGBD1 | ZNF323*	INTERGENIC	2	1.607	1.373	1.881	**3.72E-09**	-	-	-	-0.017	0.123	8.90E-01	-	-	-	-0.081	0.122	5.10E-01	-	-	-	0.089	0.074	2.32E-01
rs6936428	6	32847152	T/A	*HLA-DQB2 | HLA-DOB*	INTERGENIC	2	0.623	0.532	0.729	**3.73E-09**	0.027	0.033	4.10E-01	0.311	0.117	**9.98E-03**	-0.076	0.033	**1.90E-02**	0.120	0.122	3.26E-01	0.111	0.033	**6.80E-04**	-0.054	0.074	4.63E-01
rs1778484	6	28348777	G/A	*ZNF187*	INTRON	2	1.593	1.365	1.860	**3.76E-09**	-0.019	0.033	5.60E-01	-0.008	0.123	9.47E-01	0.061	0.033	6.20E-02	-0.105	0.122	3.93E-01	-0.081	0.033	**1.35E-02**	0.079	0.074	2.84E-01
rs853690	6	28393461	A/G	*PGBD1 | ZNF323*	INTERGENIC	2	1.607	1.372	1.881	**3.76E-09**	0.000	0.033	1.00E+00	-0.017	0.123	8.90E-01	0.077	0.033	**2.00E-02**	-0.081	0.122	5.10E-01	-0.080	0.033	**1.70E-02**	0.089	0.074	2.32E-01
rs2076530	6	32471794	G/A	*BTNL2*	CODING	2	0.611	0.518	0.720	**3.77E-09**	0.031	0.032	3.30E-01	-0.027	0.123	8.26E-01	-0.094	0.032	**3.30E-03**	0.098	0.122	4.25E-01	0.129	0.032	**6.30E-05**	0.020	0.074	7.87E-01
rs707907	6	28399219	G/A	*PGBD1 | ZNF323*	INTERGENIC	2	1.606	1.372	1.881	**3.84E-09**	-0.006	0.033	8.70E-01	-0.017	0.123	8.90E-01	0.057	0.033	8.50E-02	-0.081	0.122	5.10E-01	-0.063	0.033	6.00E-02	0.089	0.074	2.32E-01
rs165255	6	30097674	C/G	*HCG9 | ZNRD1*	INTERGENIC	2	1.611	1.374	1.888	**4.00E-09**	-0.010	0.034	7.80E-01	-0.049	0.123	6.93E-01	0.167	0.034	**9.00E-07**	-0.032	0.123	7.92E-01	-0.191	0.034	**2.40E-08**	-0.091	0.074	2.20E-01
rs259929	6	30110031	G/A	*HCG9 | ZNRD1*	INTERGENIC	2	1.611	1.374	1.888	**4.00E-09**	-0.010	0.034	7.80E-01	-0.049	0.123	6.93E-01	0.167	0.034	**9.00E-07**	-0.032	0.123	7.92E-01	-0.191	0.034	**2.40E-08**	-0.091	0.074	2.20E-01
rs3800307	6	27293771	T/A	*HIST1H2AH | PRSS16*	INTERGENIC	2	1.690	1.419	2.013	**4.06E-09**	0.022	0.038	5.70E-01	0.107	0.123	3.86E-01	0.099	0.038	**9.30E-03**	-0.077	0.122	5.33E-01	-0.076	0.038	**4.60E-02**	0.050	0.075	5.06E-01
rs3130391	6	30298619	A/G	*TRIM26 | FLJ45422*	INTERGENIC	2	1.587	1.361	1.851	**4.12E-09**	0.103	0.033	**1.80E-03**	0.128	0.122	2.98E-01	0.018	0.033	5.70E-01	0.032	0.123	7.95E-01	0.075	0.033	**2.30E-02**	0.098	0.074	1.87E-01
rs2259571	6	31691806	C/A	*AIF1 / AIF1*	UTR	2	0.603	0.509	0.714	**4.19E-09**	0.048	0.035	1.70E-01	0.085	0.123	4.92E-01	-0.080	0.035	**2.20E-02**	-0.084	0.122	4.95E-01	0.116	0.035	**9.40E-04**	0.062	0.074	4.01E-01
rs13196552	6	26082183	G/A	*TRIM38*	INTRON	2	1.865	1.515	2.296	**4.20E-09**	0.021	0.049	6.70E-01	0.026	0.123	8.34E-01	0.108	0.048	**2.50E-02**	-0.113	0.122	3.55E-01	-0.105	0.048	**3.10E-02**	-0.077	0.074	2.98E-01
rs9469246	6	32800205	C/A	*HLA-DQB1 | HLA-DQA2*	INTERGENIC	2	0.613	0.520	0.722	**4.24E-09**	0.020	0.033	5.30E-01	0.267	0.119	**2.76E-02**	-0.061	0.032	5.90E-02	0.155	0.121	2.04E-01	0.087	0.032	**7.60E-03**	-0.045	0.074	5.40E-01
rs3130389	6	30296178	A/G	*TRIM26 | FLJ45422*	INTERGENIC	2	1.571	1.351	1.827	**4.25E-09**	-	-	-	0.128	0.122	2.98E-01	-	-	-	0.032	0.123	7.95E-01	-	-	-	0.105	0.074	1.54E-01
rs3132674	6	30310408	A/G	*TRIM26 | FLJ45422*	INTERGENIC	2	1.571	1.351	1.827	**4.25E-09**	0.103	0.033	**1.80E-03**	0.128	0.122	2.98E-01	0.018	0.033	5.70E-01	0.032	0.123	7.95E-01	0.075	0.033	**2.30E-02**	0.105	0.074	1.54E-01
rs916571	6	30309934	A/G	*TRIM26 | FLJ45422*	INTERGENIC	2	1.571	1.351	1.826	**4.26E-09**	0.103	0.033	**1.80E-03**	0.128	0.122	2.98E-01	0.018	0.033	5.70E-01	0.032	0.123	7.95E-01	0.075	0.033	**2.30E-02**	0.105	0.074	1.54E-01
rs9263875	6	31278893	G/A	*HCG27*	UTR	2	0.622	0.531	0.729	**4.26E-09**	0.070	0.033	**3.30E-02**	-0.016	0.123	9.00E-01	-0.196	0.032	**1.40E-09**	0.090	0.122	4.64E-01	0.270	0.033	**1.20E-16**	-0.015	0.074	8.44E-01
rs6940007	6	26039936	C/A	*SLC17A2 | TRIM38*	INTERGENIC	2	1.884	1.525	2.328	**4.28E-09**	0.050	0.051	3.20E-01	0.003	0.123	9.79E-01	0.182	0.050	**2.90E-04**	-0.137	0.121	2.63E-01	-0.157	0.051	**1.90E-03**	-0.083	0.074	2.61E-01
rs1362089	6	30317781	C/A	*TRIM26 | FLJ45422*	INTERGENIC	2	1.577	1.354	1.835	**4.30E-09**	0.102	0.033	**2.00E-03**	0.117	0.122	3.41E-01	0.021	0.033	5.20E-01	0.019	0.122	8.75E-01	0.073	0.033	**2.60E-02**	0.101	0.074	1.71E-01
rs2285801	6	30317472	G/A	*TRIM26 | FLJ45422*	INTERGENIC	2	1.577	1.354	1.835	**4.31E-09**	0.102	0.033	**2.00E-03**	0.117	0.122	3.41E-01	0.021	0.033	5.20E-01	0.019	0.122	8.75E-01	0.073	0.033	**2.60E-02**	0.101	0.074	1.71E-01
rs213230	6	28438243	G/A	*ZKSCAN3*	INTRON	2	1.630	1.385	1.920	**4.38E-09**	0.004	0.035	9.00E-01	-0.040	0.123	7.48E-01	0.096	0.035	**6.00E-03**	-0.143	0.121	2.43E-01	-0.095	0.035	**7.30E-03**	0.138	0.073	6.13E-02
rs1264550	6	30485177	A/G	*RPP21 | HLA-E*	INTERGENIC	2	1.569	1.350	1.823	**4.39E-09**	-0.052	0.032	1.05E-01	-0.004	0.123	9.76E-01	0.064	0.032	**4.50E-02**	0.001	0.123	9.93E-01	-0.108	0.032	**7.70E-04**	-0.046	0.074	5.33E-01
rs1264562	6	30480791	A/C	*RPP21 | HLA-E*	INTERGENIC	2	1.569	1.350	1.823	**4.42E-09**	-0.052	0.032	1.05E-01	-0.004	0.123	9.76E-01	0.064	0.032	**4.50E-02**	0.001	0.123	9.93E-01	-0.108	0.032	**7.70E-04**	-0.046	0.074	5.33E-01
rs259934	6	30114855	A/G	*HCG9 | ZNRD1*	INTERGENIC	2	1.609	1.372	1.886	**4.43E-09**	-0.010	0.034	7.80E-01	-0.052	0.123	6.73E-01	0.167	0.034	**9.00E-07**	-0.041	0.123	7.42E-01	-0.191	0.034	**2.40E-08**	-0.092	0.074	2.14E-01
rs9366778	6	31377152	A/G	*HLA-C | HLA-B*	INTERGENIC	2	0.608	0.515	0.718	**4.44E-09**	0.053	0.033	1.07E-01	0.093	0.123	4.51E-01	-0.123	0.032	**1.40E-04**	0.240	0.119	**4.74E-02**	0.184	0.032	**1.60E-08**	-0.125	0.074	9.30E-02
rs3132671	6	30286266	A/G	*TRIM26*	INTRON	2	1.576	1.353	1.834	**4.52E-09**	0.101	0.033	**2.30E-03**	0.128	0.122	2.98E-01	0.017	0.033	6.00E-01	0.032	0.123	7.95E-01	0.074	0.033	**2.40E-02**	0.105	0.074	1.56E-01
rs356954	6	30091517	G/A	*HCG9 | ZNRD1*	INTERGENIC	2	1.608	1.372	1.885	**4.52E-09**	-0.010	0.034	7.80E-01	-0.052	0.123	6.73E-01	0.167	0.034	**9.00E-07**	-0.041	0.123	7.42E-01	-0.191	0.034	**2.40E-08**	-0.092	0.074	2.14E-01
rs3734528	6	26130223	G/A	*HIST1H4A*	UTR	2	1.850	1.506	2.273	**4.56E-09**	0.012	0.048	7.90E-01	0.026	0.123	8.34E-01	0.103	0.047	**2.80E-02**	-0.113	0.122	3.55E-01	-0.107	0.048	**2.50E-02**	-0.077	0.074	2.98E-01
rs418914	6	28676507	G/A	*ZNF452 | LOC646160*	INTERGENIC	2	1.591	1.362	1.858	**4.56E-09**	0.011	0.034	7.50E-01	-0.075	0.123	5.43E-01	0.043	0.034	2.10E-01	-0.196	0.121	1.09E-01	-0.033	0.034	3.30E-01	-0.044	0.074	5.57E-01
rs9380240	6	31376811	G/A	*HLA-C | HLA-B*	INTERGENIC	2	0.609	0.515	0.718	**4.58E-09**	0.042	0.033	2.00E-01	0.093	0.123	4.51E-01	-0.142	0.033	**1.50E-05**	0.240	0.119	**4.74E-02**	0.196	0.033	**2.90E-09**	-0.125	0.074	9.30E-02
rs3095302	6	31201045	A/G	*CDSN | PSORS1C1*	INTERGENIC	2	0.624	0.532	0.730	**4.70E-09**	0.056	0.032	8.20E-02	0.057	0.124	6.44E-01	-0.180	0.032	**1.20E-08**	0.056	0.123	6.49E-01	0.238	0.032	**7.30E-14**	0.061	0.074	4.15E-01
rs757257	6	30250437	A/G	*TRIM15 | TRIM26*	INTERGENIC	2	1.578	1.355	1.839	**4.83E-09**	0.097	0.033	**3.50E-03**	0.128	0.123	2.98E-01	0.012	0.033	7.00E-01	0.025	0.123	8.38E-01	0.075	0.033	**2.20E-02**	0.110	0.074	1.39E-01
rs3096702	6	32300309	A/G	*NOTCH4 | C6orf10*	INTERGENIC	2	0.617	0.525	0.726	**4.84E-09**	-0.091	0.032	**4.70E-03**	-0.089	0.123	4.71E-01	0.054	0.032	8.70E-02	-0.139	0.121	2.55E-01	-0.136	0.032	**2.00E-05**	-0.033	0.074	6.60E-01
rs3130383	6	30287005	C/A	*TRIM26*	INTRON	2	1.574	1.352	1.832	**4.91E-09**	-	-	-	0.128	0.123	2.98E-01	-	-	-	0.025	0.123	8.38E-01	-	-	-	0.105	0.074	1.55E-01
rs442439	6	28680985	A/G	*ZNF452 | LOC646160*	INTERGENIC	2	1.587	1.359	1.853	**4.93E-09**	0.014	0.034	6.70E-01	-0.038	0.123	7.58E-01	0.058	0.033	8.10E-02	-0.129	0.121	2.92E-01	-0.050	0.034	1.35E-01	-0.057	0.074	4.41E-01
rs1573295	6	30307046	A/G	*TRIM26 | FLJ45422*	INTERGENIC	2	1.577	1.354	1.838	**4.99E-09**	0.103	0.033	**1.80E-03**	0.128	0.122	2.98E-01	0.018	0.033	5.70E-01	0.032	0.123	7.95E-01	0.075	0.033	**2.30E-02**	0.102	0.074	1.68E-01

rs3094140	6	30304292	A/G	*TRIM26 | FLJ45422*	INTERGENIC	2	1.577	1.354	1.838	**4.99E-09**	0.103	0.033	**1.80E-03**	0.128	0.122	2.98E-01	0.018	0.033	5.70E-01	0.032	0.123	7.95E-01	0.075	0.033	**2.30E-02**	0.102	0.074	1.68E-01
rs3129690	6	30304834	G/A	*TRIM26 | FLJ45422*	INTERGENIC	2	1.577	1.354	1.838	**4.99E-09**	0.103	0.033	**1.80E-03**	0.128	0.122	2.98E-01	0.018	0.033	5.70E-01	0.032	0.123	7.95E-01	0.075	0.033	**2.30E-02**	0.102	0.074	1.68E-01
rs3132666	6	30301492	G/A	*TRIM26 | FLJ45422*	INTERGENIC	2	1.577	1.354	1.838	**4.99E-09**	-	-	-	0.128	0.122	2.98E-01	-	-	-	0.032	0.123	7.95E-01	-	-	-	0.102	0.074	1.68E-01
rs916568	6	30310031	A/G	*TRIM26 | FLJ45422*	INTERGENIC	2	1.577	1.354	1.838	**4.99E-09**	0.103	0.033	**1.80E-03**	0.128	0.122	2.98E-01	0.018	0.033	5.70E-01	0.032	0.123	7.95E-01	0.075	0.033	**2.30E-02**	0.102	0.074	1.68E-01
rs3130388	6	30296137	A/C	*TRIM26 | FLJ45422*	INTERGENIC	2	1.577	1.354	1.838	**5.00E-09**	-	-	-	0.128	0.122	2.98E-01	-	-	-	0.032	0.123	7.95E-01	-	-	-	0.102	0.074	1.71E-01
rs418092	6	28641925	A/G	*GPX5 | ZNF452*	INTERGENIC	2	1.589	1.360	1.855	**5.02E-09**	0.011	0.035	7.50E-01	-0.105	0.122	3.94E-01	0.041	0.034	2.30E-01	-0.202	0.120	9.55E-02	-0.031	0.034	3.70E-01	-0.044	0.074	5.52E-01
rs1013518	6	30307491	A/G	*TRIM26 | FLJ45422*	INTERGENIC	2	1.577	1.354	1.838	**5.03E-09**	0.103	0.033	**1.80E-03**	0.128	0.122	2.98E-01	0.018	0.033	5.70E-01	0.032	0.123	7.95E-01	0.075	0.033	**2.30E-02**	0.102	0.074	1.68E-01
rs760804	6	29279018	A/G	*OR2J2 | LOC651503*	INTERGENIC	2	1.629	1.383	1.918	**5.05E-09**	0.043	0.039	2.70E-01	0.001	0.123	9.96E-01	0.094	0.039	**1.60E-02**	-0.179	0.120	1.42E-01	-0.056	0.039	1.50E-01	-0.044	0.074	5.51E-01
rs213228	6	28439231	C/A	*ZKSCAN3*	INTRON	2	1.596	1.364	1.867	**5.07E-09**	-0.011	0.033	7.40E-01	-0.072	0.123	5.57E-01	0.072	0.033	**3.00E-02**	-0.118	0.122	3.34E-01	-0.083	0.033	**1.30E-02**	0.122	0.074	9.89E-02
rs1233704	6	28274902	A/G	*ZNF192 | LOC222699*	INTERGENIC	2	1.624	1.380	1.912	**5.12E-09**	0.079	0.037	**3.60E-02**	0.054	0.124	6.62E-01	0.120	0.037	**1.21E-03**	-0.118	0.123	3.41E-01	-0.049	0.037	1.80E-01	-0.006	0.074	9.37E-01
rs1237875	6	28280989	A/G	*ZNF192 | LOC222699*	INTERGENIC	2	1.624	1.380	1.912	**5.16E-09**	0.079	0.037	**3.60E-02**	0.054	0.124	6.62E-01	0.120	0.037	**1.21E-03**	-0.118	0.123	3.41E-01	-0.049	0.037	1.80E-01	-0.006	0.074	9.37E-01
rs1264537	6	30498178	T/A	*RPP21 | HLA-E*	INTERGENIC	2	1.565	1.347	1.819	**5.19E-09**	-0.053	0.032	9.70E-02	-0.004	0.123	9.76E-01	0.062	0.032	5.30E-02	0.001	0.123	9.93E-01	-0.107	0.032	**8.80E-04**	-0.046	0.074	5.33E-01
rs6910071	6	32390832	G/A	*C6orf10*	INTRON	2	0.519	0.417	0.647	**5.21E-09**	0.048	0.039	2.10E-01	-0.088	0.123	4.73E-01	-0.051	0.038	1.80E-01	-0.120	0.121	3.25E-01	0.091	0.038	**1.70E-02**	0.054	0.074	4.69E-01
rs9268145	6	32365262	C/A	*NOTCH4 | C6orf10*	INTERGENIC	2	0.519	0.417	0.647	**5.24E-09**	0.048	0.039	2.10E-01	-0.088	0.123	4.73E-01	-0.051	0.038	1.80E-01	-0.120	0.121	3.25E-01	0.091	0.038	**1.70E-02**	0.054	0.074	4.69E-01
rs2571377	6	30046550	A/C	*HLA-A | HCG9*	INTERGENIC	1	1.670	1.406	1.984	**5.39E-09**	-0.009	0.034	7.90E-01	-0.031	0.123	8.03E-01	0.137	0.034	**4.70E-05**	-0.044	0.123	7.23E-01	-0.155	0.034	**4.80E-06**	-0.090	0.074	2.24E-01
rs3130393	6	30302510	C/A	*TRIM26 | FLJ45422*	INTERGENIC	2	1.576	1.352	1.836	**5.45E-09**	-	-	-	0.128	0.123	2.98E-01	-	-	-	0.025	0.123	8.38E-01	-	-	-	0.102	0.074	1.67E-01
rs7774730	6	30253719	A/C	*TRIM15 | TRIM26*	INTERGENIC	2	1.572	1.350	1.830	**5.46E-09**	0.097	0.033	**3.50E-03**	0.128	0.123	2.98E-01	0.012	0.033	7.10E-01	0.025	0.123	8.38E-01	0.076	0.033	**2.10E-02**	0.105	0.074	1.55E-01
rs9261539	6	30253062	A/G	*TRIM15 | TRIM26*	INTERGENIC	2	1.572	1.350	1.830	**5.46E-09**	-	-	-	0.128	0.123	2.98E-01	-	-	-	0.025	0.123	8.38E-01	-	-	-	0.105	0.074	1.55E-01
rs4713605	6	33093970	A/T	*HLA-DOA | HLA-DPA1*	INTERGENIC	2	1.559	1.343	1.810	**5.48E-09**	-0.038	0.033	2.50E-01	-0.123	0.122	3.19E-01	-0.028	0.033	3.90E-01	-0.020	0.122	8.72E-01	-0.004	0.033	9.10E-01	0.067	0.074	3.65E-01
rs9267821	6	32277552	G/A	*NOTCH4*	INTRON	2	0.599	0.504	0.711	**5.67E-09**	0.046	0.036	2.00E-01	0.076	0.123	5.36E-01	-0.110	0.036	**2.00E-03**	0.193	0.120	1.12E-01	0.164	0.036	**5.30E-06**	0.054	0.074	4.62E-01
rs3130384	6	30287131	G/A	*TRIM26*	INTRON	2	1.575	1.352	1.835	**5.68E-09**	-	-	-	0.128	0.123	2.98E-01	-	-	-	0.025	0.123	8.38E-01	-	-	-	0.105	0.074	1.55E-01
rs1150724	6	28358215	A/G	*PGBD1*	INTRON	2	1.585	1.357	1.851	**5.72E-09**	-0.020	0.033	5.50E-01	-0.008	0.123	9.47E-01	0.059	0.033	6.90E-02	-0.105	0.122	3.93E-01	-0.080	0.033	**1.50E-02**	0.079	0.074	2.84E-01
rs7752195	6	25527073	A/G	*LRRC16*	INTRON	2	1.972	1.569	2.478	**5.74E-09**	0.090	0.058	1.22E-01	0.045	0.124	7.19E-01	0.149	0.058	**9.60E-03**	-0.077	0.123	5.30E-01	-0.082	0.058	1.60E-01	-0.084	0.074	2.53E-01
rs2284178	6	31540104	A/G	*HCP5*	UTR	2	0.623	0.531	0.730	**5.90E-09**	0.061	0.031	5.00E-02	-0.001	0.124	9.94E-01	-0.216	0.031	**3.20E-12**	0.191	0.121	1.19E-01	0.284	0.031	**8.70E-20**	-0.032	0.074	6.70E-01
rs17587597	6	26094430	A/G	*TRIM38 | HIST1H1A*	INTERGENIC	2	1.870	1.514	2.310	**6.12E-09**	-	-	-	0.049	0.123	6.91E-01	-	-	-	-0.106	0.122	3.85E-01	-	-	-	-0.067	0.074	3.66E-01
rs3094626	6	30431602	G/A	*RPP21 | HLA-E*	INTERGENIC	2	1.569	1.348	1.827	**6.15E-09**	0.028	0.032	3.80E-01	-0.147	0.122	2.31E-01	0.035	0.032	2.70E-01	-0.094	0.122	4.45E-01	0.002	0.032	9.60E-01	0.049	0.074	5.11E-01
rs1150726	6	28351021	A/G	*ZNF187*	INTRON	2	1.583	1.356	1.849	**6.16E-09**	-0.020	0.033	5.40E-01	-0.008	0.123	9.47E-01	0.060	0.033	6.70E-02	-0.105	0.122	3.93E-01	-0.080	0.033	**1.42E-02**	0.079	0.074	2.84E-01
rs10484433	6	26138471	A/C	*HIST1H4B | HIST1H3B*	INTERGENIC	2	1.848	1.502	2.274	**6.25E-09**	0.012	0.048	7.90E-01	0.026	0.123	8.34E-01	0.103	0.047	**2.80E-02**	-0.113	0.122	3.55E-01	-0.107	0.048	**2.50E-02**	-0.077	0.074	2.98E-01
rs9268475	6	32466209	A/G	*C6orf10 | BTNL2*	INTERGENIC	1	0.530	0.428	0.657	**6.30E-09**	-	-	-	-0.016	0.123	8.97E-01	-	-	-	0.156	0.121	2.02E-01	-	-	-	0.039	0.074	5.96E-01
rs3129151	6	29246294	A/G	*OR2J3 | OR2J2*	INTERGENIC	2	1.618	1.376	1.904	**6.33E-09**	0.043	0.039	2.70E-01	0.001	0.123	9.96E-01	0.094	0.039	**1.60E-02**	-0.179	0.120	1.42E-01	-0.056	0.039	1.50E-01	-0.044	0.074	5.51E-01
rs3132672	6	30283826	C/A	*TRIM26*	INTRON	2	1.573	1.350	1.832	**6.33E-09**	0.101	0.033	**2.30E-03**	0.128	0.123	2.98E-01	0.017	0.033	6.00E-01	0.025	0.123	8.38E-01	0.074	0.033	**2.40E-02**	0.105	0.074	1.55E-01
rs2394737	6	30250978	G/A	*TRIM15 | TRIM26*	INTERGENIC	2	1.572	1.350	1.832	**6.40E-09**	-	-	-	0.128	0.123	2.98E-01	-	-	-	0.025	0.123	8.38E-01	-	-	-	0.105	0.074	1.55E-01
rs58432754	6	30284290	G/C	*TRIM26*	INTRON	2	1.572	1.350	1.832	**6.40E-09**	-	-	-	0.128	0.123	2.98E-01	-	-	-	0.025	0.123	8.38E-01	-	-	-	0.105	0.074	1.55E-01
rs9261543	6	30254629	G/A	*TRIM15 | TRIM26*	INTERGENIC	2	1.572	1.349	1.832	**6.43E-09**	-	-	-	0.128	0.123	2.98E-01	-	-	-	0.025	0.123	8.38E-01	-	-	-	0.105	0.074	1.55E-01
rs7776233	6	31092107	A/G	*C6orf205 | LOC729792*	INTERGENIC	2	0.580	0.483	0.697	**6.61E-09**	0.043	0.033	2.00E-01	-0.182	0.121	1.39E-01	-0.117	0.033	**3.70E-04**	-0.043	0.123	7.25E-01	0.157	0.033	**2.10E-06**	-0.059	0.074	4.25E-01
rs9276234	6	32808826	T/A	*HLA-DQB1 | HLA-DQA2*	INTERGENIC	1	0.590	0.494	0.705	**6.68E-09**	-0.035	0.032	2.80E-01	0.262	0.121	**3.42E-02**	0.115	0.032	**3.50E-04**	0.331	0.117	**6.34E-03**	-0.158	0.032	**8.90E-07**	-0.086	0.074	2.49E-01
rs259940	6	30119913	G/A	*HCG9 | ZNRD1*	INTERGENIC	2	1.603	1.366	1.880	**6.95E-09**	-0.010	0.034	7.70E-01	-0.049	0.123	6.93E-01	0.166	0.034	**1.00E-06**	-0.032	0.123	7.92E-01	-0.191	0.034	**2.60E-08**	-0.091	0.074	2.20E-01
rs2857209	6	32849896	A/G	*HLA-DQB2 | HLA-DOB*	INTERGENIC	2	0.600	0.505	0.714	**7.05E-09**	-0.018	0.034	5.90E-01	0.148	0.122	2.29E-01	-0.092	0.033	**5.90E-03**	0.044	0.122	7.22E-01	0.081	0.034	**1.60E-02**	0.027	0.074	7.22E-01
rs9267958	6	32322594	C/A	*NOTCH4 | C6orf10*	INTERGENIC	2	0.611	0.518	0.722	**7.05E-09**	0.063	0.033	5.50E-02	0.023	0.123	8.51E-01	-0.128	0.032	**7.80E-05**	0.102	0.122	4.09E-01	0.194	0.033	**2.70E-09**	0.083	0.074	2.59E-01
rs2076529	6	32471933	G/A	*BTNL2*	CODING	2	0.616	0.522	0.726	**7.06E-09**	0.031	0.032	3.30E-01	-0.027	0.123	8.26E-01	-0.094	0.032	**3.30E-03**	0.098	0.122	4.25E-01	0.129	0.032	**6.30E-05**	0.020	0.074	7.87E-01
rs401618	6	30058189	G/A	*HCG9 | ZNRD1*	INTERGENIC	2	1.593	1.361	1.866	**7.09E-09**	-0.011	0.035	7.60E-01	-0.014	0.123	9.13E-01	0.170	0.034	**6.50E-07**	-0.041	0.123	7.38E-01	-0.195	0.034	**1.40E-08**	-0.082	0.074	2.68E-01
rs3817973	6	32469089	A/G	*C6orf10 | BTNL2*	INTERGENIC	2	0.616	0.523	0.726	**7.15E-09**	0.031	0.032	3.30E-01	-0.027	0.123	8.26E-01	-0.094	0.032	**3.30E-03**	0.098	0.122	4.25E-01	0.129	0.032	**6.30E-05**	0.020	0.074	7.87E-01
rs9268472	6	32463583	A/G	*C6orf10 | BTNL2*	INTERGENIC	2	0.616	0.523	0.726	**7.15E-09**	-	-	-	-0.027	0.123	8.26E-01	-	-	-	0.098	0.122	4.25E-01	-	-	-	0.020	0.074	7.87E-01
rs4373382	6	32458846	C/A	*C6orf10 | BTNL2*	INTERGENIC	2	0.616	0.523	0.726	**7.16E-09**	-	-	-	-0.027	0.123	8.26E-01	-	-	-	0.098	0.122	4.25E-01	-	-	-	0.021	0.074	7.78E-01
rs2735067	6	30059085	G/A	*HCG9 | ZNRD1*	INTERGENIC	2	1.593	1.361	1.866	**7.17E-09**	-0.011	0.035	7.60E-01	-0.014	0.123	9.13E-01	0.170	0.034	**6.50E-07**	-0.041	0.123	7.38E-01	-0.195	0.034	**1.40E-08**	-0.082	0.074	2.68E-01
rs2395300	6	33076254	A/G	*BRD2 | HLA-DOA*	INTERGENIC	2	1.579	1.353	1.844	**7.37E-09**	-0.029	0.034	4.00E-01	0.006	0.123	9.63E-01	-0.001	0.034	9.80E-01	0.029	0.122	8.12E-01	-0.025	0.034	4.70E-01	0.009	0.074	9.08E-01
rs12216336	6	33075719	C/G	*BRD2 | HLA-DOA*	INTERGENIC	2	1.569	1.347	1.828	**7.60E-09**	-0.029	0.034	4.00E-01	0.006	0.123	9.63E-01	-0.001	0.034	9.80E-01	0.029	0.122	8.12E-01	-0.025	0.034	4.70E-01	0.009	0.074	9.08E-01
rs259943	6	30123309	G/A	*HCG9 | ZNRD1*	INTERGENIC	2	1.601	1.365	1.878	**7.60E-09**	-	-	-	-0.052	0.123	6.73E-01	-	-	-	-0.041	0.123	7.42E-01	-	-	-	-0.092	0.074	2.14E-01
rs1150742	6	30136810	G/A	*HCG9 | ZNRD1*	INTERGENIC	2	1.600	1.364	1.877	**7.76E-09**	-0.010	0.034	7.70E-01	-0.052	0.123	6.73E-01	0.166	0.034	**1.00E-06**	-0.041	0.123	7.42E-01	-0.191	0.034	**2.60E-08**	-0.092	0.074	2.14E-01
rs259931	6	30112350	T/A	*HCG9 | ZNRD1*	INTERGENIC	2	1.600	1.364	1.877	**7.76E-09**	-0.010	0.034	7.80E-01	-0.052	0.123	6.73E-01	0.167	0.034	**9.00E-07**	-0.041	0.123	7.42E-01	-0.191	0.034	**2.40E-08**	-0.092	0.074	2.14E-01
rs2844802	6	30056791	A/T	*HCG9 | ZNRD1*	INTERGENIC	2	1.600	1.364	1.877	**7.76E-09**	-0.011	0.035	7.60E-01	-0.014	0.123	9.13E-01	0.170	0.034	**6.50E-07**	-0.041	0.123	7.38E-01	-0.195	0.034	**1.40E-08**	-0.082	0.074	2.68E-01
rs9267853	6	32303229	A/G	*NOTCH4 | C6orf10*	INTERGENIC	2	0.625	0.533	0.733	**7.76E-09**	-0.091	0.032	**4.70E-03**	-0.089	0.123	4.71E-01	0.054	0.032	8.70E-02	-0.139	0.121	2.55E-01	-0.136	0.032	**2.00E-05**	-0.038	0.074	6.04E-01
rs4713604	6	33087748	C/G	*HLA-DOA | HLA-DPA1*	INTERGENIC	2	1.561	1.342	1.816	**7.93E-09**	-0.038	0.033	2.40E-01	-0.123	0.122	3.19E-01	-0.027	0.033	4.00E-01	-0.020	0.122	8.72E-01	-0.004	0.033	9.00E-01	0.067	0.074	3.65E-01
rs954037	6	30311581	A/G	*TRIM26 | FLJ45422*	INTERGENIC	2	1.577	1.351	1.841	**8.02E-09**	0.103	0.033	**1.80E-03**	0.128	0.123	2.98E-01	0.018	0.033	5.70E-01	0.025	0.123	8.38E-01	0.075	0.033	**2.30E-02**	0.104	0.074	1.61E-01
rs2844472	6	31697655	G/A	*BAT2*	INTRON	2	0.595	0.499	0.710	**8.13E-09**	0.070	0.034	**4.10E-02**	0.085	0.123	4.92E-01	-0.105	0.034	**1.80E-03**	-0.084	0.122	4.95E-01	0.163	0.034	**1.40E-06**	0.062	0.074	4.01E-01
rs2859112	6	32805991	A/G	*HLA-DQB1 | HLA-DQA2*	INTERGENIC	2	0.609	0.515	0.721	**8.37E-09**	0.020	0.033	5.30E-01	0.267	0.119	**2.76E-02**	-0.061	0.032	5.90E-02	0.155	0.121	2.04E-01	0.087	0.032	**7.60E-03**	-0.045	0.074	5.40E-01
rs9393777	6	27050006	G/A	*LOC100133205*	INTRON	1	1.850	1.501	2.281	**8.44E-09**	0.094	0.045	**3.70E-02**	0.059	0.124	6.39E-01	0.212	0.044	**1.80E-06**	-0.079	0.123	5.23E-01	-0.144	0.045	**1.35E-03**	-0.027	0.075	7.22E-01
rs9461799	6	32797507	G/A	*HLA-DQB1 | HLA-DQA2*	INTERGENIC	2	0.609	0.515	0.721	**8.45E-09**	0.020	0.033	5.30E-01	0.267	0.119	**2.76E-02**	-0.061	0.032	5.90E-02	0.155	0.121	2.04E-01	0.087	0.032	**7.60E-03**	-0.045	0.074	5.40E-01
rs7755802	6	31090188	G/A	*C6orf205 | LOC729792*	INTERGENIC	2	0.616	0.522	0.726	**8.53E-09**	0.050	0.032	1.16E-01	-0.138	0.122	2.60E-01	-0.102	0.032	**1.28E-03**	-0.054	0.122	6.62E-01	0.148	0.032	**3.90E-06**	-0.063	0.074	3.94E-01
rs707939	6	31834667	A/C	*MSH5*	INTRON	2	0.594	0.498	0.709	**8.58E-09**	0.014	0.034	6.90E-01	0.050	0.123	6.82E-01	-0.084	0.034	**1.32E-02**	-0.078	0.122	5.23E-01	0.102	0.034	**2.80E-03**	0.041	0.074	5.85E-01
rs9267873	6	32307330	G/A	*NOTCH4 | C6orf10*	INTERGENIC	2	0.622	0.529	0.731	**8.99E-09**	-0.097	0.032	**2.60E-03**	-0.087	0.123	4.78E-01	0.072	0.032	**2.30E-02**	-0.191	0.120	1.17E-01	-0.162	0.032	**4.20E-07**	-0.015	0.074	8.36E-01
rs3132668	6	30295143	T/A	*TRIM26 | FLJ45422*	INTERGENIC	2	1.569	1.346	1.830	**9.06E-09**	0.103	0.033	**1.80E-03**	0.128	0.122	2.98E-01	0.018	0.033	5.70E-01	0.032	0.123	7.95E-01	0.075	0.033	**2.30E-02**	0.102	0.074	1.68E-01
rs2040486	6	30302082	C/A	*TRIM26 | FLJ45422*	INTERGENIC	2	1.569	1.345	1.829	**9.10E-09**	0.103	0.033	**1.80E-03**	0.128	0.122	2.98E-01	0.018	0.033	5.70E-01	0.032	0.123	7.95E-01	0.075	0.033	**2.30E-02**	0.102	0.074	1.68E-01
rs3132667	6	30299566	G/A	*TRIM26 | FLJ45422*	INTERGENIC	2	1.569	1.345	1.829	**9.10E-09**	0.103	0.033	**1.80E-03**	0.128	0.122	2.98E-01	0.018	0.033	5.70E-01	0.032	0.123	7.95E-01	0.075	0.033	**2.30E-02**	0.102	0.074	1.68E-01
rs9267810	6	32229572	G/A	*LOC100130536*	CODING	1	0.540	0.438	0.666	**9.24E-09**	-	-	-	0.024	0.123	8.48E-01	-	-	-	0.155	0.121	2.05E-01	-	-	-	0.045	0.074	5.45E-01
rs3094614	6	30289254	G/A	*TRIM26 | FLJ45422*	INTERGENIC	2	1.568	1.345	1.829	**9.30E-09**	0.101	0.033	**2.30E-03**	0.128	0.122	2.98E-01	0.017	0.033	6.00E-01	0.032	0.123	7.95E-01	0.074	0.033	**2.40E-02**	0.105	0.074	1.56E-01
rs1339898	6	28503485	A/G	*ZSCAN23*	INTRON	2	1.576	1.349	1.840	**9.31E-09**	-0.009	0.033	7.80E-01	-0.072	0.123	5.57E-01	0.071	0.033	**3.10E-02**	-0.118	0.122	3.34E-01	-0.081	0.033	**1.50E-02**	0.122	0.074	9.89E-02
rs1233701	6	28276705	C/G	*ZNF192 | LOC222699*	INTERGENIC	2	1.606	1.366	1.888	**9.37E-09**	0.079	0.037	**3.60E-02**	0.054	0.124	6.62E-01	0.120	0.037	**1.21E-03**	-0.118	0.123	3.41E-01	-0.049	0.037	1.80E-01	-0.008	0.075	9.19E-01
rs2859100	6	32807457	C/G	*HLA-DQB1 | HLA-DQA2*	INTERGENIC	2	0.614	0.520	0.725	**9.44E-09**	0.025	0.033	4.40E-01	0.254	0.119	**3.73E-02**	-0.059	0.032	6.80E-02	0.141	0.121	2.50E-01	0.088	0.032	**6.40E-03**	-0.045	0.074	5.40E-01

rs1265156	6	31250276	A/C	*POU5F1 | LOC100130889*	INTERGENIC	1	0.570	0.470	0.691	**9.53E-09**	0.076	0.035	**3.10E-02**	0.096	0.123	4.38E-01	-0.125	0.035	**3.20E-04**	0.172	0.121	1.58E-01	0.194	0.035	**2.70E-08**	-0.040	0.074	5.90E-01
rs9276427	6	32819835	G/A	*HLA-DQA2*	INTRON	2	0.624	0.531	0.733	**9.55E-09**	-	-	-	0.151	0.122	2.20E-01	-	-	-	0.188	0.120	1.22E-01	-	-	-	-0.110	0.074	1.37E-01
rs2071550	6	32838918	A/C	*HLA-DQB2*	INTRON	2	0.594	0.498	0.710	**9.59E-09**	0.022	0.036	5.50E-01	0.209	0.120	8.75E-02	-0.035	0.036	3.40E-01	0.240	0.119	**4.70E-02**	0.063	0.036	8.10E-02	-0.072	0.074	3.33E-01
rs2071473	6	32890583	A/G	*HLA-DOB*	INTRON	2	0.618	0.524	0.729	**9.85E-09**	-0.010	0.034	7.60E-01	0.131	0.122	2.87E-01	-0.098	0.033	**3.30E-03**	0.027	0.122	8.26E-01	0.096	0.034	**4.50E-03**	0.016	0.074	8.30E-01
rs16891235	6	26125521	G/A	*HIST1H1A*	CODING	2	1.826	1.486	2.244	**9.88E-09**	0.010	0.048	8.40E-01	-0.007	0.123	9.53E-01	0.103	0.048	**3.20E-02**	-0.120	0.122	3.29E-01	-0.109	0.048	**2.40E-02**	-0.077	0.074	2.99E-01
rs3129152	6	29246756	C/A	*OR2J3 | OR2J2*	INTERGENIC	2	1.615	1.371	1.902	**9.91E-09**	0.043	0.039	2.70E-01	-0.016	0.123	8.95E-01	0.094	0.039	**1.60E-02**	-0.190	0.120	1.19E-01	-0.056	0.039	1.50E-01	-0.043	0.074	5.61E-01
rs2516677	6	30532602	C/A	*RPP21 | HLA-E*	INTERGENIC	2	1.561	1.340	1.818	**1.06E-08**	-0.055	0.032	8.70E-02	-0.019	0.123	8.81E-01	0.055	0.032	8.20E-02	-0.001	0.123	9.93E-01	-0.101	0.032	**1.60E-03**	-0.063	0.074	3.91E-01
rs2524179	6	30534131	G/A	*RPP21 | HLA-E*	INTERGENIC	2	1.561	1.340	1.818	**1.06E-08**	-	-	-	-0.019	0.123	8.81E-01	-	-	-	-0.001	0.123	9.93E-01	-	-	-	-0.063	0.074	3.91E-01
rs2524182	6	30533922	A/T	*RPP21 | HLA-E*	INTERGENIC	2	1.561	1.340	1.818	**1.06E-08**	-0.055	0.032	8.50E-02	-0.019	0.123	8.81E-01	0.055	0.032	8.00E-02	-0.001	0.123	9.93E-01	-0.102	0.032	**1.46E-03**	-0.063	0.074	3.91E-01
rs2395163	6	32495787	G/A	*BTNL2 | HLA-DRA*	INTERGENIC	2	0.529	0.425	0.658	**1.08E-08**	0.041	0.039	2.90E-01	0.001	0.124	9.93E-01	-0.088	0.038	**2.10E-02**	-0.012	0.123	9.22E-01	0.129	0.039	**8.20E-04**	0.045	0.074	5.43E-01
rs6457327	6	31182009	A/C	*HCG22 | C6orf15*	INTERGENIC	2	0.620	0.526	0.731	**1.09E-08**	0.027	0.033	4.20E-01	0.162	0.121	1.88E-01	0.031	0.033	3.50E-01	-0.069	0.122	5.76E-01	-0.019	0.033	5.60E-01	0.032	0.074	6.70E-01
rs3095301	6	31201335	G/A	*CDSN | PSORS1C1*	INTERGENIC	2	0.618	0.523	0.729	**1.10E-08**	-	-	-	0.066	0.123	5.94E-01	-	-	-	0.064	0.122	6.00E-01	-	-	-	0.061	0.074	4.15E-01
rs3887152	6	31284314	A/G	*HCG27 | HLA-C*	INTERGENIC	2	0.633	0.541	0.740	**1.13E-08**	-	-	-	0.003	0.124	9.82E-01	-	-	-	0.110	0.122	3.70E-01	-	-	-	-0.015	0.074	8.41E-01
rs9263872	6	31278587	A/G	*HCG27*	UTR	2	0.625	0.532	0.735	**1.13E-08**	0.046	0.032	1.60E-01	-0.043	0.125	7.33E-01	-0.123	0.032	**1.20E-04**	0.102	0.124	4.11E-01	0.170	0.032	**1.20E-07**	-0.085	0.074	2.53E-01
rs2517448	6	31170646	A/G	*HCG22 | C6orf15*	INTERGENIC	2	0.621	0.527	0.731	**1.15E-08**	0.027	0.033	4.20E-01	0.162	0.121	1.88E-01	0.031	0.033	3.50E-01	-0.069	0.122	5.76E-01	-0.019	0.033	5.60E-01	0.032	0.074	6.63E-01
rs2763982	6	31980530	C/G	*ZBTB12 | C2*	INTERGENIC	2	0.604	0.508	0.718	**1.15E-08**	0.076	0.034	**2.70E-02**	0.055	0.123	6.58E-01	-0.108	0.034	**1.43E-03**	0.060	0.122	6.24E-01	0.179	0.034	**1.60E-07**	0.062	0.074	4.01E-01
rs1269556	6	30516766	A/G	*RPP21 | HLA-E*	INTERGENIC	2	1.547	1.332	1.797	**1.16E-08**	-0.061	0.032	6.00E-02	-0.032	0.124	7.98E-01	0.060	0.032	5.80E-02	0.001	0.123	9.92E-01	-0.112	0.032	**4.90E-04**	-0.037	0.074	6.21E-01
rs9263873	6	31278692	G/A	*HCG27*	UTR	2	0.626	0.532	0.735	**1.16E-08**	0.046	0.032	1.60E-01	-0.043	0.125	7.33E-01	-0.123	0.032	**1.20E-04**	0.102	0.124	4.11E-01	0.170	0.032	**1.20E-07**	-0.085	0.074	2.53E-01
rs1619379	6	29893214	A/G	*HCG4 | HLA-G*	INTERGENIC	2	1.562	1.340	1.821	**1.17E-08**	-0.017	0.032	6.00E-01	-0.078	0.124	5.32E-01	0.050	0.032	1.20E-01	-0.137	0.122	2.66E-01	-0.056	0.032	8.20E-02	0.033	0.074	6.53E-01
rs1052248	6	31664560	A/T	*LST1 / NCR3*	UTR	2	0.560	0.459	0.684	**1.19E-08**	-0.019	0.036	6.10E-01	0.115	0.123	3.53E-01	-0.142	0.036	**8.50E-05**	0.113	0.122	3.58E-01	0.135	0.036	**1.90E-04**	-0.097	0.075	2.01E-01
rs356968	6	30083026	A/C	*HCG9 | ZNRD1*	INTERGENIC	2	1.599	1.361	1.879	**1.20E-08**	-0.014	0.035	6.90E-01	-0.052	0.123	6.73E-01	0.162	0.034	**2.30E-06**	-0.041	0.123	7.42E-01	-0.189	0.035	**4.40E-08**	-0.092	0.074	2.14E-01
rs2844477	6	31686751	G/A	*NCR3 | AIF1*	INTERGENIC	2	0.599	0.502	0.714	**1.23E-08**	0.073	0.034	**3.30E-02**	0.085	0.123	4.92E-01	-0.108	0.034	**1.32E-03**	-0.084	0.122	4.95E-01	0.169	0.034	**5.70E-07**	0.062	0.074	4.01E-01
rs2524201	6	30523883	G/A	*RPP21 | HLA-E*	INTERGENIC	2	1.560	1.338	1.818	**1.29E-08**	-	-	-	0.007	0.124	9.56E-01	-	-	-	0.002	0.123	9.89E-01	-	-	-	-0.041	0.074	5.80E-01
rs13220395	6	26163347	A/G	*HIST1H3C | HIST1H1C*	INTERGENIC	2	1.838	1.490	2.267	**1.30E-08**	0.033	0.049	5.10E-01	0.049	0.123	6.91E-01	0.153	0.049	**1.70E-03**	-0.106	0.122	3.85E-01	-0.141	0.049	**4.10E-03**	-0.067	0.074	3.66E-01
rs2621412	6	32850978	C/A	*HLA-DQB2 | HLA-DOB*	INTERGENIC	2	0.611	0.515	0.724	**1.30E-08**	-0.018	0.034	5.90E-01	0.148	0.122	2.29E-01	-0.092	0.033	**5.90E-03**	0.044	0.122	7.22E-01	0.081	0.034	**1.60E-02**	0.024	0.074	7.42E-01
rs2524181	6	30534082	T/A	*RPP21 | HLA-E*	INTERGENIC	2	1.545	1.330	1.795	**1.31E-08**	-0.066	0.032	**3.90E-02**	-0.019	0.123	8.81E-01	0.050	0.032	1.14E-01	-0.001	0.123	9.93E-01	-0.106	0.032	**9.30E-04**	-0.061	0.074	4.13E-01
rs2260050	6	31699907	G/A	*BAT2*	INTRON	2	0.604	0.508	0.719	**1.35E-08**	-	-	-	0.085	0.123	4.92E-01	-	-	-	-0.084	0.122	4.95E-01	-	-	-	0.062	0.074	4.01E-01
rs2531804	6	28519282	G/A	*ZSCAN23 | COX11P*	INTERGENIC	2	1.541	1.327	1.789	**1.36E-08**	-0.027	0.032	4.00E-01	-0.006	0.123	9.61E-01	0.115	0.032	**2.90E-04**	-0.153	0.121	2.10E-01	-0.147	0.032	**4.40E-06**	0.135	0.073	6.68E-02
rs3130955	6	31162490	A/C	*HCG22 | C6orf15*	INTERGENIC	2	1.546	1.330	1.797	**1.36E-08**	0.059	0.033	7.70E-02	-0.006	0.123	9.60E-01	0.041	0.033	2.10E-01	-0.120	0.121	3.24E-01	0.006	0.033	8.60E-01	0.092	0.074	2.16E-01
rs16891264	6	26180424	G/A	*HIST1H1C | HFE*	INTERGENIC	2	1.836	1.489	2.265	**1.37E-08**	0.033	0.049	5.10E-01	0.049	0.123	6.91E-01	0.153	0.049	**1.70E-03**	-0.106	0.122	3.85E-01	-0.141	0.049	**4.10E-03**	-0.067	0.074	3.66E-01
rs9262499	6	31095077	A/C	*C6orf205 | LOC729792*	INTERGENIC	2	0.624	0.530	0.735	**1.43E-08**	0.035	0.032	2.80E-01	-0.138	0.122	2.60E-01	-0.103	0.032	**1.30E-03**	-0.054	0.122	6.62E-01	0.134	0.032	**2.90E-05**	-0.063	0.074	3.94E-01
rs2736171	6	31703466	G/A	*BAT2*	INTRON	2	0.610	0.514	0.723	**1.44E-08**	0.056	0.034	9.30E-02	0.096	0.123	4.41E-01	-0.110	0.033	**9.50E-04**	-0.104	0.123	3.98E-01	0.156	0.033	**3.00E-06**	0.051	0.074	4.95E-01
rs7758976	6	31095765	G/A	*C6orf205 | LOC729792*	INTERGENIC	2	0.624	0.530	0.735	**1.44E-08**	0.048	0.032	1.36E-01	-0.138	0.122	2.60E-01	-0.101	0.032	**1.60E-03**	-0.054	0.122	6.62E-01	0.144	0.032	**7.00E-06**	-0.063	0.074	3.94E-01
rs2858331	6	32789255	G/A	*HLA-DQB1 | HLA-DQA2*	INTERGENIC	2	0.593	0.494	0.710	**1.49E-08**	0.066	0.033	**4.70E-02**	-0.044	0.123	7.19E-01	-0.074	0.033	**2.40E-02**	0.237	0.119	5.01E-02	0.138	0.033	**3.40E-05**	-0.035	0.074	6.38E-01
rs241429	6	32911818	A/G	*TAP2*	INTRON	2	1.547	1.330	1.799	**1.56E-08**	-0.018	0.032	5.60E-01	-0.016	0.123	8.95E-01	0.065	0.031	**3.80E-02**	0.168	0.121	1.68E-01	-0.093	0.031	**3.20E-03**	0.034	0.074	6.49E-01
rs3131003	6	31201461	A/G	*PSORS1C1*	UTR	2	0.621	0.527	0.733	**1.61E-08**	0.056	0.032	8.20E-02	0.066	0.123	5.94E-01	-0.180	0.032	**1.20E-08**	0.064	0.122	6.00E-01	0.238	0.032	**7.30E-14**	0.061	0.074	4.15E-01
rs2596473	6	31538778	A/C	*LOC100129668 | HCP5*	INTERGENIC	2	0.632	0.539	0.741	**1.66E-08**	0.061	0.031	5.00E-02	-0.001	0.124	9.94E-01	-0.216	0.031	**3.20E-12**	0.191	0.121	1.19E-01	0.284	0.031	**8.70E-20**	-0.028	0.074	7.03E-01
rs5025825	6	33133802	A/C	*HLA-DOA | HLA-DPA1*	INTERGENIC	2	1.566	1.340	1.830	**1.68E-08**	-	-	-	0.001	0.124	9.93E-01	-	-	-	-0.086	0.123	4.85E-01	-	-	-	0.063	0.074	3.95E-01
rs6457709	6	33133931	G/A	*HLA-DOA | HLA-DPA1*	INTERGENIC	2	1.566	1.340	1.830	**1.68E-08**	-	-	-	0.001	0.124	9.93E-01	-	-	-	-0.086	0.123	4.85E-01	-	-	-	0.063	0.074	3.95E-01
rs6899389	6	28489119	A/C	*ZSCAN23*	INTRON	2	1.576	1.345	1.846	**1.70E-08**	-	-	-	-0.055	0.123	6.58E-01	-	-	-	-0.107	0.122	3.82E-01	-	-	-	0.143	0.074	5.29E-02
rs356969	6	30085124	G/C	*HCG9 | ZNRD1*	INTERGENIC	1	1.670	1.397	1.996	**1.74E-08**	-0.010	0.034	7.80E-01	-0.052	0.123	6.73E-01	0.167	0.034	**9.00E-07**	-0.041	0.123	7.42E-01	-0.191	0.034	**2.40E-08**	-0.092	0.074	2.14E-01
rs2524099	6	31344030	A/G	*HCG27 | HLA-C*	INTERGENIC	2	0.641	0.549	0.748	**1.83E-08**	0.065	0.032	**4.20E-02**	0.115	0.122	3.49E-01	-0.179	0.032	**1.40E-08**	0.264	0.118	**2.88E-02**	0.248	0.032	**7.30E-15**	-0.079	0.074	2.85E-01
rs4713447	6	31270942	G/A	*LOC100130889 | HCG27*	INTERGENIC	2	0.634	0.541	0.743	**1.86E-08**	0.037	0.032	2.60E-01	-0.045	0.126	7.22E-01	-0.125	0.032	**9.50E-05**	0.129	0.124	3.03E-01	0.165	0.032	**3.00E-07**	-0.095	0.074	2.02E-01
rs9366768	6	31276473	A/G	*HCG27*	INTRON	2	0.634	0.541	0.743	**1.86E-08**	-	-	-	-0.045	0.126	7.22E-01	-	-	-	0.129	0.124	3.03E-01	-	-	-	-0.095	0.074	2.02E-01
rs9501077	6	31275491	G/A	*HCG27*	INTRON	2	0.634	0.541	0.743	**1.86E-08**	-0.046	0.038	2.30E-01	-0.045	0.126	7.22E-01	-0.093	0.037	**1.31E-02**	0.129	0.124	3.03E-01	0.059	0.038	1.20E-01	-0.095	0.074	2.02E-01
rs2075800	6	31885925	A/G	*HSPA1L*	CODING	2	0.602	0.504	0.718	**1.89E-08**	0.033	0.034	3.40E-01	0.095	0.122	4.42E-01	-0.066	0.034	5.40E-02	-0.056	0.122	6.49E-01	0.098	0.034	**4.50E-03**	0.062	0.074	4.04E-01
rs6909636	6	31454578	A/G	*LOC729816 | LOC100129668*	INTERGENIC	2	0.628	0.534	0.739	**1.91E-08**	0.040	0.033	2.30E-01	0.035	0.123	7.75E-01	-0.121	0.033	**2.00E-04**	-0.003	0.122	9.82E-01	0.161	0.033	**8.40E-07**	-0.028	0.074	7.08E-01
rs2894176	6	31094017	C/G	*C6orf205 | LOC729792*	INTERGENIC	2	0.623	0.528	0.734	**1.92E-08**	0.039	0.032	2.30E-01	-0.138	0.122	2.60E-01	-0.112	0.032	**4.90E-04**	-0.054	0.122	6.62E-01	0.147	0.032	**5.10E-06**	-0.064	0.074	3.85E-01
rs3135338	6	32509195	G/A	*BTNL2 | HLA-DRA*	INTERGENIC	2	0.599	0.501	0.716	**1.93E-08**	-0.073	0.034	**3.20E-02**	0.068	0.123	5.80E-01	0.038	0.034	2.60E-01	0.181	0.120	1.37E-01	-0.099	0.034	**3.50E-03**	-0.116	0.073	1.17E-01
rs3135395	6	32513170	A/C	*BTNL2 | HLA-DRA*	INTERGENIC	2	0.599	0.501	0.716	**1.93E-08**	-0.073	0.034	**3.20E-02**	0.068	0.123	5.80E-01	0.038	0.034	2.60E-01	0.181	0.120	1.37E-01	-0.099	0.034	**3.50E-03**	-0.116	0.073	1.17E-01
rs3819721	6	32912776	A/G	*TAP2*	INTRON	2	0.574	0.473	0.697	**1.93E-08**	0.037	0.036	3.10E-01	0.211	0.123	9.24E-02	-0.044	0.036	2.20E-01	-0.023	0.125	8.53E-01	0.074	0.036	**4.10E-02**	-0.045	0.074	5.45E-01
rs9461458	6	28463854	A/G	*ZKSCAN3 | ZSCAN12*	INTERGENIC	1	1.660	1.391	1.981	**1.96E-08**	-0.005	0.033	8.90E-01	-0.055	0.123	6.58E-01	0.087	0.033	**8.70E-03**	-0.107	0.122	3.82E-01	-0.093	0.033	**5.30E-03**	0.111	0.074	1.34E-01
rs2857697	6	31693198	A/G	*AIF1 | BAT2*	INTERGENIC	2	0.613	0.516	0.727	**2.07E-08**	0.056	0.034	9.30E-02	0.096	0.123	4.41E-01	-0.110	0.033	**9.50E-04**	-0.104	0.123	3.98E-01	0.156	0.033	**3.00E-06**	0.051	0.074	4.95E-01
rs3094132	6	30295413	G/C	*TRIM26 | FLJ45422*	INTERGENIC	2	1.540	1.324	1.791	**2.15E-08**	0.103	0.033	**1.80E-03**	0.128	0.122	2.98E-01	0.018	0.033	5.70E-01	0.032	0.123	7.95E-01	0.075	0.033	**2.30E-02**	0.101	0.074	1.71E-01
rs805262	6	31736712	A/G	*C6orf47 | BAT4*	INTERGENIC	2	1.538	1.323	1.788	**2.19E-08**	0.083	0.032	**1.06E-02**	0.021	0.123	8.67E-01	0.030	0.032	3.50E-01	-0.196	0.120	1.08E-01	0.037	0.032	2.50E-01	0.058	0.074	4.31E-01
rs406477	6	33113622	G/A	*HLA-DOA | HLA-DPA1*	INTERGENIC	2	1.678	1.400	2.012	**2.24E-08**	0.013	0.047	7.80E-01	-0.006	0.124	9.58E-01	0.015	0.046	7.50E-01	0.131	0.122	2.88E-01	-0.001	0.047	9.90E-01	-0.061	0.074	4.13E-01
rs6457372	6	31355100	A/G	*HLA-C | HLA-B*	INTERGENIC	2	0.630	0.536	0.741	**2.27E-08**	0.000	0.033	9.90E-01	0.139	0.122	2.61E-01	0.022	0.032	4.90E-01	0.118	0.122	3.37E-01	-0.040	0.032	2.20E-01	0.112	0.074	1.31E-01
rs9368741	6	32845485	A/G	*HLA-DQB2 | HLA-DOB*	INTERGENIC	2	0.602	0.504	0.720	**2.27E-08**	0.022	0.036	5.50E-01	0.209	0.120	8.75E-02	-0.034	0.036	3.40E-01	0.240	0.119	**4.70E-02**	0.063	0.036	8.50E-02	-0.072	0.074	3.33E-01
rs9467632	6	25997685	G/A	*SLC17A3 | SLC17A2*	INTERGENIC	2	1.834	1.482	2.269	**2.34E-08**	0.041	0.051	4.10E-01	0.025	0.123	8.43E-01	0.165	0.050	**9.50E-04**	-0.124	0.121	3.09E-01	-0.147	0.050	**3.60E-03**	-0.068	0.074	3.61E-01
rs396660	6	29754144	A/G	*ZFP57*	INTRON	2	1.574	1.342	1.846	**2.42E-08**	-0.004	0.034	9.00E-01	-0.029	0.123	8.17E-01	0.071	0.033	**3.50E-02**	-0.094	0.122	4.44E-01	-0.074	0.034	**2.80E-02**	-0.052	0.074	4.84E-01
rs445150	6	29754858	G/A	*ZFP57*	INTRON	2	1.574	1.342	1.846	**2.44E-08**	-0.004	0.034	9.00E-01	-0.029	0.123	8.17E-01	0.071	0.033	**3.50E-02**	-0.094	0.122	4.44E-01	-0.074	0.034	**2.80E-02**	-0.052	0.074	4.84E-01
rs172274	6	33077435	A/G	*BRD2 | HLA-DOA*	INTERGENIC	2	1.549	1.328	1.806	**2.45E-08**	-0.021	0.034	5.30E-01	-0.015	0.123	9.00E-01	0.007	0.034	8.50E-01	0.129	0.122	2.92E-01	-0.026	0.034	4.50E-01	0.002	0.075	9.81E-01
rs2532929	6	31005753	G/A	*VARS2 | SFTPG*	INTERGENIC	2	0.629	0.534	0.740	**2.55E-08**	-0.003	0.033	9.30E-01	-0.113	0.123	3.59E-01	-0.068	0.033	**4.00E-02**	-0.024	0.123	8.46E-01	0.076	0.033	**2.30E-02**	-0.013	0.074	8.60E-01
rs29228	6	29731718	A/G	*GABBR1 | MOG*	INTERGENIC	1	1.720	1.421	2.082	**2.56E-08**	0.007	0.039	8.50E-01	-0.018	0.123	8.82E-01	0.143	0.039	**2.00E-04**	-0.039	0.122	7.53E-01	-0.153	0.039	**8.30E-05**	-0.028	0.075	7.06E-01
rs7773051	6	28448295	A/G	*ZKSCAN3 | ZSCAN12*	INTERGENIC	2	1.566	1.337	1.835	**2.61E-08**	-0.005	0.033	8.90E-01	-0.055	0.123	6.58E-01	0.087	0.033	**8.70E-03**	-0.107	0.122	3.82E-01	-0.093	0.033	**5.30E-03**	0.122	0.074	1.00E-01
rs3799378	6	26512353	G/A	*BTN3A1*	INTRON	1	1.700	1.410	2.050	**2.73E-08**	0.009	0.037	8.20E-01	-0.024	0.126	8.51E-01	0.098	0.037	**8.20E-03**	-0.155	0.124	2.14E-01	-0.098	0.037	**8.10E-03**	-0.058	0.075	4.40E-01
rs7764722	6	28462512	A/G	*ZKSCAN3 | ZSCAN12*	INTERGENIC	2	1.560	1.334	1.825	**2.77E-08**	-0.005	0.033	8.90E-01	-0.055	0.123	6.58E-01	0.087	0.033	**8.70E-03**	-0.107	0.122	3.82E-01	-0.093	0.033	**5.30E-03**	0.122	0.074	9.91E-02
rs6938200	6	27339129	G/A	*PRSS16 | POM121L2*	INTERGENIC	2	1.644	1.379	1.959	**2.82E-08**	0.010	0.038	7.90E-01	0.107	0.123	3.86E-01	0.104	0.038	**5.50E-03**	-0.077	0.122	5.33E-01	-0.094	0.038	**1.30E-02**	0.050	0.075	5.08E-01
rs9468370	6	28476919	A/G	*ZSCAN12 | ZSCAN23*	INTERGENIC	2	1.560	1.333	1.825	**2.83E-08**	-0.006	0.033	8.50E-01	-0.055	0.123	6.58E-01	0.086	0.033	**9.80E-03**	-0.107	0.122	3.82E-01	-0.093	0.033	**5.40E-03**	0.122	0.074	9.91E-02

rs1131896	6	31487094	A/G	*LOC100129668*	CODING	2	0.557	0.453	0.685	**2.86E-08**	0.043	0.038	2.60E-01	-0.049	0.123	6.92E-01	-0.168	0.038	**9.00E-06**	0.135	0.121	2.71E-01	0.224	0.038	**4.10E-09**	0.029	0.074	6.94E-01
rs1383264	6	32847945	T/A	*HLA-DQB2 | HLA-DOB*	INTERGENIC	2	1.539	1.322	1.793	**2.96E-08**	-0.009	0.033	7.90E-01	-0.276	0.118	**2.27E-02**	0.117	0.032	**2.70E-04**	-0.131	0.121	2.85E-01	-0.141	0.032	**1.30E-05**	0.047	0.074	5.21E-01
rs12661281	6	31950577	T/A	*SLC44A4*	CODING	2	0.499	0.390	0.638	**3.04E-08**	0.014	0.051	7.80E-01	-0.008	0.123	9.51E-01	-0.050	0.050	3.20E-01	-0.128	0.121	2.97E-01	0.062	0.051	2.20E-01	0.023	0.074	7.53E-01
rs16891315	6	26219735	G/A	*HIST1H1T | HIST1H2BC*	INTERGENIC	2	1.784	1.453	2.189	**3.04E-08**	0.005	0.048	9.10E-01	0.030	0.123	8.06E-01	0.104	0.047	**2.80E-02**	-0.108	0.122	3.77E-01	-0.113	0.047	**1.70E-02**	-0.071	0.074	3.36E-01
rs11967684	6	31307745	A/G	*HCG27 | HLA-C*	INTERGENIC	1	0.620	0.523	0.735	**3.31E-08**	0.035	0.033	2.90E-01	0.029	0.124	8.13E-01	-0.145	0.033	**1.30E-05**	0.197	0.121	1.07E-01	0.191	0.033	**1.00E-08**	-0.081	0.074	2.76E-01
rs9275224	6	32767856	G/A	*HLA-DQB1 | HLA-DQA2*	INTERGENIC	2	0.628	0.533	0.741	**3.45E-08**	0.095	0.032	**2.80E-03**	0.132	0.122	2.84E-01	-0.022	0.031	4.80E-01	0.176	0.120	1.49E-01	0.104	0.032	**1.02E-03**	-0.037	0.074	6.16E-01
rs9468925	6	31366816	A/G	*HLA-C | HLA-B*	INTERGENIC	2	0.628	0.533	0.741	**3.61E-08**	0.063	0.033	6.00E-02	0.127	0.122	3.02E-01	-0.112	0.033	**7.00E-04**	0.209	0.120	8.63E-02	0.175	0.033	**1.40E-07**	-0.091	0.074	2.18E-01
rs2523765	6	29925085	A/T	*LOC100133214 | HLA-H*	INTERGENIC	2	1.534	1.317	1.786	**3.63E-08**	-0.056	0.032	7.40E-02	-0.083	0.123	5.02E-01	0.148	0.031	**2.10E-06**	-0.303	0.116	**1.15E-02**	-0.215	0.031	**8.90E-12**	-0.019	0.074	8.01E-01
rs9268404	6	32449697	A/G	*C6orf10 | BTNL2*	INTERGENIC	1	0.530	0.423	0.664	**3.67E-08**	-	-	-	-0.016	0.123	8.97E-01	-	-	-	0.156	0.121	2.02E-01	-	-	-	0.039	0.074	5.96E-01
rs9268515	6	32487273	C/G	*BTNL2 | HLA-DRA*	INTERGENIC	2	0.521	0.413	0.657	**3.70E-08**	-	-	-	-0.088	0.123	4.73E-01	-	-	-	-0.120	0.121	3.25E-01	-	-	-	0.062	0.074	4.05E-01
rs16891334	6	26232282	G/A	*HIST1H2BC | HIST1H2AC*	INTERGENIC	2	1.793	1.456	2.207	**3.71E-08**	0.012	0.048	7.90E-01	0.030	0.123	8.06E-01	0.113	0.047	**1.70E-02**	-0.108	0.122	3.77E-01	-0.117	0.048	**1.39E-02**	-0.071	0.074	3.36E-01
rs6457658	6	32845127	A/G	*HLA-DQB2 | HLA-DOB*	INTERGENIC	2	0.638	0.544	0.749	**3.76E-08**	0.027	0.033	4.10E-01	0.311	0.117	**9.98E-03**	-0.076	0.033	**1.90E-02**	0.120	0.122	3.26E-01	0.111	0.033	**6.80E-04**	-0.054	0.074	4.64E-01
rs28361060	6	32411826	A/G	*C6orf10*	INTRON	2	0.536	0.429	0.670	**3.85E-08**	-	-	-	-0.088	0.123	4.73E-01	-	-	-	-0.120	0.121	3.25E-01	-	-	-	0.054	0.074	4.69E-01
rs9268644	6	32516022	C/A	*HLA-DRA*	INTRON	2	0.639	0.545	0.750	**3.93E-08**	-0.118	0.032	**2.00E-04**	0.047	0.123	7.02E-01	0.124	0.031	**8.30E-05**	0.238	0.119	**4.91E-02**	-0.242	0.032	**2.10E-14**	-0.007	0.074	9.29E-01
rs1124131	6	28488227	C/A	*ZSCAN23*	INTRON	1	1.640	1.375	1.957	**3.95E-08**	-0.007	0.033	8.40E-01	-0.055	0.123	6.58E-01	0.085	0.033	**1.04E-02**	-0.107	0.122	3.82E-01	-0.093	0.033	**5.40E-03**	0.122	0.074	9.91E-02
rs2428507	6	30337285	G/A	*FLJ45422*	INTRON	2	1.525	1.312	1.773	**3.98E-08**	-0.002	0.033	9.40E-01	-0.148	0.123	2.33E-01	0.123	0.032	**1.60E-04**	0.025	0.124	8.42E-01	-0.125	0.033	**1.30E-04**	-0.173	0.074	**1.95E-02**
rs1558205	6	28490241	A/C	*ZSCAN23*	INTRON	2	1.557	1.329	1.823	**4.06E-08**	-0.009	0.033	7.80E-01	-0.072	0.123	5.57E-01	0.071	0.033	**3.10E-02**	-0.118	0.122	3.34E-01	-0.081	0.033	**1.50E-02**	0.122	0.074	9.89E-02
rs9283880	6	27823222	C/A	*ZNF184 | LOC100131289*	INTERGENIC	2	1.571	1.337	1.846	**4.24E-08**	0.039	0.037	2.90E-01	0.103	0.123	4.04E-01	0.125	0.037	**6.50E-04**	0.017	0.123	8.93E-01	-0.091	0.037	**1.36E-02**	-0.016	0.075	8.33E-01
rs6910549	6	25888790	A/G	*SLC17A4 | SLC17A1*	INTERGENIC	2	1.796	1.457	2.215	**4.29E-08**	0.025	0.050	6.10E-01	-0.018	0.123	8.85E-01	0.139	0.049	**4.80E-03**	-0.136	0.121	2.67E-01	-0.132	0.050	**7.70E-03**	-0.097	0.074	1.90E-01
rs2249742	6	31348700	A/G	*HLA-C | HLA-B*	INTERGENIC	2	0.637	0.542	0.749	**4.30E-08**	-0.025	0.032	4.20E-01	-0.167	0.122	1.78E-01	-0.166	0.031	**1.30E-07**	-0.010	0.123	9.38E-01	0.154	0.032	**1.10E-06**	0.027	0.076	7.20E-01
rs2395173	6	32512837	A/G	*BTNL2 | HLA-DRA*	INTERGENIC	2	0.598	0.497	0.719	**4.37E-08**	-0.073	0.034	**3.20E-02**	0.068	0.123	5.80E-01	0.038	0.034	2.60E-01	0.181	0.120	1.37E-01	-0.099	0.034	**3.50E-03**	-0.094	0.074	2.03E-01
rs984778	6	32508066	G/A	*BTNL2 | HLA-DRA*	INTERGENIC	2	0.598	0.497	0.719	**4.37E-08**	-0.073	0.034	**3.20E-02**	0.068	0.123	5.80E-01	0.038	0.034	2.60E-01	0.181	0.120	1.37E-01	-0.099	0.034	**3.50E-03**	-0.116	0.073	1.17E-01
rs3135335	6	32509823	G/C	*BTNL2 | HLA-DRA*	INTERGENIC	2	0.598	0.497	0.719	**4.38E-08**	-0.073	0.034	**3.20E-02**	0.068	0.123	5.80E-01	0.038	0.034	2.60E-01	0.181	0.120	1.37E-01	-0.099	0.034	**3.50E-03**	-0.116	0.073	1.17E-01
rs3734563	6	28457704	G/A	*ZKSCAN3 | ZSCAN12*	INTERGENIC	2	1.558	1.329	1.826	**4.45E-08**	-	-	-	-0.055	0.123	6.58E-01	-	-	-	-0.107	0.122	3.82E-01	-	-	-	0.122	0.074	9.91E-02
rs1361385	6	28466299	G/A	*ZSCAN12*	UTR	2	1.558	1.329	1.826	**4.50E-08**	-0.005	0.033	8.90E-01	-0.055	0.123	6.58E-01	0.087	0.033	**8.70E-03**	-0.107	0.122	3.82E-01	-0.093	0.033	**5.30E-03**	0.122	0.074	9.91E-02
rs2041230	6	28473494	G/A	*ZSCAN12*	INTRON	2	1.558	1.329	1.826	**4.50E-08**	-0.006	0.033	8.50E-01	-0.055	0.123	6.58E-01	0.086	0.033	**9.80E-03**	-0.107	0.122	3.82E-01	-0.093	0.033	**5.40E-03**	0.122	0.074	9.91E-02
rs4357130	6	28475662	C/A	*ZSCAN12 | ZSCAN23*	INTERGENIC	2	1.557	1.328	1.825	**4.64E-08**	-0.006	0.033	8.50E-01	-0.055	0.123	6.58E-01	0.086	0.033	**9.80E-03**	-0.107	0.122	3.82E-01	-0.093	0.033	**5.40E-03**	0.122	0.074	9.91E-02
rs6907950	6	28478225	A/G	*ZSCAN12 | ZSCAN23*	INTERGENIC	2	1.552	1.326	1.817	**4.66E-08**	-0.006	0.033	8.50E-01	-0.055	0.123	6.58E-01	0.086	0.033	**9.80E-03**	-0.107	0.122	3.82E-01	-0.093	0.033	**5.40E-03**	0.123	0.074	9.82E-02
rs6908137	6	28478372	C/A	*ZSCAN12 | ZSCAN23*	INTERGENIC	2	1.552	1.325	1.817	**4.75E-08**	-0.006	0.033	8.50E-01	-0.055	0.123	6.58E-01	0.085	0.033	**1.06E-02**	-0.107	0.122	3.82E-01	-0.091	0.033	**6.50E-03**	0.122	0.074	9.91E-02
rs6924102	6	32919361	G/A	*PSMB8*	INTRON	2	1.526	1.311	1.776	**4.80E-08**	0.084	0.032	**9.80E-03**	0.106	0.122	3.91E-01	0.001	0.032	9.70E-01	-0.083	0.122	4.98E-01	0.064	0.032	**4.70E-02**	0.022	0.074	7.68E-01
rs3094188	6	31250224	C/A	*POU5F1 | LOC100130889*	INTERGENIC	2	0.634	0.538	0.746	**4.82E-08**	-0.052	0.033	1.15E-01	0.166	0.123	1.83E-01	0.118	0.033	**3.10E-04**	-0.009	0.124	9.44E-01	-0.177	0.033	**7.30E-08**	-0.011	0.074	8.78E-01
rs2229094	6	31648535	G/A	*LTA*	CODING	2	0.582	0.479	0.707	**4.95E-08**	-	-	-	-0.079	0.123	5.26E-01	-	-	-	0.014	0.123	9.10E-01	-	-	-	-0.151	0.075	**4.48E-02**
rs4713411	6	31095155	A/C	*C6orf205 | LOC729792*	INTERGENIC	2	0.623	0.526	0.739	5.01E-08	0.048	0.032	1.36E-01	-0.138	0.122	2.60E-01	-0.101	0.032	**1.60E-03**	-0.054	0.122	6.62E-01	0.144	0.032	**7.00E-06**	-0.063	0.074	3.94E-01
rs4254981	6	28479381	C/A	*ZSCAN12 | ZSCAN23*	INTERGENIC	2	1.558	1.328	1.828	5.12E-08	-0.006	0.033	8.50E-01	-0.055	0.123	6.58E-01	0.086	0.033	**9.80E-03**	-0.107	0.122	3.82E-01	-0.093	0.033	**5.40E-03**	0.122	0.074	9.91E-02
rs6922169	6	28490911	A/G	*ZSCAN23*	INTRON	2	1.558	1.328	1.828	5.12E-08	-0.006	0.033	8.50E-01	-0.055	0.123	6.58E-01	0.086	0.033	**9.80E-03**	-0.107	0.122	3.82E-01	-0.093	0.033	**5.40E-03**	0.122	0.074	9.91E-02
rs7740351	6	28507391	C/A	*ZSCAN23*	INTRON	2	1.558	1.328	1.828	5.17E-08	-	-	-	-0.055	0.123	6.58E-01	-	-	-	-0.107	0.122	3.82E-01	-	-	-	0.122	0.074	9.91E-02
rs7745603	6	27198383	A/G	*LOC100133205 | HIST1H2BJ*	INTERGENIC	2	1.583	1.342	1.868	5.20E-08	0.031	0.038	4.00E-01	0.036	0.123	7.73E-01	0.130	0.037	**4.80E-04**	-0.053	0.122	6.65E-01	-0.105	0.037	**5.30E-03**	0.037	0.074	6.14E-01
rs12660860	6	30872220	G/A	*IER3 | DDR1*	INTERGENIC	2	0.502	0.392	0.644	5.27E-08	0.052	0.044	2.40E-01	-0.108	0.123	3.84E-01	-0.087	0.043	**4.60E-02**	-0.127	0.122	2.99E-01	0.143	0.044	**1.04E-03**	-0.040	0.074	5.89E-01
rs1150736	6	30150934	A/G	*RNF39*	INTRON	2	1.546	1.321	1.809	5.56E-08	-0.016	0.033	6.40E-01	-0.084	0.124	5.01E-01	0.143	0.033	**1.40E-05**	-0.049	0.123	6.93E-01	-0.166	0.033	**6.00E-07**	-0.103	0.074	1.64E-01
rs3132454	6	31597623	G/A	*MICB | MCCD1*	INTERGENIC	2	0.627	0.530	0.742	5.57E-08	-0.009	0.033	7.70E-01	0.040	0.125	7.49E-01	-0.150	0.032	**3.80E-06**	-0.037	0.124	7.67E-01	0.146	0.033	**7.70E-06**	-0.020	0.075	7.90E-01
rs2516708	6	30336700	A/G	*FLJ45422*	INTRON	2	1.516	1.305	1.762	5.59E-08	-	-	-	-0.132	0.123	2.86E-01	-	-	-	0.037	0.123	7.64E-01	-	-	-	-0.174	0.073	**1.90E-02**
rs1892253	6	25890293	A/C	*SLC17A4 | SLC17A1*	INTERGENIC	2	1.795	1.453	2.217	5.63E-08	0.025	0.050	6.10E-01	-0.018	0.123	8.85E-01	0.139	0.049	**4.80E-03**	-0.136	0.121	2.67E-01	-0.132	0.050	**7.70E-03**	-0.097	0.074	1.90E-01
rs16894095	6	28498209	A/G	*ZSCAN23*	INTRON	1	1.630	1.366	1.945	5.79E-08	-0.006	0.033	8.50E-01	-0.055	0.123	6.58E-01	0.086	0.033	**9.80E-03**	-0.107	0.122	3.82E-01	-0.093	0.033	**5.40E-03**	0.124	0.073	9.30E-02
rs614549	6	31948604	G/A	*SLC44A4*	INTRON	2	0.612	0.512	0.731	6.17E-08	0.018	0.034	5.90E-01	0.055	0.123	6.55E-01	-0.098	0.033	**3.30E-03**	0.213	0.119	7.97E-02	0.123	0.034	**2.60E-04**	0.022	0.074	7.65E-01
rs2071465	6	32913448	C/G	*TAP2*	INTRON	2	1.524	1.308	1.776	6.48E-08	0.102	0.033	**1.80E-03**	0.110	0.123	3.75E-01	0.008	0.032	8.00E-01	-0.096	0.122	4.36E-01	0.073	0.033	**2.50E-02**	0.021	0.074	7.81E-01
rs563412	6	32323041	A/G	*NOTCH4 | C6orf10*	INTERGENIC	1	0.610	0.510	0.730	6.56E-08	0.063	0.033	5.50E-02	0.023	0.123	8.51E-01	-0.128	0.032	**7.80E-05**	0.102	0.122	4.09E-01	0.194	0.033	**2.70E-09**	0.083	0.074	2.59E-01
rs1610649	6	29876896	G/A	*HCG4 | HLA-G*	INTERGENIC	1	1.620	1.360	1.930	6.57E-08	-0.012	0.033	7.00E-01	-0.032	0.123	7.97E-01	0.052	0.032	1.08E-01	-0.116	0.122	3.45E-01	-0.054	0.032	9.60E-02	0.026	0.074	7.28E-01
rs6457300	6	31106721	C/A	*LOC729792*	INTRON	2	0.637	0.541	0.751	6.64E-08	0.056	0.034	9.70E-02	0.038	0.125	7.60E-01	-0.113	0.033	**6.90E-04**	0.010	0.125	9.38E-01	0.172	0.034	**3.00E-07**	0.028	0.074	7.06E-01
rs3131043	6	30866445	G/A	*IER3 | DDR1*	INTERGENIC	2	1.527	1.309	1.781	7.16E-08	0.015	0.032	6.50E-01	-0.028	0.123	8.18E-01	0.067	0.032	**3.60E-02**	-0.052	0.122	6.73E-01	-0.052	0.032	1.06E-01	-0.006	0.074	9.40E-01
rs9261425	6	30189087	A/G	*TRIM31 | TRIM40*	INTERGENIC	2	1.518	1.304	1.767	7.19E-08	-0.014	0.032	6.70E-01	0.058	0.123	6.41E-01	0.133	0.031	**2.00E-05**	-0.077	0.122	5.31E-01	-0.163	0.032	**2.20E-07**	0.076	0.074	3.03E-01
rs3800328	6	28498822	A/G	*ZSCAN23*	INTRON	2	1.551	1.322	1.819	7.22E-08	-0.006	0.033	8.50E-01	-0.055	0.123	6.58E-01	0.086	0.033	**9.80E-03**	-0.107	0.122	3.82E-01	-0.093	0.033	**5.40E-03**	0.122	0.074	9.91E-02
rs453779	6	33083359	A/G	*HLA-DOA*	INTRON	2	1.523	1.307	1.774	7.30E-08	0.003	0.032	9.20E-01	-0.114	0.122	3.53E-01	0.003	0.032	9.20E-01	-0.179	0.120	1.41E-01	-0.001	0.032	9.90E-01	-0.040	0.074	5.91E-01
rs9277341	6	33147603	G/A	*HLA-DPA1*	INTRON	2	1.542	1.317	1.805	7.49E-08	-0.046	0.034	1.80E-01	0.004	0.124	9.75E-01	-0.031	0.034	3.60E-01	-0.077	0.122	5.31E-01	-0.008	0.034	8.20E-01	0.059	0.074	4.27E-01
rs9266638	6	31455036	A/G	*LOC729816 | LOC100129668*	INTERGENIC	2	0.591	0.488	0.716	7.98E-08	0.075	0.036	**4.00E-02**	-0.038	0.123	7.56E-01	-0.168	0.036	**3.00E-06**	0.122	0.122	3.18E-01	0.248	0.036	**6.50E-12**	-0.057	0.075	4.49E-01
rs2856448	6	32122553	G/A	*TNXB*	INTRON	2	0.654	0.560	0.764	8.08E-08	-	-	-	-0.127	0.122	3.02E-01	-	-	-	0.153	0.121	2.09E-01	-	-	-	-0.017	0.074	8.20E-01
rs9275393	6	32777417	A/G	*HLA-DQB1 | HLA-DQA2*	INTERGENIC	2	0.604	0.502	0.726	8.45E-08	-0.020	0.046	6.70E-01	0.170	0.122	1.68E-01	-0.050	0.046	2.80E-01	0.115	0.122	3.49E-01	0.035	0.046	4.50E-01	-0.033	0.075	6.62E-01
rs9275428	6	32778956	G/A	*HLA-DQB1 | HLA-DQA2*	INTERGENIC	2	0.604	0.502	0.726	8.45E-08	-0.020	0.046	6.70E-01	0.170	0.122	1.68E-01	-0.050	0.046	2.80E-01	0.115	0.122	3.49E-01	0.035	0.046	4.50E-01	-0.033	0.075	6.62E-01
rs9275390	6	32777134	G/A	*HLA-DQB1 | HLA-DQA2*	INTERGENIC	2	0.604	0.502	0.726	8.50E-08	0.009	0.037	8.20E-01	0.170	0.122	1.68E-01	-0.028	0.037	4.40E-01	0.115	0.122	3.49E-01	0.032	0.037	3.90E-01	-0.033	0.075	6.62E-01
rs9275332	6	32774921	A/G	*HLA-DQB1 | HLA-DQA2*	INTERGENIC	2	0.604	0.502	0.726	8.57E-08	0.009	0.037	8.20E-01	0.170	0.122	1.68E-01	-0.028	0.037	4.40E-01	0.115	0.122	3.49E-01	0.032	0.037	3.90E-01	-0.033	0.075	6.62E-01
rs9275425	6	32778852	A/C	*HLA-DQB1 | HLA-DQA2*	INTERGENIC	2	0.604	0.502	0.726	8.65E-08	-0.020	0.046	6.70E-01	0.170	0.122	1.68E-01	-0.050	0.046	2.80E-01	0.115	0.122	3.49E-01	0.035	0.046	4.50E-01	-0.033	0.075	6.62E-01
rs2844762	6	30344733	G/A	*FLJ45422 | LOC100133303*	INTERGENIC	2	1.515	1.301	1.764	8.77E-08	-	-	-	-0.132	0.123	2.86E-01	-	-	-	0.037	0.123	7.64E-01	-	-	-	-0.174	0.073	**1.90E-02**
rs2844766	6	30338640	G/A	*FLJ45422*	INTRON	2	1.515	1.301	1.764	8.77E-08	-0.002	0.033	9.40E-01	-0.132	0.123	2.86E-01	0.125	0.033	**1.20E-04**	0.037	0.123	7.64E-01	-0.127	0.033	**1.00E-04**	-0.174	0.073	**1.90E-02**
rs3129867	6	32512198	G/C	*BTNL2 | HLA-DRA*	INTERGENIC	2	0.586	0.482	0.713	8.92E-08	-0.085	0.034	**1.30E-02**	0.059	0.123	6.30E-01	0.034	0.034	3.20E-01	0.180	0.120	1.40E-01	-0.107	0.034	**1.80E-03**	-0.100	0.074	1.77E-01
rs3104406	6	32790421	A/G	*HLA-DQB1 | HLA-DQA2*	INTERGENIC	2	0.611	0.510	0.732	8.98E-08	-	-	-	0.022	0.123	8.61E-01	-	-	-	0.206	0.120	9.08E-02	-	-	-	0.046	0.074	5.38E-01
rs6903535	6	28525201	G/A	*COX11P | GPX6*	INTERGENIC	2	1.507	1.297	1.752	9.16E-08	-0.038	0.032	2.30E-01	0.011	0.123	9.30E-01	0.126	0.032	**7.00E-05**	-0.141	0.121	2.48E-01	-0.170	0.032	**1.10E-07**	0.133	0.073	7.18E-02
rs11752919	6	28511582	G/A	*ZSCAN23*	INTRON	2	1.507	1.296	1.752	9.17E-08	-0.038	0.032	2.30E-01	0.011	0.123	9.30E-01	0.126	0.032	**7.00E-05**	-0.141	0.121	2.48E-01	-0.170	0.032	**1.10E-07**	0.133	0.073	7.18E-02
rs3130814	6	29304160	C/A	*OR2J2 | LOC651503*	INTERGENIC	2	1.503	1.294	1.745	9.27E-08	0.035	0.033	2.80E-01	-0.060	0.123	6.27E-01	0.043	0.033	1.80E-01	-0.136	0.122	2.67E-01	-0.007	0.033	8.30E-01	0.043	0.074	5.63E-01
rs3116803	6	29775851	G/A	*ZFP57 | HLA-F*	INTERGENIC	1	1.640	1.368	1.967	9.35E-08	0.019	0.037	6.00E-01	-0.103	0.123	4.03E-01	0.138	0.036	**1.30E-04**	-0.079	0.122	5.19E-01	-0.133	0.036	**2.50E-04**	-0.070	0.074	3.49E-01
rs3131888	6	29771868	G/A	*ZFP57 | HLA-F*	INTERGENIC	2	1.506	1.296	1.750	9.46E-08	-0.002	0.032	9.50E-01	-0.136	0.122	2.69E-01	0.030	0.032	3.40E-01	0.093	0.122	4.49E-01	-0.032	0.032	3.20E-01	-0.035	0.074	6.39E-01

rs2508037	6	30026415	A/G	*HLA-A | HCG9*	INTERGENIC	2	1.509	1.297	1.755	9.60E-08	-0.066	0.033	**4.20E-02**	0.051	0.124	6.80E-01	0.153	0.032	**2.00E-06**	-0.253	0.119	**3.78E-02**	-0.228	0.032	**2.20E-12**	-0.015	0.075	8.43E-01
rs6917130	6	28514800	G/A	*ZSCAN23*	INTRON	2	1.506	1.295	1.751	1.01E-07	-0.038	0.032	2.30E-01	0.011	0.123	9.30E-01	0.126	0.032	**7.00E-05**	-0.141	0.121	2.48E-01	-0.170	0.032	**1.10E-07**	0.133	0.074	7.26E-02
rs7453920	6	32837990	G/A	*HLA-DQB2*	INTRON	1	0.620	0.520	0.740	1.09E-07	-0.045	0.032	1.60E-01	0.256	0.123	**4.10E-02**	0.113	0.032	**4.00E-04**	0.254	0.122	**4.19E-02**	-0.165	0.032	**2.80E-07**	-0.087	0.075	2.47E-01
rs2535335	6	30920476	G/A	*IER3 | DDR1*	INTERGENIC	2	0.648	0.552	0.760	1.11E-07	-0.002	0.033	9.50E-01	-0.226	0.120	6.43E-02	-0.077	0.033	**1.90E-02**	-0.135	0.121	2.71E-01	0.088	0.033	**7.70E-03**	-0.038	0.074	6.08E-01
rs12660883	6	30872399	G/A	*IER3 | DDR1*	INTERGENIC	2	0.504	0.391	0.649	1.16E-07	0.052	0.044	2.40E-01	-0.108	0.123	3.84E-01	-0.087	0.043	**4.60E-02**	-0.127	0.122	2.99E-01	0.143	0.044	**1.04E-03**	-0.040	0.074	5.89E-01
rs28670020	6	30872817	A/G	*IER3 | DDR1*	INTERGENIC	2	0.504	0.391	0.649	1.16E-07	-	-	-	-0.108	0.123	3.84E-01	-	-	-	-0.127	0.122	2.99E-01	-	-	-	-0.040	0.074	5.89E-01
rs9275407	6	32778015	A/C	*HLA-DQB1 | HLA-DQA2*	INTERGENIC	2	0.612	0.510	0.734	1.23E-07	-0.020	0.046	6.70E-01	0.170	0.122	1.68E-01	-0.050	0.046	2.80E-01	0.115	0.122	3.49E-01	0.035	0.046	4.50E-01	-0.033	0.075	6.62E-01
rs1736936	6	29902296	A/G	*HCG4 | HLA-G*	INTERGENIC	2	1.511	1.297	1.761	1.25E-07	-0.010	0.031	7.50E-01	0.009	0.123	9.44E-01	0.055	0.031	7.90E-02	-0.006	0.122	9.62E-01	-0.053	0.031	9.20E-02	0.016	0.074	8.26E-01
rs3131784	6	31011927	G/A	*SFTPG | LOC100129065*	INTERGENIC	2	0.658	0.564	0.769	1.26E-07	-0.040	0.032	2.20E-01	-0.109	0.122	3.75E-01	0.012	0.032	7.00E-01	-0.096	0.122	4.35E-01	-0.046	0.032	1.60E-01	0.007	0.074	9.29E-01
rs2523656	6	31552027	C/G	*3.8-1 | MICB*	INTERGENIC	2	0.615	0.514	0.737	1.28E-07	-	-	-	0.036	0.123	7.69E-01	-	-	-	0.176	0.121	1.49E-01	-	-	-	-0.085	0.074	2.52E-01
rs2516714	6	30334283	G/A	*TRIM26 | FLJ45422*	INTERGENIC	2	1.515	1.298	1.767	1.30E-07	-0.007	0.033	8.40E-01	-0.148	0.123	2.33E-01	0.110	0.033	**8.30E-04**	0.025	0.124	8.42E-01	-0.115	0.033	**5.50E-04**	-0.173	0.074	**1.95E-02**
rs1610677	6	29897150	G/A	*HCG4 | HLA-G*	INTERGENIC	2	1.510	1.295	1.760	1.34E-07	-0.014	0.031	6.50E-01	0.006	0.123	9.61E-01	0.054	0.031	8.20E-02	-0.014	0.122	9.11E-01	-0.056	0.031	7.40E-02	0.016	0.074	8.34E-01
rs2734335	6	32001923	A/G	*ZBTB12 | C2*	INTERGENIC	2	0.654	0.559	0.766	1.36E-07	0.020	0.032	5.30E-01	0.160	0.122	1.93E-01	-0.152	0.032	**1.60E-06**	0.185	0.120	1.29E-01	0.182	0.032	**1.20E-08**	-0.012	0.074	8.68E-01
rs68600	6	33011702	A/G	*HLA-DMB*	INTRON	2	0.661	0.567	0.771	1.37E-07	0.046	0.032	1.49E-01	0.087	0.123	4.79E-01	-0.010	0.031	7.60E-01	0.025	0.123	8.40E-01	0.042	0.032	1.80E-01	0.031	0.074	6.71E-01
rs6931277	6	32691335	A/T	*HLA-DRB1 | HLA-DQA1*	INTERGENIC	2	0.547	0.437	0.685	1.43E-07	-	-	-	0.091	0.123	4.63E-01	-	-	-	0.012	0.123	9.26E-01	-	-	-	0.019	0.074	7.98E-01
rs3094187	6	31234923	G/A	*TCF19*	UTR	2	0.662	0.568	0.772	1.45E-07	-0.048	0.032	1.32E-01	0.071	0.123	5.63E-01	0.049	0.032	1.26E-01	-0.092	0.122	4.55E-01	-0.100	0.032	**1.90E-03**	-0.042	0.074	5.68E-01
rs6928399	6	31303197	A/G	*HCG27 | HLA-C*	INTERGENIC	2	0.637	0.539	0.754	1.45E-07	0.035	0.034	3.00E-01	0.001	0.124	9.94E-01	-0.143	0.033	**1.60E-05**	0.232	0.120	5.75E-02	0.189	0.033	**1.40E-08**	-0.082	0.074	2.69E-01
rs1884123	6	29364399	A/G	*LOC651503 | OR5U1*	INTERGENIC	2	1.505	1.292	1.753	1.46E-07	0.046	0.033	1.70E-01	-0.003	0.124	9.81E-01	0.064	0.033	5.30E-02	-0.064	0.123	6.04E-01	-0.021	0.033	5.30E-01	0.053	0.074	4.79E-01
rs4713598	6	32914764	C/A	*TAP2 | PSMB8*	INTERGENIC	2	1.509	1.294	1.759	1.47E-07	0.086	0.032	**8.00E-03**	0.106	0.122	3.91E-01	0.004	0.032	9.00E-01	-0.083	0.122	4.98E-01	0.063	0.032	5.00E-02	0.023	0.074	7.60E-01
rs9380192	6	30880323	G/A	*IER3 | DDR1*	INTERGENIC	2	0.636	0.537	0.753	1.50E-07	0.081	0.035	**2.00E-02**	-0.118	0.123	3.38E-01	-0.072	0.034	**3.60E-02**	-0.166	0.121	1.76E-01	0.160	0.034	**3.20E-06**	0.004	0.074	9.54E-01
rs9268543	6	32492779	T/A	*BTNL2 | HLA-DRA*	INTERGENIC	2	0.534	0.422	0.675	1.54E-07	0.084	0.042	**4.60E-02**	-0.088	0.123	4.73E-01	-0.051	0.042	2.30E-01	-0.120	0.121	3.25E-01	0.120	0.042	**4.30E-03**	0.053	0.074	4.73E-01
rs2517527	6	31129526	A/G	*LOC729792 | HCG22*	INTERGENIC	2	0.648	0.551	0.762	1.60E-07	0.028	0.033	3.90E-01	0.190	0.121	1.21E-01	0.029	0.033	3.70E-01	-0.074	0.122	5.45E-01	-0.015	0.033	6.50E-01	0.021	0.074	7.82E-01
rs10807077	6	31109446	G/A	*LOC729792*	INTRON	2	0.646	0.549	0.761	1.61E-07	0.056	0.034	9.70E-02	0.038	0.125	7.60E-01	-0.119	0.034	**4.00E-04**	0.010	0.125	9.38E-01	0.178	0.034	**1.40E-07**	0.036	0.074	6.28E-01
rs3094551	6	29462778	A/G	*OR12D3 | OR12D2*	INTERGENIC	2	1.495	1.286	1.738	1.73E-07	0.062	0.032	5.20E-02	0.010	0.125	9.35E-01	0.022	0.032	4.90E-01	-0.071	0.124	5.69E-01	0.042	0.032	1.90E-01	-0.125	0.074	9.16E-02
rs3094550	6	29462788	C/A	*OR12D3 | OR12D2*	INTERGENIC	2	1.494	1.285	1.737	1.77E-07	0.062	0.032	5.20E-02	0.010	0.125	9.35E-01	0.022	0.032	4.90E-01	-0.071	0.124	5.69E-01	0.042	0.032	1.90E-01	-0.125	0.074	9.16E-02
rs3905495	6	31373518	A/G	*HLA-C | HLA-B*	INTERGENIC	2	0.634	0.535	0.753	1.80E-07	0.046	0.034	1.70E-01	0.073	0.123	5.55E-01	-0.147	0.033	**1.00E-05**	0.217	0.120	7.40E-02	0.204	0.033	**1.10E-09**	-0.109	0.074	1.43E-01
rs2187688	6	32979979	A/G	*PSMB9 | HLA-DMB*	INTERGENIC	2	0.665	0.571	0.775	1.83E-07	-0.068	0.032	**3.50E-02**	-0.086	0.123	4.84E-01	-0.026	0.032	4.20E-01	0.122	0.121	3.17E-01	-0.023	0.032	4.80E-01	0.044	0.074	5.50E-01
rs379157	6	29464566	G/A	*OR12D3 | OR12D2*	INTERGENIC	2	1.494	1.285	1.738	1.92E-07	0.031	0.032	3.30E-01	-0.004	0.124	9.75E-01	0.010	0.032	7.40E-01	-0.073	0.123	5.55E-01	0.022	0.032	4.90E-01	-0.086	0.074	2.45E-01
rs241458	6	32996544	G/C	*PSMB9 | HLA-DMB*	INTERGENIC	2	0.662	0.567	0.774	1.97E-07	-0.067	0.032	**3.70E-02**	-0.086	0.123	4.84E-01	-0.018	0.032	5.70E-01	0.122	0.121	3.17E-01	-0.031	0.032	3.40E-01	0.044	0.074	5.50E-01
rs13218591	6	26484811	G/A	*BTN3A2*	UTR	2	1.517	1.297	1.776	2.04E-07	-	-	-	0.027	0.124	8.27E-01	-	-	-	-0.108	0.122	3.82E-01	-	-	-	-0.046	0.074	5.33E-01
rs9380010	6	27791551	G/A	*ZNF184 | LOC100131289*	INTERGENIC	2	1.527	1.301	1.791	2.11E-07	0.045	0.037	2.30E-01	0.103	0.123	4.04E-01	0.135	0.037	**2.60E-04**	0.017	0.123	8.93E-01	-0.096	0.037	**9.90E-03**	-0.010	0.075	8.94E-01
rs9393848	6	27796348	A/G	*ZNF184 | LOC100131289*	INTERGENIC	2	1.527	1.301	1.791	2.11E-07	0.045	0.037	2.30E-01	0.103	0.123	4.04E-01	0.135	0.037	**2.60E-04**	0.017	0.123	8.93E-01	-0.096	0.037	**9.90E-03**	-0.010	0.075	8.94E-01
rs2535323	6	30826159	G/A	*IER3 | DDR1*	INTERGENIC	2	0.528	0.414	0.672	2.21E-07	-	-	-	-0.151	0.122	2.18E-01	-	-	-	-0.168	0.121	1.68E-01	-	-	-	-0.015	0.074	8.38E-01
rs6932517	6	32786160	G/C	*HLA-DQB1 | HLA-DQA2*	INTERGENIC	2	0.647	0.549	0.763	2.21E-07	-0.098	0.032	**2.10E-03**	0.109	0.122	3.75E-01	0.081	0.032	**1.03E-02**	0.279	0.117	**2.02E-02**	-0.174	0.032	**4.80E-08**	-0.032	0.074	6.67E-01
rs7776351	6	27834710	A/G	*ZNF184 | LOC100131289*	INTERGENIC	2	1.525	1.299	1.789	2.30E-07	0.031	0.037	4.00E-01	0.103	0.123	4.04E-01	0.127	0.037	**4.90E-04**	0.017	0.123	8.93E-01	-0.102	0.037	**5.90E-03**	-0.010	0.075	8.94E-01
rs742046	6	27847233	G/A	*LOC100131289 | HIST1H2BL*	INTERGENIC	2	1.532	1.303	1.801	2.34E-07	-	-	-	0.103	0.123	4.04E-01	-	-	-	0.017	0.123	8.93E-01	-	-	-	-0.010	0.074	8.93E-01
rs1265093	6	31215166	A/G	*PSORS1C2 | PSORS1C1*	INTERGENIC	2	0.620	0.517	0.743	2.37E-07	0.101	0.035	**3.50E-03**	-0.031	0.123	8.02E-01	-0.172	0.034	**5.40E-07**	0.141	0.121	2.47E-01	0.274	0.035	**2.30E-15**	-0.011	0.074	8.82E-01
rs7759217	6	27838442	G/A	*LOC100131289*	UTR	2	1.532	1.303	1.801	2.37E-07	0.031	0.037	4.00E-01	0.103	0.123	4.04E-01	0.127	0.037	**4.90E-04**	0.017	0.123	8.93E-01	-0.102	0.037	**5.90E-03**	-0.010	0.074	8.93E-01
rs23544	6	33011615	A/G	*HLA-DMB*	INTRON	2	0.667	0.572	0.778	2.51E-07	0.045	0.032	1.60E-01	0.096	0.123	4.39E-01	-0.009	0.031	7.80E-01	-0.097	0.122	4.32E-01	0.040	0.032	2.00E-01	0.045	0.074	5.39E-01
rs4639381	6	30874744	C/G	*IER3 | DDR1*	INTERGENIC	2	0.645	0.546	0.762	2.53E-07	0.077	0.035	**2.60E-02**	-0.118	0.123	3.38E-01	-0.076	0.034	**2.80E-02**	-0.166	0.121	1.76E-01	0.162	0.035	**3.10E-06**	0.004	0.074	9.54E-01
rs6901520	6	27822554	A/C	*ZNF184 | LOC100131289*	INTERGENIC	2	1.514	1.293	1.772	2.60E-07	0.042	0.037	2.60E-01	0.103	0.123	4.04E-01	0.139	0.037	**1.70E-04**	0.017	0.123	8.93E-01	-0.105	0.037	**5.00E-03**	-0.006	0.075	9.37E-01
rs2240804	6	31028869	A/G	*LOC100129065 / DPCR1*	MISSENSE	2	0.636	0.535	0.756	2.63E-07	0.008	0.033	8.00E-01	-0.100	0.123	4.16E-01	-0.064	0.033	**4.90E-02**	-0.124	0.122	3.13E-01	0.082	0.033	**1.26E-02**	0.049	0.074	5.09E-01
rs6923313	6	31349349	G/A	*HLA-C | HLA-B*	INTERGENIC	2	0.639	0.539	0.758	2.73E-07	-0.072	0.033	**2.80E-02**	0.108	0.122	3.78E-01	0.021	0.033	5.20E-01	-0.069	0.122	5.72E-01	-0.098	0.033	**2.70E-03**	0.132	0.073	7.28E-02
rs3130481	6	31947735	C/G	*SLC44A4*	INTRON	2	0.670	0.575	0.781	2.80E-07	-0.023	0.032	4.80E-01	0.168	0.123	1.77E-01	0.195	0.031	**4.90E-10**	0.223	0.121	6.92E-02	-0.228	0.032	**5.10E-13**	0.117	0.074	1.17E-01
rs3130482	6	31947761	A/C	*SLC44A4*	INTRON	2	0.670	0.575	0.781	2.80E-07	-	-	-	0.168	0.123	1.77E-01	-	-	-	0.223	0.121	6.92E-02	-	-	-	0.117	0.074	1.17E-01
rs3132442	6	31947473	A/G	*SLC44A4*	INTRON	2	0.670	0.575	0.781	2.80E-07	-	-	-	0.168	0.123	1.77E-01	-	-	-	0.223	0.121	6.92E-02	-	-	-	0.117	0.074	1.17E-01
rs3130827	6	29338662	A/G	*LOC651503*	CODING	2	1.483	1.276	1.724	2.82E-07	0.038	0.033	2.60E-01	-0.004	0.124	9.71E-01	0.053	0.033	1.08E-01	-0.122	0.122	3.21E-01	-0.016	0.033	6.20E-01	0.059	0.074	4.29E-01
rs4713422	6	31107881	G/C	*LOC729792*	INTRON	2	0.648	0.549	0.765	2.90E-07	0.063	0.034	5.90E-02	0.067	0.124	5.90E-01	-0.104	0.033	**1.80E-03**	0.093	0.123	4.53E-01	0.167	0.033	**5.40E-07**	0.007	0.075	9.29E-01
rs2523856	6	31129609	G/A	*LOC729792 | HCG22*	INTERGENIC	2	0.651	0.553	0.767	2.95E-07	0.028	0.033	3.90E-01	0.190	0.121	1.21E-01	0.029	0.033	3.70E-01	-0.074	0.122	5.45E-01	-0.015	0.033	6.50E-01	0.018	0.074	8.12E-01
rs9276411	6	32818453	G/A	*HLA-DQA2*	INTRON	1	0.640	0.539	0.759	3.07E-07	-	-	-	0.269	0.121	**2.97E-02**	-	-	-	0.267	0.120	**2.97E-02**	-	-	-	-0.088	0.074	2.33E-01
rs9461405	6	27827354	A/C	*ZNF184 | LOC100131289*	INTERGENIC	2	1.522	1.294	1.789	3.64E-07	0.039	0.037	2.90E-01	0.103	0.123	4.04E-01	0.125	0.037	**6.50E-04**	0.017	0.123	8.93E-01	-0.091	0.037	**1.36E-02**	-0.010	0.075	8.94E-01
rs6904669	6	31300775	G/A	*HCG27 | HLA-C*	INTERGENIC	2	0.658	0.559	0.773	3.73E-07	0.043	0.032	1.80E-01	0.013	0.123	9.15E-01	-0.202	0.032	**1.90E-10**	0.145	0.121	2.36E-01	0.258	0.032	**5.90E-16**	-0.050	0.074	4.97E-01
rs2207338	6	29307803	C/A	*OR2J2 | LOC651503*	INTERGENIC	2	1.484	1.274	1.729	3.92E-07	0.038	0.033	2.60E-01	-0.004	0.124	9.71E-01	0.053	0.033	1.08E-01	-0.122	0.122	3.21E-01	-0.016	0.033	6.20E-01	0.059	0.074	4.29E-01
rs2858312	6	32775208	G/C	*HLA-DQB1 | HLA-DQA2*	INTERGENIC	2	0.596	0.488	0.728	3.99E-07	0.075	0.034	**2.80E-02**	-0.019	0.124	8.78E-01	-0.046	0.034	1.70E-01	0.224	0.120	6.52E-02	0.122	0.034	**3.70E-04**	-0.011	0.075	8.86E-01
rs1029239	6	30246141	C/G	*TRIM15*	INTRON	2	1.485	1.274	1.730	4.05E-07	0.011	0.032	7.50E-01	-0.046	0.124	7.13E-01	0.074	0.032	**2.10E-02**	0.001	0.123	9.94E-01	-0.061	0.032	6.00E-02	-0.043	0.074	5.60E-01
rs154989	6	32985151	A/G	*PSMB9 | HLA-DMB*	INTERGENIC	2	0.662	0.564	0.776	4.11E-07	-0.068	0.032	**3.50E-02**	-0.086	0.123	4.84E-01	-0.026	0.032	4.20E-01	0.122	0.121	3.17E-01	-0.023	0.032	4.80E-01	0.044	0.074	5.50E-01
rs396960	6	32299559	A/T	*NOTCH4*	INTRON	2	0.574	0.463	0.712	4.14E-07	0.058	0.038	1.30E-01	0.082	0.123	5.05E-01	-0.120	0.038	**1.60E-03**	0.455	0.109	**8.92E-05**	0.179	0.038	**2.80E-06**	-0.101	0.074	1.71E-01
rs7454108	6	32789461	G/A	*HLA-DQB1 | HLA-DQA2*	INTERGENIC	2	0.497	0.379	0.652	4.24E-07	0.051	0.052	3.20E-01	0.046	0.123	7.09E-01	0.019	0.051	7.10E-01	0.033	0.122	7.91E-01	0.013	0.051	7.90E-01	-0.013	0.074	8.64E-01
rs17189441	6	30866534	A/G	*IER3 | DDR1*	INTERGENIC	2	0.512	0.395	0.664	4.25E-07	-	-	-	-0.067	0.123	5.86E-01	-	-	-	-0.124	0.121	3.10E-01	-	-	-	-0.028	0.074	7.02E-01
rs171329	6	33012639	G/A	*HLA-DMB*	INTRON	2	1.482	1.272	1.725	4.27E-07	-0.002	0.032	9.60E-01	0.013	0.123	9.16E-01	-0.014	0.031	6.70E-01	0.164	0.121	1.79E-01	0.013	0.031	6.90E-01	-0.008	0.074	9.15E-01
rs2535331	6	30924249	A/G	*IER3 | DDR1*	INTERGENIC	2	0.648	0.548	0.767	4.27E-07	0.003	0.034	9.40E-01	-0.128	0.122	2.98E-01	-0.065	0.033	5.10E-02	-0.079	0.122	5.19E-01	0.080	0.033	**1.60E-02**	-0.006	0.074	9.31E-01
rs376646	6	30022356	A/G	*HLA-A | HCG9*	INTERGENIC	1	1.550	1.308	1.837	4.27E-07	-	-	-	0.041	0.126	7.46E-01	-	-	-	-0.240	0.121	5.12E-02	-	-	-	-0.032	0.075	6.68E-01
rs17481190	6	30850993	A/G	*IER3 | DDR1*	INTERGENIC	2	0.513	0.395	0.664	4.32E-07	0.061	0.044	1.60E-01	-0.067	0.123	5.86E-01	-0.085	0.043	**4.90E-02**	-0.124	0.121	3.10E-01	0.149	0.043	**6.10E-04**	-0.028	0.074	7.02E-01
rs3104413	6	32690628	G/C	*HLA-DRB1 | HLA-DQA1*	INTERGENIC	2	0.547	0.433	0.692	4.39E-07	-	-	-	0.091	0.123	4.63E-01	-	-	-	0.012	0.123	9.26E-01	-	-	-	0.019	0.074	7.98E-01
rs2844664	6	30923924	A/G	*IER3 | DDR1*	INTERGENIC	2	0.649	0.548	0.767	4.42E-07	0.016	0.034	6.50E-01	-0.128	0.122	2.98E-01	-0.056	0.034	1.01E-01	-0.079	0.122	5.19E-01	0.081	0.034	**1.80E-02**	-0.006	0.074	9.31E-01
rs9468922	6	31363265	A/G	*HLA-C | HLA-B*	INTERGENIC	2	0.652	0.552	0.770	4.46E-07	0.065	0.033	5.40E-02	0.060	0.123	6.27E-01	-0.110	0.033	**8.70E-04**	0.139	0.121	2.55E-01	0.174	0.033	**1.60E-07**	-0.078	0.074	2.94E-01
rs9468926	6	31368197	A/G	*HLA-C | HLA-B*	INTERGENIC	2	0.652	0.552	0.770	4.53E-07	0.053	0.035	1.29E-01	0.060	0.123	6.27E-01	-0.159	0.034	**3.70E-06**	0.139	0.121	2.55E-01	0.221	0.034	**1.50E-10**	-0.078	0.074	2.94E-01
rs13214831	6	30839484	A/G	*IER3 | DDR1*	INTERGENIC	2	0.513	0.396	0.665	4.54E-07	0.045	0.043	3.00E-01	-0.067	0.123	5.86E-01	-0.104	0.043	**1.50E-02**	-0.124	0.121	3.10E-01	0.154	0.043	**3.40E-04**	-0.028	0.074	7.02E-01
rs3117330	6	29333774	A/G	*OR2J2 | LOC651503*	INTERGENIC	2	1.484	1.273	1.730	4.54E-07	0.038	0.033	2.60E-01	-0.004	0.124	9.71E-01	0.053	0.033	1.08E-01	-0.122	0.122	3.21E-01	-0.016	0.033	6.20E-01	0.059	0.074	4.29E-01

rs3869075	6	30835261	C/G	*IER3 | DDR1*	INTERGENIC	2	0.513	0.396	0.665	4.60E-07	0.043	0.043	3.20E-01	-0.067	0.123	5.86E-01	-0.104	0.043	**1.50E-02**	-0.124	0.121	3.10E-01	0.153	0.043	**3.80E-04**	-0.028	0.074	7.02E-01
rs2532936	6	31002387	C/A	*VARS2 | SFTPG*	INTERGENIC	2	0.649	0.549	0.768	4.69E-07	-0.001	0.033	9.70E-01	-0.128	0.122	2.98E-01	-0.069	0.033	**3.90E-02**	-0.079	0.122	5.19E-01	0.079	0.033	**1.70E-02**	-0.006	0.074	9.31E-01
rs9262290	6	30993800	A/G	*VARS2*	INTRON	2	0.649	0.549	0.768	4.69E-07	-	-	-	-0.128	0.122	2.98E-01	-	-	-	-0.079	0.122	5.19E-01	-	-	-	-0.006	0.074	9.31E-01
rs1264303	6	30990492	G/A	*VARS2*	UTR	2	0.649	0.549	0.768	4.70E-07	-0.001	0.033	9.70E-01	-0.128	0.122	2.98E-01	-0.069	0.033	**3.90E-02**	-0.079	0.122	5.19E-01	0.079	0.033	**1.70E-02**	-0.006	0.074	9.31E-01
rs1634761	6	31382006	G/A	*HLA-C | HLA-B*	INTERGENIC	2	1.498	1.280	1.754	4.71E-07	0.087	0.032	**6.20E-03**	-0.010	0.124	9.34E-01	-0.139	0.031	**9.60E-06**	-0.098	0.123	4.26E-01	0.227	0.032	**7.40E-13**	0.074	0.074	3.19E-01
rs2523608	6	31430537	G/A	*HLA-B*	INTRON	2	0.673	0.577	0.785	4.72E-07	-0.030	0.032	3.50E-01	-0.096	0.123	4.35E-01	-0.033	0.032	2.90E-01	-0.041	0.122	7.41E-01	0.008	0.033	7.90E-01	-0.001	0.075	9.84E-01
rs2252856	6	31001230	A/G	*VARS2*	INTRON	2	0.649	0.549	0.768	4.73E-07	-0.001	0.033	9.70E-01	-0.128	0.122	2.98E-01	-0.069	0.033	**3.90E-02**	-0.079	0.122	5.19E-01	0.079	0.033	**1.70E-02**	-0.006	0.074	9.31E-01
rs1264298	6	30992415	G/A	*VARS2*	INTRON	2	0.649	0.549	0.768	4.74E-07	-0.001	0.033	9.70E-01	-0.128	0.122	2.98E-01	-0.069	0.033	**3.90E-02**	-0.079	0.122	5.19E-01	0.079	0.033	**1.70E-02**	-0.006	0.074	9.31E-01
rs1264299	6	30991899	A/G	*VARS2*	INTRON	2	0.649	0.549	0.768	4.74E-07	-0.001	0.033	9.70E-01	-0.128	0.122	2.98E-01	-0.069	0.033	**3.90E-02**	-0.079	0.122	5.19E-01	0.079	0.033	**1.70E-02**	-0.006	0.074	9.31E-01
rs1264300	6	30990835	G/A	*VARS2*	INTRON	2	0.649	0.549	0.768	4.74E-07	-0.001	0.033	9.70E-01	-0.128	0.122	2.98E-01	-0.069	0.033	**3.90E-02**	-0.079	0.122	5.19E-01	0.079	0.033	**1.70E-02**	-0.006	0.074	9.31E-01
rs1264309	6	30983878	G/A	*DDR1 | GTF2H4*	INTERGENIC	2	0.649	0.549	0.768	4.74E-07	-0.001	0.033	9.70E-01	-0.128	0.122	2.98E-01	-0.069	0.033	**3.90E-02**	-0.079	0.122	5.19E-01	0.079	0.033	**1.70E-02**	-0.006	0.074	9.31E-01
rs2074506	6	30998462	A/C	*VARS2*	CODING	2	0.649	0.549	0.768	4.74E-07	-0.001	0.033	9.70E-01	-0.128	0.122	2.98E-01	-0.069	0.033	**3.90E-02**	-0.079	0.122	5.19E-01	0.079	0.033	**1.70E-02**	-0.006	0.074	9.31E-01
rs2517462	6	31004169	G/A	*VARS2 | SFTPG*	INTERGENIC	2	0.649	0.549	0.768	4.74E-07	-0.001	0.033	9.70E-01	-0.128	0.122	2.98E-01	-0.069	0.033	**3.90E-02**	-0.079	0.122	5.19E-01	0.079	0.033	**1.70E-02**	-0.006	0.074	9.31E-01
rs2517466	6	30998034	G/A	*VARS2*	INTRON	2	0.649	0.549	0.768	4.74E-07	-0.001	0.033	9.70E-01	-0.128	0.122	2.98E-01	-0.069	0.033	**3.90E-02**	-0.079	0.122	5.19E-01	0.079	0.033	**1.70E-02**	-0.006	0.074	9.31E-01
rs2532935	6	31002552	A/G	*VARS2 | SFTPG*	INTERGENIC	2	0.649	0.549	0.768	4.74E-07	-	-	-	-0.128	0.122	2.98E-01	-	-	-	-0.079	0.122	5.19E-01	-	-	-	-0.006	0.074	9.31E-01
rs2532938	6	31001810	A/G	*VARS2*	INTRON	2	0.649	0.549	0.768	4.74E-07	-	-	-	-0.128	0.122	2.98E-01	-	-	-	-0.079	0.122	5.19E-01	-	-	-	-0.006	0.074	9.31E-01
rs7738138	6	30995323	G/A	*VARS2*	INTRON	2	0.649	0.549	0.768	4.74E-07	-0.001	0.033	9.70E-01	-0.128	0.122	2.98E-01	-0.069	0.033	**3.90E-02**	-0.079	0.122	5.19E-01	0.079	0.033	**1.70E-02**	-0.006	0.074	9.31E-01
rs7756286	6	30995290	A/G	*VARS2*	INTRON	2	0.649	0.549	0.768	4.74E-07	-0.001	0.033	9.70E-01	-0.128	0.122	2.98E-01	-0.069	0.033	**3.90E-02**	-0.079	0.122	5.19E-01	0.079	0.033	**1.70E-02**	-0.006	0.074	9.31E-01
rs9262293	6	30994329	C/G	*VARS2*	INTRON	2	0.649	0.549	0.768	4.74E-07	-	-	-	-0.128	0.122	2.98E-01	-	-	-	-0.079	0.122	5.19E-01	-	-	-	-0.006	0.074	9.31E-01
rs1264301	6	30990760	A/C	*VARS2*	CODING	2	0.649	0.549	0.768	4.75E-07	-	-	-	-0.128	0.122	2.98E-01	-	-	-	-0.079	0.122	5.19E-01	-	-	-	-0.006	0.074	9.31E-01
rs2535339	6	30947409	A/G	*IER3 | DDR1*	INTERGENIC	2	0.649	0.549	0.768	4.75E-07	-	-	-	-0.128	0.122	2.98E-01	-	-	-	-0.079	0.122	5.19E-01	-	-	-	-0.006	0.074	9.31E-01
rs1264307	6	30988736	A/G	*GTF2H4*	INTRON	2	0.649	0.549	0.768	4.76E-07	-0.001	0.033	9.70E-01	-0.128	0.122	2.98E-01	-0.069	0.033	**3.90E-02**	-0.079	0.122	5.19E-01	0.079	0.033	**1.70E-02**	-0.006	0.074	9.31E-01
rs1264333	6	30952293	G/A	*IER3 | DDR1*	INTERGENIC	2	0.649	0.549	0.768	4.76E-07	-0.001	0.034	9.60E-01	-0.128	0.122	2.98E-01	-0.069	0.033	**3.70E-02**	-0.079	0.122	5.19E-01	0.081	0.033	**1.50E-02**	-0.006	0.074	9.31E-01
rs2844654	6	30946667	C/A	*IER3 | DDR1*	INTERGENIC	2	0.649	0.549	0.768	4.76E-07	-0.001	0.034	9.60E-01	-0.128	0.122	2.98E-01	-0.069	0.033	**3.70E-02**	-0.079	0.122	5.19E-01	0.081	0.033	**1.50E-02**	-0.006	0.074	9.31E-01
rs3130657	6	30945625	G/A	*IER3 | DDR1*	INTERGENIC	2	0.649	0.549	0.768	4.76E-07	-	-	-	-0.128	0.122	2.98E-01	-	-	-	-0.079	0.122	5.19E-01	-	-	-	-0.006	0.074	9.31E-01
rs9262289	6	30993665	T/A	*VARS2*	INTRON	2	0.650	0.549	0.768	4.81E-07	-	-	-	-0.128	0.122	2.98E-01	-	-	-	-0.079	0.122	5.19E-01	-	-	-	-0.006	0.074	9.31E-01
rs2844764	6	30342647	T/A	*FLJ45422*	UTR	1	1.540	1.302	1.822	4.89E-07	-0.002	0.033	9.40E-01	-0.132	0.123	2.86E-01	0.125	0.033	**1.20E-04**	0.037	0.123	7.64E-01	-0.127	0.033	**1.00E-04**	-0.174	0.073	**1.90E-02**
rs12214383	6	28331710	A/G	*ZKSCAN4 | NKAPL*	INTERGENIC	2	1.476	1.268	1.718	4.96E-07	0.015	0.032	6.40E-01	0.017	0.123	8.93E-01	0.061	0.032	5.40E-02	-0.147	0.121	2.28E-01	-0.049	0.032	1.21E-01	0.098	0.074	1.84E-01
rs3823417	6	31208848	A/G	*PSORS1C1 | PSORS1C2*	INTERGENIC	2	0.558	0.445	0.701	5.09E-07	-0.007	0.038	8.60E-01	0.028	0.123	8.18E-01	-0.092	0.038	**1.47E-02**	0.327	0.116	**6.12E-03**	0.103	0.038	**6.80E-03**	0.110	0.076	1.53E-01
rs241408	6	32970689	A/G	*PSMB9 | HLA-DMB*	INTERGENIC	2	0.661	0.562	0.777	5.10E-07	-0.066	0.032	**4.20E-02**	-0.084	0.123	4.97E-01	-0.019	0.032	5.60E-01	0.156	0.121	2.01E-01	-0.027	0.032	4.00E-01	0.049	0.074	5.08E-01
rs1736895	6	28327805	A/G	*ZKSCAN4*	UTR	2	1.476	1.268	1.717	5.11E-07	-	-	-	0.017	0.123	8.93E-01	-	-	-	-0.147	0.121	2.28E-01	-	-	-	0.098	0.074	1.84E-01
rs1150739	6	30139324	G/A	*ZNRD1*	INTRON	2	1.486	1.273	1.735	5.17E-07	-0.040	0.032	2.10E-01	-0.122	0.122	3.22E-01	0.125	0.032	**7.50E-05**	-0.096	0.122	4.31E-01	-0.166	0.032	**1.70E-07**	-0.087	0.074	2.42E-01
rs762815	6	32837620	C/G	*HLA-DQB2*	UTR	1	0.650	0.549	0.769	5.20E-07	-0.045	0.032	1.60E-01	0.254	0.121	**3.88E-02**	0.113	0.032	**4.00E-04**	0.258	0.120	**3.45E-02**	-0.165	0.032	**2.80E-07**	-0.092	0.074	2.15E-01
rs9276558	6	32832039	G/A	*HLA-DQB2*	UTR	1	0.650	0.549	0.769	5.20E-07	-0.045	0.032	1.60E-01	0.254	0.121	**3.88E-02**	0.113	0.032	**4.00E-04**	0.258	0.120	**3.45E-02**	-0.165	0.032	**2.80E-07**	-0.088	0.074	2.36E-01
rs2596501	6	31429190	A/G	*HLA-C | HLA-B*	INTERGENIC	2	0.671	0.575	0.784	5.28E-07	-0.008	0.032	8.00E-01	0.039	0.124	7.56E-01	-0.211	0.032	**2.80E-11**	0.057	0.123	6.43E-01	0.218	0.032	**7.00E-12**	0.052	0.075	4.90E-01
rs241399	6	32979536	A/G	*PSMB9 | HLA-DMB*	INTERGENIC	2	0.665	0.567	0.780	5.39E-07	-	-	-	-0.086	0.123	4.84E-01	-	-	-	0.122	0.121	3.17E-01	-	-	-	0.044	0.074	5.50E-01
rs241405	6	32973776	A/G	*PSMB9 | HLA-DMB*	INTERGENIC	2	0.665	0.567	0.780	5.42E-07	-0.066	0.032	**4.10E-02**	-0.086	0.123	4.84E-01	-0.025	0.032	4.30E-01	0.122	0.121	3.17E-01	-0.022	0.032	5.00E-01	0.044	0.074	5.50E-01
rs2532927	6	31006413	G/A	*VARS2 | SFTPG*	INTERGENIC	2	0.654	0.554	0.772	5.44E-07	-	-	-	-0.128	0.122	2.98E-01	-	-	-	-0.079	0.122	5.19E-01	-	-	-	-0.006	0.074	9.31E-01
rs2517550	6	31116347	G/A	*LOC729792*	INTRON	2	0.668	0.570	0.782	5.49E-07	0.030	0.033	3.60E-01	0.182	0.121	1.37E-01	-0.001	0.033	9.70E-01	-0.084	0.122	4.91E-01	0.021	0.033	5.20E-01	0.110	0.074	1.38E-01
rs853684	6	28402529	G/A	*ZNF323*	CODING	2	1.475	1.267	1.718	5.50E-07	0.038	0.032	2.40E-01	0.009	0.123	9.44E-01	0.059	0.032	6.30E-02	-0.140	0.121	2.50E-01	-0.027	0.032	4.10E-01	0.110	0.073	1.35E-01
rs9275334	6	32775085	G/A	*HLA-DQB1 | HLA-DQA2*	INTERGENIC	2	0.497	0.379	0.654	5.50E-07	0.046	0.051	3.70E-01	0.046	0.123	7.09E-01	0.011	0.050	8.20E-01	0.033	0.122	7.91E-01	0.018	0.051	7.30E-01	-0.018	0.074	8.13E-01
rs1116222	6	30179258	C/A	*TRIM31*	UTR	1	1.580	1.320	1.891	5.87E-07	0.023	0.035	5.00E-01	-0.108	0.123	3.81E-01	0.075	0.035	**2.90E-02**	-0.199	0.120	1.03E-01	-0.055	0.035	1.14E-01	0.047	0.074	5.27E-01
rs2517449	6	31027680	G/A	*DPCR1*	UTR	2	0.655	0.555	0.774	6.02E-07	0.008	0.033	8.00E-01	-0.133	0.122	2.81E-01	-0.064	0.033	**4.90E-02**	-0.115	0.122	3.47E-01	0.082	0.033	**1.26E-02**	0.026	0.074	7.30E-01
rs2532921	6	31030549	G/C	*LOC100129065*	UTR	2	0.656	0.555	0.774	6.03E-07	0.008	0.033	8.00E-01	-0.133	0.122	2.81E-01	-0.064	0.033	5.00E-02	-0.115	0.122	3.47E-01	0.082	0.033	**1.26E-02**	0.026	0.074	7.30E-01
rs7206	6	28309117	G/A	*ZNF193*	UTR	2	1.477	1.267	1.722	6.34E-07	0.026	0.032	4.20E-01	0.012	0.123	9.23E-01	0.060	0.032	5.90E-02	-0.132	0.121	2.80E-01	-0.037	0.032	2.50E-01	0.118	0.073	1.11E-01
rs4573120	6	30875926	C/A	*IER3 | DDR1*	INTERGENIC	2	0.643	0.541	0.765	6.50E-07	0.077	0.035	**2.60E-02**	-0.118	0.123	3.38E-01	-0.076	0.034	**2.80E-02**	-0.166	0.121	1.76E-01	0.162	0.035	**3.10E-06**	0.004	0.074	9.54E-01
rs4711232	6	30876391	A/G	*IER3 | DDR1*	INTERGENIC	2	0.643	0.541	0.765	6.50E-07	-	-	-	-0.118	0.123	3.38E-01	-	-	-	-0.166	0.121	1.76E-01	-	-	-	0.004	0.074	9.54E-01
rs4711233	6	30876405	G/A	*IER3 | DDR1*	INTERGENIC	2	0.643	0.541	0.765	6.50E-07	-	-	-	-0.118	0.123	3.38E-01	-	-	-	-0.166	0.121	1.76E-01	-	-	-	0.004	0.074	9.54E-01
rs4713372	6	30878757	G/C	*IER3 | DDR1*	INTERGENIC	2	0.643	0.541	0.765	6.50E-07	0.081	0.035	**2.00E-02**	-0.118	0.123	3.38E-01	-0.072	0.034	**3.60E-02**	-0.166	0.121	1.76E-01	0.160	0.034	**3.20E-06**	0.004	0.074	9.54E-01
rs9366759	6	30876934	G/A	*IER3 | DDR1*	INTERGENIC	2	0.643	0.541	0.765	6.50E-07	-	-	-	-0.118	0.123	3.38E-01	-	-	-	-0.166	0.121	1.76E-01	-	-	-	0.004	0.074	9.54E-01
rs2523705	6	31559659	A/G	*3.8-1 | MICB*	INTERGENIC	2	0.565	0.451	0.707	6.53E-07	0.122	0.038	**1.60E-03**	0.060	0.125	6.29E-01	-0.059	0.038	1.20E-01	0.038	0.124	7.61E-01	0.163	0.038	**2.20E-05**	0.028	0.075	7.11E-01
rs13408	6	28320727	A/G	*ZKSCAN4*	UTR	2	1.474	1.265	1.718	6.86E-07	-	-	-	0.021	0.123	8.66E-01	-	-	-	-0.158	0.121	1.95E-01	-	-	-	0.111	0.074	1.33E-01
rs276371	6	28018687	T/A	*OR2B2 | OR2B6*	INTERGENIC	2	1.470	1.263	1.712	7.05E-07	-	-	-	0.039	0.123	7.54E-01	-	-	-	0.034	0.122	7.84E-01	-	-	-	0.065	0.074	3.79E-01
rs1264331	6	30954809	A/C	*IER3 | DDR1*	INTERGENIC	2	0.657	0.557	0.776	7.15E-07	-0.001	0.034	9.60E-01	-0.128	0.122	2.98E-01	-0.069	0.033	**3.70E-02**	-0.079	0.122	5.19E-01	0.081	0.033	**1.50E-02**	-0.006	0.074	9.31E-01
rs4947248	6	31410004	G/A	*HLA-C | HLA-B*	INTERGENIC	1	0.550	0.434	0.697	7.21E-07	-	-	-	0.133	0.124	2.86E-01	-	-	-	0.111	0.124	3.74E-01	-	-	-	0.159	0.074	**3.40E-02**
rs2248372	6	31554445	A/G	*3.8-1 | MICB*	INTERGENIC	2	0.628	0.523	0.755	7.58E-07	0.073	0.034	**3.20E-02**	-0.070	0.123	5.69E-01	-0.105	0.034	**1.90E-03**	-0.081	0.122	5.11E-01	0.174	0.034	**2.90E-07**	0.026	0.075	7.27E-01
rs1264344	6	30908556	A/G	*IER3 | DDR1*	INTERGENIC	2	0.679	0.582	0.792	7.77E-07	-0.022	0.032	5.00E-01	-0.224	0.120	6.60E-02	-0.103	0.032	**1.10E-03**	-0.097	0.122	4.30E-01	0.096	0.032	**2.30E-03**	-0.010	0.074	8.94E-01
rs1150770	6	30491935	A/G	*RPP21 | HLA-E*	INTERGENIC	1	1.520	1.287	1.795	8.36E-07	-	-	-	-0.032	0.124	7.98E-01	-	-	-	0.001	0.123	9.92E-01	-	-	-	-0.036	0.074	6.25E-01
rs356966	6	30080963	A/G	*HCG9 | ZNRD1*	INTERGENIC	2	1.484	1.268	1.736	8.40E-07	-0.015	0.033	6.60E-01	-0.084	0.124	5.01E-01	0.146	0.033	**9.80E-06**	-0.049	0.123	6.93E-01	-0.168	0.033	**4.40E-07**	-0.103	0.074	1.64E-01
rs149974	6	28093075	G/A	*LOC442175 | ZNF165*	INTERGENIC	1	1.530	1.292	1.812	8.56E-07	0.041	0.032	2.00E-01	0.079	0.123	5.24E-01	0.070	0.032	**2.80E-02**	0.034	0.122	7.79E-01	-0.031	0.032	3.40E-01	0.068	0.074	3.60E-01
rs2596530	6	31495352	A/G	*LOC100129668 | HCP5*	INTERGENIC	2	0.681	0.584	0.794	8.67E-07	0.035	0.032	2.70E-01	0.057	0.125	6.50E-01	-0.167	0.032	**1.20E-07**	0.181	0.122	1.42E-01	0.208	0.032	**5.90E-11**	-0.069	0.075	3.61E-01
rs2844662	6	30925858	A/G	*IER3 | DDR1*	INTERGENIC	2	0.665	0.565	0.782	9.22E-07	-	-	-	-0.226	0.120	6.43E-02	-	-	-	-0.135	0.121	2.71E-01	-	-	-	-0.039	0.074	5.96E-01
rs1225715	6	28221352	G/A	*ZNF192*	INTRON	2	1.459	1.255	1.697	9.23E-07	0.033	0.032	3.00E-01	0.079	0.123	5.24E-01	0.063	0.032	**4.70E-02**	0.034	0.122	7.79E-01	-0.033	0.032	3.10E-01	0.094	0.074	2.03E-01
rs2844663	6	30923930	A/G	*IER3 | DDR1*	INTERGENIC	2	0.665	0.565	0.783	9.28E-07	-0.008	0.033	8.10E-01	-0.226	0.120	6.43E-02	-0.081	0.033	**1.34E-02**	-0.135	0.121	2.71E-01	0.087	0.033	**8.20E-03**	-0.039	0.074	5.96E-01
rs2516514	6	31555114	G/A	*3.8-1 | MICB*	INTERGENIC	2	0.561	0.445	0.707	9.41E-07	0.103	0.037	**5.80E-03**	0.075	0.123	5.43E-01	-0.062	0.037	9.70E-02	0.022	0.123	8.58E-01	0.149	0.037	**6.30E-05**	0.033	0.074	6.59E-01
rs2844702	6	31020460	A/G	*SFTPG | LOC100129065*	INTERGENIC	2	0.664	0.564	0.782	9.49E-07	0.003	0.033	9.40E-01	-0.153	0.122	2.13E-01	-0.071	0.033	**3.20E-02**	-0.090	0.122	4.60E-01	0.085	0.033	**1.03E-02**	0.010	0.074	8.91E-01
rs203878	6	28156975	C/A	*ZNF165*	UTR	2	1.459	1.254	1.697	9.52E-07	0.035	0.032	2.80E-01	0.079	0.123	5.24E-01	0.067	0.032	**3.50E-02**	0.034	0.122	7.79E-01	-0.034	0.032	2.80E-01	0.094	0.074	2.03E-01
rs2248462	6	31554775	A/G	*3.8-1 | MICB*	INTERGENIC	2	0.574	0.460	0.717	9.56E-07	0.122	0.038	**1.60E-03**	0.075	0.123	5.43E-01	-0.059	0.038	1.20E-01	0.022	0.123	8.58E-01	0.163	0.038	**2.20E-05**	0.033	0.075	6.63E-01
rs2535326	6	30940042	A/G	*IER3 | DDR1*	INTERGENIC	2	0.665	0.565	0.783	9.67E-07	-0.012	0.033	7.20E-01	-0.226	0.120	6.43E-02	-0.085	0.033	**9.20E-03**	-0.135	0.121	2.71E-01	0.088	0.033	**7.50E-03**	-0.039	0.074	5.96E-01

rs9275599	6	32790407	A/G	*HLA-DQB1 | HLA-DQA2*	INTERGENIC	2	0.493	0.371	0.654	9.70E-07	0.053	0.051	3.00E-01	0.046	0.123	7.09E-01	0.015	0.051	7.60E-01	0.033	0.122	7.91E-01	0.019	0.051	7.10E-01	0.005	0.074	9.41E-01
rs1264334	6	30952239	A/G	*IER3 | DDR1*	INTERGENIC	2	0.666	0.565	0.783	9.99E-07	-0.012	0.033	7.20E-01	-0.226	0.120	6.43E-02	-0.085	0.033	**9.20E-03**	-0.135	0.121	2.71E-01	0.088	0.033	**7.50E-03**	-0.039	0.074	5.96E-01
rs156737	6	28003192	G/A	*OR2B2 | OR2B6*	INTERGENIC	2	1.457	1.252	1.694	1.07E-06	0.029	0.032	3.70E-01	0.039	0.123	7.54E-01	0.069	0.032	**3.20E-02**	0.034	0.122	7.84E-01	-0.039	0.032	2.30E-01	0.065	0.074	3.79E-01
rs9461406	6	27827743	G/A	*ZNF184 | LOC100131289*	INTERGENIC	2	1.497	1.273	1.760	1.07E-06	0.042	0.037	2.60E-01	0.120	0.123	3.33E-01	0.139	0.037	**1.70E-04**	0.013	0.123	9.17E-01	-0.105	0.037	**5.00E-03**	0.005	0.075	9.51E-01
rs1042147	6	31191135	G/A	*CDSN*	UTR	2	0.675	0.576	0.790	1.11E-06	0.054	0.031	8.50E-02	0.046	0.124	7.15E-01	-0.169	0.031	**4.70E-08**	0.137	0.123	2.70E-01	0.226	0.031	**3.70E-13**	0.022	0.075	7.69E-01
rs3094214	6	31193361	A/C	*CDSN*	INTRON	2	0.675	0.576	0.790	1.11E-06	0.054	0.031	8.50E-02	0.046	0.124	7.15E-01	-0.169	0.031	**4.70E-08**	0.137	0.123	2.70E-01	0.226	0.031	**3.70E-13**	0.022	0.075	7.69E-01
rs1042126	6	31192267	G/A	*CDSN*	CODING	2	0.675	0.576	0.790	1.12E-06	-	-	-	0.046	0.124	7.15E-01	-	-	-	0.137	0.123	2.70E-01	-	-	-	0.022	0.075	7.69E-01
rs1042134	6	31191643	A/G	*CDSN*	UTR	2	0.675	0.576	0.790	1.12E-06	0.054	0.031	8.50E-02	0.046	0.124	7.15E-01	-0.169	0.031	**4.70E-08**	0.137	0.123	2.70E-01	0.226	0.031	**3.70E-13**	0.022	0.075	7.69E-01
rs1042141	6	31191445	C/G	*CDSN*	UTR	2	0.675	0.576	0.790	1.12E-06	0.054	0.031	8.50E-02	0.046	0.124	7.15E-01	-0.169	0.031	**4.70E-08**	0.137	0.123	2.70E-01	0.226	0.031	**3.70E-13**	0.022	0.075	7.69E-01
rs1042145	6	31191307	G/A	*CDSN*	UTR	2	0.675	0.576	0.790	1.12E-06	-	-	-	0.046	0.124	7.15E-01	-	-	-	0.137	0.123	2.70E-01	-	-	-	0.022	0.075	7.69E-01
rs2523933	6	30040271	A/C	*HLA-A | HCG9*	INTERGENIC	1	1.550	1.299	1.849	1.12E-06	-0.009	0.034	7.90E-01	-0.031	0.123	8.03E-01	0.137	0.034	**4.70E-05**	-0.044	0.123	7.23E-01	-0.155	0.034	**4.80E-06**	-0.092	0.074	2.12E-01
rs3094217	6	31191635	A/G	*CDSN*	UTR	2	0.675	0.576	0.790	1.12E-06	0.054	0.031	8.50E-02	0.046	0.124	7.15E-01	-0.169	0.031	**4.70E-08**	0.137	0.123	2.70E-01	0.226	0.031	**3.70E-13**	0.022	0.075	7.69E-01
rs3094219	6	31191265	A/G	*CDSN*	UTR	2	0.675	0.576	0.790	1.12E-06	0.052	0.031	9.80E-02	0.046	0.124	7.15E-01	-0.168	0.031	**5.20E-08**	0.137	0.123	2.70E-01	0.224	0.031	**6.60E-13**	0.022	0.075	7.69E-01
rs3130555	6	31193526	C/G	*CDSN*	INTRON	2	0.675	0.576	0.790	1.12E-06	-	-	-	0.046	0.124	7.15E-01	-	-	-	0.137	0.123	2.70E-01	-	-	-	0.022	0.075	7.69E-01
rs3130983	6	31192771	A/G	*CDSN*	CODING	2	0.675	0.576	0.790	1.12E-06	0.055	0.031	8.00E-02	0.046	0.124	7.15E-01	-0.168	0.031	**6.20E-08**	0.137	0.123	2.70E-01	0.225	0.031	**5.10E-13**	0.019	0.075	8.04E-01
rs9468203	6	27796646	G/A	*ZNF184 | LOC100131289*	INTERGENIC	1	1.550	1.299	1.849	1.12E-06	0.043	0.037	2.50E-01	0.103	0.123	4.04E-01	0.121	0.037	**9.80E-04**	0.017	0.123	8.93E-01	-0.083	0.037	**2.50E-02**	-0.010	0.075	8.94E-01
rs259933	6	30113284	A/G	*HCG9 | ZNRD1*	INTERGENIC	2	1.477	1.262	1.728	1.15E-06	-0.001	0.032	9.80E-01	-0.084	0.124	5.01E-01	0.125	0.032	**8.70E-05**	-0.049	0.123	6.93E-01	-0.130	0.032	**5.10E-05**	-0.103	0.074	1.64E-01
rs259938	6	30115982	C/G	*HCG9 | ZNRD1*	INTERGENIC	2	1.477	1.262	1.728	1.16E-06	-0.015	0.033	6.60E-01	-0.084	0.124	5.01E-01	0.146	0.033	**9.80E-06**	-0.049	0.123	6.93E-01	-0.168	0.033	**4.40E-07**	-0.103	0.074	1.64E-01
rs259939	6	30119560	G/A	*HCG9 | ZNRD1*	INTERGENIC	2	1.477	1.262	1.728	1.16E-06	-0.015	0.033	6.60E-01	-0.084	0.124	5.01E-01	0.146	0.033	**9.80E-06**	-0.049	0.123	6.93E-01	-0.168	0.033	**4.40E-07**	-0.103	0.074	1.64E-01
rs1150707	6	28305584	A/G	*ZNF193*	INTRON	2	1.462	1.254	1.703	1.17E-06	0.026	0.032	4.20E-01	0.012	0.123	9.23E-01	0.060	0.032	5.90E-02	-0.132	0.121	2.80E-01	-0.037	0.032	2.50E-01	0.165	0.073	**2.52E-02**
rs259935	6	30115030	A/G	*HCG9 | ZNRD1*	INTERGENIC	2	1.476	1.262	1.727	1.17E-06	-0.015	0.033	6.60E-01	-0.084	0.124	5.01E-01	0.146	0.033	**9.80E-06**	-0.049	0.123	6.93E-01	-0.168	0.033	**4.40E-07**	-0.103	0.074	1.64E-01
rs259919	6	30133482	A/G	*HCG9 | ZNRD1*	INTERGENIC	2	1.468	1.257	1.713	1.20E-06	0.008	0.032	8.10E-01	-0.056	0.123	6.49E-01	0.121	0.032	**1.70E-04**	-0.037	0.123	7.65E-01	-0.129	0.032	**6.60E-05**	-0.093	0.074	2.07E-01
rs2523591	6	31434939	A/G	*HLA-B | LOC729816*	INTERGENIC	1	0.640	0.534	0.766	1.21E-06	-	-	-	-0.038	0.124	7.59E-01	-	-	-	0.117	0.123	3.43E-01	-	-	-	-0.095	0.076	2.15E-01
rs6917363	6	31355148	G/A	*HLA-C | HLA-B*	INTERGENIC	1	0.640	0.534	0.766	1.21E-06	-	-	-	0.150	0.123	2.28E-01	-	-	-	0.125	0.123	3.10E-01	-	-	-	0.112	0.074	1.31E-01
rs149976	6	28095755	G/A	*LOC442175 | ZNF165*	INTERGENIC	2	1.453	1.249	1.690	1.23E-06	0.041	0.032	2.00E-01	0.079	0.123	5.24E-01	0.070	0.032	**2.80E-02**	0.034	0.122	7.79E-01	-0.031	0.032	3.40E-01	0.068	0.074	3.60E-01
rs9275532	6	32783612	G/C	*HLA-DQB1 | HLA-DQA2*	INTERGENIC	2	0.515	0.393	0.673	1.23E-06	-	-	-	0.046	0.123	7.09E-01	-	-	-	0.033	0.122	7.91E-01	-	-	-	-0.016	0.074	8.31E-01
rs9275495	6	32781552	T/A	*HLA-DQB1 | HLA-DQA2*	INTERGENIC	2	0.515	0.394	0.673	1.24E-06	-	-	-	0.046	0.123	7.09E-01	-	-	-	0.033	0.122	7.91E-01	-	-	-	-0.016	0.074	8.31E-01
rs2523857	6	31129483	A/G	*LOC729792 | HCG22*	INTERGENIC	2	0.674	0.574	0.790	1.25E-06	0.028	0.033	3.90E-01	0.107	0.123	3.86E-01	0.029	0.033	3.70E-01	-0.145	0.121	2.37E-01	-0.015	0.033	6.50E-01	0.011	0.074	8.85E-01
rs3823363	6	30051694	A/G	*HCG9*	INTRON	2	1.459	1.252	1.700	1.25E-06	-0.023	0.031	4.50E-01	-0.184	0.121	1.32E-01	0.056	0.031	6.90E-02	-0.094	0.122	4.45E-01	-0.076	0.031	**1.46E-02**	-0.132	0.073	7.34E-02
rs2106070	6	30294842	G/A	*TRIM26 | FLJ45422*	INTERGENIC	1	1.530	1.288	1.817	1.28E-06	-	-	-	0.126	0.123	3.10E-01	-	-	-	0.030	0.123	8.11E-01	-	-	-	0.103	0.074	1.68E-01
rs2516509	6	31557973	G/A	*3.8-1 | MICB*	INTERGENIC	2	0.567	0.450	0.713	1.31E-06	0.122	0.038	**1.60E-03**	0.075	0.123	5.43E-01	-0.059	0.038	1.20E-01	0.022	0.123	8.58E-01	0.163	0.038	**2.20E-05**	0.033	0.074	6.59E-01
rs1225710	6	28208619	G/A	*ZSCAN16 | LOC100129195*	INTERGENIC	2	1.451	1.248	1.688	1.33E-06	0.037	0.032	2.50E-01	0.079	0.123	5.24E-01	0.067	0.032	**3.40E-02**	0.034	0.122	7.79E-01	-0.033	0.032	3.10E-01	0.094	0.074	2.03E-01
rs3131886	6	29772431	G/A	*ZFP57 | HLA-F*	INTERGENIC	2	1.445	1.244	1.678	1.39E-06	0.001	0.032	9.70E-01	-0.176	0.121	1.50E-01	0.028	0.032	3.70E-01	0.055	0.122	6.56E-01	-0.025	0.032	4.30E-01	-0.040	0.074	5.86E-01
rs417162	6	30024484	G/A	*HLA-A | HCG9*	INTERGENIC	1	1.510	1.277	1.786	1.46E-06	-0.066	0.033	**4.20E-02**	0.051	0.124	6.80E-01	0.153	0.032	**2.00E-06**	-0.253	0.119	**3.78E-02**	-0.228	0.032	**2.20E-12**	-0.015	0.075	8.43E-01
rs1966	6	31215712	A/G	*PSORS1C1*	UTR	2	0.619	0.510	0.753	1.51E-06	0.111	0.041	**6.30E-03**	0.001	0.123	9.93E-01	-0.149	0.040	**2.20E-04**	0.223	0.119	6.52E-02	0.258	0.040	**1.90E-10**	-0.027	0.074	7.19E-01
rs2853923	6	31373716	G/A	*HLA-C | HLA-B*	INTERGENIC	1	0.670	0.569	0.789	1.51E-06	0.020	0.032	5.30E-01	-0.036	0.123	7.71E-01	-0.214	0.031	**9.40E-12**	0.137	0.121	2.64E-01	0.256	0.032	**4.90E-16**	-0.127	0.074	8.67E-02
rs3129769	6	32705000	A/G	*HLA-DRB1 | HLA-DQA1*	INTERGENIC	1	0.550	0.431	0.702	1.52E-06	-	-	-	0.091	0.123	4.63E-01	-	-	-	0.012	0.123	9.26E-01	-	-	-	0.019	0.074	7.98E-01
rs2516511	6	31556604	A/G	*3.8-1 | MICB*	INTERGENIC	2	0.574	0.457	0.720	1.55E-06	0.122	0.038	**1.60E-03**	0.060	0.125	6.29E-01	-0.059	0.038	1.20E-01	0.038	0.124	7.61E-01	0.163	0.038	**2.20E-05**	0.028	0.075	7.11E-01
rs1225618	6	28237692	C/A	*ZNF192 | LOC222699*	INTERGENIC	2	1.450	1.246	1.688	1.58E-06	0.033	0.032	3.00E-01	0.079	0.123	5.24E-01	0.063	0.032	**4.70E-02**	0.034	0.122	7.79E-01	-0.033	0.032	3.10E-01	0.080	0.074	2.77E-01
rs1165189	6	25957758	C/A	*SLC17A3*	INTRON	2	1.495	1.269	1.761	1.59E-06	-0.048	0.037	1.90E-01	0.003	0.123	9.81E-01	0.030	0.036	4.10E-01	-0.082	0.122	5.01E-01	-0.077	0.037	**3.50E-02**	0.081	0.074	2.76E-01
rs9357155	6	32917826	A/G	*PSMB8*	INTRON	2	0.517	0.394	0.677	1.62E-06	-0.057	0.049	2.50E-01	0.089	0.125	4.77E-01	-0.078	0.048	1.06E-01	-0.064	0.124	6.11E-01	0.016	0.049	7.40E-01	-0.009	0.074	9.05E-01
rs6921589	6	25530348	A/C	*LRRC16*	INTRON	2	1.585	1.313	1.914	1.66E-06	0.070	0.045	1.16E-01	-0.080	0.124	5.22E-01	0.066	0.044	1.36E-01	-0.056	0.123	6.49E-01	-0.012	0.045	7.80E-01	-0.112	0.074	1.33E-01
rs213237	6	28431917	G/A	*ZNF323 | ZKSCAN3*	INTERGENIC	2	1.444	1.243	1.679	1.68E-06	0.043	0.032	1.80E-01	0.001	0.123	9.94E-01	0.057	0.032	7.50E-02	-0.162	0.121	1.85E-01	-0.019	0.032	5.50E-01	0.118	0.073	1.09E-01
rs2395178	6	32513340	G/C	*BTNL2 | HLA-DRA*	INTERGENIC	1	0.610	0.498	0.747	1.76E-06	-0.073	0.034	**3.20E-02**	0.068	0.123	5.80E-01	0.038	0.034	2.60E-01	0.181	0.120	1.37E-01	-0.099	0.034	**3.50E-03**	-0.116	0.073	1.17E-01
rs2517582	6	30916741	A/G	*IER3 | DDR1*	INTERGENIC	2	0.673	0.572	0.792	1.83E-06	-	-	-	-0.188	0.121	1.25E-01	-	-	-	-0.115	0.122	3.49E-01	-	-	-	-0.030	0.074	6.91E-01
rs3998159	6	32789997	C/A	*HLA-DQB1 | HLA-DQA2*	INTERGENIC	2	0.507	0.384	0.671	1.91E-06	0.052	0.051	3.10E-01	0.046	0.123	7.09E-01	0.019	0.051	7.00E-01	0.033	0.122	7.91E-01	0.014	0.051	7.90E-01	-0.017	0.074	8.16E-01
rs149971	6	28090131	A/G	*LOC442175 | ZNF165*	INTERGENIC	2	1.446	1.242	1.683	1.95E-06	0.041	0.032	2.00E-01	0.089	0.123	4.69E-01	0.070	0.032	**2.80E-02**	0.045	0.122	7.11E-01	-0.031	0.032	3.40E-01	0.067	0.074	3.63E-01
rs2844479	6	31680935	C/A	*NCR3 | AIF1*	INTERGENIC	2	0.658	0.553	0.782	1.95E-06	-0.004	0.035	9.00E-01	0.062	0.123	6.14E-01	-0.141	0.034	**3.70E-05**	0.093	0.122	4.50E-01	0.144	0.034	**2.70E-05**	0.009	0.074	9.01E-01
rs9272353	6	32712434	C/G	*HLA-DRB1 | HLA-DQA1*	INTERGENIC	1	0.560	0.441	0.711	2.03E-06	-	-	-	0.099	0.124	4.27E-01	-	-	-	0.002	0.124	9.88E-01	-	-	-	0.027	0.074	7.16E-01
rs9275530	6	32783501	C/G	*HLA-DQB1 | HLA-DQA2*	INTERGENIC	2	0.515	0.392	0.678	2.19E-06	-	-	-	0.046	0.123	7.09E-01	-	-	-	0.033	0.122	7.91E-01	-	-	-	-0.016	0.074	8.31E-01
rs3957146	6	32789508	G/A	*HLA-DQB1 | HLA-DQA2*	INTERGENIC	2	0.516	0.392	0.678	2.23E-06	0.051	0.052	3.20E-01	0.046	0.123	7.09E-01	0.019	0.051	7.10E-01	0.033	0.122	7.91E-01	0.013	0.051	7.90E-01	-0.016	0.074	8.31E-01
rs1165158	6	25972877	A/C	*SLC17A3*	INTRON	2	1.487	1.262	1.753	2.24E-06	-0.043	0.037	2.50E-01	0.003	0.123	9.81E-01	0.031	0.036	3.90E-01	-0.082	0.122	5.01E-01	-0.074	0.037	**4.40E-02**	0.081	0.074	2.75E-01
rs1531681	6	28334857	G/A	*ZKSCAN4 | NKAPL*	INTERGENIC	2	1.444	1.240	1.681	2.27E-06	0.022	0.032	4.80E-01	0.017	0.123	8.93E-01	0.062	0.032	**4.90E-02**	-0.147	0.121	2.28E-01	-0.046	0.032	1.50E-01	0.098	0.074	1.84E-01
rs10182181	2	25003800	G/A	*ADCY3 | RBJ*	INTERGENIC	2	1.437	1.236	1.669	2.32E-06	0.013	0.032	7.00E-01	0.083	0.124	5.08E-01	-0.030	0.032	3.50E-01	0.038	0.124	7.60E-01	0.040	0.032	2.20E-01	0.046	0.074	5.33E-01
rs6752378	2	25003620	A/C	*ADCY3 | RBJ*	INTERGENIC	2	1.437	1.236	1.669	2.32E-06	0.013	0.032	7.00E-01	0.083	0.124	5.08E-01	-0.030	0.032	3.50E-01	0.038	0.124	7.60E-01	0.040	0.032	2.20E-01	0.046	0.074	5.33E-01
rs9394113	6	32773145	G/A	*HLA-DQB1 | HLA-DQA2*	INTERGENIC	2	0.603	0.489	0.744	2.33E-06	0.120	0.037	**1.16E-03**	-0.009	0.123	9.45E-01	-0.004	0.037	9.10E-01	0.096	0.122	4.34E-01	0.113	0.037	**2.20E-03**	-0.008	0.074	9.16E-01
rs2531803	6	28519199	C/A	*ZSCAN23*	UTR	2	1.422	1.228	1.647	2.48E-06	-	-	-	0.064	0.123	6.05E-01	-	-	-	-0.203	0.120	9.45E-02	-	-	-	0.132	0.073	7.26E-02
rs2531825	6	28457243	A/G	*ZKSCAN3 | ZSCAN12*	INTERGENIC	2	1.433	1.234	1.665	2.53E-06	0.041	0.032	2.10E-01	0.001	0.123	9.94E-01	0.062	0.032	5.20E-02	-0.162	0.121	1.85E-01	-0.027	0.032	4.00E-01	0.118	0.073	1.09E-01
rs9263869	6	31277993	G/A	*HCG27*	INTRON	2	0.671	0.568	0.792	2.62E-06	-	-	-	0.013	0.123	9.18E-01	-	-	-	0.015	0.122	9.03E-01	-	-	-	0.015	0.074	8.39E-01
rs2523873	6	31120242	C/G	*LOC729792*	INTRON	2	0.677	0.575	0.797	2.64E-06	0.031	0.033	3.60E-01	0.182	0.121	1.37E-01	0.000	0.033	1.00E+00	-0.084	0.122	4.91E-01	0.020	0.033	5.40E-01	0.110	0.074	1.38E-01
rs2736176	6	31695540	C/G	*AIF1 | BAT2*	INTERGENIC	2	0.657	0.552	0.783	2.78E-06	0.010	0.036	7.80E-01	0.091	0.123	4.63E-01	-0.099	0.036	**5.10E-03**	-0.077	0.123	5.31E-01	0.108	0.036	**2.50E-03**	0.067	0.074	3.65E-01
rs1264297	6	30992785	C/A	*VARS2*	INTRON	2	0.666	0.562	0.790	2.97E-06	-0.001	0.033	9.70E-01	-0.128	0.122	2.98E-01	-0.069	0.033	**3.90E-02**	-0.079	0.122	5.19E-01	0.079	0.033	**1.70E-02**	-0.006	0.074	9.31E-01
rs3830076	6	32204222	A/G	*CREBL1 | FKBPL*	INTERGENIC	2	0.458	0.330	0.636	3.21E-06	0.116	0.066	7.60E-02	0.021	0.124	8.68E-01	0.019	0.065	7.70E-01	0.074	0.123	5.50E-01	0.083	0.065	2.00E-01	-0.116	0.073	1.15E-01
rs916403	6	28516237	G/A	*ZSCAN23*	INTRON	2	1.422	1.226	1.650	3.44E-06	0.008	0.032	8.10E-01	0.064	0.123	6.05E-01	0.109	0.032	**5.40E-04**	-0.203	0.120	9.45E-02	-0.111	0.032	**4.60E-04**	0.132	0.073	7.26E-02
rs1265045	6	31193319	A/G	*CDSN*	INTRON	2	0.615	0.501	0.755	3.50E-06	-	-	-	0.148	0.122	2.28E-01	-	-	-	-0.064	0.122	6.02E-01	-	-	-	0.025	0.074	7.31E-01
rs1165162	6	25971584	A/G	*SLC17A3*	INTRON	2	1.478	1.253	1.744	3.54E-06	-0.043	0.037	2.50E-01	0.003	0.123	9.81E-01	0.031	0.036	3.90E-01	-0.082	0.122	5.01E-01	-0.074	0.037	**4.40E-02**	0.083	0.074	2.63E-01
rs1182814	6	25967533	G/A	*SLC17A3*	INTRON	2	1.478	1.253	1.744	3.57E-06	-0.043	0.037	2.50E-01	0.003	0.123	9.81E-01	0.031	0.036	3.90E-01	-0.082	0.122	5.01E-01	-0.074	0.037	**4.40E-02**	0.083	0.074	2.63E-01
rs2517538	6	31121520	G/C	*LOC729792 | HCG22*	INTERGENIC	2	0.681	0.578	0.801	3.62E-06	0.021	0.033	5.20E-01	0.204	0.120	9.44E-02	0.009	0.033	7.80E-01	-0.084	0.122	4.92E-01	0.002	0.033	9.50E-01	0.037	0.074	6.17E-01
rs2517552	6	31115569	G/A	*LOC729792*	CODING	2	0.681	0.578	0.801	3.62E-06	0.021	0.033	5.30E-01	0.204	0.120	9.44E-02	0.009	0.033	7.90E-01	-0.084	0.122	4.92E-01	0.002	0.033	9.50E-01	0.037	0.074	6.17E-01

rs2523870	6	31122095	G/A	*LOC729792 | HCG22*	INTERGENIC	2	0.681	0.578	0.801	3.62E-06	0.024	0.033	4.70E-01	0.204	0.120	9.44E-02	0.014	0.033	6.80E-01	-0.084	0.122	4.92E-01	-0.001	0.033	9.80E-01	0.037	0.074	6.17E-01
rs1165164	6	25971460	A/G	*SLC17A3*	INTRON	2	1.479	1.252	1.747	4.02E-06	-0.045	0.037	2.20E-01	0.038	0.123	7.57E-01	0.027	0.037	4.60E-01	-0.086	0.122	4.84E-01	-0.072	0.037	5.10E-02	0.091	0.074	2.20E-01
rs3134939	6	32277599	T/A	*NOTCH4*	INTRON	2	0.701	0.602	0.815	4.09E-06	-	-	-	0.061	0.124	6.22E-01	-	-	-	-0.117	0.123	3.42E-01	-	-	-	-0.053	0.075	4.84E-01
rs1052215	6	28456137	A/C	*ZKSCAN3 | ZSCAN12*	INTERGENIC	2	1.422	1.224	1.652	4.13E-06	0.041	0.032	2.10E-01	0.001	0.123	9.94E-01	0.062	0.032	5.20E-02	-0.162	0.121	1.85E-01	-0.027	0.032	4.00E-01	0.118	0.073	1.09E-01
rs1265085	6	31218650	G/C	*CCHCR1*	INTRON	2	0.621	0.507	0.761	4.27E-06	0.111	0.041	**6.30E-03**	0.001	0.123	9.93E-01	-0.149	0.040	**2.20E-04**	0.223	0.119	6.52E-02	0.258	0.040	**1.90E-10**	-0.027	0.074	7.19E-01
rs2524222	6	30619149	A/G	*HLA-E | GNL1*	INTERGENIC	2	0.601	0.484	0.747	4.32E-06	-0.029	0.037	4.30E-01	0.211	0.121	8.66E-02	-0.069	0.036	5.80E-02	0.138	0.122	2.63E-01	0.043	0.037	2.40E-01	0.068	0.074	3.61E-01
rs9268473	6	32463661	G/A	*C6orf10 | BTNL2*	INTERGENIC	1	0.650	0.541	0.781	4.39E-06	0.031	0.032	3.30E-01	-0.027	0.123	8.26E-01	-0.094	0.032	**3.30E-03**	0.098	0.122	4.25E-01	0.129	0.032	**6.30E-05**	0.020	0.074	7.87E-01
rs9348859	6	31357956	A/G	*HLA-C | HLA-B*	INTERGENIC	2	0.687	0.585	0.807	4.64E-06	-	-	-	-0.067	0.124	5.92E-01	-	-	-	0.082	0.123	5.06E-01	-	-	-	-0.069	0.074	3.55E-01
rs2284190	6	32927495	G/A	*TAP1*	INTRON	2	0.537	0.411	0.701	4.80E-06	-0.068	0.047	1.49E-01	0.089	0.125	4.77E-01	-0.085	0.046	6.60E-02	-0.064	0.124	6.11E-01	0.015	0.047	7.50E-01	-0.005	0.074	9.44E-01
rs2844480	6	31672800	A/G	*NCR3 | AIF1*	INTERGENIC	1	0.570	0.448	0.726	5.25E-06	0.034	0.041	4.10E-01	0.061	0.123	6.24E-01	-0.132	0.040	**1.06E-03**	0.077	0.122	5.33E-01	0.166	0.040	**4.20E-05**	-0.107	0.074	1.52E-01
rs166327	6	30110860	G/A	*HCG9 | ZNRD1*	INTERGENIC	2	1.428	1.225	1.665	5.50E-06	-0.025	0.031	4.20E-01	-0.185	0.121	1.32E-01	0.112	0.031	**3.00E-04**	-0.110	0.122	3.68E-01	-0.136	0.031	**1.30E-05**	-0.096	0.074	1.93E-01
rs1165148	6	25952689	A/C	*SLC17A1 | SLC17A3*	INTERGENIC	2	1.470	1.245	1.735	5.56E-06	-0.048	0.037	1.90E-01	0.003	0.123	9.81E-01	0.030	0.036	4.10E-01	-0.082	0.122	5.01E-01	-0.077	0.037	**3.50E-02**	0.083	0.074	2.63E-01
rs5027459	6	32827006	G/A	*HLA-DQA2 | HLA-DQB2*	INTERGENIC	1	0.630	0.516	0.769	5.62E-06	-	-	-	-0.230	0.120	6.08E-02	-	-	-	-0.095	0.122	4.39E-01	-	-	-	0.002	0.074	9.83E-01
rs130067	6	31226490	C/A	*CCHCR1*	CODING	2	0.629	0.515	0.769	5.89E-06	0.111	0.041	**6.30E-03**	0.001	0.123	9.93E-01	-0.149	0.040	**2.20E-04**	0.223	0.119	6.52E-02	0.258	0.040	**1.90E-10**	-0.035	0.074	6.39E-01
rs755714	6	31717792	A/G	*BAT3*	INTRON	1	0.640	0.528	0.776	5.97E-06	0.010	0.036	7.80E-01	0.053	0.124	6.71E-01	-0.096	0.036	**6.90E-03**	-0.081	0.123	5.14E-01	0.108	0.036	**2.50E-03**	0.055	0.074	4.61E-01
rs194675	6	33013724	A/T	*HLA-DMB*	INTRON	2	0.710	0.610	0.825	8.42E-06	0.036	0.032	2.60E-01	-0.048	0.123	6.97E-01	-0.017	0.031	5.80E-01	-0.076	0.122	5.33E-01	0.042	0.032	1.80E-01	0.067	0.074	3.63E-01
rs3132569	6	31209405	A/G	*PSORS1C1 | PSORS1C2*	INTERGENIC	2	0.639	0.524	0.780	9.83E-06	0.104	0.040	**8.70E-03**	0.075	0.123	5.43E-01	-0.142	0.039	**3.00E-04**	-0.089	0.122	4.65E-01	0.240	0.040	**1.30E-09**	-0.015	0.074	8.36E-01
rs150359	6	33030046	A/T	*HLA-DMA | BRD2*	INTERGENIC	2	1.409	1.210	1.641	1.02E-05	0.032	0.031	3.00E-01	0.097	0.124	4.37E-01	0.016	0.031	6.10E-01	0.057	0.124	6.46E-01	0.011	0.031	7.30E-01	-0.024	0.074	7.49E-01
rs2516448	6	31498389	G/A	*LOC100129668 | HCP5*	INTERGENIC	2	0.696	0.593	0.818	1.04E-05	0.035	0.032	2.70E-01	0.057	0.125	6.50E-01	-0.167	0.032	**1.20E-07**	0.181	0.122	1.42E-01	0.208	0.032	**5.90E-11**	-0.069	0.075	3.61E-01
rs2853977	6	31487283	T/A	*LOC100129668*	INTRON	2	0.696	0.593	0.818	1.04E-05	0.037	0.032	2.50E-01	0.057	0.125	6.50E-01	-0.167	0.032	**1.30E-07**	0.181	0.122	1.42E-01	0.209	0.032	**5.00E-11**	-0.069	0.075	3.61E-01
rs707919	6	31749118	G/A	*LY6G5B | LY6G5C*	INTERGENIC	2	0.667	0.557	0.799	1.04E-05	0.009	0.036	8.00E-01	0.056	0.123	6.52E-01	-0.096	0.036	**6.90E-03**	-0.078	0.122	5.24E-01	0.107	0.036	**2.70E-03**	0.056	0.074	4.48E-01
rs805297	6	31730585	A/C	*BAT3 | APOM*	INTERGENIC	2	0.667	0.557	0.799	1.04E-05	0.010	0.036	7.80E-01	0.056	0.123	6.52E-01	-0.096	0.036	**6.90E-03**	-0.078	0.122	5.24E-01	0.108	0.036	**2.50E-03**	0.056	0.074	4.48E-01
rs2857211	6	32848420	A/G	*HLA-DQB2 | HLA-DOB*	INTERGENIC	2	0.680	0.573	0.808	1.06E-05	-0.079	0.033	**1.70E-02**	-0.175	0.122	1.57E-01	0.000	0.033	9.90E-01	0.061	0.123	6.20E-01	-0.074	0.033	**2.70E-02**	-0.054	0.074	4.70E-01
rs2844498	6	31584833	A/G	*MICB*	INTRON	2	0.693	0.588	0.816	1.08E-05	0.027	0.032	4.00E-01	0.030	0.124	8.08E-01	-0.162	0.031	**2.50E-07**	-0.018	0.123	8.87E-01	0.193	0.032	**1.00E-09**	0.050	0.074	5.01E-01
rs8084	6	32519013	C/A	*HLA-DRA*	CODING	2	0.705	0.604	0.824	1.12E-05	-0.058	0.032	6.70E-02	0.072	0.123	5.57E-01	0.114	0.032	**2.90E-04**	0.235	0.119	5.18E-02	-0.174	0.032	**4.40E-08**	0.058	0.074	4.37E-01
rs2253907	6	31444849	G/A	*LOC729816 | LOC100129668*	INTERGENIC	2	0.704	0.600	0.825	1.52E-05	-0.098	0.032	**2.50E-03**	0.034	0.124	7.84E-01	-0.018	0.032	5.80E-01	0.108	0.122	3.81E-01	-0.069	0.032	**3.20E-02**	0.057	0.074	4.41E-01
rs3104404	6	32790152	A/C	*HLA-DQB1 | HLA-DQA2*	INTERGENIC	2	0.572	0.443	0.737	1.62E-05	0.145	0.045	**1.36E-03**	-0.135	0.122	2.71E-01	0.032	0.045	4.70E-01	0.117	0.121	3.37E-01	0.087	0.045	5.30E-02	-0.049	0.074	5.05E-01
rs2844521	6	31476943	A/G	*LOC729816 | LOC100129668*	INTERGENIC	2	0.665	0.551	0.803	2.23E-05	0.052	0.035	1.39E-01	0.063	0.124	6.15E-01	-0.148	0.035	**2.00E-05**	0.177	0.122	1.50E-01	0.200	0.035	**9.70E-09**	0.012	0.075	8.76E-01
rs9276160	6	32806399	A/G	*HLA-DQB1 | HLA-DQA2*	INTERGENIC	2	0.683	0.572	0.815	2.33E-05	-0.070	0.037	5.80E-02	-0.243	0.120	**4.62E-02**	0.103	0.037	**4.80E-03**	-0.110	0.122	3.71E-01	-0.181	0.037	**8.30E-07**	0.007	0.074	9.26E-01
rs9275765	6	32797302	A/T	*HLA-DQB1 | HLA-DQA2*	INTERGENIC	2	0.683	0.572	0.815	2.37E-05	-0.076	0.037	**3.90E-02**	-0.243	0.120	**4.62E-02**	0.096	0.037	**8.80E-03**	-0.110	0.122	3.71E-01	-0.182	0.037	**7.40E-07**	0.007	0.074	9.26E-01
rs9276296	6	32810908	G/A	*HLA-DQB1 | HLA-DQA2*	INTERGENIC	2	0.683	0.572	0.815	2.38E-05	-0.070	0.037	5.80E-02	-0.243	0.120	**4.62E-02**	0.103	0.037	**4.80E-03**	-0.110	0.122	3.71E-01	-0.181	0.037	**8.30E-07**	0.007	0.074	9.26E-01
rs2853973	6	31461971	C/G	*LOC729816 | LOC100129668*	INTERGENIC	2	0.667	0.553	0.805	2.44E-05	0.054	0.035	1.25E-01	0.063	0.124	6.15E-01	-0.160	0.035	**3.70E-06**	0.177	0.122	1.50E-01	0.213	0.035	**9.60E-10**	0.012	0.075	8.76E-01
rs2428486	6	31462083	G/A	*LOC729816 | LOC100129668*	INTERGENIC	2	0.668	0.553	0.806	2.50E-05	0.054	0.035	1.25E-01	0.063	0.124	6.15E-01	-0.160	0.035	**3.70E-06**	0.177	0.122	1.50E-01	0.213	0.035	**9.60E-10**	0.012	0.075	8.76E-01
rs2844529	6	31461572	A/G	*LOC729816 | LOC100129668*	INTERGENIC	2	0.668	0.553	0.806	2.51E-05	0.054	0.035	1.25E-01	0.063	0.124	6.15E-01	-0.160	0.035	**3.70E-06**	0.177	0.122	1.50E-01	0.213	0.035	**9.60E-10**	0.012	0.075	8.76E-01
rs2429657	6	30579499	G/A	*HLA-E | GNL1*	INTERGENIC	2	0.635	0.513	0.786	3.01E-05	-0.043	0.036	2.30E-01	0.181	0.122	1.44E-01	-0.100	0.036	**5.10E-03**	0.131	0.123	2.88E-01	0.067	0.036	6.10E-02	0.041	0.074	5.80E-01
rs9275653	6	32793843	G/A	*HLA-DQB1 | HLA-DQA2*	INTERGENIC	2	0.710	0.602	0.836	4.11E-05	-0.033	0.032	3.10E-01	-0.212	0.120	8.21E-02	0.026	0.032	4.20E-01	0.045	0.122	7.15E-01	-0.062	0.032	5.30E-02	-0.032	0.074	6.66E-01
rs8512	6	30819336	A/G	*IER3*	UTR	2	0.611	0.482	0.773	4.23E-05	-0.063	0.042	1.31E-01	0.241	0.120	**4.93E-02**	-0.058	0.042	1.60E-01	0.136	0.122	2.71E-01	0.006	0.042	8.80E-01	0.036	0.074	6.23E-01
rs7755596	6	32814456	G/A	*HLA-DQB1 | HLA-DQA2*	INTERGENIC	2	0.678	0.562	0.817	4.48E-05	-0.077	0.037	**3.80E-02**	-0.230	0.120	6.08E-02	0.101	0.037	**5.80E-03**	-0.095	0.122	4.39E-01	-0.185	0.037	**5.50E-07**	0.007	0.074	9.26E-01
rs3129736	6	32793931	G/A	*HLA-DQB1 | HLA-DQA2*	INTERGENIC	2	0.686	0.572	0.823	4.70E-05	-0.076	0.037	**3.90E-02**	-0.243	0.120	**4.62E-02**	0.096	0.037	**8.80E-03**	-0.110	0.122	3.71E-01	-0.182	0.037	**7.40E-07**	0.007	0.074	9.26E-01
rs2523453	6	31476104	C/G	*LOC729816 | LOC100129668*	INTERGENIC	2	0.677	0.561	0.817	4.75E-05	0.051	0.035	1.42E-01	0.063	0.124	6.15E-01	-0.147	0.035	**2.20E-05**	0.177	0.122	1.50E-01	0.199	0.035	**1.20E-08**	0.012	0.075	8.76E-01
rs2428475	6	31474601	G/A	*LOC729816 | LOC100129668*	INTERGENIC	2	0.677	0.561	0.817	4.87E-05	0.048	0.035	1.70E-01	0.063	0.124	6.15E-01	-0.148	0.035	**1.90E-05**	0.177	0.122	1.50E-01	0.197	0.035	**1.40E-08**	0.012	0.075	8.76E-01
rs2523467	6	31470909	A/G	*LOC729816 | LOC100129668*	INTERGENIC	2	0.678	0.561	0.818	4.93E-05	0.054	0.035	1.21E-01	0.063	0.124	6.15E-01	-0.159	0.035	**4.40E-06**	0.177	0.122	1.50E-01	0.212	0.035	**1.10E-09**	0.012	0.075	8.76E-01
rs2523475	6	31469689	G/A	*LOC729816 | LOC100129668*	INTERGENIC	2	0.678	0.562	0.818	4.94E-05	0.054	0.035	1.21E-01	0.063	0.124	6.15E-01	-0.159	0.035	**4.40E-06**	0.177	0.122	1.50E-01	0.212	0.035	**1.10E-09**	0.012	0.075	8.76E-01

Supplementary Table S15. Pleiotropy assessment of genetic variants of non-LS (ref. 17) in T-lymphocytes (CD4 and CD8 relative counts) and CD4/CD8 ratio in blood of the healthy (ref. 22) and BAL of non-LS cases (unpublished data)

							Fixed effects model (ref. 17)				Blood CD4 (in healthy)			BAL CD4 (in diseased)			Blood CD8 (in healthy)			BAL CD8 (in diseased)			Blood CD4/CD8 (in healthy)			BAL CD4/CD8 (in diseased)		
SNP	CHR	BP	CA/NCA	Gene Symbol	Gene Location	Num. Cohorts	OR	L95	U95	Meta-P	Beta	SE	P	Beta	SE	P	Beta	SE	P	Beta	SE	P	Beta	SE	P	Beta	SE	P
rs1964995	6	32557389	G/A	*HLA-DRA | HLA-DRB5*	INTERGENIC	5	0.601	0.552	0.654	**2.53E-32**	-	-	-	-0.03321	0.1097	7.63E-01	-	-	-	0.1295	0.1086	2.36E-01	-	-	-	0.1817	0.06083	**3.09E-03**
rs2213585	6	32521128	G/A	*HLA-DRA | HLA-DRB5*	INTERGENIC	3	1.505	1.387	1.633	**8.82E-23**	-0.098	0.032	**2.50E-03**	0.02025	0.1081	8.52E-01	0.133	0.032	**3.20E-05**	-0.06565	0.1077	5.44E-01	-0.228	0.032	**1.40E-12**	-0.1582	0.06046	**9.37E-03**
rs2227139	6	32521437	G/A	*HLA-DRA | HLA-DRB5*	INTERGENIC	3	1.505	1.387	1.632	**8.96E-23**	-0.098	0.032	**2.60E-03**	0.02025	0.1081	8.52E-01	0.135	0.032	**2.60E-05**	-0.06565	0.1077	5.44E-01	-0.23	0.032	**1.10E-12**	-0.1582	0.06046	**9.37E-03**
rs2213586	6	32521072	A/G	*HLA-DRA | HLA-DRB5*	INTERGENIC	3	1.502	1.385	1.630	**1.29E-22**	-0.098	0.032	**2.50E-03**	0.02025	0.1081	8.52E-01	0.133	0.032	**3.20E-05**	-0.06565	0.1077	5.44E-01	-0.228	0.032	**1.40E-12**	-0.1582	0.06046	**9.37E-03**
rs7192	6	32519624	A/C	*HLA-DRA*	CODING	3	1.503	1.385	1.631	**1.31E-22**	-0.098	0.032	**2.50E-03**	-	-	-	0.133	0.032	**3.20E-05**	-	-	-	-0.228	0.032	**1.40E-12**	-	-	-
rs2395153	6	32453573	C/G	*C6orf10 | BTNL2*	INTERGENIC	3	0.632	0.577	0.693	**1.76E-22**	0.038	0.032	2.40E-01	0.01609	0.107	8.81E-01	-0.092	0.032	**4.20E-03**	0.07806	0.1065	4.65E-01	0.13	0.032	**5.40E-05**	0.1627	0.06053	**7.65E-03**
rs7195	6	32520517	A/G	*HLA-DRA*	UTR	3	1.501	1.384	1.629	**1.77E-22**	-0.098	0.032	**2.50E-03**	0.02025	0.1081	8.52E-01	0.133	0.032	**3.20E-05**	-0.06565	0.1077	5.44E-01	-0.228	0.032	**1.40E-12**	-0.1582	0.06046	**9.37E-03**
rs3763327	6	32521808	G/C	*HLA-DRA | HLA-DRB5*	INTERGENIC	2	1.568	1.420	1.732	**7.36E-19**	-0.096	0.032	**3.10E-03**	0.02025	0.1081	8.52E-01	0.129	0.032	**5.90E-05**	-0.06565	0.1077	5.44E-01	-0.221	0.032	**7.70E-12**	-0.1582	0.06046	**9.37E-03**
rs477515	6	32677669	A/G	*HLA-DRB1 | HLA-DQA1*	INTERGENIC	3	0.638	0.577	0.706	**3.93E-18**	-	-	-	-0.05423	0.1088	6.20E-01	-	-	-	-0.07636	0.1085	4.83E-01	-	-	-	0.2212	0.06026	**2.93E-04**
rs2395182	6	32521295	C/A	*HLA-DRA | HLA-DRB5*	INTERGENIC	3	1.485	1.357	1.625	**7.05E-18**	-0.192	0.039	**6.30E-07**	0.01955	0.1064	8.55E-01	0.133	0.038	**4.70E-04**	-0.189	0.1043	7.33E-02	-0.308	0.038	**1.00E-15**	-0.0304	0.06113	6.19E-01
rs4373382	6	32458846	C/A	*C6orf10 | BTNL2*	INTERGENIC	3	0.686	0.629	0.748	**1.17E-17**	-	-	-	-0.02866	0.1074	7.90E-01	-	-	-	0.03954	0.1072	7.13E-01	-	-	-	0.1039	0.06121	9.08E-02
rs3129952	6	32467741	C/G	*C6orf10 | BTNL2*	INTERGENIC	2	1.640	1.464	1.838	**1.54E-17**	-0.118	0.034	**5.20E-04**	0.0921	0.1068	3.91E-01	0.118	0.034	**4.50E-04**	-0.1341	0.106	2.09E-01	-0.23	0.034	**1.00E-11**	0.03191	0.0613	6.03E-01
rs4424066	6	32462406	G/A	*C6orf10 | BTNL2*	INTERGENIC	3	0.686	0.629	0.748	**1.73E-17**	0.031	0.032	3.30E-01	-0.03259	0.1067	7.61E-01	-0.094	0.032	**3.30E-03**	0.03562	0.1065	7.39E-01	0.129	0.032	**6.30E-05**	0.1039	0.06088	8.92E-02
rs2076529	6	32471933	G/A	*BTNL2*	CODING	3	0.687	0.630	0.749	**1.74E-17**	0.031	0.032	3.30E-01	-0.03259	0.1067	7.61E-01	-0.094	0.032	**3.30E-03**	0.03562	0.1065	7.39E-01	0.129	0.032	**6.30E-05**	0.1039	0.06088	8.92E-02
rs3817973	6	32469089	A/G	*C6orf10 | BTNL2*	INTERGENIC	3	0.687	0.630	0.749	**1.96E-17**	0.031	0.032	3.30E-01	-0.03259	0.1067	7.61E-01	-0.094	0.032	**3.30E-03**	0.03562	0.1065	7.39E-01	0.129	0.032	**6.30E-05**	0.1039	0.06088	8.92E-02
rs2239802	6	32519824	C/G	*HLA-DRA*	INTRON	3	1.476	1.349	1.615	**1.97E-17**	-0.192	0.039	**6.30E-07**	0.01955	0.1064	8.55E-01	0.133	0.038	**4.70E-04**	-0.189	0.1043	7.33E-02	-0.308	0.038	**1.00E-15**	-0.0304	0.06113	6.19E-01
rs9268472	6	32463583	A/G	*C6orf10 | BTNL2*	INTERGENIC	3	0.690	0.632	0.752	**3.84E-17**	-	-	-	-0.03259	0.1067	7.61E-01	-	-	-	0.03562	0.1065	7.39E-01	-	-	-	0.1039	0.06088	8.92E-02
rs3177928	6	32520413	A/G	*HLA-DRA*	UTR	3	0.533	0.460	0.618	**8.37E-17**	0.041	0.046	3.70E-01	0.02724	0.107	8.00E-01	-0.049	0.045	2.70E-01	0.2206	0.1042	**3.71E-02**	0.079	0.045	8.10E-02	-0.03798	0.06118	5.35E-01
rs2076530	6	32471794	G/A	*BTNL2*	CODING	3	0.694	0.637	0.757	**1.21E-16**	0.031	0.032	3.30E-01	-0.04282	0.1072	6.90E-01	-0.094	0.032	**3.30E-03**	0.0377	0.107	7.25E-01	0.129	0.032	**6.30E-05**	0.09328	0.06102	1.28E-01
rs9268969	6	32542327	A/G	*HLA-DRA | HLA-DRB5*	INTERGENIC	3	0.659	0.597	0.727	**1.29E-16**	0.094	0.032	**3.60E-03**	-0.03342	0.1096	7.61E-01	-0.028	0.032	3.70E-01	0.005004	0.1094	9.64E-01	0.113	0.032	**4.00E-04**	0.2155	0.06022	**4.09E-04**
rs3129888	6	32519704	G/A	*HLA-DRA*	INTRON	3	1.480	1.348	1.625	**2.07E-16**	-0.192	0.039	**6.30E-07**	0.03153	0.1064	7.68E-01	0.133	0.038	**4.70E-04**	-0.182	0.1044	8.49E-02	-0.308	0.038	**1.00E-15**	-0.02512	0.06113	6.82E-01
rs3129948	6	32462622	C/A	*C6orf10 | BTNL2*	INTERGENIC	3	1.445	1.323	1.577	**2.30E-16**	-0.119	0.035	**5.60E-04**	0.09238	0.1062	3.87E-01	0.128	0.034	**1.80E-04**	-0.1335	0.1055	2.09E-01	-0.242	0.034	**1.70E-12**	0.0303	0.06108	6.20E-01
rs2294878	6	32475773	A/C	*BTNL2*	INTRON	3	0.699	0.642	0.762	**3.66E-16**	-	-	-	-0.1204	0.1057	2.58E-01	-	-	-	-0.0366	0.1062	7.31E-01	-	-	-	0.07947	0.06097	1.94E-01
rs3117098	6	32466491	G/A	*C6orf10 | BTNL2*	INTERGENIC	3	1.436	1.315	1.568	**6.96E-16**	-0.119	0.035	**5.60E-04**	0.09238	0.1062	3.87E-01	0.128	0.034	**1.80E-04**	-0.1335	0.1055	2.09E-01	-0.242	0.034	**1.70E-12**	0.0303	0.06108	6.20E-01
rs3129961	6	32486918	G/A	*BTNL2 | HLA-DRA*	INTERGENIC	3	1.431	1.311	1.562	**1.02E-15**	-	-	-	0.09238	0.1062	3.87E-01	-	-	-	-0.1335	0.1055	2.09E-01	-	-	-	0.0303	0.06108	6.20E-01
rs3129954	6	32473558	A/G	*BTNL2*	INTRON	3	1.431	1.311	1.563	**1.36E-15**	-0.119	0.035	**5.60E-04**	0.09238	0.1062	3.87E-01	0.128	0.034	**1.80E-04**	-0.1335	0.1055	2.09E-01	-0.242	0.034	**1.70E-12**	0.0303	0.06108	6.20E-01
rs3129955	6	32473818	A/G	*BTNL2*	INTRON	3	1.433	1.312	1.566	**1.55E-15**	-	-	-	0.0949	0.1068	3.77E-01	-	-	-	-0.1318	0.1061	2.18E-01	-	-	-	0.02218	0.06216	7.22E-01
rs9268882	6	32539601	A/G	*HLA-DRA | HLA-DRB5*	INTERGENIC	2	1.501	1.356	1.663	**5.94E-15**	-0.102	0.034	**2.60E-03**	0.1055	0.1058	3.22E-01	0.1	0.033	**2.90E-03**	-0.1396	0.1052	1.88E-01	-0.193	0.034	**9.60E-09**	0.000374	0.06113	9.95E-01
rs6913309	6	32447818	A/T	*C6orf10 | BTNL2*	INTERGENIC	3	0.670	0.605	0.741	**1.09E-14**	0.05	0.037	1.80E-01	-0.02418	0.1068	8.21E-01	-0.124	0.036	**6.80E-04**	0.1913	0.1046	7.08E-02	0.179	0.037	**1.10E-06**	0.02373	0.06129	6.99E-01
rs3104389	6	32703075	A/C	*HLA-DRB1 | HLA-DQA1*	INTERGENIC	3	0.672	0.608	0.744	**1.21E-14**	-	-	-	-0.02112	0.1093	8.47E-01	-	-	-	0.009282	0.1091	9.32E-01	-	-	-	0.2201	0.06021	**3.09E-04**
rs7197	6	32520558	A/G	*HLA-DRA*	UTR	3	1.472	1.334	1.625	**1.37E-14**	-0.215	0.039	**4.80E-08**	0.0719	0.1069	5.03E-01	0.133	0.039	**6.80E-04**	-0.1477	0.1057	1.66E-01	-0.329	0.039	**5.40E-17**	-0.02478	0.06143	6.87E-01
rs3129727	6	32787668	A/G	*HLA-DQB1 | HLA-DQA2*	INTERGENIC	3	2.341	1.877	2.920	**4.36E-14**	-0.063	0.11	5.70E-01	0.09115	0.1106	4.12E-01	-0.268	0.109	**1.39E-02**	0.1162	0.1102	2.94E-01	0.24	0.109	**2.80E-02**	-0.07834	0.06223	2.09E-01
rs9271588	6	32698931	G/A	*HLA-DRB1 | HLA-DQA1*	INTERGENIC	3	0.725	0.666	0.789	**9.50E-14**	-	-	-	0.005218	0.1071	9.61E-01	-	-	-	0.1559	0.1056	1.43E-01	-	-	-	0.1468	0.06068	**1.62E-02**
rs3129882	6	32517508	G/A	*HLA-DRA*	INTRON	3	1.347	1.243	1.460	**4.18E-13**	-0.081	0.032	**1.23E-02**	0.02108	0.1065	8.44E-01	0.08	0.032	**1.17E-02**	-0.155	0.105	1.44E-01	-0.154	0.032	**1.60E-06**	-0.02041	0.0611	7.39E-01
rs2076536	6	32447326	G/A	*C6orf10*	INTRON	2	1.447	1.309	1.599	**4.84E-13**	-0.118	0.034	**5.40E-04**	0.03384	0.1089	7.57E-01	0.036	0.034	2.80E-01	-0.1477	0.1073	1.73E-01	-0.143	0.034	**2.40E-05**	-0.07391	0.06146	2.30E-01
rs3129886	6	32518554	A/G	*HLA-DRA*	INTRON	3	1.380	1.264	1.506	**6.77E-13**	-0.127	0.036	**5.00E-04**	0.03588	0.1065	7.37E-01	0.097	0.036	**7.40E-03**	-0.1563	0.105	1.40E-01	-0.213	0.036	**4.60E-09**	-0.02796	0.06119	6.48E-01
rs2395185	6	32541145	A/C	*HLA-DRA | HLA-DRB5*	INTERGENIC	3	0.699	0.634	0.771	**7.46E-13**	0.095	0.034	**4.60E-03**	-0.03342	0.1096	7.61E-01	-0.112	0.033	**7.30E-04**	0.005004	0.1094	9.64E-01	0.205	0.033	**8.20E-10**	0.2155	0.06022	**4.09E-04**
rs9268923	6	32540813	A/G	*HLA-DRA | HLA-DRB5*	INTERGENIC	3	0.700	0.635	0.772	**7.76E-13**	0.094	0.032	**3.60E-03**	-0.03342	0.1096	7.61E-01	-0.028	0.032	3.70E-01	0.005004	0.1094	9.64E-01	0.113	0.032	**4.00E-04**	0.2155	0.06022	**4.09E-04**
rs9268853	6	32537621	G/A	*HLA-DRA | HLA-DRB5*	INTERGENIC	3	0.700	0.635	0.772	**7.82E-13**	0.096	0.034	**4.30E-03**	-0.03342	0.1096	7.61E-01	-0.107	0.033	**1.20E-03**	0.005004	0.1094	9.64E-01	0.201	0.033	**1.90E-09**	0.2155	0.06022	**4.09E-04**
rs9405108	6	32546626	A/G	*HLA-DRA | HLA-DRB5*	INTERGENIC	3	0.700	0.635	0.772	**8.32E-13**	-	-	-	-0.03342	0.1096	7.61E-01	-	-	-	0.005004	0.1094	9.64E-01	-	-	-	0.2155	0.06022	**4.09E-04**
rs9271640	6	32700178	A/G	*HLA-DRB1 | HLA-DQA1*	INTERGENIC	2	1.461	1.316	1.622	**1.18E-12**	-	-	-	0.0679	0.1063	5.25E-01	-	-	-	-0.1428	0.1052	1.78E-01	-	-	-	-0.03124	0.06165	6.13E-01
rs9368726	6	32546520	G/A	*HLA-DRA | HLA-DRB5*	INTERGENIC	3	0.702	0.637	0.774	**1.20E-12**	-	-	-	-0.03342	0.1096	7.61E-01	-	-	-	0.005004	0.1094	9.64E-01	-	-	-	0.2155	0.06022	**4.09E-04**
rs1794265	6	32782715	C/G	*HLA-DQB1 | HLA-DQA2*	INTERGENIC	2	2.403	1.880	3.071	**2.58E-12**	-0.063	0.11	5.70E-01	0.09115	0.1106	4.12E-01	-0.268	0.109	**1.39E-02**	0.1162	0.1102	2.94E-01	0.24	0.109	**2.80E-02**	-0.07855	0.06223	2.08E-01
rs9271775	6	32702306	G/A	*HLA-DRB1 | HLA-DQA1*	INTERGENIC	2	1.461	1.313	1.626	**3.24E-12**	-0.212	0.041	**2.30E-07**	0.0679	0.1063	5.25E-01	0.166	0.04	**4.20E-05**	-0.1428	0.1052	1.78E-01	-0.359	0.041	**1.20E-18**	-0.02224	0.06117	7.17E-01
rs3830135	6	32656442	A/G	*HLA-DRB1*	INTRON	3	0.556	0.471	0.657	**5.30E-12**	-	-	-	-0.00306	0.1065	9.77E-01	-	-	-	0.2047	0.104	5.22E-02	-	-	-	-0.05102	0.06103	4.04E-01
rs1953600	10	81901705	A/G	*PLAC9 | ANXA11*	INTERGENIC	3	0.733	0.670	0.802	**1.34E-11**	-0.001	0.032	9.60E-01	-0.05117	0.11	6.43E-01	-0.004	0.031	9.10E-01	0.04746	0.1098	6.67E-01	0.011	0.032	7.20E-01	-0.07032	0.06115	2.51E-01
rs9391858	6	32449376	G/A	*C6orf10 | BTNL2*	INTERGENIC	3	0.622	0.542	0.715	**2.03E-11**	0.038	0.046	4.10E-01	0.009164	0.1073	9.32E-01	-0.067	0.045	1.41E-01	0.2	0.1049	5.99E-02	0.096	0.045	**3.60E-02**	-0.01839	0.06129	7.64E-01
rs1049550	10	81916682	A/G	*ANXA11*	CODING	3	0.733	0.669	0.803	**2.61E-11**	0.002	0.032	9.60E-01	-0.0528	0.1102	6.33E-01	-0.007	0.032	8.30E-01	0.06372	0.11	5.64E-01	0.017	0.032	5.90E-01	-0.1095	0.06085	7.32E-02
rs17533167	6	32698822	G/C	*HLA-DRB1 | HLA-DQA1*	INTERGENIC	3	0.571	0.484	0.673	**2.86E-11**	-0.051	0.049	3.00E-01	-0.00306	0.1065	9.77E-01	-0.046	0.049	3.40E-01	0.2047	0.104	5.22E-02	-0.006	0.049	9.00E-01	-0.04513	0.06124	4.62E-01
rs11754183	6	32674134	A/G	*HLA-DRB1 | HLA-DQA1*	INTERGENIC	2	1.847	1.539	2.217	**4.50E-11**	-	-	-	-	-	-	-	-	-	-	-	-	-	-	-	-	-	-
rs9273363	6	32734250	A/C	*HLA-DQA1 | HLA-DQB1*	INTERGENIC	3	0.713	0.644	0.790	**9.72E-11**	0.093	0.036	**8.70E-03**	0.07808	0.1062	4.64E-01	0.082	0.035	**1.90E-02**	0.165	0.1048	1.19E-01	-0.012	0.035	7.30E-01	0.0932	0.06089	1.27E-01
rs17422797	6	32373506	A/G	*C6orf10*	INTRON	3	0.485	0.389	0.604	**1.03E-10**	-0.079	0.067	2.40E-01	0.05018	0.1064	6.38E-01	0.01	0.066	8.80E-01	0.2697	0.1023	**9.91E-03**	-0.071	0.067	2.90E-01	-0.1104	0.06073	7.02E-02
rs3129768	6	32703061	C/A	*HLA-DRB1 | HLA-DQA1*	INTERGENIC	2	1.415	1.273	1.573	**1.17E-10**	-0.195	0.04	**1.10E-06**	0.0679	0.1063	5.25E-01	0.178	0.04	**7.30E-06**	-0.1428	0.1052	1.78E-01	-0.36	0.04	**1.50E-19**	-0.02498	0.06115	6.83E-01
rs2573346	10	81908021	A/G	*ANXA11*	INTRON	3	0.745	0.680	0.815	**1.90E-10**	-0.001	0.032	9.60E-01	-0.05117	0.11	6.43E-01	-0.004	0.031	9.10E-01	0.04746	0.1098	6.67E-01	0.011	0.032	7.20E-01	-0.07032	0.06115	2.51E-01
rs9268832	6	32535767	A/G	*HLA-DRA | HLA-DRB5*	INTERGENIC	3	1.294	1.194	1.403	**3.90E-10**	-0.091	0.032	**4.70E-03**	0.0298	0.1079	7.83E-01	0.139	0.032	**1.40E-05**	-0.06997	0.1074	5.17E-01	-0.23	0.032	**9.70E-13**	-0.1534	0.06049	**1.18E-02**
rs4530903	6	32689867	A/G	*HLA-DRB1 | HLA-DQA1*	INTERGENIC	3	0.598	0.507	0.705	**8.29E-10**	-0.075	0.052	1.49E-01	-0.00306	0.1065	9.77E-01	-0.068	0.051	1.80E-01	0.2047	0.104	5.22E-02	0.002	0.052	9.60E-01	-0.05102	0.06103	4.04E-01
rs9271488	6	32696978	A/C	*HLA-DRB1 | HLA-DQA1*	INTERGENIC	2	0.683	0.604	0.771	**8.81E-10**	-	-	-	-0.02112	0.1093	8.47E-01	-	-	-	0.009282	0.1091	9.32E-01	-	-	-	0.2201	0.06021	**3.09E-04**
rs9268473	6	32463661	G/A	*C6orf10 | BTNL2*	INTERGENIC	1	0.637	0.551	0.736	**1.06E-09**	0.031	0.032	3.30E-01	-0.03405	0.1066	7.50E-01	-0.094	0.032	**3.30E-03**	0.03232	0.1064	7.62E-01	0.129	0.032	**6.30E-05**	0.1059	0.06085	8.29E-02
rs9269110	6	32551247	A/C	*HLA-DRA | HLA-DRB5*	INTERGENIC	3	1.294	1.190	1.406	**1.25E-09**	-	-	-	0.1272	0.106	2.33E-01	-	-	-	-0.09929	0.1055	3.49E-01	-	-	-	-0.01267	0.0617	8.38E-01
rs13209234	6	32523953	A/G	*HLA-DRA | HLA-DRB5*	INTERGENIC	2	0.575	0.481	0.688	**1.49E-09**	0.036	0.046	4.30E-01	0.02724	0.107	8.00E-01	-0.05	0.045	2.70E-01	0.2206	0.1042	**3.71E-02**	0.074	0.045	1.03E-01	-0.03184	0.06133	6.04E-01
rs9269081	6	32549078	A/C	*HLA-DRA | HLA-DRB5*	INTERGENIC	3	1.291	1.187	1.405	**2.43E-09**	-0.134	0.035	**1.10E-04**	0.1055	0.1058	3.22E-01	0.086	0.034	**1.17E-02**	-0.1396	0.1052	1.88E-01	-0.205	0.034	**2.50E-09**	0.003326	0.06112	9.57E-01
rs6903608	6	32536263	G/A	*HLA-DRA | HLA-DRB5*	INTERGENIC	3	1.284	1.182	1.395	**3.00E-09**	-0.125	0.034	**2.60E-04**	0.1055	0.1058	3.22E-01	0.097	0.034	**4.00E-03**	-0.1396	0.1052	1.88E-01	-0.211	0.034	**5.60E-10**	0.000374	0.06113	9.95E-01
rs17496549	6	32517686	A/G	*HLA-DRA*	INTRON	3	0.627	0.537	0.732	**3.79E-09**	-0.044	0.052	4.00E-01	-0.00348	0.1065	9.74E-01	-0.024	0.051	6.40E-01	0.1942	0.1042	6.58E-02	-0.024	0.052	6.50E-01	-0.04516	0.06109	4.60E-01
rs17496307	6	32509014	T/A	*BTNL2 | HLA-DRA*	INTERGENIC	3	0.629	0.539	0.734	**4.05E-09**	-0.047	0.052	3.60E-01	-0.02246	0.1066	8.34E-01	-0.028	0.051	5.80E-01	0.1708	0.1048	1.07E-01	-0.023	0.052	6.60E-01	-0.01744	0.06128	7.76E-01
rs6923504	6	32536164	G/C	*HLA-DRA | HLA-DRB5*	INTERGENIC	3	1.280	1.178	1.391	**4.99E-09**	-0.125	0.034	**2.60E-04**	0.1055	0.1058	3.22E-01	0.097	0.034	**4.00E-03**	-0.1396	0.1052	1.88E-01	-0.211	0.034	**5.60E-10**	0.000374	0.06113	9.95E-01
rs3998158	6	32789970	G/A	*HLA-DQB1 | HLA-DQA2*	INTERGENIC	3	0.725	0.650	0.809	**7.46E-09**	-0.002	0.039	9.60E-01	-0.05591	0.1077	6.05E-01	-0.026	0.038	5.00E-01	0.04878	0.1076	6.51E-01	0.016	0.038	6.70E-01	0.1022	0.06105	9.52E-02

rs6930777	6	32459544	A/G	*C6orf10 | BTNL2*	INTERGENIC	3	0.642	0.550	0.750	**2.29E-08**	-0.07	0.052	1.80E-01	0.01503	0.1064	8.88E-01	-0.017	0.052	7.40E-01	0.2172	0.1037	**3.90E-02**	-0.057	0.052	2.70E-01	-0.05264	0.06104	3.89E-01
rs522308	6	32689900	A/G	*HLA-DRB1 | HLA-DQA1*	INTERGENIC	2	0.698	0.615	0.792	**2.57E-08**	0.09	0.034	**7.50E-03**	-0.05423	0.1088	6.20E-01	-0.105	0.033	**1.70E-03**	-0.07636	0.1085	4.83E-01	0.192	0.034	**1.20E-08**	0.2205	0.06018	**3.00E-04**
rs3135388	6	32521029	A/G	*HLA-DRA | HLA-DRB5*	INTERGENIC	3	1.408	1.247	1.590	**3.31E-08**	-0.249	0.043	**5.50E-09**	0.1165	0.1058	2.74E-01	0.182	0.042	**1.70E-05**	-0.1168	0.1056	2.71E-01	-0.412	0.042	**3.00E-22**	0.00124	0.0612	9.84E-01
rs3129889	6	32521523	G/A	*HLA-DRA | HLA-DRB5*	INTERGENIC	3	1.393	1.234	1.572	7.85E-08	-0.25	0.043	**4.70E-09**	0.1165	0.1058	2.74E-01	0.181	0.042	**1.80E-05**	-0.1168	0.1056	2.71E-01	-0.413	0.043	**2.80E-22**	0.00124	0.0612	9.84E-01
rs3135391	6	32518965	A/G	*HLA-DRA*	CODING	3	1.388	1.232	1.565	7.93E-08	-0.249	0.043	**5.50E-09**	0.1165	0.1058	2.74E-01	0.182	0.042	**1.70E-05**	-0.1168	0.1056	2.71E-01	-0.412	0.042	**3.00E-22**	0.00124	0.0612	9.84E-01
rs9277357	6	33157957	G/A	*HLA-DPB1*	INTRON	2	1.333	1.200	1.482	9.13E-08	-	-	-	-0.00936	0.1079	9.31E-01	-	-	-	-0.00839	0.1077	9.38E-01	-	-	-	-0.04974	0.06141	4.19E-01
rs3819717	6	32912277	G/A	*TAP2*	INTRON	3	1.246	1.149	1.351	1.02E-07	-0.049	0.033	1.37E-01	0.1361	0.1062	2.03E-01	-0.008	0.033	8.10E-01	-0.03462	0.1068	7.47E-01	-0.031	0.033	3.40E-01	-0.02439	0.06185	6.94E-01
rs9272353	6	32712434	C/G	*HLA-DRB1 | HLA-DQA1*	INTERGENIC	2	0.664	0.571	0.773	1.23E-07	-	-	-	-0.0525	0.1093	6.32E-01	-	-	-	-0.1391	0.1083	2.03E-01	-	-	-	0.2148	0.06073	**4.81E-04**
rs3129868	6	32512355	A/C	*BTNL2 | HLA-DRA*	INTERGENIC	3	1.381	1.225	1.557	1.30E-07	-0.254	0.043	**2.90E-09**	0.1145	0.1058	2.82E-01	0.182	0.042	**1.60E-05**	-0.1102	0.1057	3.00E-01	-0.418	0.043	**9.20E-23**	-0.0013	0.06122	9.83E-01
rs34102154	6	32680084	A/G	*HLA-DRB1 | HLA-DQA1*	INTERGENIC	1	1.517	1.295	1.778	2.55E-07	-	-	-	-	-	-	-	-	-	-	-	-	-	-	-	-	-	-
rs9275582	6	32788048	A/G	*HLA-DQB1 | HLA-DQA2*	INTERGENIC	3	0.759	0.683	0.845	3.80E-07	-0.003	0.039	9.40E-01	-0.06838	0.1079	5.28E-01	-0.026	0.038	4.90E-01	0.04438	0.1078	6.82E-01	0.016	0.039	6.70E-01	0.09025	0.0611	1.41E-01
rs9277472	6	33161701	G/A	*HLA-DPB1*	INTRON	3	1.240	1.141	1.347	3.85E-07	-	-	-	0.03763	0.1079	7.28E-01	-	-	-	-0.00474	0.1078	9.65E-01	-	-	-	-0.0496	0.06106	4.17E-01
rs2856717	6	32778286	A/G	*HLA-DQB1 | HLA-DQA2*	INTERGENIC	3	1.225	1.129	1.329	1.11E-06	-	-	-	0.1732	0.1083	1.14E-01	-	-	-	0.05581	0.1095	6.12E-01	-	-	-	-0.1722	0.06063	**4.86E-03**
rs9268199	6	32386613	G/A	*C6orf10*	INTRON	3	1.317	1.179	1.472	1.12E-06	-0.164	0.04	**4.30E-05**	0.1288	0.1062	2.29E-01	0.098	0.04	**1.31E-02**	-0.04422	0.1068	6.80E-01	-0.251	0.04	**2.70E-10**	-0.07696	0.06106	2.09E-01
rs9276826	6	32938546	G/A	*PSMB9 | HLA-DMB*	INTERGENIC	2	0.640	0.534	0.767	1.33E-06	-0.004	0.055	9.40E-01	0.04483	0.1075	6.78E-01	-0.004	0.055	9.30E-01	0.3671	0.09977	**4.07E-04**	-0.002	0.055	9.70E-01	-0.09994	0.06093	1.02E-01
rs2647012	6	32772436	A/G	*HLA-DQB1 | HLA-DQA2*	INTERGENIC	3	1.217	1.122	1.319	2.25E-06	-0.106	0.032	**9.20E-04**	0.1732	0.1083	1.14E-01	0.082	0.032	**9.40E-03**	0.05581	0.1095	6.12E-01	-0.183	0.032	**9.50E-09**	-0.1722	0.06063	**4.86E-03**
rs9273448	6	32735725	A/G	*HLA-DQB1*	UTR	3	1.230	1.127	1.342	3.47E-06	-	-	-	-	-	-	-	-	-	-	-	-	-	-	-	-	-	-
rs3129769	6	32705000	A/G	*HLA-DRB1 | HLA-DQA1*	INTERGENIC	1	0.640	0.530	0.773	3.80E-06	-	-	-	-0.07269	0.1081	5.03E-01	-	-	-	-0.1452	0.1071	1.79E-01	-	-	-	0.1749	0.06082	**4.37E-03**
rs2858324	6	32768353	A/G	*HLA-DQB1 | HLA-DQA2*	INTERGENIC	3	1.210	1.116	1.313	4.32E-06	-	-	-	0.1732	0.1083	1.14E-01	-	-	-	0.05581	0.1095	6.12E-01	-	-	-	-0.1722	0.06063	**4.86E-03**
rs1048087	6	32717264	G/A	*HLA-DQA1*	CODING	1	1.369	1.196	1.568	5.46E-06	-	-	-	0.05564	0.1073	6.06E-01	-	-	-	-0.01435	0.1073	8.94E-01	-	-	-	-0.1779	0.06048	**3.56E-03**
rs1044506	6	32280043	A/C	*NOTCH4*	CODING	3	1.337	1.180	1.515	5.49E-06	-0.189	0.044	**1.70E-05**	0.04188	0.1073	6.97E-01	0.218	0.043	**4.90E-07**	-0.108	0.1065	3.14E-01	-0.401	0.044	**4.30E-20**	0.003012	0.06136	9.61E-01
rs3131294	6	32288124	A/G	*NOTCH4*	INTRON	3	1.333	1.176	1.511	7.09E-06	-0.201	0.043	**3.10E-06**	0.04188	0.1073	6.97E-01	0.179	0.043	**2.70E-05**	-0.108	0.1065	3.14E-01	-0.373	0.043	**4.00E-18**	0.005794	0.06133	9.25E-01
rs3132940	6	32269374	A/C	*GPSM3*	INTRON	3	1.328	1.171	1.506	1.03E-05	-0.189	0.044	**1.40E-05**	0.04188	0.1073	6.97E-01	0.213	0.043	**8.10E-07**	-0.108	0.1065	3.14E-01	-0.394	0.043	**1.10E-19**	0.003012	0.06136	9.61E-01
rs3129867	6	32512198	G/C	*BTNL2 | HLA-DRA*	INTERGENIC	3	1.210	1.112	1.317	1.03E-05	-0.085	0.034	**1.30E-02**	0.05104	0.1065	6.33E-01	0.034	0.034	3.20E-01	-0.139	0.1054	1.91E-01	-0.107	0.034	**1.80E-03**	-0.05277	0.06118	3.89E-01
rs3132946	6	32298006	A/G	*NOTCH4*	INTRON	3	1.325	1.168	1.503	1.18E-05	-0.192	0.044	**1.30E-05**	0.05403	0.107	6.15E-01	0.213	0.044	**1.10E-06**	-0.1037	0.1064	3.32E-01	-0.398	0.044	**1.50E-19**	0.01161	0.0613	8.50E-01
rs9267992	6	32328375	G/A	*NOTCH4 | C6orf10*	INTERGENIC	3	1.293	1.152	1.451	1.33E-05	-0.225	0.043	**2.10E-07**	0.1623	0.1057	1.28E-01	0.204	0.043	**2.10E-06**	-0.07436	0.1066	4.87E-01	-0.418	0.043	**3.80E-22**	-0.00401	0.06123	9.48E-01
rs1800684	6	32259972	T/A	*AGER*	SYNONYMOUS	3	1.312	1.158	1.487	2.14E-05	-0.164	0.043	**1.20E-04**	0.04995	0.1074	6.43E-01	0.197	0.042	**2.90E-06**	-0.07091	0.1071	5.10E-01	-0.352	0.042	**1.00E-16**	0.01706	0.0616	7.82E-01
rs9275425	6	32778852	A/C	*HLA-DQB1 | HLA-DQA2*	INTERGENIC	3	0.815	0.741	0.897	2.65E-05	-0.02	0.046	6.70E-01	0.1065	0.1066	3.21E-01	-0.05	0.046	2.80E-01	0.03401	0.1087	7.55E-01	0.035	0.046	4.50E-01	0.0756	0.06148	2.20E-01
rs3117182	6	32174797	A/T	*TNXB*	INTRON	3	1.309	1.153	1.486	3.36E-05	-0.182	0.043	**2.70E-05**	0.07802	0.1089	4.76E-01	0.204	0.043	**2.10E-06**	-0.04302	0.1089	6.94E-01	-0.375	0.043	**4.10E-18**	0.03495	0.06314	5.80E-01
rs28362678	6	32470723	A/G	*BTNL2*	CODING	3	0.764	0.672	0.868	3.47E-05	-	-	-	-0.07941	0.1066	4.58E-01	-	-	-	0.04441	0.1059	6.76E-01	-	-	-	-0.1053	0.06125	8.67E-02
rs9267955	6	32321128	A/G	*NOTCH4 | C6orf10*	INTERGENIC	3	1.271	1.134	1.425	3.95E-05	-0.225	0.043	**2.10E-07**	0.1623	0.1057	1.28E-01	0.204	0.043	**2.10E-06**	-0.07436	0.1066	4.87E-01	-0.418	0.043	**3.80E-22**	-0.00401	0.06123	9.48E-01
rs3130342	6	32188124	A/C	*TNXB | CREBL1*	INTERGENIC	3	1.301	1.146	1.477	4.88E-05	-0.181	0.043	**2.90E-05**	0.07572	0.1067	4.80E-01	0.204	0.043	**1.90E-06**	-0.04308	0.1067	6.88E-01	-0.373	0.043	**4.40E-18**	0.03592	0.06163	5.61E-01

Supplementary Table S16. Enrichment analysis in intersected sets between LS and T-cells (CD4+, CD8+, and CD4/CD8 ratio) associated variants in healthy and diseased groups (A - F)

A. LS-associated variants in blood CD4 of healthy individuals

Enrichment by Pathway Maps

					overlap between LS and blood.cd4 SNPs			
#	Maps	Total	pValue	Min FDR	p-value	FDR	In Data	Network Objects from Active Data
1	Immune response_Antigen presentation by MHC class I	28	1.43E-07	5.15E-06	1.43E-07	**5.15E-06**	4	TAP2 (PSF2), TAP1 (PSF1), MHC class I, PSMB8(LMP7)
2	Immune response_Lectin induced complement pathway	50	9.00E-05	1.02E-03	9.00E-05	**1.02E-03**	3	C2b, C2a, C2
3	Immune response_Classical complement pathway	53	1.07E-04	1.02E-03	1.07E-04	**1.02E-03**	3	C2b, C2a, C2
4	Role of IL-23/ T17 pathogenic axis in psoriasis	54	1.13E-04	1.02E-03	1.13E-04	**1.02E-03**	3	HLA-Cw6, HLA-C, MHC class I
5	Role of B cells in SLE	57	4.59E-03	3.30E-02	4.59E-03	**3.30E-02**	2	TNF-beta, MHC class I

Enrichment by Process Networks

					overlap between LS and blood.cd4 SNPs			
#	Networks	Total	pValue	Min FDR	p-value	FDR	In Data	Network Objects from Active Data
1	Immune response_Antigen presentation	197	7.05E-09	2.04E-07	7.05E-09	**2.04E-07**	9	TAP2 (PSF2), HLA-C, HA2Z, TAP, PSMB9, HLA-Cw3, HLA-DRA1, TAP1 (PSF1), MHC class I
2	Immune response_Phagosome in antigen presentation	243	7.35E-07	1.07E-05	7.35E-07	**1.07E-05**	8	TAP2 (PSF2), HLA-C, TAP, PSMB9, HLA-Cw3, HLA-DRA1, TAP1 (PSF1), MHC class I
3	Inflammation_Complement system	75	1.92E-03	1.86E-02	1.92E-03	**1.86E-02**	3	C2b, C2a, C2

Enrichment by GO Processes

					overlap betwwen LS and blood.cd4 SNPs			
#	Processes	Total	pValue	Min FDR	p-value	FDR	In Data	Network Objects from Active Data
1	antigen processing and presentation of peptide antigen	292	6.62E-19	8.15E-16	6.62E-19	**8.15E-16**	15	MHC class II alpha chain, TAP2 (PSF2), HLA-Cw6, HLA-C, HLA-Cw7, HA2Z, TAP, PSMB9, BAT3, HLA-Cw3, HLA-DRA1, TAP1 (PSF1), MHC class I, PSMB8(LMP7), HLA-Cw4
2	antigen processing and presentation of endogenous peptide antigen via MHC class I via ER pathway	30	7.27E-19	8.15E-16	7.27E-19	**8.15E-16**	9	TAP2 (PSF2), HLA-Cw6, HLA-C, HLA-Cw7, TAP, HLA-Cw3, TAP1 (PSF1), MHC class I, HLA-Cw4
3	antigen processing and presentation of endogenous peptide antigen via MHC class I	34	2.65E-18	1.98E-15	2.65E-18	**1.98E-15**	9	TAP2 (PSF2), HLA-Cw6, HLA-C, HLA-Cw7, TAP, HLA-Cw3, TAP1 (PSF1), MHC class I, HLA-Cw4
4	antigen processing and presentation of exogenous peptide antigen	260	6.22E-18	2.81E-15	6.22E-18	**2.81E-15**	14	MHC class II alpha chain, TAP2 (PSF2), HLA-Cw6, HLA-C, HLA-Cw7, HA2Z, TAP, PSMB9, HLA-Cw3, HLA-DRA1, TAP1 (PSF1), MHC class I, PSMB8(LMP7), HLA-Cw4
5	antigen processing and presentation of endogenous peptide antigen	37	6.27E-18	2.81E-15	6.27E-18	**2.81E-15**	9	TAP2 (PSF2), HLA-Cw6, HLA-C, HLA-Cw7, TAP, HLA-Cw3, TAP1 (PSF1), MHC class I, HLA-Cw4
6	antigen processing and presentation of exogenous antigen	270	1.06E-17	3.95E-15	1.06E-17	**3.95E-15**	14	MHC class II alpha chain, TAP2 (PSF2), HLA-Cw6, HLA-C, HLA-Cw7, HA2Z, TAP, PSMB9, HLA-Cw3, HLA-DRA1, TAP1 (PSF1), MHC class I, PSMB8(LMP7), HLA-Cw4
7	antigen processing and presentation	360	1.53E-17	4.90E-15	1.53E-17	**4.90E-15**	15	MHC class II alpha chain, TAP2 (PSF2), HLA-Cw6, HLA-C, HLA-Cw7, HA2Z, TAP, PSMB9, BAT3, HLA-Cw3, HLA-DRA1, TAP1 (PSF1), MHC class I, PSMB8(LMP7), HLA-Cw4
8	antigen processing and presentation of endogenous antigen	41	1.76E-17	4.92E-15	1.76E-17	**4.92E-15**	9	TAP2 (PSF2), HLA-Cw6, HLA-C, HLA-Cw7, TAP, HLA-Cw3, TAP1 (PSF1), MHC class I, HLA-Cw4
9	antigen processing and presentation of exogenous peptide antigen via MHC class I, TAP-dependent	115	5.67E-17	1.41E-14	5.67E-17	**1.41E-14**	11	TAP2 (PSF2), HLA-Cw6, HLA-C, HLA-Cw7, TAP, PSMB9, HLA-Cw3, TAP1 (PSF1), MHC class I, PSMB8(LMP7), HLA-Cw4
10	antigen processing and presentation of peptide antigen via MHC class I	169	7.13E-17	1.60E-14	7.13E-17	**1.60E-14**	12	TAP2 (PSF2), HLA-Cw6, HLA-C, HLA-Cw7, TAP, PSMB9, BAT3, HLA-Cw3, TAP1 (PSF1), MHC class I, PSMB8(LMP7), HLA-Cw4

B. LS-associated variants in BAL CD4 T-cells of LS cases

Enrichment by Pathway Maps

					overlap between LS and bal.cd4 SNPs			
#	Maps	Total	pValue	Min FDR	p-value	FDR	In Data	Network Objects from Active Data
1	Rheumatoid arthritis (general schema)	50	8.21E-05	2.96E-03	8.21E-05	**2.96E-03**	2	MHC class II beta chain, MHC class II
2	SLE genetic marker-specific pathways in T cells	101	3.37E-04	6.07E-03	3.37E-04	**6.07E-03**	2	MHC class II beta chain, MHC class II
3	Immune response_Antigen presentation by MHC class II	12	3.81E-03	2.16E-02	3.81E-03	**2.16E-02**	1	MHC class II
4	LRRK2 and immune function in Parkinson's disease	22	6.97E-03	2.16E-02	6.97E-03	**2.16E-02**	1	MHC class II
5	Immune response_Role of HMGB1 in dendritic cell maturation and migration	27	8.55E-03	2.16E-02	8.55E-03	**2.16E-02**	1	MHC class II
6	G-protein signaling_N-RAS regulation pathway	33	1.04E-02	2.16E-02	1.04E-02	**2.16E-02**	1	MHC class II
7	Immune response_IL-22 signaling pathway	34	1.08E-02	2.16E-02	1.08E-02	**2.16E-02**	1	MHC class II
8	Immune response_Th17 cell differentiation	35	1.11E-02	2.16E-02	1.11E-02	**2.16E-02**	1	MHC class II
9	Immune response_Differentiation of natural regulatory T cells	35	1.11E-02	2.16E-02	1.11E-02	**2.16E-02**	1	MHC class II
10	Immune response_Generation of memory CD4+ T cells	37	1.17E-02	2.16E-02	1.17E-02	**2.16E-02**	1	MHC class II

Enrichment by Process Networks

					LS and bal.cd4 SNPs overlap			
#	Networks	Total	pValue	Min FDR	p-value	FDR	In Data	Network Objects from Active Data
1	Immune response_Antigen presentation	197	4.38E-03	3.94E-02	4.38E-03	**3.94E-02**	2	MHC class II beta chain, MHC class II

Enrichment by GO Processes

					overlap between LS and bal.cd4 SNPs			
#	Processes	Total	pValue	Min FDR	p-value	FDR	In Data	Network Objects from Active Data
1	T cell costimulation	105	4.19E-11	9.57E-09	4.19E-11	**9.57E-09**	5	MHC class II beta chain, MHC class II alpha chain, HLA-DQA2, MHC class II, HLA-DQB2
2	lymphocyte costimulation	106	4.40E-11	9.57E-09	4.40E-11	**9.57E-09**	5	MHC class II beta chain, MHC class II alpha chain, HLA-DQA2, MHC class II, HLA-DQB2
3	interferon-gamma-mediated signaling pathway	107	4.61E-11	9.57E-09	4.61E-11	**9.57E-09**	5	MHC class II beta chain, MHC class II alpha chain, HLA-DQA2, MHC class II, HLA-DQB2
4	antigen processing and presentation of exogenous peptide antigen via MHC class II	136	1.56E-10	1.46E-08	1.56E-10	**1.46E-08**	5	MHC class II beta chain, MHC class II alpha chain, HLA-DQA2, MHC class II, HLA-DQB2
5	positive regulation of cell-cell adhesion	385	1.67E-10	1.46E-08	1.67E-10	**1.46E-08**	6	MHC class II beta chain, Flotillin-1, MHC class II alpha chain, HLA-DQA2, MHC class II, HLA-DQB2
6	antigen processing and presentation of peptide antigen via MHC class II	138	1.68E-10	1.46E-08	1.68E-10	**1.46E-08**	5	MHC class II beta chain, MHC class II alpha chain, HLA-DQA2, MHC class II, HLA-DQB2
7	negative regulation of antigen processing and presentation of peptide antigen via MHC class II	5	1.84E-10	1.46E-08	1.84E-10	**1.46E-08**	3	MHC class II beta chain, MHC class II alpha chain, MHC class II
8	antigen processing and presentation of peptide or polysaccharide antigen via MHC class II	141	1.87E-10	1.46E-08	1.87E-10	**1.46E-08**	5	MHC class II beta chain, MHC class II alpha chain, HLA-DQA2, MHC class II, HLA-DQB2
9	negative regulation of antigen processing and presentation of peptide or polysaccharide antigen via MHC class II	6	3.69E-10	2.55E-08	3.69E-10	**2.55E-08**	3	MHC class II beta chain, MHC class II alpha chain, MHC class II
10	regulation of antigen processing and presentation of peptide antigen via MHC class II	7	6.45E-10	3.65E-08	6.45E-10	**3.65E-08**	3	MHC class II beta chain, MHC class II alpha chain, MHC class II

C. LS-associated variants in blood CD8 T-cells of healthy individuals

Enrichment by Pathway Maps

					overlap between LS and blood.cd8 SNPs			
#	Maps	Total	pValue	Min FDR	p-value	FDR	In Data	Network Objects from Active Data
1	NETosis in SLE	31	6.48E-06	1.08E-03	6.48E-06	**1.08E-03**	4	Histone H4, Histone H2, Histone H2A, Histone H1
2	Role of IL-23/ T17 pathogenic axis in psoriasis	54	6.09E-05	4.20E-03	6.09E-05	**4.20E-03**	4	HLA-C, MHC class I, HLA-Cw6, MHC class II
3	Transcription_Epigenetic regulation of gene expression	57	7.55E-05	4.20E-03	7.55E-05	**4.20E-03**	4	Histone H4, Histone H2A, Histone H2B, G9a
4	Immune response_Role of HMGB1 in dendritic cell maturation and migration	27	1.64E-04	6.86E-03	1.64E-04	**6.86E-03**	3	MHC class II, TNF-alpha, RAGE
5	SLE genetic marker-specific pathways in T cells	101	6.87E-04	1.94E-02	6.87E-04	**1.94E-02**	4	MICB, MHC class II beta chain, RFP, MHC class II
6	Immune response_Antigen presentation by MHC class II	12	1.01E-03	1.94E-02	1.01E-03	**1.94E-02**	2	HLA-DRA1, MHC class II
7	Immune response_Lectin induced complement pathway	50	1.03E-03	1.94E-02	1.03E-03	**1.94E-02**	3	C2b, C2a, C2
8	Rheumatoid arthritis (general schema)	50	1.03E-03	1.94E-02	1.03E-03	**1.94E-02**	3	MHC class II beta chain, MHC class II, TNF-alpha
9	Immune response_Classical complement pathway	53	1.22E-03	1.94E-02	1.22E-03	**1.94E-02**	3	C2b, C2a, C2
10	Immune response_Alternative complement pathway	53	1.22E-03	1.94E-02	1.22E-03	**1.94E-02**	3	Factor B, Factor Ba, Factor Bb

Enrichment by Process Networks

					overlap between LS and blood.cd8 SNPs			
#	Networks	Total	pValue	Min FDR	p-value	FDR	In Data	Network Objects from Active Data
1	Immune response_Antigen presentation	197	1.08E-11	8.43E-10	1.08E-11	**8.43E-10**	15	HLA-C, MICB, HLA-DRA1, HLA-B, TAP2 (PSF2), MHC class II beta chain, MHC class I, HLA-B7, MHC class II, HLA-F, TNF-alpha, HLA-DQA1, MICA, HLA-B27, HLA-Cw3
2	Inflammation_NK cell cytotoxicity	164	3.72E-07	1.45E-05	3.72E-07	**1.45E-05**	10	HLA-C, MICB, HLA-B, NKp30, MHC class I, Histone H1, Histone H2B, TNF-alpha, MICA, HLA-B27
3	Immune response_Phagosome in antigen presentation	243	8.53E-05	2.22E-03	8.53E-05	**2.22E-03**	9	HLA-C, HLA-DRA1, HLA-B, TAP2 (PSF2), MHC class I, MHC class II, HLA-DQA1, HLA-B27, HLA-Cw3
4	Inflammation_Complement system	75	2.58E-04	5.03E-03	2.58E-04	**5.03E-03**	5	C2b, C2a, C2, Factor B, Factor Bb
5	Transcription_Chromatin modification	127	3.99E-04	6.23E-03	3.99E-04	**6.23E-03**	6	Histone H4, Histone H2, Histone H2A, Histone H1, Histone H2B, G9a
6	Reproduction_Male sex differentiation	243	2.44E-03	3.17E-02	2.44E-03	**3.17E-02**	7	Histone H1.1, Histone H2, Histone H2A, Olfactory receptor, Histone H1, Casein kinase II, Oct-3/4

Enrichment by GO Processes

					overlap between LS and blood.cd8 SNPs			
#	Processes	Total	pValue	Min FDR	p-value	FDR	In Data	Network Objects from Active Data
1	interferon-gamma-mediated signaling pathway	107	1.09E-33	3.05E-30	1.09E-33	**3.05E-30**	25	RORET, MHC class II alpha chain, HLA-C, TRIM26, HLA-DRA1, HLA-Cw4, HLA-B, 1B35_HUMAN, MHC class II beta chain, HLA-DQA2, 1B57_HUMAN, MHC class I, HLA-B7, HLAB, TRIM31, 1B08_HUMAN, HLA-Cw6, HLA-Cw7, MHC class II, HLA-F, 1B73, HLA-DQA1, HLA-B27, HLA-DQB2, HLA-Cw3
2	cellular response to interferon-gamma	193	2.38E-28	2.37E-25	2.38E-28	**2.37E-25**	26	RORET, MHC class II alpha chain, HLA-C, TRIM26, HLA-DRA1, HLA-Cw4, HLA-B, 1B35_HUMAN, MHC class II beta chain, HLA-DQA2, 1B57_HUMAN, MHC class I, HLA-B7, IRT-1, HLAB, TRIM31, 1B08_HUMAN, HLA-Cw6, HLA-Cw7, MHC class II, HLA-F, 1B73, HLA-DQA1, HLA-B27, HLA-DQB2, HLA-Cw3
3	antigen processing and presentation of endogenous peptide antigen via MHC class I via ER pathway, TAP-independen	19	2.55E-28	2.37E-25	2.55E-28	**2.37E-25**	14	HLA-C, HLA-Cw4, HLA-B, 1B35_HUMAN, 1B57_HUMAN, MHC class I, HLA-B7, HLAB, 1B08_HUMAN, HLA-Cw6, HLA-Cw7, 1B73, HLA-B27, HLA-Cw3
4	antigen processing and presentation of exogenous peptide antigen via MHC class I, TAP-independent	25	3.61E-28	2.45E-25	3.61E-28	**2.45E-25**	15	HLA-C, HLA-Cw4, HLA-B, 1B35_HUMAN, 1B57_HUMAN, MHC class I, HLA-B7, HLAB, 1B08_HUMAN, HLA-Cw6, HLA-Cw7, HLA-F, 1B73, HLA-B27, HLA-Cw3
5	response to interferon-gamma	224	4.40E-28	2.45E-25	4.40E-28	**2.45E-25**	27	RORET, MHC class II alpha chain, HLA-C, TRIM26, HLA-DRA1, HLA-Cw4, HLA-B, 1B35_HUMAN, FAT10, MHC class II beta chain, HLA-DQA2, 1B57_HUMAN, MHC class I, HLA-B7, IRT-1, HLAB, TRIM31, 1B08_HUMAN, HLA-Cw6, HLA-Cw7, MHC class II, HLA-F, 1B73, HLA-DQA1, HLA-B27, HLA-DQB2, HLA-Cw3
6	antigen processing and presentation of endogenous peptide antigen via MHC class I via ER pathway	30	1.67E-26	7.77E-24	1.67E-26	**7.77E-24**	15	HLA-C, HLA-Cw4, HLA-B, TAP2 (PSF2), 1B35_HUMAN, 1B57_HUMAN, MHC class I, HLA-B7, HLAB, 1B08_HUMAN, HLA-Cw6, HLA-Cw7, 1B73, HLA-B27, HLA-Cw3
7	antigen processing and presentation of endogenous peptide antigen via MHC class I	34	1.96E-25	7.81E-23	1.96E-25	**7.81E-23**	15	HLA-C, HLA-Cw4, HLA-B, TAP2 (PSF2), 1B35_HUMAN, 1B57_HUMAN, MHC class I, HLA-B7, HLAB, 1B08_HUMAN, HLA-Cw6, HLA-Cw7, 1B73, HLA-B27, HLA-Cw3
8	antigen processing and presentation of endogenous peptide antigen	37	9.76E-25	3.40E-22	9.76E-25	**3.40E-22**	15	HLA-C, HLA-Cw4, HLA-B, TAP2 (PSF2), 1B35_HUMAN, 1B57_HUMAN, MHC class I, HLA-B7, HLAB, 1B08_HUMAN, HLA-Cw6, HLA-Cw7, 1B73, HLA-B27, HLA-Cw3
9	antigen processing and presentation of endogenous antigen	41	6.49E-24	2.01E-21	6.49E-24	**2.01E-21**	15	HLA-C, HLA-Cw4, HLA-B, TAP2 (PSF2), 1B35_HUMAN, 1B57_HUMAN, MHC class I, HLA-B7, HLAB, 1B08_HUMAN, HLA-Cw6, HLA-Cw7, 1B73, HLA-B27, HLA-Cw3
10	antigen processing and presentation	360	1.65E-22	4.60E-20	1.65E-22	**4.60E-20**	27	MHC class II alpha chain, HLA-C, MICB, BAT3, HLA-DRA1, HLA-Cw4, HLA-B, TAP2 (PSF2), 1B35_HUMAN, MHC class II beta chain, HLA-DQA2, 1B57_HUMAN, MHC class I, HLA-B7, HLAB, 1B08_HUMAN, HLA-Cw6, HLA-Cw7, HLADOB, MHC class II, HLA-F, 1B73, HLA-DQA1, MICA, HLA-B27, HLA-DQB2, HLA-Cw3

D. LS-associated variants in BAL CD8 T-cells of LS cases

Enrichment by Pathway Maps

					overlap between LS and bal.cd8 SNPs			
#	Maps	Total	pValue	Min FDR	p-value	FDR	In Data	Network Objects from Active Data
1	Role of IL-23/ T17 pathogenic axis in psoriasis	54	6.56E-08	2.76E-06	6.56E-08	**2.76E-06**	4	HLA-Cw6, HLA-C, MHC class I, MHC class II
2	Immune response_Antigen presentation by MHC class II	12	4.12E-05	8.66E-04	4.12E-05	**8.66E-04**	2	HLA-DRA1, MHC class II
3	Immune response_Differentiation and clonal expansion of CD8+ T cells	39	4.58E-04	5.14E-03	4.58E-04	**5.14E-03**	2	MHC class I, MHC class II
4	Rheumatoid arthritis (general schema)	50	7.53E-04	5.14E-03	7.53E-04	**5.14E-03**	2	MHC class II beta chain, MHC class II
5	Immune response_Antiviral actions of Interferons	52	8.15E-04	5.14E-03	8.15E-04	**5.14E-03**	2	MHC class I, MHC class II
6	Immune response_Inhibitory PD-1 signaling in T cells	53	8.46E-04	5.14E-03	8.46E-04	**5.14E-03**	2	MHC class I, MHC class II
7	Immune response_HSP60 and HSP70/ TLR signaling pathway	54	8.78E-04	5.14E-03	8.78E-04	**5.14E-03**	2	MHC class I, MHC class II
8	Role of B cells in SLE	57	9.78E-04	5.14E-03	9.78E-04	**5.14E-03**	2	MHC class I, MHC class II
9	SLE genetic marker-specific pathways in T cells	101	3.04E-03	1.42E-02	3.04E-03	**1.42E-02**	2	MHC class II beta chain, MHC class II

Enrichment by Process Networks

					overlap between LS and bal.cd8 SNPs			
#	Networks	Total	pValue	Min FDR	p-value	FDR	In Data	Network Objects from Active Data
1	Immune response_Antigen presentation	197	3.29E-09	5.59E-08	3.29E-09	**5.59E-08**	7	MICA, MHC class II beta chain, HLA-C, HLA-Cw3, HLA-DRA1, MHC class I, MHC class II
2	Immune response_Phagosome in antigen presentation	243	1.71E-05	1.45E-04	1.71E-05	**1.45E-04**	5	HLA-C, HLA-Cw3, HLA-DRA1, MHC class I, MHC class II
3	Inflammation_NK cell cytotoxicity	164	1.71E-03	8.63E-03	1.71E-03	**8.63E-03**	3	MICA, HLA-C, MHC class I
4	Immune response_TCR signaling	174	2.03E-03	8.63E-03	2.03E-03	**8.63E-03**	3	MICA, MHC class I, MHC class II
5	Cell adhesion_Amyloid proteins	195	2.81E-03	9.57E-03	2.81E-03	**9.57E-03**	3	Notch, NOTCH4, NOTCH4 (ICD4)
6	Inflammation_IL-4 signaling	115	1.29E-02	3.64E-02	1.29E-02	**3.64E-02**	2	HLA-DRA1, MHC class II

Enrichment by GO Processes

					overlap between LS and bal.cd8 SNPs			
#	Processes	Total	pValue	Min FDR	p-value	FDR	In Data	Network Objects from Active Data
1	interferon-gamma-mediated signaling pathway	107	3.47E-24	4.97E-21	3.47E-24	**4.97E-21**	12	MHC class II alpha chain, MHC class II beta chain, HLA-Cw6, HLA-DQA2, HLA-C, HLA-Cw7, HLA-DQB2, HLA-Cw3, HLA-DRA1, MHC class I, MHC class II, HLA-Cw4
2	cellular response to interferon-gamma	193	2.35E-23	1.68E-20	2.35E-23	**1.68E-20**	13	MHC class II alpha chain, MHC class II beta chain, HLA-Cw6, HLA-DQA2, HLA-C, HLA-Cw7, HLA-DQB2, HLA-Cw3, HLA-DRA1, IRT-1, MHC class I, MHC class II, HLA-Cw4
3	response to interferon-gamma	224	1.71E-22	8.17E-20	1.71E-22	**8.17E-20**	13	MHC class II alpha chain, MHC class II beta chain, HLA-Cw6, HLA-DQA2, HLA-C, HLA-Cw7, HLA-DQB2, HLA-Cw3, HLA-DRA1, IRT-1, MHC class I, MHC class II, HLA-Cw4
4	antigen processing and presentation	360	6.06E-22	2.17E-19	6.06E-22	**2.17E-19**	14	MHC class II alpha chain, MICA, MHC class II beta chain, HLA-Cw6, BAT3, HLA-DQA2, HLA-C, HLA-Cw7, HLA-DQB2, HLA-Cw3, HLA-DRA1, MHC class I, MHC class II, HLA-Cw4
5	antigen processing and presentation of peptide antigen	292	5.74E-21	1.64E-18	5.74E-21	**1.64E-18**	13	MHC class II alpha chain, MHC class II beta chain, HLA-Cw6, BAT3, HLA-DQA2, HLA-C, HLA-Cw7, HLA-DQB2, HLA-Cw3, HLA-DRA1, MHC class I, MHC class II, HLA-Cw4
6	antigen processing and presentation of exogenous peptide antigen	260	2.06E-19	4.92E-17	2.06E-19	**4.92E-17**	12	MHC class II alpha chain, MHC class II beta chain, HLA-Cw6, HLA-DQA2, HLA-C, HLA-Cw7, HLA-DQB2, HLA-Cw3, HLA-DRA1, MHC class I, MHC class II, HLA-Cw4
7	antigen processing and presentation of exogenous antigen	270	3.27E-19	6.68E-17	3.27E-19	**6.68E-17**	12	MHC class II alpha chain, MHC class II beta chain, HLA-Cw6, HLA-DQA2, HLA-C, HLA-Cw7, HLA-DQB2, HLA-Cw3, HLA-DRA1, MHC class I, MHC class II, HLA-Cw4
8	antigen processing and presentation of endogenous peptide antigen via MHC class I via ER pathway, TAP-independent	19	4.06E-15	7.27E-13	4.06E-15	**7.27E-13**	6	HLA-Cw6, HLA-C, HLA-Cw7, HLA-Cw3, MHC class I, HLA-Cw4
9	immune system process	3132	6.10E-15	9.70E-13	6.10E-15	**9.70E-13**	18	MHC class II alpha chain, MICA, Notch, MHC class II beta chain, HLA-Cw6, BAT3, HLA-DQA2, G18, NOTCH4, HLA-C, HLA-Cw7, HLA-DQB2, HLA-Cw3, HLA-DRA1, IRT-1, MHC class I, MHC class II, HLA-Cw4
10	immune response	2035	1.41E-14	2.02E-12	1.41E-14	**2.02E-12**	16	MHC class II alpha chain, MICA, Notch, MHC class II beta chain, HLA-Cw6, HLA-DQA2, G18, HLA-C, HLA-Cw7, HLA-DQB2, HLA-Cw3, HLA-DRA1, IRT-1, MHC class I, MHC class II, HLA-Cw4

E. LS-associated variants in blood CD4/CD8 ratio of healthy individuals

Enrichment by Pathway Maps

#	Maps	Total	pValue	Min FDR	overlap between LS and blood.cd4/cd8 SNPs p-value	FDR	In Data	Network Objects from Active Data
1	Immune response_Antigen presentation by MHC class I	28	5.80E-06	7.64E-04	5.80E-06	**7.64E-04**	4	PSMB8(LMP7), TAP2 (PSF2), MHC class I, TAP1 (PSF1)
2	NETosis in SLE	31	8.83E-06	7.64E-04	8.83E-06	**7.64E-04**	4	Histone H4, Histone H2, Histone H2A, Histone H1
3	Immune response_HSP60 and HSP70/ TLR signaling pathway	54	8.26E-05	3.53E-03	8.26E-05	**3.53E-03**	4	MHC class I, HSP70, MHC class II, TNF-alpha
4	Role of IL-23/ T17 pathogenic axis in psoriasis	54	8.26E-05	3.53E-03	8.26E-05	**3.53E-03**	4	HLA-C, MHC class I, HLA-Cw6, MHC class II
5	Transcription_Epigenetic regulation of gene expression	57	1.02E-04	3.53E-03	1.02E-04	**3.53E-03**	4	Histone H4, Histone H2A, Histone H2B, G9a
6	Immune response_Role of HMGB1 in dendritic cell maturation and migration	27	2.06E-04	5.95E-03	2.06E-04	**5.95E-03**	3	MHC class II, TNF-alpha, RAGE
7	SLE genetic marker-specific pathways in antigen-presenting cells (APC)	84	4.59E-04	1.13E-02	4.59E-04	**1.13E-02**	4	MICB, RFP, ATF-6 beta, TNF-alpha
8	SLE genetic marker-specific pathways in T cells	101	9.20E-04	1.99E-02	9.20E-04	**1.99E-02**	4	MICB, MHC class II beta chain, RFP, MHC class II
9	Immune response_Antigen presentation by MHC class II	12	1.18E-03	2.03E-02	1.18E-03	**2.03E-02**	2	HLA-DRA1, MHC class II
10	Immune response_Lectin induced complement pathway	50	1.29E-03	2.03E-02	1.29E-03	**2.03E-02**	3	C2b, C2a, C2

Enrichment by Process Networks

#	Networks	Total	pValue	Min FDR	overlap between LS and blood.cd4/cd8 SNPs p-value	FDR	In Data	Network Objects from Active Data
1	Immune response_Antigen presentation	197	2.03E-18	1.71E-16	2.03E-18	**1.71E-16**	22	HLA-C, MICB, HLA-DRA1, HLA-B, TAP2 (PSF2), HA2Z, MHC class II beta chain, HLA-DPB1, MHC class I, HLA-B7, PSMB9, HLADPA1, HSP70, TAP1 (PSF1), MHC class II, HLA-F, TAP, TNF-alpha, HLA-DQA1, MICA, HLA-B27, HLA-Cw3
2	Immune response_Phagosome in antigen presentation	243	5.48E-09	2.30E-07	5.48E-09	**2.30E-07**	15	HLA-C, HLA-DRA1, HLA-B, TAP2 (PSF2), HLA-DPB1, MHC class I, PSMB9, HLADPA1, HSP70, TAP1 (PSF1), MHC class II, TAP, HLA-DQA1, HLA-B27, HLA-Cw3
3	Inflammation_NK cell cytotoxicity	164	2.86E-06	8.02E-05	2.86E-06	**8.02E-05**	10	HLA-C, MICB, HLA-B, NKp30, MHC class I, Histone H1, Histone H2B, TNF-alpha, MICA, HLA-B27
4	Reproduction_Male sex differentiation	243	4.50E-04	9.45E-03	4.50E-04	**9.45E-03**	9	Histone H1.1, Histone H2, Histone H2A, HSPA1L, Olfactory receptor, Histone H1, HSP70, Casein kinase II, Oct-3/4
5	Inflammation_Complement system	75	6.96E-04	1.17E-02	6.96E-04	**1.17E-02**	5	C2b, C2a, C2, Factor B, Factor Bb
6	Transcription_Chromatin modification	127	1.25E-03	1.62E-02	1.25E-03	**1.62E-02**	6	Histone H4, Histone H2, Histone H2A, Histone H1, Histone H2B, G9a
7	Reproduction_Spermatogenesis, motility and copulation	228	1.35E-03	1.62E-02	1.35E-03	**1.62E-02**	8	Histone H1.1, Histone H2, Histone H2A, HSPA1L, Histone H1, HSP70, Casein kinase II, Oct-3/4
8	Inflammation_IL-4 signaling	115	4.62E-03	4.85E-02	4.62E-03	**4.85E-02**	5	HLA-DRA1, HLA-DPB1, HLADPA1, MHC class II, HLA-DQA1
9	Inflammation_Interferon signaling	110	2.04E-02	1.91E-01	2.04E-02	1.91E-01	4	MICB, TAP2 (PSF2), TAP1 (PSF1), MICA
10	Immune response_TCR signaling	174	2.46E-02	2.07E-01	2.46E-02	2.07E-01	5	MICB, MHC class I, MHC class II, TNF-alpha, MICA

Enrichment by GO Processes

#	Processes	Total	pValue	Min FDR	overlap between LS and blood.cd4/cd8 SNPs p-value	FDR	In Data	Network Objects from Active Data
1	interferon-gamma-mediated signaling pathway	107	2.03E-36	6.67E-33	2.03E-36	**6.67E-33**	27	RORET, MHC class II alpha chain, HLA-C, TRIM26, HLA-DRA1, HLA-Cw4, HLA-B, 1B35_HUMAN, MHC class II beta chain, HLA-DQA2, HLA-DPB1, 1B57_HUMAN, MHC class I, HLA-B7, HLAB, HLADPA1, TRIM31, 1B08_HUMAN, HLA-Cw6, HLA-Cw7, MHC class II, HLA-F, 1B73, HLA-DQA1, HLA-B27, HLA-Cw3, HLA-DQB2
2	antigen processing and presentation of exogenous peptide antigen	260	5.38E-31	8.38E-28	5.38E-31	**8.38E-28**	31	MHC class II alpha chain, HLA-C, HLA-DRA1, PSMB8(LMP7), HLA-Cw4, HLA-B, TAP2 (PSF2), HA2Z, 1B35_HUMAN, MHC class II beta chain, HLA-DQA2, HLA-DPB1, 1B57_HUMAN, MHC class I, HLA-B7, HLAB, PSMB9, HLADPA1, TAP1 (PSF1), 1B08_HUMAN, HLA-Cw6, HLA-Cw7, HLADOB, MHC class II, HLA-F, TAP, 1B73, HLA-DQA1, HLA-B27, HLA-Cw3, HLA-DQB2
3	antigen processing and presentation of peptide antigen	292	8.38E-31	8.38E-28	8.38E-31	**8.38E-28**	32	MHC class II alpha chain, HLA-C, BAT3, HLA-DRA1, PSMB8(LMP7), HLA-Cw4, HLA-B, TAP2 (PSF2), HA2Z, 1B35_HUMAN, MHC class II beta chain, HLA-DQA2, HLA-DPB1, 1B57_HUMAN, MHC class I, HLA-B7, HLAB, PSMB9, HLADPA1, TAP1 (PSF1), 1B08_HUMAN, HLA-Cw6, HLA-Cw7, HLADOB, MHC class II, HLA-F, TAP, 1B73, HLA-DQA1, HLA-B27, HLA-Cw3, HLA-DQB2
4	antigen processing and presentation of endogenous peptide antigen via MHC class I via ER pathway	30	1.29E-30	8.38E-28	1.29E-30	**8.38E-28**	17	HLA-C, HLA-Cw4, HLA-B, TAP2 (PSF2), 1B35_HUMAN, 1B57_HUMAN, MHC class I, HLA-B7, HLAB, TAP1 (PSF1), 1B08_HUMAN, HLA-Cw6, HLA-Cw7, TAP, 1B73, HLA-B27, HLA-Cw3
5	antigen processing and presentation	360	1.58E-30	8.38E-28	1.58E-30	**8.38E-28**	34	MHC class II alpha chain, HLA-C, MICB, BAT3, HLA-DRA1, PSMB8(LMP7), HLA-Cw4, HLA-B, TAP2 (PSF2), HA2Z, 1B35_HUMAN, MHC class II beta chain, HLA-DQA2, HLA-DPB1, 1B57_HUMAN, MHC class I, HLA-B7, HLAB, PSMB9, HLADPA1, TAP1 (PSF1), 1B08_HUMAN, HLA-Cw6, HLA-Cw7, HLADOB, MHC class II, HLA-F, TAP, 1B73, HLA-DQA1, MICA, HLA-B27, HLA-Cw3, HLA-DQB2
6	antigen processing and presentation of exogenous antigen	270	1.78E-30	8.38E-28	1.78E-30	**8.38E-28**	31	MHC class II alpha chain, HLA-C, HLA-DRA1, PSMB8(LMP7), HLA-Cw4, HLA-B, TAP2 (PSF2), HA2Z, 1B35_HUMAN, MHC class II beta chain, HLA-DQA2, HLA-DPB1, 1B57_HUMAN, MHC class I, HLA-B7, HLAB, PSMB9, HLADPA1, TAP1 (PSF1), 1B08_HUMAN, HLA-Cw6, HLA-Cw7, HLADOB, MHC class II, HLA-F, TAP, 1B73, HLA-DQA1, HLA-B27, HLA-Cw3, HLA-DQB2
7	cellular response to interferon-gamma	193	1.78E-30	8.38E-28	1.78E-30	**8.38E-28**	28	RORET, MHC class II alpha chain, HLA-C, TRIM26, HLA-DRA1, HLA-Cw4, HLA-B, 1B35_HUMAN, MHC class II beta chain, HLA-DQA2, HLA-DPB1, 1B57_HUMAN, MHC class I, HLA-B7, IRT-1, HLAB, HLADPA1, TRIM31, 1B08_HUMAN, HLA-Cw6, HLA-Cw7, MHC class II, HLA-F, 1B73, HLA-DQA1, HLA-B27, HLA-Cw3, HLA-DQB2
8	response to interferon-gamma	224	4.56E-30	1.88E-27	4.56E-30	**1.88E-27**	29	RORET, MHC class II alpha chain, HLA-C, TRIM26, HLA-DRA1, HLA-Cw4, HLA-B, FAT10, 1B35_HUMAN, MHC class II beta chain, HLA-DQA2, HLA-DPB1, 1B57_HUMAN, MHC class I, HLA-B7, IRT-1, HLAB, HLADPA1, TRIM31, 1B08_HUMAN, HLA-Cw6, HLA-Cw7, MHC class II, HLA-F, 1B73, HLA-DQA1, HLA-B27, HLA-Cw3, HLA-DQB2
9	antigen processing and presentation of endogenous peptide antigen via MHC class I	34	2.46E-29	8.98E-27	2.46E-29	**8.98E-27**	17	HLA-C, HLA-Cw4, HLA-B, TAP2 (PSF2), 1B35_HUMAN, 1B57_HUMAN, MHC class I, HLA-B7, HLAB, TAP1 (PSF1), 1B08_HUMAN, HLA-Cw6, HLA-Cw7, TAP, 1B73, HLA-B27, HLA-Cw3
10	antigen processing and presentation of endogenous peptide antigen	37	1.65E-28	5.43E-26	1.65E-28	**5.43E-26**	17	HLA-C, HLA-Cw4, HLA-B, TAP2 (PSF2), 1B35_HUMAN, 1B57_HUMAN, MHC class I, HLA-B7, HLAB, TAP1 (PSF1), 1B08_HUMAN, HLA-Cw6, HLA-Cw7, TAP, 1B73, HLA-B27, HLA-Cw3

F. LS-associated variants in BAL CD4/CD8 ratio of LS cases

Enrichment by Pathway Maps

					overlap between LS and bal.cd4/cd8 SNPs			
#	Maps	Total	pValue	Min FDR	p-value	FDR	In Data	Network Objects from Active Data
1	Immune response_T cell subsets: secreted signals	25	2.65E-03	6.88E-03	2.65E-03	**6.88E-03**	1	TNF-beta
2	HCV-dependent regulation of membrane receptors signaling in HCC	27	2.86E-03	6.88E-03	2.86E-03	**6.88E-03**	1	TNF-beta
3	Apoptosis and survival_Lymphotoxin-beta receptor signaling	42	4.45E-03	6.88E-03	4.45E-03	**6.88E-03**	1	TNF-beta
4	Role of B cells in SLE	57	6.03E-03	6.88E-03	6.03E-03	**6.88E-03**	1	TNF-beta
5	Immune response_CD40 signaling	65	6.88E-03	6.88E-03	6.88E-03	**6.88E-03**	1	TNF-beta

Enrichment by Process Networks

					overlap between LS and bal.cd4/cd8 SNPs			
#	Networks	Total	pValue	Min FDR	p-value	FDR	In Data	Network Objects from Active Data
1	Apoptosis_Death Domain receptors & caspases in apoptosis	123	1.72E-02	3.01E-02	1.72E-02	**3.01E-02**	1	TNF-beta
2	Immune response_T helper cell differentiation	140	1.96E-02	3.01E-02	1.96E-02	**3.01E-02**	1	TNF-beta
3	Apoptosis_Apoptosis stimulation by external signals	144	2.02E-02	3.01E-02	2.02E-02	**3.01E-02**	1	TNF-beta
4	Inflammation_Jak-STAT Pathway	186	2.60E-02	3.01E-02	2.60E-02	**3.01E-02**	1	TNF-beta
5	Proliferation_Lymphocyte proliferation	209	2.93E-02	3.01E-02	2.93E-02	**3.01E-02**	1	TNF-beta
6	Inflammation_Neutrophil activation	215	3.01E-02	3.01E-02	3.01E-02	**3.01E-02**	1	TNF-beta

Enrichment by GO Processes

					overlap between LS and bal.cd4/cd8 SNPs			
#	Processes	Total	pValue	Min FDR	p-value	FDR	In Data	Network Objects from Active Data
1	positive regulation of chronic inflammatory response to antigenic stimulus	2	1.78E-04	2.68E-02	1.78E-04	**2.68E-02**	1	TNF-beta
2	regulation of chronic inflammatory response to antigenic stimulus	3	2.67E-04	2.68E-02	2.67E-04	**2.68E-02**	1	TNF-beta
3	positive regulation of humoral immune response mediated by circulating immunoglobulin	8	7.11E-04	3.44E-02	7.11E-04	**3.44E-02**	1	TNF-beta
4	positive regulation of chronic inflammatory response	8	7.11E-04	3.44E-02	7.11E-04	**3.44E-02**	1	TNF-beta
5	transformed cell apoptotic process	11	9.78E-04	3.44E-02	9.78E-04	**3.44E-02**	1	TNF-beta
6	regulation of chronic inflammatory response	16	1.42E-03	3.44E-02	1.42E-03	**3.44E-02**	1	TNF-beta
7	regulation of humoral immune response mediated by circulating immunoglobulin	19	1.69E-03	3.44E-02	1.69E-03	**3.44E-02**	1	TNF-beta
8	positive regulation of glial cell proliferation	20	1.78E-03	3.44E-02	1.78E-03	**3.44E-02**	1	TNF-beta
9	negative regulation of growth of symbiont involved in interaction with host	23	2.04E-03	3.44E-02	2.04E-03	**3.44E-02**	1	TNF-beta
10	negative regulation of growth of symbiont in host	23	2.04E-03	3.44E-02	2.04E-03	**3.44E-02**	1	TNF-beta

Supplementary Table S17. Enrichment analysis conducted in intersected sets between genetic variants of non-LS and of T-cells (CD4+, CD8+, CD4/CD8 ratio) in healthy and diseased groups (A - F)

A. Non-LS-associated variants in blood CD4 T-cells of healthy indivials

Enrichment by Pathway Maps

#	Maps	Total	pValue	Min FDR	overlap between non-LS and blood.cd4 SNPs p-value	FDR	In Data	Network Objects from Active Data
1	Immune response_Antigen presentation by MHC class II	12	3.81E-03	2.15E-02	3.81E-03	**2.15E-02**	1	HLA-DRA1
2	Development_NOTCH-induced EMT	19	6.02E-03	2.15E-02	6.02E-03	**2.15E-02**	1	NOTCH4
3	Immune response_Role of HMGB1 in dendritic cell maturation and migration	27	8.55E-03	2.15E-02	8.55E-03	**2.15E-02**	1	RAGE
4	Immune response_HMGB1/TLR signaling pathway	36	1.14E-02	2.15E-02	1.14E-02	**2.15E-02**	1	RAGE
5	Breast cancer (general schema)	41	1.30E-02	2.15E-02	1.30E-02	**2.15E-02**	1	NOTCH4
6	Immune response_HMGB1/RAGE signaling pathway	53	1.67E-02	2.15E-02	1.67E-02	**2.15E-02**	1	RAGE
7	TLRs-mediated IFN-alpha production by plasmacytoid dendritic cells in SLE	53	1.67E-02	2.15E-02	1.67E-02	**2.15E-02**	1	RAGE
8	Development_Regulation of epithelial-to-mesenchymal transition (EMT)	64	2.02E-02	2.27E-02	2.02E-02	**2.27E-02**	1	NOTCH4
9	Cell adhesion_Integrin inside-out signaling in neutrophils	77	2.43E-02	2.43E-02	2.43E-02	**2.43E-02**	1	RAGE

Enrichment by Process Networks

#	Networks	Total	pValue	Min FDR	overlap between non-LS and blood.cd4 SNPs p-value	FDR	In Data	Network Objects from Active Data
1	Cell adhesion_Amyloid proteins	195	3.77E-04	4.53E-03	3.77E-04	**4.53E-03**	3	NOTCH4, Notch, NOTCH4 (ICD4)
2	Signal Transduction_TGF-beta, GDF and Activin signaling	154	6.55E-03	3.59E-02	6.55E-03	**3.59E-02**	2	NOTCH4, NOTCH4 (ICD4)
3	Development_Neurogenesis in general	192	1.00E-02	3.59E-02	1.00E-02	**3.59E-02**	2	NOTCH4, Notch
4	Development_Blood vessel morphogenesis	228	1.40E-02	3.59E-02	1.40E-02	**3.59E-02**	2	NOTCH4, Notch
5	Signal transduction_NOTCH signaling	236	1.49E-02	3.59E-02	1.49E-02	**3.59E-02**	2	NOTCH4, NOTCH4 (ICD4)

Enrichment by GO Processes

#	Processes	Total	pValue	Min FDR	overlap between non-LS and blood.cd4 SNPs p-value	FDR	In Data	Network Objects from Active Data
1	regulation of cell adhesion	953	1.48E-07	1.87E-04	1.48E-07	**1.87E-04**	6	MHC class II alpha chain, BTNL2, HLA-DRA1, RAGE, Notch, Tenascin-X
2	positive regulation of cell adhesion	576	5.68E-07	3.59E-04	5.68E-07	**3.59E-04**	5	MHC class II alpha chain, BTNL2, HLA-DRA1, RAGE, Tenascin-X
3	positive regulation of immune system process	1334	1.09E-06	3.98E-04	1.09E-06	**3.98E-04**	6	MHC class II alpha chain, BTNL2, HLA-DRA1, RAGE, Notch, G18
4	positive regulation of T cell activation	324	2.82E-06	3.98E-04	2.82E-06	**3.98E-04**	4	MHC class II alpha chain, BTNL2, HLA-DRA1, RAGE
5	positive regulation of homotypic cell-cell adhesion	330	3.04E-06	3.98E-04	3.04E-06	**3.98E-04**	4	MHC class II alpha chain, BTNL2, HLA-DRA1, RAGE
6	protein-carbohydrate complex assembly	8	3.09E-06	3.98E-04	3.09E-06	**3.98E-04**	2	MHC class II alpha chain, HLA-DRA1
7	protein-carbohydrate complex subunit organization	8	3.09E-06	3.98E-04	3.09E-06	**3.98E-04**	2	MHC class II alpha chain, HLA-DRA1
8	antigen processing and presentation of polysaccharide antigen via MHC class II	8	3.09E-06	3.98E-04	3.09E-06	**3.98E-04**	2	MHC class II alpha chain, HLA-DRA1
9	polysaccharide assembly with MHC class II protein complex	8	3.09E-06	3.98E-04	3.09E-06	**3.98E-04**	2	MHC class II alpha chain, HLA-DRA1
10	positive regulation of leukocyte cell-cell adhesion	333	3.15E-06	3.98E-04	3.15E-06	**3.98E-04**	4	MHC class II alpha chain, BTNL2, HLA-DRA1, RAGE

B. Non-LS-associated variants in BAL CD4 T-cells of non-LS cases

Enrichment by Pathway Maps

#	Maps	Total	pValue	Min FDR	overlap between non-LS and bal.cd4 SNPs			
					p-value	FDR	In Data	Network Objects from Active Data

None

Enrichment by Process Networks

#	Networks	Total	pValue	Min FDR	overlap between non-LS and bal.cd4 SNPs			
					p-value	FDR	In Data	Network Objects from Active Data

None

Enrichment by GO Processes

#	Processes	Total	pValue	Min FDR	overlap between non-LS and bal.cd4 SNPs			
					p-value	FDR	In Data	Network Objects from Active Data

None

C. Non-LS-associated variants in blood CD8 T-cells of healthy indviduals

Enrichment by Pathway Maps

#	Maps	Total	pValue	Min FDR	overlap between non-LS and blood.cd8 SNPs p-value	FDR	In Data	Network Objects from Active Data
1	Immune response_Antigen presentation by MHC class II	12	3.81E-03	2.15E-02	3.81E-03	**2.15E-02**	1	HLA-DRA1
2	Development_NOTCH-induced EMT	19	6.02E-03	2.15E-02	6.02E-03	**2.15E-02**	1	NOTCH4
3	Immune response_Role of HMGB1 in dendritic cell maturation and migration	27	8.55E-03	2.15E-02	8.55E-03	**2.15E-02**	1	RAGE
4	Immune response_HMGB1/TLR signaling pathway	36	1.14E-02	2.15E-02	1.14E-02	**2.15E-02**	1	RAGE
5	Breast cancer (general schema)	41	1.30E-02	2.15E-02	1.30E-02	**2.15E-02**	1	NOTCH4
6	Immune response_HMGB1/RAGE signaling pathway	53	1.67E-02	2.15E-02	1.67E-02	**2.15E-02**	1	RAGE
7	TLRs-mediated IFN-alpha production by plasmacytoid dendritic cells in SLE	53	1.67E-02	2.15E-02	1.67E-02	**2.15E-02**	1	RAGE
8	Development_Regulation of epithelial-to-mesenchymal transition (EMT)	64	2.02E-02	2.27E-02	2.02E-02	**2.27E-02**	1	NOTCH4
9	Cell adhesion_Integrin inside-out signaling in neutrophils	77	2.43E-02	2.43E-02	2.43E-02	**2.43E-02**	1	RAGE

Enrichment by Process Networks

#	Networks	Total	pValue	Min FDR	overlap between non-LS and blood.cd8 SNPs p-value	FDR	In Data	Network Objects from Active Data
1	Cell adhesion_Amyloid proteins	195	3.77E-04	4.53E-03	3.77E-04	**4.53E-03**	3	NOTCH4, Notch, NOTCH4 (ICD4)
2	Signal Transduction_TGF-beta, GDF and Activin signaling	154	6.55E-03	3.59E-02	6.55E-03	**3.59E-02**	2	NOTCH4, NOTCH4 (ICD4)
3	Development_Neurogenesis in general	192	1.00E-02	3.59E-02	1.00E-02	**3.59E-02**	2	NOTCH4, Notch
4	Development_Blood vessel morphogenesis	228	1.40E-02	3.59E-02	1.40E-02	**3.59E-02**	2	NOTCH4, Notch
5	Signal transduction_NOTCH signaling	236	1.49E-02	3.59E-02	1.49E-02	**3.59E-02**	2	NOTCH4, NOTCH4 (ICD4)

Enrichment by GO Processes

#	Processes	Total	pValue	Min FDR	overlap between non-LS and blood.cd8 SNPs p-value	FDR	In Data	Network Objects from Active Data
1	regulation of cell adhesion	953	1.48E-07	1.87E-04	1.48E-07	**1.87E-04**	6	MHC class II alpha chain, BTNL2, RAGE, HLA-DRA1, Notch, Tenascin-X
2	positive regulation of cell adhesion	576	5.68E-07	3.59E-04	5.68E-07	**3.59E-04**	5	MHC class II alpha chain, BTNL2, RAGE, HLA-DRA1, Tenascin-X
3	positive regulation of immune system process	1334	1.09E-06	3.98E-04	1.09E-06	**3.98E-04**	6	MHC class II alpha chain, BTNL2, RAGE, HLA-DRA1, Notch, G18
4	positive regulation of T cell activation	324	2.82E-06	3.98E-04	2.82E-06	**3.98E-04**	4	MHC class II alpha chain, BTNL2, RAGE, HLA-DRA1
5	positive regulation of homotypic cell-cell adhesion	330	3.04E-06	3.98E-04	3.04E-06	**3.98E-04**	4	MHC class II alpha chain, BTNL2, RAGE, HLA-DRA1
6	protein-carbohydrate complex assembly	8	3.09E-06	3.98E-04	3.09E-06	**3.98E-04**	2	MHC class II alpha chain, HLA-DRA1
7	protein-carbohydrate complex subunit organization	8	3.09E-06	3.98E-04	3.09E-06	**3.98E-04**	2	MHC class II alpha chain, HLA-DRA1
8	antigen processing and presentation of polysaccharide antigen via MHC class II	8	3.09E-06	3.98E-04	3.09E-06	**3.98E-04**	2	MHC class II alpha chain, HLA-DRA1
9	polysaccharide assembly with MHC class II protein complex	8	3.09E-06	3.98E-04	3.09E-06	**3.98E-04**	2	MHC class II alpha chain, HLA-DRA1
10	positive regulation of leukocyte cell-cell adhesion	333	3.15E-06	3.98E-04	3.15E-06	**3.98E-04**	4	MHC class II alpha chain, BTNL2, RAGE, HLA-DRA1

D. Non-LS-associated variants in BAL CD8 T-cells of non-LS cases

Enrichment by Pathway Maps

					overlap between non-LS and bal.cd8 SNPs			
#	Maps	Total	pValue	Min FDR	p-value	FDR	In Data	Network Objects from Active Data
1	Immune response_Antigen presentation by MHC class II	12	1.27E-03	1.27E-03	1.27E-03	**1.27E-03**	1	HLA-DRA1

Enrichment by Process Networks

					overlap between non-LS and bal.cd8 SNPs			
#	Networks	Total	pValue	Min FDR	p-value	FDR	In Data	Network Objects from Active Data
1	Inflammation_IL-4 signaling	115	1.61E-02	3.40E-02	1.61E-02	**3.40E-02**	1	HLA-DRA1
2	Immune response_Antigen presentation	197	2.76E-02	3.40E-02	2.76E-02	**3.40E-02**	1	HLA-DRA1
3	Immune response_Phagosome in antigen presentation	243	3.40E-02	3.40E-02	3.40E-02	**3.40E-02**	1	HLA-DRA1

Enrichment by GO Processes

					overlap between non-LS and bal.cd8 SNPs			
#	Processes	Total	pValue	Min FDR	p-value	FDR	In Data	Network Objects from Active Data
1	protein-carbohydrate complex subunit organization	8	1.11E-07	6.36E-06	1.11E-07	**6.36E-06**	2	MHC class II alpha chain, HLA-DRA1
2	protein-carbohydrate complex assembly	8	1.11E-07	6.36E-06	1.11E-07	**6.36E-06**	2	MHC class II alpha chain, HLA-DRA1
3	polysaccharide assembly with MHC class II protein complex	8	1.11E-07	6.36E-06	1.11E-07	**6.36E-06**	2	MHC class II alpha chain, HLA-DRA1
4	antigen processing and presentation of polysaccharide antigen via MHC class II	8	1.11E-07	6.36E-06	1.11E-07	**6.36E-06**	2	MHC class II alpha chain, HLA-DRA1
5	peptide antigen assembly with MHC class II protein complex	11	2.17E-07	8.33E-06	2.17E-07	**8.33E-06**	2	MHC class II alpha chain, HLA-DRA1
6	MHC class II protein complex assembly	11	2.17E-07	8.33E-06	2.17E-07	**8.33E-06**	2	MHC class II alpha chain, HLA-DRA1
7	peptide antigen assembly with MHC protein complex	12	2.61E-07	8.57E-06	2.61E-07	**8.57E-06**	2	MHC class II alpha chain, HLA-DRA1
8	MHC protein complex assembly	13	3.08E-07	8.86E-06	3.08E-07	**8.86E-06**	2	MHC class II alpha chain, HLA-DRA1
9	T cell costimulation	105	2.16E-05	4.69E-04	2.16E-05	**4.69E-04**	2	MHC class II alpha chain, HLA-DRA1
10	lymphocyte costimulation	106	2.20E-05	4.69E-04	2.20E-05	**4.69E-04**	2	MHC class II alpha chain, HLA-DRA1

E. Non-LS-associated variants in blood CD4/CD8 ratio of healthy individuals

Enrichment by Pathway Maps

					overlap between non-LS and blood.cd4/cd8 SNPs			
#	Maps	Total	pValue	Min FDR	p-value	FDR	In Data	Network Objects from Active Data
1	Immune response_Antigen presentation by MHC class II	12	3.81E-03	2.15E-02	3.81E-03	**2.15E-02**	1	HLA-DRA1
2	Development_NOTCH-induced EMT	19	6.02E-03	2.15E-02	6.02E-03	**2.15E-02**	1	NOTCH4
3	Immune response_Role of HMGB1 in dendritic cell maturation and migration	27	8.55E-03	2.15E-02	8.55E-03	**2.15E-02**	1	RAGE
4	Immune response_HMGB1/TLR signaling pathway	36	1.14E-02	2.15E-02	1.14E-02	**2.15E-02**	1	RAGE
5	Breast cancer (general schema)	41	1.30E-02	2.15E-02	1.30E-02	**2.15E-02**	1	NOTCH4
6	Immune response_HMGB1/RAGE signaling pathway	53	1.67E-02	2.15E-02	1.67E-02	**2.15E-02**	1	RAGE
7	TLRs-mediated IFN-alpha production by plasmacytoid dendritic cells in SLE	53	1.67E-02	2.15E-02	1.67E-02	**2.15E-02**	1	RAGE
8	Development_Regulation of epithelial-to-mesenchymal transition (EMT)	64	2.02E-02	2.27E-02	2.02E-02	**2.27E-02**	1	NOTCH4
9	Cell adhesion_Integrin inside-out signaling in neutrophils	77	2.43E-02	2.43E-02	2.43E-02	**2.43E-02**	1	RAGE

Enrichment by Process Networks

					overlap between non-LS and blood.cd4/cd8 SNPs			
#	Networks	Total	pValue	Min FDR	p-value	FDR	In Data	Network Objects from Active Data
1	Cell adhesion_Amyloid proteins	195	3.77E-04	4.53E-03	3.77E-04	**4.53E-03**	3	NOTCH4, Notch, NOTCH4 (ICD4)
2	Signal Transduction_TGF-beta, GDF and Activin signaling	154	6.55E-03	3.59E-02	6.55E-03	**3.59E-02**	2	NOTCH4, NOTCH4 (ICD4)
3	Development_Neurogenesis in general	192	1.00E-02	3.59E-02	1.00E-02	**3.59E-02**	2	NOTCH4, Notch
4	Development_Blood vessel morphogenesis	228	1.40E-02	3.59E-02	1.40E-02	**3.59E-02**	2	NOTCH4, Notch
5	Signal transduction_NOTCH signaling	236	1.49E-02	3.59E-02	1.49E-02	**3.59E-02**	2	NOTCH4, NOTCH4 (ICD4)

Enrichment by GO Processes

					overlap between non-LS and blood.cd4/cd8 SNPs			
#	Processes	Total	pValue	Min FDR	p-value	FDR	In Data	Network Objects from Active Data
1	regulation of cell adhesion	953	1.48E-07	1.87E-04	1.48E-07	**1.87E-04**	6	MHC class II alpha chain, BTNL2, RAGE, HLA-DRA1, Notch, Tenascin-X
2	positive regulation of cell adhesion	576	5.68E-07	3.59E-04	5.68E-07	**3.59E-04**	5	MHC class II alpha chain, BTNL2, RAGE, HLA-DRA1, Tenascin-X
3	positive regulation of immune system process	1334	1.09E-06	3.98E-04	1.09E-06	**3.98E-04**	6	MHC class II alpha chain, BTNL2, RAGE, HLA-DRA1, Notch, G18
4	positive regulation of T cell activation	324	2.82E-06	3.98E-04	2.82E-06	**3.98E-04**	4	MHC class II alpha chain, BTNL2, RAGE, HLA-DRA1
5	positive regulation of homotypic cell-cell adhesion	330	3.04E-06	3.98E-04	3.04E-06	**3.98E-04**	4	MHC class II alpha chain, BTNL2, RAGE, HLA-DRA1
6	protein-carbohydrate complex assembly	8	3.09E-06	3.98E-04	3.09E-06	**3.98E-04**	2	MHC class II alpha chain, HLA-DRA1
7	protein-carbohydrate complex subunit organization	8	3.09E-06	3.98E-04	3.09E-06	**3.98E-04**	2	MHC class II alpha chain, HLA-DRA1
8	antigen processing and presentation of polysaccharide antigen via MHC class II	8	3.09E-06	3.98E-04	3.09E-06	**3.98E-04**	2	MHC class II alpha chain, HLA-DRA1
9	polysaccharide assembly with MHC class II protein complex	8	3.09E-06	3.98E-04	3.09E-06	**3.98E-04**	2	MHC class II alpha chain, HLA-DRA1
10	positive regulation of leukocyte cell-cell adhesion	333	3.15E-06	3.98E-04	3.15E-06	**3.98E-04**	4	MHC class II alpha chain, BTNL2, RAGE, HLA-DRA1

F. Non-LS-associated variants in BAL CD4/CD8 ratio of non-LS cases

Enrichment by Pathway Maps					overlap between non-LS and bal.cd4/cd8 SNPs			
#	Maps	Total	pValue	Min FDR	p-value	FDR	In Data	Network Objects from Active Data
1	Immune response_Antigen presentation by MHC class II	12	1.27E-03	1.27E-03	1.27E-03	**1.27E-03**	1	HLA-DRA1

Enrichment by Process Networks					overlap between non-LS and bal.cd4/cd8 SNPs			
#	Networks	Total	pValue	Min FDR	p-value	FDR	In Data	Network Objects from Active Data
1	Inflammation_IL-4 signaling	115	1.61E-02	3.40E-02	1.61E-02	**3.40E-02**	1	HLA-DRA1
2	Immune response_Antigen presentation	197	2.76E-02	3.40E-02	2.76E-02	**3.40E-02**	1	HLA-DRA1
3	Immune response_Phagosome in antigen presentation	243	3.40E-02	3.40E-02	3.40E-02	**3.40E-02**	1	HLA-DRA1

Enrichment by GO Processes					overlap between non-LS and bal.cd4/cd8 SNPs			
#	Processes	Total	pValue	Min FDR	p-value	FDR	In Data	Network Objects from Active Data
1	T cell costimulation	105	9.88E-08	7.63E-06	9.88E-08	**7.63E-06**	3	HLA-DQA1, MHC class II alpha chain, HLA-DRA1
2	lymphocyte costimulation	106	1.02E-07	7.63E-06	1.02E-07	**7.63E-06**	3	HLA-DQA1, MHC class II alpha chain, HLA-DRA1
3	interferon-gamma-mediated signaling pathway	107	1.05E-07	7.63E-06	1.05E-07	**7.63E-06**	3	HLA-DQA1, MHC class II alpha chain, HLA-DRA1
4	antigen processing and presentation of exogenous peptide antigen via MHC class II	136	2.16E-07	7.63E-06	2.16E-07	**7.63E-06**	3	HLA-DQA1, MHC class II alpha chain, HLA-DRA1
5	antigen processing and presentation of peptide antigen via MHC class II	138	2.26E-07	7.63E-06	2.26E-07	**7.63E-06**	3	HLA-DQA1, MHC class II alpha chain, HLA-DRA1
6	antigen processing and presentation of peptide or polysaccharide antigen via MHC class II	141	2.41E-07	7.63E-06	2.41E-07	**7.63E-06**	3	HLA-DQA1, MHC class II alpha chain, HLA-DRA1
7	protein-carbohydrate complex subunit organization	8	3.32E-07	7.63E-06	3.32E-07	**7.63E-06**	2	MHC class II alpha chain, HLA-DRA1
8	protein-carbohydrate complex assembly	8	3.32E-07	7.63E-06	3.32E-07	**7.63E-06**	2	MHC class II alpha chain, HLA-DRA1
9	polysaccharide assembly with MHC class II protein complex	8	3.32E-07	7.63E-06	3.32E-07	**7.63E-06**	2	MHC class II alpha chain, HLA-DRA1
10	antigen processing and presentation of polysaccharide antigen via MHC class II	8	3.32E-07	7.63E-06	3.32E-07	**7.63E-06**	2	MHC class II alpha chain, HLA-DRA1
